# Comprehensive Structure–Activity Relationship Studies of Cepafungin Enabled by Biocatalytic C–H Oxidations

**DOI:** 10.1021/acscentsci.2c01219

**Published:** 2023-01-27

**Authors:** Alexander Amatuni, Anton Shuster, Daniel Abegg, Alexander Adibekian, Hans Renata

**Affiliations:** † Skaggs Doctoral Program in the Chemical and Biological Sciences, Scripps Research, La Jolla, California 92037, United States; ‡ Department of Chemistry, University of Illinois at Chicago, Chicago, Illinois 60607, United States; § Department of Chemistry, BioScience Research Collaborative, Rice University, Houston, Texas 77005, United States

## Abstract

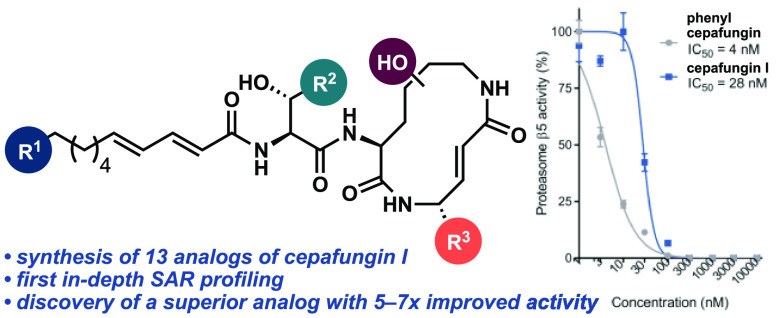

The cepafungins are a class of highly potent and selective eukaryotic proteasome inhibitor natural products with potential to treat refractory multiple myeloma and other cancers. The structure–activity relationship of the cepafungins is not fully understood. This Article chronicles the development of a chemoenzymatic approach to cepafungin I. A failed initial route involving derivatization of pipecolic acid prompted us to examine the biosynthetic pathway for the production of 4-hydroxylysine, which culminated in the development of a 9-step synthesis of cepafungin I. An alkyne-tagged analogue enabled chemoproteomic studies of cepafungin and comparison of its effects on global protein expression in human multiple myeloma cells to the clinical drug bortezomib. A preliminary series of analogues elucidated critical determinants of potency in proteasome inhibition. Herein we report the chemoenzymatic syntheses of 13 additional analogues of cepafungin I guided by a proteasome-bound crystal structure, 5 of which are more potent than the natural product. The lead analogue was found to have 7-fold greater proteasome β5 subunit inhibitory activity and has been evaluated against several multiple myeloma and mantle cell lymphoma cell lines in comparison to the clinical drug bortezomib.

## Abstract 2

13 cepafungin I analogues were prepared for comprehensive structure−activity relationship study. The best analogue has in vitro cytotoxicity similar to bortezomib in several cancer cell lines.

## Introduction

**Figure 1:**
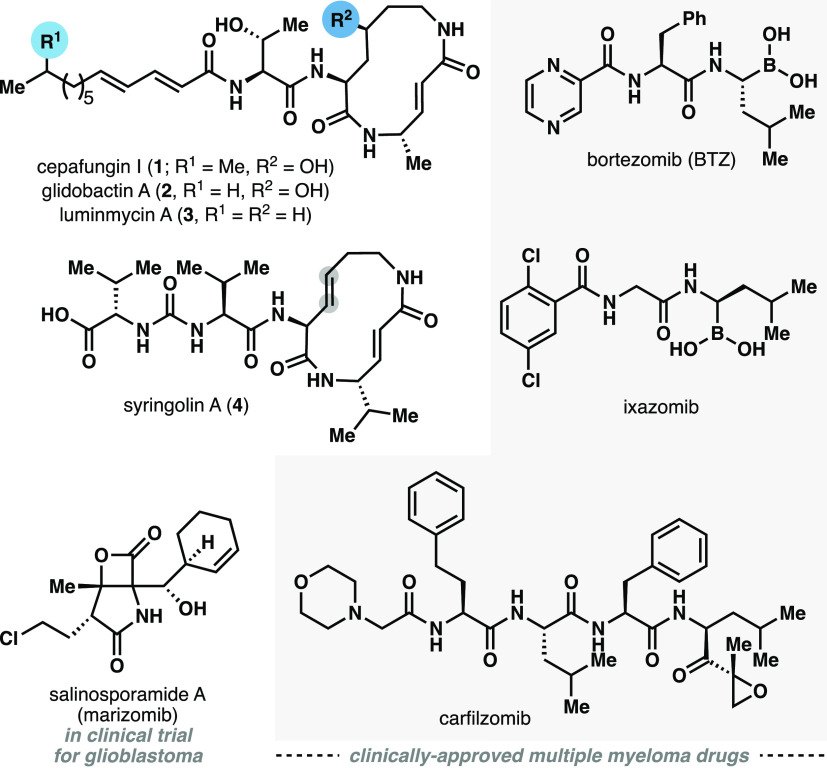
Representative structures of syrbactin natural products and clinical proteasome inhibitors.

Cepafungin I is the most potent member of a peptide–polyketide hybrid natural product family known as the syrbactins. All syrbactins share a 12-membered macrolactam core built from a side chain-cyclized lysine residue with or without (*S*)-4-hydroxylation, or with C3–C4 unsaturation as seen in syringolin A. The macrocycle also contains an α,β-unsaturated amide that serves as a Michael acceptor toward the catalytically active N-terminal threonines of proteasome subunits β1 (caspase-like), β2 (trypsin-like), and β5 (chymotrypsin-like) (Figure 1).^1^ The syrbactins have garnered significant interest for their irreversible covalent inhibition of the 20S proteasome core particle, which is implicated in cell cycle regulation and apoptosis and is highly dysregulated in multiple myeloma. Currently, the three proteasome inhibitors (PIs) bortezomib, carfilzomib, and ixazomib are approved by the FDA for the treatment of multiple myeloma (MM). It is noteworthy that carfilzomib is a derivative of the natural product epoxomicin, and the natural product salinosporamide A is currently in clinical trials for glioblastoma,^2^ highlighting the viability of natural-product-based proteasome inhibitors as cancer chemotherapies (Figure 1). However, chemoresistance and other side effects remain a significant challenge for currently approved PIs.^3^ While these drugs primarily inhibit the β5 subunit, potent coinhibition of both β2 and β5 subunits has been indicated as a promising strategy to overcome chemoresistance toward PIs in MM and enhance activity toward solid tumors.^4^ Considering that cepafungin I is the most potent PI among the syrbactins and has low-nanomolar inhibitory activity toward both β2 and β5 subunits, this natural product serves as a promising scaffold for further structure–activity relationship (SAR) optimization toward a new anticancer agent.

Syntheses of syringolins A (**4**) and B have been reported by Kaiser,^5^ Stephenson,^6^ Pirrung,^7^ and Ichikawa,^8^ and Schmidt in 1992 achieved the first total synthesis of glidobactin A.^9^ Early SARs were established with semisynthetic glidobactin analogues harboring unnatural tail fragments, though the syrbactins’ mechanism of action had not been elucidated at the time.^10^ More recent studies on the syringolins and unnatural analogues have elucidated their activity in the context of proteasome inhibition. Collectively, these studies have revealed some key factors contributing to the syrbactins’ potent bioactivity. The presence of longer lipophilic tails seen in glidobactins, cepafungins, and synthetic analogues versus those of natural syringolins has been shown to result in greater proteasome inhibition in vitro, likely by increased hydrophobic interactions distal to the active site.^8,11^ Notably, a single methyl branching at the end of the cepafungin I (**1**) fatty acid leads to ca. 5-fold improvement of β5-subunit inhibition compared to glidobactin A (**2**).^12^ Further exploration of hydrophobic functionalities at this position may further improve the proteasome binding affinity at one or more subunits. Larger hydrophobic residues in place of the macrocycle-adjacent threonine are tolerated by the S3 subsite and can improve binding affinity.^8^ However, other β-hydroxy amino acids in place of threonine have not been explored, nor has there been a direct comparison between threonine and its nonhydroxylated counterpart. Interestingly, several bacteria produce glidobactin-like syrbactins, and several other bacteria have bioinformatically been shown to harbor syrbactin-like gene clusters.^13,14^ While several lysine hydroxylases are found in nature, syrbactins with different lysine hydroxylation patterns have not been found, except for minor amounts of desoxy-glidobactins known as “luminmycins” from *Photorhabdus laumondii*, such as luminmycin A (**3**).^15^ Given the cepafungins’ potent bioactivity and the current gap in SAR data, we sought to develop a versatile and modular strategy that would allow concise and scalable access to cepafungin and analogues harboring modifications at all regions of the scaffold. This full report traces the development of such strategy. Unique in our approach is the utilization of several enzymes for site-selective C–H hydroxylation of amino acids, facilitating rapid exploration of chemical space around the cepafungin macrocycle and tail fragment linker. This approach has resulted in the first comprehensive SAR analysis of cepafungin’s proteasome inhibitory activity and five unnatural analogues with improved bioactivity, one of which exhibits comparable cytotoxicity to the clinically approved drug bortezomib.

## Results and Discussion

### Initial Synthetic Approach Involving Pipecolic Acid Derivatization

**Scheme 1:**
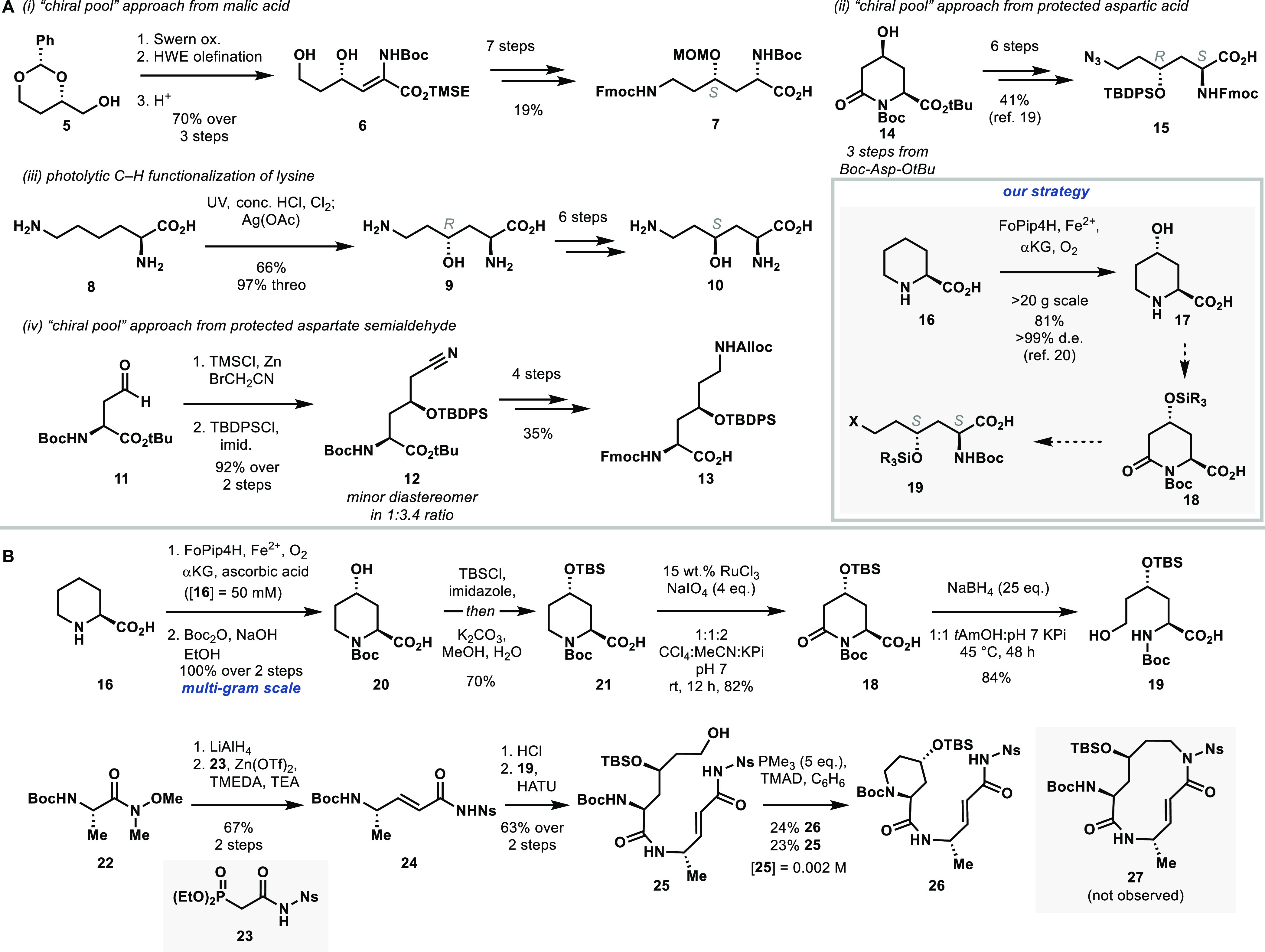
(A) Prior Approaches to Diastereomers of 4-Hydroxylysine and (B) First-Generation Synthetic Strategy toward Cepafungin See the Supporting Information for experimental details.

Key challenges in the synthesis of cepafungins and glidobactins are the construction of the (2*S*,4*S*)-4-hydroxylysine residue present in the macrocycle, and the identification of an efficient method for the 12-membered macrolactam construction (Figure 1). The first total synthesis of a syrbactin harboring macrocycle hydroxylation, glidobactin A, was achieved in 21 steps where 12 steps were required to synthesize a suitably protected (2*S*,4*S*)-4-hydroxylysine from l-malic acid.^9^ This approach includes Horner–Wadsworth–Emmons olefination with a glycine-derived phosphonate and asymmetric hydrogenation with rhodium catalyst and expensive phosphine ligand to establish the α-stereocenter in moderate yield (Scheme 1A). A further 5 steps of functional and protecting group manipulations provide **7** for subsequent acylation with an alanine derivative. In this route, macrolactamization via a pentafluorophenyl ester of the linear dipeptide was ultimately achieved in 20% yield. An earlier semisynthesis of glidobactin achieved a 2.3% cyclization yield for the alternate amide formation between lysyl α-CO_2_H and alanyl α-NH_2_.^16^ Earlier reports of direct C–H functionalization of free l-lysine **8** proceed through a harsh photochlorination using concentrated acids and excess chlorine gas, followed by treatment with excess silver acetate to hydrolyze the intermediate 4-chlorolysine.^17^ However, this process yields primarily the (2*S*,4*R*)-4-hydroxylysine diastereomer **9**, requires six steps to access the (2*S*,4*S*) diastereomer present in syrbactins, and proceeds through the lactones of both diastereomers.^18^ Generally, 4-hydroxylysines are prone to lactonization upon N-protection, thereby requiring secondary alcohol protection to facilitate subsequent coupling steps. A more recent approach employed a Reformatsky condensation of l-aspartic acid semialdehyde **11** and bromoacetonitrile, giving a mixture of (2*S*,4*S*) and (2*S*,4*R*) diastereomers.^19^ While this reaction favors the correct configuration of 4-hydroxylysine for use in syrbactin synthesis, an additional 4 steps of functional group interconversion and protecting group manipulation are needed to access differentially protected **13**. Finally, an approach by Marin et al. proceeded through an oxidized l-pipecolic acid derivative **14** that was built from protected l-aspartic acid in three steps. Reductive cleavage of the piperidine ring C6–N_α_ amide bond and further elaboration provided a functional equivalent of (2*S*,4*R*)-4-hydroxylysine **15**.^20^ However, this approach selectively provides the (2*S*,4*R*) diastereomer which would be unsuitable for cepafungin synthesis.

Our initial approach drew inspiration from Marin’s strategy by targeting the C4 diastereomer of **14**. We noted that a family of fungal iron and α-ketoglutarate dependent dioxygenases (Fe/αKG’s) have been functionally characterized to perform a *trans*-C4 hydroxylation of l-pipecolic acid that could then provide the correct diastereomer of 4-hydroxylysine upon piperidine ring cleavage (Scheme 1A).^21^ FoPip4H was shown to have the highest activity for this hydroxylation and gives the product as a single diastereomer on decagram scales when heterologously expressed in *Escherichia coli*. We sought to merge this enzymatic transformation with subsequent chemical manipulations to ultimately cleave the C6–N_α_ bond of **18** and provide a functional equivalent of (2*S*,4*S*)-4-hydroxylysine in the form of **19**. This fragment would comprise half of the cepafungin macrocycle. The other macrocycle fragment would be prepared from an alanine derivative to introduce the unsaturated amide moiety through simple acylation. In lieu of macrocyclization by amide formation, with typically <30% yields reported in prior syrbactin syntheses, we planned to form the 12-membered ring by an intramolecular Fukuyama–Mitsunobu N-alkylation between an alanine-derived enamide nitrogen and the terminal alcohol of **19**. Though we acknowledged the possibility of a competing intramolecular cyclization pathway to reform the piperidine ring, such direct displacement of primary alcohols by nosylamides has enabled the efficient synthesis of medium-size rings and would avoid additional functional group manipulations after C6–N_α_ cleavage of **18** for the traditional macrolactamization strategy.^22^

Our first-generation synthesis of the cepafungin macrocycle thus began with FoPip4H-mediated oxidation of l-pipecolic acid **16** (Scheme 1B). FoPip4H was heterologously overexpressed in *E. coli* as an N-His_6_-tagged protein and was directly used as the clarified cell lysate in multigram scale reactions with prelysis OD_600_ = 30. Hydroxylation was followed by in situ Boc protection to provide **20** in quantitative yield over two steps. TBS protection of the secondary alcohol concomitantly formed the silyl ester which was cleaved in situ with methanol.^23^ Oxidation at C6 was achieved with catalytic ruthenium(III) chloride and excess sodium periodate as stoichiometric oxidant to provide **18** in 82% yield.^24^ Subsequent reductive cleavage of the C6–N_α_ bond was attempted with the NaBH_4_/EtOH conditions reported for the (2*S*,4*R*)-diastereomer,^20^ but resulted in trace product formation by LCMS with either **18** or the corresponding tert-butyl ester as substrate. Other silyl protecting groups for the secondary alcohol of **18**, such as TBDPS and TIPS, also failed to promote lactam cleavage under these conditions. Other reducing agents such as LiBH_4_, LiEt_3_BH, and LiNH_2_BH_3_^25−27^ primarily resulted in substrate decomposition. Solvent screening with NaBH_4_ as reductant indicated that *tert*-amyl alcohol (*t*AmOH) provided greater conversion to **19**. However, we observed a significant amount of an over-reduced byproduct with solely NaBH_4_ and *t*AmOH in the reaction mixture, likely arising from silyl ether elimination under basic conditions. Buffering the reaction with 1 M pH 7 phosphate and portion-wise addition of 25 equiv of NaBH_4_ over 48 h with mild heating was found to produce the desired amino alcohol **19** in high yield, thus completing half of the cepafungin macrocycle. Synthesis of the *para*-nitrobenzenesulfonyl (Ns) activated enamide for Fukuyama–Mitsunobu alkylation began with protected alanine Weinreb amide **22**. LAH reduction followed by a modified Horner–Wadsworth–Emmons olefination^28^ with nosylamide phosphonate **23** produced the desired *trans*-alkene **24**. Deprotection and acylation with **19** completed the fully protected linear macrocycle precursor **25**. Initial attempts at Mitsunobu macrocyclization using PPh_3_ and DEAD led to complete decomposition. Preforming the betaines with PPh_3_/DEAD or DIAD resulted in minimal conversion. However, preforming the betaine of the more reactive phosphine PMe_3_ and TMAD resulted in the competing six-membered species **26** as the major product arising from Boc-N_α_ cyclization, with a similar amount of recovered starting material. The desired 12-membered ring **27** was not observed under any conditions (Table S1). The structure of **26** was confirmed by comparison to a sample prepared by coupling of **21** and deprotected **24**. The failure to obtain the 12-membered macrolactam led us to reconsider our approach. While a workaround involving installation of a terminal azide on the 4-hydroxylysine surrogate and Staudinger macrolactamization could be envisioned, the need for extensive functional group manipulation dissuaded us from pursuing this alternative. We next turned to a macrolactamization strategy with the hope that a newer peptide coupling reagent would overcome the historically low yields for cyclization. Additionally, we sought to shorten the total synthesis by directly hydroxylating free l-lysine, taking note of a putative Fe/αKG annotated in the biosynthetic gene cluster of glidobactin A.^13^

### Characterization of GlbB, a Lysine 4-Hydroxylase from Glidobactin Biosynthesis and Its Use in the Synthesis of Cepafungin

**Scheme 2:**
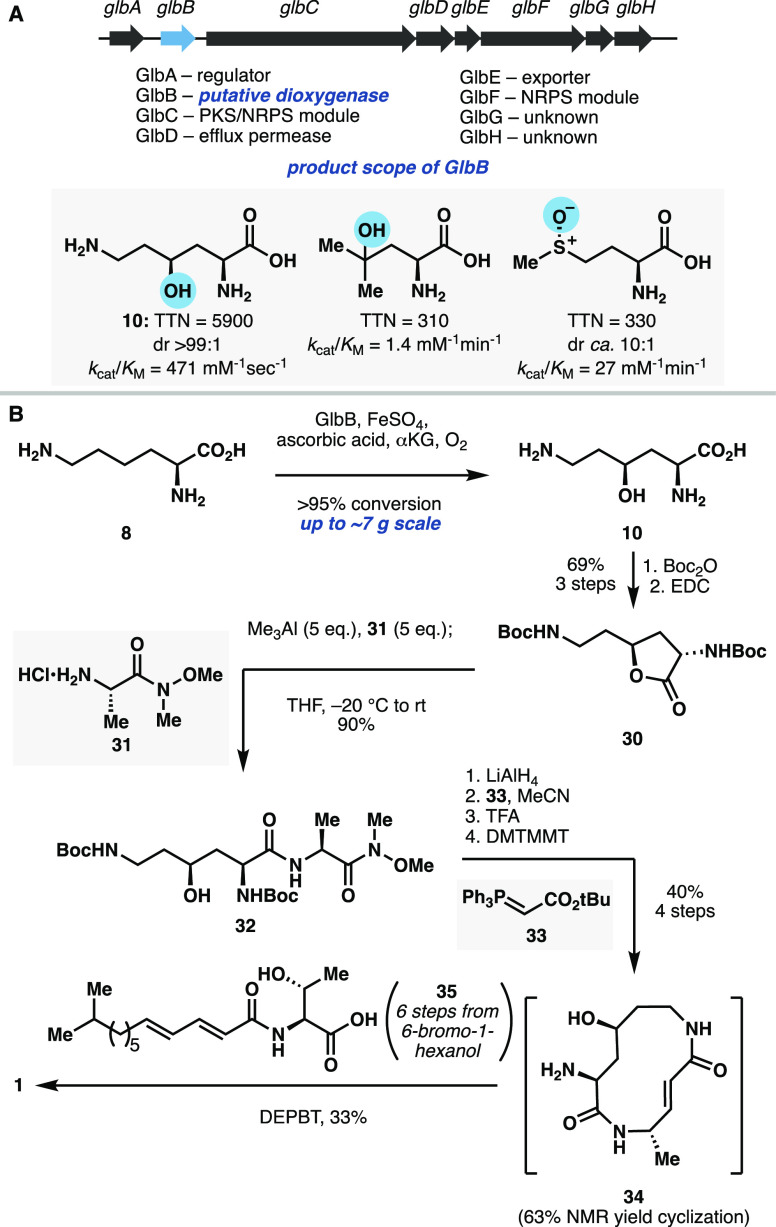
(A) Annotation of Glidobactin Biosynthetic Gene Cluster and Product Scope of GlbB and (B) Optimized Chemoenzymatic Route to **1**

Seminal studies on the biosynthesis of syrbactins suggested that the biosynthetic gene clusters for the production of syringolins and glidobactin-type natural products are similar across several bacterial genomes.^13−15,29^ In the case of syringolin, the five genes *sylA–E* are solely responsible for producing syringolin A from the phytopathogen *Pseudomonas syringae* pv *Syringae*, including the 3,4-dehydrolysine moiety and the unusual bis-ureido-valine motif. The origin of 3,4-dehydrolysine seen in syringolin A likely arises from the action of SylB, a putative desaturase. Alternatively, the presence of a canonical lysine residue in the macrocycle of syringolin B results from relaxed substrate specificity of the SylC nonribosomal peptide synthetase (NRPS) module and possibly inefficient lysine desaturation by SylB.^30^ The Dudler group later obtained the biosynthetic gene cluster for glidobactin from a soil bacterium of *Burkholderiales* order, which is composed of the eight genes *glbA–H* (Scheme 2A).^13^ Similar to *sylD* in the syringolin biosynthesis, *glbC* encodes a hybrid of two NRPS modules and one polyketide synthase (PKS) module. Together with *glbF*, an NRPS module responsible for N-acylation of the Thr residue with fatty acyl-CoA donors,^31^*glbC* produces the final cyclized cepafungin structure. *glbA*, *glbD*, and *glbE* are annotated to encode for regulator, efflux permease, and exporter proteins, respectively. Between *glbH* and *glbB*, a disruption mutant of the former led to greatly reduced glidobactin production, while the *glbB* mutant did not produce any glidobactin. Furthermore, a syrbactin synthetase from *Photorhabdus luminescens* containing homologues of only *glbB–G* was able to produce glidobactin A. While *glbB* was initially annotated as a conserved protein within DUF2257, we noted that this domain was later found to be a family of Fe/αKG enzymes (PF10014) capable of hydroxylating free amino acids.^32^

Homology modeling of GlbB against a solved crystal structure template of a PF10014 member from *Methylibium petroleiphilum* indicated the hallmark Fe/αKG metal binding triad His^1^-X-Asp-X_*n*_-His,^2^ composed of residues His188, Asp190, and His251. Arg267 likely facilitates αKG-binding opposite to this triad. Several polar amino acids also likely form a hydrophilic pocket and appear poised to form polar contacts with an amino acid substrate. Thus, GlbB was seen as the most likely candidate responsible for lysine hydroxylation, prompting us to heterologously overexpress GlbB in *E. coli* as an N_6_–His-tagged protein for purification and in vitro assays. Routine functional characterization established GlbB as an l-lysine hydroxylase. Upon scale-up, 0.02 mol % of purified GlbB performed full conversion of 0.6 mmol of **8** to provide **10** as a single diastereomer with 5900 total turnover number (TTN) (Scheme 2A). Interestingly, GlbB was also able to hydroxylate free l-leucine and l-methione to provide **28** and **29**, respectively, albeit with substantially lower catalytic efficiencies.^33^ Following this result, we sought to employ GlbB in a revised chemoenzymatic synthesis of cepafungin I starting from l-lysine hydroxylation.

The scale-up hydroxylation of lysine was carried out with the clarified lysate of *E. coli* overexpressing GlbB and chaperone proteins GroES/GroEL for increased soluble expression. Full conversion could be achieved with up to ∼7 g of lysine at 40 mM substrate loading and prelysis OD_600_ = 25 (Scheme 2B). Initially, the free amino acid product **10** was isolated by cation exchange resin, as we intended to introduce orthogonal amine protecting groups for eventual macrolactamization with its ε-NH_2_. The first strategy commonly used for differential lysine protection involves chelation of the α-NH_2_ and α-CO_2_H groups with copper(II) salts, which provides an insoluble bis-lysine species that can be easily isolated (Scheme S1A).^34^ Subsequent protection of the ε-NH_2_, followed by EDTA-mediated dechelation, then allows for α-NH_2_ protection. In our hands, with pure **10** this protocol failed to precipitate the desired chelated species **S4**, possibly due to the increased polarity of hydroxylysine or mixed modes of chelation involving the secondary alcohol. Further attempts at one-pot/four-step differential protection by this protocol resulted in complex mixtures of mono- and bis-protected hydroxylysines and their corresponding lactones. An alternate strategy involves selectively forming the Schiff base of anisaldehyde with the more basic ε-NH_2_ of lysine at low temperature. Protection of the free α-NH_2_ followed by imine hydrolysis then allows for differential ε-NH_2_ protection (Scheme S1B).^34,35^ With **10**, however, the Schiff base intermediate **S6** was not isolable as the expected solid from the condensation mixture. In turn, attempts at a one-pot/four-step sequence again resulted in an intractable mixture of mono- and bis-protected products and their lactone counterparts.

Considering these results, global Boc protection was performed directly in the GlbB reaction supernatant, which resulted in partial lactonization. Subsequent peptide coupling would require the open chain hydroxy-acid, but carbonyl activation would facilitate rapid lactonization and prevent the desired coupling. Indeed, treating the crude Boc-protected mixture of **10** with EDC gave rapid and complete conversion to lactone **30** in 69% yield over three steps (Scheme 2B).^36^ This lactone comprises a functional equivalent of (2*S*,4*S*)-4-hydroxylysine suitable for coupling by way of lactone aminolysis with an alanine derivative. Initial attempts at aminolysis with l-alaninol successfully provided the desired dipeptide in high yield, but this strategy was hindered by poor chemoselectivity and efficiency in subsequent primary-selective alcohol oxidation conditions (Scheme S2, Table S2).^33^ An alternative strategy was devised to utilize the Weinreb amide of l-alanine as an aldehyde surrogate. Initial attempts at aminolysis were beset by harshly basic and high-temperature conditions that led to Boc-deprotection, epimerization, relactonization, or polymerization of lactone **30**. Ultimately, amine **31** was found to form a stable aluminum amide reagent upon treatment with AlMe_3_ that did not self-polymerize with its Weinreb amide functionality. These conditions enabled the lactone aminolysis to proceed at low temperature and with very high efficiency, giving the desired dipeptide **32** in 90% yield. Subsequent reduction to the aldehyde and Wittig olefination provided the fully protected linear macrocycle precursor. Global deprotection proceeded cleanly, and the diamino acid product was screened against several peptide coupling reagents for macrocyclization. DMTMMT was found to form the desired macrolactam **34** most efficiently, as judged by an NMR yield of ∼60% compared to 0–40% for several other common reagents. This macrolactamization has been successfully performed on gram scale with similar yields. Finally, the tail fragment **35** was prepared in 6 steps, starting from a Kochi coupling of isopropyl Grignard with 6-bromo-1-hexanol. Final fragment coupling with the crude macrocycle completed the synthesis of cepafungin I in a total of 9 longest linear steps and 7.9% overall yield. Importantly, this route builds the natural product modularly and has been readily scaled up to provide ≥40 mg of final compound, which is sufficient for cell-based assays and in vivo mouse studies for toxicity and DMPK evaluation.

GlbB was thus established as a privileged biocatalyst for concise synthesis of cepafungin I. While cepafungin and related syrbactins had been shown to inhibit β2 and β5 subunits of purified yeast and mammalian proteasomes, this activity had not been directly observed in mammalian cells at the time of our work. To determine the targets of cepafungin I in human MM cells through chemoproteomics, a clickable analogue (**S10**) was designed with alkyne functionality distal to the reactive macrocycle (i.e., at the end of the fatty acid chain). The synthesis of **S10** proceeded in a similar fashion as in Scheme 2B except with the tail fragment built from initial alkyne substitution of 6-bromo-1-hexanol. Gel-based competitive profiling in MM cell line RPMI 8226 revealed that very few **S10**-labeled bands were competed by **1** at nanomolar concentrations, indicating that cepafungin is indeed highly potent and selective toward its cellular targets. To identify these targets, a competitive in situ LC-MS/MS-based pull-down experiment was performed using alkyne probe **S10** and **1**. Following live cell treatment with just the probe or the probe together with the natural product, alkyne conjugation to biotin-azide and pull-down with streptavidin beads, the probe analogue enriched 764 proteins from RPMI 8226. Surprisingly, only 5 of these proteins were competed >50% with 100 nM **1**. These targets were identified as the 20S proteasome subunits β5, β2i, α5, β1, and β2, highlighting cepafungin’s exceptional selectivity for the proteasome in MM cells (Figure S1 and Table S3). Notably, **1** competes for subunits β2/2i, β5/5i, and β1i at low-nanomolar concentrations, and shows very little engagement of β1 even at high concentrations. The “i” suffix here denotes subunits of the immunoproteasome, which is produced by most cells exposed to oxidative stress or proinflammatory cytokines and also by immune cells at especially high baseline levels.^37^ Targeting the immunoproteasome has been highlighted as a promising strategy in MM treatment.^38^ In contrast to the constitutively expressed β1 subunits (caspase-like), β1i subunits display largely chymotrypsin-like (β5) activity. Therefore, the inhibition pattern seen by in-gel labeling with **1** corresponds to potent inhibition of both chymotrypsin-like (β5/5i/1i) and trypsin-like (β2/2i) activities at low-nanomolar concentrations. This reactivity pattern differs from that of bortezomib (Figure 1), a slowly reversible PI that engages chymotrypsin-like (β5/5i/1i) and caspase-like (β1) subunits.^39,40^ Carfilzomib, the second FDA-approved PI for relapsed/refractory MM (RRMM), inhibits primarily the chymotrypsin-like (β5) activity in an irreversible fashion.^41^ Ixazomib, the latest FDA-approved treatment for RRMM, reversibly inhibits primarily β5 activity and at higher concentrations the β1 activity, and very little of the β2 activity.^42^ Potent inhibition of both β5 and β2 subunits has been shown to increase PI cytotoxicity and prevent recovery of proteasome activity by a compensatory hyperactivation mechanism. Such coinhibition also sensitizes triple-negative breast cancer and other solid tumors to bortezomib and carfilzomib while neither have been effective on their own.^4^ At the same time, **1** displays a subunit engagement pattern more like that of salinosporamide A, which irreversibly targets β5 and β2 subunits, and β1 to a lesser extent. Salinosporamide A has thus far shown a unique efficacy and safety profile in MM and other cancers and does not exhibit cross-resistance with other PIs.^41^ Taken together, these observations highlight the potential of cepafungin and derivatives thereof to serve as novel PIs with unique pharmacological properties and the potential to overcome chemoresistance.

To gain a clearer picture of cepafungin’s downstream cellular effects, a global proteomics experiment compared **1** and bortezomib in RPMI 8226 cells. A total of ∼3700 proteins were quantified by LC-MS/MS-based peptide analysis, where 88 proteins were upregulated upon treatment with **1**, and 25 were upregulated upon treatment with bortezomib compared to DMSO control. Of the latter 25 targets, 19 were in common with the **1**-treated samples, suggesting a similar mechanism of action between **1** and bortezomib. The overlap in upregulated proteins between **1** and bortezomib may be further increased by accounting for factors such as differential cell permeability, binding kinetics, and metabolic stability. Nevertheless, some of this difference in upregulation may be caused by cepafungin’s unique proteasome subunit inhibition profile and warrants further investigation in future studies. Following these initial chemoproteomic studies, we sought to synthesize a short series of analogues to establish preliminary SAR data for the cepafungins in MM cells for the first time.

### Analogue Synthesis and Structure–Activity Relationship Studies

**Figure 2:**
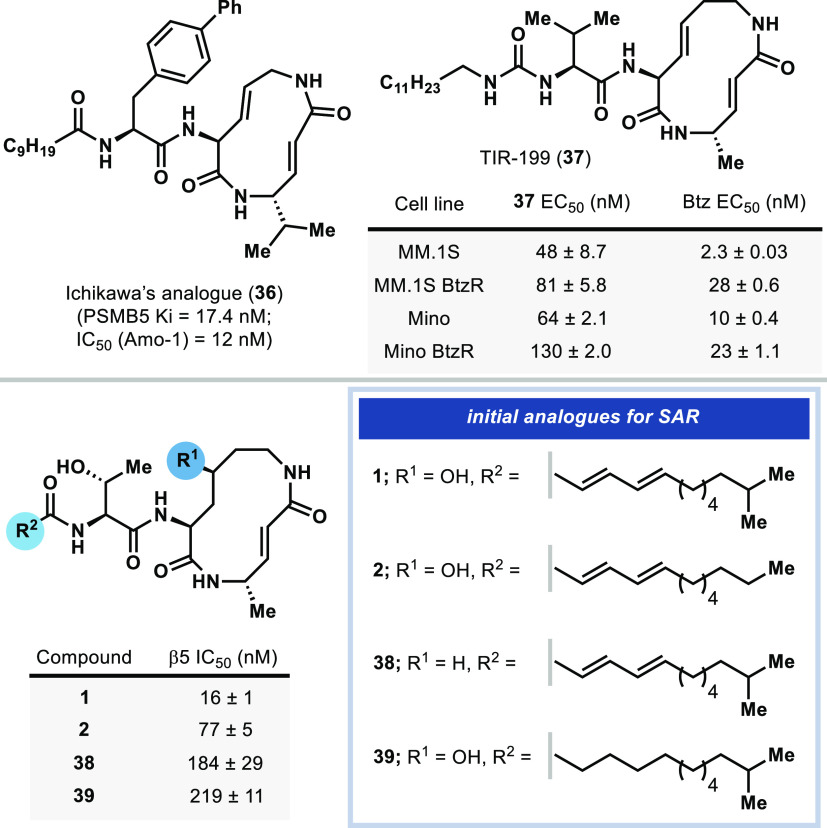
Preliminary series of cepafungin analogues and their cytotoxicities and comparison to prior syringolin analogues.

Early syntheses of the syringolins ultimately led to the development of various lipophilic analogues with improved cytotoxicity and proteasome inhibition. Ichikawa’s analogue **36** and Pirrung’s analogue **37** both benefited from replacement of one or more C-terminal Val residues in the syringolin A tail (Figure 2).^8,43^ Ichikawa reported that a Phe macrocycle linker was found to increase β5 inhibition 15-fold compared to syringolin A, likely by improved binding at the hydrophobic pocket of the S3 subsite, although this analogue still exhibited low cytotoxicity. Exploration of cell-permeable lipid tails led to the discovery of analogue **36** which exhibited low-nanomolar cytotoxicity in MM cell lines. Likewise, Pirrung reported a syringolin A analogue **37** containing a dodecanoyl-ureido-Val tail fragment. This analogue had inhibitory activity comparable to ixazomib and displays lower resistance index in MM cell lines than bortezomib and prolonged survival in MM mouse xenografts. However, prior SAR studies on syrbactins have not explored analogues that are more cepafungin-/glidobactin-like.

A preliminary series of analogues, including the natural product glidobactin A (**2**), were synthesized by largely the same route (Scheme 2B, Figure 2).^36^ Desoxycepafungin (**38**) was synthesized from Boc-Lys(Boc)-OH by peptide couplings/macrocyclization to assess the role of the macrocycle hydroxylation in **1** toward proteasome inhibition. The saturated cepafungin analogue **39** was designed to address the possible role of rigidity in the tail fragment induced by the diene seen in many natural cepafungins and glidobactins. An analogue bearing unsaturation at its macrocyclic lysine was envisioned to provide a head-to-head comparison between the cepafungin and syringolin macrocyclic core. However, dehydration conditions on the hydroxylated macrocycle resulted in decomposition and inseparable mixtures of olefin isomers. Proteasome subunit β5 IC_50_ values were determined in situ in RPMI 8226 cells. In close agreement with prior measurement of β5 IC_50_ for natural **1** and **2** in purified yeast proteasome,^12^ in our hands the additional branching methyl in **1** indeed leads to ca. 5-fold improved IC_50_ compared to **2** in RPMI 8226 cells. Notably, the macrocyclic hydroxyl group in **1** leads to ca. 11-fold improved β5 inhibition compared to its “desoxycepafungin” analogue **38**. Likewise, saturation of the fatty acid fragment in **39** causes ca. 14-fold decrease in β5 inhibition. This preliminary series of analogues highlighted that the macrocyclic hydroxylation, fatty acid unsaturation, and terminal lipid functionality are critical for potent proteasome inhibitory activity.

**Figure 3:**
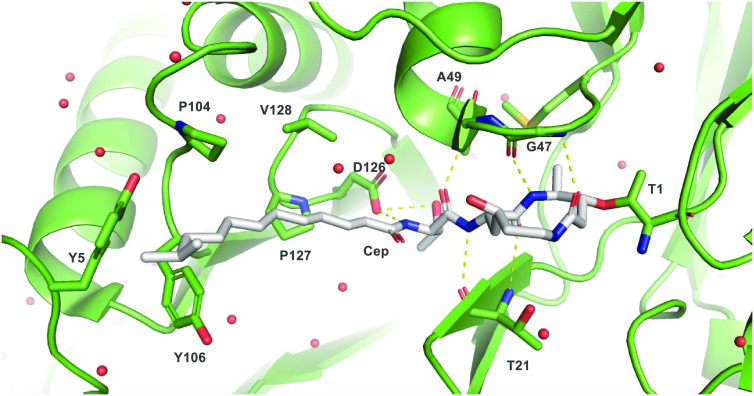
Bound crystal structure of cepafungin I at β5 subunit of yeast 20S proteasome. PDB ID: 4FZC.^11^ Dashed lines indicate polar contacts. The catalytic β5 N-terminal threonine covalently bonded to the cepafungin macrocycle is labeled “T1”. β5 residues involved in polar contacts are Gly47, Ala49, Thr21, and Asp126. A hydrophobic channel for the tail fragment involves β6 subunit residues Pro127, Val128, Pro104, Tyr106, and Tyr5.

To gain a more comprehensive understanding of cepafungin’s SAR, we designed a broader series of analogues guided by a previous crystal structure of proteasome:cepafungin complex^12^ (Figure 3), insights from prior SAR studies on related syrbactins,^8,10,11,44,45^ and our preliminary SAR data. In the absence of the structure of human proteasome:cepafungin complex, that of yeast proteasome:cepafungin complex was used as a reference as the two proteins are highly homologous. In total, four divergence points were identified, namely, the oxygenation pattern of the macrocycle, the vinylogous amino acid residue in the macrocycle, the side chain of the β-OH amino acid linker, and the terminal functionality of the lipid tail. Regarding the first point, we envisioned l-lysine (**8**) as the key divergence point in analogue generation (Scheme 2B). That desoxycepafungin (**38**) has ca. 11-fold higher IC_50_ than **1** just from omission of the lysine hydroxylation raises the question whether the reduced potency arises from a general decrease in polarity of the macrocycle or from a more specific electrostatic interaction in the active site. Notably, no hydrogen bonds or water molecules are seen near the hydroxy group of **1** and the β5 subunit of yeast proteasome in the crystal structure (Figure 3). To probe the relevance of this hydroxylation, we sought to compare analogues containing different lysine oxidation patterns. In addition to the aforementioned interactions, it is also possible that the introduction of non-natural oxidation patterns might induce alternative ring conformations that might lead to superior binding in the active site. In prior studies, the conformations of proteasome-bound syringolin B and glidobactin A were noted to be almost identical but showed marked differences to that of proteasome-bound syringolin A.^5a^ The (2*S*,4*R*) diastereomer of **10** could be prepared via KDO3, an Fe/αKG enzyme from *Flavobacterium johnsoniae* that performs regio- and stereoselective (*R*)-C4-hydroxylation on free lysine. Elaboration to the macrocycle would then follow the general route outlined in Scheme 2B. Additionally, the (2*S*,3*S*)-3-hydroxylysine regioisomer could be prepared with KDO1, an Fe/αKG enzyme from *Catenulispora acidiphila* that performs selective (*S*)-C3-hydroxylation on free lysine.^46^ As this monomer is not expected to undergo spontaneous lactonization upon amine protection, the protected 3-hydroxy amino acid could be directly coupled to an Ala derivative for elaboration to the macrocycle.

The second divergence point is the vinylogous amino acid residue in the macrocycle (Scheme 2B). The naturally occurring syringolins incorporate an l-Val residue in place of the l-Ala seen in the glidobactins and cepafungins, and in both cases these side chains occupy the S1 subsite at the β5 subunit. However, a direct comparison between cepafungin and the Val-substituted macrocycle analogue has yet to be reported. The third divergence point is the β-OH amino acid adjacent to the macrocycle, which for cepafungins and glidobactins is canonically occupied by an l-Thr residue. Late-stage introduction of this residue in the tail fragment synthesis facilitates the incorporation of other β-OH amino acids. Notably, the hydroxy group of Thr is involved in hydrogen bonding with Asp126 at the S3 subsite, which comprises a prominent pocket adjacent to the methyl group of Thr and can accommodate larger functional groups (Figure 3). Ichikawa’s syringolin analogues incorporated fatty-acyl-Phe residues in place of the canonical bis-ureido-Val (Figure 2) and resulted in greater proteasome inhibition.^8^ However, direct comparison of cepafungin I against analogues containing desoxy-Thr or other β-OH amino acids has not been performed. Such β-OH amino acids can be prepared by enzymatic or chemical methods.

Finally, our preliminary SAR results (Figure 2) indicate that the diene functionality in the fatty acid as well as the terminal methyl branching are very important for bioactivity. The diene may assist in orienting the lipid tail along a hydrophobic patch on the adjacent β6 subunit and between residues Pro127 and Val128 (Figure 3). Additionally, early cytotoxicity assays and more recent studies on glidobactin-like natural products highlight that C_12_ fatty acid chains in the tail fragment are the optimal length for potent bioactivity.^10,15^ Therefore, additional analogues were designed to keep the C_12_ chain length and the diene moiety but explore alternate groups in place of the terminal isopropyl in **1**. Substitutions can readily be made by variations of the Grignard reagent during the initial Kochi coupling to 6-bromo-1-hexanol. Notably, the cepafungin lipid terminates near β6 subunit residues Tyr5 and Tyr106, which appear poised to facilitate potential π-stacking interactions (Figure 3). Thus, we sought an analogue with a phenyl ring at the lipid terminus to induce π-stacking with either of these residues. Larger hydrophobic groups also include *tert*-butyl, cyclopentyl, and cyclohexyl. Additionally, as trifluoromethyl groups may prefer interactions with Phe, Met, Leu, and Tyr residues compared to the corresponding methyl-substituted compound,^47^ an analogue containing a CF_3_-substituted lipid fragment was sought. Direct adaptation of our original synthetic route to this analogue would require the introduction of a terminal hexafluoroisopropyl moiety by Wittig olefination.^48^ Due to operational hazards involved in working with highly volatile and toxic hexafluoroacetone, we opted to instead make the CF3-substituted lipid fragment via photocatalytic trifluoromethylation of 5-hexen-1-ol,^49^ followed by further elaboration based on our synthetic route to cepafungin. Taken together, this expanded series of analogues spans modifications of the macrocycle, tail fragment β-OH amino acid, and fatty acid fragments. Importantly, the modularity of our synthesis would readily allow combinations of modified building blocks after initial screening of monosubstitutions to identify any synergistic effects.

**Scheme 3:**
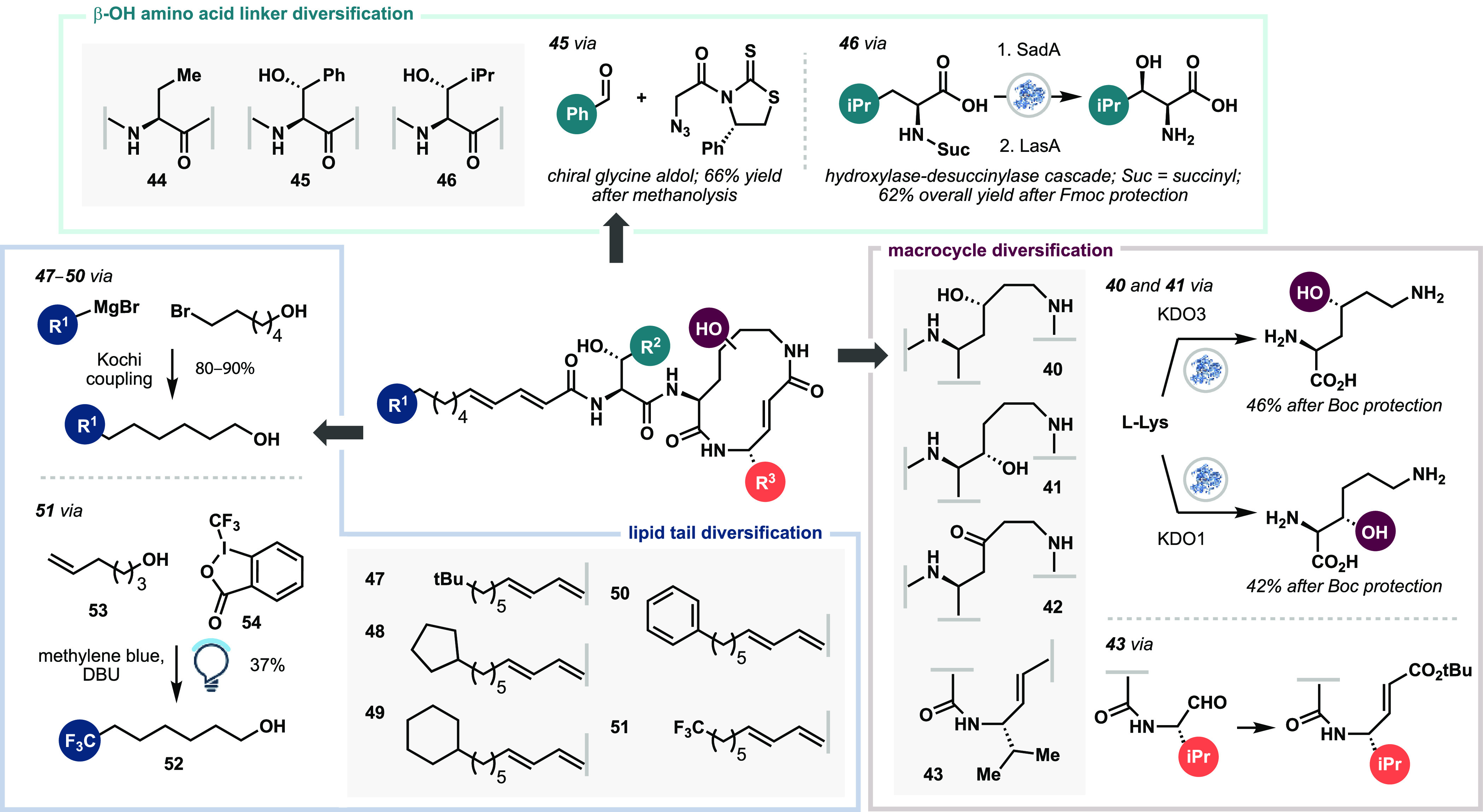
Synthesis of Cepafungin Analogues **40**–**51**

In our initial attempts to hydroxylate lysine with KDO3, full consumption of starting material was observed with 2 equiv of αKG cosubstrate, but no desired hydroxylysine product could be isolated by ion-exchange resin or by preparative TLC after Fmoc derivatization. NMR and LCMS analysis indicated primarily decomposition of the starting material. With 1.25 equiv of αKG cosubstrate, LCMS analysis indicated cleaner full conversion to the desired hydroxylated product. Upon scale-up, performing the reaction with 20 mM Lys at prelysis OD_600_ = 10 revealed the overoxidized keto lysine **S13** as the major product (32%), and (2*S*,4*R*)-4-hydroxylysine **S11** (11%) and lactone **S12** (6%) as minor products. To our knowledge, this reactivity pattern for a lysine hydroxylase was not described before in the initial disclosure of KDO3. To favor mono-oxidation, the reaction was performed with 40 mM lysine substrate, 1.25 equiv of αKG, and prelysis OD_600_ = 4.3 (see the Supporting Information for optimization, Table S4). On gram scale, these conditions provided a combined 42% 2-step yield of (2*S*,4*R*)-4-hydroxylysine **S11** and its spontaneously lactonized counterpart **S12**, and 12% of keto-lysine byproduct **S13** after Boc protection (Scheme S3). Lactone **S12** was carried forward as in Scheme 2B to access the *epi*-4-OH cepafungin analogue **40** (Scheme 3).

While the keto-lysine product **S13** was unexpected, it raised a question of whether a cepafungin macrocycle harboring a C4-ketone in place of a hydroxyl would be beneficial. We envisioned that the ketone sp^2^ center could introduce additional strain to the 12-membered ring, potentially making the α,β-unsaturated amide more reactive. Additionally, this substitution would swap a hydrogen bond donor for an acceptor. The protected keto-lysine byproduct could be coupled directly to an Ala derivative toward the keto-macrocycle analogue, though we opted instead to directly oxidize intermediate **S9** from the natural product synthesis. Subsequent deprotection and macrocyclization proceeded in similar yields as in cepafungin I, despite the additional sp^2^ center. KDO1 was used for selective C3 oxidation of lysine as described in the literature.^50^ After peptide coupling to **31**, subsequent steps followed those of the natural product synthesis to provide **41** in good yield. The valinyl cepafungin analogue **43** was prepared by aminolysis of lactone **30** with a dimethylaluminum amide reagent generated from Val derivative **S14b**. Gratifyingly, the aminolysis conditions developed in the total synthesis tolerated the bulkier Val side chain, giving 85% yield of dipeptide **S15c** on gram scale.

For diversification of the β-OH amino acid linker, the desoxy-Thr variant **44** was synthesized from commercial (*S*)-ethylglycine. Toward β-OH-Phe **45**, Franck’s auxiliary-based aldol chemistry^51^ readily allowed the union of a chiral glycine equivalent to benzaldehyde. The addition product was directly subjected to methanolysis in one pot to cleave the auxiliary and form methyl ester **S34** in 66% yield over 2 steps. Catalytic hydrogenation and subsequent couplings to the unsaturated fatty acid and core macrocycle completed the synthesis of β-OH-Phe cepafungin **45**. Next, we targeted β-OH-Leu to compare the natural product against both larger aromatic and aliphatic β-OH amino acids at the S3 subsite. Unfortunately, the Franck aldol reaction did not produce any of the desired adduct with either isobutyraldehyde or methacrolein. Instead, we turned to the Fe/αKG dioxygenase SadA, which performs stereoselective β-hydroxylation on a variety of aliphatic N-succinyl amino acids, as well as LasA, an N-desuccinylase enzyme from the same producing organism for a cascade synthesis of β-hydroxy-Leu.^52^ While the original report for the enzymes describes a one-pot transformation with both enzymes, we opted for flash C18 purification of intermediate **S36** to facilitate optimization of the LasA reaction that had initially failed to produce the desired product or with only minimal conversion. Ultimately, 10 mM loading of N-succinyl-β-hydroxy-Leu substrate and 20 °C reaction with LasA at prelysis OD_600_ = 20 promoted nearly complete conversion to free β-hydroxy-Leu, isolated in 81% yield as the Fmoc derivative over two steps. Fatty acid analogues **47**–**50** were prepared by Kochi couplings of 6-bromo-1-hexanol with the corresponding Grignard reagents, then carried forward following the cepafungin I route. Finally, the CF_3_ fatty acid derivative **51** was prepared from trifluoromethylhexanol (**52**), made via photocatalytic trifluoromethylation of 5-hexenol (**53**) with Togni reagent II (**54**).^49^ In total, 12 analogues were synthesized in this initial campaign.

**Figure 4:**
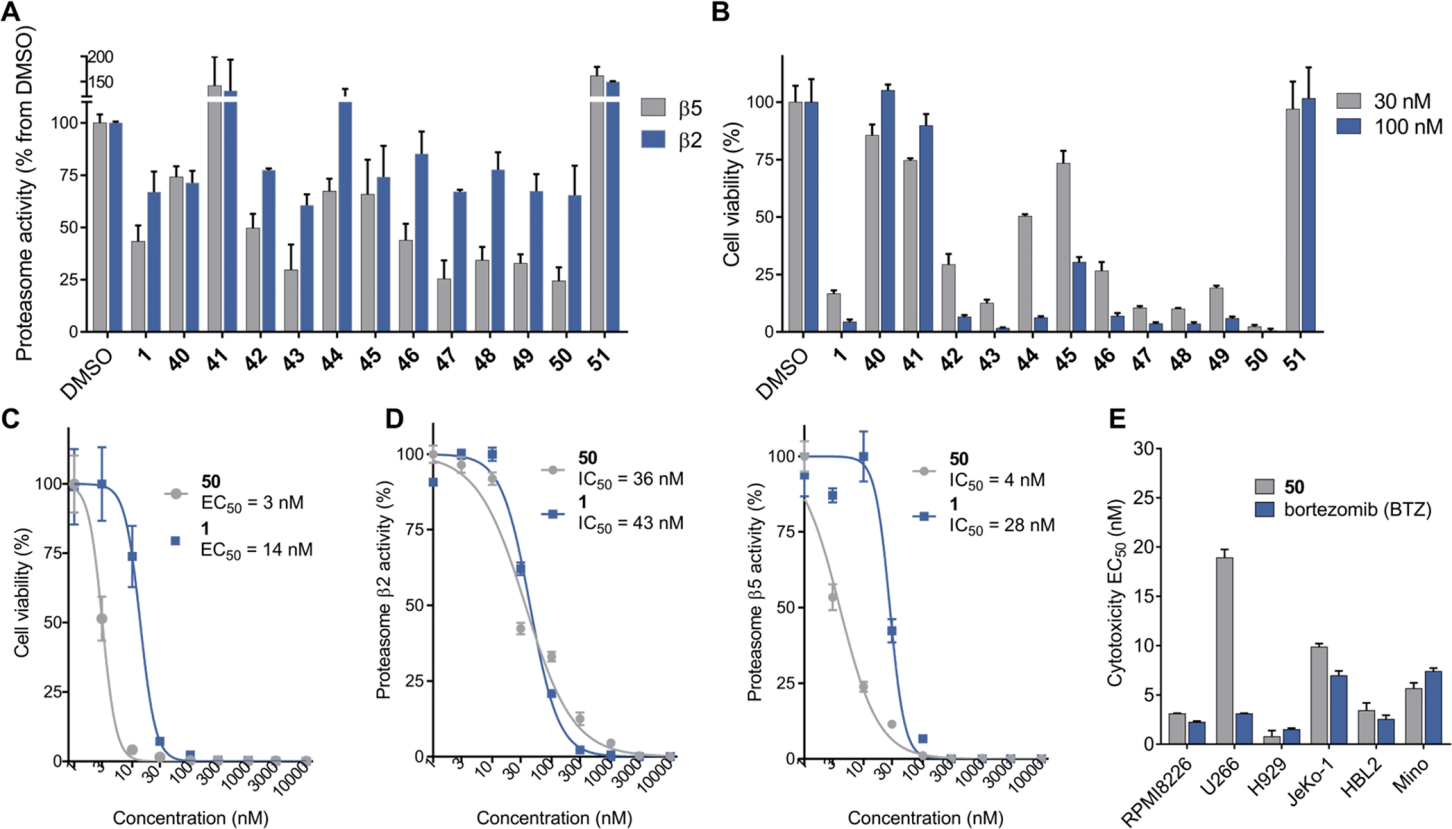
Biological evaluation of cepafungin (**1**) and analogues **40**–**51** in RPMI 8226 cells. (A) Proteasome inhibition screening at 30 nM compound concentration with subunit-specific fluorogenic proteasome substrates Suc-LLVY-AMC (β5) and Ac-RLR-AMC (β2) (*n* = 3). (B) Cytotoxicity screening at 30 and 100 nM compound concentration (*n* = 3). (C) EC_50_ measurement of **50** in comparison to **1** (*n* = 3). (D) Proteasome subunit IC_50_ measurements of **50** in comparison to **1** (*n* = 3). (E) Cytotoxicity comparison of **50** and bortezomib in human MM (RPMI8226, U266, H929) and MCL (JeKo-1, HBL2, Mino) cell lines (*n* = 3). All error bars represent standard deviation.

Cellular assays were performed on human multiple myeloma RPMI 8226 cells (Figure 4). First, relative inhibition of proteasome subunits β5 and β2 for all analogues and the natural product was measured at 30 nM compound concentration. Following incubation of the compounds in cell culture for 6 h, cells were lysed, and fluorogenic subunit-specific peptide substrates Suc-LLVY-AMC (β5) or Ac-RLR-AMC (β2) were incubated with the lysates; fluorescence readings provided relative proteasome activity levels in comparison with DMSO (Figure 4A). Next, cytotoxicity screening was performed at 30 and 100 nM concentrations for sufficient coverage of dynamic range (Figure 4B). From these results, cytotoxicities track closely with relative proteasome inhibition, suggesting that proteasome inhibition is a primary cause of cytotoxicity. Notably, compounds **43**, **47**, **48**, and **50** are more potent than the parent natural product **1**, with phenyl cepafungin **50** being the most potent. EC_50_ measurement in RPMI 8226 revealed that **50** has ca. 4–5-fold greater cytotoxicity than the parent cepafungin I **1** with an EC_50_ of 3 nM (Figure 4C). Proteasome subunit activity measurements revealed that **50** has 7-fold lower IC_50_ than **1** for the β5 subunit and roughly similar IC_50_ for the β2 subunit (Figure 4D). Therefore, the improved cytotoxicity of phenyl cepafungin **50** likely results from a more pronounced loss of β5 function in RPMI 8226 cells. This drastic increase in proteasome inhibition may be due to π-stacking of the lipid tail phenyl ring with the β6 subunit residues Tyr106 and/or Tyr5. The β2 subunit-bound natural product has residues Tyr102 and Phe103 on the adjacent β3 subunit oriented further away from the terminal lipid methyl groups, and presumably would not facilitate π-stacking to the extent possible at the adjacent β6 subunit (see Figure 3 and Figure S4). The *tert*-butyl and cyclopentyl cepafungins **47** and **48**, respectively, also have improved cytotoxicity relative to **1** albeit to a lesser extent than **50**, suggesting the aromatic moiety in the lipid tail is favored over large aliphatic groups. Cyclohexyl cepafungin **49** appears to have roughly similar activity as the natural product. Interestingly, valinyl cepafungin **43** displayed slightly greater cytotoxicity than the natural product and prompted the synthesis of a hybrid analogue (**S39**) containing the phenyl cepafungin tail fragment and valinyl cepafungin macrocycle. These combined modifications were made in hopes that the effects of both substitutions would be additive. While this hybrid analogue was significantly more potent than the natural product with an EC_50_ of 4.5 nM against RPMI 8226, its cytotoxicity did not surpass that of **50** (Figure S3). Of the other modified macrocycle analogues, only keto cepafungin **42** displayed potency approaching that of the natural product, while epi-4-OH (**40**) and 3-OH cepafungin (**41**) were nearly inactive at both concentrations. This result indicates that the regio- and stereochemistry of lysine hydroxylation in the cepafungin and glidobactin biosyntheses is critical for potent proteasome inhibition, though whether the alternative oxidation patterns might have led to undesired electrostatic interaction or suboptimal macrocyclic conformation for binding is beyond the scope of the current study. The presence of a Thr residue next to the cepafungin macrocycle appears to be favored over the ethyl side chain of desoxy-Thr analogue **44** as indicated by cytotoxicity and hydrogen-bonding seen in the cepafungin-bound crystal structure (Figures 3 and 4B). However, the β-OH-Phe analogue **45** was substantially weaker, while the β-OH-Leu analogue **46** approached the potency of the natural product. The trifluoromethyl analogue **51** was essentially inactive at both concentrations tested.

With a new lead analogue (**50**) in hand, we sought to compare it against the clinical drug bortezomib. It has been shown that, in addition to MM, mantle cell lymphoma (MCL) is also sensitive to proteasome inhibition. Indeed, bortezomib itself is FDA-approved for the treatment of MCL.^53^ Therefore, we compared **50** and bortezomib in 3 different cell lines of both MM and MCL (Figure 4E and Figure S2). Gratifyingly, phenyl cepafungin displayed similarly low-nanomolar cytotoxicity in all cell lines as bortezomib except for U266, for which it has an EC_50_ of ∼20 nM.

**Figure 5:**
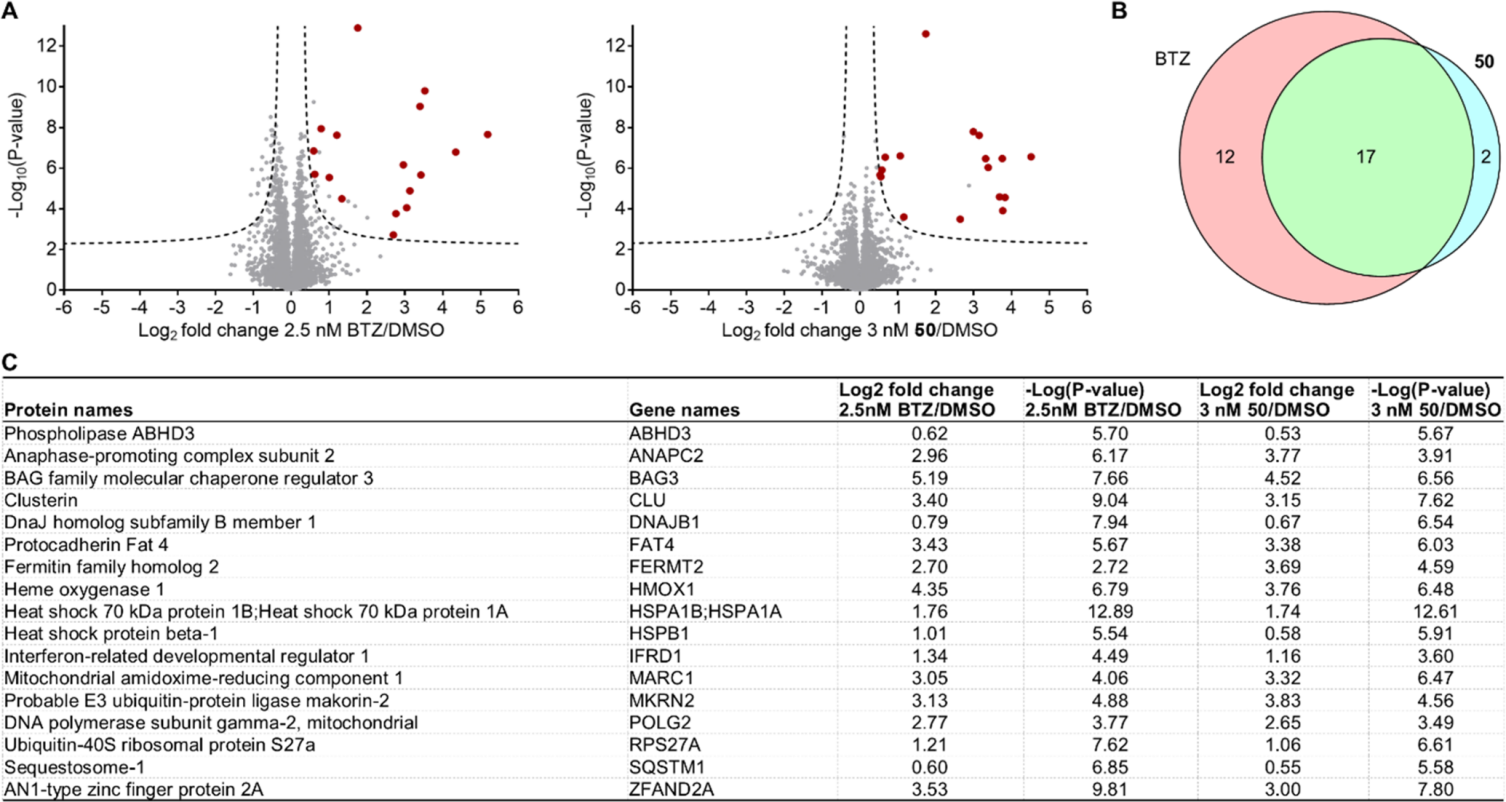
Comparative global proteomics study of the mode of action of BTZ and **50**. (A) Volcano plots representing the global proteome profile of RPMI 8226 cells treated with BTZ or **50** versus DMSO for 14 h. Data are represented as log2 fold change; dotted lines represent a false discovery rate of 5% and an *S*_0_ of 0.1. Quantification was performed using the LFQ method (*n* = 6). Red dots indicate proteins with a significant increase in expression levels in response to treatment with both BTZ and **50**. (B) Venn diagram representing the overlap in significantly upregulated proteins from panel A. (C) Table of common upregulated proteins between BTZ and **50** compared to DMSO. Shown are log2 fold change ratios and −log(*P*-values).

Finally, we evaluated the changes in global protein expression following treatment of RPMI 8226 cells with either BTZ or **50**. Cells were treated for 14 h with compounds at their 24 h EC_50_ concentration (2.5 nM BTZ and 3 nM **50**) to minimize induction of apoptosis and protein abundance changes caused by toxicity. Following treatment, cells were collected via centrifugation, and protein lysates were digested with trypsin and analyzed using LC-MS/MS. Out of the 4080 quantified proteins, 29 showed a statistically significant (false discovery rate of 5% and an *S*_0_ of 0.1) increase in expression after BTZ treatment and 19 proteins following treatment with **50** compared to the DMSO control (Figure 5 and Table S5). Importantly, 17 proteins were commonly affected by both compounds (59% overlap for BTZ and 89% for **50**, Figure 5C). Indeed, the expression of 8 out of these 17 proteins is known to be elevated following BTZ treatment: BAG3, DNAJB1, HMOX1, HSPA1A and HSPA1B, HSPB1, RPS27A, SQSTM1, and ZFAND2A.^54−60^ Two more proteins (CLU and FERMT2) were described to be upregulated following treatment with other proteasomal inhibitors.^61,62^ Altogether, this result demonstrates that treatment with BTZ and **50** leads to largely overlapping changes in protein expression thus confirming that both compounds share an identical mode of action.

## Conclusion

The chemoenzymatic strategies described herein enabled the first synthesis of cepafungin I. Early route exploration examined the use of the fungal Fe/αKG FoPip4H for site-selective C–H hydroxylation of l-pipecolic acid, which was followed by functional group interconversions to generate a 4-hydroxylysine surrogate. Unfortunately, all attempts at macrocyclization failed to provide the desired 12-membered macrolactam and instead resulted in the formation of a 6-membered cyclization product in low yield. This result underscored the challenge of forming this 12-membered ring and prompted a more biomimetic protecting group-free macrolactamization strategy, which led to our study on the glidobactin biosynthetic gene cluster to discover and characterize the native lysine hydroxylase. The Fe/αKG from the cluster, GlbB, hydroxylates l-lysine with exquisite regio-/stereoselectivity and very high total turnover, allowing for easy scale-up whereby ∼7 g of free l-lysine could be hydroxylated with 1 L of clarified lysate. By leveraging the high efficiency of this biocatalytic reaction, we completed the first synthesis of cepafungin I in 7.9% yield over 9 longest linear steps.

Prior to our work, a major knowledge gap in the bioactivity of cepafungin was its engagement of the proteasome in human MM cells and selectivity in targeting the proteasome over a multitude of other proteases. To address this, an alkyne-tagged probe analogue was synthesized by our chemoenzymatic route and used for classical chemoproteomics studies. A combination of in-gel competitive profiling and in situ competitive LC-MS/MS-based chemoproteomics demonstrated cepafungin’s exceptional selectivity toward several proteasome subunits. Moreover, global proteomics experiments with **1** and bortezomib indicated a high degree of overlap in upregulated protein expression, suggesting a similar mechanism of action between the two molecules, which was also confirmed in this study with compound **50**.

The modularity of our chemoenzymatic route enables facile modification of all regions of the natural product scaffold to gain comprehensive SAR data, and its efficiency allows for routine scale-up to provide ≥40 mg of final compounds. By taking advantage of these features, modifications to the natural product were made at three distinct regions of the scaffold, guided by proteasome-bound crystal structure and preliminary SAR data. The regio- and stereochemistry of lysine hydroxylation is critical for potent proteasome inhibition, whereby the configuration found in the parent natural product was found to be optimal for proteasome inhibition, despite a lack of obvious binding interactions in the crystal structure of yeast 20S proteasome:cepafungin complex. The S3 subsite appears to prefer aliphatic β-OH amino acids linked to the macrocycle, and a larger aliphatic residue proximal to the macrocycle’s reactive electrophile is tolerated. The diene moiety in the tail fragment is also essential for potent inhibition, as is the terminal functionality of the fatty acid. This work constitutes the most comprehensive SAR study on the cepafungin series to date, undoubtedly made possible by the high modularity of our chemoenzymatic route. One fatty acid analogue, phenyl cepafungin (**50**), has 7-fold greater β5 inhibitory activity and exhibits similar cytotoxicity to the clinically approved drug bortezomib in several MM and MCL cell lines. Potent β5 and β2 coinhibitory activity, as seen in **50** and other analogues, may serve as a viable means of overcoming bortezomib and carfilzomib resistance in multiple myeloma. In vivo mouse studies, evaluation against PI-resistant cancers, and further structural refinements on **50** are ongoing in our laboratories.

## Supporting Information Available

Experimental details, analytical data, and ^1^H and ^13^C NMR data (PDF)
Proteomics data set (Table S3) (XLSX)
Proteomics data set (Table S5) (XLSX)
Transparent Peer Review report available (PDF)
