# Supplementary material for: Comprehensive Structure–Activity Relationship Studies of Cepafungin Enabled by Biocatalytic C–H Oxidations
Source: ACS Cent Sci. 2023 Jan 27;9(2):239–51. doi: 10.1021/acscentsci.2c01219 (PMC9951290; doi:10.1021/acscentsci.2c01219)
Supplement: Supplementary file 1 — oc2c01219_si_001.pdf [file oc2c01219_si_001.pdf]

Comprehensive Structure-Activity Relationship Studies of Cepafungin Enabled by Biocatalytic C–H Oxidation

Alexander Amatuni,[1] Anton Shuster,[1] Daniel Abegg,[2] Alexander Adibekian,[2,*] Hans Renata[3,*]
[1]Skaggs Doctoral Program in the Chemical and Biological Sciences, Scripps Research, La Jolla, CA 92037, USA
[2]Department of Chemistry, University of Illinois at Chicago, Chicago, IL 60607, USA
[3]Department of Chemistry, BioScience Research Collaborative, Rice University, Houston, TX 77005, USA

SUPPLEMENTARY MATERIAL

Table of Contents

General materials and methods……………………………………………..page S2
Protein and DNA sequences…………………………………………………...page S3
Mammalian cell culture and lysate preparation……………………………..page S4
In situ β2 and β5 proteasome activity assays………………………………..page S4
Cytotoxicity measurements……………………………………………………page S5
Figure S1. Identification of the Cepafungin I (**1**) cellular targets…………..page S5
Global proteomics analysis via LC-MS/MS…………………………………...page S6
Figure S2. Cytotoxicity of **50** and BTZ in MM and MCL cell lines………..page S7
Figure S3. Cytotoxicity of **50** and **S39** in RPMI 8226 cells………………...page S8
Figure S4. Crystal structure of **1** at β2 subunit of yeast 20S proteasome…...page S8
Synthetic procedures and characterization data……………………………..page S9
Table S1. Condition screening for Mitsunobu macrocyclization of **25**……..page S19
Scheme S1. Attempted differential lysine amine protections………………page S19
Scheme S2. **S7** primary alcohol selective oxidation route………………….page S20
Table S2. Screening conditions for selective oxidation of **S7**……………...page S20
Initial proteomics results……………………………………………………page S21
Preliminary analog series……………………………………………………page S21
KDO3 reaction optimization…………………………………………………...page S21
Scheme S3. Hydroxylation of L-lysine with KDO3…………………………...page S21
Table S4. KDO3 reaction optimization………………………………………...page S23
Expanded analog series - general procedures A-L…………………………..page S25
Analog synthesis procedure…………………………………………………...page S32
References……………………………………………………………………page S77
NMR spectral data……………………………………………………………page S81

General Materials and Methods

Unless otherwise noted, all chemicals and reagents for chemical reactions were purchased at the highest commercial quality and used without further purification. Reactions were monitored by thin layer chromatography (TLC). TLC was performed with 0.25 mm E. Merck silica plates (60F-254) using short-wave UV light as the visualizing agent and KMnO4, ninhydrin, vanillin, bromocresol green, iodine and heat as developing agents. LC/HRMS was performed on a Thermo Vanquish UHPLC coupled to an Orbitrap Exploris 120 (HRESI) equipped with an Accucore C18 column (100 mm × 2.1 mm), or on an Agilent 1260 Infinity system equipped with Poroshell 120 EC-C18 column (4.6 x 50 mm, 2.7 µm) and an Agilent G6230B TOF LC/MS with water and acetonitrile buffered with 0.1% formic acid as mobile phases. LCMS analysis was performed on an Agilent 1260 Infinity System coupled to an Agilent 6100 Series Single Quadrupole LCMS equipped with Poroshell 120 EC-C18 column (3.0 x 50 mm, 2.7 µm). Preparative HPLC purification was performed on an Agilent 1260 Infinity system equipped with a Zorbax Eclipse XDB-C18 PrepHT column (21.2 x 250 mm, 7 µm) with water and acetonitrile buffered with 0.1% formic acid as mobile phases. NMR spectra were recorded on a Bruker AVANCE AV400 (400 MHz and 101 MHz) or Bruker AVANCE AV600 (600 MHz and 151 MHz) at 23 °C unless otherwise noted. Optical rotations were measured on Autopol IV polarimeter (Rudolph Research Analytical). Sonication was performed using a Qsonica Q500 sonicator. Biochemicals and media components were purchased from standard commercial sources. Expression vectors were obtained via DNA synthesis from Twist Bioscience and were used directly with electrocompetent *E. coli* BL21(DE3). Electrocompetent *E. coli* BL21(DE3) strains were purchased from Lucigen. All *E. coli* strains generated in this work are stored as glycerol stocks at –80 °C.

Protein and DNA Sequences

Protein sequence of FoPip4H (Uniprot accession code: A0A125SY85)

MAALNADTLDMSLFFGTPSQKQDFCDSLLRLLKKRGGVKLINHPIPSTSIHELFAQTKRFFNLP
LETKMLAKHPPQANPNRGYSFVGQENVANISGYEKGLGPLKTRDIKETVDFGSANDELVDNLWV
PEEELPGFRSFMEGFYELAFKTEMQLLEALAIALGVSPDHLKSLHNRAENEFRILHYPAIPASE
LADGTATRIAEHTDFGTITMLFQDSVGGLQVEDQENLGTFNNVESASPTDIILNIGDSLQRLTN
DTFKAACHRVTYPPSIKAGDGEQVIPERYSIAYFAKPNRSASLFPLKEFIEEGVPCKYEDVTAW
EWNNRRIEKLFSAEAKA

DNA sequence of FoPip4H (codon optimized by Genscript)

ATGGCGGCGCTGAACGCGGACACCCTGGATATGAGCCTGTTCTTTGGTACCCCGAGCCAGAAGC
AAGACTTCTGCGATAGCCTGCTGCGTCTGCTGAAGAAACGTGGTGGCGTGAAACTGATCAACCA
CCCGATTCCGAGCACCAGCATCCACGAACTGTTTGCGCAGACCAAGCGTTTCTTTAACCTGCCG
CTGGAGACCAAGATGCTGGCGAAGCACCCGCCGCAGGCGAACCCGAACCGTGGTTACAGCTTCG
TGGGCCAAGAAAACGTTGCGAACATCAGCGGTTATGAGAAAGGTCTGGGCCCGCTGAAGACCCG
TGACATTAAAGAAACCGTGGATTTTGGCAGCGCGAACGACGAGCTGGTTGACAACCTGTGGGTT
CCGGAGGAAGAGCTGCCGGGTTTCCGTAGCTTTATGGAAGGCTTCTACGAGCTGGCGTTTAAGA
CCGAAATGCAACTGCTGGAAGCGCTGGCGATTGCGCTGGGTGTTAGCCCGGACCACCTGAAAAG
CCTGCACAACCGTGCGGAAAACGAGTTCCGTATCCTGCACTATCCGGCGATTCCGGCGAGCGAA
CTGGCGGATGGTACCGCGACCCGTATTGCGGAGCACACCGATTTCGGCACCATTACCATGCTGT
TTCAAGACAGCGTGGGTGGCCTGCAGGTTGAAGATCAAGAGAACCTGGGTACCTTCAACAACGT
TGAAAGCGCGAGCCCGACCGACATCATTCTGAACATCGGCGATAGCCTGCAGCGTCTGACCAAC
GACACCTTTAAAGCGGCGTGCCACCGTGTGACCTACCCGCCGAGCATTAAAGCGGGTGATGGCG
AACAAGTTATCCCGGAGCGTTACAGCATTGCGTATTTCGCGAAGCCGAACCGTAGCGCGAGCCT
GTTCCCGCTGAAGGAGTTTATCGAAGAGGGCGTGCCGTGCAAATATGAAGATGTTACCGCGTGG
GAGTGGAACAACCGTCGTATTGAAAAGCTGTTTAGCGCGGAGGCGAAAGCGTGA

Protein sequence of KDO3 (UniProt accession code: A5FF23)

MKSQSLIEDEIPVKENYAYQIPTSPLIVEVTPQERNILSNVGALLEKAFKSYENPDYIEALHLY
SFQLLPERIARILSRFGTDFSADQYGAIIFRGLLEVDQDHLGPTPANWQSADYSKLNKYGFICS
LLHGAVPSKPVQYYAQRKGGGILHAVIPDEKMAATQTGSGSKTNLYVHTEDAFLLHQADFLSFL
YLRNEERVPSTLYSVRSHGKVNKIMEKLFDPIYQCPKDANYQEEINDGPLASVLYGNKKLPFIR
FDAAEQIFNENAGQTPEALYNLTEFWNEAKELINSDYIPDSGDVIFVNNHLCAHGRSAFTAGQK
EENGKLVPCERRQMLRMMSKTSLIHIRSMTHTDDPYFVMEEHLGKVFDQA

DNA sequence of KDO3 (codon optimized by Genscript)

AAGTCTCAGAGCTTAATTGAAGACGAAATCCCGGTGAAGGAGAATTATGCATACCAGATTCCTA
CGTCTCCTCTCATTGTCGAAGTGACGCCACAAGAACGCAATATCCTGTCAAATGTAGGTGCGCT
CTTGGAGAAGGCGTTTAAATCGTACGAGAATCCTGATTATATCGAGGCCCTTCACCTCTACTCA
TTCCAATTGTTGCCGGAGCGCATCGCGCGTATTCTCTCTCGTTTCGGCACGGATTTCTCCGCTG

ATCAGTACGGCGCAATCATTTTCCGCGGCCTGCTTGAGGTCGATCAGGACCACTTAGGCCCGAC
CCCCGCGAACTGGCAAAGCGCTGACTACAGCAAACTGAATAAGTATGGTTTCATCTGTAGCCTC
TTACACGGTGCCGTGCCAAGTAAACCCGTGCAATACTACGCACAACGCAAGGGCGGCGGTATCC
TGCATGCGGTGATTCCGGACGAGAAGATGGCAGCTACCCAAACTGGGTCCGGAAGTAAAACTAA
TCTGTACGTTCATACGGAAGATGCCTTCCTGCTGCATCAAGCCGACTTCTTAAGCTTTCTTTAT
CTGCGCAACGAAGAACGCGTGCCTTCAACATTGTATTCGGTTCGCTCTCACGGTAAGGTAAACA
AGATTATGGAGAAGTTGTTCGACCCGATTTATCAATGTCCTAAGGATGCGAACTACCAAGAGGA
AATCAACGACGGCCCGCTGGCCTCAGTTCTGTATGGTAATAAGAAGTTGCCCTTCATTCGCTTC
GATGCCGCGGAACAGATCTTTAACGAGAACGCAGGTCAGACCCCGGAAGCCTTGTACAACTTAA
CAGAGTTCTGGAATGAGGCGAAAGAGTTGATCAACAGTGACTATATCCCTGACAGTGGGGACGT
CATTTTCGTTAATAACCACCTCTGCGCACATGGCCGCTCGGCTTTCACCGCTGGTCAGAAAGAG
GAAAATGGTAAGCTGGTCCCATGTGAGCGCCGTCAAATGTTACGTATGATGAGCAAGACGAGTT
TGATCCATATTCGCAGTATGACTCACACTGATGACCCTTATTTTGTTATGGAAGAGCACTTGGG
AAAGGTCTTTGATCAAGCCTAA

Expression vectors for KDO1[1] and SadA/LasA[2] were constructed as described in previously reported procedures. For the construction of expression vectors, each of the above sequences was inserted between the NdeI and BamHI restriction sites within the commercial pET28a(+) vector. The resulting expression vector was used directly to transform electrocompetent *E. coli* strain BL21(DE3). Variants were stored as glycerol stocks at –80 °C.

Mammalian cell culture and lysate preparation

Mammalian cells were maintained in RPMI-1640 media. All media were supplemented with 10% fetal bovine serum (FBS, Life Technologies Cat# 10437028), non-essential amino acids (Sigma-Aldrich Cat# M7145) and penicillin/streptomycin (Sigma-Aldrich Cat# P4333). Cells were grown at 37 °C under 5% CO_2 atmosphere. Cells were harvested by pipetting, then washed 2 times in PBS by brief centrifugation at 150 x g at 4 °C and resuspended in PBS. Cells were lysed by needle sonication to obtain cell lysates and protein concentration was determined using the Bradford assay.

In situ β2 and β5 proteasome activity assays

RPMI 8226 cells were seeded in a 6-well plate (1,000,000 cells/well) in cell culture media supplemented with **indicated compounds** at given concentrations (1,000x stock in DMSO) or DMSO and cultured for 6 hours. Cells were then collected and lysed. Lysates (1 mg/mL, 25 µL) were dispensed into 100 µL assay buffer containing 100 µM proteasome substrate **Suc-LLVY-**

AMC (β5) or **Ac-RLR-AMC** (β2). The plates were incubated at 37 °C for one hour and fluorescence was read at A360ex/A460em. (n = 3)

Cytotoxicity Measurements

Cells of the indicated cell line origin were seeded in a 96-well plate (20,000 cells/well) in cell culture media supplemented with **indicated compounds** at given concentrations (1,000x stock in DMSO) or DMSO and cultured for 48 hours. Toxicity was determined using the WST-1 assay (Roche) according to the protocol of the manufacturer. (n = 3)

A

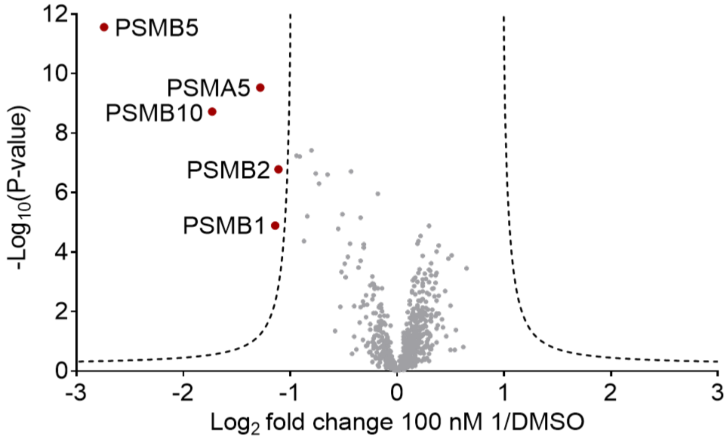

B

Protein names	Gene names	Log2 fold change 100nM 1/DMSO	-Log(P-value) 100nM 1/DMSO
Proteasome subunit β5	PSMB5	-2.74	11.56
Proteasome subunit β2i	PSMB10	-1.73	8.72
Proteasome subunit α5	PSMA5	-1.28	9.53
Proteasome subunit β1	PSMB1	-1.14	4.89
Proteasome subunit β2	PSMB2	-1.11	6.78

Figure S1. Identification of the Cepafungin I (**1**) cellular targets in RPMI 8226 cells. **A.** Volcano plot analysis of the *in-situ* competitive LC-MS/MS pulldown experiment with 100 nM **1** or DMSO and with 10 µM **S10** (n = 6). Quantification was performed using the LFQ method and data are represented as log2 fold change; dotted lines represent a false discovery rate of 1% and an S_0 of 2. Red dots indicate significantly competed proteins (>50% competition) by **1**. **B.** Table with enriched and competed cellular targets of **1**.

Global proteomics analysis via LC-MS/MS.

RPMI 8226 cells were seeded in a 6-well plate (1,000,000 cells/well) in cell culture media supplemented with DMSO, BTZ (2.5 nM, 1000x stock in DMSO), or **50** (3 nM, 1000x stock in DMSO) for 14 h. Cells were centrifuged (3,000 x g 5 min 4°C) and washed twice with PBS. Cell pellets were resuspended in PBS and protein concentration was determined using the Bradford assay (Bio-Rad). Urea (9 M in 100 mM NH_4HCO_3 pH 8) was added to a final concentration of 6 M to 30 µg lysate (15 µL PBS). Proteins were reduced with 10 mM tris(2-carboxyethyl)phosphine hydrochloride (TCEP, 20x fresh stock in water) for 30 min at r.t., and alkylated with 25 mM iodoacetamide (250 mM fresh stock in water) for 30 min at r.t. in the dark. The sample was then diluted to 2 M urea with 50 mM NH_4HCO_3 pH 8 and digested with trypsin (Thermo scientific, 1.5 µL of 0.5 µg/µL) overnight at 37°C in the presence of 1 mM $CaCl_2$. Peptides were desalted over a self-packed C18 spin column and dried. Peptides were resuspended in water with 0.1 % FA and analyzed using EASY-nLC 1200 nano-UHPLC coupled to Q Exactive HF-X Quadrupole-Orbitrap mass spectrometer (Thermo Scientific). The chromatography column consisted of a 50 cm long, 75 µm i.d. microcapillary capped by a 5 µm tip and packed with ReproSil-Pur 120 C18-AQ 2.4 µm beads (Dr. Maisch GmbH). LC solvents were 0.1 % FA in H2O (Buffer A) and 0.1 % FA in 90 % MeCN: 10 % H_2O (Buffer B). Peptides were eluted into the mass spectrometer at a flow rate of 300 nL/min. over a 90-min linear gradient (5-35 % Buffer B) at 65 °C. Data was acquired in data-dependent mode (top-20, NCE 28, R = 15,000) after full MS scan (R = 60,000, m/z 400 – 1,300). Dynamic exclusion was set to 10 s, peptide match to prefer and isotope exclusion was enabled. The MS data were analyzed with MaxQuant (V2.0.3.0) and searched against the human proteome (Uniprot) and a common list of contaminants (included in MaxQuant). The first peptide search tolerance was set at 20 ppm, 10 ppm was used for the main peptide search and fragment mass tolerance was set to 0.02 Da. The false discovery rate for peptides, proteins, and site identification was set to 1%. The minimum peptide length was set to 6 amino acids and peptide re-quantification and “match between runs” was enabled. Methionine oxidation and N-terminal acetylation were searched as variable modifications and carbamidomethylation of cysteine as fixed modification.

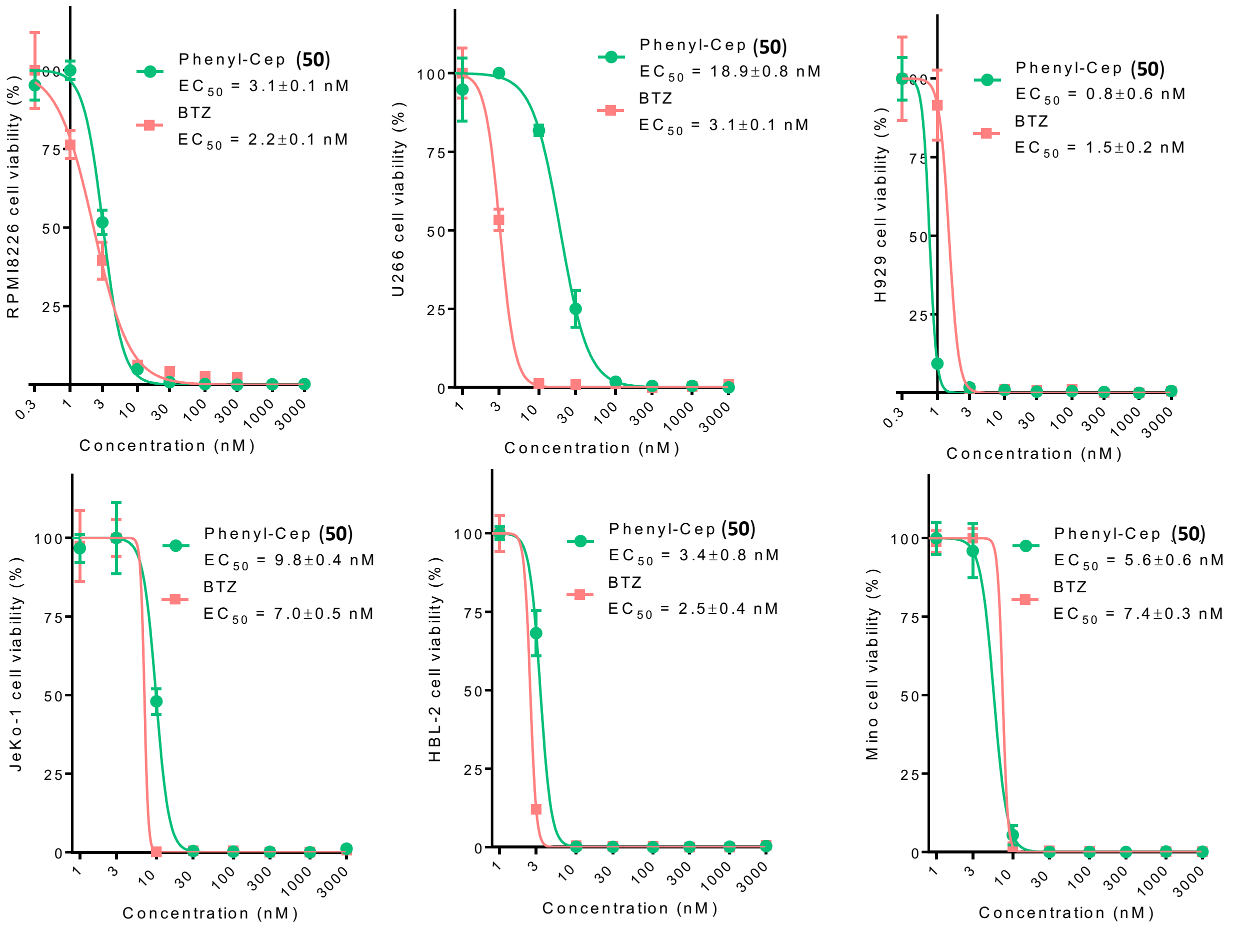

Figure S2. Cytotoxicity EC_{50} measurements for **50** and bortezomib (BTZ) in MM and MCL cell lines. Data shown are average values ± standard deviations (n = 3).

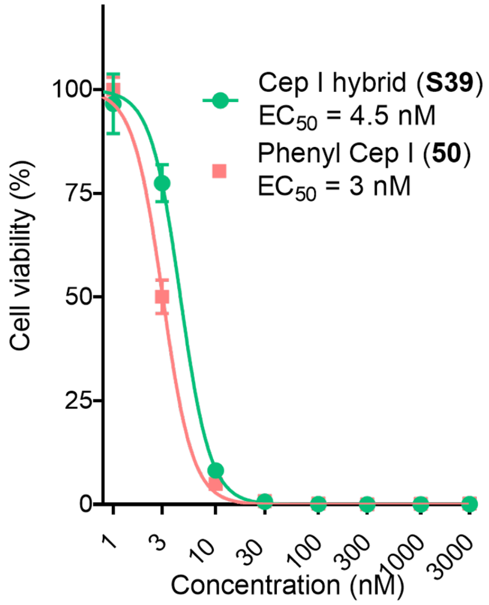

Figure S3. Cytotoxicity of phenyl cepafungin (**50**) and hybrid analog (**S39**) in RPMI 8226 cells. Data shown are average values ± standard deviations (n = 3).

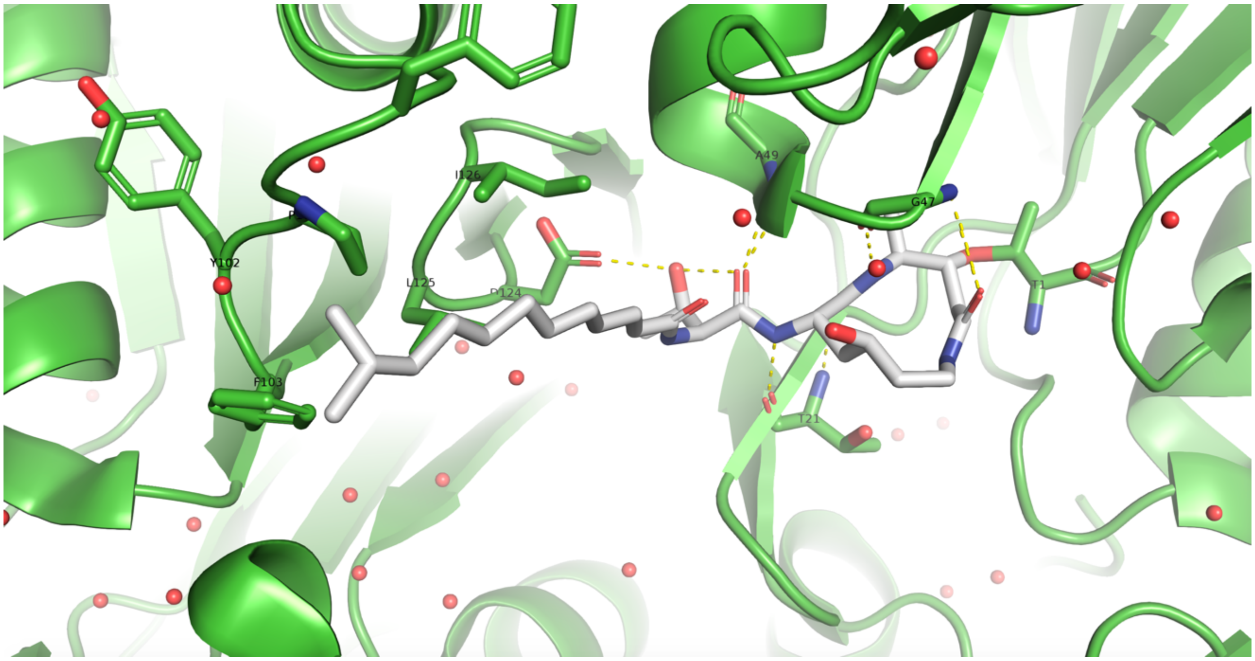

Figure S4. Bound crystal structure of cepafungin I at β2 subunit of yeast 20S proteasome. PDB ID: 4FZC.[3] Dashed lines indicate polar contacts. The catalytic β2 N-terminal threonine covalently bonded to the cepafungin macrocycle is labeled "T1". β2 residues involved in polar contacts are Gly47, Ala49, Thr21 and Asp124. A hydrophobic channel for the tail fragment involves β3 subunit residues Leu125, Ile126, Pro101 and Phe103.

Synthetic Procedures

1st-generation route to cepafungin using FoPip4H

(2*S*,4*S*)-1-(*tert*-butoxycarbonyl)-4-hydroxypiperidine-2-carboxylic acid (20)

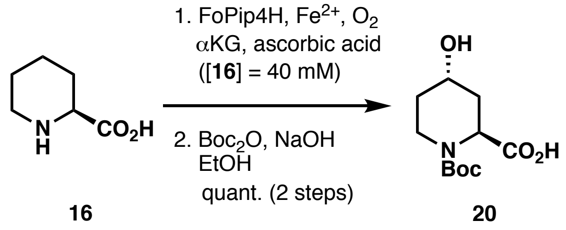

A glycerol stock of *E. coli* BL21(DE3) cells harboring pET-28a(+)-FoPip4H plasmid was used to inoculate an overnight culture of LB media (4 mL) containing 50 μg/mL kanamycin. This culture was used to inoculate 500 mL TB media (in 1 x 2 L non-beveled Erlenmeyer flask) containing 50 μg/mL kanamycin. The culture was shaken at 250 rpm at 37 °C for 2.5 h or until an OD_{600} = 0.7 was reached. The culture was cooled on ice (15 min), induced by adding IPTG to final concentration of 25 μM, then allowed to continue shaking at 250 rpm/20 °C for another 20 h. Cells were harvested by centrifugation (4 °C, 15 min, 4200 rpm), then resuspended in 50 mM pH = 7 KPi buffer (ca. 200 mL) to a final OD_{600} = 30. The cell suspension was lysed by sonication at 50% amplitude for 4 min (1 s on, 4 s off) in an ice bath. The cell debris was pelleted by centrifugation (4 °C, 15 min, 4200 rpm), and the clarified lysate supernatant was diluted 1:1 with pH = 7 KPi (final volume ca. 400 mL) and partitioned equally into 2 x 1 L non-beveled Erlenmeyer flasks, each containing L-pipecolic acid (1.03 g, 8.00 mmol, 1 equiv, 40 mM final concentration), α-ketoglutaric acid (3.62 g, 16.0 mmol, 2 equiv), ascorbic acid (704 mg, 4.00 mmol, 0.5 equiv) and $FeSO_4$•$7H_2O$ (111 mg, 0.400 mmol, 0.05 equiv). The reaction mixtures were then shaken for 14 h at 200 rpm/22 °C in the open Erlenmeyer flasks. The reactions were then quenched by addition of 6 M HCl to final pH = 2 and centrifuged (4 °C, 15 min, 4200 RPM). The combined supernatants were concentrated to approx. 300 mL and directly subjected to the next reaction.

The supernatant was basified with 6 M NaOH to pH = 10. With rapid stirring, Boc_2O (8.73 g, 40.0 mmol, 2.5 equiv) dissolved in 150 mL EtOH was slowly added at room temperature. After 30 min, the mixture was adjusted back to pH = 10 with additional 6 M NaOH and allowed to stir

overnight or until completion as judged by LCMS. Ethanol was removed from the reaction mixture *in vacuo*, and the aqueous layer was adjusted to pH = 1 with 6 M HCl and extracted with 75 mL EtOAc. The aqueous layer was separated and extracted with an additional 9 x 50 mL EtOAc or until complete extraction as judged by TLC. The combined organic layers were dried over Na_2SO_4, filtered, and concentrated. The crude material was purified by flash chromatography (step gradient 94:5:1 to 90:9:1 DCM:MeOH:AcOH) followed by evaporation from 2 x 100 mL toluene to provide **20** (4.37 g, quantitative over 2 steps) as a white solid.

^{1}H NMR (600 MHz, $CDCl_3$): δ 6.29 (br s, 2H), 5.08 – 4.72 (m, 1H), 4.11 – 3.95 (m, 1H), 3.83 – 3.48 (m, 1H), 3.19 – 2.79 (m, 1H), 2.58 – 2.36 (m, 1H), 1.97 – 1.83 (m, 1H), 1.69 – 1.57 (m, 1H), 1.49 – 1.37 (m, 10H).

^{13}C NMR (151 MHz, $CDCl_3$): δ 175.8, 156.1, 155.6, 81.1, 81.0, 66.1, 54.7, 53.8, 40.8, 40.0, 35.2, 33.7, 28.5, 28.4.

HRMS (ESI): calculated for $C_{11}H_{20}NO_5^+$ ($[M+H]^+$) 246.1336, found 246.1335

$[\alpha]_D^{27}$ = –82.2 (*c* 0.33, MeOH)

(2*S*,4*S*)-1-(*tert*-butoxycarbonyl)-4-((*tert*-butyldimethylsilyl)oxy)piperidine-2-carboxylic acid (21)

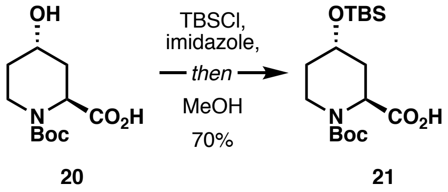

A 100 mL flame-dried round bottom flask was charged with **20** (1.97 g, 8.04 mmol, 1 equiv) and dissolved in 40 mL anhydrous DMF. Imidazole (1.37 g, 20.1 mmol, 2.5 equiv) then *tert*-butyldimethylsilyl chloride (2.67 g, 17.7 mmol, 2.2 equiv) were added as solids and the mixture was stirred under argon at rt overnight. An additional portion of imidazole (0.684 g, 10.0 mmol, 1.25 equiv) and *tert*-butyldimethylsilyl chloride (1.33 g, 8.84 mmol, 1.1 equiv) were then added and the reaction stirred a further 9 h or until complete consumption of **20** as indicated by TLC. The reaction mixture was concentrated *in vacuo*, then stirred in 20 mL MeOH for 5 h or until complete conversion to **21** as judged by TLC. The reaction was concentrated *in vacuo* then partitioned into 100 mL EtOAc and 50 mL 1 M HCl. The aqueous layer was separated then

extracted with 2 x 50 mL EtOAc. The combined organic layers were washed with brine then dried over Na_2SO_4, filtered and concentrated. The crude residue was purified by flash chromatography (97:3:0.2 DCM:MeOH:AcOH) and evaporated twice from toluene to provide **21** (2.01 g, 70% yield) as an off-white solid.

^{1}H NMR (600 MHz, $CDCl_3$): δ 5.06 – 4.77 (m, 1H), 4.13 – 3.91 (m, 1H), 3.70 – 3.62 (m, 1H), 3.09 – 2.85 (m, 1H), 2.43 – 2.28 (m, 1H), 1.87 – 1.73 (m, 1H), 1.70 – 1.60 (m, 1H), 1.51 – 1.41 (m, 10H), 0.88 (s, 9H), 0.06 (d, *J* = 3.0 Hz, 6H).

^{13}C NMR (151 MHz, $CDCl_3$): δ 177.0, 176.8, 156.1, 155.3, 80.9, 80.8, 66.8, 54.7, 53.8, 40.8, 40.1, 35.6, 34.6, 28.4, 25.9, 18.2, -4.50, -4.55.

HRMS (ESI): calculated for $C_{17}H_{34}NO_5Si^+$ ($[M+H]^+$) 360.2201, found 360.2200

$[\alpha]_D^{26}$ = –21.0 (*c* 0.42, MeOH)

(2*S*,4*R*)-1-(*tert*-butoxycarbonyl)-4-((*tert*-butyldimethylsilyl)oxy)-6-oxopiperidine-2-carboxylic acid (18)

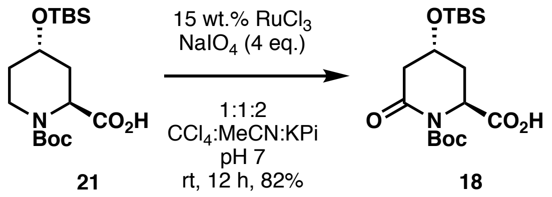

A 100 mL round bottom flask was charged with **21** (600 mg, 1.67 mmol, 1 equiv) dissolved in 11 mL of a 1:1:2 mixture of CCl_4:MeCN:0.5 M pH 7 KPi. With rapid stirring sodium periodate (1.43 g, 6.68 mmol, 4 equiv) was added, followed by $RuCl_3 \cdot xH_2O$ (90 mg, 15 wt. % with respect to **21**). The homogeneous mixture was stirred at rt overnight or until completion by TLC. The mixture was then diluted with 50 mL EtOAc and filtered through a celite plug. The filtrate was washed with 10 mL 1 M HCl and 4 x 10 mL saturated $Na_2S_2O_3$. The combined aqueous layers were then back-extracted with 50 mL EtOAc. The combined organic layers were washed with 20 mL 1:1 brine:1 M HCl, dried over Na_2SO_4, filtered and concentrated. The resulting tan-brown solids were dissolved in ~10 mL Et_2O, stirred with activated carbon for 30 min, and celite filtered to provide **18** (510 mg, 82% yield) as an off-white crystalline solid.

^{1}H NMR (600 MHz, CDCl$_3$): δ 4.75 (t, *J* = 6.1 Hz, 1H), 4.17 – 4.07 (m, 1H), 2.75 (ddd, *J* = 16.9, 4.8, 1.5 Hz, 1H), 2.51 (ddd, *J* = 17.0, 7.0, 1.0 Hz, 1H), 2.28 – 2.20 (m, 1H), 2.17 – 2.10 (m, 1H), 1.50 (s, 9H), 0.87 (s, 9H), 0.06 (d, *J* = 5.1 Hz, 6H).

^{13}C NMR (151 MHz, CDCl$_3$): δ 177.0, 168.8, 152.2, 84.4, 63.3, 55.8, 44.2, 34.4, 28.0, 25.8, 18.1, -4.7, -4.7.

HRMS (ESI): calculated for $C_{17}H_{32}NO_6Si^+$ ($[M+H]^+$) 374.1993, found 374.1990

$[\alpha]_D^{27}$ = +1.9 (*c* 0.36, MeOH)

(2*S*,4*R*)-2-((*tert*-butoxycarbonyl)amino)-4-((*tert*-butyldimethylsilyl)oxy)-6-hydroxyhexanoic acid (19)

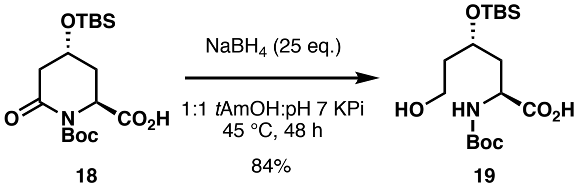

A 50 mL round bottom flask was charged with **18** (300 mg, 0.803 mmol, 1 equiv) dissolved in 2 mL *t*AmOH, followed by 2 mL 1.0 M pH 7 KPi buffer. With rapid stirring, solid $NaBH_4$ (304 mg, 8.03 mmol, 10 equiv) was added portion wise to control foaming. The mixture was stirred for 1 h at rt and then heated at 45 °C. After 7 h, additional $NaBH_4$ (456 mg, 12.1 mmol, 15 equiv) was added portion wise and the resulting slurry was stirred rapidly at 45 °C an additional 40 h or until completion by LCMS. The reaction mixture was carefully quenched at rt by adding 25 mL MeOH then minimal H_2O to dissolve any remaining solids, then stirring for 1 h. The mixture was concentrated *in vacuo* then partitioned into 30 mL 1 M HCl and 50 mL EtOAc. The aqueous layer was further extracted with 2 x 20 mL EtOAc. The combined organic layers were washed with brine and dried over Na_2SO_4, filtered and concentrated. The residue was purified by flash chromatography (95:5:1 DCM:MeOH:AcOH) and evaporated from toluene to provide **19** (255 mg, 84% yield) as a white solid.

^{1}H NMR (600 MHz, CDCl$_3$): δ 6.49 (br s, 2H), 5.54 (d, *J* = 7.1 Hz, 1H), 4.30 – 4.21 (m, 1H), 4.07 (p, *J* = 5.7 Hz, 1H), 3.77 (t, *J* = 5.9 Hz, 2H), 2.20 – 2.06 (m, 1H), 1.99 – 1.83 (m, 2H), 1.82 – 1.73 (m, 1H), 1.43 (s, 9H), 0.89 (s, 9H), 0.09 (s, 6H).

^{13}C NMR (151 MHz, CDCl$_3$): δ 176.2, 156.2, 80.5, 69.0, 59.5, 51.6, 39.6, 38.0, 28.4, 26.0, 18.1, -4.5, -4.6.

HRMS (ESI): calculated for $C_{17}H_{36}NO_6Si^+$ ($[M+H]^+$) 378.2306, found 378.2306

$[\alpha]_D^{27}$ = –7.9 (*c* 0.42, MeOH)

***tert*-butyl (*S*)-(1-(methoxy(methyl)amino)-1-oxopropan-2-yl)carbamate (22)**

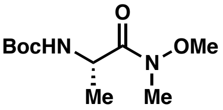

22

Weinreb amide **22** was prepared as described in a previously reported procedure.[4]

4-nitrobenzenesulfonamide (S1)

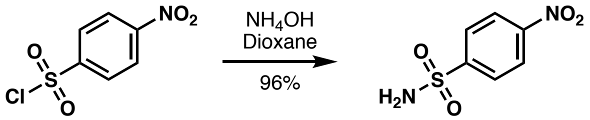

S1

A 100 mL round bottom flask was charged with 4-nitrobenzene sulfonyl chloride (4.00 g, 18.1 mmol, 1 equiv) dissolved in 36 mL DCM. Concentrated ammonium hydroxide (29%) was diluted to a 2M stock solution in dioxane (29.8 mL, 59.6 mmol, 3.3 equiv) and slowly added at rt. The reaction was stirred for 3.5 h or until completion by TLC. The mixture was concentrated *in vacuo* to dryness, and evaporated once from anhydrous dioxane. The residue was purified by flash chromatography (95:5 DCM:MeOH) to provide **S1** (3.53 g, 96% yield) as a light yellow solid.

^{1}H NMR (600 MHz, Methanol-*d*$_4$): δ 8.45 – 8.33 (m, 2H), 8.18 – 8.05 (m, 2H).

^{13}C NMR (151 MHz, Methanol-*d*$_4$): δ 151.2, 150.7, 128.6, 125.3.

HRMS (ESI): calculated for $C_6H_5N_2O_4S^-$ ($[M–H]^-$) 200.9976, found 200.9976

diethyl (2-((4-nitrophenyl)sulfonamido)-2-oxoethyl)phosphonate (23)

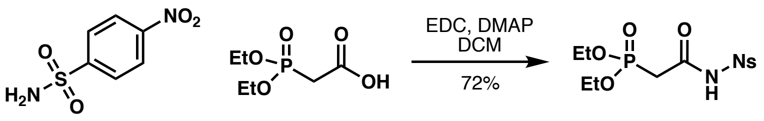

A flame-dried 250 mL round bottom flask was charged with 2-(diethoxyphosphoryl)acetic acid (3.25 g, 16.6 mmol, 1 equiv), **S1** (3.52 g, 17.4 mmol, 1.05 equiv) and 4-dimethylaminopyridine (2.03 g, 16.6 mmol, 1 equiv) in 70 mL anhydrous DCM and stirred to suspend. Solid EDC hydrochloride (6.36 g, 33.2 mmol, 2 equiv) was added and the reaction was stirred at rt under argon overnight or until completion by TLC. The reaction mixture was concentrated *in vacuo*. The residue was dissolved in 150 mL EtOAc and washed with 50 mL each of 1 M HCl, H_2O, saturated $NaHCO_3$, and brine. The combined aqueous layers were back-extracted once with 50 mL EtOAc and the combined organic layers were dried over Na_2SO_4, filtered and concentrated. The residue was purified by flash chromatography (step gradient 99:1 DCM:AcOH to 98:2:1 DCM:MeOH:AcOH to 95:5:1 DCM:MeOH:AcOH) to provide **23** (4.74 g, 72% yield) as a light yellow solid.

^{1}H NMR (600 MHz, $CDCl_3$): δ 11.14 (s, 1H), 8.40 – 8.25 (m, 4H), 4.16 – 4.06 (m, 4H), 3.00 – 2.94 (m, 2H), 1.29 (t, *J* = 7.1 Hz, 6H).

^{13}C NMR (151 MHz, $CDCl_3$): δ 162.91, 162.87, 150.85, 144.25, 130.18, 124.16, 63.92, 63.88, 36.78, 35.92, 16.37, 16.33.

HRMS (ESI): calculated for $C_{12}H_{18}N_2O_8PS^+$ ($[M+H]^+$) 381.0516, found 381.0514

tert-butyl (*S*)-(1-oxopropan-2-yl)carbamate (S2)

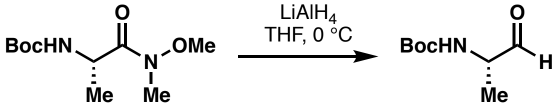

A flame-dried 100 mL round bottom flask was charged with $LiAlH_4$ (350 mg, 9.23 mmol, 1.1 equiv) and 4.6 mL anhydrous THF. The mixture was cooled to 0 °C and a solution of **22** (2.00 g, 8.39 mmol, 1 equiv) in 32 mL anhydrous THF was added dropwise. The reaction was stirred at 0 °C for 1 h or until completion by TLC. The reaction was carefully quenched at 0 °C with 9.3 mL of 1 M citric acid and stirred vigorously for 10 min. The layers were separated and the aqueous

phase extracted with 2 x 20 mL EtOAc. The combined organic layers were washed with 10 mL each of H_2O, $NaHCO_3$ and brine, then dried over Na_2SO_4, filtered and concentrated to dryness, providing **S2** (1.80 g) as a white solid. The product was used directly in the next reaction without further purification.

^{1}H NMR (400 MHz, $CDCl_3$): δ 9.55 (s, 1H), 5.27 – 4.90 (m, 1H), 4.32 – 4.04 (m, 1H), 1.44 (s, 9H), 1.32 (d, J = 7.4 Hz, 3H).

HRMS (ESI): calculated for $C_8H_{16}NO_3^+$ ($[M+H]^+$) 174.1125, found 174.1124

***tert*-butyl (*S*,*E*)-(5-((4-nitrophenyl)sulfonamido)-5-oxopent-3-en-2-yl)carbamate (24)**

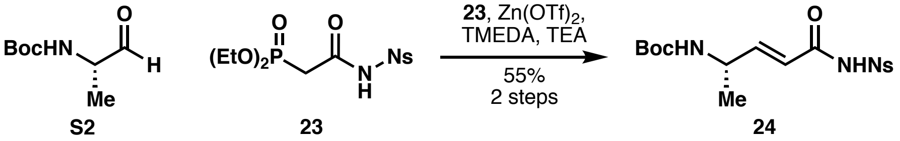

Following a procedure adapted from Helquist's report,[5] a flame-dried 100 mL round-bottom flask was charged with anhydrous zinc triflate (5.63 g, 15.5 mmol, 2.2 equiv) and **23** (2.68 g, 7.04 mmol, 1 equiv) and cycled 3x through high vacuum and argon purges. The mixture was suspended in 15 ml of anhydrous THF, and freshly distilled TMEDA (1.26 mL, 8.45 mmol, 1.2 eq) and TEA (2.16 mL, 15.5 mmol, 2.2 equiv) were added. A solution of aldehyde **S2** in 5 mL anhydrous THF was then added, and the mixture was rapidly stirred at rt under argon overnight or until completion by LCMS. The reaction was quenched with 10 mL 1 M HCl and diluted with 10 mL H_2O. THF was removed *in vacuo*, and the remaining aqueous phase was extracted with 5 x 30 mL EtOAc. The combined organic layers were dried over Na_2SO_4, filtered and concentrated. The residue was purified by flash chromatography (45:45:10 hexanes:EtOAc:MeOH) to provide **24** (1.38 g, 55% over 2 steps) as a light yellow solid.

^{1}H NMR (400 MHz, Methanol-d_4): δ 8.38 – 8.31 (m, 2H), 8.21 – 8.15 (m, 2H), 6.70 (dd, J = 15.5, 5.2 Hz, 1H), 5.91 (dd, J = 15.5, 1.7 Hz, 1H), 4.26 – 4.17 (m, 1H), 4.14 – 3.87 (m, 1H), 1.42 (s, 9H), 1.19 (d, J = 7.0 Hz, 3H).

^{13}C NMR (151 MHz, $CDCl_3$): δ 163.2, 155.5, 151.4, 150.8, 144.2, 130.1, 124.2, 120.1, 80.6, 47.7, 28.5, 20.2.

HRMS (ESI): calculated for $C_{16}H_{22}N_3O_7S^+$ ($[M+H]^+$) 400.1173, found 400.1172

$[\alpha]_D^{27}$ = –43.9 (*c* 0.46, MeOH)

(*S*,*E*)-4-amino-*N*-((4-nitrophenyl)sulfonyl)pent-2-enamide, trifluoroacetate salt (S3)

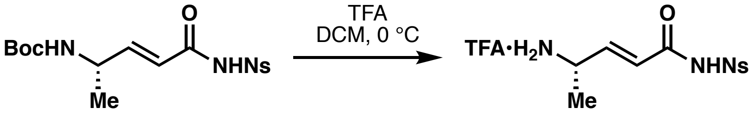

24 **S3**

A 25 mL vial was charged with **24** (100 mg, 0.250 mmol, 1 equiv) and dissolved in 1.75 mL DCM. The mixture was cooled to 0 °C and TFA (0.75 mL) was added dropwise. The reaction was stirred at 0 °C for 1 h or until completion by TLC. The mixture was diluted with 10 mL toluene and concentrated *in vacuo*, then evaporated twice more from toluene to provide **S3** (114 mg) which was used without further purification.

^{1}H NMR (400 MHz, Methanol-*d*$_4$): δ 8.46 – 8.40 (m, 2H), 8.30 – 8.24 (m, 2H), 6.84 (dd, *J* = 15.5, 6.6 Hz, 1H), 6.14 (dd, *J* = 15.6, 1.4 Hz, 1H), 4.14 – 4.00 (m, 2H), 1.40 (d, *J* = 6.8 Hz, 3H).

HRMS (ESI): calculated for $C_{11}H_{14}N_3O_5S^+$ ($[M+H]^+$) 300.0649, found 300.0648

***tert*-butyl ((2*S*,4*R*)-4-((*tert*-butyldimethylsilyl)oxy)-6-hydroxy-1-(((*S*,*E*)-5-((4-nitrophenyl)sulfonamido)-5-oxopent-3-en-2-yl)amino)-1-oxohexan-2-yl)carbamate (25)**

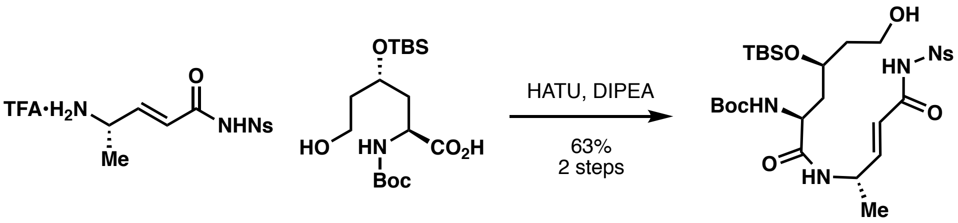

S3 **19** **25**

A flame-dried 8 mL vial was charged with **S3** (82 mg, 0.199 mmol, 1.5 equiv) and **19** (50 mg, 0.132 mmol, 1.0 equiv) and 1.7 mL anhydrous DMF. HATU (50 mg, 0.132 mmol, 1 equiv) was added followed by DIPEA (104 µL, 0.596 mmol, 4.5 equiv) and the mixture was stirred at rt overnight or until completion by TLC. The reaction was concentrated *in vacuo* to dryness. The residue was dissolved in 15 mL EtOAc and washed with 3 x 2 mL H_2O then 2 mL brine, dried

over Na_2SO_4, filtered and concentrated. The residue was purified by flash chromatography (93:7 DCM:MeOH) to provide **25** (55 mg, 63% over two steps) as an off-white solid.

^{1}H NMR (600 MHz, Methanol-*d*4): δ 8.42 – 8.38 (m, 2H), 8.25 – 8.20 (m, 2H), 6.76 (dd, *J* = 15.5, 5.4 Hz, 1H), 5.91 (dd, *J* = 15.4, 1.6 Hz, 1H), 4.58 – 4.49 (m, 1H), 4.06 (dt, *J* = 9.8, 5.0 Hz, 1H), 4.00 – 3.94 (m, 1H), 3.68 (ddd, *J* = 10.4, 7.0, 6.1 Hz, 1H), 3.62 (dt, *J* = 10.6, 6.8 Hz, 1H), 1.97 – 1.88 (m, 1H), 1.83 – 1.67 (m, 3H), 1.43 (s, 9H), 1.25 (d, *J* = 6.9 Hz, 3H), 0.90 (s, 9H), 0.08 (d, *J* = 2.4 Hz, 6H).

^{13}C NMR (151 MHz, Methanol-*d*4): δ 174.43, 174.35, 167.00, 157.65, 151.82, 149.68, 147.25, 130.65, 124.98, 123.12, 80.84, 69.31, 59.51, 54.03, 54.00, 47.28, 47.18, 40.76, 40.31, 28.70, 26.43, 19.64, 19.60, 18.85, -4.22, -4.46.

HRMS (ESI): calculated for $C_{28}H_{47}N_4O_{10}SSi^+$ ($[M+H]^+$) 659.2777, found 659.2776

$[\alpha]_D^{27}$ = –18.4 (*c* 0.32, MeOH)

***tert*-butyl (2*S*,4*S*)-4-((*tert*-butyldimethylsilyl)oxy)-2-(((*S*,*E*)-5-((4-nitrophenyl)sulfonamido)-5-oxopent-3-en-2-yl)carbamoyl)piperidine-1-carboxylate (26)**

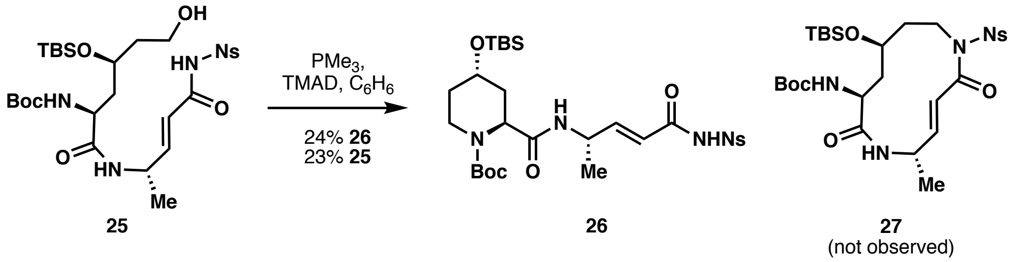

A flame-dried 25 mL vial was charged with Me_3P (5.8 mg, 0.076 mmol, 5 equiv) and tetramethylazodicarboxamide (13.1 mg, 0.076 mmol, 5 equiv) in 1.3 mL anhydrous benzene and allowed to stir for 10 min at rt. The mixture was then diluted with 5.0 mL anhydrous benzene and **25** (10 mg, 0.0152 mmol, 1 equiv) in 1.3 mL anhydrous THF was added slowly. The reaction was stirred at rt under argon overnight or until completion by TLC. The reaction was concentrated *in vacuo* to dryness and the residue was purified by preparative TLC (95:5 DCM:MeOH) to provide **26** (2.3 mg, 24%) and recovered **25** (2.2 mg, 23%).

^{1}H NMR (600 MHz, Methanol-*d*4, 45 °C): δ 8.44 – 8.35 (m, 2H), 8.29 – 8.20 (m, 2H), 6.75 (dd, *J* = 15.5, 6.4 Hz, 1H), 5.95 (dd, *J* = 15.5, 1.4 Hz, 1H), 4.58 – 4.52 (m, 1H), 3.99 – 3.93 (m, 1H), 3.74 (tt, *J* = 10.1, 4.6 Hz, 1H), 3.18 (td, *J* = 13.3, 3.2 Hz, 1H), 2.24 – 2.06 (m, 1H), 1.83 – 1.76 (m,

1H), 1.64 – 1.54 (m, 1H), 1.44 (s, 9H), 1.40 – 1.32 (m, 2H), 1.26 (d, *J* = 6.9 Hz, 3H), 0.84 (s, 9H), -0.02 (d, *J* = 5.6 Hz, 6H).

^{13}C NMR (151 MHz, Methanol-*d*$_4$, 45 °C): δ 173.31, 166.21, 157.37, 152.02, 149.85, 147.00, 130.76, 125.02, 123.12, 81.82, 67.43, 56.24, 47.22, 42.04, 37.55, 35.59, 28.62, 26.28, 19.66, 18.85, -4.50, -4.53.

HRMS (ESI): calculated for $C_{28}H_{45}N_4O_9SSi^+$ ($[M+H]^+$) 641.2671, found 641.2670

$[\alpha]_D^{27}$ = –35.6 (*c* 0.09, MeOH)

Table S1. Condition screening for Mitsunobu macrocyclization of **25**.

Reagents	Conditions	Result
PPh_3/DEAD	3:1 Toluene:THF, 0.01M, rt, 1d	decomposition
PPh_3/DEAD	THF, 0.002M, rt, 10 min	decomposition
PPh_3/(DEAD or DIAD)	pre-form betaines, 0.002M THF, rt, 3d	N.R.
PMe_3/TMAD (5 eq)	pre-form betaine, 0.002M PhH, rt, 1d	24% **26**, 23% **25**

Attempted differential amine protections of (2S,4S)-4-hydroxylysine

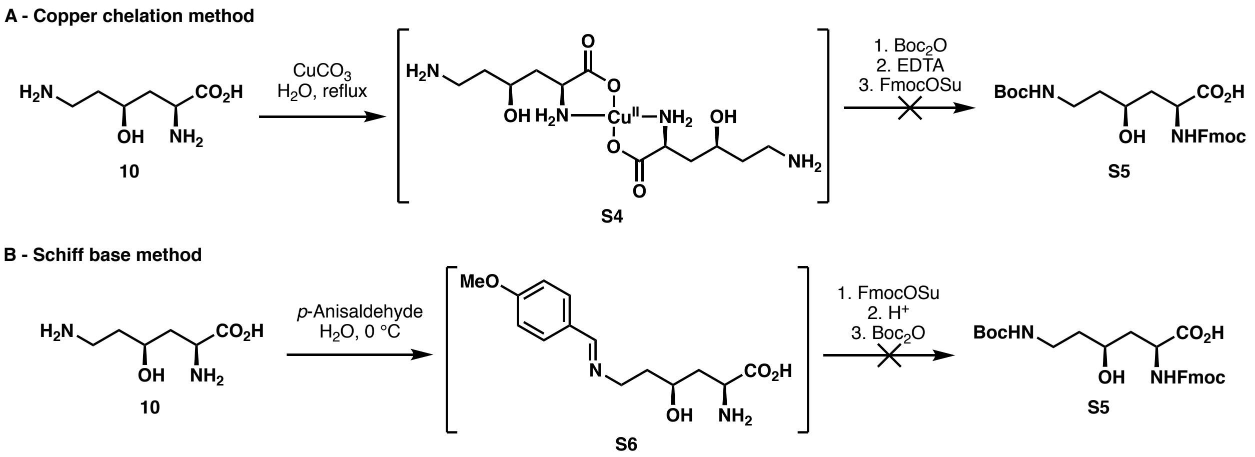

Scheme S1. (a) Differential lysine amine protections via in situ cuprate formation.[6] (b) Differential lysine amine protections via transient Schiff base intermediate.[7]

(2*S*,4*S*)-4-hydroxylysine **10** was prepared and isolated by cation exchange resin as described in a previously reported procedure.[4] Differential amine protections were attempted as described by Parrish[6] and Kishore[7] but did not produce **S4** or **S6** as isolable intermediates. One-pot protocols adapted from these procedures failed to selectively protect the amine functionalities and provide **S5** in significant/isolable quantities.

Screening of primary-selective alcohol oxidation conditions

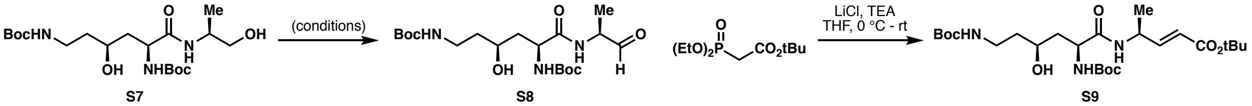

Scheme S2. Primary selective oxidation and subsequent Masamune-Roush modified HWE olefination.

Diol **S7** was prepared as described in a previously reported procedure.[8] Primary-selective alcohol oxidation conditions were performed as described in the literature and conversion to **S8** was screened by NMR, or crude **S8** was directly subjected to a modified Horner-Wadsworth Emmons olefination[9] for isolated yields of **S9**. Characterization data for **S9** is reported in a previous publication.[4]

Table S2. Screening conditions for primary-selective alcohol oxidation of **S7**.

Entry	Conditions	Outcome
1	0.1 eq TEMPO, 0.1 eq nBu_4NBr, 1.5 eq NCS, 1:1 DCM:$NaHCO_3$/K_2CO_3 pH 8.6 buffer	Full conversion of **S7**, 30% **S9** (2 steps)
2	0.1 eq TEMPO, 0.1 eq nBu_4NCl (recrystallized), 1.5 eq NCS, 1:1 DCM:$NaHCO_3$/K_2CO_3 pH 8.6 buffer	Incomplete conversion of **S7**, 29% **S9** (2 steps)
3	0.05 eq TEMPO, 0.05 eq nBu_4NCl, 1 eq NCS (portion wise), 1 eq NaBr, 1:1 DCM:$NaHCO_3$/K_2CO_3 pH 8.6 buffer (ref. 10)	~30% **S8**
4	0.01 eq TEMPO, 2.4 eq NaOCl, 0.1 eq KBr, 15 eq $NaHCO_3$, 0.007 M DCM (ref. 11)	~1.3:1 **S7**:**S8**
5	0.15 eq TEMPO, 0.9 eq PIDA, 0.1 M DCM, 0 °C (ref. 12)	Decomposed
6	0.01 eq TEMPO, 1.05 eq trichloroisocyanuric acid, 0.2 M DCM, rt (ref. 13)	0% **S9**
7	0.05 eq $Cu^{I}Br$, 0.05 eq bpy, 0.05 eq TEMPO, 0.1 eq NMI, 1 M MeCN, rt (ref. 14)	~1:1 **S7**:**S8**
8	1.05 eq DMP, 0.1 M THF, 0 °C	~1:1 **S7**:**S8**, messy

Initial proteomics results

Cepafungin alkyne **S10** was synthesized and proteomics experiments were performed as described previously.[4]

Cepafungin analog syntheses

Preliminary analog series:

Compounds **1**, **2**, **38** and **39** were synthesized as described previously (Figure 2).[4]

KDO3 Reaction Optimization:

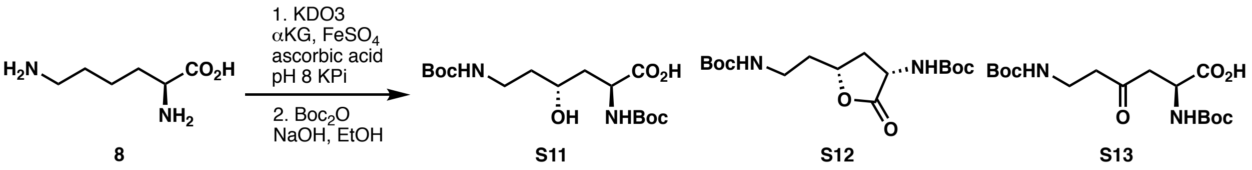

Scheme S3. Hydroxylation of L-lysine **8** with clarified lysate of *E. coli* expressing KDO3 and in situ Boc protection of the reaction supernatant.

Standard conditions:

A glycerol stock of *E. coli* BL21(DE3) cells harboring pET-28a(+)-KDO3 plasmid was used to inoculate an overnight culture of LB media (4 mL) containing 50 μg/mL kanamycin. 0.5 mL of this culture was used to inoculate 100 mL TB media containing 50 μg/mL kanamycin in a 500 mL non-beveled Erlenmeyer flask. The culture was shaken at 250 rpm at 37 °C for 2.5 h or until an OD_{600} = 0.6 was reached. The culture was cooled on ice (15 min), induced by adding IPTG to final concentration of 25 μM, then allowed to continue shaking at 250 rpm/22 °C for another 22 h. Cells were harvested by centrifugation (4 °C, 15 min, 4200 rpm), then resuspended in 50 mM pH = 8 KPi buffer (ca. 25 mL) to a final OD_{600} = 30. The cell suspension was lysed by sonication at 50% amplitude for 3 min (1 s on, 4 s off) in an ice bath. The cell debris was pelleted by centrifugation (4 °C, 15 min, 4200 rpm), and the clarified lysate supernatant was diluted with 50 mM pH = 8 KPi and added to a non-beveled Erlenmeyer flask (≥80% headspace) containing L-lysine (1 equiv), α-ketoglutaric acid (1.25 equiv), ascorbic acid (0.5 equiv) and $FeSO_4$•$7H_2O$ (0.1

equiv). The reaction mixture was then shaken for 10 h at 200 rpm/23 °C in the open Erlenmeyer flask. The reaction was then quenched by addition of 6 M HCl to final pH = 2 and centrifuged (4 °C, 15 min, 4200 RPM). The supernatant was directly subjected to the next reaction.

The supernatant was basified with 6 M NaOH to pH = 11. With rapid stirring, Boc_2O (4 equiv) dissolved in EtOH (final 2:1 H_2O:EtOH) was slowly added at rt. After 30 min, the mixture was adjusted back to pH = 10 with additional 6 M NaOH and allowed to stir overnight. The mixture was again adjusted back to pH = 10 with 6 M NaOH and Boc_2O (2 equiv) dissolved in minimal EtOH was slowly added at rt. The mixture was allowed to stir overnight or until completion as judged by LCMS. Ethanol was removed from the reaction mixture *in vacuo*. The aqueous phase was adjusted to pH = 1 with 6 M HCl and diluted with 75 mL EtOAc. The aqueous layer was separated and extracted with an additional 3 x 50 mL EtOAc. The combined organic layers were washed with brine and dried over Na_2SO_4, filtered, and concentrated. The crude material was purified by flash chromatography (97:3:1 DCM:MeOH:AcOH) followed by two evaporations from toluene to provide compounds **S11**–**13** as white solids.

Table S4. KDO3 reaction optimization. Clarified lysates generated from 100 mL cell culture at pre-lysis OD_{600} = 30 were diluted with varying volumetric equivalents of 50 mM pH 8 KPi and reacted with varying final concentrations of L-lysine substrate. KDO3 reactions in entries 1, 2 and 5–8 were performed under standard conditions. Co-substrate ratios and buffer conditions in entries 3 and 4 (products not isolated) are adapted from the procedure reported by Baud.[15]

Entry	Conditions[a]	NMR ratio **S11**:**S13**	Yield **S11**[b]	Yield **S12**[b]	Yield **S13**[b]	Yield (**S11**+**S12**)[b]
1	20 mM Lys, lysate diluted 2x	1.0:2.9	11%	6%	32%	17%
2	40 mM Lys, lysate diluted 2x	1.1:1.0	32%	7%	17%	39%
3	20 mM Lys, 1.5 eq αKG, 0.25 eq ascorbate, 0.1 eq $FeSO_4$. pH 7.5 HEPES. Lysate diluted 1x	Only **S13** + byproducts	-	-	-	-
4	40 mM Lys, 1.5 eq αKG, 0.25 eq ascorbate, 0.1 eq $FeSO_4$. pH 7.5 HEPES. Lysate diluted 1x	0.4:1.0	-	-	-	-
5	40 mM Lys, lysate diluted 3x	1.0:1.3	24%	17%	19%	41%
6	40 mM Lys, lysate diluted 4x	1.0:0.9	5%	30%	17%	35%
7	40 mM Lys, lysate diluted 5x	1.0:0.8	17%	22%	14%	39%
8	40 mM Lys (gram scale), lysate diluted 6x	1.0:0.5	20%	22%	12%	42%

[a] Standard conditions: (1) 1.25 eq. αKG, 0.1 eq. $FeSO_4 \cdot 7H_2O$, 0.5 eq. ascorbic acid, 50 mM pH 8 KPi, 200 RPM/23 °C, 10 h; HCl quench. (2) 6 eq. Boc_2O, NaOH to pH = 11, 2:1 H_2O:EtOH
[b] Isolated yield after purification by flash chromatography as described above.

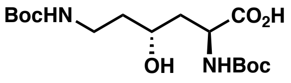

S11

(2*S*,4*R*)-2,6-bis((*tert*-butoxycarbonyl)amino)-4-hydroxyhexanoic acid (S11).

Prepared on gram-scale following standard conditions in entry 8, Table S2. L-lysine **8** (1.02 g, 7 mmol, 1 equiv), α-ketoglutaric acid (1.98 g, 8.75 mmol, 1.25 equiv), ascorbic acid (616 mg, 3.5 mmol, 0.5 equiv), Fe_2SO_4•$7H_2O$ (195 mg, 0.7 mmol, 0.1 equiv) and Boc_2O (9.17 g, 10.5 mmol, 6 equiv) were used to afford **S11** (500 mg, 20% over 2 steps) as a white solid.

^{1}H NMR (400 MHz, Methanol-*d*$_4$): δ 4.33 – 4.13 (m, 1H), 3.78 – 3.65 (m, 1H), 3.15 (td, *J* = 6.8, 2.7 Hz, 2H), 1.91 – 1.79 (m, 1H), 1.79 – 1.67 (m, 1H), 1.65 – 1.53 (m, 2H), 1.44 (d, *J* = 3.9 Hz, 18H).

^{13}C NMR (151 MHz, Methanol-*d*$_4$): δ 176.6, 158.8, 158.4, 80.5, 80.0, 66.5, 52.3, 39.8, 39.0, 38.1, 28.8, 28.7.

HRMS (ESI): calculated for $C_{16}H_{31}N_2O_7^+$ ($[M+H]^+$) 363.2126, found 363.2125

$[\alpha]_D^{24}$ = –3.8 (*c* 0.7, MeOH)

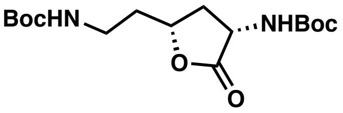

S12

***tert*-butyl (2-((2*R*,4*S*)-4-((*tert*-butoxycarbonyl)amino)-5-oxotetrahydrofuran-2-yl)ethyl)carbamate (S12)**.

Prepared on gram-scale following standard conditions in entry 8, Table S2. L-lysine **8** (1.02 g, 7 mmol, 1 equiv), α-ketoglutaric acid (1.98 g, 8.75 mmol, 1.25 equiv), ascorbic acid (616 mg, 3.5 mmol, 0.5 equiv), Fe_2SO_4•$7H_2O$ (195 mg, 0.7 mmol, 0.1 equiv) and Boc_2O (9.17 g, 10.5 mmol, 6 equiv) were used to afford **S12** (515 mg, 22% over 2 steps) as a white solid.

^{1}H NMR (400 MHz, Methanol-*d*$_4$): δ 4.54 – 4.24 (m, 2H), 3.28 – 3.12 (m, 2H), 2.61 (ddd, *J* = 12.1, 8.8, 5.5 Hz, 1H), 2.03 – 1.78 (m, 3H), 1.44 (d, *J* = 6.2 Hz, 18H).

^{13}C NMR (151 MHz, Methanol-*d*$_4$): δ 177.2, 158.5, 157.6, 80.8, 80.1, 77.0, 52.2, 37.7, 36.6, 35.8, 28.8, 28.7.

HRMS (ESI): calculated for $C_{16}H_{29}N_2O_6^+$ ($[M+H]^+$) 345.2020, found 345.2015

$[\alpha]_D^{27}$ = +12.7 (*c* 0.5, MeOH)

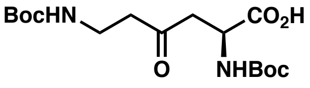

S13

(*S*)-2,6-bis((*tert*-butoxycarbonyl)amino)-4-oxohexanoic acid (S13).

Prepared on gram-scale following standard conditions in entry 8, Table S2. L-lysine **8** (1.02 g, 7 mmol, 1 equiv), α-ketoglutaric acid (1.98 g, 8.75 mmol, 1.25 equiv), ascorbic acid (616 mg, 3.5 mmol, 0.5 equiv), $Fe_2SO_4•7H_2O$ (195 mg, 0.7 mmol, 0.1 equiv) and Boc_2O (9.17 g, 10.5 mmol, 6 equiv) were used to afford **S13** (292 mg, 12% over 2 steps) as a white solid.

^{1}H NMR (600 MHz, Methanol-*d*$_4$): δ 4.53 – 4.37 (m, 1H), 3.28 (t, *J* = 6.5 Hz, 2H), 3.02 – 2.84 (m, 2H), 2.74 – 2.58 (m, 2H), 1.43 (d, *J* = 10.2 Hz, 18H).

^{13}C NMR (151 MHz, Methanol-*d*$_4$): δ 208.16, 175.09, 158.35, 157.83, 80.65, 80.13, 50.60, 45.08, 43.62, 36.29, 28.75, 28.70.

HRMS (ESI): calculated for $C_{16}H_{29}N_2O_7^+$ ($[M+H]^+$) 361.1969, found 361.1967

$[\alpha]_D^{24}$ = –2.2 (c 1.0, MeOH)

Expanded analog series:

General Procedure A: aminolysis of 4-hydroxylysine lactones

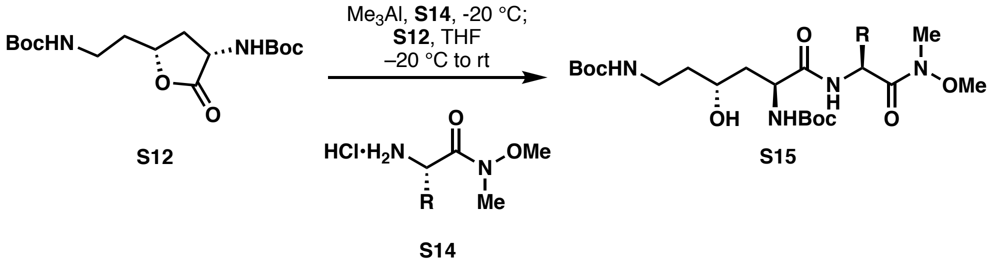

Amine hydrochloride **S14** (5 equiv) was set stirring in anhydrous THF (0.4 M). The mixture was cooled to –20 °C, then a 2 M solution of trimethylaluminum (4.95 equiv) in anhydrous toluene was added dropwise. The reaction was stirred under argon at –20 °C for 30 minutes to provide a clear, light-yellow solution. Lactone **S12** (0.4 M, 1 equiv) in anhydrous THF was added dropwise at –20 °C. The reaction was stirred to room temperature over 28 h or until completion

by TLC. The reaction was cooled to 0 °C and quenched by dropwise addition of 2 M $NaHSO_4$ solution. The mixture was adjusted to pH = 1 with 1 M HCl, then concentrated *in vacuo* to remove THF. The aqueous phase was extracted three times with ethyl acetate. The combined organic layers were then washed with H_2O, $NaHCO_3$, H_2O, brine and dried over Na_2SO_4, filtered and concentrated. The residue was purified by flash chromatography using a step gradient of hexanes/EtOAc/MeOH mixture as eluent to provide **S15** as a white solid.

General Procedure B: reduction of Weinreb amides

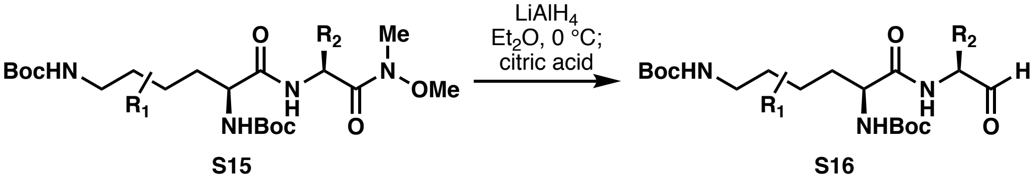

Weinreb amide **S15** (1 equiv, 0.1 M) was set stirring in anhydrous Et_2O and cooled to 0 °C. 1 M $LiAlH_4$ solution (2.5 equiv) in Et_2O was added dropwise. The mixture was stirred at 0 °C under argon for 30 minutes or until completion by TLC, then quenched at 0 °C by dropwise addition of 2 M citric acid (2.5 equiv). The mixture was diluted with 10% v/v H_2O and stirred for 5 min. The mixture was diluted with additional H_2O and Et_2O, then the layers were separated. The aqueous layer was extracted three times with Et_2O, and the combined organic layers were washed with H_2O, $NaHCO_3$, brine and dried over Na_2SO_4, filtered and concentrated to provide **S16** as white solid (d.r. ≥ 98:2) used immediately without further purification.

General Procedure C: Wittig olefination

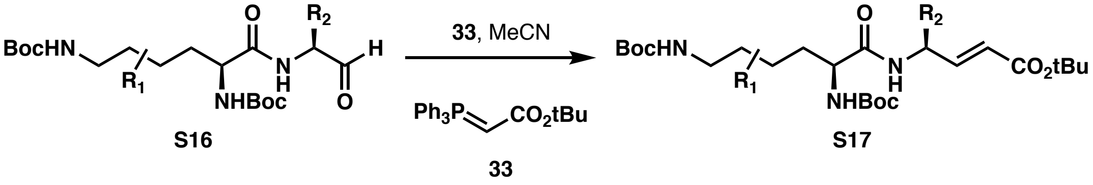

Ylide **33** (1.25 equiv) was dissolved in freshly-distilled anhydrous acetonitrile (0.2 M), then a solution of aldehyde **S16** (1 equiv) in acetonitrile (0.2 M) was added dropwise at room temperature. The reaction was stirred for 30 minutes or until completion by TLC, and then

concentrated to dryness. Purification by silica flash chromatography using a mixture of DCM/EtOAc/MeOH as eluent provided **S17** as an off-white solid.

General Procedure D: global deprotection

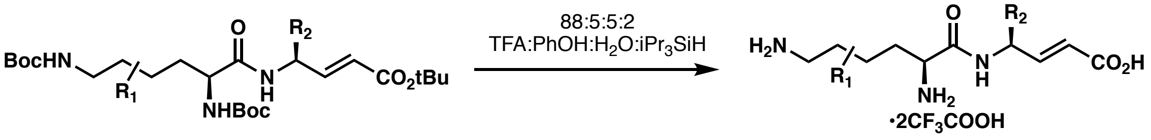

S17 **S18**

Dipeptide **S17** (1 equiv) was dissolved (0.04 M) in a freshly prepared solution of ''Reagent B'' (88:5:5:2 TFA:PhOH:H_2O:*i*Pr_3SiH) and stirred for 1 hour at rt or until completion by LCMS. The reaction was diluted with toluene and concentrated *in vacuo* to dryness, then evaporated twice more from 30 mL toluene. The residue was dissolved in minimal MeOH and triturated into diethyl ether at 0 °C. The solids were collected by centrifugation (4 °C, 10 min, 4200 rpm), then triturated into ether and centrifuged as above once more, then dried from MeOH to provide pure **S18** as an off-white solid.

General Procedure E: macrolactamization

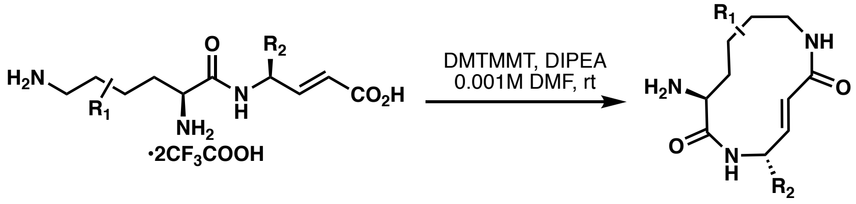

S18 **S19**

Linear macrocycle precursor **S18** (1 equiv) was dissolved anhydrous DMF (0.001 M) and treated with diisopropylethylamine (4 equiv) then 4-(4,6-dimethoxy-1,3,5-triazin-2-yl)-4-methyl-morpholinium tetrafluoroborate (DMTMMT) (1.50 equiv) at rt. The reaction was stirred under argon for 36 hours, then quenched with 10% v/v H_2O and concentrated *in vacuo* to dryness. The residue was dissolved in minimal methanol and triturated into diethyl ether with rapid stirring. The solids were collected by centrifugation (4 °C, 15 min, 4200 rpm) and dried from MeOH to provide **S19** as a tan solid. Yield was determined by ^1H NMR analysis with 4-toluenesulfonamide as internal standard.

General Procedure F: fragment coupling

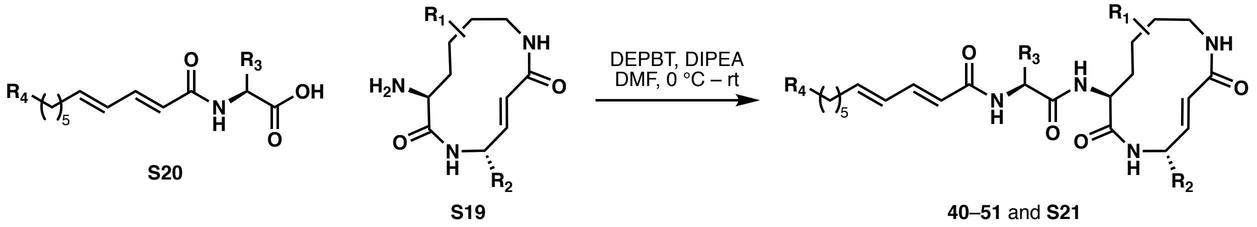

Carboxylic acid **S20** (2 equiv.) and crude macrocycle **S19** (1 equiv as judged by ^{1}H NMR analysis) were dissolved in anhydrous DMF (0.08 M) and cooled to 0 °C. To the mixture was added diisopropylethylamine (6 equiv), followed by DEPBT (2 equiv). The reaction was stirred to room temperature overnight or until completion by LCMS. The reaction was quenched at 0 °C 30 % v/v H_2O then concentrated *in vacuo* to dryness. The residue was triturated into 50 mL Et_2O at 0 °C from minimal MeOH, and the resulting solids were collected by centrifugation (4 °C, 15 min, 4200 rpm). The solids were dissolved in DMSO, filtered through a 0.2 mm PTFE membrane filter, then purified by preparative reversed-phase HPLC using a gradient of MeCN/H_2O/0.1% formic acid mixtures over 35 minutes to provide **40–51** and **S21** as fluffy white powders after lyophilization.

General Procedure G: Kochi coupling

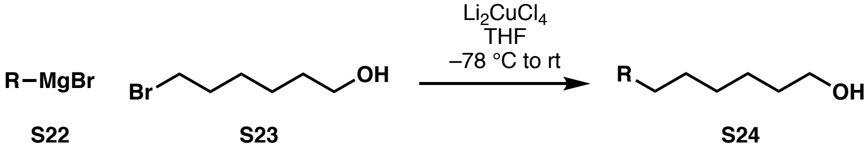

Following a procedure adapted from Melaugh et al.,[16] lithium chloride (2.05 equiv) was flame-dried in a round-bottom flask under high vacuum and purged with argon (3x). Solid copper(II) chloride (1 equiv) was added under argon. The combined reagents were then dissolved in anhydrous THF (0.7 M with respect to $CuCl_2$) with rapid stirring to provide a dark red solution of Li_2CuCl_4.

Grignard reagent **S22** was used as provided from commercial sources or prepared as described previously.[4] A flame-dried flask was charged with **S22** (6.5 equiv) in anhydrous THF (1 M). The mixture was cooled to –78 °C and **S23** (1 equiv) was added in anhydrous THF (7 M), followed by the above solution of Li_2CuCl (1.1 equiv with respect to **S24**). Upon complete

addition, the reaction was removed from its cooling bath and allowed to warm to room temperature. Reaction progress was monitored by TLC until completion at 1 h. The reaction was then carefully quenched at 0 °C with saturated NH_4Cl and filtered through a sintered glass funnel. The filtrate was concentrated *in vacuo* to remove most of the THF, then diluted with ethyl acetate and 1 M HCl to a final pH = 1. The layers were separated, and the aqueous phase was extracted three times with ethyl acetate. The combined organic layers were washed with H_2O, $NaHCO_3$ and brine, then dried over Na_2SO_4, filtered and concentrated. Purification by silica flash chromatography using hexanes/EtOAc as eluent provided pure **S24** as a colorless liquid.

General Procedure H: Swern oxidation

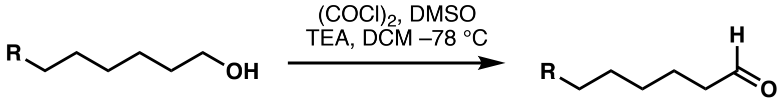

S24 **S25**

Oxalyl chloride (1.5 equiv) was dissolved in anhydrous DCM (1.3 M) at –78 °C. A solution of anhydrous DMSO (3 equiv) in anhydrous DCM (7 M) was added dropwise and the resulting mixture was stirred at –78 °C for 15 minutes. A solution of **S24** (1 equiv) in anhydrous DCM (1 M) was then added dropwise over 10 minutes. The mixture was stirred at –78 °C for 1 hour then treated with triethylamine (5 equiv) dropwise. The resulting thick slurry was diluted with additional DCM to aid stirring, and reaction progress was monitored by TLC until completion at 1 hour. The reaction was quenched at –78 °C with H_2O and allowed to stir to room temperature. The layers were separated, and the aqueous layer was extracted three times with DCM. The combined organic layers were washed with H_2O, brine, then dried over Na_2SO_4 and filtered through a plug of silica with ether as eluent. Concentration *in vacuo* provided **S25** as a colorless oil which was used immediately in the next reaction (General Procedure I) without further purification.

General Procedure I: Allylic HWE

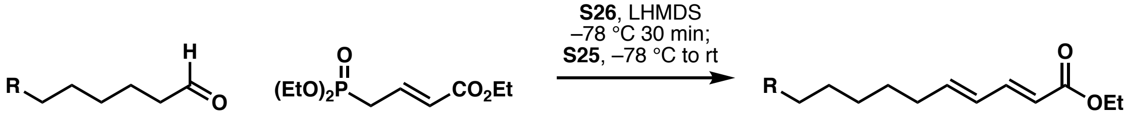

S25 **S26** **S27**

In a procedure adapted from Imker et al.,[17] **S26** (1.5 equiv) in anhydrous THF (0.5 M) was cooled to –78 °C and treated with lithium hexamethyldisilazide (1 M THF, 1.4 equiv) dropwise, then allowed to stir under argon at –78 °C for 30 min. Aldehyde **S25** (1 equiv) in anhydrous THF (1.4 M) was added dropwise at –78 °C and the mixture was allowed to stir to room temperature overnight or until completion by TLC. The reaction was quenched at 0 °C with saturated NH_4Cl solution. The layers were separated and the aqueous layer was extracted three times with ethyl acetate. The combined organic layers were washed with H_2O, $NaHCO_3$, brine and dried over Na_2SO_4, filtered and concentrated. Purification by silica flash chromatography using hexanes/Et_2O as eluent provided **S27** as a light yellow oil.

General Procedure J: ester hydrolysis

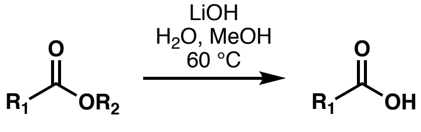

S27 **S28**

Ester **S27** (1 equiv) was dissolved in MeOH (0.2 M). With rapid stirring, an aqueous solution of lithium hydroxide (1 M, 5 equiv) was added, and the reaction was heated to 60 °C for 5 hours or until completion by TLC. Methanol was evaporated *in vacuo*, then the mixture was adjusted to pH 1 with 1 M HCl and diluted with 20 mL DCM. The layers were separated, and the aqueous layer was extracted three times with DCM. The combined organic layers were washed with brine, dried over Na_2SO_4, filtered and concentrated to provide pure **S28** as a waxy white solid.

General Procedure K: amide coupling

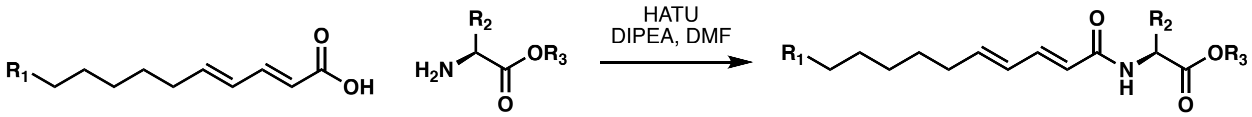

S28 **S29** **S30**

Fatty acid **S28** (1 equiv) was combined with amine **S29** (1–1.5 equiv) in anhydrous DMF (0.2 M). Diisopropylethylamine was added (1–3 eq), followed by HATU (1.05 equiv). The reaction was stirred at room temperature under argon for 4 hours or until completion by LCMS.

The reaction was then quenched with 10% v/v H_2O and concentrated *in vacuo*. The residue was dissolved in ethyl acetate and washed with 1M HCl (2x), H_2O, $NaHCO_3$, and brine, then dried over Na_2SO_4, filtered and concentrated. Purification by flash chromatography using hexanes:EtOAc as eluent provided **S30** as a colorless oil.

General Procedure L: tail fragment deprotection

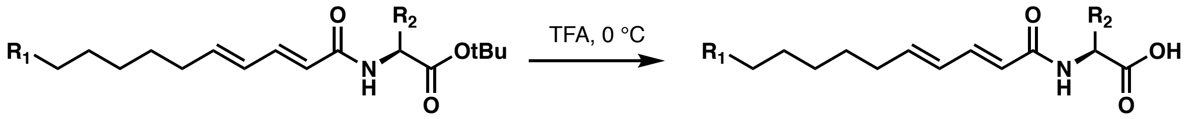

S30 **S31**

Amide **S30** (1 equiv) was dissolved in TFA (0.1 M) at 0 °C and stirred for 5 min, then stirred at rt for 1 hour. The reaction was then diluted with toluene and concentrated *in vacuo* to dryness. The residue was evaporated twice more from toluene to provide **S31** as a pale yellow resin.

Analog Synthesis Procedures

Synthesis of epi-4-OH cepafungin (**40**)

Scheme S4. Synthesis route to epi-4-OH cepafungin (**40**)

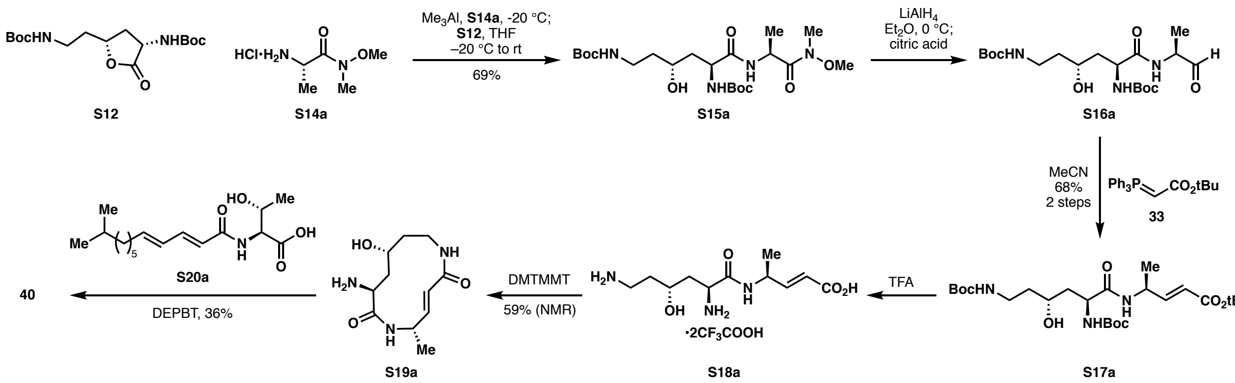

di-*tert*-butyl ((3*R*,5*S*)-3-hydroxy-6-(((*S*)-1-(methoxy(methyl)amino)-1-oxopropan-2-yl)amino)-6-oxohexane-1,5-diyl)dicarbamate (S15a)

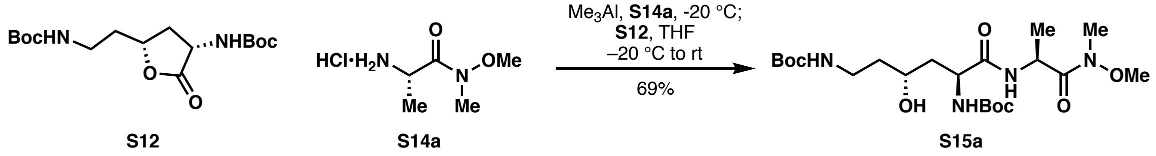

Lactone **S12** was prepared as described above in Table S2, entry 8. Amine **S14a** was prepared as described in a previous report.[4] Following General Procedure A: **S12** (638 mg, 1.85 mmol, 1 equiv), **S14a** (1.56 g, 9.26 mmol, 5 equiv) and Me_3Al (4.58 mL 2 M toluene, 9.16 mmol, 4.95 equiv) in THF (28 mL) were used to produce **S15a** (609 mg, 69%) as a white solid.

^{1}H NMR (600 MHz, $CDCl_3$): δ 7.10 – 6.92 (m, 1H), 6.01 – 5.72 (m, 1H), 4.95 – 4.84 (m, 1H), 4.36 (q, *J* = 6.3, 5.8 Hz, 1H), 3.76 (s, 4H), 3.41 (qd, *J* = 8.5, 5.5, 4.6 Hz, 1H), 3.20 (s, 3H), 3.10 (dt, *J* = 14.2, 5.4 Hz, 1H), 2.96 (s, 1H), 1.79 (t, *J* = 6.5 Hz, 2H), 1.64 – 1.49 (m, 2H), 1.46 – 1.40 (m, 19H), 1.34 (d, *J* = 6.9 Hz, 3H).

^{13}C NMR (151 MHz, $CDCl_3$): δ 172.8, 171.4, 157.0, 156.6, 80.4, 79.6, 66.5, 61.7, 52.3, 46.0, 40.9, 37.8, 37.5, 32.3, 28.5, 28.4, 18.4.

HRMS (ESI): calculated for $C_{21}H_{41}N_4O_8^+$ ($[M+H]^+$) 477.2919, found 477.2920

$[\alpha]_D^{27}$ = –24.4 (*c* 1.4, MeOH)

di-*tert*-butyl ((3*R*,5*S*)-3-hydroxy-6-oxo-6-(((*S*)-1-oxopropan-2-yl)amino)hexane-1,5-diyl)dicarbamate (S16a)

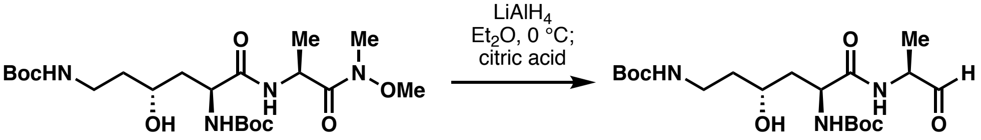

S15a S16a

Following General Procedure B: **S15a** (300 mg, 0.630 mmol, 1 equiv) and LAH (60 mg, 1.57 mmol, 2.5 equiv) in Et_2O (7.9 mL) were used to provide **S16a** (323 mg) as a white solid.

^{1}H NMR (400 MHz, $CDCl_3$): δ 9.53 (s, 1H), 7.24 (d, *J* = 5.0 Hz, 1H), 6.12 (d, *J* = 7.4 Hz, 1H), 4.91 (s, 1H), 4.68 (s, 1H), 4.45 – 4.34 (m, 2H), 3.84 – 3.78 (m, 1H), 3.13 – 3.01 (m, 1H), 1.98 – 1.88 (m, 1H), 1.87 – 1.73 (m, 2H), 1.64 – 1.48 (m, 2H), 1.43 (d, *J* = 7.0 Hz, 18H), 1.35 (d, *J* = 7.4 Hz, 3H).

HRMS (ESI): calculated for $C_{19}H_{36}N_3O_7^+$ ($[M+H]^+$) 418.2548, found 418.2553

***tert*-butyl (*S*,*E*)-4-((2*S*,4*R*)-2,6-bis((*tert*-butoxycarbonyl)amino)-4-hydroxyhexanamido)pent-2-enoate (S17a)**

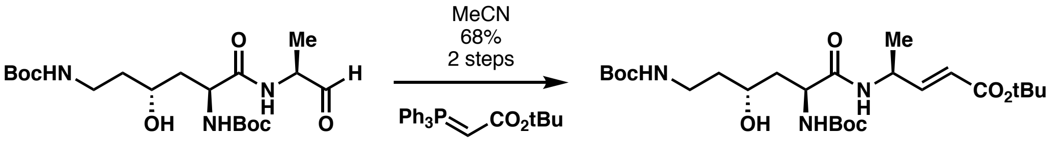

S16a 33 S17a

Following General Procedure C: **S16a** (94 mg, 0.225 mmol, 1 equiv), **33** (106 mg, 0.281 mmol, 1.25 equiv) in MeCN (3.2 mL) were used to provide **S17a** (80 mg, 68% over 2 steps) as a white solid.

^{1}H NMR (600 MHz, Methanol-*d*$_4$): δ 6.77 (dd, *J* = 15.7, 4.9 Hz, 1H), 5.81 (dd, *J* = 15.7, 1.7 Hz, 1H), 4.63 – 4.56 (m, 1H), 4.27 – 4.15 (m, 1H), 3.75 – 3.66 (m, 1H), 3.15 (td, *J* = 6.8, 4.6 Hz, 2H), 1.79 – 1.68 (m, 2H), 1.63 – 1.55 (m, 2H), 1.48 – 1.42 (m, 27H), 1.28 (d, *J* = 7.1 Hz, 3H).

^{13}C NMR (151 MHz, Methanol-*d*$_4$): δ 174.88, 167.38, 158.82, 157.96, 149.24, 122.79, 81.72, 80.76, 80.03, 66.53, 53.70, 46.91, 40.42, 39.11, 38.11, 28.83, 28.77, 28.35, 19.91.

HRMS (ESI): calculated for $C_{25}H_{46}N_3O_8^+$ ($[M+H]^+$) 516.3279, found 516.3289

$[\alpha]_D^{27}$ = –18.2 (*c* 1.1, MeOH)

(*S*,*E*)-4-((2*S*,4*R*)-2,6-diamino-4-hydroxyhexanamido)pent-2-enoic acid (S18a)

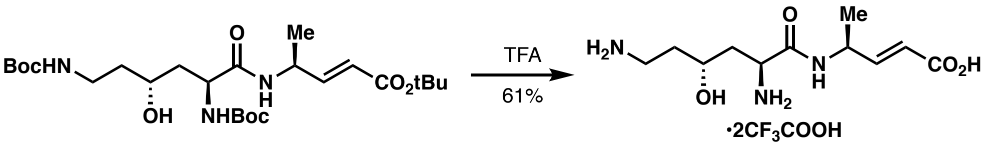

Following General Procedure D: **S17a** (336 mg, 0.652 mmol, 1 equiv) was used to provide **S18a** (194 mg, 61%) as a white solid.

^{1}H NMR (400 MHz, Methanol-*d*$_4$): δ 6.84 (dd, *J* = 15.7, 5.3 Hz, 1H), 5.86 (dd, *J* = 15.7, 1.6 Hz, 1H), 4.70 – 4.61 (m, 1H), 4.08 (t, *J* = 5.7 Hz, 1H), 3.94 – 3.84 (m, 1H), 3.15 – 3.00 (m, 2H), 2.08 – 1.98 (m, 2H), 1.92 – 1.75 (m, 2H), 1.33 (d, *J* = 7.0 Hz, 3H).

^{13}C NMR (101 MHz, Methanol-*d*$_4$): δ 170.0, 169.3, 149.3, 122.5, 66.9, 52.4, 47.4, 38.7, 38.0, 35.8, 19.7.

HRMS (ESI): calculated for $C_{11}H_{22}N_3O_4^+$ ([M+H]$^+$) 260.1605, found 260.1606

$[\alpha]_D^{27}$ = –17.4 (*c* 0.5, MeOH)

(5*S*,8*S*,10*R*,*E*)-8-amino-10-hydroxy-5-methyl-1,6-diazacyclododec-3-ene-2,7-dione (S19a)

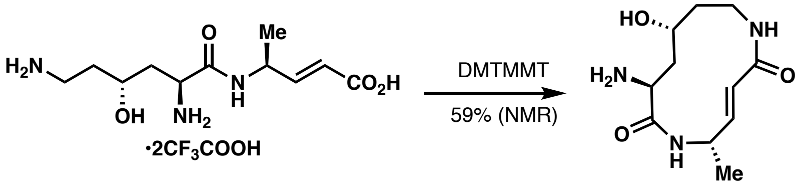

Following General Procedure E: **S18a** (163 mg, 0.335 mmol, 1 equiv) was used to provide **S19a** (276 mg, 59%) as a tan solid. Yield was determined by ^{1}H NMR analysis of a 6.2 mg sample of crude **S19a** and 5.1 mg 4-toluenesulfonamide added as internal standard.

^{1}H NMR (400 MHz, Methanol-*d*$_4$): 4-toluenesulfonamide: δ 7.85 – 7.70 (m, 2H), 7.41 – 7.28 (m, 2H), 2.42 (s, 3H). Compound **S19a**: δ 7.07 (ddd, *J* = 15.3, 11.8, 4.8 Hz, 1H), 6.29 (ddd, *J* = 15.4, 7.8, 1.3 Hz, 1H), 4.69 – 4.60 (m, 1H), 4.56 (q, *J* = 6.5 Hz, 1H), 3.49 – 3.35 (m, 2H), 2.49 (dd, *J* = 16.1, 3.7 Hz, 1H), 2.11 – 1.96 (m, 1H), 1.87 (ddd, *J* = 15.7, 10.5, 3.4 Hz, 1H), 1.80 – 1.58 (m, 3H).

HRMS (ESI): calculated for $C_{11}H_{20}N_3O_3^+$ ([M+H]$^+$) 242.1499, found 242.1495

(2*E*,4*E*)-*N*-((2*S*,3*R*)-3-hydroxy-1-(((5*S*,8*S*,10*R*,*E*)-10-hydroxy-5-methyl-2,7-dioxo-1,6-diazacyclododec-3-en-8-yl)amino)-1-oxobutan-2-yl)-11-methyldodeca-2,4-dienamide (40)

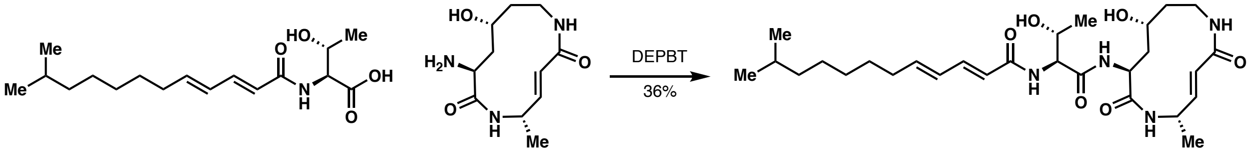

S20a **S19a** **40**

Following General Procedure F: Carboxylic acid **S20a** was synthesized as described previously.[4] **S20a** (38 mg, 0.121 mmol, 2 equiv), **S19a** (85 mg, 0.0604 mmol by ^{1}H NMR analysis, 1 equiv), DEPBT (36 mg, 0.121 mmol, 2 equiv) and DIPEA (63 µL, 0.362 mmol, 6 equiv) were used to provide **40** as a fluffy white solid (11.6 mg, 36%).

^{1}H NMR (600 MHz, DMSO-d_6): δ 8.42 (d, *J* = 7.8 Hz, 1H), 8.07 (d, *J* = 7.7 Hz, 1H), 7.92 (d, *J* = 8.6 Hz, 1H), 7.42 (dd, *J* = 8.0, 6.1 Hz, 1H), 6.99 (dd, *J* = 15.1, 10.7 Hz, 1H), 6.79 (dd, *J* = 15.3, 4.7 Hz, 1H), 6.22 – 6.04 (m, 4H), 4.97 (d, *J* = 3.3 Hz, 1H), 4.84 (d, *J* = 5.7 Hz, 1H), 4.48 (dt, *J* = 7.7, 3.8 Hz, 1H), 4.38 (tdd, *J* = 12.3, 6.3, 3.8 Hz, 1H), 4.26 (dd, *J* = 8.6, 4.0 Hz, 1H), 4.03 – 3.93 (m, 1H), 3.55 – 3.47 (m, 1H), 3.20 – 3.10 (m, 1H), 3.02 – 2.92 (m, 1H), 2.43 – 2.33 (m, 1H), 2.12 (q, *J* = 7.2 Hz, 2H), 1.58 – 1.47 (m, 3H), 1.47 – 1.41 (m, 1H), 1.41 – 1.35 (m, 2H), 1.28 – 1.22 (m, 4H), 1.20 (d, *J* = 7.1 Hz, 3H), 1.16 – 1.11 (m, 2H), 1.03 (d, *J* = 6.3 Hz, 3H), 0.84 (d, *J* = 6.6 Hz, 6H).

^{13}C NMR (151 MHz, DMSO-d_6): δ 170.9, 169.3, 165.5, 165.4, 147.3, 142.0, 139.7, 128.6, 123.0, 118.3, 66.5, 64.4, 58.2, 51.8, 45.7, 41.1, 38.4, 37.9, 34.8, 32.2, 28.9, 28.4, 27.4, 26.6, 22.5, 19.4, 18.3.

HRMS (ESI): calculated for $C_{28}H_{47}N_4O_6^+$ ([M+H]$^+$) 535.3490, found 535.3474

$[\alpha]_D^{27}$ = –51.5 (*c* 0.1, MeOH)

Synthesis of 3-OH-cepafungin (**41**)

Scheme S5. Synthesis route to 3-OH-cepafungin (**41**).

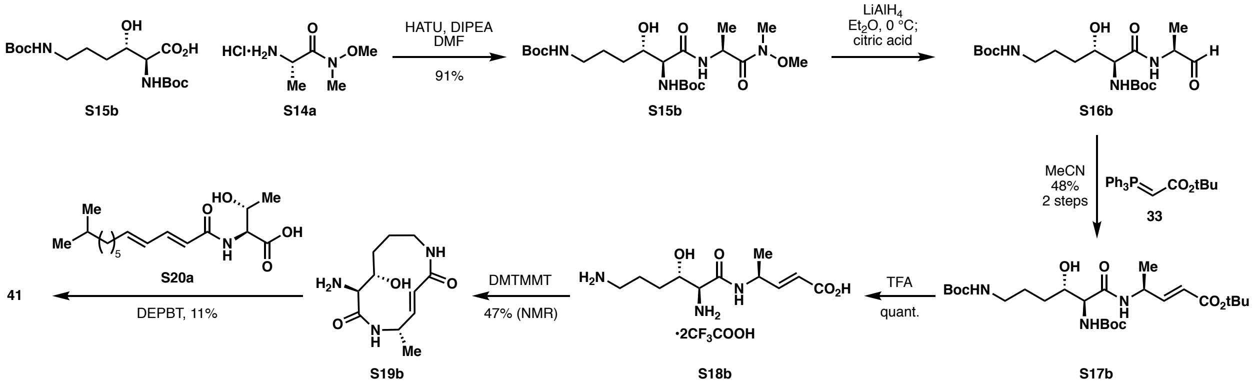

di-*tert*-butyl ((4*S*,5*S*)-4-hydroxy-6-(((*S*)-1-(methoxy(methyl)amino)-1-oxopropan-2-yl)amino)-6-oxohexane-1,5-diyl)dicarbamate (S15b)

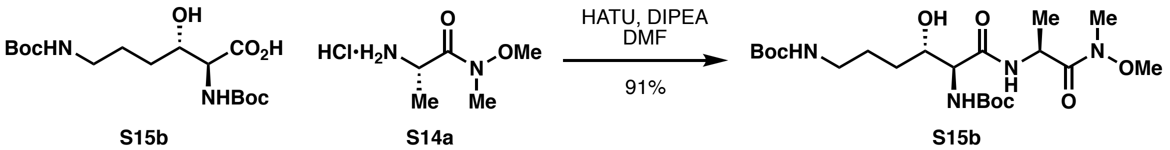

Compound **S15b** was prepared as described in a previously reported procedure using lysine hydroxylase KDO1.[18] Amine **S14a** was prepared as described previously.[4] Following General Procedure K: **S15b** (1.00 g, 2.76 mmol, 1 equiv), **S14a** (698 mg, 4.14 mmol, 1.5 equiv), HATU (1.10 g, 2.90 mmol, 1.05 equiv) and DIPEA (1.44 mL, 8.28 mmol, 3 equiv) were used to provide **S15b** (1.20 g, 91%) as a white solid.

^{1}H NMR (400 MHz, Methanol-*d*$_4$): δ 4.83 (d, J = 7.2 Hz, 1H), 4.11 – 3.92 (m, 1H), 3.82 (s, 3H), 3.76 – 3.68 (m, 1H), 3.21 (s, 3H), 3.14 – 2.98 (m, 2H), 1.71 – 1.49 (m, 3H), 1.49 – 1.37 (m, 19H), 1.34 (d, J = 7.1 Hz, 3H).

^{13}C NMR (101 MHz, Methanol-*d*$_4$): δ 174.7, 173.1, 158.6, 157.8, 80.8, 79.8, 72.4, 62.1, 60.4, 47.5, 41.1, 32.5, 31.2, 28.9, 28.7, 27.1, 17.1.

HRMS (ESI): calculated for $C_{21}H_{41}N_4O_8^+$ ([M+H]$^+$) 477.2919, found 477.2916

$[\alpha]_D^{27}$ = –28.1 (c 0.5, MeOH)

di-*tert*-butyl ((4*S*,5*S*)-4-hydroxy-6-oxo-6-(((*S*)-1-oxopropan-2-yl)amino)hexane-1,5-diyl)dicarbamate (S16b)

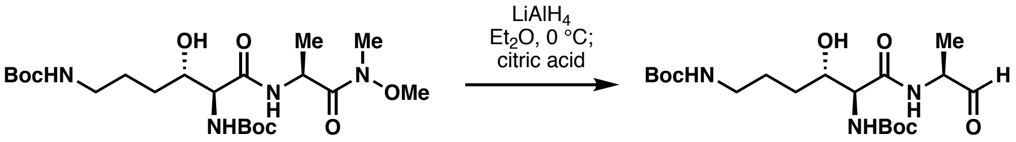

S15b **S16b**

Following General Procedure B: **S15b** (500 mg, 1.05 mmol, 1 equiv) and LAH (149 mg, 3.94 mmol, 3.75 equiv) were used to provide **S16b** (332 mg) as a white solid used without further purification.

***tert*-butyl (*S*,*E*)-4-((2*S*,3*S*)-2,6-bis((*tert*-butoxycarbonyl)amino)-3-hydroxyhexanamido)pent-2-enoate (S17b)**

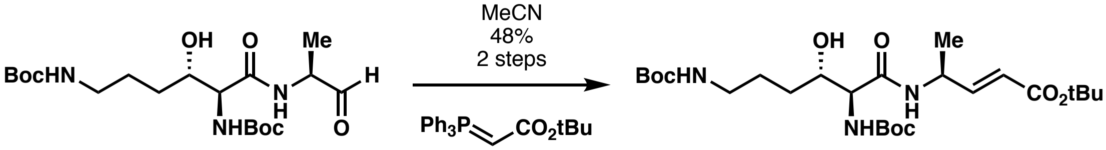

S16b **33** **S17b**

Following General Procedure C: **S16b** (332 mg) and **33** (374 mg, 0.994 mmol, 1.25 equiv) were used to provide **S17b** (262 mg, 48% over 2 steps) as a white solid.

^{1}H NMR (400 MHz, Methanol-*d*$_4$): δ 6.78 (dd, *J* = 15.6, 4.8 Hz, 1H), 5.89 (dd, *J* = 15.7, 1.8 Hz, 1H), 4.66 – 4.57 (m, 1H), 4.00 (d, *J* = 6.9 Hz, 1H), 3.74 (ddd, *J* = 9.7, 6.9, 2.6 Hz, 1H), 3.05 (td, *J* = 6.6, 3.9 Hz, 2H), 1.74 – 1.49 (m, 4H), 1.47 (s, 9H), 1.46 (s, 9H), 1.43 (s, 9H), 1.29 (d, *J* = 7.1 Hz, 3H).

^{13}C NMR (101 MHz, Methanol-*d*$_4$): δ 172.7, 167.5, 158.6, 157.7, 149.2, 122.9, 81.7, 80.8, 79.9, 72.1, 60.9, 47.0, 41.2, 31.7, 28.8, 28.7, 28.4, 27.2, 19.9.

HRMS (ESI): calculated for $C_{25}H_{46}N_3O_8^+$ ([M+H]$^+$) 516.3279, found 516.3277

$[\alpha]_D^{27}$ = –33.5 (*c* 1.1, MeOH)

(*S*,*E*)-4-((2*S*,3*S*)-2,6-diamino-3-hydroxyhexanamido)pent-2-enoic acid (S18b)

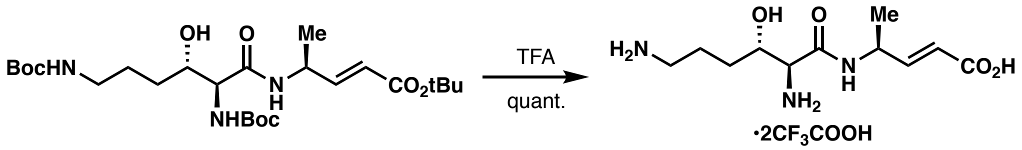

S17b **S18b**

Following General Procedure D: **S17b** (100 mg, 0.194 mmol, 1 equiv) was used to provide **S18b** (100 mg, ~quant.) as a white solid.

^{1}H NMR (400 MHz, Methanol-*d*$_4$): δ 6.87 (dd, *J* = 15.7, 5.3 Hz, 1H), 5.88 (dd, *J* = 15.7, 1.6 Hz, 1H), 4.67 (qdd, *J* = 7.0, 5.1, 1.7 Hz, 1H), 4.07 – 3.99 (m, 1H), 3.93 (d, *J* = 4.9 Hz, 1H), 2.97 (ddt, *J* = 8.9, 6.7, 3.5 Hz, 2H), 2.00 – 1.87 (m, 1H), 1.82 – 1.69 (m, 1H), 1.55 – 1.48 (m, 2H), 1.33 (d, *J* = 7.0 Hz, 3H).

^{13}C NMR (101 MHz, Methanol-*d*$_4$): δ 169.6, 167.0, 149.8, 122.0, 70.0, 59.2, 47.4, 40.5, 29.6, 25.4, 19.6.

HRMS (ESI): calculated for $C_{11}H_{22}N_3O_4^+$ ([M+H]$^+$) 260.1605, found 260.1611

$[\alpha]_D^{24}$ = –6.6 (*c* 1.2, MeOH)

(5*S*,8*S*,9*S*,*E*)-8-amino-9-hydroxy-5-methyl-1,6-diazacyclododec-3-ene-2,7-dione (S19b)

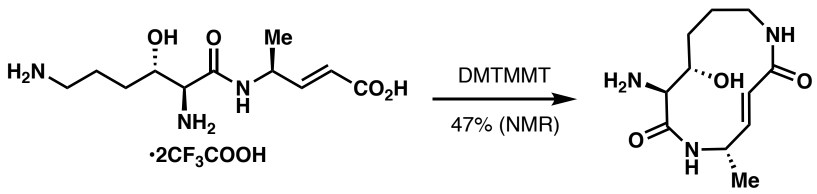

S18b **S19b**

Following General Procedure E: **S18b** (80 mg, 0.164 mmol, 1 equiv) was used to provide **S19b** (57 mg, 47%) as a tan solid. Yield was determined by ^{1}H NMR analysis of a 6.2 mg sample of crude **S18b** and 6.5 mg of 4-toluenesulfonamide added as internal standard.

^{1}H NMR (400 MHz, DMSO-*d*$_6$): 4-toluenesulfonamide: δ 7.70 (d, *J* = 7.8 Hz, 2H), 7.36 (d, *J* = 7.8 Hz, 2H), 7.27 (br s, 2H), 2.37 (s, 3H). Compound **S19b**: δ 8.61 (d, *J* = 7.4 Hz, 1H), 6.78 (d, *J* = 14.2 Hz, 1H), 6.19 (d, *J* = 15.1 Hz, 1H), 4.98 – 4.72 (m, 2H), 4.45 – 4.26 (m, 2H), 3.06 – 2.92 (m, 1H), 1.73 (d, *J* = 11.4 Hz, 1H), 1.56 – 1.46 (m, 1H).

HRMS (ESI): calculated for $C_{11}H_{20}N_3O_3^+$ ([M+H]$^+$) 242.1499, found 242.1497

(2*E*,4*E*)-*N*-((2*S*,3*R*)-3-hydroxy-1-(((5*S*,8*S*,9*S*,*E*)-9-hydroxy-5-methyl-2,7-dioxo-1,6-diazacyclododec-3-en-8-yl)amino)-1-oxobutan-2-yl)-11-methyldodeca-2,4-dienamide (41)

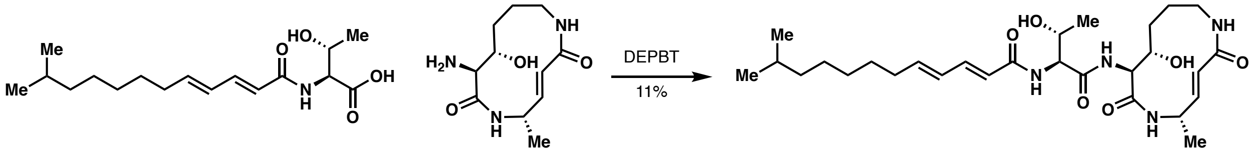

S20a **S19a** **41**

Following General Procedure F: Carboxylic acid **S20a** was synthesized as described previously.[4] **S20a** (39 mg, 0.125 mmol, 2 equiv), **S19a** (38 mg, 0.0626 mmol as judged by ^{1}H NMR, 1 equiv), DEPBT (37 mg, 0.125 mmol, 2 equiv) and DIPEA (65 µL, 0.375 mmol, 6 equiv) were used to provide **41** (3.6 mg, 11%) as a fluffy white solid.

^{1}H NMR (600 MHz, DMSO-*d*$_6$): δ 8.61 (d, *J* = 7.5 Hz, 1H), 7.96 (d, *J* = 8.4 Hz, 1H), 7.68 (d, *J* = 7.9 Hz, 1H), 7.35 (dd, *J* = 8.3, 6.0 Hz, 1H), 6.99 (dd, *J* = 15.1, 10.8 Hz, 1H), 6.78 (dd, *J* = 15.3, 4.7 Hz, 1H), 6.21 – 6.13 (m, 3H), 6.12 – 6.06 (m, 1H), 4.97 (d, *J* = 4.8 Hz, 1H), 4.80 (d, *J* = 6.5 Hz, 1H), 4.70 (dd, *J* = 7.9, 3.9 Hz, 1H), 4.44 (dd, *J* = 8.4, 4.3 Hz, 1H), 4.39 – 4.32 (m, 1H), 4.31 – 4.22 (m, 1H), 4.03 – 3.90 (m, 1H), 3.03 – 2.90 (m, 1H), 2.12 (q, *J* = 7.2 Hz, 2H), 1.71 (td, *J* = 12.4, 4.5 Hz, 1H), 1.55 – 1.45 (m, 1H), 1.38 (p, *J* = 7.2 Hz, 2H), 1.34 – 1.28 (m, 2H), 1.27 – 1.23 (m, 4H), 1.19 (d, *J* = 7.1 Hz, 3H), 1.16 – 1.12 (m, 3H), 1.04 (d, *J* = 6.3 Hz, 3H), 0.84 (d, *J* = 6.6 Hz, 6H).

^{13}C NMR (151 MHz, DMSO-*d*$_6$): δ 170.7, 169.2, 165.6, 165.4, 146.9, 142.0, 139.7, 128.6, 123.1, 118.7, 67.5, 67.0, 58.0, 57.7, 45.9, 38.4, 37.8, 32.2, 28.9, 28.4, 27.4, 26.6, 26.4, 25.0, 22.5, 19.1, 18.2.

HRMS (ESI): calculated for $C_{28}H_{47}N_4O_6^+$ ([M+H]$^+$) 535.3490, found 535.3488

$[\alpha]_D^{27}$ = –32.2 (*c* 0.1, MeOH)

Synthesis of keto-cepafungin **42**

Scheme S6. Synthesis route to keto-cepafungin **42**.

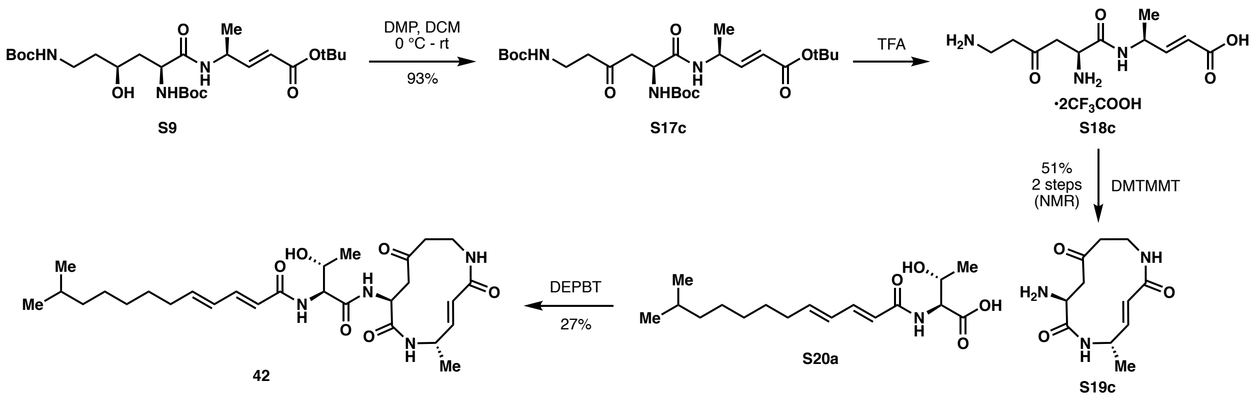

***tert*-butyl (*S*,*E*)-4-((*S*)-2,6-bis((*tert*-butoxycarbonyl)amino)-4-oxohexanamido)pent-2-enoate (S17c)**

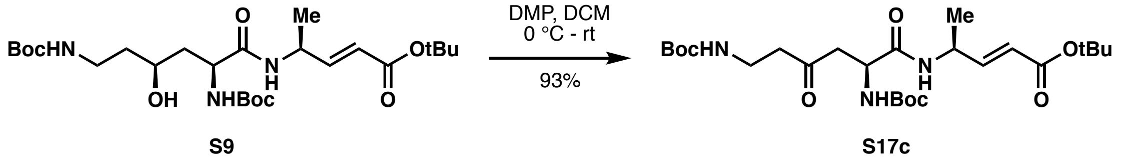

Compound **S9** was synthesized as previously reported.[4] A 50 mL round-bottom flask was charged with **S9** (171 mg, 0.332 mmol, 1 equiv), dissolved in 17 mL anhydrous DCM, and cooled to 0 °C. Dess-Martin Periodinane (169 mg, 0.398 mmol, 1.2 equiv) was added and the reaction was stirred under argon to rt overnight. The mixture was diluted with 6 mL Et_2O, filtered through a plug of celite and concentrated. The residue was purified by flash chromatography (70:29:1 DCM:EtOAc:MeOH) to provide **S17c** (159 mg, 93%) as a white solid.

^{1}H NMR (400 MHz, Methanol-*d*$_4$): δ 6.77 (dd, *J* = 15.7, 4.8 Hz, 1H), 5.83 (dd, *J* = 15.7, 1.8 Hz, 1H), 4.62 – 4.52 (m, 1H), 4.42 (d, *J* = 6.6 Hz, 1H), 3.28 (t, *J* = 7.1 Hz, 2H), 2.87 (tt, *J* = 17.4, 9.5 Hz, 2H), 2.65 (t, *J* = 6.5 Hz, 2H), 1.47 (s, 9H), 1.46 (s, 9H), 1.42 (s, 9H), 1.28 (d, *J* = 7.1 Hz, 3H).

^{13}C NMR (101 MHz, Methanol-*d*$_4$): δ 208.0, 173.5, 167.4, 158.3, 157.7, 149.1, 122.9, 81.7, 80.9, 80.1, 52.0, 47.1, 45.0, 43.7, 36.4, 28.8, 28.7, 28.4, 19.8.

HRMS (ESI): calculated for $C_{25}H_{44}N_3O_8^+$ ([M+H]$^+$) 514.3123, found 514.3122

$[\alpha]_D^{27}$ = –17.3 (*c* 1.5, MeOH)

(S,E)-4-((S)-2,6-diamino-4-oxohexanamido)pent-2-enoic acid (S18c)

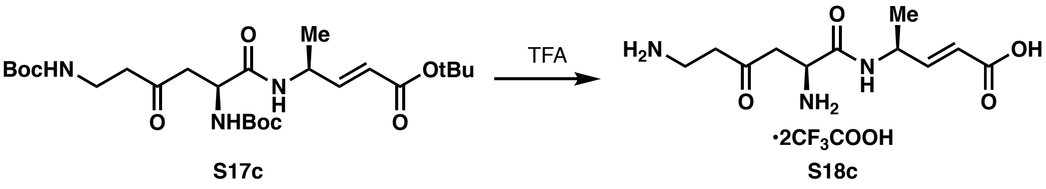

Following General Procedure D: **S17c** (76 mg, 0.148 mmol, 1 equiv) was used to provide **S18c** (71 mg) as a white solid used without further purification.

^{1}H NMR (600 MHz, Methanol-d_4): δ 6.81 (dd, J = 15.7, 5.1 Hz, 1H), 5.84 (dd, J = 15.7, 1.7 Hz, 1H), 4.62 (ddt, J = 10.5, 7.1, 3.6 Hz, 1H), 4.25 – 4.19 (m, 1H), 3.26 – 3.10 (m, 4H), 2.98 (q, J = 6.1 Hz, 2H), 1.57 (dd, J = 11.8, 7.0 Hz, 1H), 1.32 (d, J = 7.1 Hz, 3H).

HRMS (ESI): calculated for $C_{11}H_{20}N_3O_4^+$ ($[M+H]^+$) 258.1448, found 258.1447

(5S,8S,E)-8-amino-5-methyl-1,6-diazacyclododec-3-ene-2,7,10-trione (S19c)

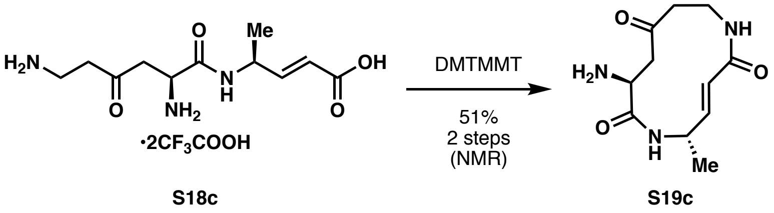

Following General Procedure E: **S18c** (58 mg, 0.119 mmol, 1 equiv) was used to provide **S19c** (72 mg, 51% over 2 steps) as a tan solid. Yield was determined by ^{1}H NMR analysis of a 2.4 mg sample of crude **S19c** and 2.8 mg of 4-toluenesulfonamide added as internal standard.

^{1}H NMR (400 MHz, Methanol-d_4): 4-toluenesulfonamide: δ 7.86 – 7.63 (m, 2H), 7.41 – 7.26 (m, 2H), 2.42 (s, 3H). Compound **S19c**: δ 6.86 (dd, J = 15.5, 5.1 Hz, 1H), 6.00 – 5.92 (m, 1H), 4.67 – 4.56 (m, 1H), 3.16 – 3.11 (m, 1H), 3.03 – 2.92 (m, 1H), 1.54 (s, 1H), 1.31 (d, J = 7.2 Hz, 3H).

HRMS (ESI): calculated for $C_{11}H_{18}N_3O_3^+$ ($[M+H]^+$) 240.1343, found 240.1333

(2*E*,4*E*)-*N*-((2*S*,3*R*)-3-hydroxy-1-(((5*S*,8*S*,*E*)-5-methyl-2,7,10-trioxo-1,6-diazacyclododec-3-en-8-yl)amino)-1-oxobutan-2-yl)-11-methyldodeca-2,4-dienamide (42)

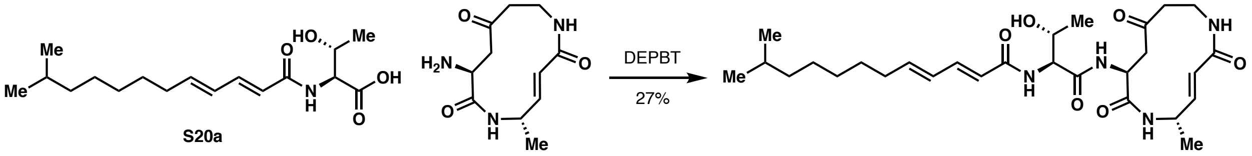

Following General Procedure F: Compound **S20a** was synthesized by a previously reported procedure.[4] **S20a** (38 mg, 0.122 mmol, 2 equiv), **S19c** (69 mg, 0.0612 mmol as judged by ^{1}H NMR analysis, 1 equiv), DEPBT (37 mg, 0.122 mmol, 2 equiv) and DIPEA (64 µL, 0.367 mmol, 6 equiv) were used to provide **42** (4.2 mg, 27%) as a fluffy white solid.

^{1}H NMR (600 MHz, DMSO-*d*$_6$): δ 8.60 (d, *J* = 7.1 Hz, 1H), 7.94 (d, *J* = 8.6 Hz, 1H), 7.80 (d, *J* = 7.9 Hz, 1H), 7.51 (dd, *J* = 7.1, 5.4 Hz, 1H), 7.00 (dd, *J* = 15.1, 10.8 Hz, 1H), 6.61 (dd, *J* = 15.4, 4.9 Hz, 1H), 6.22 – 6.06 (m, 3H), 5.72 (dd, *J* = 15.4, 1.4 Hz, 1H), 4.93 (d, *J* = 5.1 Hz, 1H), 4.66 (ddd, *J* = 10.2, 7.9, 4.0 Hz, 1H), 4.37 – 4.26 (m, 2H), 4.00 – 3.91 (m, 1H), 3.62 (dq, *J* = 14.9, 4.7 Hz, 1H), 3.04 – 2.95 (m, 1H), 2.95 – 2.82 (m, 2H), 2.67 (dd, *J* = 13.9, 4.0 Hz, 1H), 2.39 (dt, *J* = 18.4, 4.4 Hz, 1H), 2.13 (q, *J* = 7.2 Hz, 2H), 1.53 – 1.46 (m, 1H), 1.38 (p, *J* = 7.2 Hz, 2H), 1.29 – 1.22 (m, 4H), 1.17 – 1.11 (m, 5H), 1.00 (d, *J* = 6.3 Hz, 3H), 0.84 (d, *J* = 6.6 Hz, 6H).

^{13}C NMR (151 MHz, DMSO-*d*$_6$): δ 207.3, 169.7, 169.3, 166.3, 165.5, 146.0, 142.1, 139.8, 128.6, 123.0, 119.5, 66.7, 58.2, 50.4, 46.8, 45.7, 45.6, 38.4, 34.3, 32.2, 28.8, 28.4, 27.4, 26.6, 22.5, 19.8, 18.2.

HRMS (ESI): calculated for $C_{28}H_{45}N_4O_6^+$ ([M+H]$^+$) 533.3334, found 533.3321

$[\alpha]_D^{27}$ = –81.5 (*c* 0.1, MeOH)

Synthesis of valinyl cepafungin (**43**)

Scheme S7. Synthetic route to valinyl cepafungin **43**.

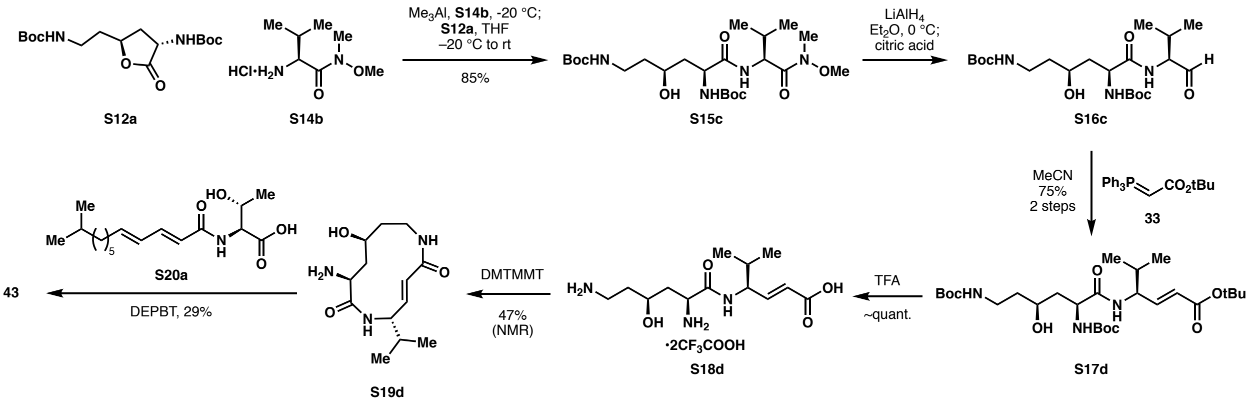

(*S*)-2-amino-*N*-methoxy-*N*,3-dimethylbutanamide (S14b)

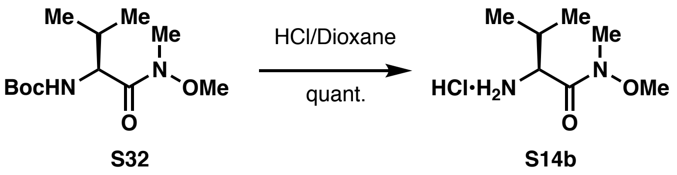

Following a previously reported procedure,[4] a flame-dried 100 mL round-bottom flask was charged with **S32** (3.85 g, 14.8 mmol, 1 equiv) and dissolved in 24 mL 4 M HCl/dioxane solution. The mixture was stirred under argon at rt for 1 h, then concentrated in vacuo to dryness. The residue was then evaporated from 50 mL 1:1 MeOH:toluene, then twice again from 50 mL toluene to provide **S14b** (3.19 g, quant.) as a clear, colorless solid used without further purification.

^{1}H NMR (400 MHz, Methanol-*d*$_4$): δ 4.22 (d, *J* = 5.3 Hz, 1H), 3.80 (s, 3H), 3.27 (s, 3H), 2.28 (dhept, *J* = 7.1, 5.4 Hz, 1H), 1.05 (dd, *J* = 22.6, 7.0 Hz, 6H).

^{13}C NMR (101 MHz, Methanol-*d*$_4$): δ 169.8, 62.3, 56.9, 32.3, 30.8, 19.2, 17.4.

HRMS (ESI): calculated for $C_7H_{17}N_2O_2^+$ ([M+H]$^+$) 161.1285, found 161.1277

$[\alpha]_D^{24}$ = –0.9 (*c* 1.2, MeOH)

di-*tert*-butyl ((3*S*,5*S*)-3-hydroxy-6-(((*S*)-1-(methoxy(methyl)amino)-3-methyl-1-oxobutan-2-yl)amino)-6-oxohexane-1,5-diyl)dicarbamate (S15c)

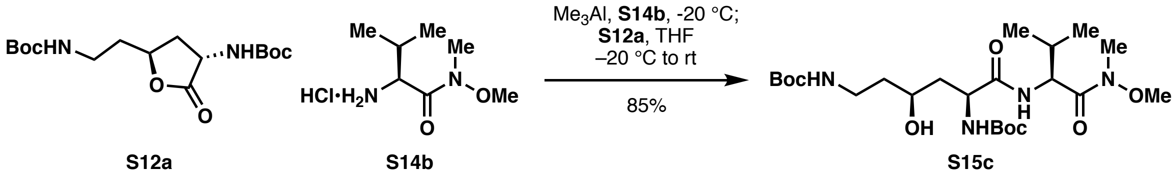

Compound **S12a** was synthesized as described previously.[4] Following General Procedure A: **S12a** (1.02 g, 2.96 mmol, 1 equiv) and **S14b** (3.19 g, 14.8 mmol, 5 equiv) were used to provide **S15c** (1.27 g, 85%) as a white solid.

^{1}H NMR (400 MHz, Methanol-*d*$_4$): δ 4.81 (d, *J* = 6.5 Hz, 1H), 4.20 (t, *J* = 7.4 Hz, 1H), 3.81 (s, 3H), 3.72 – 3.63 (m, 1H), 3.21 (s, 3H), 3.18 – 3.11 (m, 2H), 2.07 (hept, *J* = 6.8 Hz, 1H), 1.93 – 1.78 (m, 1H), 1.72 – 1.60 (m, 2H), 1.57 – 1.50 (m, 1H), 1.48 – 1.39 (m, 18H), 0.95 (t, *J* = 6.6 Hz, 6H).

^{13}C NMR (101 MHz, Methanol-*d*$_4$): δ 174.9, 173.6, 158.6, 157.7, 80.7, 80.0, 67.3, 62.1, 55.4, 53.7, 40.6, 38.4, 38.1, 32.2, 32.1, 28.8, 28.7, 19.6, 18.4.

HRMS (ESI): calculated for $C_{23}H_{45}N_4O_8^+$ ([M+H]$^+$) 505.3232, found 505.3231

$[\alpha]_D^{27}$ = –37.7 (*c* 0.6, MeOH)

di-*tert*-butyl ((3*S*,5*S*)-3-hydroxy-6-(((*S*)-3-methyl-1-oxobutan-2-yl)amino)-6-oxohexane-1,5-diyl)dicarbamate (S16c)

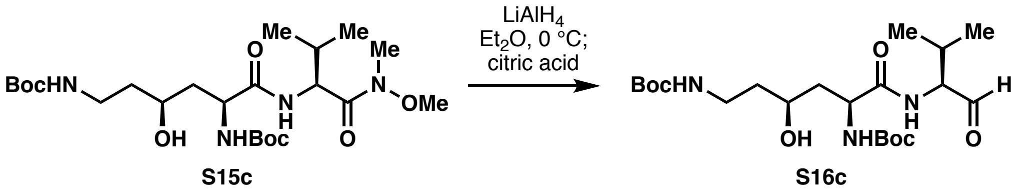

Following General Procedure B: **S15c** (600 mg, 1.19 mmol, 1 equiv) and LAH (169 mg, 4.46 mmol, 3.75 equiv) were used to provide **S16c** (519 mg) as a white solid that was directly subjected to the next reaction.

^{1}H NMR (400 MHz, CDCl$_3$): δ 9.61 (d, *J* = 4.0 Hz, 1H), 7.25 – 7.09 (m, 1H), 5.87 – 5.57 (m, 1H), 4.92 – 4.78 (m, 1H), 4.55 – 4.39 (m, 1H), 4.38 – 4.12 (m, 2H), 3.84 – 3.72 (m, 1H), 3.51 –

3.40 (m, 1H), 3.17 – 3.05 (m, 1H), 2.39 – 2.25 (m, 1H), 2.03 – 1.93 (m, 1H), 1.84 – 1.77 (m, 1H), 1.62 – 1.55 (m, 2H), 1.46 – 1.41 (m, 18H), 1.02 – 0.93 (m, 6H).

tert-butyl (_S_,_E_)-4-((2_S_,4_S_)-2,6-bis((_tert_-butoxycarbonyl)amino)-4-hydroxyhexanamido)-5-methylhex-2-enoate (S17d)

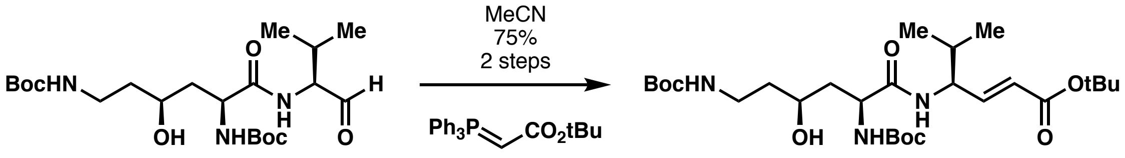

S16c **33** **S17d**

Following General Procedure C: **S16c** (519 mg) was used to provide **S17d** (487 mg, 75% over 2 steps) as a white solid.

^{1}H NMR (400 MHz, Methanol-d_4): δ 6.79 (dd, J = 15.7, 5.9 Hz, 1H), 5.86 (dd, J = 15.7, 1.6 Hz, 1H), 4.34 (t, J = 6.3 Hz, 1H), 4.18 (t, J = 7.4 Hz, 1H), 3.70 – 3.61 (m, 1H), 3.16 (dd, J = 7.7, 6.0 Hz, 2H), 1.96 – 1.82 (m, 2H), 1.74 – 1.63 (m, 2H), 1.61 – 1.51 (m, 1H), 1.50 – 1.40 (m, 27H), 0.97 – 0.90 (m, 6H).

^{13}C NMR (101 MHz, Methanol-d_4): δ 174.7, 167.2, 158.7, 157.7, 147.1, 124.4, 81.8, 80.7, 80.0, 67.6, 56.9, 54.2, 40.5, 38.4, 38.1, 33.4, 28.8, 28.8, 28.4, 19.6, 18.7.

HRMS (ESI): calculated for $C_{27}H_{50}N_3O_8^+$ ([M+H]$^+$) 544.3592, found 544.3591

$[\alpha]_D^{27}$ = –35.3 (c 0.5, MeOH)

(_S_,_E_)-4-((2_S_,4_S_)-2,6-diamino-4-hydroxyhexanamido)-5-methylhex-2-enoic acid (S18d)

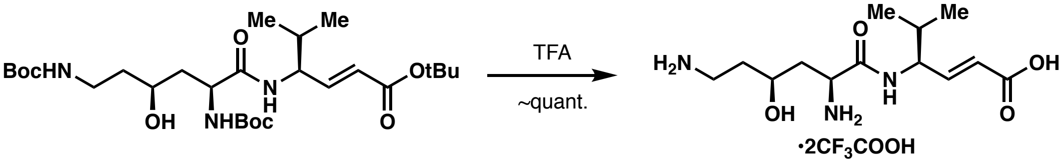

S17d **S18d**

Following General Procedure D: **S17d** (150 mg, 0.276 mmol, 1 equiv) was used to provide **S18d** (143 mg, quant.) as a white solid.

^{1}H NMR (400 MHz, Methanol-d_4): δ 6.81 (dd, J = 15.7, 6.6 Hz, 1H), 5.90 (dd, J = 15.7, 1.4 Hz, 1H), 4.44 – 4.30 (m, 1H), 4.08 (dd, J = 8.1, 5.2 Hz, 1H), 4.00 – 3.86 (m, 1H), 3.15 – 2.99 (m, 2H), 2.06 – 1.97 (m, 1H), 1.97 – 1.72 (m, 4H), 1.03 – 0.92 (m, 6H).

^{13}C NMR (101 MHz, Methanol-*d*$_4$): δ 169.9, 169.8, 146.9, 124.3, 68.4, 57.7, 53.6, 39.2, 37.8, 36.0, 33.2, 19.3, 19.0.

HRMS (ESI): calculated for $C_{13}H_{26}N_3O_4^+$ ($[M+H]^+$) 288.1918, found 288.1917

$[\alpha]_D^{27}$ = +2.6 (*c* 0.4, MeOH)

(5*S*,8*S*,10*S*,*E*)-8-amino-10-hydroxy-5-isopropyl-1,6-diazacyclododec-3-ene-2,7-dione (S19d)

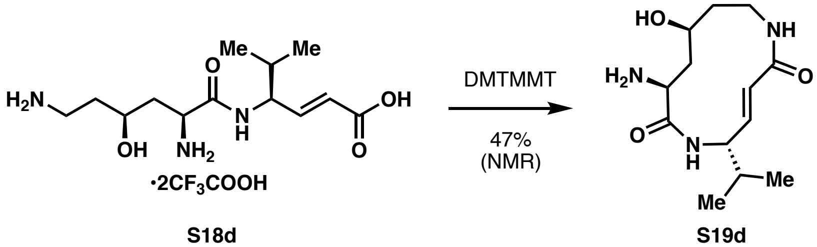

Following General Procedure E: **S18d** (131 mg, 0.254 mmol, 1 equiv) was used to provide **S19d** (138 mg, 47%). Yield was determined by ^{1}H NMR analysis of a 8.6 mg sample of crude **S19d** and 7.1 mg of 4-toluenesulfonamide added as internal standard.

^{1}H NMR (400 MHz, Methanol-*d*$_4$): 4-toluenesulfonamide: δ 7.78 (d, *J* = 8.2 Hz, 2H), 7.35 (d, *J* = 8.0 Hz, 2H), 2.41 (s, 3H). Compound **S19d**: δ 6.59 (dd, *J* = 16.1, 6.9 Hz, 1H), 6.40 (dd, *J* = 16.1, 10.6 Hz, 1H), 4.36 – 4.24 (m, 1H), 3.98 – 3.88 (m, 4H), 2.26 – 2.14 (m, 1H), 1.97 – 1.75 (m, 3H), 1.68 (s, 2H), 1.03 (dd, *J* = 13.4, 6.7 Hz, 6H).

HRMS (ESI): calculated for $C_{13}H_{24}N_3O_3^+$ ($[M+H]^+$) 270.1812, found 270.1815

(2*E*,4*E*)-*N*-((2*S*,3*R*)-3-hydroxy-1-(((5*S*,8*S*,10*S*,*E*)-10-hydroxy-5-isopropyl-2,7-dioxo-1,6-diazacyclododec-3-en-8-yl)amino)-1-oxobutan-2-yl)-11-methyldodeca-2,4-dienamide (43)

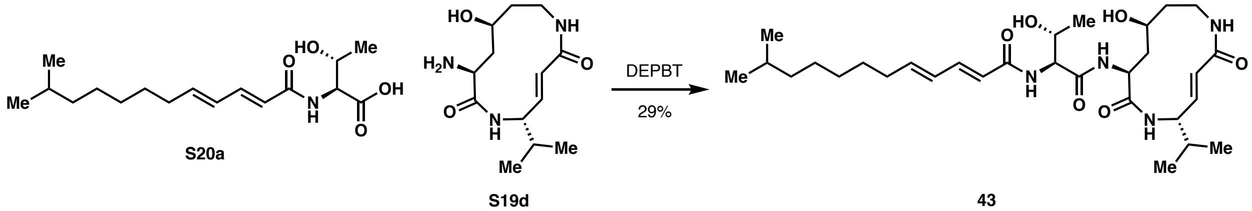

Compound **S20a** was synthesized by a previously reported procedure.[4] Following General Procedure F: **S19d** (60 mg, 0.0573 mmol as judged by ^{1}H NMR analysis, 1 equiv) and **S20a** (36 mg, 0.115 mmol, 2 equiv) were used to provide **43** (9.2 mg, 29%) as a fluffy white powder.

^{1}H NMR (600 MHz, DMSO-d_6): δ 8.48 (d, J = 8.6 Hz, 1H), 7.93 (d, J = 8.7 Hz, 1H), 7.78 (d, J = 7.6 Hz, 1H), 7.41 (t, J = 6.3 Hz, 1H), 6.99 (dd, J = 15.1, 10.8 Hz, 1H), 6.32 (dd, J = 15.8, 6.7 Hz, 1H), 6.24 – 6.01 (m, 4H), 4.85 (br s, 1H), 4.67 (br s, 1H), 4.37 (t, J = 9.9 Hz, 1H), 4.28 (dd, J = 8.8, 4.5 Hz, 1H), 4.05 (q, J = 8.0 Hz, 1H), 3.98 – 3.82 (m, 1H), 3.62 – 3.52 (m, 1H), 3.05 – 2.92 (m, 2H), 2.13 (q, J = 7.2 Hz, 2H), 1.86 (td, J = 12.5, 6.8 Hz, 1H), 1.81 – 1.69 (m, 1H), 1.57 (d, J = 13.3 Hz, 1H), 1.50 (hept, J = 6.6 Hz, 1H), 1.46 – 1.34 (m, 4H), 1.29 – 1.22 (m, 4H), 1.17 – 1.11 (m, 2H), 0.99 (d, J = 6.3 Hz, 3H), 0.91 (d, J = 6.6 Hz, 3H), 0.88 (d, J = 6.7 Hz, 3H), 0.84 (d, J = 6.6 Hz, 6H).

^{13}C NMR (151 MHz, DMSO-d_6): δ 171.5, 169.4, 167.8, 165.4, 142.0, 140.8, 139.7, 128.6, 124.8, 123.1, 66.9, 66.8, 58.2, 55.6, 51.2, 42.2, 38.4, 32.2, 30.6, 28.8, 28.4, 27.4, 26.6, 22.5, 19.8, 19.6, 19.1.

HRMS (ESI): calculated for $C_{30}H_{51}N_4O_6^+$ ($[M+H]^+$) 563.3803, found 563.3804

$[\alpha]_D^{27} = -135.3$ (c 0.2, MeOH)

Synthesis of desoxy-Thr cepafungin (**44**)

Scheme S8. Synthetic route to desoxy-Thr cepafungin **44**.

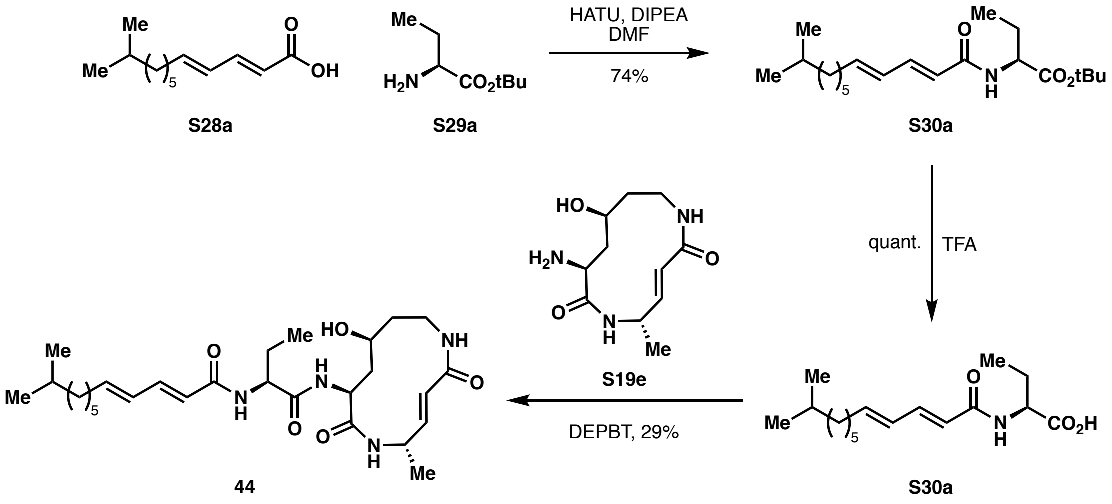

***tert*-butyl (*S*)-2-((2*E*,4*E*)-11-methyldodeca-2,4-dienamido)butanoate (S30a)**

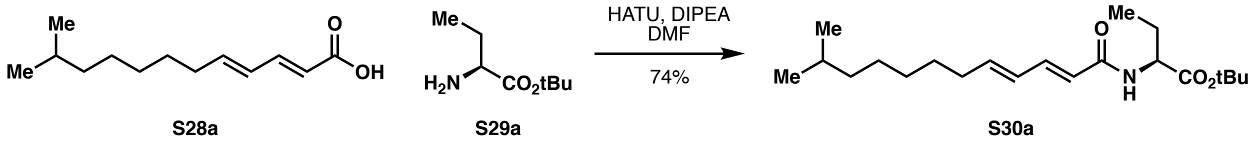

Compound **S28a** was synthesized as described previously.[4] Following General Procedure K: **S28a** (500 mg, 2.38 mmol, 1 equiv), **S29a** (698 mg, 3.57 mmol, 1.5 equiv), HATU (949 mg, 2.50 mmol, 1.05 equiv) and DIPEA (1.24 mL, 7.13 mmol, 3 equiv) were used to provide **S30a** as a colorless resin.

^{1}H NMR (400 MHz, $CDCl_3$): δ 7.18 (dd, *J* = 15.0, 10.1 Hz, 1H), 6.21 – 5.99 (m, 3H), 5.79 (d, *J* = 15.1 Hz, 1H), 4.57 (dt, *J* = 8.0, 5.8 Hz, 1H), 2.14 (q, *J* = 7.0 Hz, 2H), 1.90 (dqd, *J* = 15.1, 7.5, 5.4 Hz, 1H), 1.79 – 1.69 (m, 1H), 1.55 – 1.48 (m, 1H), 1.47 (s, 9H), 1.40 (q, *J* = 6.8 Hz, 2H), 1.30 – 1.23 (m, 4H), 1.18 – 1.11 (m, 2H), 0.89 (t, *J* = 7.4 Hz, 3H), 0.85 (d, *J* = 6.6 Hz, 6H).

^{13}C NMR (101 MHz, $CDCl_3$): δ 172.0, 165.9, 143.7, 141.9, 128.3, 121.5, 82.2, 53.7, 39.0, 33.1, 29.5, 28.9, 28.2, 28.1, 27.3, 26.0, 22.8, 9.3.

HRMS (ESI): calculated for $C_{21}H_{38}NO_3^+$ ($[M+H]^+$) 352.2846, found 352.2845

$[\alpha]_D^{27}$ = –40.6 (*c* 0.6, MeOH)

(*S*)-2-((2*E*,4*E*)-11-methyldodeca-2,4-dienamido)butanoic acid (S31a)

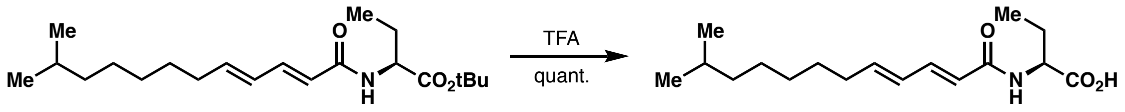

Following General Procedure L: **S30a** (60 mg, 0.171 mmol, 1 equiv) was used to provide **S31a** (57 mg, quant.) as a clear, colorless resin.

^{1}H NMR (400 MHz, $CDCl_3$): δ 7.25 – 7.18 (m, 1H), 6.37 (br s, 1H), 6.21 (d, *J* = 7.5 Hz, 1H), 6.17 – 6.06 (m, 2H), 5.82 (d, *J* = 15.0 Hz, 1H), 4.63 (td, *J* = 7.1, 5.5 Hz, 1H), 2.15 (q, *J* = 7.0 Hz, 2H), 2.06 – 1.94 (m, 1H), 1.88 – 1.75 (m, 1H), 1.57 – 1.45 (m, 1H), 1.41 (p, *J* = 7.0 Hz, 2H), 1.30 – 1.24 (m, 4H), 1.18 – 1.11 (m, 2H), 0.97 (t, *J* = 7.5 Hz, 3H), 0.86 (d, *J* = 6.6 Hz, 6H).

^{13}C NMR (101 MHz, $CDCl_3$): δ 175.3, 167.3, 145.0, 143.3, 128.2, 120.4, 53.9, 39.0, 33.2, 29.6, 28.9, 28.1, 27.3, 25.3, 22.8, 9.7.

HRMS (ESI): calculated for $C_{17}H_{30}NO_3^+$ ($[M+H]^+$) 296.2220, found 296.2225

$[\alpha]_D^{26}$ = –15.7 (*c* 0.4, MeOH)

(2*E*,4*E*)-*N*-((*S*)-1-(((5*S*,8*S*,10*S*,*E*)-10-hydroxy-5-methyl-2,7-dioxo-1,6-diazacyclododec-3-en-8-yl)amino)-1-oxobutan-2-yl)-11-methyldodeca-2,4-dienamide (44)

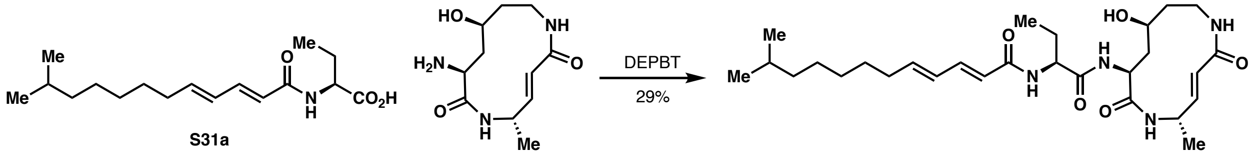

Compound **S19e** was synthesized as previously described.[4] Following General Procedure F: **S31a** (50 mg, 0.169 mmol, 2 equiv) and **S19e** (102 mg, 0.847 mmol as judged by ^{1}H NMR analysis, 1 equiv) were used to provide **44** (12.8 mg, 29%) as a fluffy white powder.

^{1}H NMR (600 MHz, DMSO-*d*$_6$): δ 8.80 – 8.40 (m, 1H), 8.03 (d, *J* = 8.2 Hz, 1H), 7.96 (d, *J* = 7.7 Hz, 1H), 7.41 (t, *J* = 6.2 Hz, 1H), 6.98 (dd, *J* = 15.1, 10.8 Hz, 1H), 6.40 (d, *J* = 14.1 Hz, 1H), 6.18 (dt, *J* = 15.2, 5.0 Hz, 2H), 6.11 – 6.00 (m, 2H), 4.82 – 4.51 (m, 1H), 4.38 (s, 1H), 4.34 – 4.20 (m, 2H), 3.56 (s, 1H), 3.07 – 2.94 (m, 2H), 2.12 (q, *J* = 7.2 Hz, 2H), 1.97 – 1.77 (m, 1H), 1.67 – 1.57 (m, 1H), 1.57 – 1.42 (m, 4H), 1.41 – 1.34 (m, 3H), 1.31 – 1.22 (m, 5H), 1.22 – 1.18 (m, 2H), 1.14 (q, *J* = 6.9 Hz, 2H), 0.84 (d, *J* = 6.6 Hz, 6H), 0.81 (t, *J* = 7.4 Hz, 3H).

^{13}C NMR (151 MHz, DMSO-*d*$_6$): δ 171.1, 170.8, 167.6, 165.1, 143.2, 142.0, 139.6, 128.6, 123.2, 123.0, 67.0, 53.6, 51.2, 44.8, 42.4, 38.4, 32.2, 28.8, 28.4, 27.4, 26.6, 25.5, 22.5, 18.6, 10.2.

HRMS (ESI): calculated for $C_{28}H_{47}N_4O_5^+$ ([M+H]$^+$) 519.3541, found 519.3544

$[\alpha]_D^{27}$ = –125.7 (*c* 0.2, MeOH)

Synthesis of β-OH-Phe cepafungin (**45**)

Scheme S9. Synthetic route to β-OH-Phe cepafungin (**45**).

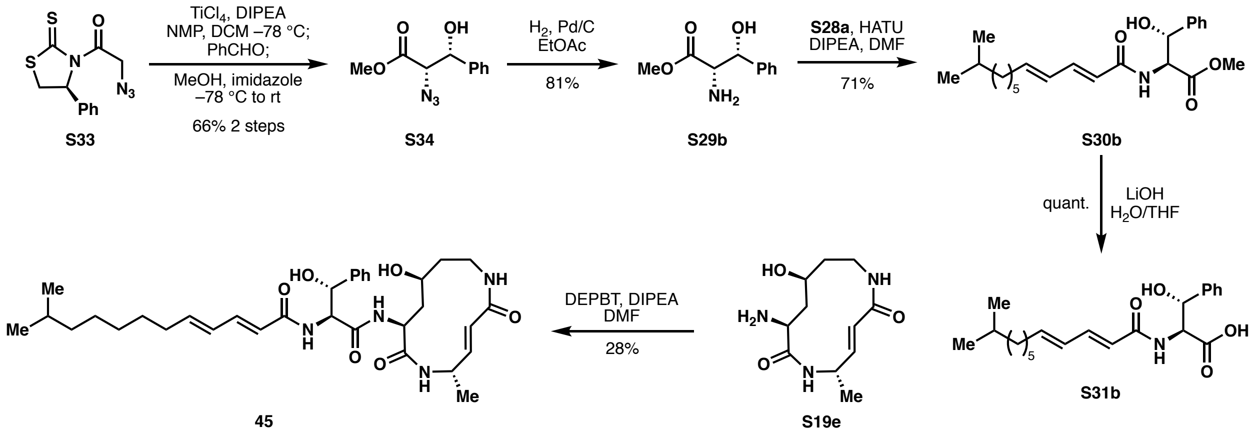

methyl (2*S*,3*R*)-2-azido-3-hydroxy-3-phenylpropanoate (S34)

Compound **S33** was prepared from (*S*)-2-amino-2-phenylethan-1-ol following previously reported procedures.[19] Following a procedure adapted from Patel et al.,[20] in a flame-dried 100 mL round bottom flask **S33** (1.00 g, 3.59 mmol, 1 equiv) was dissolved in 36 mL anhydrous DCM and cooled to –78 °C. Neat $TiCl_4$ (414 µL, 3.77 mmol, 1.05 equiv) was added dropwise, followed by DIPEA (688 µL, 3.95 mmol, 1.1 equiv). The mixture was stirred for 1 h under argon at –78 °C. Then, N-methylpyrrolidone (693 µL, 7.19 mmol, 2 equiv) was added and stirred a further 15 min. A solution of benzaldehyde (548 µL, 5.39 mmol, 1.5 equiv) in 2 mL anhydrous DCM was added dropwise, and the reaction was stirred under argon at –78 °C for 6 h or until complete consumption of **S33** as judged by TLC. At –78 °C, a solution of imidazole (4.89 g, 71.9 mmol, 20 equiv) dissolved in 31 mL anhydrous MeOH was slowly added and stirred to ~35 °C over 1.5 h. The reaction was then quenched with 50 mL H_2O and the organics were removed in vacuo. The aqueous phase was diluted with 100 mL H_2O and extracted with 5 x 30 mL DCM. The combined organic layers were washed with 30 mL 1M HCl, H_2O, $NaHCO_3$, and brine, dried over Na_2SO_4, filtered and concentrated. The residue was precipitated into 45 mL cyclohexane from 5 mL DCM, and the solids were collected by centrifugation (4 °C, 10 min, 4200 rpm). The supernatant was concentrated and purified by flash chromatography (100% DCM) to provide **S34** (527 mg, 66% over 2 steps) as an oil.

^{1}H NMR (400 MHz, $CDCl_3$): δ 7.42 – 7.31 (m, 5H), 5.21 (d, J = 4.3 Hz, 1H), 4.05 (d, J = 4.3 Hz, 1H), 3.77 (s, 3H), 2.67 (s, 1H).

^{13}C NMR (101 MHz, CDCl$_3$): δ 169.17, 139.27, 128.75, 128.66, 126.23, 74.56, 67.78, 52.98.
HRMS (ESI): calculated for $C_{10}H_{11}N_3NaO_3^+$ ([M+Na]$^+$) 244.0693, found 244.0692
$[\alpha]_D^{27}$ = –77.2 (*c* 2.4, MeOH)

methyl (2*S*,3*R*)-2-amino-3-hydroxy-3-phenylpropanoate (S29b)

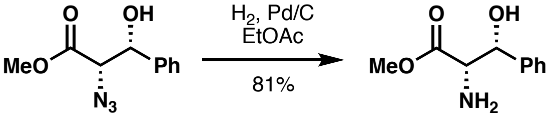

S34 S29b

In a 25 mL reaction vial, azide **S34** (200 mg, 0.904 mmol, 1 equiv) was dissolved in 6 mL EtOAc, then 10% Pd/C (40 mg, 20 wt % relative to **S34**) was added. The mixture was sparged with argon for 25 min, then with H_2 for 15 min, then stirred under an atmosphere of H_2 overnight. An additional 10 wt % Pd/C were added twice over the course of 2 days. At 72 h, the reaction was filtered through a plug of celite then purified by flash chromatography (95:5 DCM:MeOH) to provide **S29b** (118 mg) as a colorless oil.
^{1}H NMR (400 MHz, CDCl$_3$): δ 7.38 – 7.27 (m, 5H), 4.97 (d, *J* = 5.4 Hz, 1H), 3.86 – 3.81 (m, 1H), 3.61 (s, 3H), 3.01 (br s, 2H), 1.25 (d, *J* = 1.9 Hz, 1H).
^{13}C NMR (101 MHz, CDCl$_3$): δ 172.4, 140.4, 128.6, 128.2, 126.3, 73.8, 60.5, 52.6.
HRMS (ESI): calculated for $C_{10}H_{14}NO_3^+$ ([M+H]$^+$) 196.0968, found 196.0969
$[\alpha]_D^{27}$ = +12.8 (*c* 0.3, MeOH)

methyl (2*S*,3*R*)-3-hydroxy-2-((2*E*,4*E*)-11-methyldodeca-2,4-dienamido)-3-phenylpropanoate (S30b)

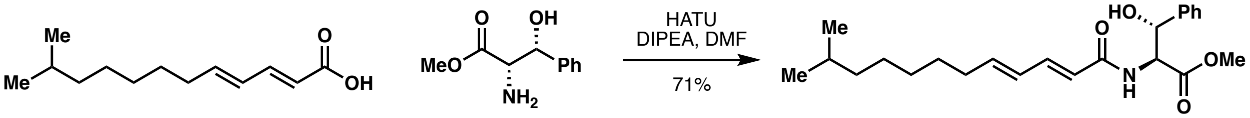

S28a S29b S30b

Following General Procedure K: **S28a** (95 mg, 0.451 mmol, 1 equiv), **S29b** (80 mg, 0.496 mmol, 1.1 equiv), HATU (180 mg, 0.474 mmol, 1.05 equiv) and DIPEA (94 µL, 0.541 mmol, 1.2 equiv) were used to provide **S30b** (124 mg, 71%) as a clear, colorless resin.

^{1}H NMR (400 MHz, CDCl$_3$): δ 7.38 – 7.26 (m, 5H), 7.12 (dd, *J* = 15.0, 9.8 Hz, 1H), 6.24 (d, *J* = 8.6 Hz, 1H), 6.15 – 5.99 (m, 2H), 5.75 (d, *J* = 15.0 Hz, 1H), 5.28 (d, *J* = 3.4 Hz, 1H), 4.95 (dd, *J* = 8.5, 3.5 Hz, 1H), 3.72 (s, 3H), 2.13 (q, *J* = 6.7 Hz, 2H), 1.57 – 1.45 (m, 1H), 1.46 – 1.34 (m, 2H), 1.30 – 1.22 (m, 4H), 1.19 – 1.09 (m, 2H), 0.86 (d, *J* = 6.7 Hz, 6H).

^{13}C NMR (101 MHz, CDCl$_3$): δ 171.2, 166.7, 144.4, 142.8, 139.7, 128.6, 128.3, 128.2, 126.0, 120.6, 74.2, 58.4, 52.8, 39.0, 33.1, 29.6, 28.9, 28.1, 27.3, 22.8.

HRMS (ESI): calculated for $C_{23}H_{34}NO_4^+$ ($[M+H]^+$) 388.2482, found 388.2474

$[\alpha]_D^{27}$ = –50.9 (*c* 0.6, MeOH)

(2*S*,3*R*)-3-hydroxy-2-((2*E*,4*E*)-11-methyldodeca-2,4-dienamido)-3-phenylpropanoic acid (S31b)

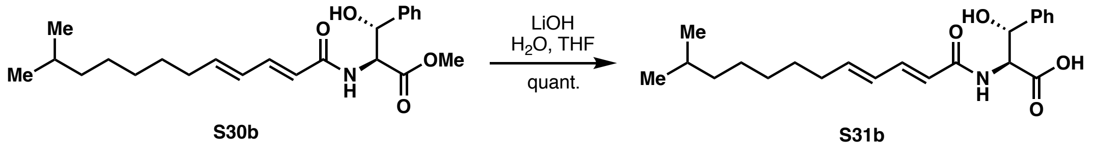

Following General Procedure J: **S30b** (88 mg, 0.227 mmol, 1 equiv), LiOH (11 mg, 0.454 mmol, 2 equiv) and 22 mL of 1:1 THF:H_2O reaction solvent were used to provide **S31b** (95 mg, quant.) as a clear, colorless resin.

^{1}H NMR (400 MHz, CDCl$_3$): δ 7.37 – 7.26 (m, 5H), 7.14 – 7.02 (m, 1H), 6.61 (d, *J* = 7.8 Hz, 1H), 6.13 – 5.96 (m, 2H), 5.72 (d, *J* = 15.0 Hz, 1H), 5.46 (d, *J* = 2.9 Hz, 1H), 4.91 (dd, *J* = 7.7, 2.9 Hz, 1H), 2.18 – 2.08 (m, 2H), 1.56 – 1.45 (m, 1H), 1.38 (p, *J* = 6.6 Hz, 2H), 1.29 – 1.22 (m, 5H), 1.18 – 1.11 (m, 2H), 0.86 (d, *J* = 6.6 Hz, 6H).

^{13}C NMR (101 MHz, CDCl$_3$): δ 173.0, 168.4, 145.3, 143.8, 139.0, 128.7, 128.2, 128.1, 125.9, 119.9, 72.2, 58.5, 39.0, 33.2, 29.6, 28.9, 28.1, 27.3, 22.8.

HRMS (ESI): calculated for $C_{22}H_{32}NO_4^+$ ($[M+H]^+$) 374.2326, found 374.2327

$[\alpha]_D^{27}$ = –29.2 (*c* 0.4, MeOH)

(2*E*,4*E*)-*N*-((1*R*,2*S*)-1-hydroxy-3-(((5*S*,8*S*,10*S*,*E*)-10-hydroxy-5-methyl-2,7-dioxo-1,6-diazacyclododec-3-en-8-yl)amino)-3-oxo-1-phenylpropan-2-yl)-11-methyldodeca-2,4-dienamide (45)

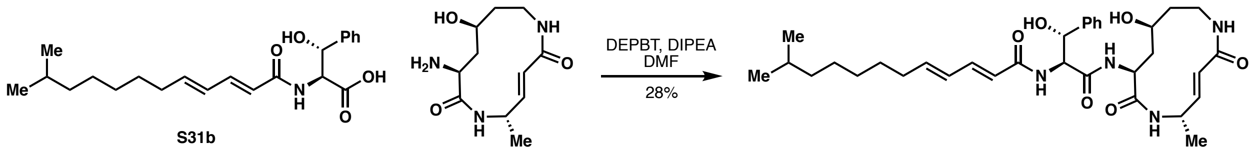

S31b

S19e

Compound **S19e** was synthesized as previously described.[4] Following General Procedure F: **S31b** (43 mg, 0.116 mmol, 2 equiv) and **S19e** (70 mg, 0.0580 mmol as judged by ^{1}H NMR analysis, 1 equiv) were used to provide **45** (9.7 mg, 28%) as a fluffy white powder.

^{1}H NMR (600 MHz, DMSO-*d*$_6$): δ 8.87 – 8.42 (m, 1H), 8.01 (d, *J* = 9.2 Hz, 1H), 7.71 (d, *J* = 8.3 Hz, 1H), 7.42 (t, *J* = 6.4 Hz, 1H), 7.35 (d, *J* = 7.4 Hz, 2H), 7.25 (t, *J* = 7.6 Hz, 2H), 7.20 – 7.15 (m, 1H), 6.84 (dd, *J* = 15.2, 10.8 Hz, 1H), 6.42 (d, *J* = 15.7 Hz, 1H), 6.19 (d, *J* = 15.8 Hz, 1H), 6.16 – 5.99 (m, 3H), 5.72 (d, *J* = 4.7 Hz, 1H), 5.09 (t, *J* = 3.6 Hz, 1H), 4.70 (s, 1H), 4.55 (dd, *J* = 9.3, 3.0 Hz, 1H), 4.41 (br s, 2H), 3.57 (s, 1H), 3.09 – 2.93 (m, 2H), 2.10 (q, *J* = 7.2 Hz, 2H), 1.86 (br s, 1H), 1.72 – 1.55 (m, 1H), 1.54 – 1.41 (m, 2H), 1.41 – 1.32 (m, 3H), 1.28 – 1.18 (m, 7H), 1.16 – 1.10 (m, 2H), 0.84 (d, *J* = 6.6 Hz, 6H).

^{13}C NMR (151 MHz, DMSO-*d*$_6$): δ 170.8, 168.9, 167.7, 165.3, 143.0, 142.5, 142.1, 139.7, 128.5, 127.6, 126.9, 126.2, 123.3, 122.9, 72.3, 67.2, 58.7, 51.2, 44.8, 42.9, 38.4, 32.2, 28.8, 28.4, 27.4, 26.6, 22.5, 18.5.

HRMS (ESI): calculated for $C_{33}H_{49}N_4O_6^+$ ([M+H]$^+$) 597.3647, found 597.3639

$[\alpha]_D^{27}$ = –94.6 (*c* 0.1, MeOH)

Synthesis of β-OH-Leu cepafungin **46**

Scheme S10. Synthetic route to β-OH-Leu cepafungin **46**.

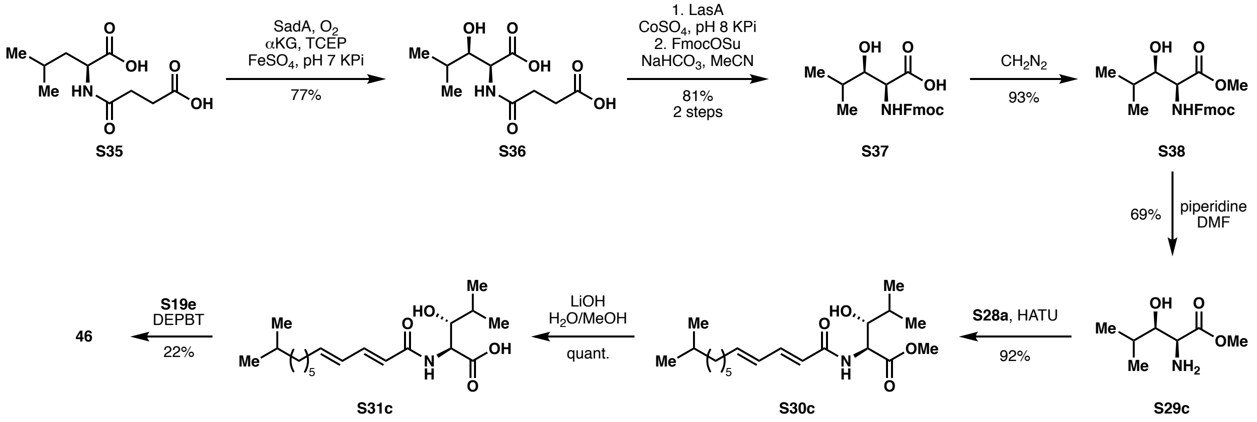

(2*S*,3*R*)-2-(3-carboxypropanamido)-3-hydroxy-4-methylpentanoic acid (S36)

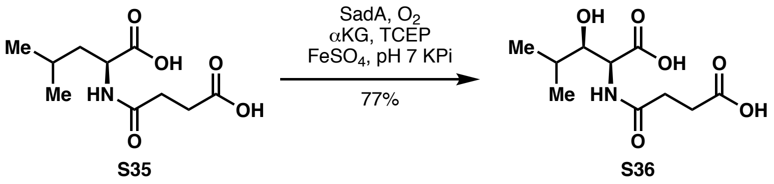

Compound **S35** was prepared from L-leucine as described in the literature.[21] Following a procedure adapted from Hibi et al.,[22] a glycerol stock of *E. coli* BL21(DE3) cells harboring pET-28a(+)-SadA plasmid was used to inoculate an overnight culture of LB media (6 mL) containing 50 μg/mL kanamycin. 1.0 mL of this culture was used to inoculate each of 4 x 200 mL TB media containing 50 μg/mL kanamycin in a 1 L non-beveled Erlenmeyer flask. The cultures were shaken at 250 rpm/37 °C for 2.5 h or until an OD_{600} = 0.6 was reached. The culture was cooled on ice (15 min), induced by adding IPTG to final concentration of 25 μM, then allowed to continue shaking at 250 rpm/23 °C for another 21 h. Cells were harvested by centrifugation (4 °C, 15 min, 4200 rpm), then resuspended in 50 mM pH = 7 KPi buffer (ca. 45 mL per 200 mL culture) to a final OD_{600} = 30. The cell suspension was lysed in 45 mL batches by sonication at 50% amplitude for 5 min (1 s on, 4 s off) in an ice bath. The cell debris was pelleted by centrifugation (4 °C, 15 min, 4200 rpm), and the clarified lysate supernatant was diluted 1:1 with 50 mM pH = 7 KPi and added to a non-beveled Erlenmeyer flask (≥80% headspace) containing **S35** (833 mg, 3.6 mmol, 1 equiv, 10 mM final concentration), α-ketoglutaric acid (1.22 g, 5.4 mmol, 1.5 equiv), TCEP (206 mg,

0.720 mmol, 0.2 equiv) and $FeSO_4•7H_2O$ (200 mg, 0.720 mmol, 0.2 equiv). The reaction mixture was then shaken at 250 rpm/30 °C in an open Erlenmeyer flask overnight or until completion by LCMS. The reaction was then quenched by addition of 6 M HCl to final pH = 2 and centrifuged (4 °C, 15 min, 4200 RPM). The supernatant was concentrated in vacuo to a final volume of ~25 mL and centrifuged. The resulting pellet was resuspended in ~5 mL H_2O, centrifuged, and the supernatants were combined and purified by C18 flash chromatography (gradient 0–50% MeOH in H_2O over 10 column volumes, then 3 column volumes to 100% MeOH) to provide **S36** (681 mg, 77%) as an off-white solid after lyophilization.

^{1}H NMR (400 MHz, DMSO-*d*$_6$): δ 12.24 (br s, 2H), 7.76 (d, *J* = 9.1 Hz, 1H), 4.88 (br s, 1H), 4.44 (dd, *J* = 9.1, 2.7 Hz, 1H), 3.51 (dd, *J* = 8.9, 2.6 Hz, 1H), 2.48 – 2.31 (m, 4H), 1.64 – 1.49 (m, 1H), 0.91 (d, *J* = 6.6 Hz, 3H), 0.76 (d, *J* = 6.7 Hz, 3H).

^{13}C NMR (101 MHz, DMSO-*d*$_6$): δ 173.8, 172.9, 171.4, 76.2, 54.4, 30.7, 29.9, 29.2, 19.2, 18.9.

HRMS (ESI): calculated for $C_{10}H_{18}NO_6^+$ ($[M+H]^+$) 248.1129, found 248.1136

$[\alpha]_D^{27}$ = +20.4 (*c* 1.6, MeOH)

(2*S*,3*R*)-2-(((9*H*-fluoren-9-yl)methoxy)carbonyl)amino)-3-hydroxy-4-methylpentanoic acid (S37)

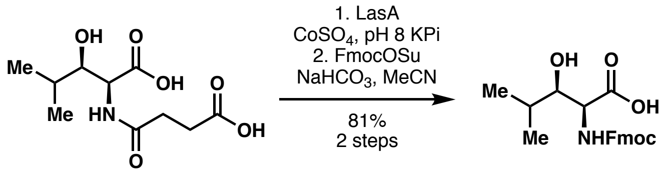

S36 S37

Following a procedure adapted from Hibi et al.,[22] a glycerol stock of *E. coli* BL21(DE3) cells harboring pET-28a(+)-LasA plasmid was used to inoculate an overnight culture of LB media (4 mL) containing 50 µg/mL kanamycin. 0.5 mL of this culture was used to inoculate each of 2 x 100 mL TB media containing 50 µg/mL kanamycin in 2 x 500 mL non-beveled Erlenmeyer flasks. The cultures were shaken at 250 rpm/37 °C for 2.5 h or until an OD_{600} = 0.6 was reached. The culture was cooled on ice (15 min), induced by adding IPTG to final concentration of 25 µM, then allowed to continue shaking at 250 rpm/23 °C for another 20 h. Cells were harvested by centrifugation (4 °C, 15 min, 4200 rpm), then resuspended in 50 mM pH = 8 KPi buffer (ca. 50 mL per 100 mL culture) to a final OD_{600} = 20. The combined cell suspensions were lysed by sonication at 50%

amplitude for 2 x 3 min (1 s on, 4 s off) in an ice bath. The cell debris was pelleted by centrifugation (4 °C, 15 min, 4200 rpm), and the clarified lysate supernatant was added to a non-beveled 500 mL Erlenmeyer flask containing **S36** (247 mg, 1 mmol, 1 equiv, 10 mM final concentration) and $CoSO_4$•$7H_2O$ (2.81 mg, 0.0100 mmol, 0.01 equiv). The mixture was shaken at 200 rpm/20 °C overnight or until completion by LCMS. The reaction was then directly treated with 10 mL saturated $NaHCO_3$ solution and FmocOSu (1.35 g, 4 mmol, 4 equiv) dissolved in 30 mL MeCN and stirred at rt for 4 h or until completion by LCMS. The mixture was centrifuged (4 °C, 15 min, 4200 rpm) and the supernatant was concentrated in vacuo to remove MeCN. The aqueous phase was adjusted to pH = 1 with 6 M HCl and extracted with 4 x 50 mL EtOAc. The combined organic layers were washed with 1:1 brine:1 M HCl mixture and dried over Na_2SO_4, filtered and concentrated. The residue was purified by flash chromatography (95:5:1 DCM:MeOH:AcOH) and evaporated twice from toluene to provide **S37** (294 mg, 81% over 2 steps) as a white solid.

^{1}H NMR (400 MHz, Methanol-*d*$_4$): δ 7.80 (d, *J* = 7.6 Hz, 2H), 7.70 – 7.65 (m, 2H), 7.44 – 7.25 (m, 5H), 4.44 – 4.36 (m, 3H), 4.24 (t, *J* = 7.0 Hz, 1H), 3.68 (dd, *J* = 9.3, 2.4 Hz, 1H), 1.73 – 1.62 (m, 1H), 1.02 (d, *J* = 6.6 Hz, 3H), 0.90 (d, *J* = 6.7 Hz, 3H).

^{13}C NMR (151 MHz, Methanol-*d*$_4$): δ 174.90, 158.81, 145.37, 145.06, 142.60, 142.58, 128.78, 128.77, 128.17, 128.14, 126.26, 126.24, 120.92, 120.90, 78.37, 68.00, 48.42, 32.36, 26.26, 19.62, 19.48.

HRMS (ESI): calculated for $C_{21}H_{24}NO_5^+$ ($[M+H]^+$) 370.1649, found 370.1646

$[\alpha]_D^{24}$ = +2.4 (*c* 0.5, MeOH)

methyl (2*S*,3*R*)-2-((((9*H*-fluoren-9-yl)methoxy)carbonyl)amino)-3-hydroxy-4-methylpentanoate (S38)

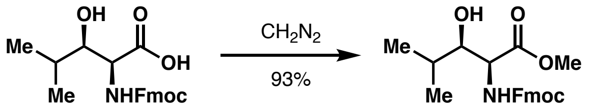

S37 **S38**

Diazomethane was prepared from *N*-methyl-*N*-nitrosourea as described in the literature.[23] Compound **S37** (50 mg, 0.135 mmol, 1 equiv) was dissolved in 1.4 mL of a 1.5:1 mixture of Et_2O:MeOH and cooled to 0 °C. A solution of diazomethane (ca. 10 eq) was added dropwise, and

the reaction was stirred at 0 °C for 20 min or until completion by TLC. The reaction was quenched at 0 °C with 0.5 mL AcOH, diluted with 30 mL Et_2O, washed with 2 x 50 mL $NaHCO_3$, then brine and dried over Na_2SO_4, filtered and concentrated. The residue was purified by flash chromatography (70:30 Hexanes:EtOAc) to provide **S38** (48 mg, 93%) as a colorless oil.

^{1}H NMR (400 MHz, Methanol-*d*$_4$): δ 7.82 – 7.75 (m, 2H), 7.70 – 7.62 (m, 2H), 7.45 – 7.22 (m, 5H), 4.45 (d, *J* = 2.5 Hz, 1H), 4.39 (d, *J* = 6.7 Hz, 2H), 4.22 (t, *J* = 6.9 Hz, 1H), 3.74 (s, 3H), 3.64 (dd, *J* = 9.2, 2.5 Hz, 1H), 1.73 – 1.60 (m, 1H), 1.01 (d, *J* = 6.6 Hz, 3H), 0.88 (d, *J* = 6.7 Hz, 3H).

^{13}C NMR (101 MHz, Methanol-*d*$_4$): δ 173.62, 158.78, 145.30, 145.03, 142.61, 142.59, 128.77, 128.16, 128.13, 126.22, 126.19, 120.93, 120.91, 78.17, 68.00, 58.12, 52.81, 48.41, 32.24, 19.56, 19.38.

HRMS (ESI): calculated for $C_{22}H_{26}NO_5^+$ ($[M+H]^+$) 384.1805, found 384.1807

$[\alpha]_D^{27}$ = –3.7 (*c* 0.4, MeOH)

methyl (2*S*,3*R*)-2-amino-3-hydroxy-4-methylpentanoate (S29c)

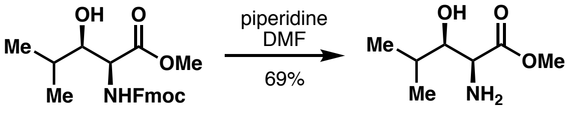

S38 S29c

A flame-dried 25 mL reaction vial was charged with **S38** (123 mg, 0.321 mmol, 1 equiv) dissolved 6.4 mL anhydrous DMF and cooled to 0 °C. Piperidine (1.6 mL, 20% v/v) was added, and the mixture was stirred at 0 °C for 1 hour or until completion by TLC. The mixture was diluted with 10 mL toluene and concentrated to dryness to provide **S29c** (36 mg, 69%) as a colorless liquid.

^{1}H NMR (400 MHz, Methanol-*d*$_4$): δ 3.73 (s, 3H), 3.57 (d, *J* = 3.1 Hz, 1H), 3.48 (dd, *J* = 8.6, 3.1 Hz, 1H), 1.79 (dhept, *J* = 8.6, 6.7 Hz, 1H), 1.01 (d, *J* = 6.6 Hz, 3H), 0.93 (d, *J* = 6.7 Hz, 3H).

^{13}C NMR (101 MHz, Methanol-*d*$_4$): δ 176.3, 79.0, 57.6, 52.5, 31.7, 19.7, 19.3.

HRMS (ESI): calculated for $C_7H_{16}NO_3^+$ ($[M+H]^+$) 162.1125, found 162.1127

$[\alpha]_D^{27}$ = +9.4 (*c* 0.5, MeOH)

methyl (2*S*,3*R*)-3-hydroxy-4-methyl-2-((2*E*,4*E*)-11-methyldodeca-2,4-dienamido)pentanoate (S30c)

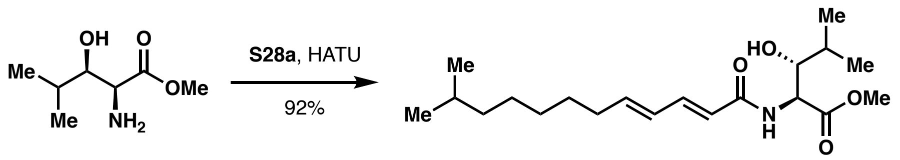

Following General Procedure K: **S29c** (29 mg, 0.180 mmol, 1 equiv), **S28a** (38 mg, 0.180 mmol, 1 equiv), HATU (68 mg, 0.180 mmol, 1 equiv) and DIPEA (31 µL, 0.180 mmol, 1 equiv) were used to provide **S30c** (59 mg, 92%) as a colorless oil.

^{1}H NMR (600 MHz, Methanol-*d*$_4$): δ 7.16 (dd, *J* = 15.1, 10.8 Hz, 1H), 6.27 – 6.21 (m, 1H), 6.14 (dt, *J* = 15.0, 6.9 Hz, 1H), 6.06 (d, *J* = 15.2 Hz, 1H), 4.81 – 4.77 (m, 1H), 3.74 (s, 3H), 3.69 (dd, *J* = 9.0, 2.5 Hz, 1H), 2.21 – 2.16 (m, 2H), 1.68 – 1.59 (m, 1H), 1.57 – 1.50 (m, 1H), 1.45 (p, *J* = 7.2 Hz, 2H), 1.34 – 1.30 (m, 4H), 1.21 – 1.17 (m, 2H), 1.01 (d, *J* = 6.6 Hz, 3H), 0.91 – 0.88 (m, 9H).

^{13}C NMR (151 MHz, Methanol-*d*$_4$): δ 173.3, 169.3, 144.8, 143.2, 129.8, 122.4, 78.1, 56.4, 52.8, 40.1, 34.0, 32.4, 30.6, 30.0, 29.1, 28.3, 23.0, 19.5, 19.4.

HRMS (ESI): calculated for $C_{20}H_{36}NO_4^+$ ([M+H]$^+$) 354.2639, found 354.2635

$[\alpha]_D^{27}$ = +10.9 (*c* 0.2, MeOH)

(2*S*,3*R*)-3-hydroxy-4-methyl-2-((2*E*,4*E*)-11-methyldodeca-2,4-dienamido)pentanoic acid (S31c)

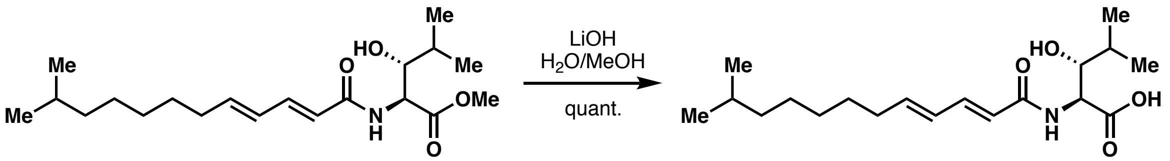

Following General Procedure J: **S30c** (42 mg, 0.1189 mmol, 1 equiv) was used to provide **S31c** (43 mg, quant.) as a colorless resin.

^{1}H NMR (600 MHz, Methanol-*d*$_4$): δ 7.16 (dd, *J* = 15.1, 10.8 Hz, 1H), 6.24 (dd, *J* = 15.1, 10.8 Hz, 1H), 6.16 – 6.10 (m, 1H), 6.06 (d, *J* = 15.1 Hz, 1H), 4.76 (d, *J* = 2.4 Hz, 1H), 3.73 (dd, *J* = 9.1, 2.4 Hz, 1H), 2.21 – 2.16 (m, 2H), 1.69 – 1.60 (m, 1H), 1.57 – 1.49 (m, 1H), 1.45 (p, *J* = 7.3 Hz, 2H), 1.35 – 1.30 (m, 4H), 1.21 – 1.16 (m, 2H), 1.02 (d, *J* = 6.6 Hz, 3H), 0.91 (d, *J* = 6.7 Hz, 3H), 0.88 (d, *J* = 6.6 Hz, 6H).

^{13}C NMR (151 MHz, Methanol-*d*$_4$): δ 174.6, 169.2, 144.6, 143.1, 129.8, 122.5, 78.2, 56.1, 40.1, 34.0, 32.5, 30.6, 30.0, 29.1, 28.3, 23.0, 19.5, 19.4.

HRMS (ESI): calculated for $C_{19}H_{34}NO_4^+$ ($[M+H]^+$) 340.2482, found 340.2478

$[\alpha]_D^{27}$ = +28.9 (*c* 0.2, MeOH)

(2*E*,4*E*)-*N*-((2*S*,3*R*)-3-hydroxy-1-(((5*S*,8*S*,10*S*,*E*)-10-hydroxy-5-methyl-2,7-dioxo-1,6-diazacyclododec-3-en-8-yl)amino)-4-methyl-1-oxopentan-2-yl)-11-methyldodeca-2,4-dienamide (46)

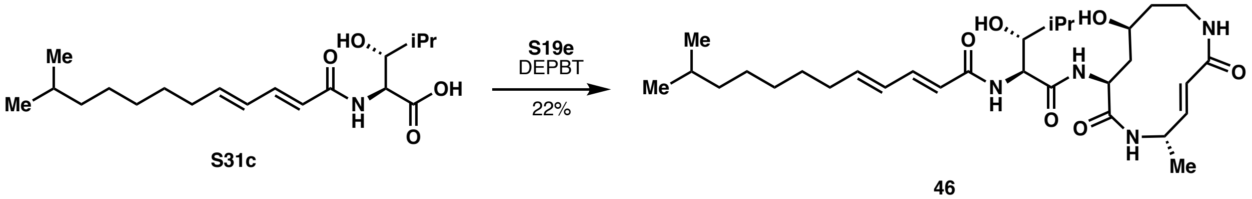

Following General Procedure F: **S31c** (31 mg, 0.0913 mmol, 1.5 equiv), **S19e** (73 mg, 0.0609 mmol as judged by ^{1}H NMR analysis, 1 equiv), DEPBT (27 mg, 0.0913 mmol, 1.5 equiv) and DIPEA (48 µL, 0.2740, 4.5 equiv) were used to provide **46** (7.6 mg, 22%).

^{1}H NMR (600 MHz, DMSO-*d*$_6$): δ 8.70 (br s, 1H), 7.93 (d, *J* = 9.3 Hz, 1H), 7.60 (d, *J* = 8.1 Hz, 1H), 7.42 (t, *J* = 6.4 Hz, 1H), 7.00 (dd, *J* = 15.1, 10.8 Hz, 1H), 6.40 (d, *J* = 13.4 Hz, 1H), 6.22 – 6.16 (m, 2H), 6.15 – 6.07 (m, 2H), 4.82 (d, *J* = 6.7 Hz, 1H), 4.68 (br s, 1H), 4.48 (dd, *J* = 9.4, 2.7 Hz, 1H), 4.38 (br s, 2H), 3.62 – 3.46 (m, 2H), 3.09 – 2.92 (m, 2H), 2.13 (q, *J* = 7.2 Hz, 2H), 1.84 (br s, 1H), 1.60 (d, *J* = 13.1 Hz, 1H), 1.53 – 1.41 (m, 3H), 1.41 – 1.34 (m, 3H), 1.31 – 1.17 (m, 7H), 1.16 – 1.11 (m, 2H), 0.88 (d, *J* = 6.6 Hz, 3H), 0.85 (d, *J* = 6.6 Hz, 6H), 0.77 (d, *J* = 6.7 Hz, 3H).

^{13}C NMR (151 MHz, DMSO-*d*$_6$): δ 171.0, 170.1, 167.7, 165.4, 143.0, 142.3, 139.9, 128.6, 123.3, 123.0, 76.0, 67.0, 55.2, 51.2, 44.8, 42.6, 38.4, 32.3, 30.8, 28.8, 28.4, 27.4, 26.6, 22.5, 19.1, 19.0, 18.5.

HRMS (ESI): calculated for $C_{30}H_{51}N_4O_6^+$ ($[M+H]^+$) 563.3803, found 563.3801

$[\alpha]_D^{27}$ = –75.0 (*c* 0.1, MeOH)

Synthesis of fatty acid analogs **47**–**51**

Scheme S11. General synthetic route to fatty acid analogs **47**–**51**.

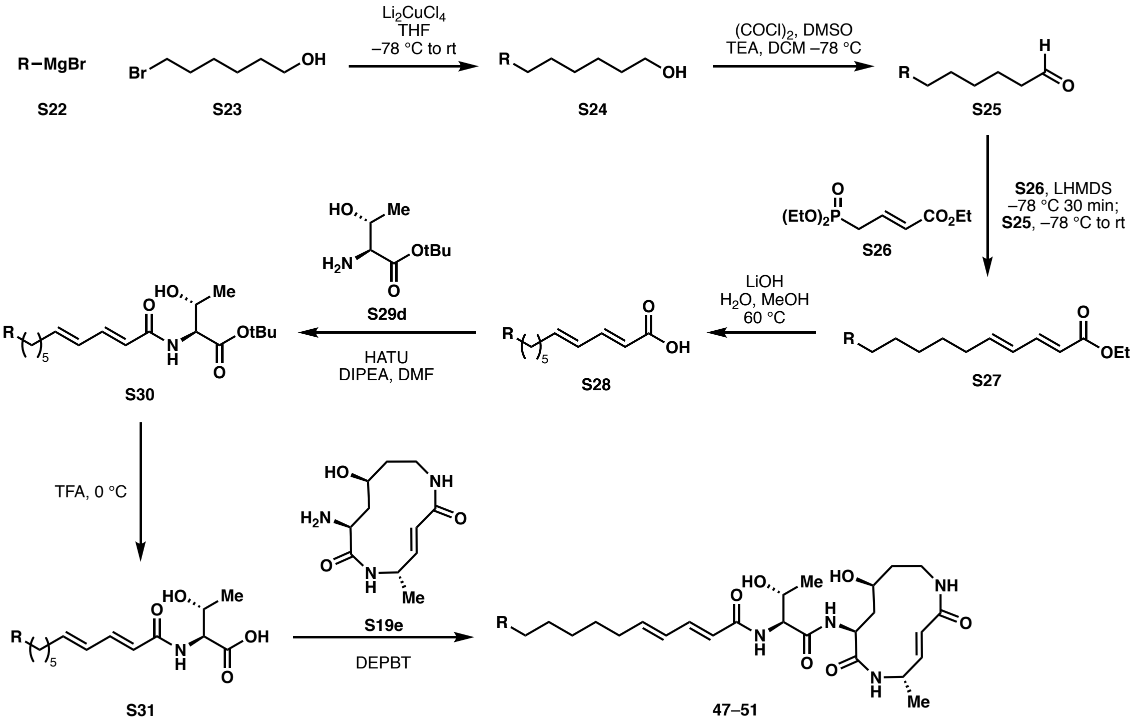

Synthesis of *tert*-butyl cepafungin (**47**):

7,7-dimethyloctan-1-ol (S24a)

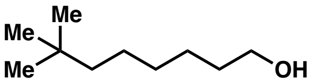

S24a

Following General Procedure G: *tert*-butylmagnesium chloride **S22a** was used to provide **S24a** (3.90 g, 89%) as a colorless oil.

^{1}H NMR (400 MHz, $CDCl_3$): δ 3.63 (t, J = 6.7 Hz, 2H), 1.62 – 1.51 (m, 2H), 1.42 – 1.10 (m, 9H), 0.85 (s, 9H).

^{13}C NMR (101 MHz, $CDCl_3$): δ 63.2, 44.3, 32.9, 30.5, 30.4, 29.5, 25.9, 24.6.

7,7-dimethyloctanal (S25a)

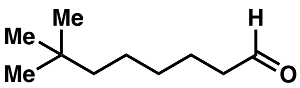

S25a

Following General Procedure H: **S24a** (2.0 g, 12.6 mmol, 1 equiv) was used to provide **S25a** (2.59 g), used without further purification.

^{1}H NMR (400 MHz, CDCl$_3$): δ 9.76 (t, *J* = 1.9 Hz, 1H), 2.42 (td, *J* = 7.4, 1.9 Hz, 2H), 1.70 – 1.57 (m, 2H), 1.33 – 1.13 (m, 6H), 0.86 (s, 9H).

ethyl (2*E*,4*E*)-11,11-dimethyldodeca-2,4-dienoate (S27a)

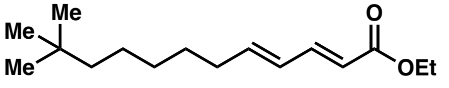

S27a

Following General Procedure I: **S25a** (2.59 g) was used to provide **S27a** (1.75 g, 55% over 2 steps) as a colorless oil.

^{1}H NMR (400 MHz, CDCl$_3$): δ 7.31 – 7.20 (m, 1H), 6.21 – 6.07 (m, 2H), 5.77 (d, *J* = 15.5 Hz, 1H), 4.19 (q, *J* = 7.1 Hz, 2H), 2.16 (td, *J* = 7.3, 5.8 Hz, 2H), 1.49 – 1.37 (m, 2H), 1.31 – 1.22 (m, 7H), 1.18 – 1.11 (m, 2H), 0.85 (s, 9H).

^{13}C NMR (101 MHz, CDCl$_3$): δ 167.5, 145.3, 144.9, 128.5, 119.3, 60.3, 44.3, 33.2, 30.4, 30.3, 29.5, 28.9, 24.5, 14.5.

HRMS (ESI): calculated for $C_{16}H_{29}O_2^+$ ([M+H]$^+$) 253.2162, found 253.2159

(2*E*,4*E*)-11,11-dimethyldodeca-2,4-dienoic acid (S28b)

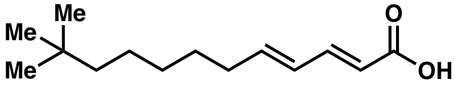

S28b

Following General Procedure J: **S27a** (700 mg, 2.77 mmol, 1 equiv) was used to provide **S28b** (648 mg, quant.) as a white solid.

^{1}H NMR (400 MHz, CDCl$_3$): δ 7.41 – 7.29 (m, 1H), 6.25 – 6.14 (m, 2H), 5.78 (d, *J* = 15.3 Hz, 1H), 2.23 – 2.13 (m, 2H), 1.49 – 1.40 (m, 2H), 1.31 – 1.22 (m, 4H), 1.15 (ddt, *J* = 12.2, 5.3, 2.6 Hz, 2H), 0.86 (s, 9H).

^{13}C NMR (101 MHz, CDCl$_3$): δ 172.7, 147.7, 146.5, 128.4, 118.3, 44.3, 33.2, 30.4, 30.3, 29.5, 28.8, 24.5.

HRMS (ESI): calculated for $C_{14}H_{25}O_2^+$ ([M+H]$^+$) 225.1849, found 225.1849

***tert*-butyl ((2*E*,4*E*)-11,11-dimethyldodeca-2,4-dienoyl)-*L*-threoninate (S30d)**

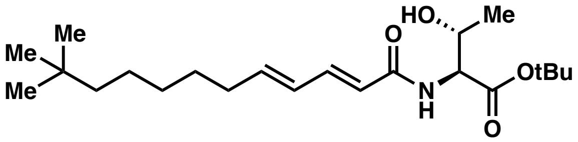

S30d

Following General Procedure K: **S28b** (320 mg, 1.43 mmol, 1 equiv), **S29d** (495 mg, 2.14 mmol, 1.5 equiv), HATU (569 mg, 1.50 mmol, 1.05 equiv) and DIPEA (745 μL, 4.28 mmol, 3 equiv) were used to provide **S30d** (303 mg, 49%) as a colorless oil.

^{1}H NMR (400 MHz, CDCl$_3$): δ 7.21 (dd, *J* = 15.0, 10.3 Hz, 1H), 6.23 – 6.00 (m, 3H), 5.87 (d, *J* = 15.0 Hz, 1H), 4.49 (dd, *J* = 9.2, 2.1 Hz, 1H), 4.21 (qd, *J* = 6.3, 2.2 Hz, 1H), 2.22 – 2.07 (m, 2H), 1.49 – 1.37 (m, 11H), 1.30 – 1.21 (m, 4H), 1.19 – 1.11 (m, 14H), 0.85 (s, 9H).

^{13}C NMR (101 MHz, CDCl$_3$): δ 170.2, 166.6, 143.6, 141.9, 128.4, 121.6, 82.0, 73.9, 67.6, 58.4, 44.3, 33.1, 30.4, 30.2, 29.5, 29.0, 28.8, 28.3, 24.5, 21.0.

HRMS (ESI): calculated for $C_{22}H_{40}NO_4^+$ ($[M+H]^+$) 438.3578, found 438.3573

$[\alpha]_D^{27}$ = +5.3 (*c* 0.6, MeOH)

((2*E*,4*E*)-11,11-dimethyldodeca-2,4-dienoyl)-*L*-threonine (S31d)

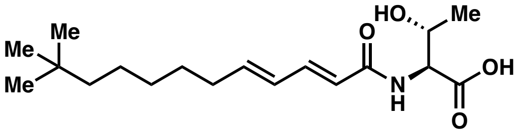

S31d

Following General Procedure L: **S31d** (108 mg, 0.247 mmol, 1 equiv) was used to provide **S31d** (96 mg, quant.) as a clear, colorless resin.

^{1}H NMR (400 MHz, CDCl$_3$): δ 7.25 – 7.19 (m, 1H), 7.02 (d, *J* = 8.2 Hz, 1H), 6.14 (s, 2H), 5.93 (d, *J* = 15.1 Hz, 1H), 4.68 – 4.59 (m, 1H), 4.54 – 4.45 (m, 1H), 2.15 (d, *J* = 6.7 Hz, 2H), 1.46 – 1.38 (m, 2H), 1.30 – 1.20 (m, 8H), 1.18 – 1.12 (m, 2H), 0.86 (s, 9H).

^{13}C NMR (101 MHz, CDCl$_3$): δ 173.6, 168.8, 145.6, 144.0, 128.2, 120.0, 67.7, 57.9, 44.3, 33.3, 30.4, 30.4, 29.6, 29.0, 24.5, 19.3.

HRMS (ESI): calculated for $C_{18}H_{32}NO_4^+$ ($[M+H]^+$) 326.2326, found 325.2329

$[\alpha]_D^{27}$ = +12.6 (*c* 0.3, MeOH)

(2*E*,4*E*)-*N*-((2*S*,3*R*)-3-hydroxy-1-(((5*S*,8*S*,10*S*,*E*)-10-hydroxy-5-methyl-2,7-dioxo-1,6-diazacyclododec-3-en-8-yl)amino)-1-oxobutan-2-yl)-11,11-dimethyldodeca-2,4-dienamide (47)

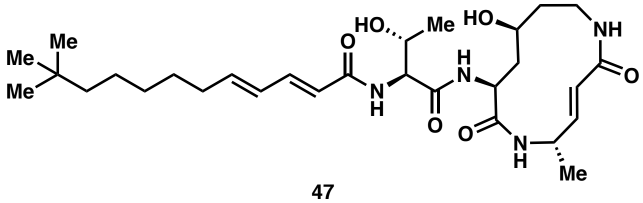

47

Following General Procedure F: **S31d** (38 mg, 0.116 mmol, 2 equiv) and **S19e** (70 mg, 0.0580 mmol as judged by ^{1}H NMR analysis, 1 equiv) were used to provide **47** (9.6 mg, 30%) as a fluffy white solid.

^{1}H NMR (600 MHz, DMSO-*d*$_6$): δ 8.66 (br s, 1H), 7.90 (d, *J* = 8.7 Hz, 1H), 7.74 (d, *J* = 7.8 Hz, 1H), 7.41 (t, *J* = 6.4 Hz, 1H), 7.00 (dd, *J* = 15.1, 10.8 Hz, 1H), 6.40 (d, *J* = 14.1 Hz, 1H), 6.23 – 6.05 (m, 4H), 4.86 (d, *J* = 4.9 Hz, 1H), 4.67 (s, 1H), 4.50 – 4.31 (m, 2H), 4.28 (dd, *J* = 8.8, 4.1 Hz, 1H), 3.96 (s, 1H), 3.57 (s, 1H), 3.07 – 2.93 (m, 2H), 2.13 (q, *J* = 7.2 Hz, 2H), 1.85 (s, 1H), 1.58 (d, *J* = 13.1 Hz, 1H), 1.49 – 1.34 (m, 4H), 1.28 – 1.18 (m, 7H), 1.16 – 1.11 (m, 2H), 1.00 (d, *J* = 6.3 Hz, 3H), 0.85 (s, 9H).

^{13}C NMR (151 MHz, DMSO-*d*$_6$): δ 171.0, 169.4, 167.6, 165.4, 143.1, 142.1, 139.7, 128.6, 123.3, 123.1, 67.0, 66.7, 58.1, 51.2, 44.7, 43.7, 42.5, 32.3, 30.1, 29.6, 29.3, 28.4, 23.9, 19.9, 18.5.

HRMS (ESI): calculated for $C_{29}H_{49}N_4O_6^+$ ([M+H]$^+$) 549.3645, found 549.3645

$[\alpha]_D^{27}$ = –86.4 (*c* 0.1, MeOH)

Synthesis of cyclopentyl cepafungin (**48**)

6-cyclopentylhexan-1-ol (S24b)

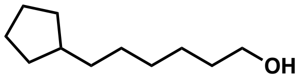

S24b

Following General Procedure G: Cyclopentylmagnesium bromide **S22b** was used to provide **S24b** (4.18 g, 89%) as a colorless oil.

^{1}H NMR (400 MHz, CDCl$_3$): δ 3.61 (t, *J* = 6.7 Hz, 2H), 1.77 – 1.66 (m, 3H), 1.65 – 1.40 (m, 7H), 1.40 – 1.22 (m, 8H), 1.12 – 0.95 (m, 2H).

^{13}C NMR (101 MHz, CDCl$_3$): δ 63.1, 40.3, 36.3, 32.9, 32.8, 29.8, 28.9, 25.9, 25.3.

6-cyclopentylhexanal (S25b)

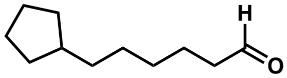

S25b

Following General Procedure H: **S24b** (2.50 g, 14.7 mmol, 1 equiv) was used to provide **S25b** (3.12 g), used without further purification.

^{1}H NMR (400 MHz, $CDCl_3$): δ 9.76 (t, *J* = 1.9 Hz, 0H), 2.41 (td, *J* = 7.4, 1.9 Hz, 2H), 1.77 – 1.68 (m, 3H), 1.65 – 1.45 (m, 6H), 1.35 – 1.25 (m, 7H), 1.09 – 1.00 (m, 2H).

ethyl (2*E*,4*E*)-10-cyclopentyldeca-2,4-dienoate (S27b)

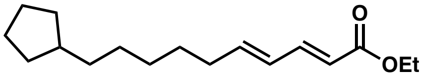

S27b

Following General Procedure I: **S25b** (3.12 g) was used to provide **S27b** (1.44 g, 37%) as a colorless oil.

^{1}H NMR (400 MHz, $CDCl_3$): δ 7.30 – 7.19 (m, 1H), 6.22 – 6.06 (m, 2H), 5.77 (d, *J* = 15.4 Hz, 1H), 4.19 (q, *J* = 7.1 Hz, 2H), 2.15 (td, *J* = 7.4, 5.9 Hz, 2H), 1.78 – 1.66 (m, 4H), 1.60 – 1.55 (m, 2H), 1.52 – 1.46 (m, 2H), 1.45 – 1.38 (m, 2H), 1.30 – 1.26 (m, 8H), 1.09 – 0.99 (m, 2H).

^{13}C NMR (101 MHz, $CDCl_3$): δ 167.5, 145.3, 144.9, 128.4, 119.3, 60.3, 40.2, 36.3, 33.1, 32.8, 29.6, 28.9, 28.7, 25.3, 14.4.\

HRMS (ESI): calculated for $C_{17}H_{29}O_2^+$ ([M+H]$^+$) 265.2162, found 265.2160

(2*E*,4*E*)-10-cyclopentyldeca-2,4-dienoic acid (S28c)

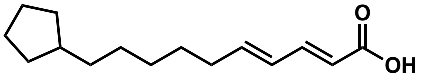

S28c

Following General Procedure J: **S27b** (718 mg, 2.72 mmol, 1 equiv) was used to provide **S28c** (645 mg, quant.) as a white solid.

^{1}H NMR (400 MHz, $CDCl_3$): δ 7.35 (ddd, *J* = 15.4, 6.8, 3.2 Hz, 1H), 6.25 – 6.13 (m, 2H), 5.78 (d, *J* = 15.3 Hz, 1H), 2.23 – 2.13 (m, 2H), 1.78 – 1.67 (m, 3H), 1.63 – 1.54 (m, 2H), 1.54 – 1.47 (m, 2H), 1.47 – 1.40 (m, 2H), 1.32 – 1.26 (m, 6H), 1.10 – 1.00 (m, 2H).

^{13}C NMR (101 MHz, $CDCl_3$): δ 172.9, 147.7, 146.5, 128.3, 118.3, 40.3, 36.3, 33.2, 32.9, 29.6, 28.8, 28.7, 25.3.

HRMS (ESI): calculated for $C_{15}H_{25}O_2^+$ ([M+H]$^+$) 237.1849, found 237.1847

tert-butyl ((2_E_,4_E_)-10-cyclopentyldeca-2,4-dienoyl)-_L_-threoninate (S30e)

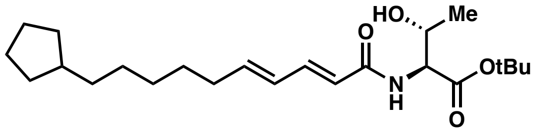

S30e

Following General Procedure K: **S28c** (350 mg, 1.48 mmol, 1 equiv), **S29d** (514 mg, 2.22 mmol, 1.5 equiv), HATU (591 mg, 1.56 mmol, 1.05 equiv) and DIPEA (774 µL, 4.44 mmol, 3 equiv) were used to provide **S30e** (457 mg, 69%) as a colorless oil.

^{1}H NMR (400 MHz, CDCl$_3$): δ 7.20 (dd, *J* = 15.0, 10.3 Hz, 1H), 6.20 – 6.01 (m, 3H), 5.87 (d, *J* = 15.0 Hz, 1H), 4.49 (dd, *J* = 9.2, 2.1 Hz, 1H), 4.21 (qd, *J* = 6.3, 2.2 Hz, 1H), 2.14 (q, *J* = 7.0 Hz, 2H), 1.77 – 1.67 (m, 3H), 1.62 – 1.47 (m, 4H), 1.45 (s, 9H), 1.43 – 1.36 (m, 2H), 1.30 – 1.24 (m, 6H), 1.18 – 1.13 (m, 12H), 1.10 – 0.99 (m, 2H).

^{13}C NMR (101 MHz, CDCl$_3$): δ 170.2, 166.6, 143.6, 141.9, 128.4, 121.6, 82.0, 73.9, 67.6, 58.4, 40.2, 36.3, 33.1, 32.9, 29.6, 29.0, 28.9, 28.7, 28.3, 25.3, 21.0.

HRMS (ESI): calculated for $C_{23}H_{40}NO_4^+$ ([M+H]$^+$) 450.3578, found 450.3589

$[\alpha]_D^{27}$ = +5.5 (*c* 0.4, MeOH)

((2_E_,4_E_)-10-cyclopentyldeca-2,4-dienoyl)-_L_-threonine (S31e)

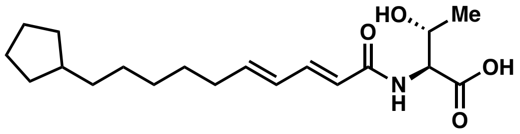

S31e

Following General Procedure L: **S30e** (62 mg, 0.138 mmol, 1 equiv) was used to provide **S31e** (57 mg, quant.) as a clear, colorless resin.

^{1}H NMR (400 MHz, CDCl$_3$): δ 7.25 – 7.14 (m, 1H), 6.88 (d, *J* = 7.9 Hz, 1H), 6.59 (br s, 1H), 6.21 – 6.06 (m, 2H), 5.91 (d, *J* = 15.0 Hz, 1H), 4.61 (d, *J* = 7.8 Hz, 1H), 4.54 – 4.43 (m, 1H), 2.21 – 2.08 (m, 2H), 1.79 – 1.66 (m, 3H), 1.63 – 1.45 (m, 4H), 1.45 – 1.36 (m, 2H), 1.34 – 1.25 (m, 7H), 1.23 (d, *J* = 6.3 Hz, 3H), 1.10 – 0.99 (m, 2H).

^{13}C NMR (101 MHz, CDCl$_3$): δ 173.4, 168.6, 145.4, 143.8, 128.2, 120.2, 67.5, 57.8, 40.3, 36.3, 33.3, 32.9, 29.7, 28.9, 28.7, 25.3, 19.2.

HRMS (ESI): calculated for $C_{19}H_{32}NO_4^+$ ([M+H]$^+$) 338.2326, found 338.2327

$[\alpha]_D^{27}$ = +12.4 (*c* 0.3, MeOH)

(2*E*,4*E*)-10-cyclopentyl-*N*-((2*S*,3*R*)-3-hydroxy-1-(((5*S*,8*S*,10*S*,*E*)-10-hydroxy-5-methyl-2,7-dioxo-1,6-diazacyclododec-3-en-8-yl)amino)-1-oxobutan-2-yl)deca-2,4-dienamide (48)

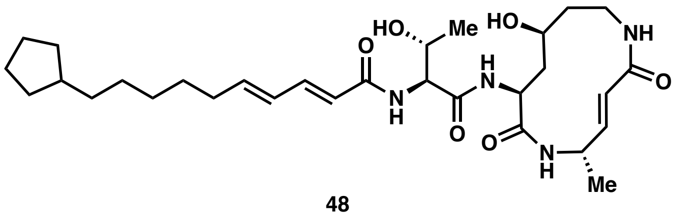

48

Following General Procedure F: **S31e** (41 mg, 0.121 mmol, 2 equiv) and **S19e** (73 mg, 0.0603 mmol as judged by ^{1}H NMR analysis, 1 equiv) were used to provide **48** (12.7 mg, 38%) as a fluffy white solid.

^{1}H NMR (600 MHz, DMSO-d_6): δ 8.77 – 8.40 (m, 1H), 7.92 (d, *J* = 8.7 Hz, 1H), 7.84 – 7.64 (m, 1H), 7.41 (t, *J* = 6.4 Hz, 1H), 7.00 (dd, *J* = 15.2, 10.7 Hz, 1H), 6.40 (d, *J* = 15.6 Hz, 1H), 6.23 – 6.04 (m, 4H), 4.89 (s, 1H), 4.68 (s, 1H), 4.51 – 4.31 (m, 2H), 4.28 (dd, *J* = 8.9, 4.1 Hz, 1H), 3.96 (s, 1H), 3.57 (s, 1H), 3.12 – 2.87 (m, 2H), 2.13 (q, *J* = 7.1 Hz, 2H), 1.84 (br s, 1H), 1.75 – 1.66 (m, 3H), 1.63 – 1.50 (m, 3H), 1.50 – 1.41 (m, 3H), 1.41 – 1.33 (m, 3H), 1.32 – 1.23 (m, 7H), 1.23 – 1.18 (m, 2H), 1.07 – 1.02 (m, 2H), 1.00 (d, *J* = 6.3 Hz, 3H).

^{13}C NMR (151 MHz, DMSO-d_6): δ 171.1, 169.4, 167.6, 165.4, 143.1, 142.1, 139.7, 128.6, 123.3, 123.1, 67.0, 66.7, 58.1, 51.2, 44.7, 42.5, 35.6, 32.3, 28.9, 28.4, 28.0, 24.7, 19.9, 18.5.

HRMS (ESI): calculated for $C_{30}H_{49}N_4O_6^+$ ([M+H]$^+$) 561.3647, found 561.3655

$[\alpha]_D^{27}$ = –98.4 (*c* 0.2, MeOH)

Synthesis of cyclohexyl cepafungin (**49**)

6-cyclohexylhexan-1-ol (S24c)

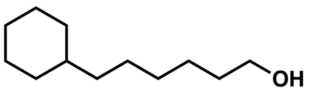

S24c

Following General Procedure G: Cyclohexylmagnesium bromide **S22c** was used to provide **S24c** (4.58 g, 90%) as a colorless oil.

^{1}H NMR (400 MHz, CDCl$_3$): δ 3.61 (t, *J* = 6.7 Hz, 2H), 1.73 – 1.59 (m, 6H), 1.58 – 1.50 (m, 2H), 1.38 – 1.23 (m, 6H), 1.23 – 1.05 (m, 6H), 0.90 – 0.77 (m, 2H).

^{13}C NMR (101 MHz, CDCl$_3$): δ 63.1, 37.8, 37.6, 33.6, 32.9, 29.9, 26.9, 26.9, 26.6, 25.9.

6-cyclohexylhexanal (S25c)

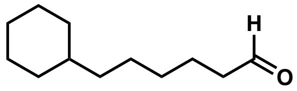

S25c

Following General Procedure H: **S24c** (2.50 g, 13.6 mmol, 1 equiv) was used to provide **S25c** (3.30 g), used without further purification.

^{1}H NMR (400 MHz, $CDCl_3$): δ 9.76 (t, *J* = 1.9 Hz, 1H), 2.41 (td, *J* = 7.4, 1.9 Hz, 2H), 1.71 – 1.58 (m, 8H), 1.35 – 1.26 (m, 5H), 1.18 – 1.12 (m, 4H), 0.85 – 0.82 (m, 2H).

ethyl (2*E*,4*E*)-10-cyclohexyldeca-2,4-dienoate (S27c)

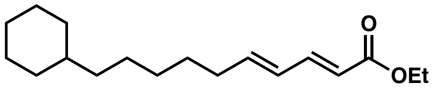

S27c

Following General Procedure I: **S25c** (3.30 g) was used to provide **S27c** (1.62 g, 43% over 2 steps) as a colorless oil.

^{1}H NMR (400 MHz, $CDCl_3$): δ 7.30 – 7.21 (m, 1H), 6.22 – 6.05 (m, 2H), 5.77 (d, *J* = 15.3 Hz, 1H), 4.19 (q, *J* = 7.1 Hz, 2H), 2.15 (td, *J* = 7.5, 6.0 Hz, 2H), 1.72 – 1.62 (m, 5H), 1.46 – 1.37 (m, 2H), 1.28 (t, *J* = 7.1 Hz, 7H), 1.23 – 1.12 (m, 6H), 0.90 – 0.79 (m, 2H).

^{13}C NMR (101 MHz, $CDCl_3$): δ 167.5, 145.3, 144.9, 128.4, 119.3, 60.3, 37.8, 37.5, 33.6, 33.1, 29.6, 28.9, 26.9, 26.8, 26.6, 14.5.

HRMS (ESI): calculated for $C_{18}H_{31}O_2^+$ ($[M+H]^+$) 279.2319, found 279.2318

(2*E*,4*E*)-10-cyclohexyldeca-2,4-dienoic acid (S28d)

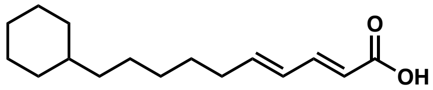

S28d

Following General Procedure J: **S27c** (800 mg, 2.87 mmol, 1 equiv) was used to provide **S28d** (717 mg, quant.) as a white solid.

^{1}H NMR (400 MHz, $CDCl_3$): δ 7.35 (ddd, *J* = 15.3, 6.8, 3.2 Hz, 1H), 6.26 – 6.12 (m, 2H), 5.78 (d, *J* = 15.3 Hz, 1H), 2.23 – 2.13 (m, 2H), 1.73 – 1.59 (m, 5H), 1.43 (s, 2H), 1.32 – 1.24 (m, 4H), 1.24 – 1.13 (m, 6H), 0.91 – 0.79 (m, 2H).

^{13}C NMR (101 MHz, $CDCl_3$): δ 172.8, 147.6, 146.4, 128.2, 118.2, 37.6, 37.4, 33.4, 33.1, 29.5, 28.7, 26.8, 26.6, 26.5.

HRMS (ESI): calculated for $C_{16}H_{27}O_2^+$ ($[M+H]^+$) 251.2006, found 251.2004

***tert*-butyl ((2*E*,4*E*)-10-cyclohexyldeca-2,4-dienoyl)-*L*-threoninate (S30f)**

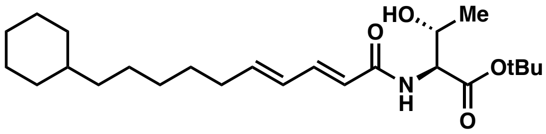

S30f

Following General Procedure K: **S28d** (350 mg, 1.40 mmol, 1 equiv), **S29d** (485 mg, 2.10 mmol, 1.5 equiv), HATU (558 mg, 1.47 mmol, 1.05 equiv) and DIPEA (731 μL, 4.19 mmol, 3 equiv) were used to provide **S30f** (582 mg, 90%).

^{1}H NMR (400 MHz, CDCl$_3$): δ 7.20 (dd, *J* = 15.0, 10.4 Hz, 1H), 6.22 – 5.99 (m, 3H), 5.87 (d, *J* = 15.0 Hz, 1H), 4.49 (dd, *J* = 9.2, 2.1 Hz, 1H), 4.21 (qd, *J* = 6.2, 2.2 Hz, 1H), 2.14 (q, *J* = 7.1 Hz, 2H), 1.71 – 1.59 (m, 5H), 1.45 (s, 9H), 1.43 – 1.35 (m, 2H), 1.30 – 1.20 (m, 5H), 1.20 – 1.11 (m, 17H), 0.90 – 0.78 (m, 2H).

^{13}C NMR (101 MHz, CDCl$_3$): δ 170.2, 166.6, 143.6, 141.9, 128.4, 121.6, 82.0, 73.9, 67.6, 58.4, 37.8, 37.6, 33.6, 33.1, 29.6, 29.0, 28.8, 28.2, 26.9, 26.8, 26.6, 21.0.

HRMS (ESI): calculated for $C_{24}H_{42}NO_4^+$ ($[M+H]^+$) 464.3734, found 464.3733

$[\alpha]_D^{27}$ = +5.5 (*c* 0.7, MeOH)

((2*E*,4*E*)-10-cyclohexyldeca-2,4-dienoyl)-*L*-threonine (S31f)

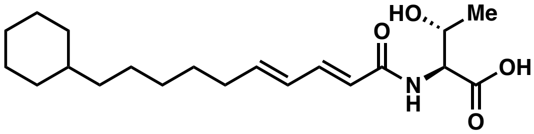

S31f

Following General Procedure L: **S30f** (60 mg, 0.129 mmol, 1 equiv) was used to provide **S31f** (61 mg, quant.).

^{1}H NMR (400 MHz, CDCl$_3$): δ 7.26 – 7.15 (m, 1H), 6.87 (d, *J* = 8.0 Hz, 1H), 6.32 (br s, 1H), 6.19 – 6.07 (m, 2H), 5.91 (d, *J* = 15.0 Hz, 1H), 4.61 (dd, *J* = 8.1, 2.3 Hz, 1H), 4.55 – 4.40 (m, 1H), 2.15 (q, *J* = 6.7 Hz, 2H), 1.73 – 1.59 (m, 5H), 1.45 – 1.36 (m, 2H), 1.33 – 1.20 (m, 8H), 1.20 – 1.12 (m, 5H), 0.91 – 0.79 (m, 2H).

^{13}C NMR (101 MHz, CDCl$_3$): δ 173.4, 168.5, 145.3, 143.7, 128.2, 120.2, 67.5, 57.8, 37.8, 37.6, 33.6, 33.3, 29.7, 29.0, 26.9, 26.8, 26.6, 19.2.

HRMS (ESI): calculated for $C_{20}H_{34}NO_4^+$ ($[M+H]^+$) 352.2482, found 352.2483

$[\alpha]_D^{27}$ = +13.4 (*c* 0.4, MeOH)

(2*E*,4*E*)-10-cyclohexyl-*N*-((2*S*,3*R*)-3-hydroxy-1-(((5*S*,8*S*,10*S*,*E*)-10-hydroxy-5-methyl-2,7-dioxo-1,6-diazacyclododec-3-en-8-yl)amino)-1-oxobutan-2-yl)deca-2,4-dienamide (49)

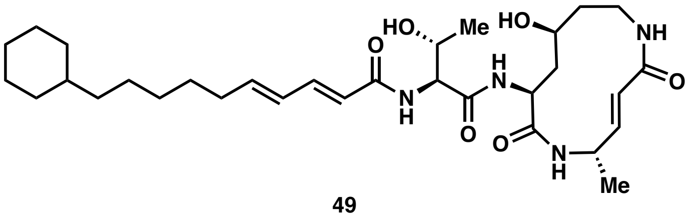

49

Following General Procedure F: **S31f** (49 mg, 0.139 mmol, 2 equiv) and **S19e** (84 mg, 0.0693 mmol as judged by ^{1}H NMR analysis, 1 equiv) were used to provide **49** (16.5 mg, 41%) as a fluffy white solid.

^{1}H NMR (600 MHz, DMSO-*d*$_6$): δ 8.85 – 8.35 (m, 1H), 7.97 – 7.85 (m, 1H), 7.82 – 7.65 (m, 1H), 7.41 (t, *J* = 6.3 Hz, 1H), 7.00 (dd, *J* = 15.1, 10.8 Hz, 1H), 6.40 (d, *J* = 14.6 Hz, 1H), 6.24 – 6.02 (m, 4H), 4.88 (s, 1H), 4.68 (s, 1H), 4.51 – 4.30 (m, 2H), 4.28 (dd, *J* = 8.8, 4.1 Hz, 1H), 4.03 – 3.88 (m, 1H), 3.64 – 3.51 (m, 1H), 3.13 – 2.85 (m, 2H), 2.12 (q, *J* = 7.2 Hz, 2H), 1.96 – 1.73 (m, 1H), 1.69 – 1.54 (m, 6H), 1.49 – 1.41 (m, 1H), 1.40 – 1.34 (m, 3H), 1.29 – 1.10 (m, 13H), 1.00 (d, *J* = 6.3 Hz, 3H), 0.88 – 0.78 (m, 2H).

^{13}C NMR (151 MHz, DMSO-*d*$_6$): δ 171.1, 169.4, 167.6, 165.4, 143.1, 142.1, 139.7, 128.6, 123.3, 123.1, 66.9, 66.7, 58.1, 51.2, 44.7, 42.5, 37.0, 36.9, 32.9, 32.2, 28.9, 28.4, 26.2, 26.1, 25.9, 19.9, 18.5.

HRMS (ESI): calculated for $C_{31}H_{51}N_4O_6^+$ ([M+H]$^+$) 575.3803, found 575.3808

$[\alpha]_D^{27}$ = –93.1 (*c* 0.2, MeOH)

Synthesis of phenyl cepafungin (**50**)

6-phenylhexan-1-ol (S24d)

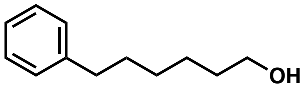

S24d

Following Procedure G: Phenylmagnesium bromide **S22d** was used to provide **S24d** (3.95 g, 80%) as a colorless oil.

^{1}H NMR (400 MHz, CDCl$_3$): δ 7.34 – 7.12 (m, 5H), 3.62 (t, *J* = 6.6 Hz, 2H), 2.67 – 2.55 (m, 2H), 1.68 – 1.52 (m, 5H), 1.44 – 1.33 (m, 4H).

^{13}C NMR (101 MHz, CDCl$_3$): δ 142.8, 128.5, 128.4, 125.7, 63.1, 36.0, 32.8, 31.5, 29.2, 25.7.

HRMS (ESI): calculated for $C_{12}H_{19}O^+$ ([M+H]$^+$) 179.1430, found 179.1430

6-phenylhexanal (S25d)

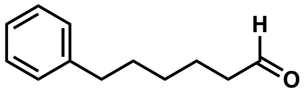

S25d

Following General Procedure H: **S24d** (2.00 g, 11.2 mmol, 1 equiv) was used to provide **S25d**, used immediately without further purification.

^{1}H NMR (400 MHz, CDCl$_3$): δ 9.76 (t, *J* = 1.8 Hz, 1H), 7.30 – 7.25 (m, 2H), 7.20 – 7.15 (m, 3H), 2.64 – 2.59 (m, 2H), 2.45 – 2.40 (m, 2H), 1.71 – 1.60 (m, 4H), 1.42 – 1.33 (m, 2H).

ethyl (2*E*,4*E*)-10-phenyldeca-2,4-dienoate (S27d)

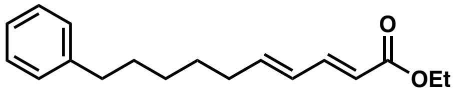

S27d

Following General Procedure I: **S25d** was used to provide **S27d** (1.08 g, 35% over 2 steps) as a colorless oil.

^{1}H NMR (400 MHz, CDCl$_3$): δ 7.31 – 7.21 (m, 3H), 7.20 – 7.15 (m, 3H), 6.21 – 6.06 (m, 2H), 5.78 (d, *J* = 15.4 Hz, 1H), 4.20 (q, *J* = 7.1 Hz, 2H), 2.65 – 2.56 (m, 2H), 2.16 (q, *J* = 7.0 Hz, 2H), 1.68 – 1.58 (m, 2H), 1.51 – 1.42 (m, 2H), 1.40 – 1.33 (m, 2H), 1.29 (t, *J* = 7.1 Hz, 3H).

^{13}C NMR (101 MHz, CDCl$_3$): δ 167.5, 145.2, 144.6, 142.8, 128.6, 128.5, 128.4, 125.8, 119.4, 60.3, 36.0, 33.0, 31.4, 28.9, 28.7, 14.5.

HRMS (ESI): calculated for $C_{18}H_{25}O_2^+$ ([M+H]$^+$) 273.1849, found 273.1848

(2*E*,4*E*)-10-phenyldeca-2,4-dienoic acid (S28e)

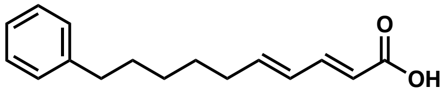

S28e

Following General Procedure J: **S27d** (500 mg, 1.84 mmol, 1 equiv) was used to provide **S28e** (474 mg, quant.) as a white solid.

^{1}H NMR (400 MHz, CDCl$_3$): δ 7.38 – 7.31 (m, 1H), 7.30 – 7.25 (m, 2H), 7.21 – 7.15 (m, 3H), 6.24 – 6.12 (m, 2H), 5.79 (d, *J* = 15.2 Hz, 1H), 2.64 – 2.58 (m, 2H), 2.18 (q, *J* = 7.0 Hz, 2H), 1.68 – 1.59 (m, 2H), 1.47 (tt, *J* = 8.0, 6.7 Hz, 2H), 1.40 – 1.33 (m, 2H).

^{13}C NMR (101 MHz, CDCl$_3$): δ 172.92, 147.65, 146.21, 142.72, 128.51, 128.44, 128.41, 125.80, 118.44, 35.95, 33.09, 31.37, 28.91, 28.61.

HRMS (ESI): calculated for $C_{16}H_{21}O_2^+$ ([M+H]$^+$) 245.1536, found 245.1535

***tert*-butyl ((2*E*,4*E*)-10-phenyldeca-2,4-dienoyl)-*L*-threoninate (S30g)**

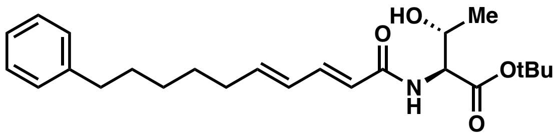

S30g

Following General Procedure K: **S28e** (237 mg, 0.970 mmol, 1 equiv), **S29d** (337 mg, 1.46 mmol, 1.5 equiv), HATU (387 mg, 1.02 mmol, 1.05 equiv) and DIPEA (507 µL, 2.91 mmol, 3 equiv) were used to provide **S30g** (253 mg, 57%) as a colorless oil.

^{1}H NMR (400 MHz, CDCl$_3$): δ 7.32 – 7.21 (m, 2H), 7.22 – 7.14 (m, 4H), 6.20 – 6.00 (m, 3H), 5.88 (d, *J* = 15.0 Hz, 1H), 4.50 (dd, *J* = 9.2, 2.1 Hz, 1H), 4.22 (qd, *J* = 6.3, 2.2 Hz, 1H), 2.64 – 2.57 (m, 2H), 2.15 (q, *J* = 7.0 Hz, 2H), 1.67 – 1.58 (m, 2H), 1.50 – 1.41 (m, 11H), 1.39 – 1.32 (m, 2H), 1.20 – 1.14 (m, 12H).

^{13}C NMR (101 MHz, CDCl$_3$): δ 170.21, 166.60, 143.26, 142.82, 141.82, 128.53, 128.40, 125.77, 121.78, 81.98, 73.94, 67.60, 58.45, 35.99, 32.97, 31.40, 28.91, 28.87, 28.79, 28.27, 21.01.

HRMS (ESI): calculated for $C_{28}H_{44}NO_4^+$ ([M+H]$^+$) 458.3265, found 458.3263

$[\alpha]_D^{27}$ = +4.6 (*c* 0.4, MeOH)

((2*E*,4*E*)-10-phenyldeca-2,4-dienoyl)-*L*-threonine (S31g)

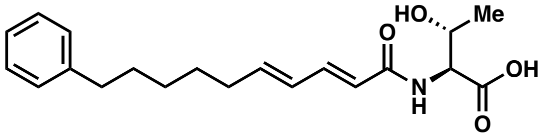

S31g

Following Procedure L: **S30g** (101 mg, 0.221 mmol, 1 equiv) was used to provide **S31g** (93 mg, quant.) as a clear, colorless resin.

^{1}H NMR (400 MHz, $CDCl_3$): δ 7.29 – 7.27 (m, 1H), 7.25 – 7.14 (m, 5H), 6.91 (d, *J* = 8.0 Hz, 1H), 6.52 (br s, 1H), 6.18 – 6.05 (m, 2H), 5.91 (d, *J* = 15.0 Hz, 1H), 4.62 (dd, *J* = 8.1, 2.4 Hz, 1H), 4.53 – 4.44 (m, 1H), 2.63 – 2.55 (m, 2H), 2.14 (q, *J* = 6.7 Hz, 2H), 1.66 – 1.57 (m, 2H), 1.49 – 1.29 (m, 5H), 1.22 (d, *J* = 6.3 Hz, 3H).

^{13}C NMR (101 MHz, $CDCl_3$): δ 173.4, 168.5, 145.1, 143.7, 142.7, 128.5, 128.4, 128.3, 125.8, 120.3, 67.5, 57.8, 36.0, 33.1, 31.4, 29.0, 28.8, 19.3.

HRMS (ESI): calculated for $C_{20}H_{28}NO_4^+$ ($[M+H]^+$) 346.2013, found 346.2014

$[\alpha]_D^{27}$ = +11.4, (*c* 0.3, MeOH)

(2*E*,4*E*)-*N*-((2*S*,3*R*)-3-hydroxy-1-(((5*S*,8*S*,10*S*,*E*)-10-hydroxy-5-methyl-2,7-dioxo-1,6-diazacyclododec-3-en-8-yl)amino)-1-oxobutan-2-yl)-10-phenyldeca-2,4-dienamide (50)

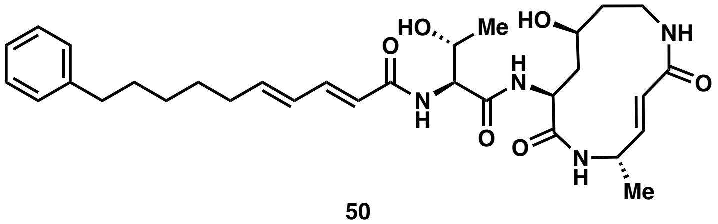

50

Following General Procedure F: **S31g** (189 mg, 0.546 mmol, 2 equiv) and **S19e** (70 mg, 0.0580 mmol as judged by ^{1}H NMR analysis, 1 equiv) were used to provide **50** (44.3 mg, 29%) as a fluffy white powder.

^{1}H NMR (600 MHz, DMSO-*d_6*): δ 8.75 – 8.43 (m, 1H), 7.93 (d, *J* = 8.7 Hz, 1H), 7.75 (d, *J* = 6.5 Hz, 1H), 7.41 (t, *J* = 6.2 Hz, 1H), 7.28 – 7.24 (m, 2H), 7.20 – 7.14 (m, 3H), 6.99 (dd, *J* = 15.1, 10.8 Hz, 1H), 6.40 (d, *J* = 15.8 Hz, 1H), 6.22 – 6.05 (m, 4H), 4.89 (br s, 1H), 4.68 (br s, 1H), 4.53 – 4.31 (m, 2H), 4.28 (dd, *J* = 8.8, 4.1 Hz, 1H), 4.02 – 3.89 (m, 1H), 3.61 – 3.53 (m, 1H), 3.07 – 2.94 (m, 2H), 2.56 (t, *J* = 7.7 Hz, 2H), 2.13 (q, *J* = 7.1 Hz, 2H), 1.95 – 1.76 (m, 1H), 1.62 – 1.53 (m, 3H), 1.49 – 1.34 (m, 4H), 1.33 – 1.26 (m, 2H), 1.25 – 1.15 (m, 3H), 1.00 (d, *J* = 6.3 Hz, 3H).

^{13}C NMR (151 MHz, DMSO-d_6): δ 171.05, 169.38, 167.63, 165.43, 143.11, 142.26, 142.02, 139.72, 128.62, 128.27, 128.23, 125.61, 123.29, 123.12, 66.95, 66.72, 58.13, 51.19, 44.74, 42.46, 35.07, 32.18, 30.78, 28.19, 28.17, 19.93, 18.55.

HRMS (ESI): calculated for $C_{31}H_{45}N_4O_6^+$ ([M+H]$^+$) 569.3334, found 569.3338

$[\alpha]_D^{27}$ = –103.3 (*c* 0.1, MeOH)

Synthesis of trifluoromethyl cepafungin (**51**)

7,7,7-trifluoroheptan-1-ol (52)

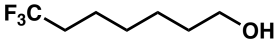

52

Compound **52** was prepared as described by Pitre et al.[24]

7,7,7-trifluoroheptanal (S25e)

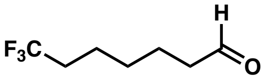

S25e

Following General Procedure H: **S24e** (308 mg, 1.81 mmol, 1 equiv) was used to provide **S25e**, used immediately without further purification.

^{1}H NMR (400 MHz, CDCl$_3$): δ 9.76 (t, *J* = 1.6 Hz, 1H), 2.52 – 2.38 (m, 2H), 2.22 – 1.99 (m, 2H), 1.82 – 1.49 (m, 4H), 1.45 – 1.22 (m, 2H).

HRMS (ESI): calculated for $C_7H_{12}F_3O^+$ ([M+H]$^+$) 169.0835, found 169.0843

ethyl (2*E*,4*E*)-11,11,11-trifluoroundeca-2,4-dienoate (S27e)

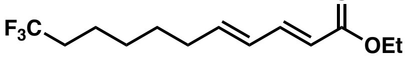

S27e

Following General Procedure I: **S25e** was used to provide **S27e** (120 mg, 25% over 2 steps).

^{1}H NMR (400 MHz, CDCl$_3$): δ 7.25 (dd, *J* = 15.4, 10.3 Hz, 1H), 6.23 – 6.02 (m, 2H), 5.79 (d, *J* = 15.5 Hz, 1H), 4.19 (q, *J* = 7.1 Hz, 2H), 2.18 (q, *J* = 7.0 Hz, 2H), 2.14 – 1.95 (m, 2H), 1.62 – 1.51 (m, 2H), 1.50 – 1.32 (m, 4H), 1.29 (t, *J* = 7.1 Hz, 3H).

^{13}C NMR (101 MHz, CDCl$_3$): δ 167.4, 144.9, 143.9, 128.9, 119.7, 60.3, 53.6, 32.8, 33.8 (q, *J* = 28.4 Hz), 28.4, 28.3, 21.8 (q, *J* = 2.9 Hz), 14.4.

HRMS (ESI): calculated for $C_{13}H_{20}F_3O_2^+$ ([M+H]$^+$) 265.1410, found 265.1409

(2*E*,4*E*)-11,11,11-trifluoroundeca-2,4-dienoic acid (S28f)

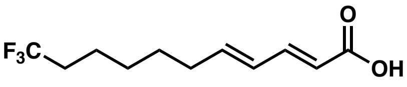

S28f

Following General Procedure J: **S27e** (95 mg, 0.359 mmol, 1 equiv) was used to provide **S28f** (77 mg, 91%).

^{1}H NMR (600 MHz, CDCl$_3$): δ 7.34 (dd, *J* = 15.3, 10.3 Hz, 1H), 6.24 – 6.13 (m, 2H), 5.80 (d, *J* = 15.3 Hz, 1H), 2.23 – 2.18 (m, 2H), 2.12 – 2.01 (m, 2H), 1.62 – 1.52 (m, 2H), 1.52 – 1.44 (m, 2H), 1.43 – 1.35 (m, 2H).

^{13}C NMR (151 MHz, CDCl$_3$): δ 172.7, 147.4, 145.5, 128.7, 118.7, 33.8 (q, *J* = 28.4 Hz), 32.8, 28.3, 21.9 (q, *J* = 2.9 Hz).

HRMS (ESI): calculated for $C_{11}H_{16}F_3O_2^+$ ([M+H]$^+$) 237.1097, found 237.1089

***tert*-butyl ((2*E*,4*E*)-11,11,11-trifluoroundeca-2,4-dienoyl)-*L*-threoninate (S30h)**

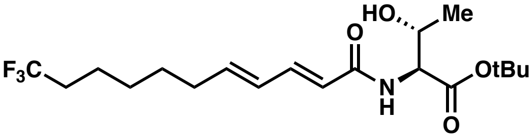

S30h

Following General Procedure K: **S28f** (66 mg, 0.279 mmol, 1 equiv), **S29d** (97 mg, 0.419 mmol, 1.5 equiv), HATU (112 mg, 0.293 mmol, 1.05 equiv) and DIPEA (49 µL, 0.279 mmol, 1 equiv) were used to provide **S30h** (98 mg, 78%).

^{1}H NMR (600 MHz, CDCl$_3$): δ 7.21 (dd, *J* = 15.0, 10.8 Hz, 1H), 6.22 – 6.10 (m, 2H), 6.04 (dt, *J* = 14.7, 6.9 Hz, 1H), 5.89 (d, *J* = 15.0 Hz, 1H), 4.49 (dd, *J* = 9.2, 2.1 Hz, 1H), 4.21 (qd, *J* = 6.2, 2.1 Hz, 1H), 2.21 – 2.14 (m, 2H), 2.12 – 1.99 (m, 2H), 1.61 – 1.51 (m, 2H), 1.51 – 1.41 (m, 11H), 1.41 – 1.33 (m, 2H), 1.20 – 1.14 (m, 12H).

^{13}C NMR (151 MHz, CDCl$_3$): δ 170.2, 166.5, 142.6, 141.7, 128.8, 122.0, 82.0, 73.9, 67.6, 58.5, 33.8 (q, *J* = 28.4 Hz), 32.7, 28.9, 28.5, 28.3, 28.3, 21.8 (q, *J* = 2.9 Hz), 21.0.

HRMS (ESI): calculated for $C_{23}H_{39}F_3NO_4^+$ ([M+H]$^+$) 450.2826, found 450.2815

$[\alpha]_D^{27}$ = +4.6 (*c* 0.3, MeOH)

((2*E*,4*E*)-11,11,11-trifluoroundeca-2,4-dienoyl)-*L*-threonine (S31h)

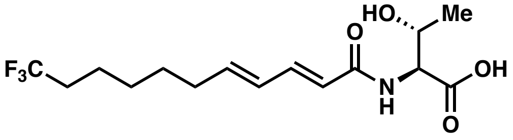

S31h

Following General Procedure L: **S30h** (45 mg, 0.100 mmol, 1 equiv) was used to provide **S31h** (43 mg, quant.)

^{1}H NMR (600 MHz, $CDCl_3$): δ 7.26 – 7.18 (m, 1H), 6.86 (s, 1H), 6.21 – 6.06 (m, 2H), 5.93 (d, *J* = 14.8 Hz, 1H), 4.60 (d, *J* = 7.0 Hz, 1H), 4.49 (s, 1H), 2.21 – 2.14 (m, 2H), 2.12 – 2.01 (m, 2H), 1.56 (p, *J* = 8.1 Hz, 2H), 1.49 – 1.42 (m, 2H), 1.41 – 1.35 (m, 2H), 1.31 – 1.17 (m, 5H).

^{13}C NMR (151 MHz, $CDCl_3$): δ 173.3, 168.5, 144.4, 143.5, 128.6, 120.5, 67.4, 57.8, 33.8 (q, *J* = 28.3 Hz), 32.8, 29.9, 28.4, 28.3, 21.8 (q, *J* = 2.9 Hz), 19.3.

HRMS (ESI): calculated for $C_{15}H_{23}F_3NO_4^+$ ($[M+H]^+$) 338.1574, found 338.1583

$[\alpha]_D^{27}$ = +8.1 (*c* 0.3, MeOH)

(2*E*,4*E*)-11,11,11-trifluoro-*N*-((2*S*,3*R*)-3-hydroxy-1-(((5*S*,8*S*,10*S*,*E*)-10-hydroxy-5-methyl-2,7-dioxo-1,6-diazacyclododec-3-en-8-yl)amino)-1-oxobutan-2-yl)undeca-2,4-dienamide (51)

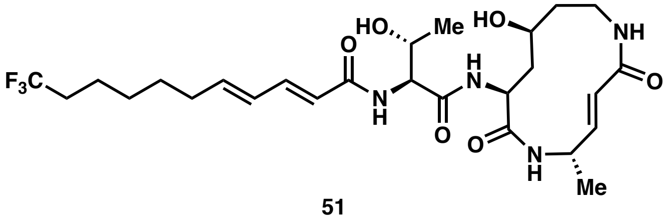

51

Following General Procedure F: **S31h** (34 mg, 0.101 mmol, 2 equiv) and **S19e** (60 mg, 0.0501 mmol as judged by ^{1}H NMR analysis, 1 equiv) were used to provide **50** (8.9 mg, 32%) as a fluffy white solid.

^{1}H NMR (600 MHz, DMSO-*d*$_6$): δ 8.67 (s, 1H), 7.92 (d, *J* = 8.6 Hz, 1H), 7.74 (d, *J* = 7.8 Hz, 1H), 7.41 (t, *J* = 6.2 Hz, 1H), 7.00 (dd, *J* = 15.1, 10.9 Hz, 1H), 6.40 (d, *J* = 14.4 Hz, 1H), 6.24 – 6.05 (m, 4H), 4.87 (d, *J* = 4.6 Hz, 1H), 4.67 (s, 1H), 4.49 – 4.30 (m, 2H), 4.28 (dd, *J* = 8.8, 4.1 Hz, 1H), 4.04 – 3.88 (m, 1H), 3.57 (s, 1H), 3.08 – 2.92 (m, 2H), 2.28 – 2.18 (m, 2H), 2.14 (q, *J* = 6.9 Hz, 2H), 1.85 (s, 1H), 1.58 (d, *J* = 13.2 Hz, 1H), 1.51 – 1.45 (m, 2H), 1.45 – 1.38 (m, 3H), 1.38 – 1.31 (m, 3H), 1.26 – 1.14 (m, 3H), 1.00 (d, *J* = 6.3 Hz, 3H).

^{13}C NMR (151 MHz, DMSO-d_6): δ 171.0, 169.4, 167.6, 165.4, 143.1, 141.8, 139.7, 128.7, 123.3, 123.2, 67.0, 66.7, 58.1, 51.2, 44.8, 42.5, 32.3 (q, *J* = 27.3 Hz), 32.0, 27.9, 27.5, 21.3 (q, *J* = 2.9 Hz), 19.9, 18.5.

HRMS (ESI): calculated for $C_{26}H_{40}F_3N_4O_6^+$ ([M+H]$^+$) 561.2894, found 561.2901

$[\alpha]_D^{27}$ = –80.6 (*c* 0.2, MeOH)

Synthesis of hybrid phenyl-valinyl cepafungin (**S39**)

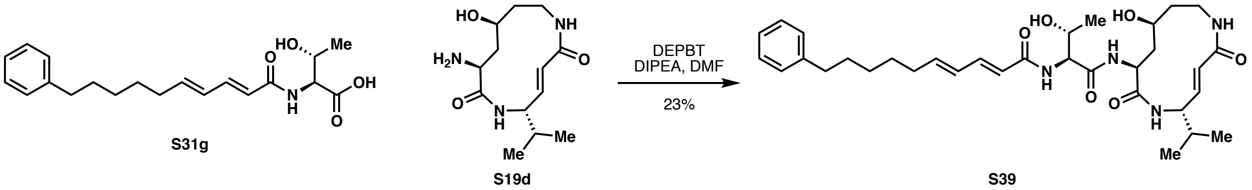

(2*E*,4*E*)-*N*-((2*S*,3*R*)-3-hydroxy-1-(((5*S*,8*S*,10*S*,*E*)-10-hydroxy-5-isopropyl-2,7-dioxo-1,6-diazacyclododec-3-en-8-yl)amino)-1-oxobutan-2-yl)-10-phenyldeca-2,4-dienamide (S39)

Following General Procedure F: **S31g** (38 mg, 0.109 mmol, 2.04 equiv) and **S19d** (56 mg, 0.0535 mmol as judged by ^{1}H NMR analysis, 1 equiv) were used to provide **S39** (7.5 mg, 23%) as a fluffy white solid.

^{1}H NMR (600 MHz, DMSO-d_6): δ 8.48 (d, *J* = 8.6 Hz, 1H), 7.92 (d, *J* = 8.7 Hz, 1H), 7.79 (d, *J* = 7.7 Hz, 1H), 7.42 (t, *J* = 6.3 Hz, 1H), 7.29 – 7.24 (m, 2H), 7.19 – 7.14 (m, 3H), 6.99 (dd, *J* = 15.1, 10.8 Hz, 1H), 6.33 (dd, *J* = 15.9, 6.6 Hz, 1H), 6.23 – 6.03 (m, 4H), 4.84 (d, *J* = 5.1 Hz, 1H), 4.67 (d, *J* = 5.0 Hz, 1H), 4.45 – 4.32 (m, 1H), 4.28 (dd, *J* = 8.8, 4.5 Hz, 1H), 4.10 – 3.99 (m, 1H), 3.99 – 3.84 (m, 1H), 3.65 – 3.49 (m, 1H), 3.05 – 2.93 (m, 2H), 2.56 (t, *J* = 7.7 Hz, 2H), 2.12 (q, *J* = 7.1 Hz, 2H), 1.86 (td, *J* = 12.3, 6.8 Hz, 1H), 1.75 (dq, *J* = 13.6, 6.9 Hz, 1H), 1.61 – 1.53 (m, 3H), 1.45 – 1.35 (m, 4H), 1.32 – 1.26 (m, 2H), 0.99 (d, *J* = 6.3 Hz, 3H), 0.94 – 0.86 (m, 6H).

^{13}C NMR (151 MHz, DMSO-d_6): δ 171.47, 169.37, 167.77, 165.38, 142.27, 141.98, 140.82, 139.68, 128.64, 128.28, 128.24, 125.62, 124.76, 123.16, 66.88, 66.79, 58.17, 55.64, 51.21, 42.25, 35.09, 32.19, 30.80, 30.58, 28.20, 28.18, 19.77, 19.63, 19.12.

HRMS (ESI): calculated for $C_{33}H_{49}N_4O_6^+$ ([M+H]$^+$) 597.3647, found 597.3643

$[\alpha]_D^{27}$ = –70.0 (*c* 0.1, MeOH)

References

1. Hibi, M.; Mori, R.; Miyake, R.; Kawabata, H.; Kozono, S.; Takahashi, S.; Ogawa, J. Novel Enzyme Family Found in Filamentous Fungi Catalyzing Trans-4-Hydroxylation of L-Pipecolic Acid. *Appl. Environ. Microbiol.* **2016**, *82* (7), 2070–2077..

2. Zwick, C. R.; Sosa, M. B.; Renata, H. Modular Chemoenzymatic Synthesis of GE81112 B1 and Related Analogues Enables Elucidation of Its Key Pharmacophores. *J. Am. Chem. Soc.* **2021**, *143* (3), 1673–1679. https://doi.org/10.1021/jacs.0c13424.

3. Stein, M. L.; Beck, P.; Kaiser, M.; Dudler, R.; Becker, C. F. W.; Groll, M. One-Shot NMR Analysis of Microbial Secretions Identifies Highly Potent Proteasome Inhibitor. *Proc. Natl. Acad. Sci. U. S. A.* **2012**, *109* (45), 18367–18371. https://doi.org/10.1073/pnas.1211423109.

4. Amatuni, A.; Shuster, A.; Adibekian, A.; Renata, H. Concise Chemoenzymatic Total Synthesis and Identification of Cellular Targets of Cepafungin I. *Cell Chem. Biol.* **2020**, *27* (10), 1318-1326.e18. https://doi.org/10.1016/j.chembiol.2020.07.012.

5. Schauer, D. J.; Helquist, P. Mild Zinc-Promoted Horner-Wadsworth-Emmons Reactions of Diprotic Phosphonate Reagents. *Synthesis (Stuttg).* **2006**, No. 21, 3654–3660. https://doi.org/10.1055/s-2006-950292.

6. Scott, J. W.; Parker, D.; Parrish, D. R. Improved Syntheses Of Nε-Tert-Butyloxycarbonyl-L-Lysine and Nε-Benzyloxycarbonyl-Nε-Tert-Butyloxycarbonyl-L-Lysine. *Synth. Commun.* **1981**, *11* (4), 303–314. https://doi.org/10.1080/00397918108063610.

7. Raje, P. S.; Kishore, V. Preparation of Amino-Protected Lysine Derivatives. Intl. Patent WO 2001027074, April 19, 2001.

8. Amatuni, A.; Renata, H. Identification of a Lysine 4-Hydroxylase from the Glidobactin Biosynthesis and Evaluation of Its Biocatalytic Potential. *Org. Biomol. Chem.* **2019**, *17* (7), 1736–1739. https://doi.org/10.1039/c8ob02054j.

9. Blanchette, M. A.; Choy, W.; Davis, J. T.; Essenfeld, A. P.; Masamune, S.; Roush, W. R.; Sakai, T. Horner-Wadsworth-Emmons Reaction: Use of Lithium Chloride and an Amine for Base-Sensitive Compounds. *Tetrahedron Lett.* **1984**, *25* (21), 2183–2186. https://doi.org/10.1016/S0040-4039(01)80205-7.

10. Snyder, S. A.; Elsohly, A. M.; Kontes, F. Synthetic and Theoretical Investigations of Myrmicarin Biosynthesis. *Angew. Chemie - Int. Ed.* **2010**, *49* (50), 9693–9698. https://doi.org/10.1002/anie.201005825.

11. Candelon, N.; Shinkaruk, S.; Bennetau, B.; Bennetau-Pelissero, C.; Dumartin, M. L.; Degueil, M.; Babin, P. New Approach to Asymmetrically Substituted Methoxypyrazines, Derivatives of Wine Flavors. *Tetrahedron* **2010**, *66* (13), 2463–2469. https://doi.org/10.1016/j.tet.2010.01.088.

12. Williams, B. D.; Smith, A. B. Total Synthesis of (+)-18-Epi-Latrunculol A: Development of a Synthetic Route. *J. Org. Chem.* **2014**, *79* (19), 9284–9296. https://doi.org/10.1021/jo501733m.

13. De Luca, L.; Giacomelli, G.; Porcheddu, A. A Very Mild and Chemoselective Oxidation of Alcohols to Carbonyl Compounds. *Org. Lett.* **2001**, *3* (19), 3041–3043. https://doi.org/10.1021/ol016501m.

14. Hoover, J. M.; Stahl, S. S. Highly Practical Copper(I)/TEMPO Catalyst System for Chemoselective Aerobic Oxidation of Primary Alcohols. *J. Am. Chem. Soc.* **2011**, *133* (42), 16901–16910. https://doi.org/10.1021/ja206230h.

15. Baud, D.; Saaidi, P. L.; Monfleur, A.; Harari, M.; Cuccaro, J.; Fossey, A.; Besnard, M.; Debard, A.; Mariage, A.; Pellouin, V.; et al. Synthesis of Mono- and Dihydroxylated Amino Acids with New α-Ketoglutarate-Dependent Dioxygenases: Biocatalytic Oxidation of C-H Bonds. *ChemCatChem* **2014**, *6* (10), 3012–3017. https://doi.org/10.1002/cctc.201402498.

16. Melaugh, G.; Giri, N.; Davidson, C. E.; James, S. L.; Del Pópolo, M. G. Designing and Understanding Permanent Microporosity in Liquids. *Phys. Chem. Chem. Phys.* **2014**, *16* (20), 9422–9431. https://doi.org/10.1039/c4cp00582a.

17. Imker, H. J.; Krahn, D.; Clerc, J.; Kaiser, M.; Walsh, C. T. N-Acylation during Glidobactin Biosynthesis by the Tridomain Nonribosomal Peptide Synthetase Module GlbF. *Chem. Biol.* **2010**, *17* (10), 1077–1083. https://doi.org/10.1016/j.chembiol.2010.08.007.

18. Zhang, X.; King-Smith, E.; Renata, H. Total Synthesis of Tambromycin by Combining Chemocatalytic and Biocatalytic C−H Functionalization. *Angew. Chemie - Int. Ed.* **2018**, *57* (18), 5037–5041. https://doi.org/10.1002/anie.201801165.

19. (a) Delaunay, D.; Toupet, L.; Le Corre, M. Reactivity of β-Amino Alcohols with Carbon Disulfide. Study on the Synthesis of 2-Oxazolidinethiones and 2-Thiazolidinethiones. *J. Org. Chem.* **1995**, *60* (20), 6604–6607. https://doi.org/10.1021/jo00125a059. (b) Franck, X.; Langlois, E.; Outurquin, F. Diastereoselective Aldolization with N-Phenylselanylacetyl Derivatives. *Synthesis (Stuttg).* **2007**, *4* (5), 719–724. https://doi.org/10.1055/s-2007-965894.

20. Patel, J.; Clavé, G.; Renard, P.-Y.; Franck, X. Straightforward Access to Protected Syn α-Amino-β-Hydroxy Acid Derivatives. *Angew. Chemie* **2008**, *120* (22), 4292–4295. https://doi.org/10.1002/ange.200800860.

21. Sakai, A.; Xiang, D. F.; Xu, C.; Song, L.; Yew, W. S.; Raushel, F. M.; Gerlt, J. A. Evolution of Enzymatic Activities in the Enolase Superfamily: N-Succinylamino Acid Racemase and a New Pathway for the Irreversible Conversion of D- to L-Amino Acids. *Biochemistry* **2006**, *45* (14), 4455–4462. https://doi.org/10.1021/bi060230b.

22. Hibi, M.; Kasahara, T.; Kawashima, T.; Yajima, H.; Kozono, S.; Smirnov, S. V.; Kodera, T.; Sugiyama, M.; Shimizu, S.; Yokozeki, K.; et al. Multi-Enzymatic Synthesis of Optically Pure β-Hydroxy α-Amino Acids. *Adv. Synth. Catal.* **2015**, *357* (4), 767–774. https://doi.org/10.1002/adsc.201400672.

23. Prechter, A.; Heinrich, M. R. Hydrogen Peroxide and Arenediazonium Salts as Reagents for a Radical Beckmann-Type Rearrangement. *Synthesis (Stuttg).* **2011**, No. 10, 1515–1525. https://doi.org/10.1055/s-0030-1260006.

24. Pitre, S. P.; McTiernan, C. D.; Ismaili, H.; Scaiano, J. C. Metal-Free Photocatalytic Radical Trifluoromethylation Utilizing Methylene Blue and Visible Light Irradiation. *ACS Catal.* **2014**, *4* (8), 2530–2535. https://doi.org/10.1021/cs5005823.

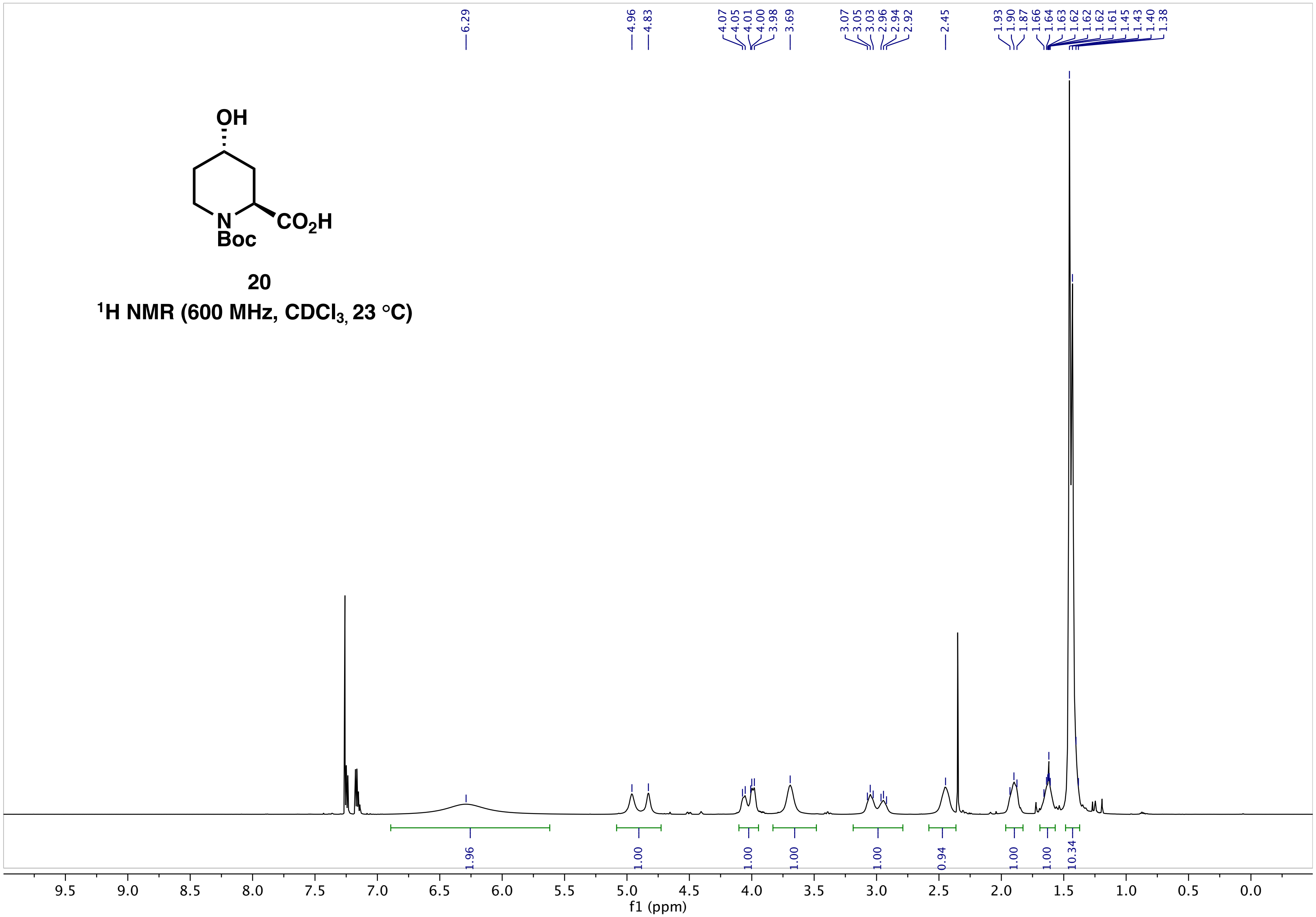

OH
N
Boc
CO2H
20
1H NMR (600 MHz, CDCl3, 23 °C)
6.29
4.96
4.83
4.07
4.05
4.01
4.00
3.98
3.69
3.07
3.05
3.03
2.96
2.94
2.92
2.45
1.93
1.90
1.87
1.66
1.64
1.63
1.62
1.62
1.61
1.45
1.43
1.40
1.38
1.96
1.00
1.00
1.00
1.00
0.94
1.00
1.00
10.34
9.5
9.0
8.5
8.0
7.5
7.0
6.5
6.0
5.5
5.0
4.5
4.0
3.5
3.0
2.5
2.0
1.5
1.0
0.5
0.0
f1 (ppm)

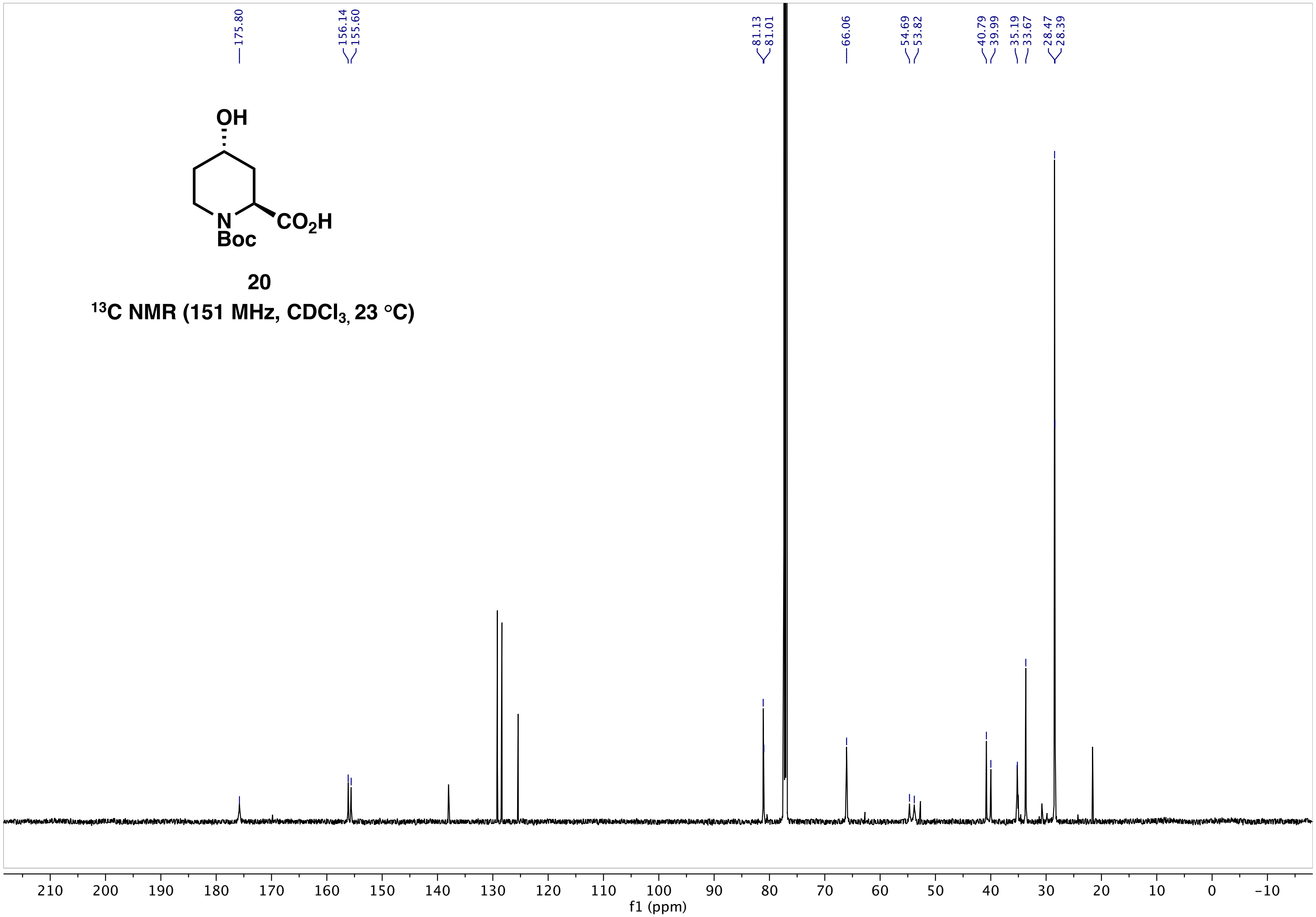
175.80
156.14
155.60
81.13
81.01
66.06
54.69
53.82
40.79
39.99
35.19
33.67
28.47
28.39
OH
N
Boc
CO2H
20
13C NMR (151 MHz, CDCl3, 23 °C)
210
200
190
180
170
160
150
140
130
120
110
100
90
80
70
60
50
40
30
20
10
0
−10
f1 (ppm)

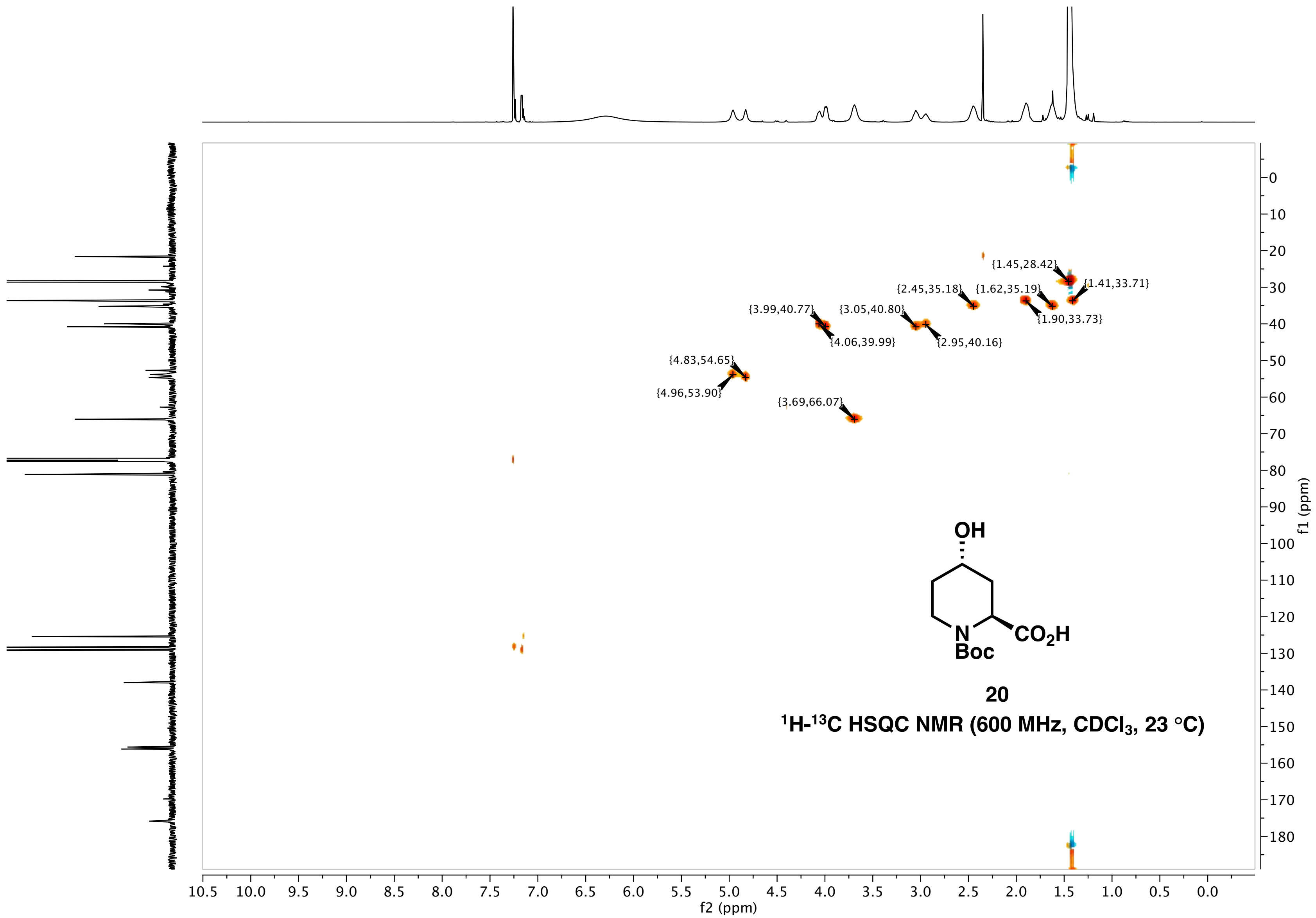

20

^{1}H-^{13}C HSQC NMR (600 MHz, $CDCl_3$, 23 °C)

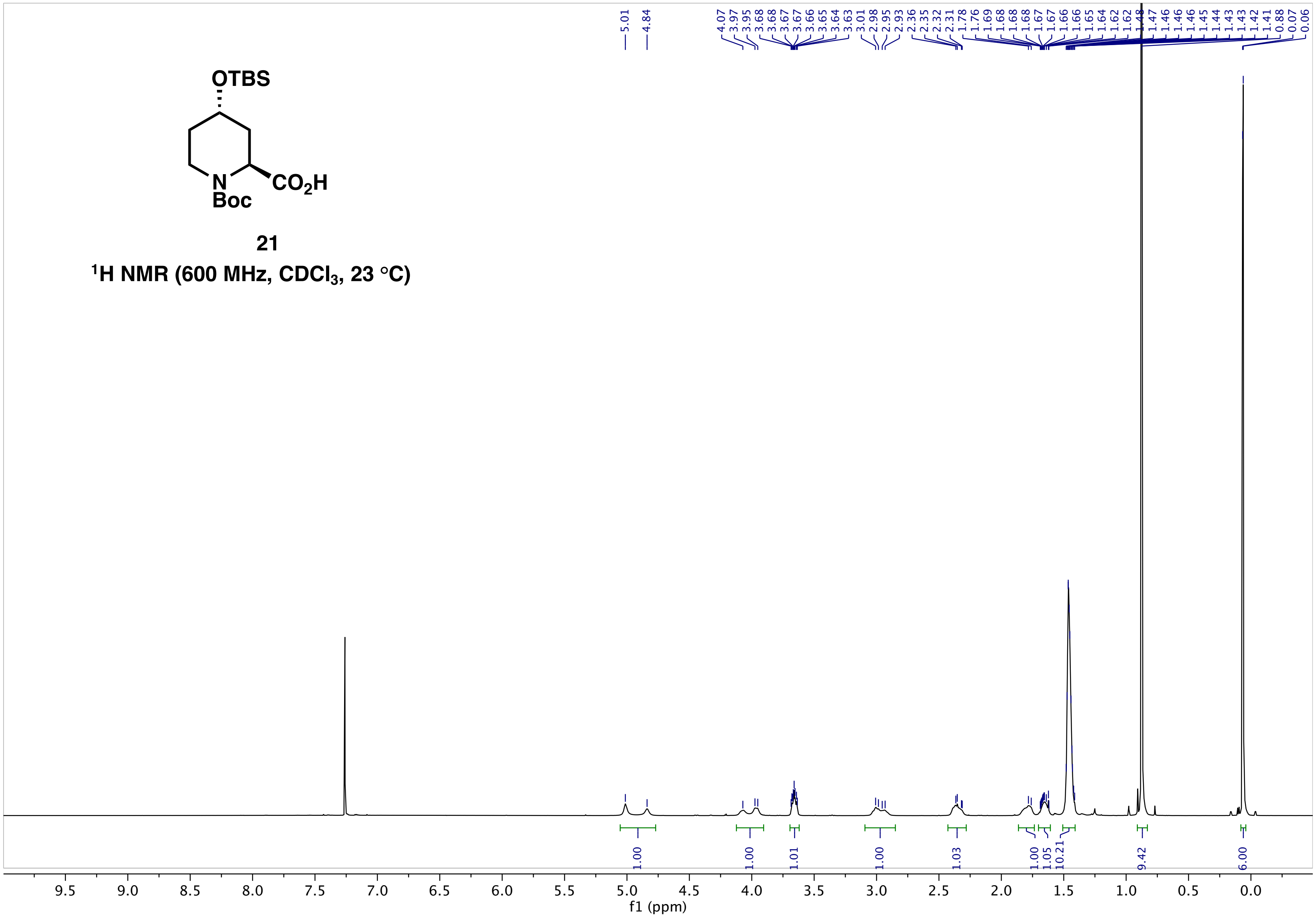

OTBS
N
Boc
CO2H
21
1H NMR (600 MHz, CDCl3, 23 °C)
f1 (ppm)

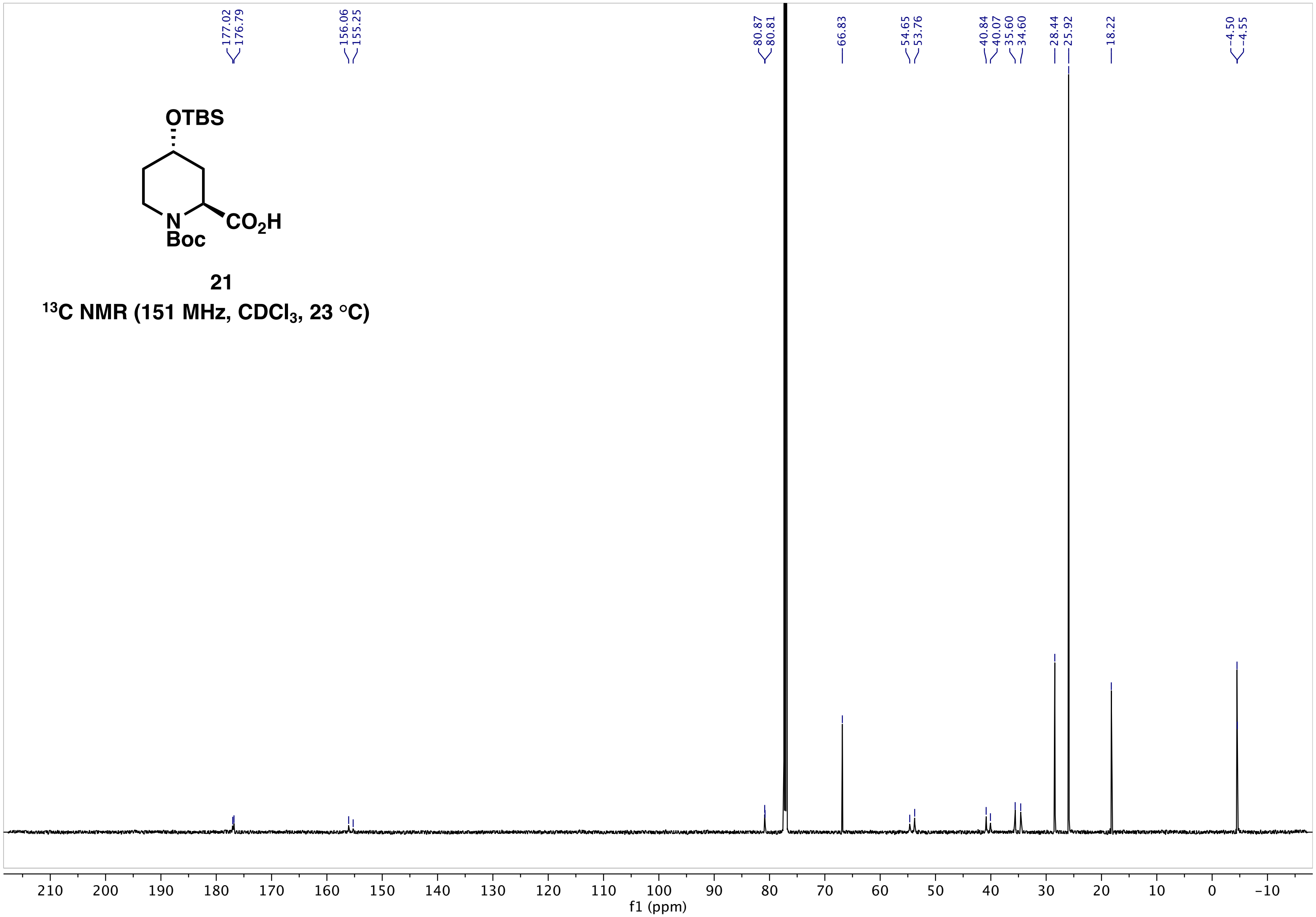
177.02
176.79
156.06
155.25
80.87
80.81
66.83
54.65
53.76
40.84
40.07
35.60
34.60
28.44
25.92
18.22
-4.50
-4.55
OTBS
N
Boc
CO_2H
21
^{13}C NMR (151 MHz, $CDCl_3$, 23 °C)
210
200
190
180
170
160
150
140
130
120
110
100
90
80
70
60
50
40
30
20
10
0
–10
f1 (ppm)

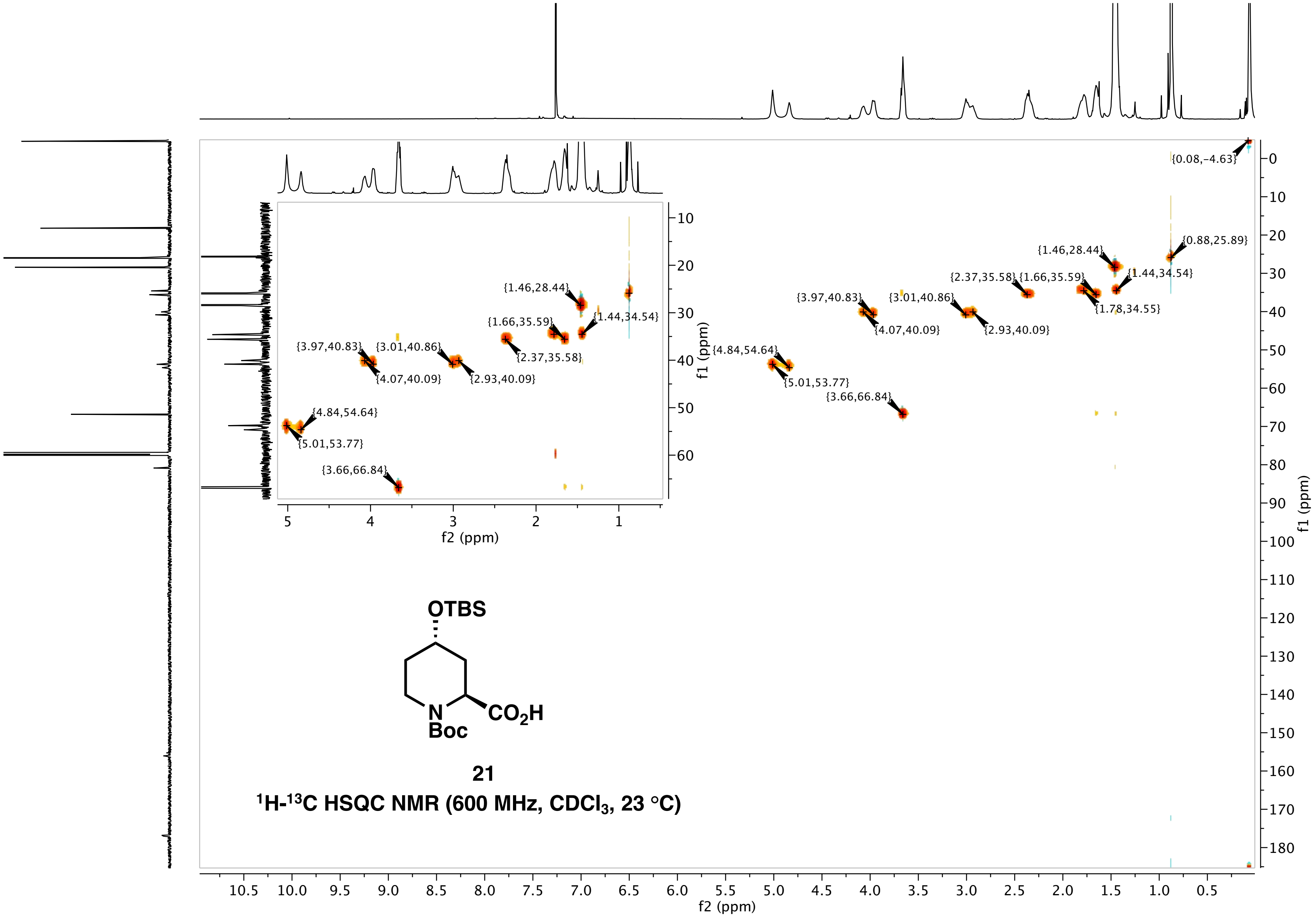

21

^{1}H-^{13}C HSQC NMR (600 MHz, $CDCl_3$, 23 °C)

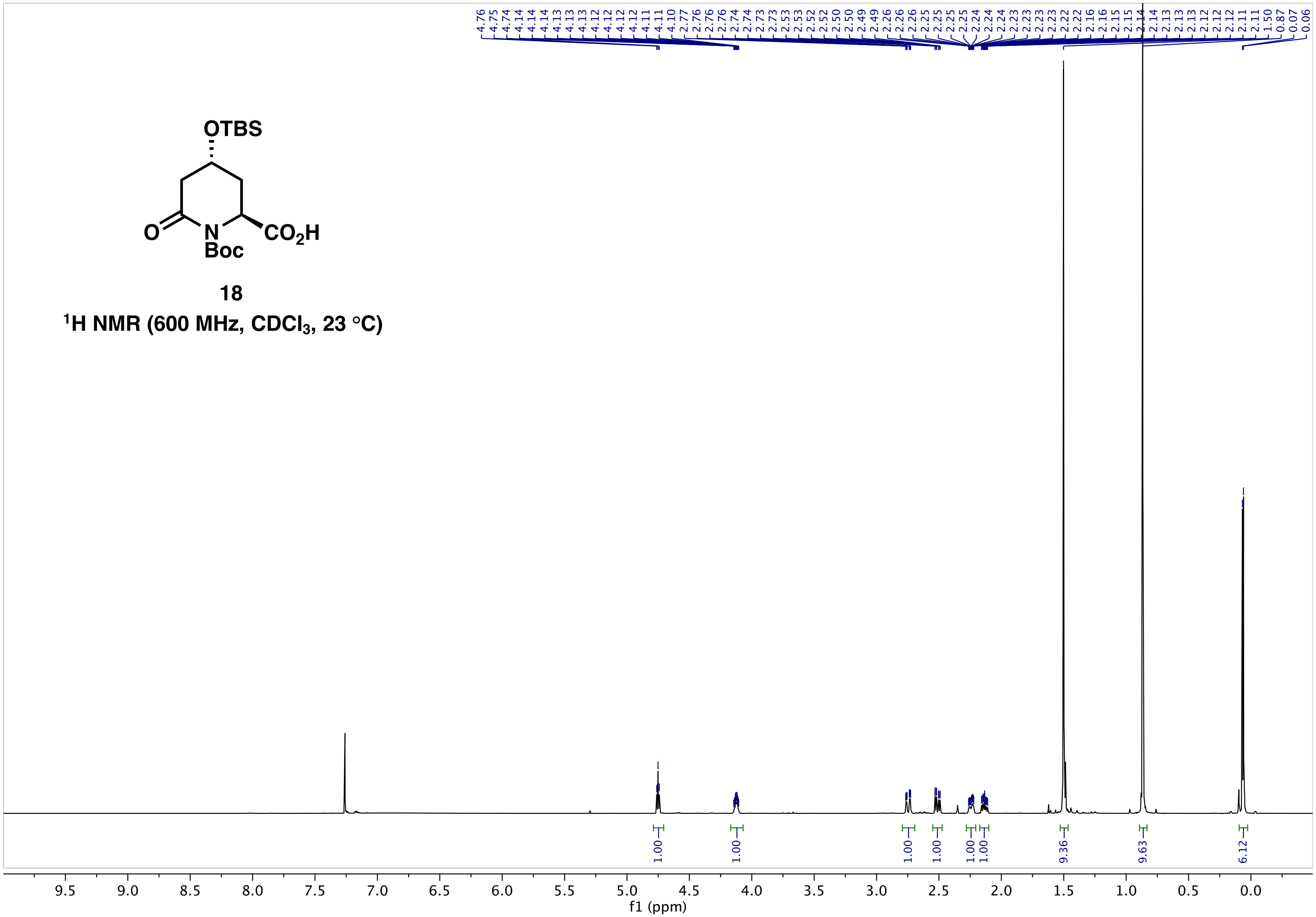

18

^1H NMR (600 MHz, $CDCl_3$, 23 °C)

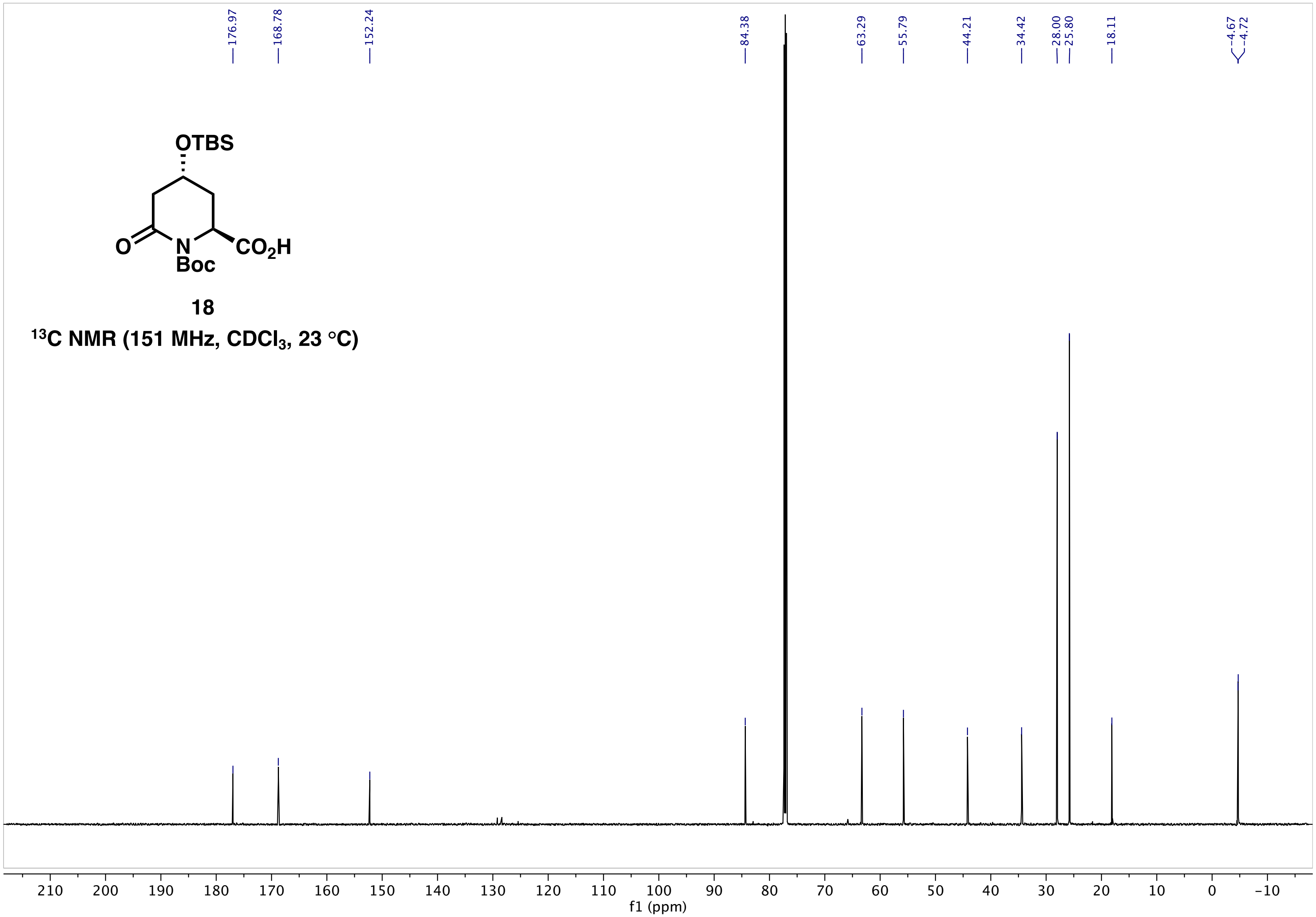
176.97
168.78
152.24
84.38
63.29
55.79
44.21
34.42
28.00
25.80
18.11
-4.67
-4.72
OTBS
O
N
Boc
CO2H
18
13C NMR (151 MHz, CDCl3, 23 °C)
210 200 190 180 170 160 150 140 130 120 110 100 90 80 70 60 50 40 30 20 10 0 -10
f1 (ppm)

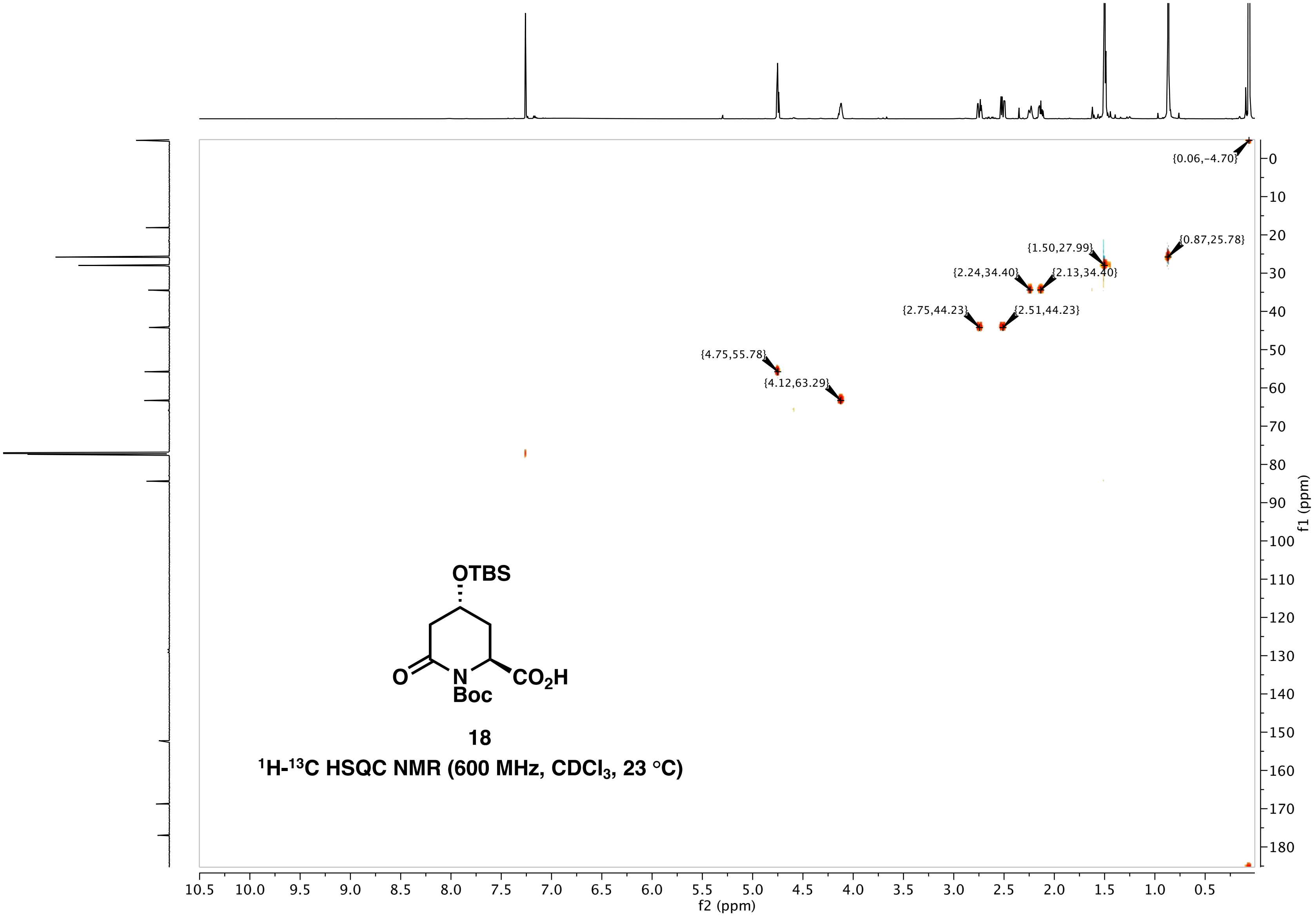

18

^{1}H-^{13}C HSQC NMR (600 MHz, $CDCl_3$, 23 °C)

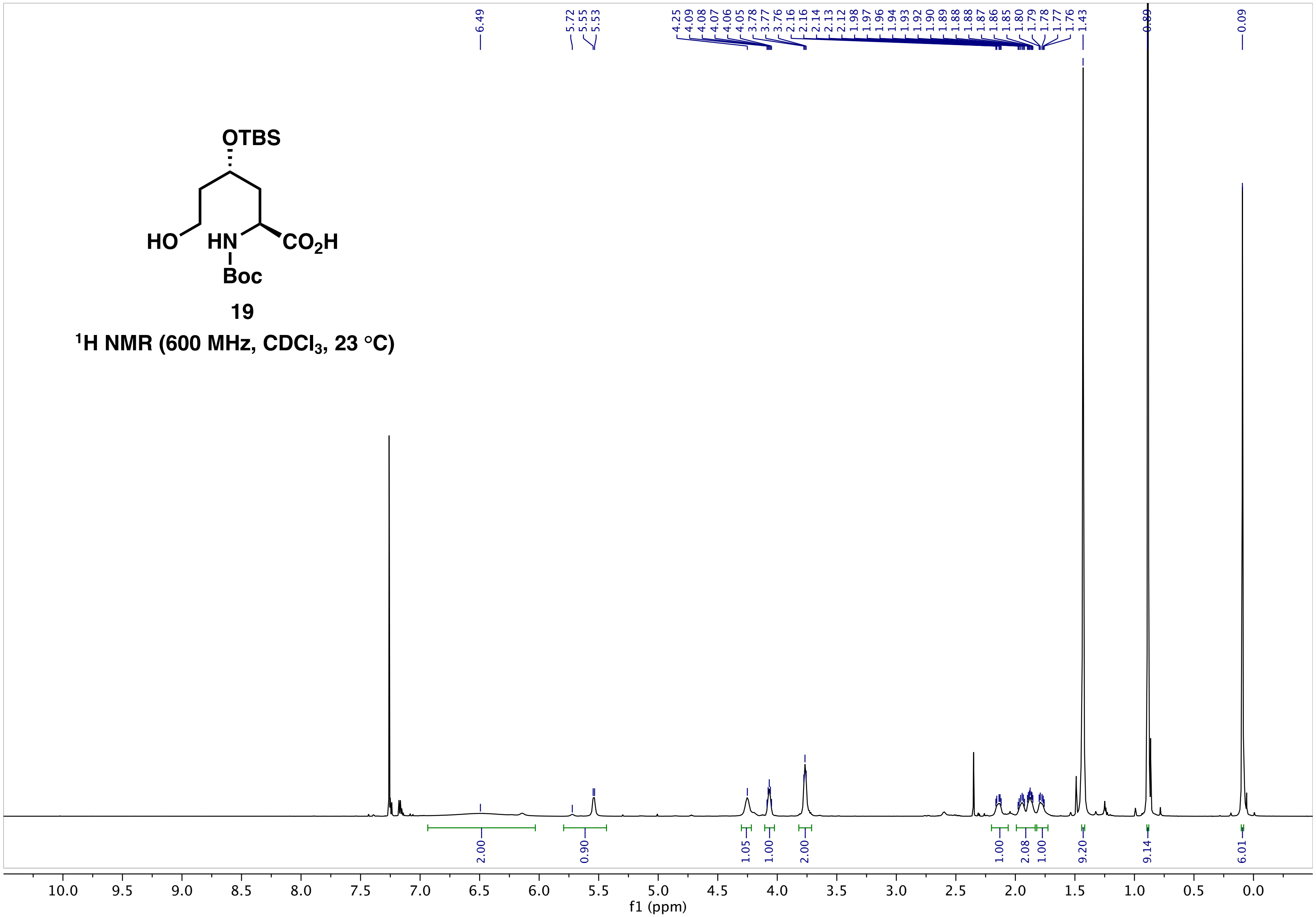
OTBS
HO
HN
Boc
CO_2H
19
^1H NMR (600 MHz, $CDCl_3$, 23 °C)
f1 (ppm)

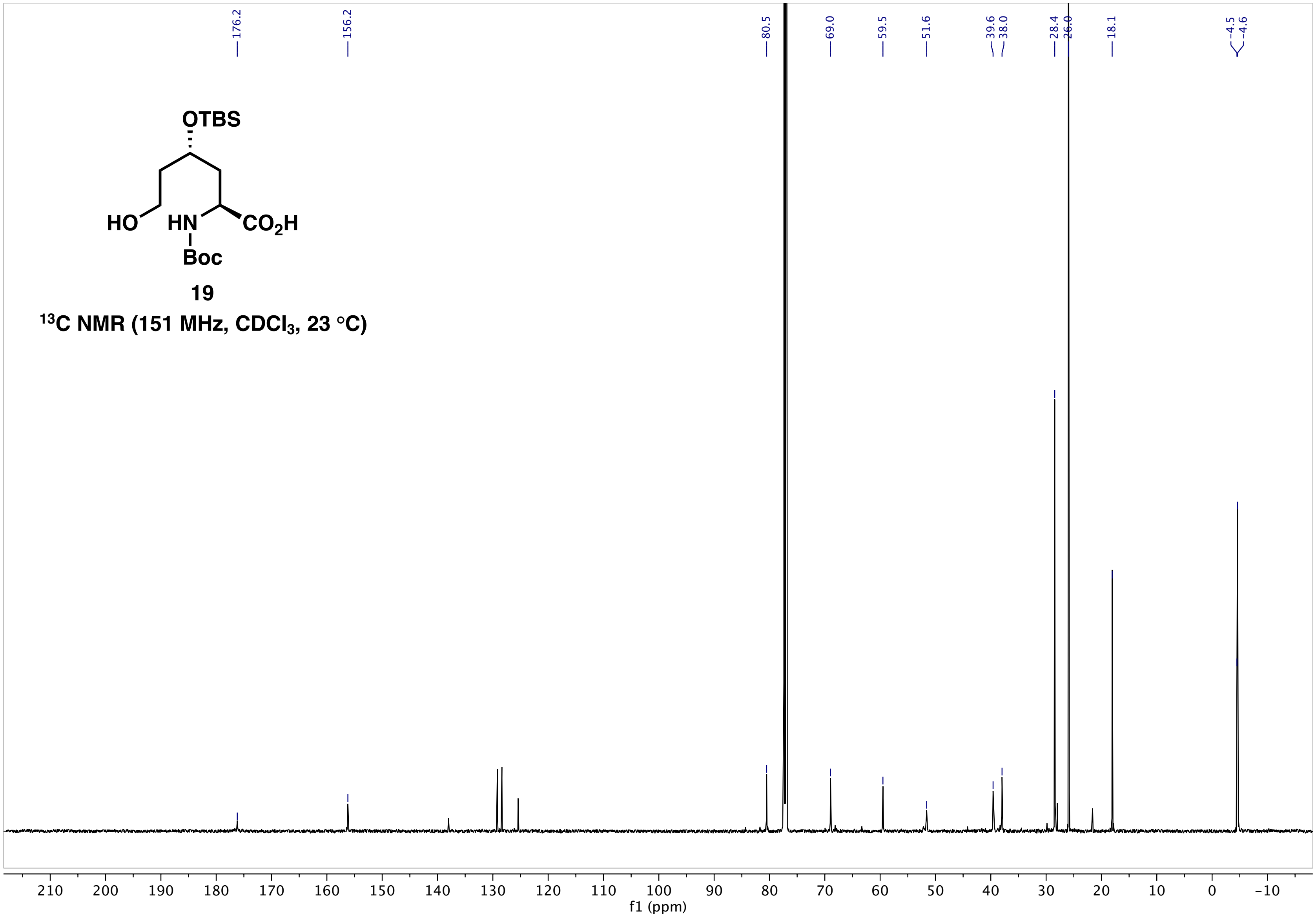

176.2
156.2
80.5
69.0
59.5
51.6
39.6
38.0
28.4
26.0
18.1
-4.5
-4.6
OTBS
HO
HN
CO2H
Boc
19
13C NMR (151 MHz, CDCl3, 23 °C)
210
200
190
180
170
160
150
140
130
120
110
100
90
80
70
60
50
40
30
20
10
0
-10
f1 (ppm)

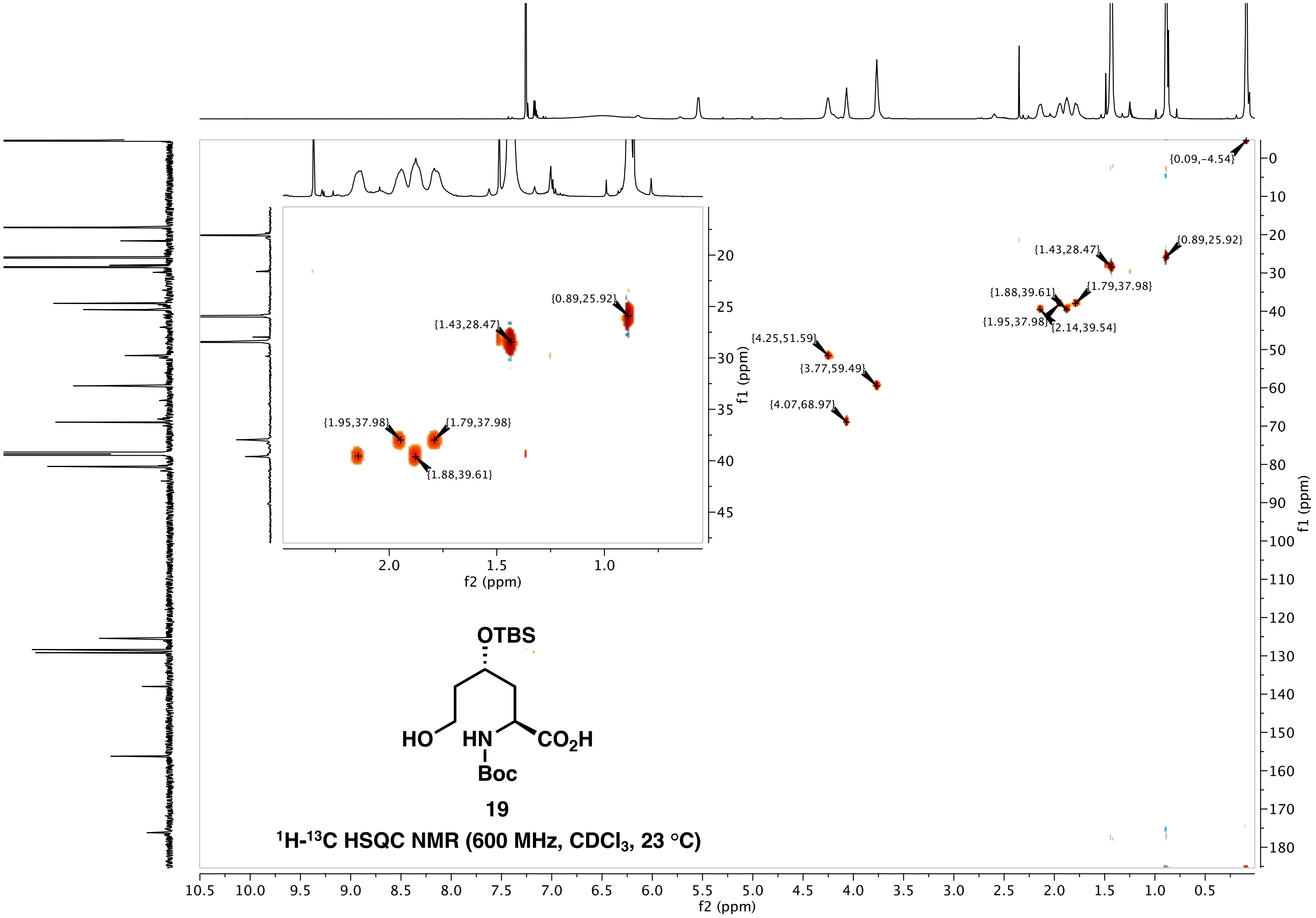

19

^1H-^{13}C HSQC NMR (600 MHz, $CDCl_3$, 23 °C)

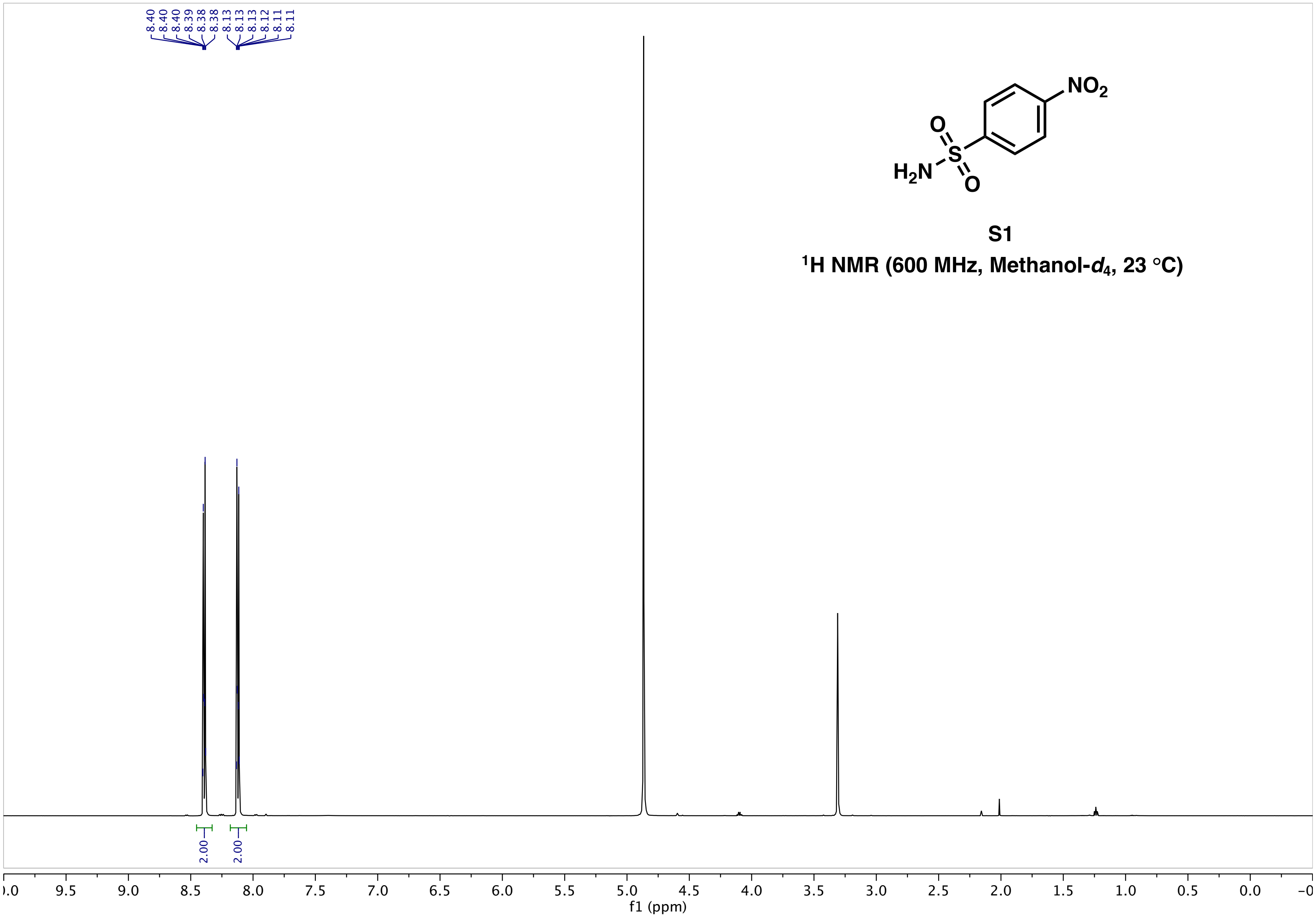

8.40
8.40
8.40
8.39
8.38
8.38
8.38
8.13
8.13
8.13
8.12
8.11
8.11
NO2
O
S
H2N
O
S1
1H NMR (600 MHz, Methanol-d4, 23 °C)
2.00
2.00
9.5
9.0
8.5
8.0
7.5
7.0
6.5
6.0
5.5
5.0
4.5
4.0
3.5
3.0
2.5
2.0
1.5
1.0
0.5
0.0
f1 (ppm)

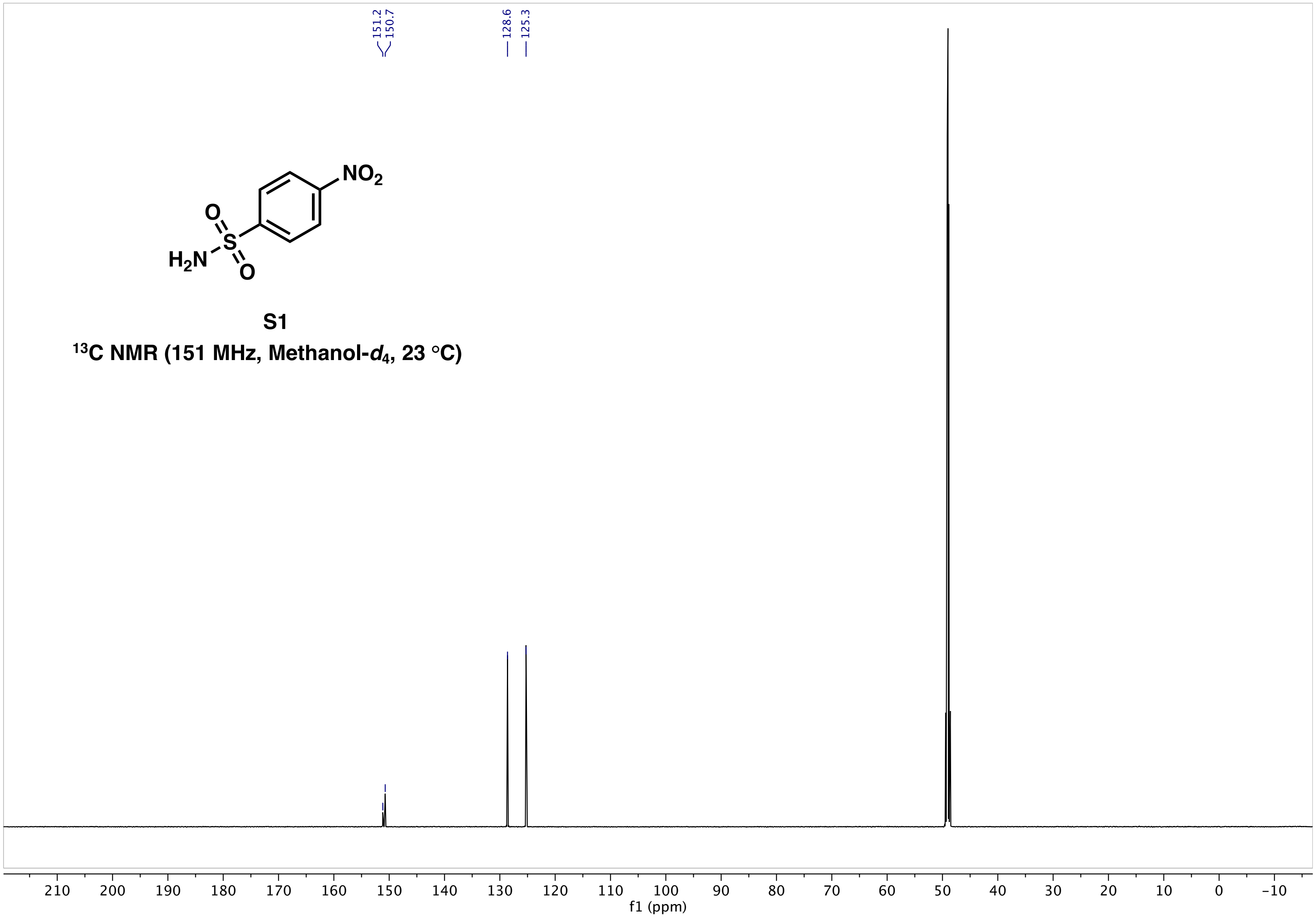
151.2
150.7
128.6
125.3
NO_2
O
S
H_2N
O
S1
^{13}C NMR (151 MHz, Methanol-d_4, 23 °C)
210 200 190 180 170 160 150 140 130 120 110 100 90 80 70 60 50 40 30 20 10 0 −10
f1 (ppm)

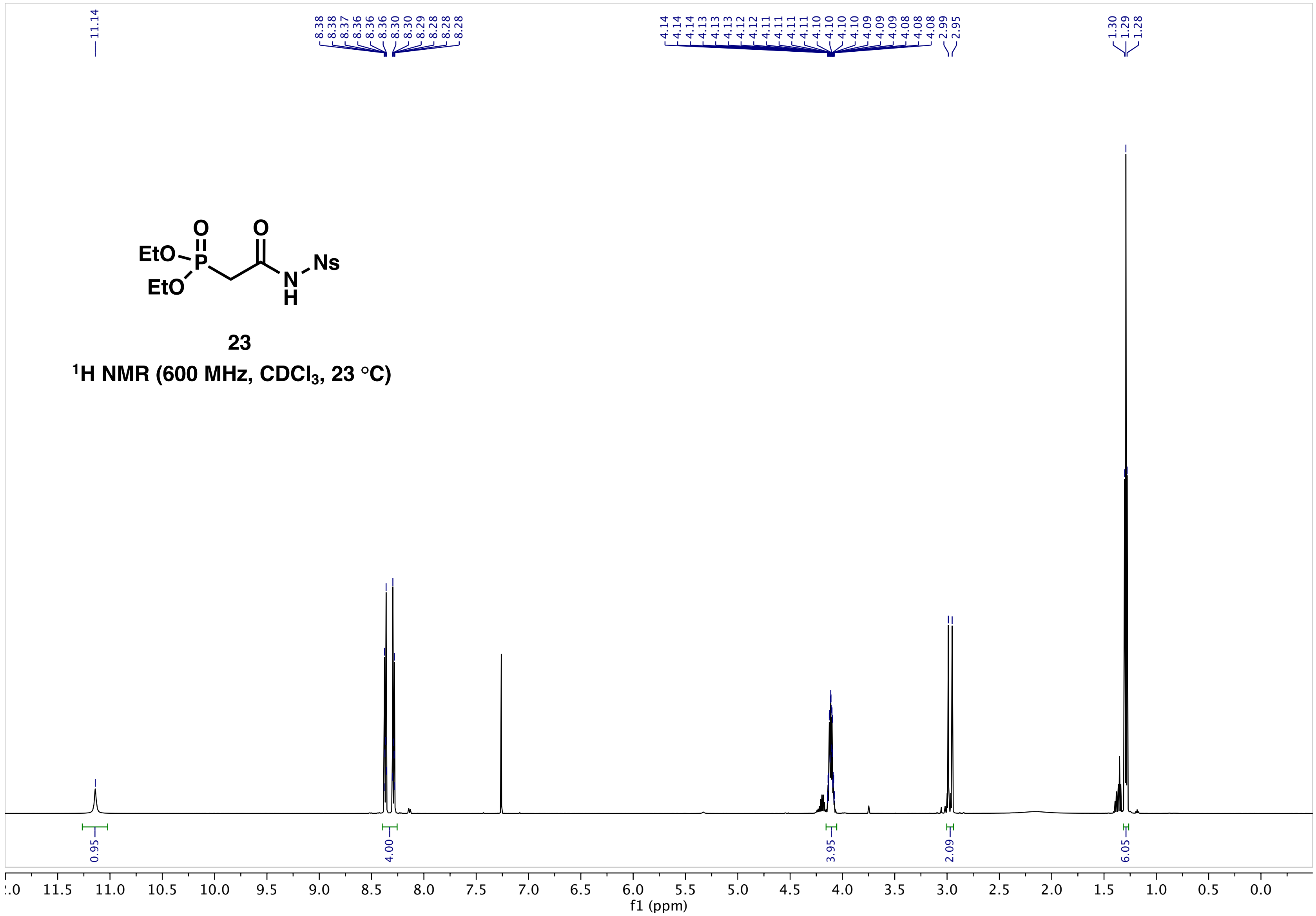

11.14
8.38 8.38 8.37 8.36 8.36 8.30 8.30 8.29 8.28 8.28
4.14 4.14 4.14 4.13 4.13 4.13 4.12 4.12 4.11 4.11 4.11 4.11 4.10 4.10 4.10 4.10 4.09 4.09 4.08 4.08 4.08 2.99 2.95
1.30 1.29 1.28
EtO
EtO
P
O
O
N
H
Ns
23
1H NMR (600 MHz, CDCl3, 23 °C)
0.95
4.00
3.95
2.09
6.05
f1 (ppm)

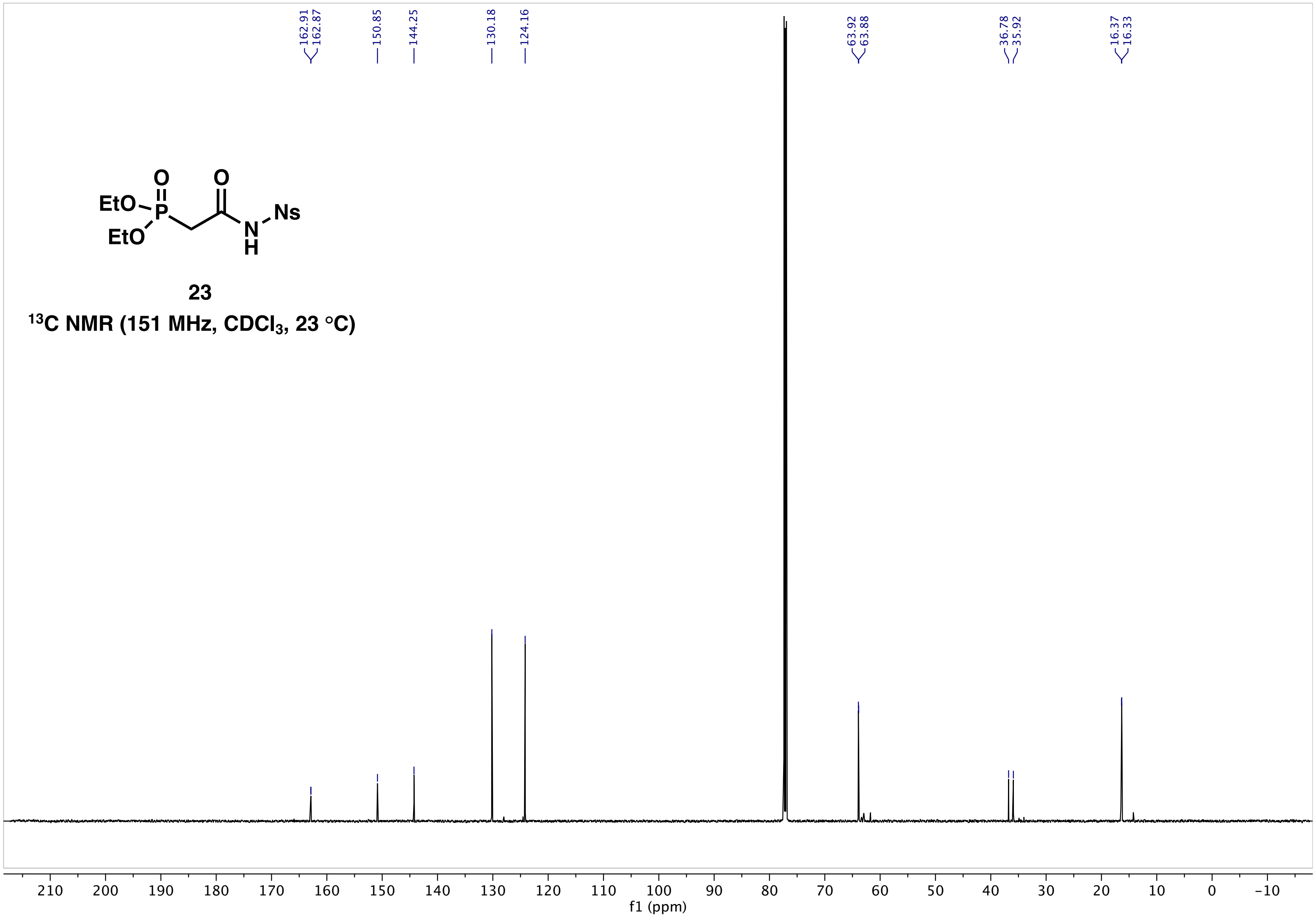

23

^{13}C NMR (151 MHz, $CDCl_3$, 23 °C)

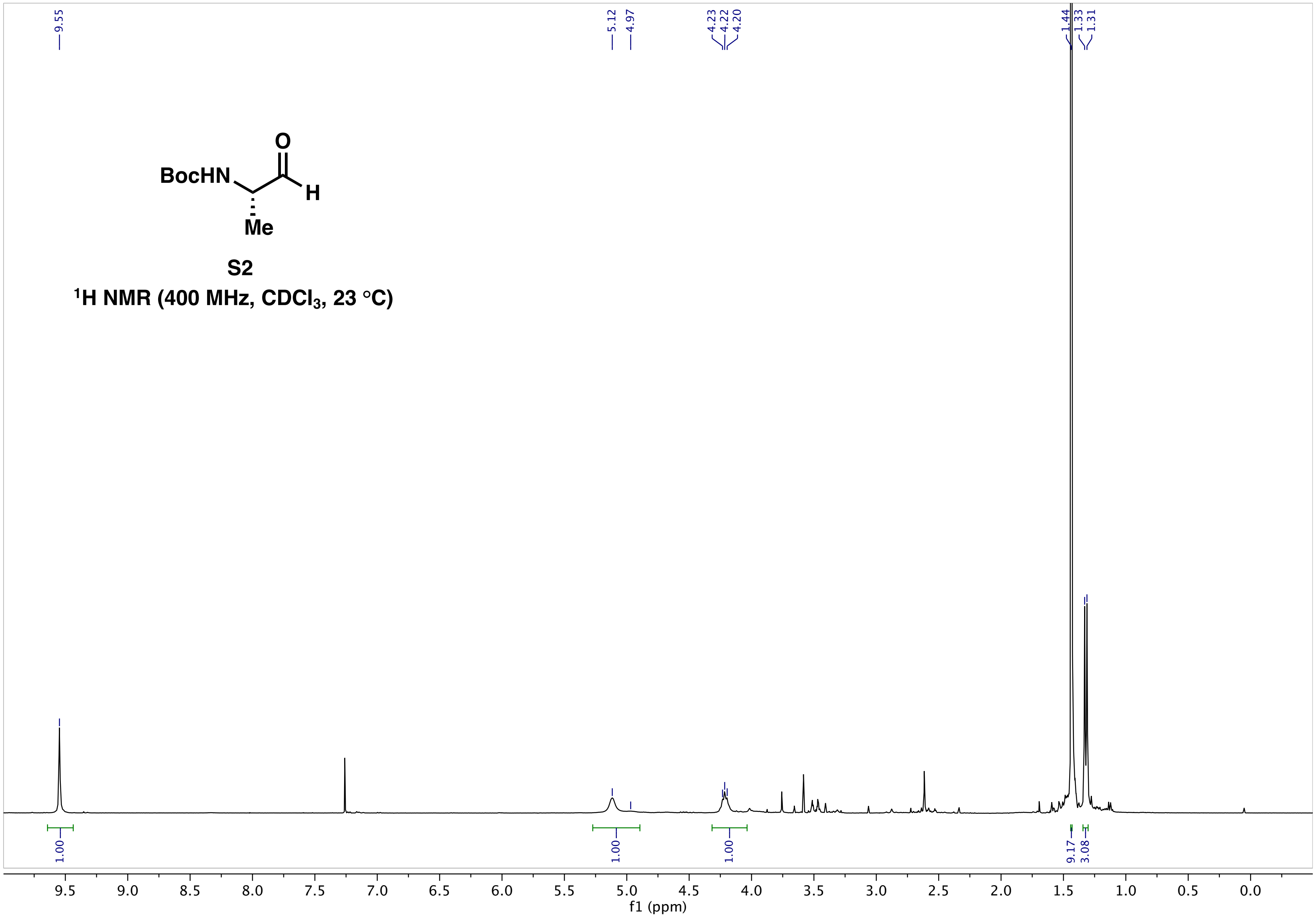

BocHN
O
H
Me
S2
1H NMR (400 MHz, CDCl3, 23 °C)
9.55
5.12
4.97
4.23
4.22
4.20
1.44
1.33
1.31
1.00
1.00
1.00
9.17
3.08
9.5
9.0
8.5
8.0
7.5
7.0
6.5
6.0
5.5
5.0
4.5
4.0
3.5
3.0
2.5
2.0
1.5
1.0
0.5
0.0
f1 (ppm)

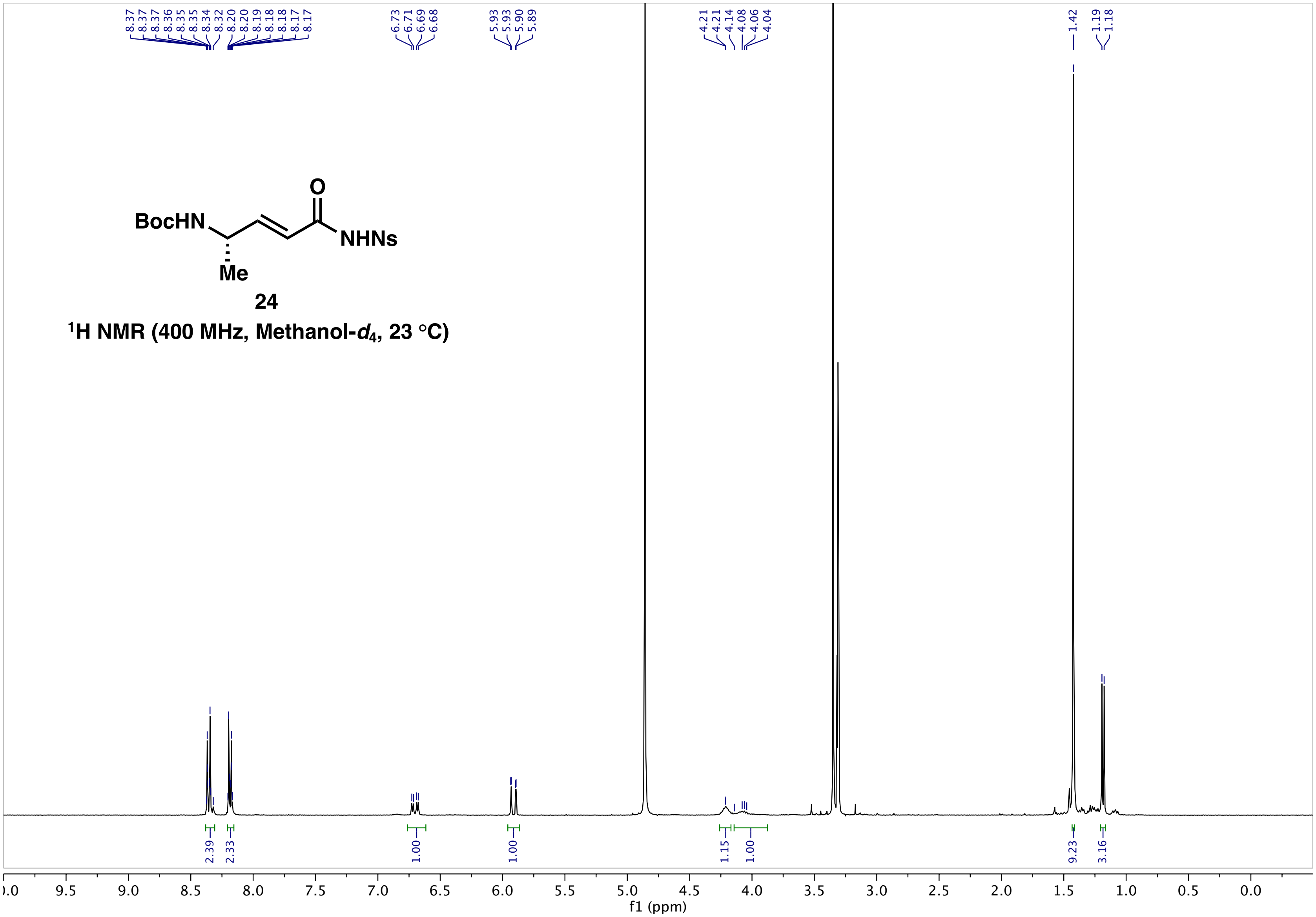
BocHN
NHNs
O
Me
24
^{1}H NMR (400 MHz, Methanol-d_4, 23 °C)
f1 (ppm)

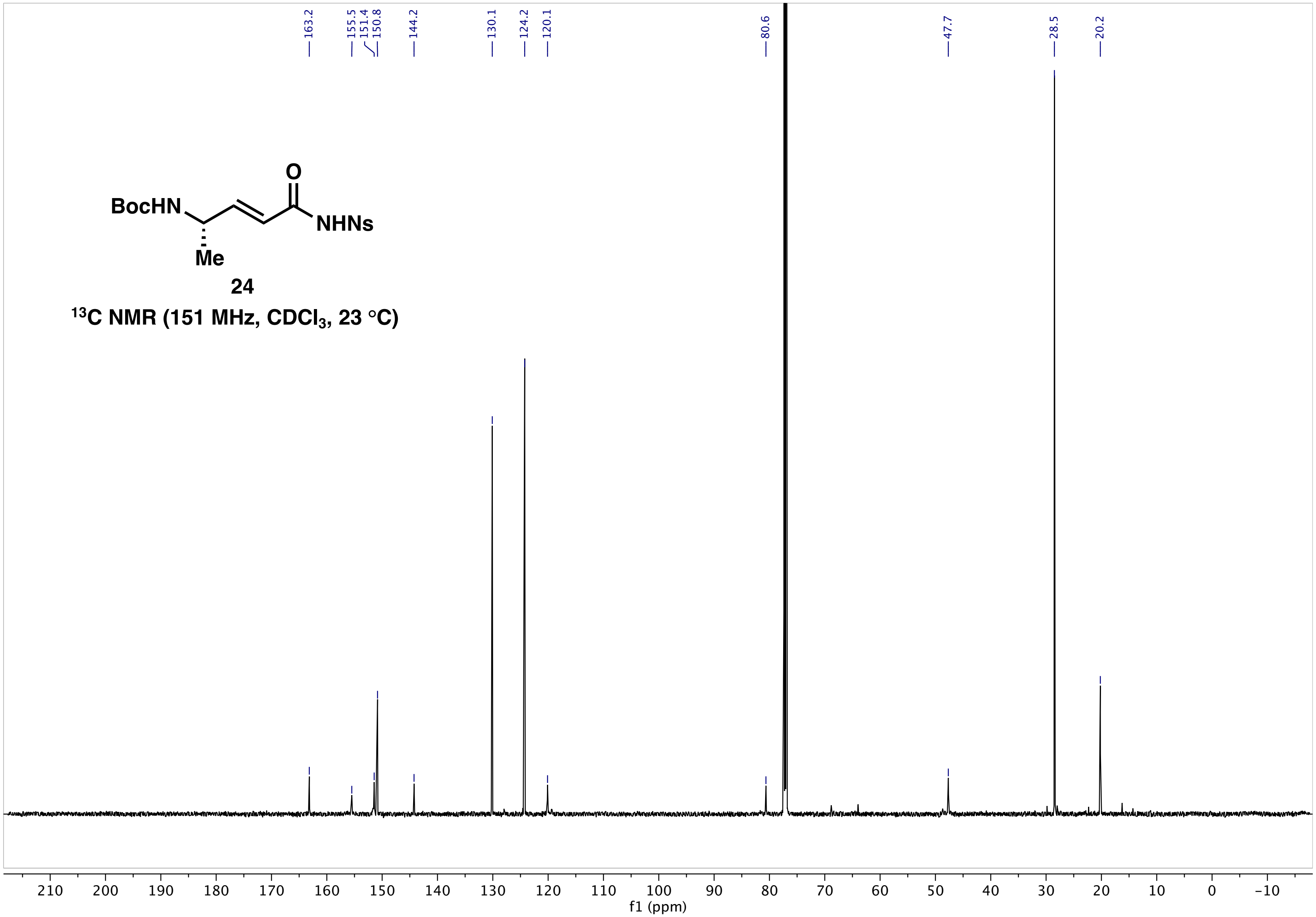
163.2
155.5
151.4
150.8
144.2
130.1
124.2
120.1
80.6
47.7
28.5
20.2
BocHN
NHNs
O
Me
24
^{13}C NMR (151 MHz, $CDCl_3$, 23 °C)
210
200
190
180
170
160
150
140
130
120
110
100
90
80
70
60
50
40
30
20
10
0
−10
f1 (ppm)

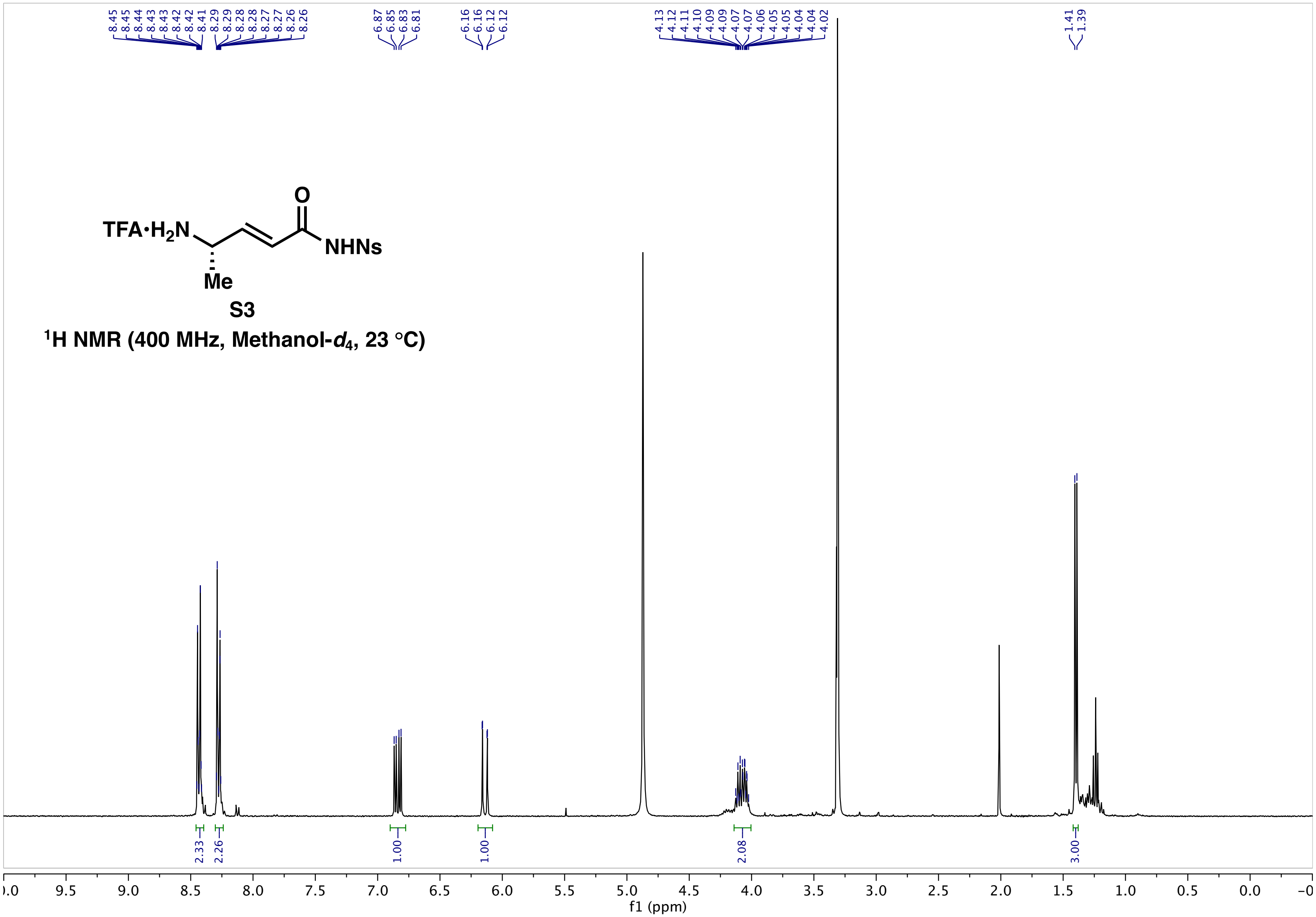

TFA·H2N
NHNs
O
Me
S3
1H NMR (400 MHz, Methanol-d4, 23 °C)
8.45 8.45 8.44 8.43 8.43 8.42 8.42 8.41 8.29 8.29 8.28 8.28 8.27 8.27 8.26 8.26
6.87 6.85 6.83 6.81
6.16 6.16 6.12 6.12
4.13 4.12 4.11 4.10 4.09 4.09 4.07 4.07 4.06 4.05 4.05 4.04 4.04 4.02
1.41 1.39
2.33 2.26 1.00 1.00 2.08 3.00
f1 (ppm)

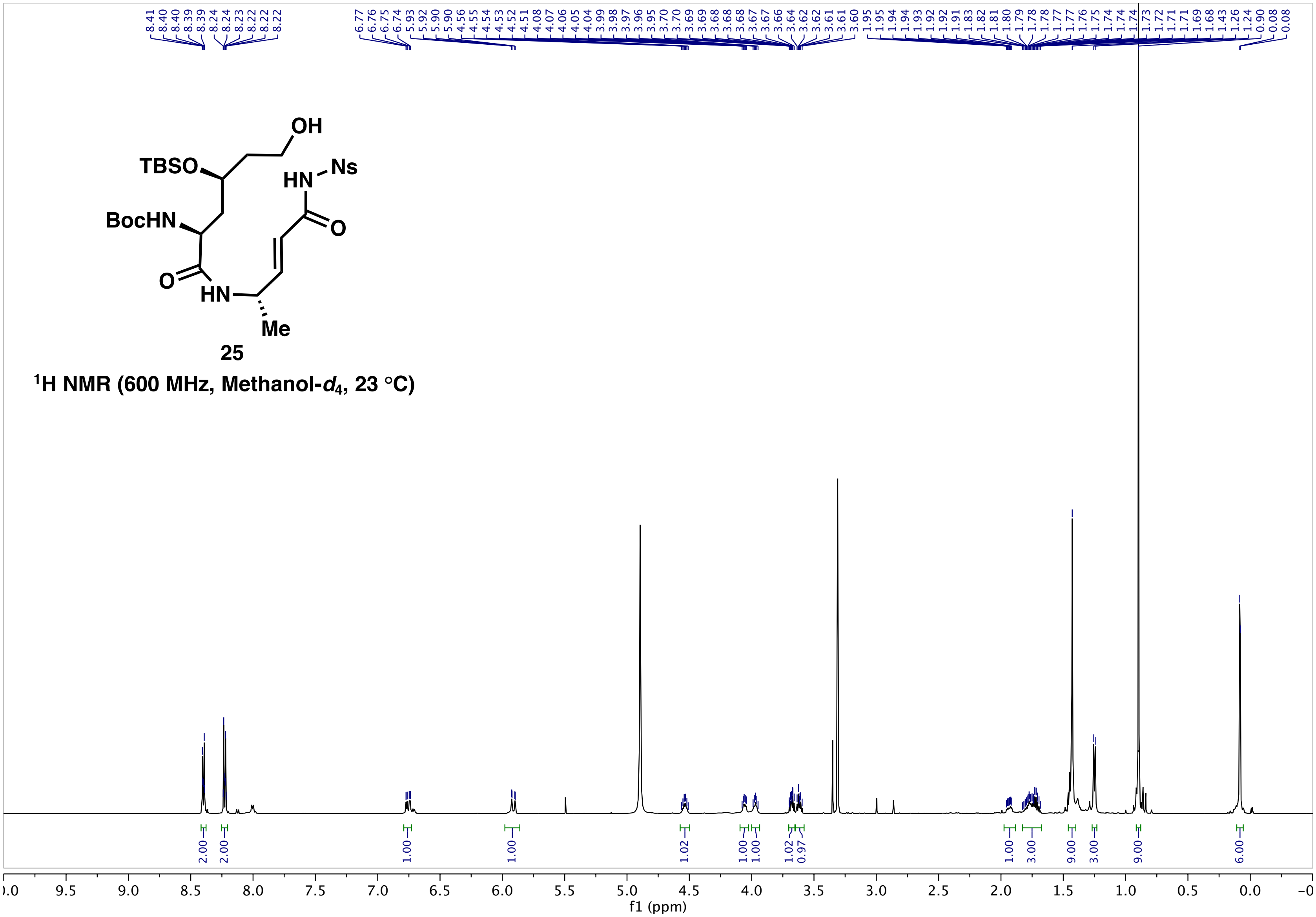

OH
TBSO
HN
Ns
BocHN
O
O
HN
Me
25
1H NMR (600 MHz, Methanol-d4, 23 °C)
f1 (ppm)

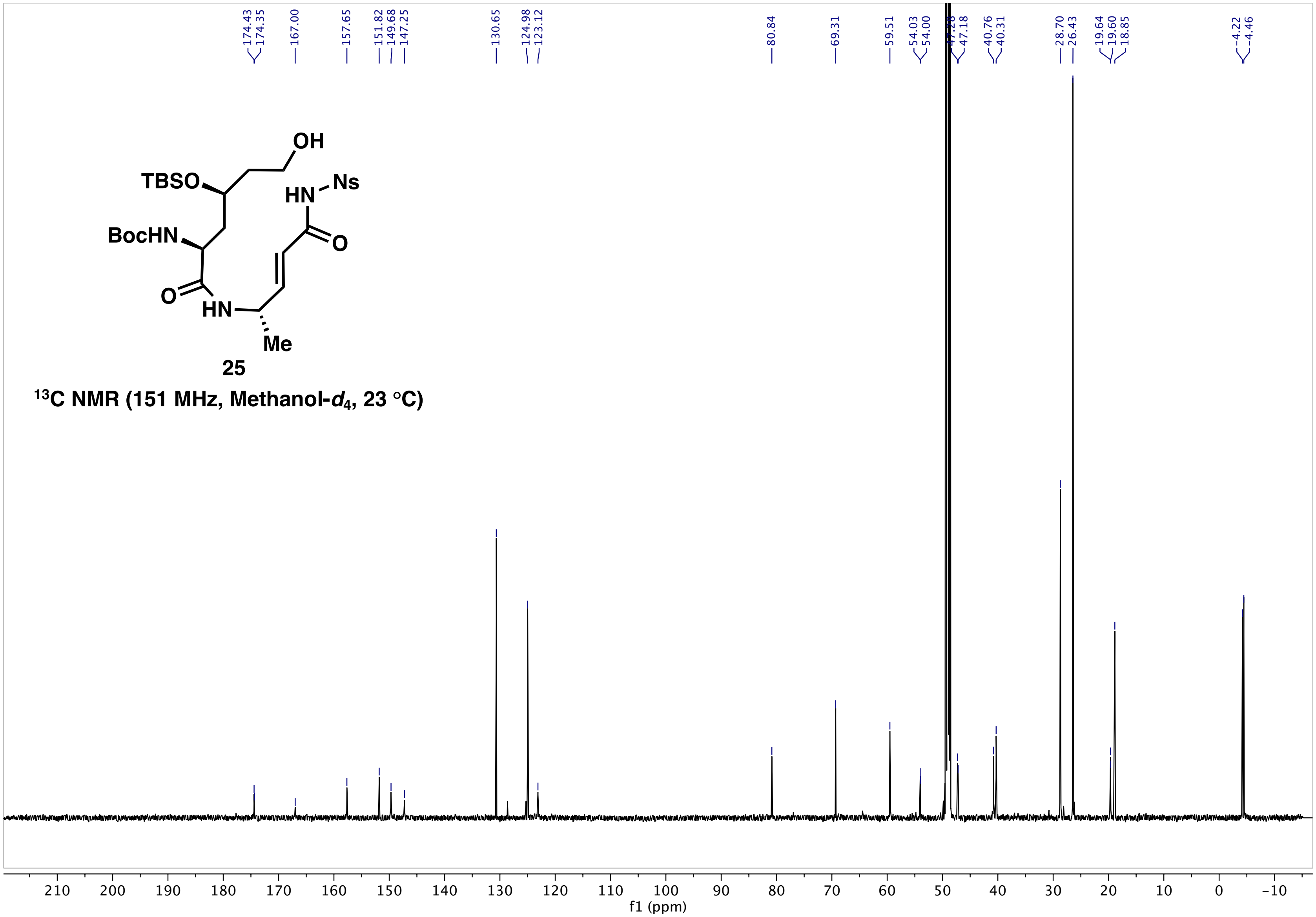

^{13}C NMR (151 MHz, Methanol-d_4, 23 °C)

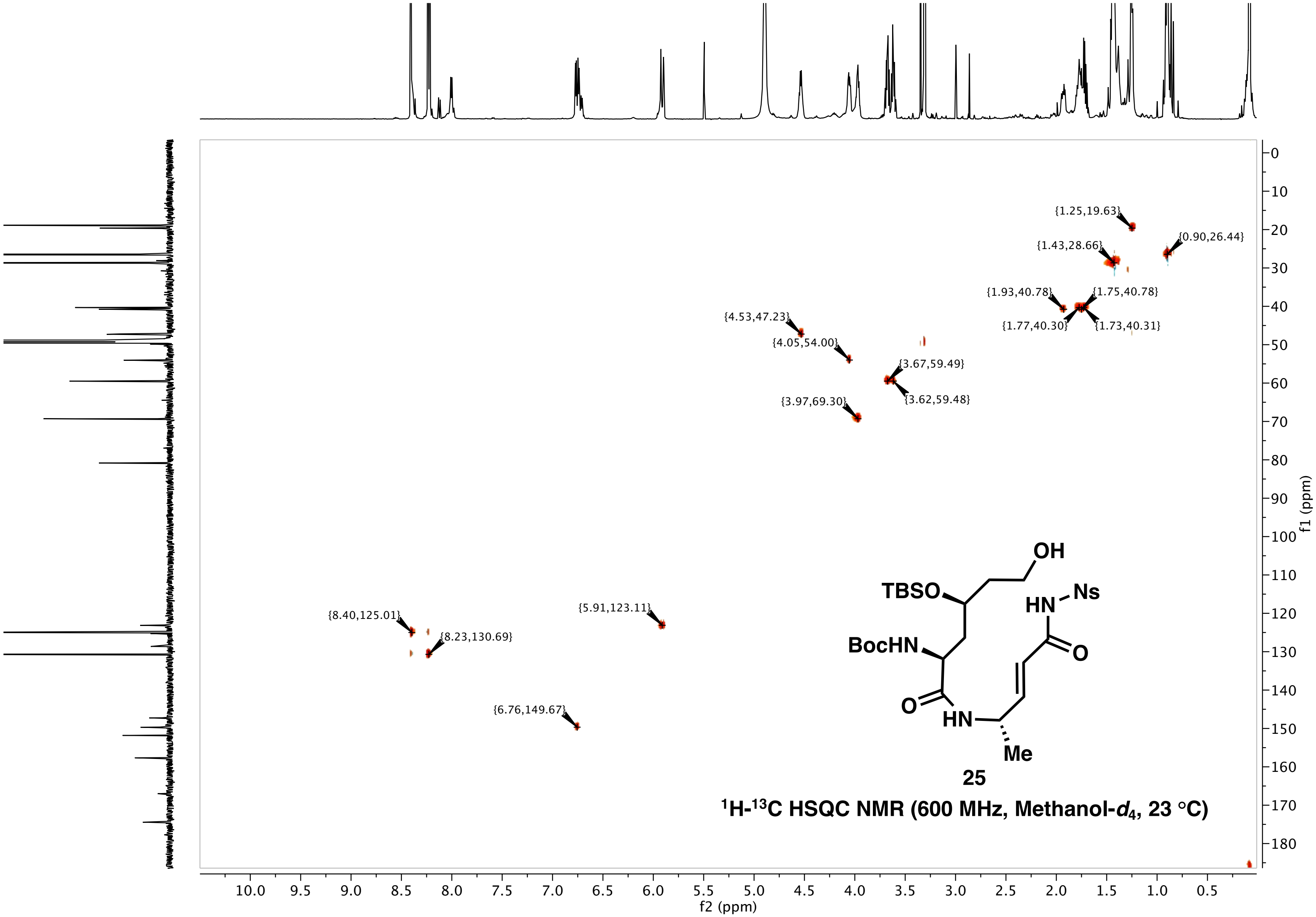

25

^1H-^{13}C HSQC NMR (600 MHz, Methanol-d_4, 23 °C)

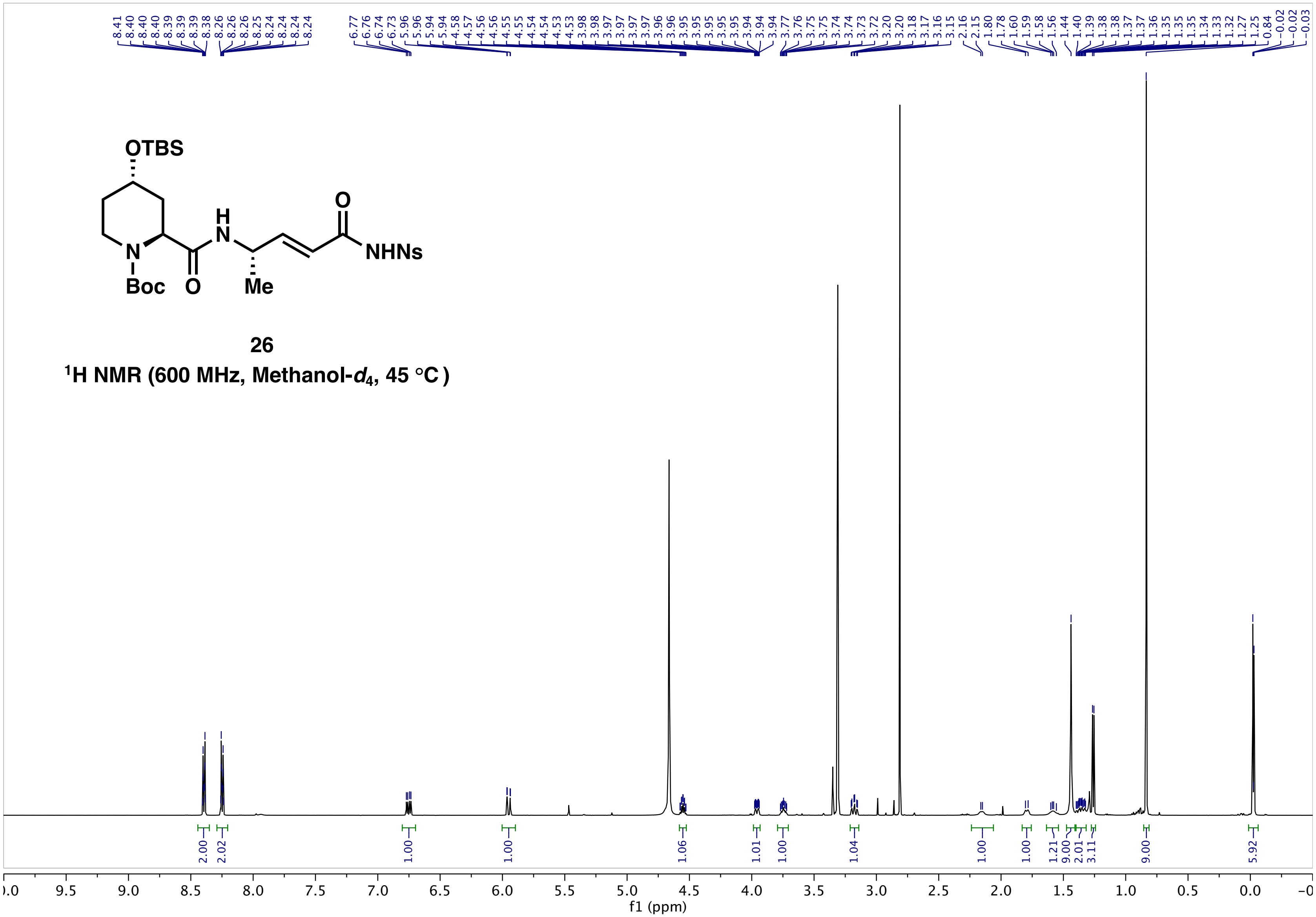

26

^{1}H NMR (600 MHz, Methanol-d_4, 45 °C)

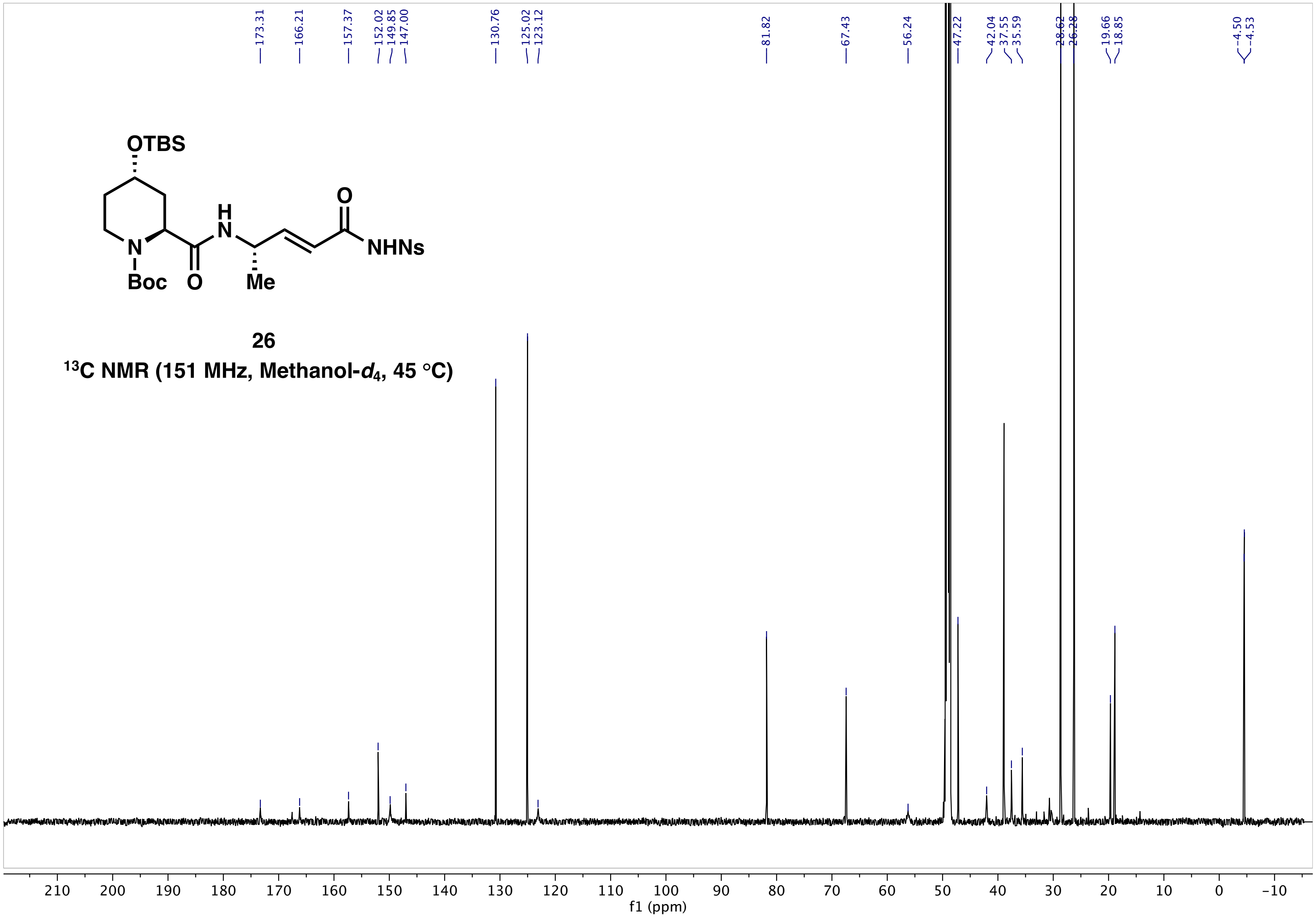

173.31
166.21
157.37
152.02
149.85
147.00
130.76
125.02
123.12
81.82
67.43
56.24
47.22
42.04
37.55
35.59
28.62
26.28
19.66
18.85
-4.50
-4.53
OTBS
H
N
O
NHNs
N
Boc
O
Me
26
^{13}C NMR (151 MHz, Methanol-d_4, 45 °C)
210
200
190
180
170
160
150
140
130
120
110
100
90
80
70
60
50
40
30
20
10
0
−10
f1 (ppm)

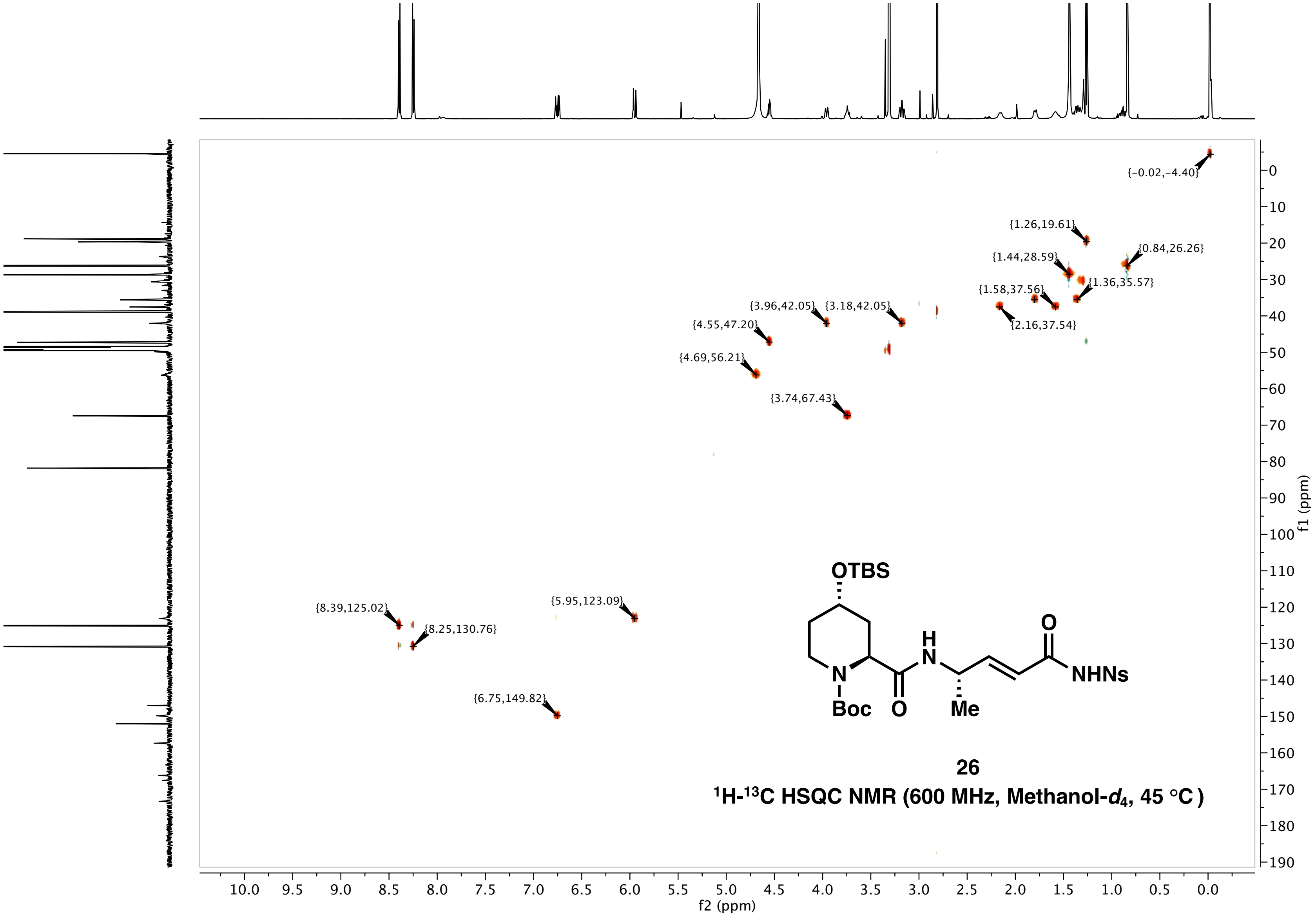

26

^{1}H-^{13}C HSQC NMR (600 MHz, Methanol-d_4, 45 °C)

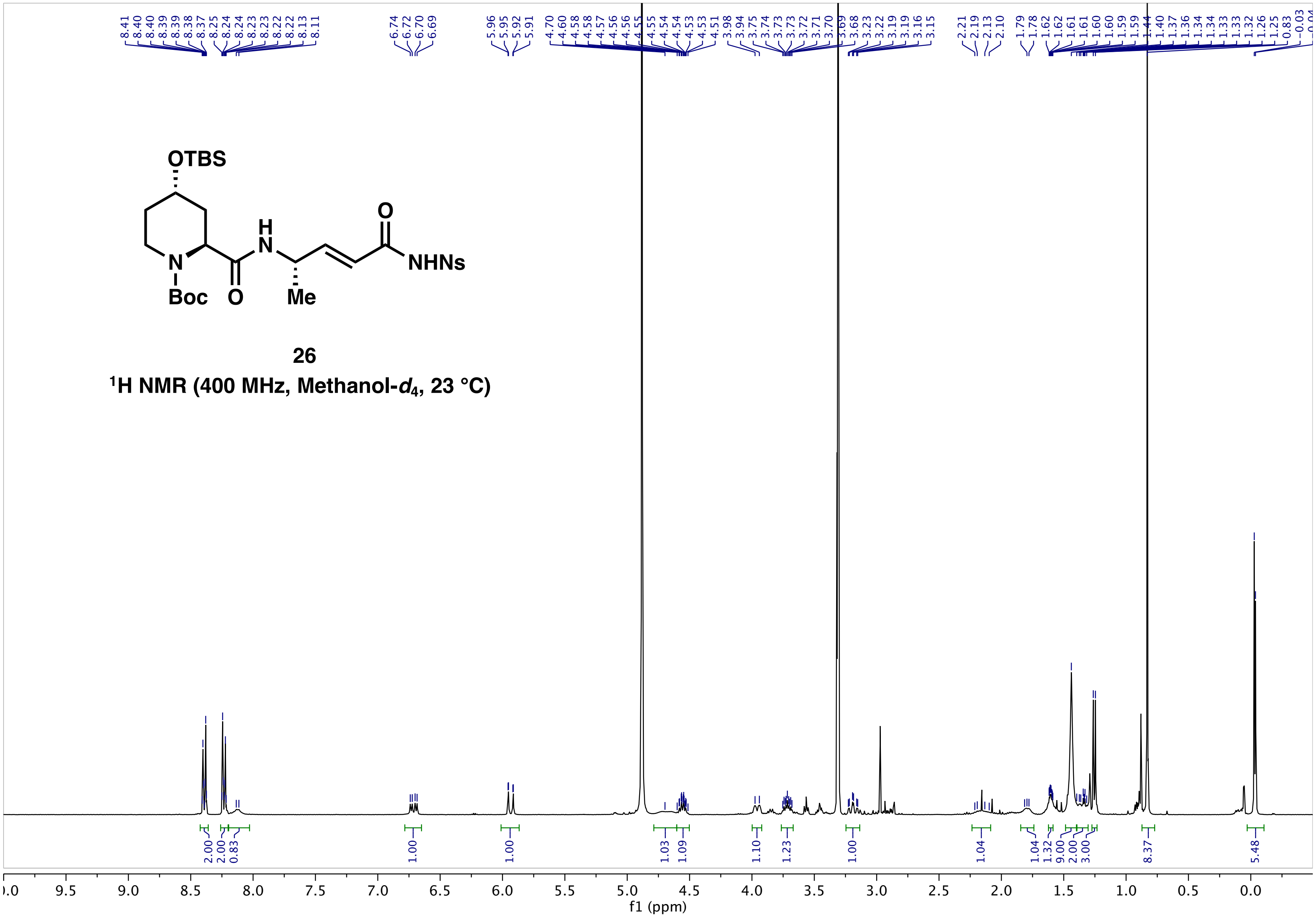
OTBS
H
N
O
NHNs
N
Boc
O
Me
26
1H NMR (400 MHz, Methanol-d4, 23 °C)
f1 (ppm)

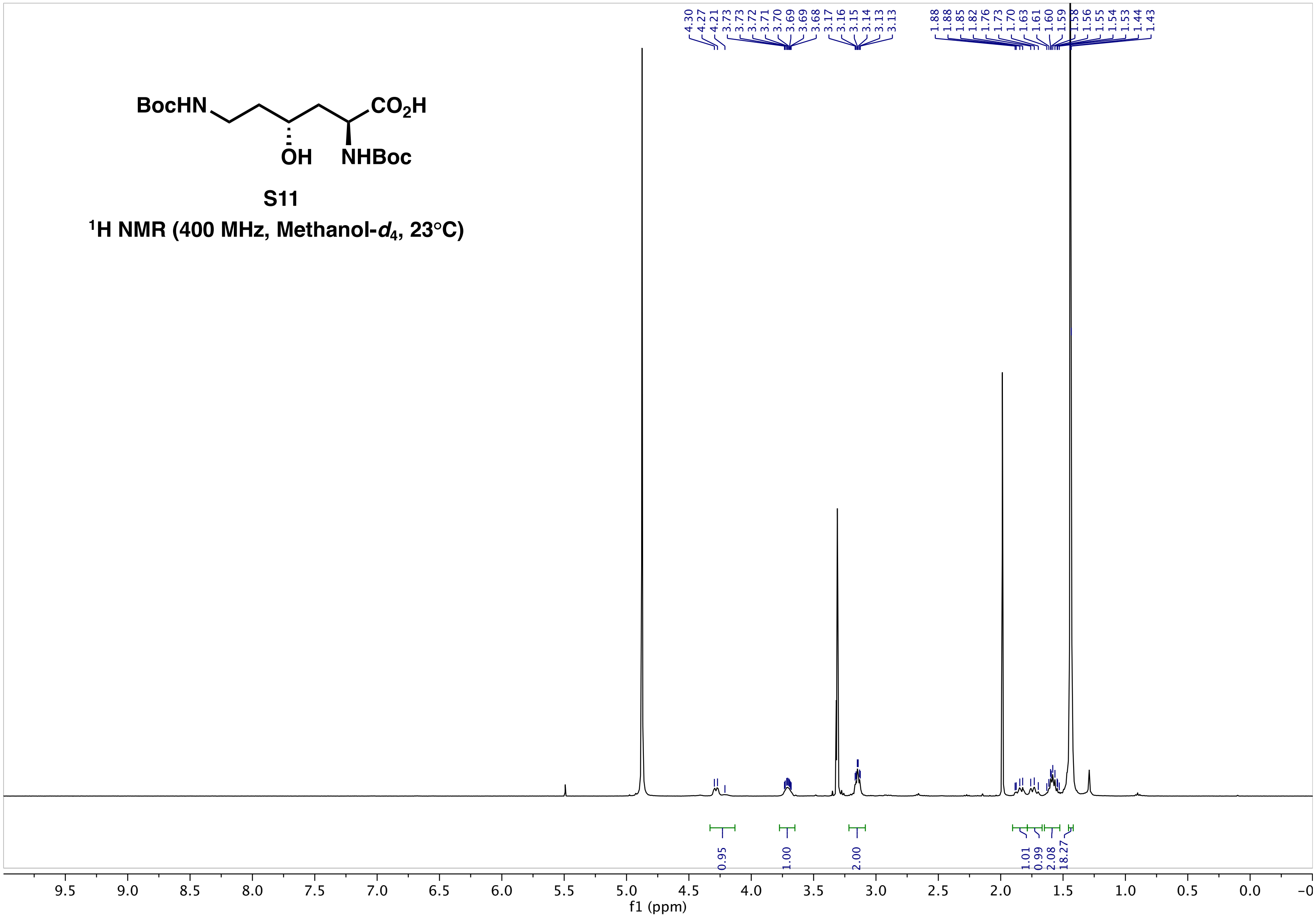

BocHN
CO2H
OH
NHBoc
S11
1H NMR (400 MHz, Methanol-d4, 23°C)
4.30 4.27 4.21 3.73 3.73 3.72 3.71 3.70 3.69 3.69 3.68 3.17 3.16 3.15 3.14 3.13 3.13
1.88 1.88 1.85 1.82 1.76 1.73 1.70 1.63 1.61 1.60 1.59 1.58 1.56 1.55 1.54 1.53 1.44 1.43
0.95 1.00 2.00 1.01 0.99 2.08 18.27
9.5 9.0 8.5 8.0 7.5 7.0 6.5 6.0 5.5 5.0 4.5 4.0 3.5 3.0 2.5 2.0 1.5 1.0 0.5 0.0
f1 (ppm)

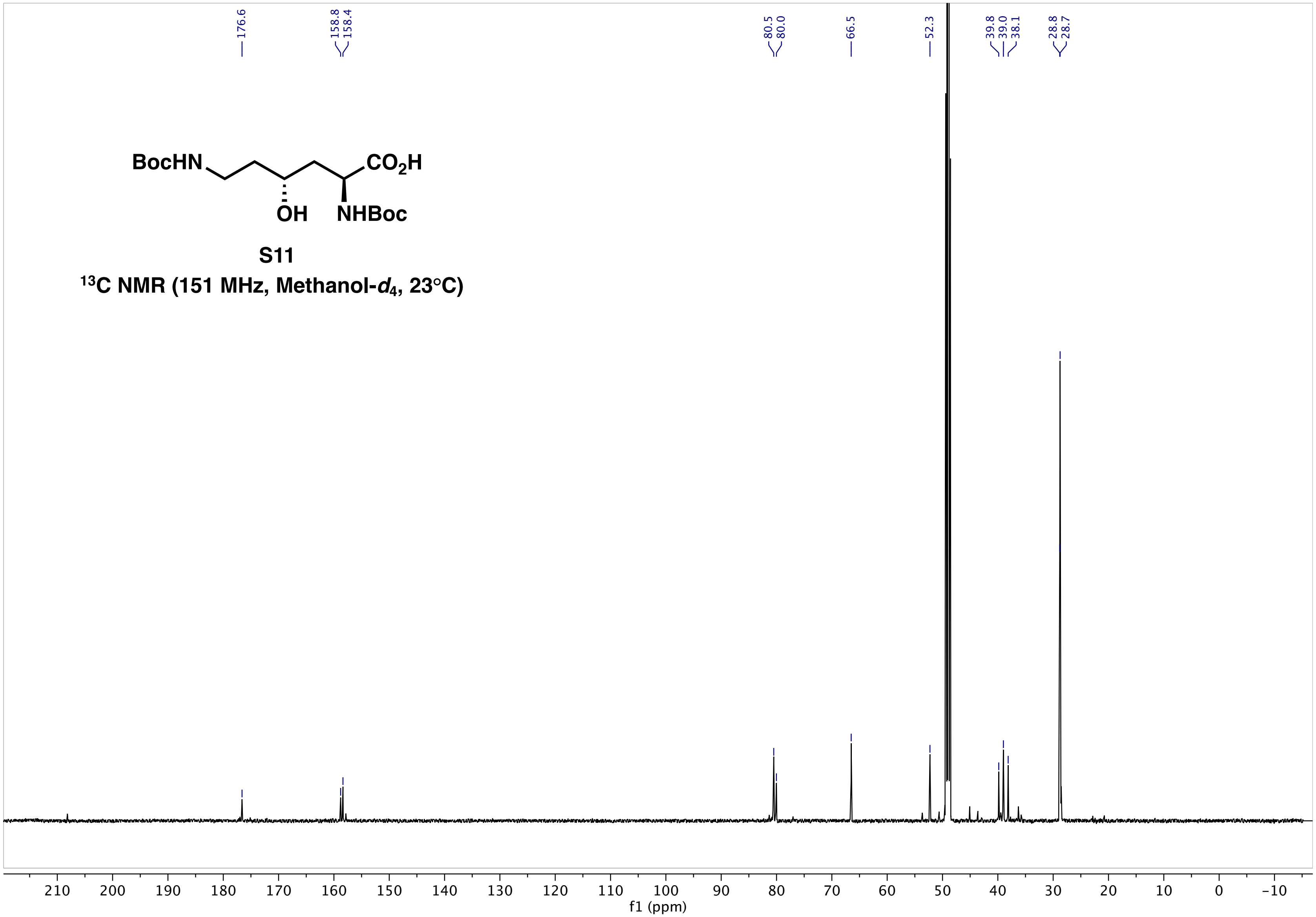
176.6
158.8
158.4
80.5
80.0
66.5
52.3
39.8
39.0
38.1
28.8
28.7
BocHN
CO2H
OH
NHBoc
S11
13C NMR (151 MHz, Methanol-d4, 23°C)
210 200 190 180 170 160 150 140 130 120 110 100 90 80 70 60 50 40 30 20 10 0 –10
f1 (ppm)

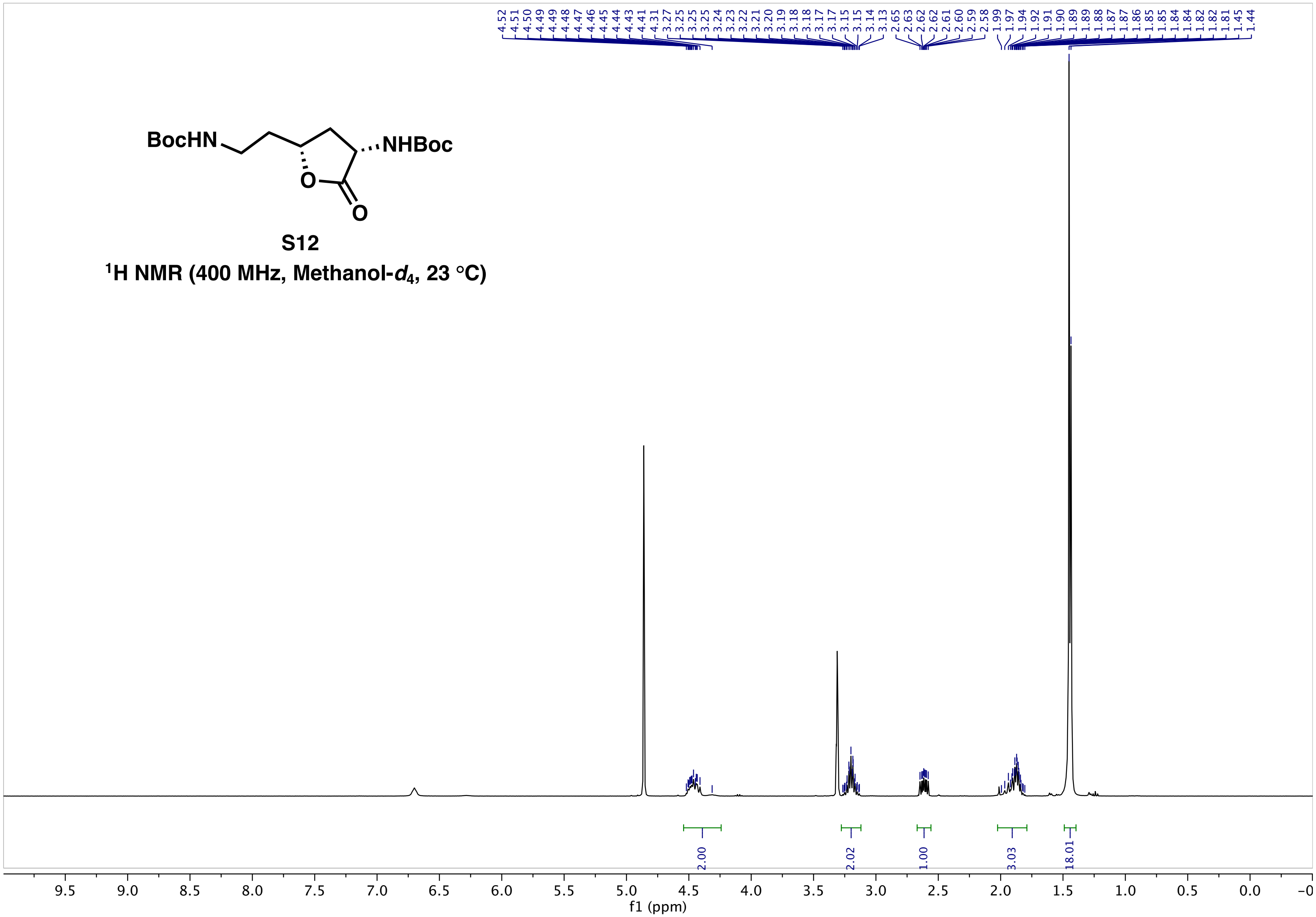
BocHN
NHBoc
O
O
S12
1H NMR (400 MHz, Methanol-d4, 23 °C)
f1 (ppm)
2.00
2.02
1.00
3.03
18.01

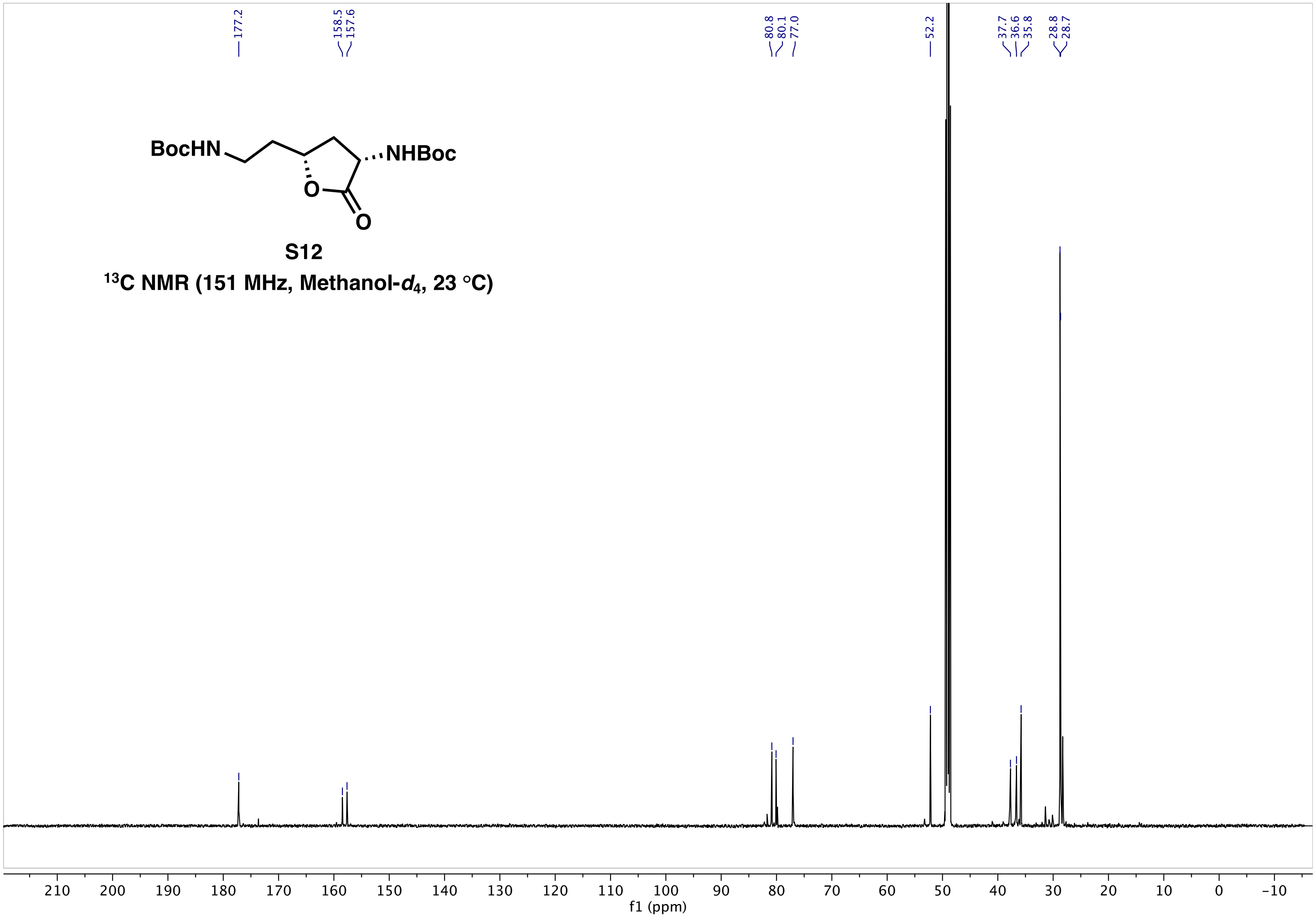

BocHN
NHBoc
O
O
S12
13C NMR (151 MHz, Methanol-d4, 23 °C)
177.2
158.5
157.6
80.8
80.1
77.0
52.2
37.7
36.6
35.8
28.8
28.7
210
200
190
180
170
160
150
140
130
120
110
100
90
80
70
60
50
40
30
20
10
0
–10
f1 (ppm)

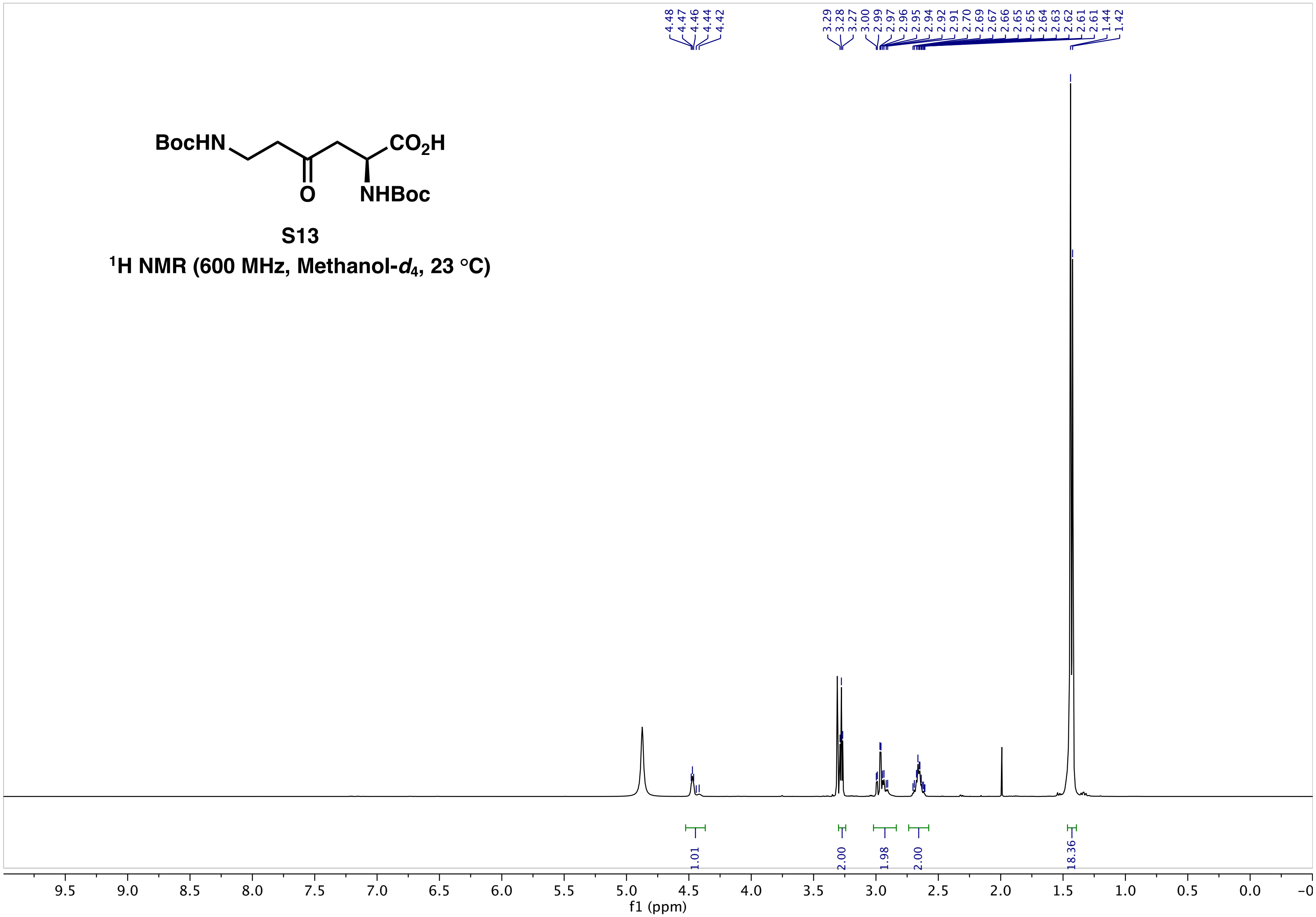

BocHN
CO2H
O
NHBoc
S13
1H NMR (600 MHz, Methanol-d4, 23 °C)
4.48
4.47
4.46
4.44
4.42
3.29
3.28
3.27
3.00
2.99
2.97
2.96
2.95
2.94
2.92
2.91
2.70
2.69
2.67
2.66
2.65
2.64
2.63
2.62
2.61
2.61
1.44
1.42
1.01
2.00
1.98
2.00
18.36
9.5
9.0
8.5
8.0
7.5
7.0
6.5
6.0
5.5
5.0
4.5
4.0
3.5
3.0
2.5
2.0
1.5
1.0
0.5
0.0
f1 (ppm)

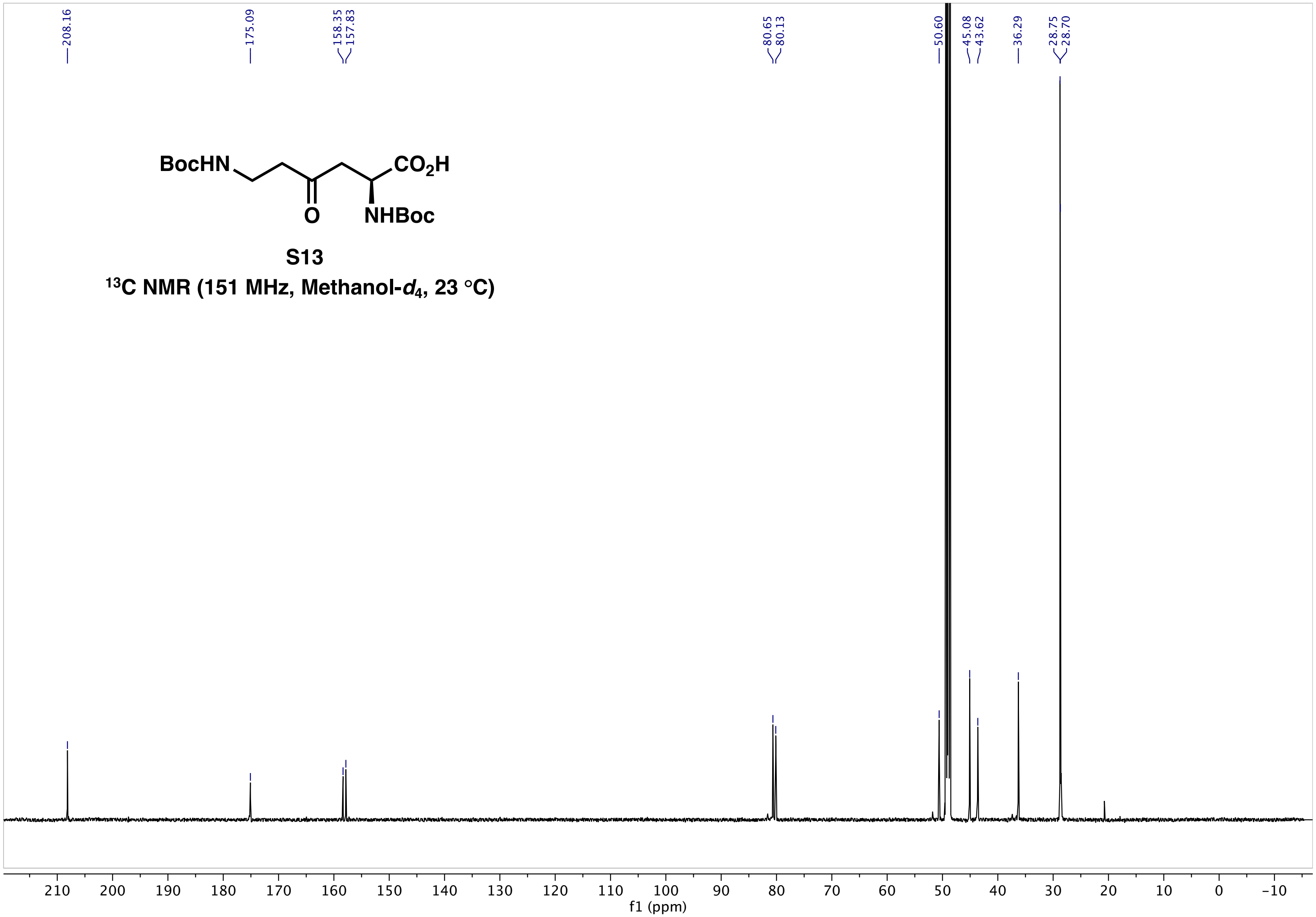

208.16
175.09
158.35
157.83
80.65
80.13
50.60
45.08
43.62
36.29
28.75
28.70
BocHN
CO2H
O
NHBoc
S13
13C NMR (151 MHz, Methanol-d4, 23 °C)
210 200 190 180 170 160 150 140 130 120 110 100 90 80 70 60 50 40 30 20 10 0 −10
f1 (ppm)

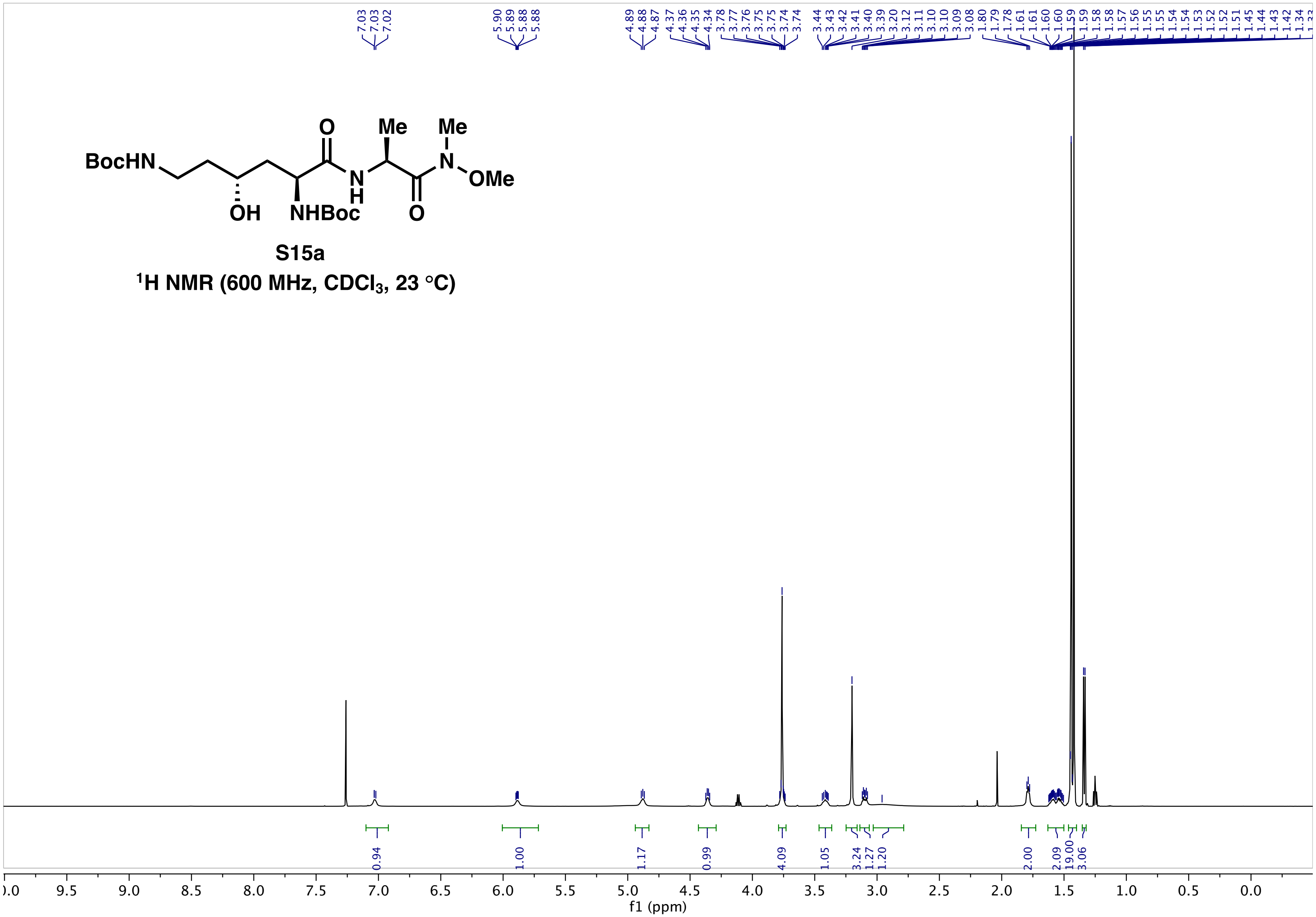
BocHN
OH
NHBoc
Me
Me
N
OMe
S15a
¹H NMR (600 MHz, CDCl₃, 23 °C)
f1 (ppm)

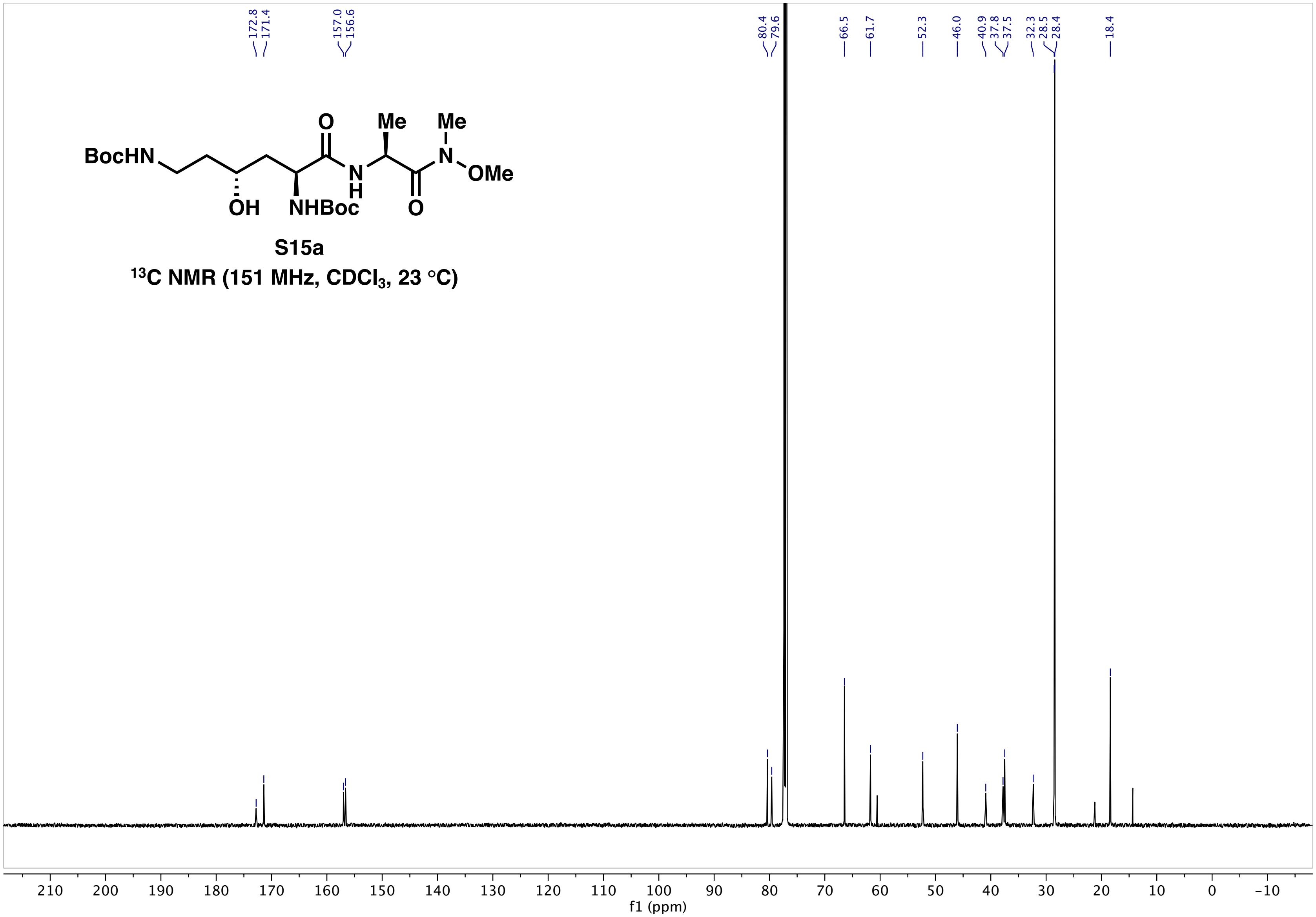

172.8
171.4
157.0
156.6
80.4
79.6
66.5
61.7
52.3
46.0
40.9
37.8
37.5
32.3
28.5
28.4
18.4
BocHN
O
Me
Me
N
OMe
N
H
OH
NHBoc
O
S15a
13C NMR (151 MHz, CDCl3, 23 °C)
210
200
190
180
170
160
150
140
130
120
110
100
90
80
70
60
50
40
30
20
10
0
−10
f1 (ppm)

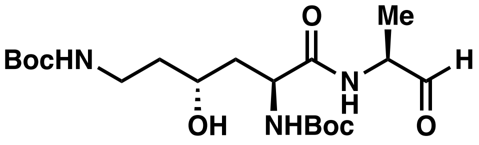

S16a

^{1}H NMR (400 MHz, $CDCl_3$, 23 °C)

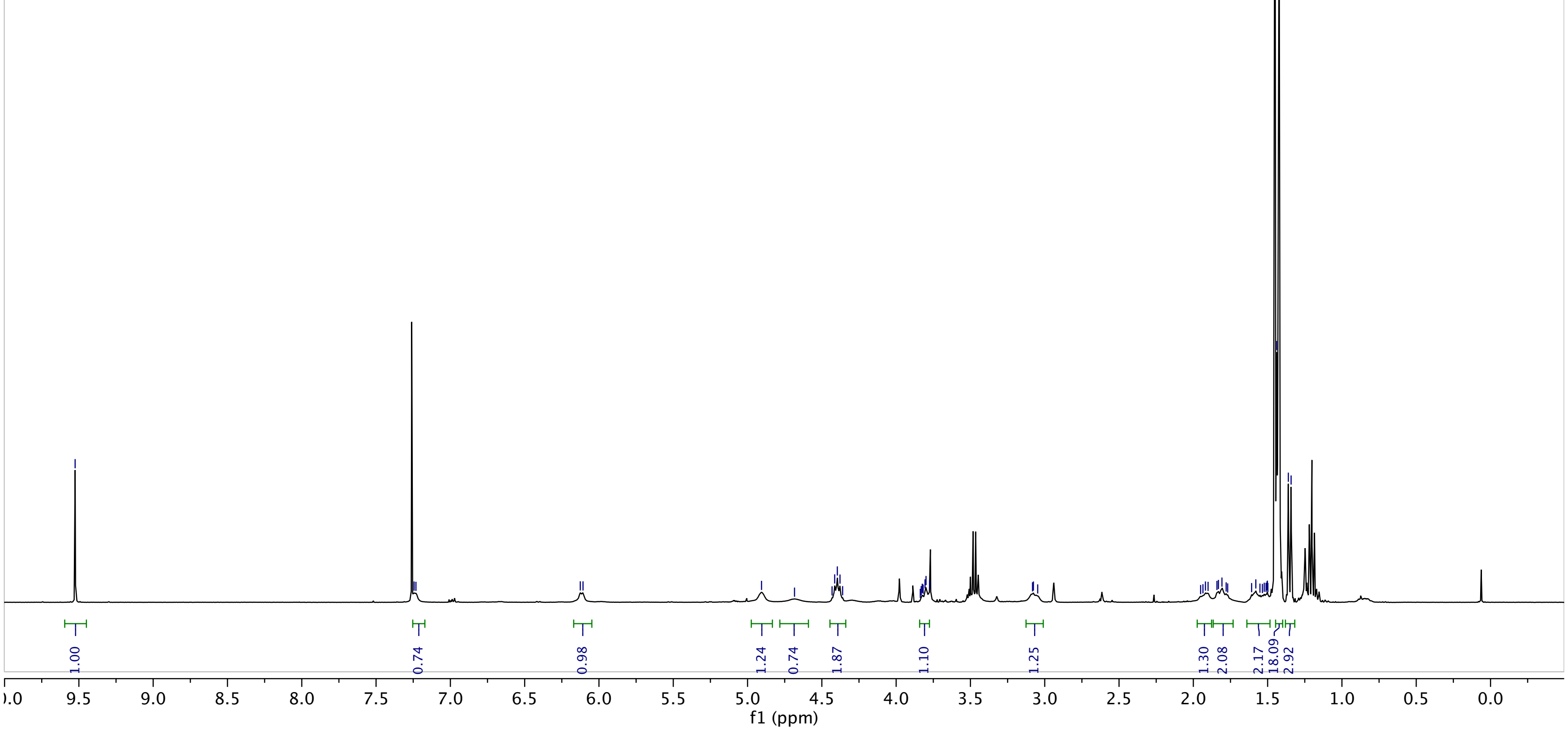

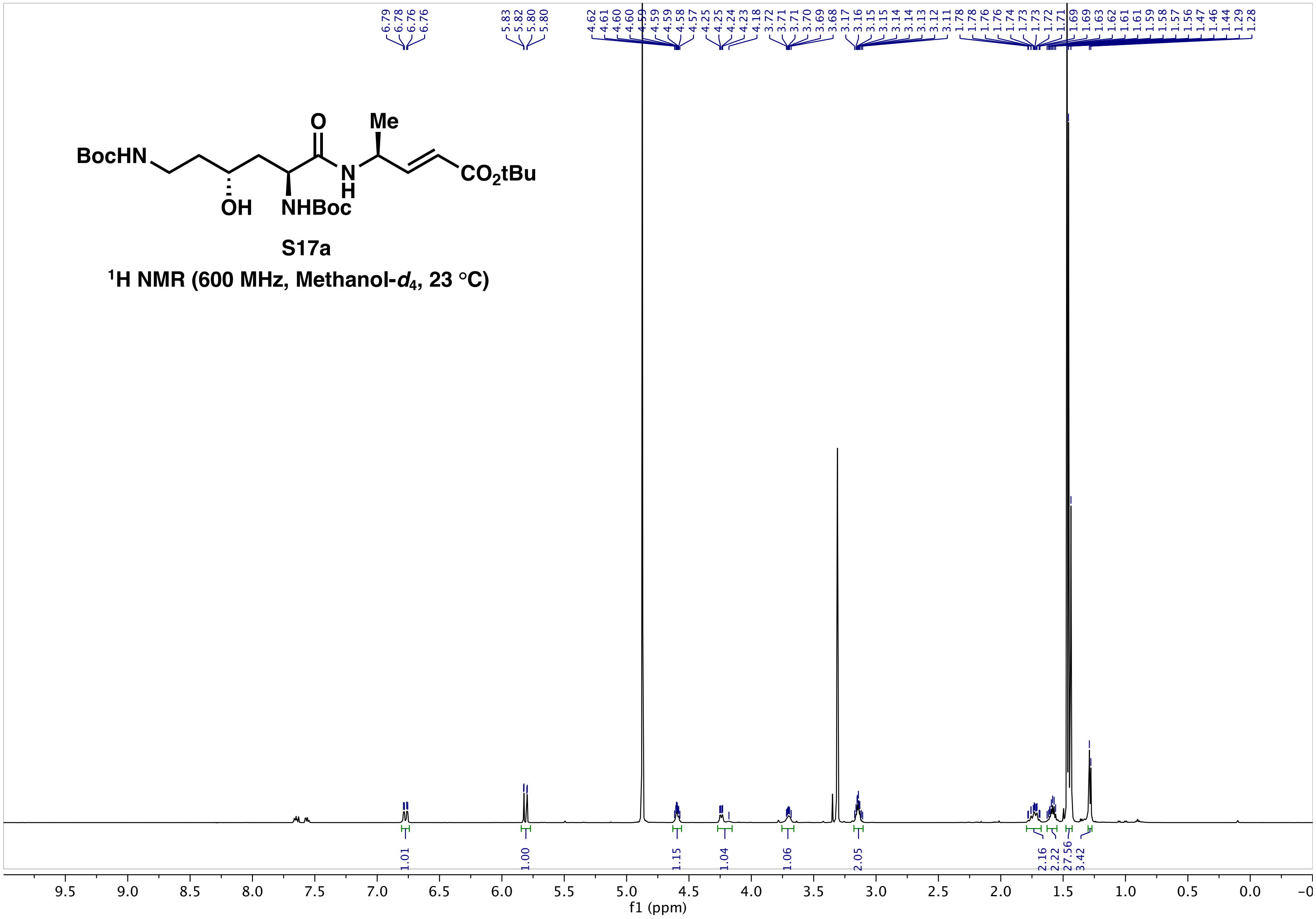
BocHN
OH
NHBoc
O
Me
N
H
CO2tBu
S17a
1H NMR (600 MHz, Methanol-d4, 23 °C)
f1 (ppm)

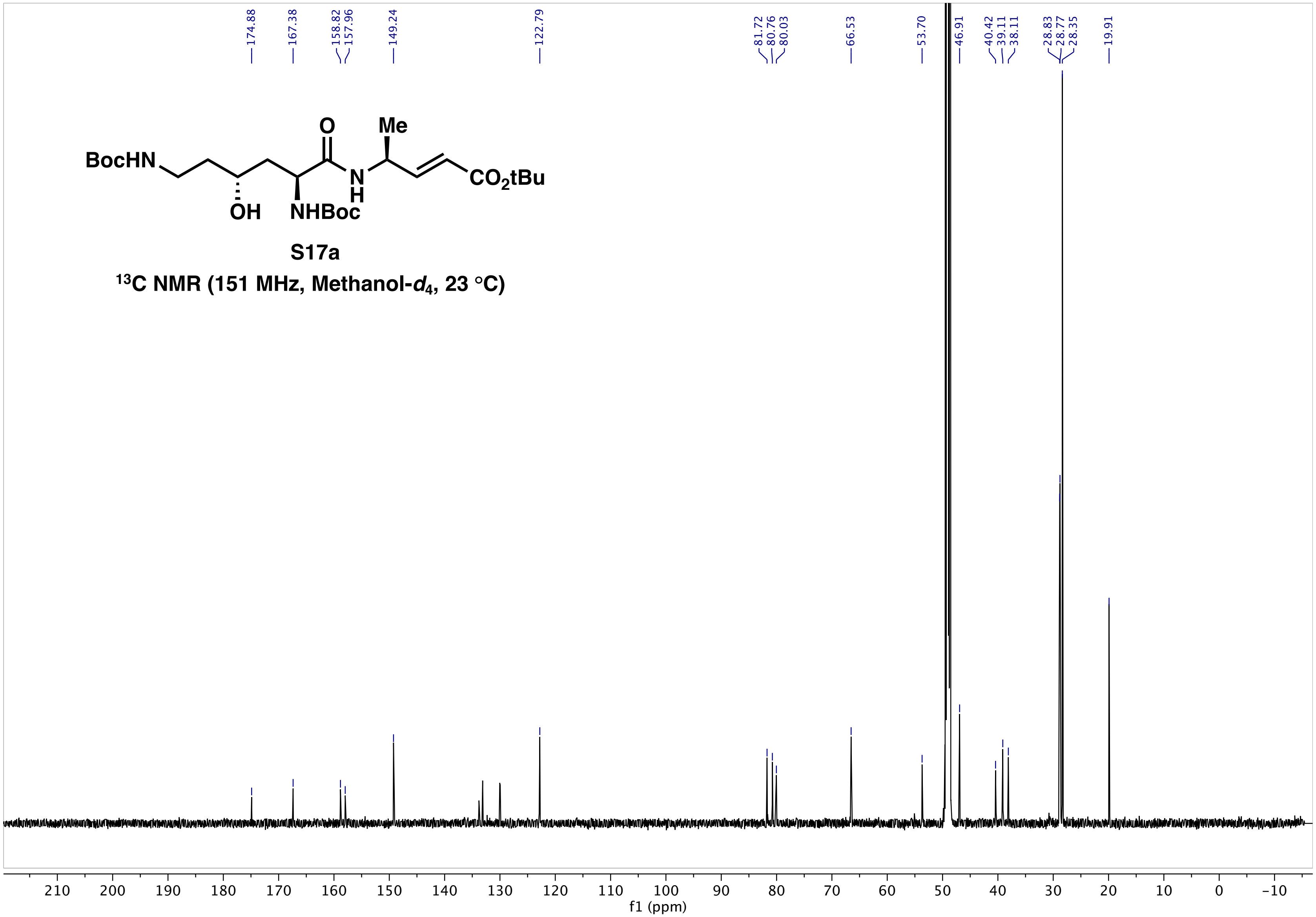

174.88
167.38
158.82
157.96
149.24
122.79
81.72
80.76
80.03
66.53
53.70
46.91
40.42
39.11
38.11
28.83
28.77
28.35
19.91
BocHN
O
Me
N
H
CO2tBu
OH
NHBoc
S17a
13C NMR (151 MHz, Methanol-d4, 23 °C)
210
200
190
180
170
160
150
140
130
120
110
100
90
80
70
60
50
40
30
20
10
0
-10
f1 (ppm)

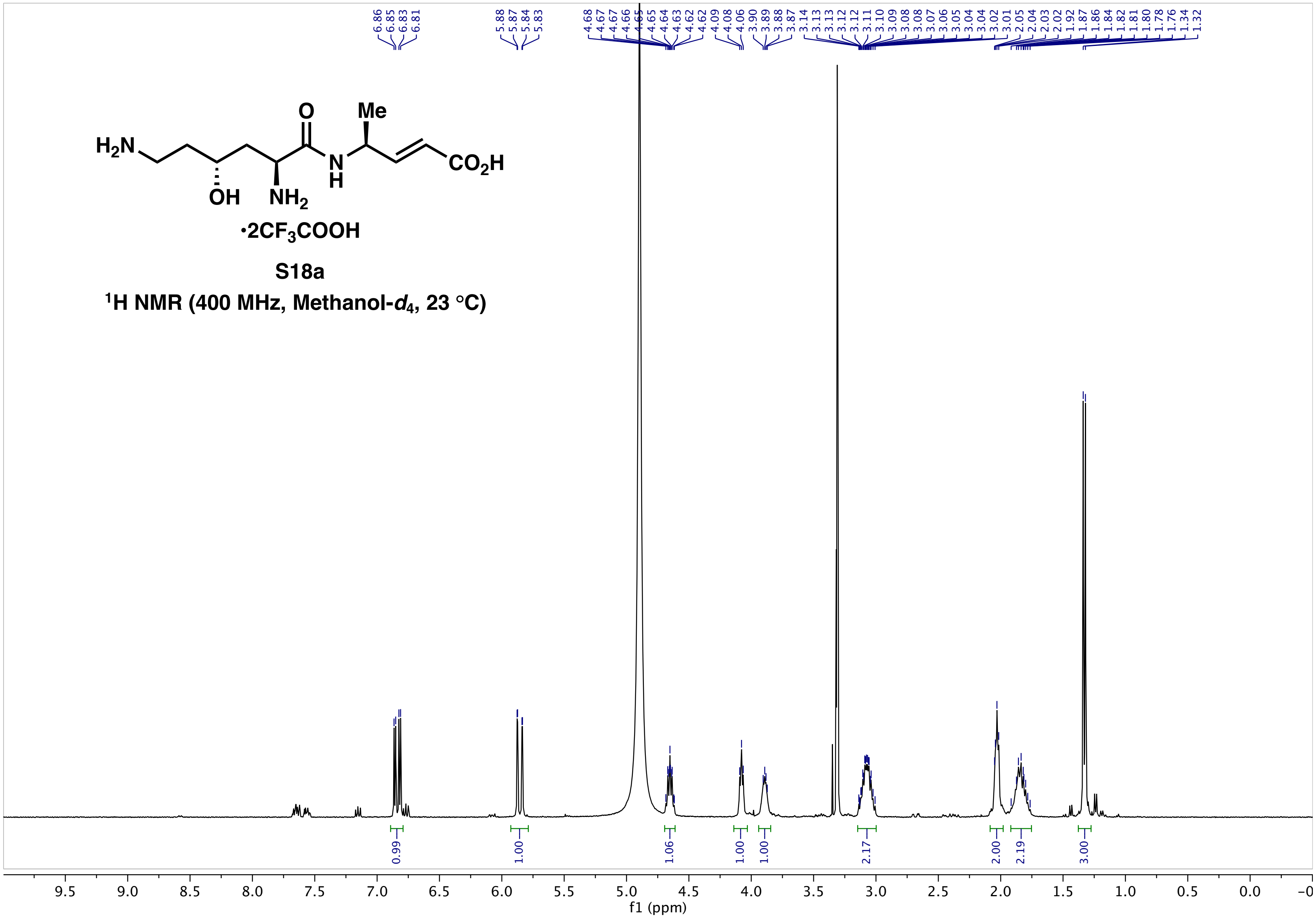

•2CF3COOH
S18a
1H NMR (400 MHz, Methanol-d4, 23 °C)
f1 (ppm)

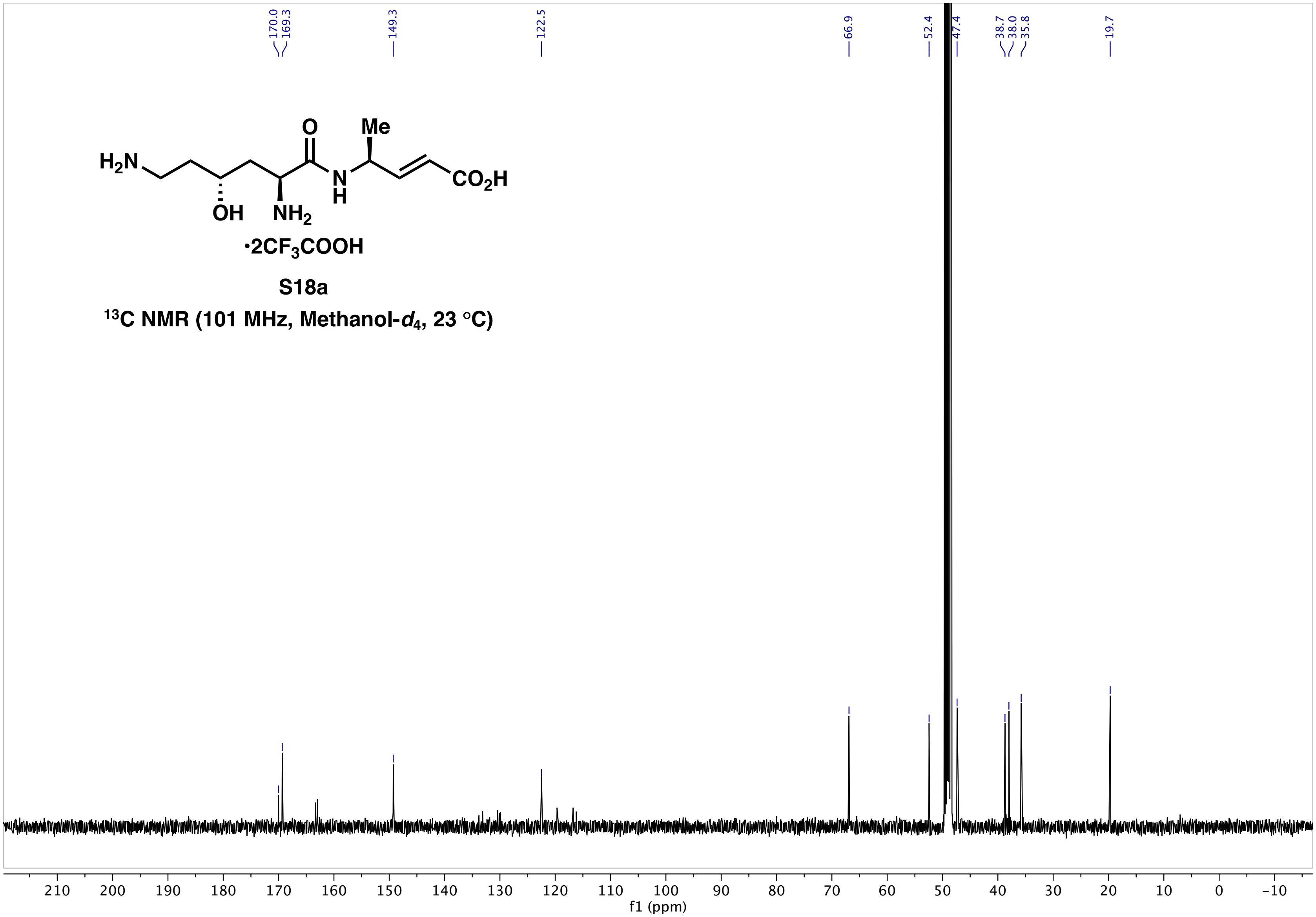
170.0
169.3
149.3
122.5
66.9
52.4
47.4
38.7
38.0
35.8
19.7
H2N
O
Me
N
H
CO2H
OH
NH2
•2CF3COOH
S18a
13C NMR (101 MHz, Methanol-d4, 23 °C)
210
200
190
180
170
160
150
140
130
120
110
100
90
80
70
60
50
40
30
20
10
0
–10
f1 (ppm)

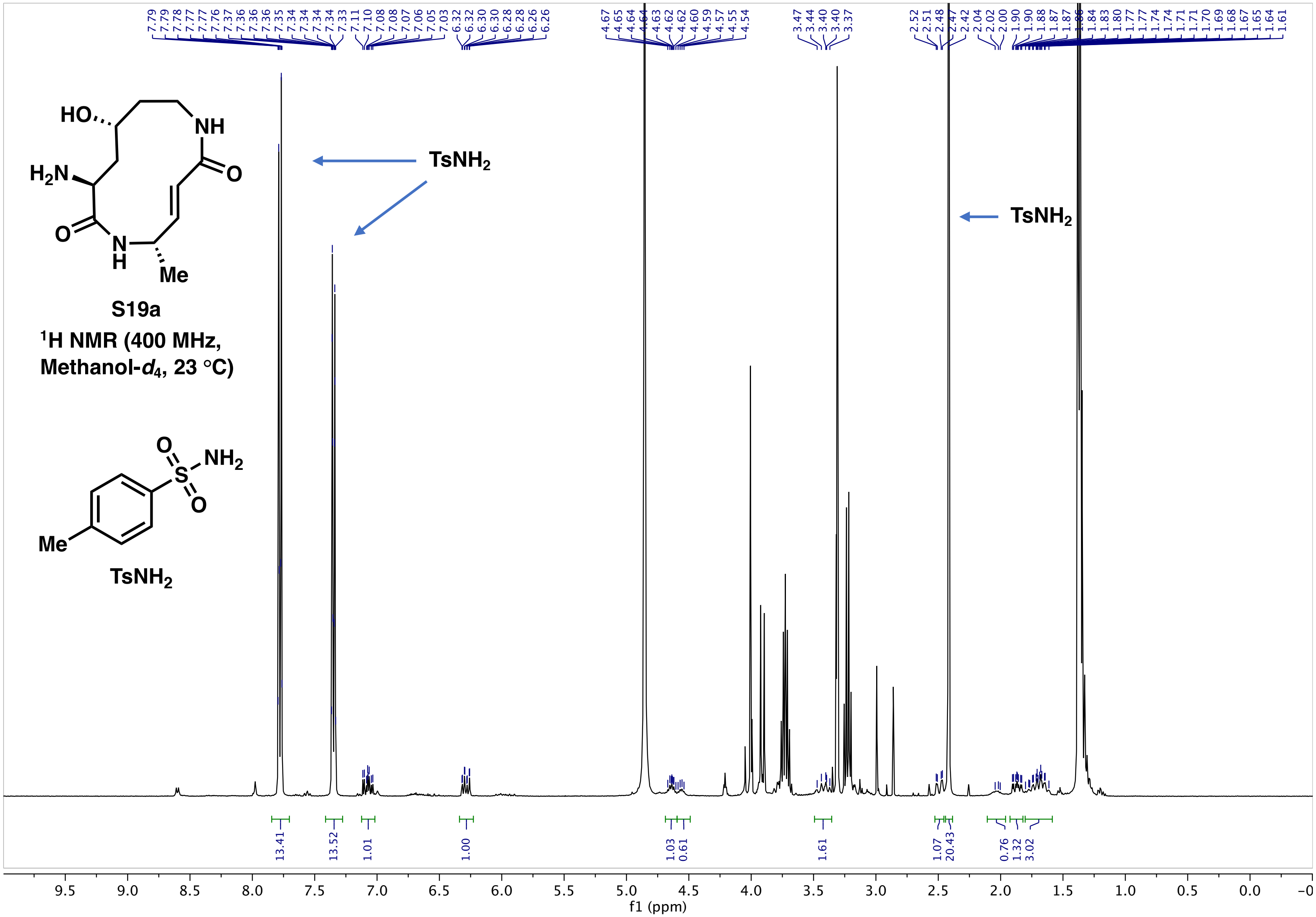
HO
NH
O
H2N
O
N
H
Me
S19a
1H NMR (400 MHz, Methanol-d4, 23 °C)
O
S
NH2
O
Me
TsNH2
TsNH2
TsNH2
f1 (ppm)

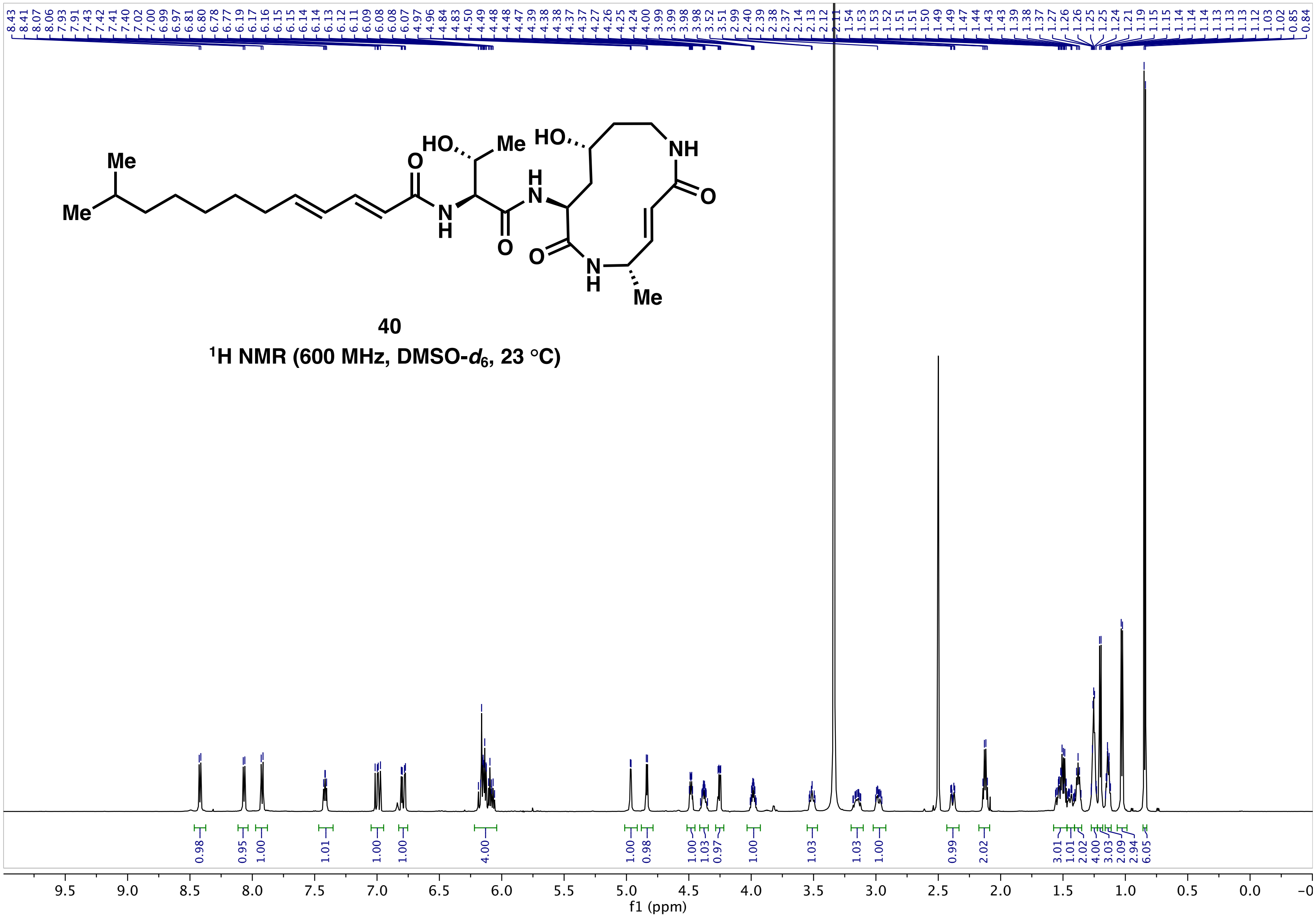
40
1H NMR (600 MHz, DMSO-d6, 23 °C)
f1 (ppm)

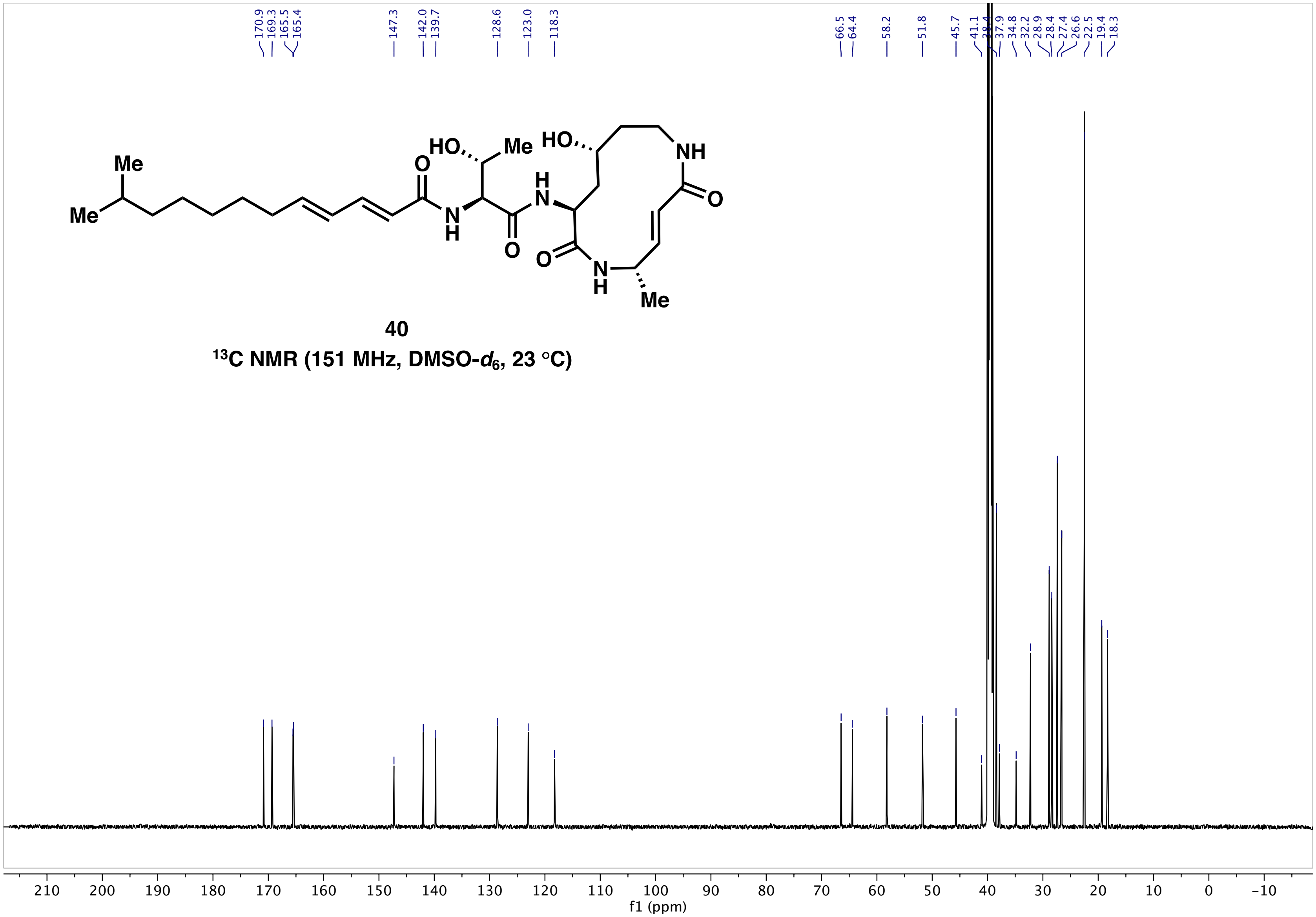
170.9
169.3
165.5
165.4
147.3
142.0
139.7
128.6
123.0
118.3
66.5
64.4
58.2
51.8
45.7
41.1
37.9
34.8
32.2
28.9
28.4
27.4
26.6
22.5
19.4
18.3
Me
Me
O
N
H
HO
Me
O
H
N
HO
NH
O
O
N
H
Me
40
^{13}C NMR (151 MHz, DMSO-d_6, 23 °C)
210
200
190
180
170
160
150
140
130
120
110
100
90
80
70
60
50
40
30
20
10
0
−10
f1 (ppm)

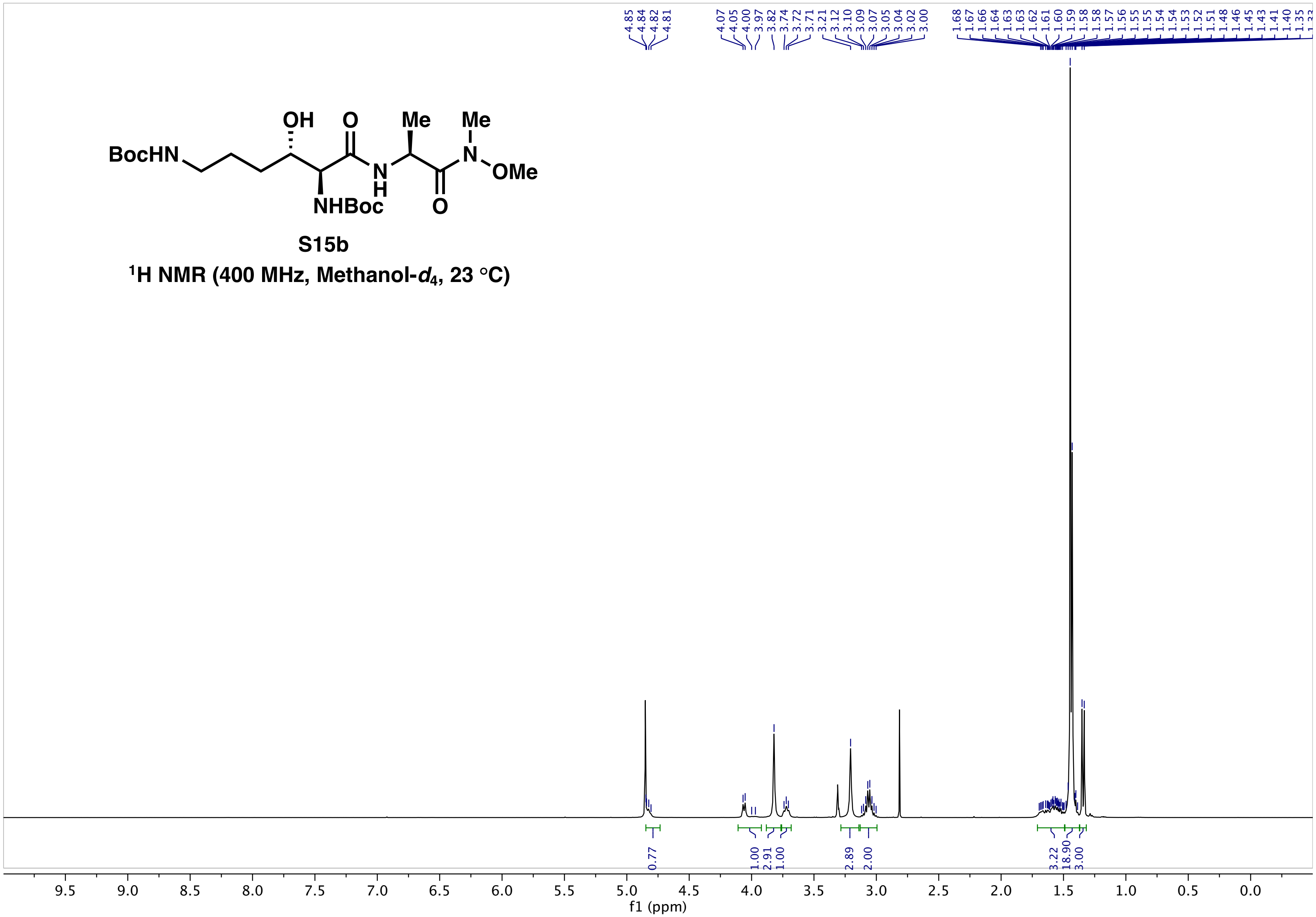

S15b

^{1}H NMR (400 MHz, Methanol-d_4, 23 °C)

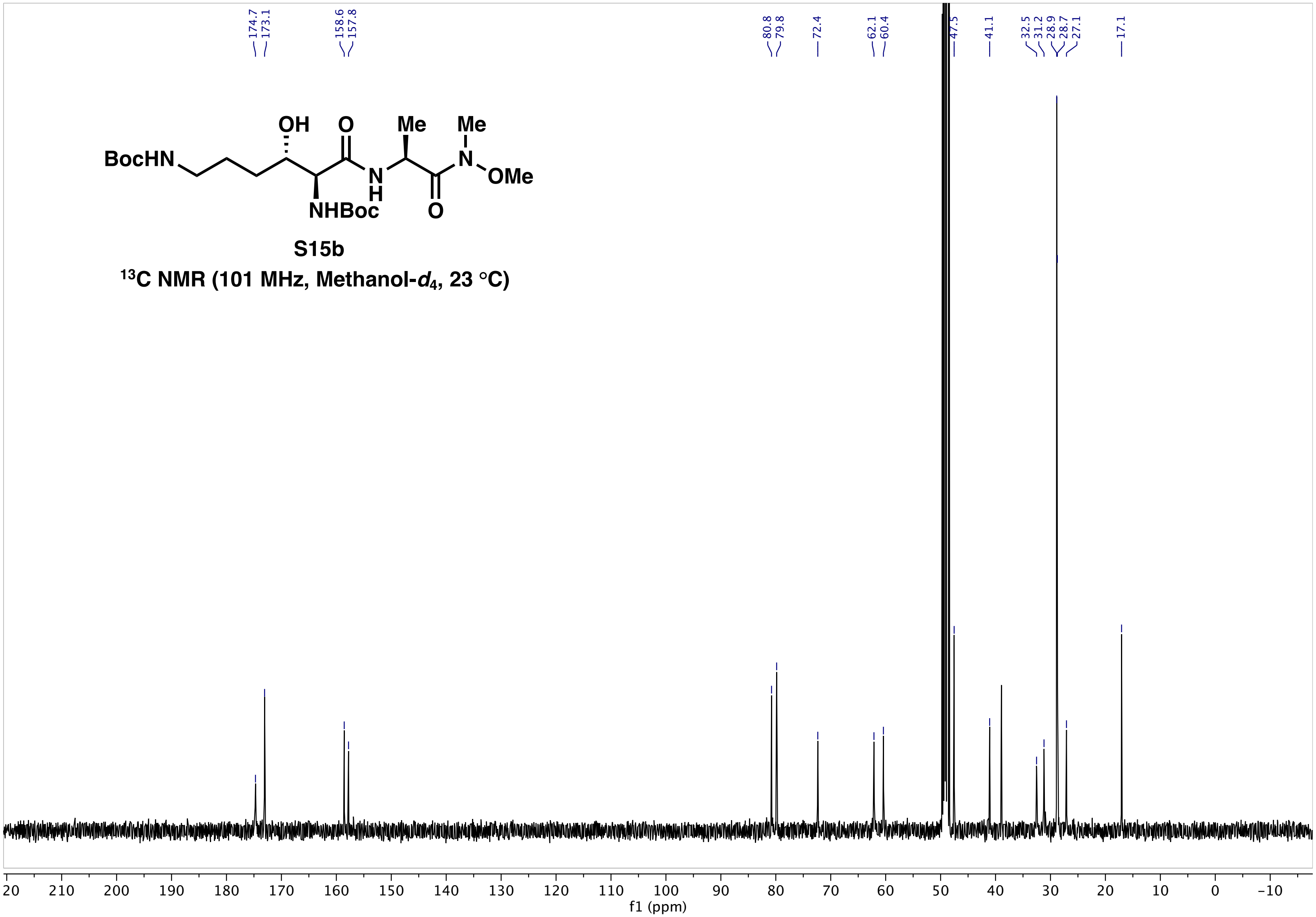
174.7
173.1
158.6
157.8
80.8
79.8
72.4
62.1
60.4
47.5
41.1
32.5
31.2
28.9
28.7
27.1
17.1
OH
O
Me
Me
BocHN
N
N
OMe
H
NHBoc
O
S15b
13C NMR (101 MHz, Methanol-d4, 23 °C)
20
210
200
190
180
170
160
150
140
130
120
110
100
90
80
70
60
50
40
30
20
10
0
-10
f1 (ppm)

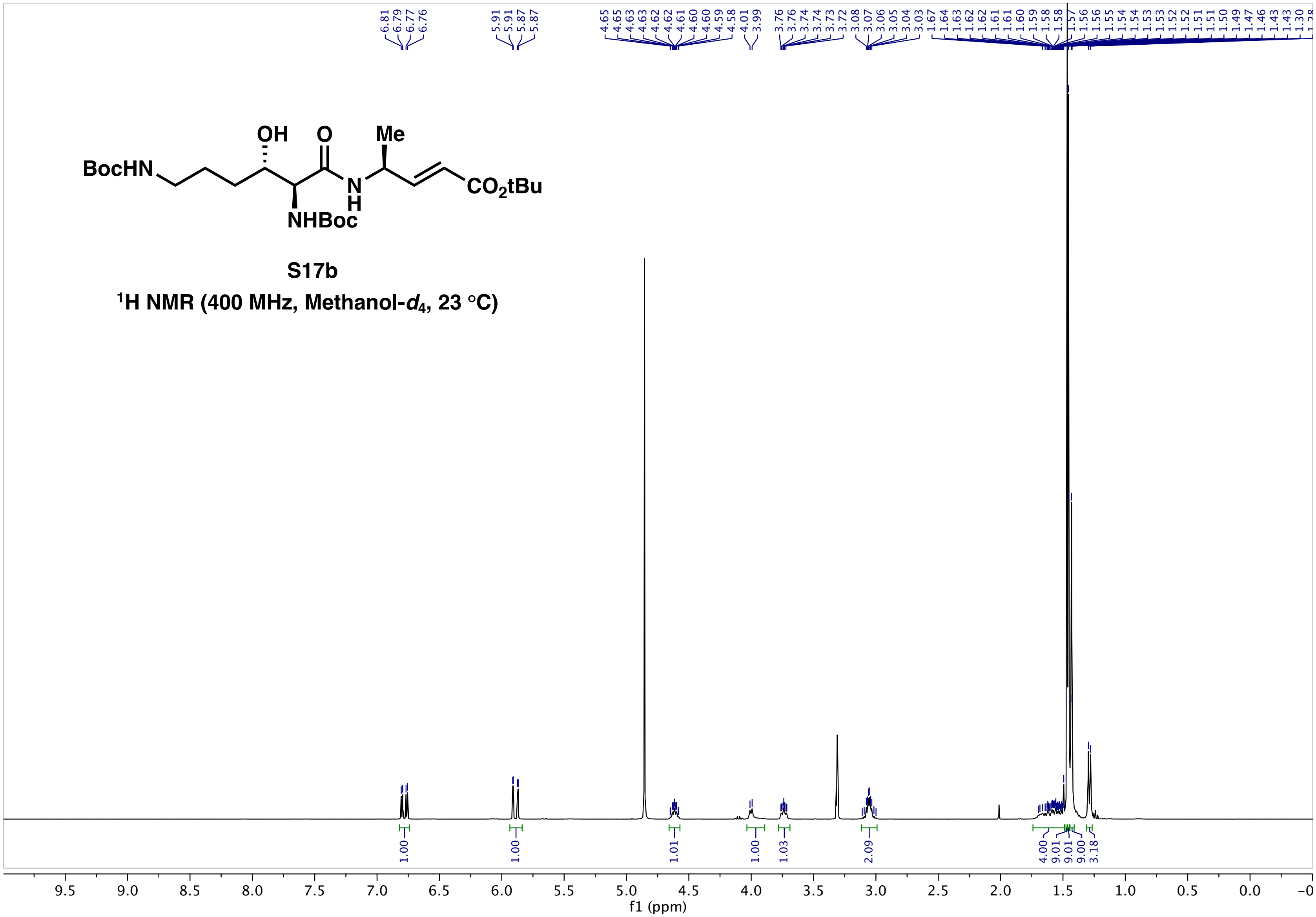

S17b

^{1}H NMR (400 MHz, Methanol-d_4, 23 °C)

S17b

^{13}C NMR (101 MHz, Methanol-d_4, 23 °C)

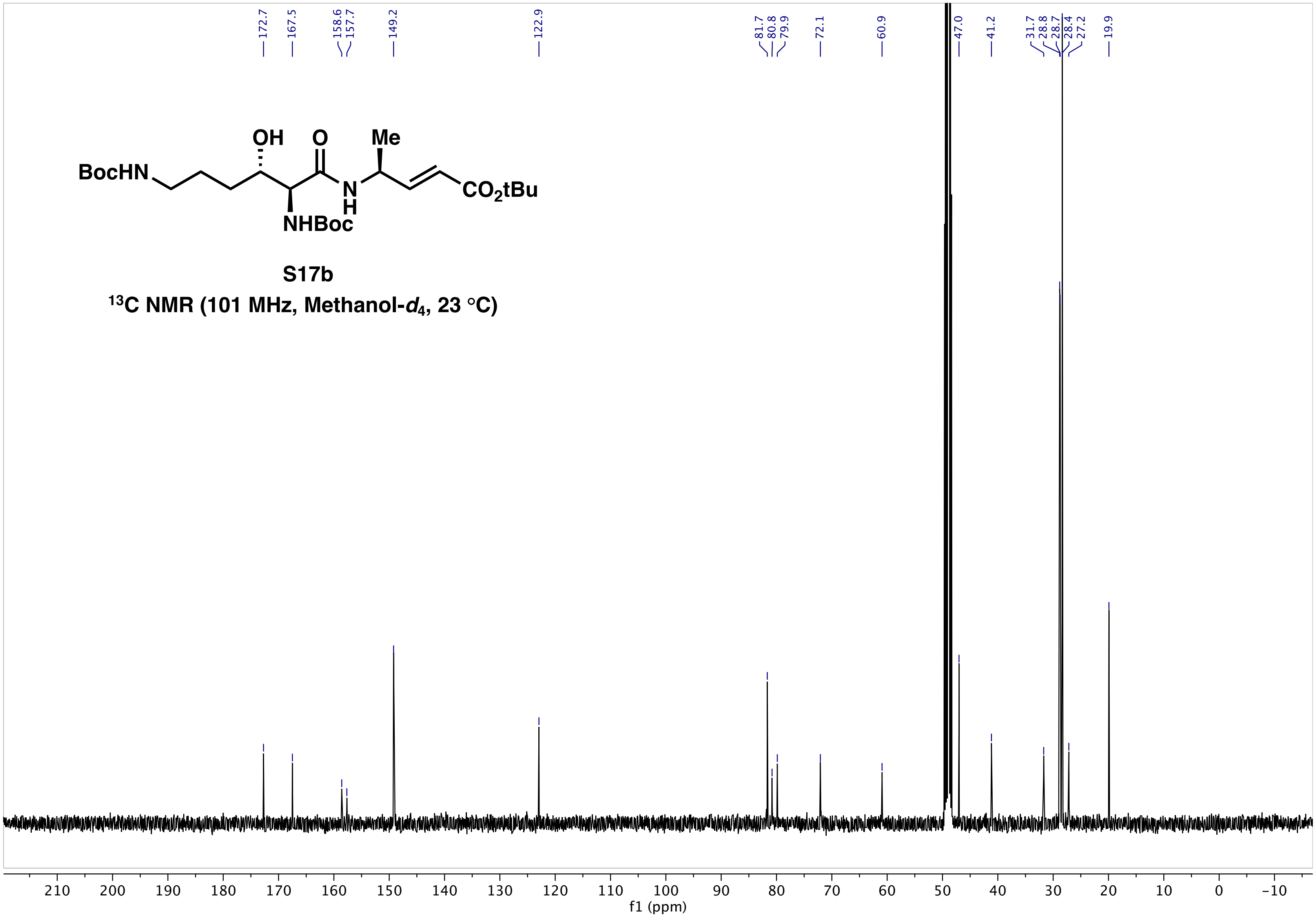

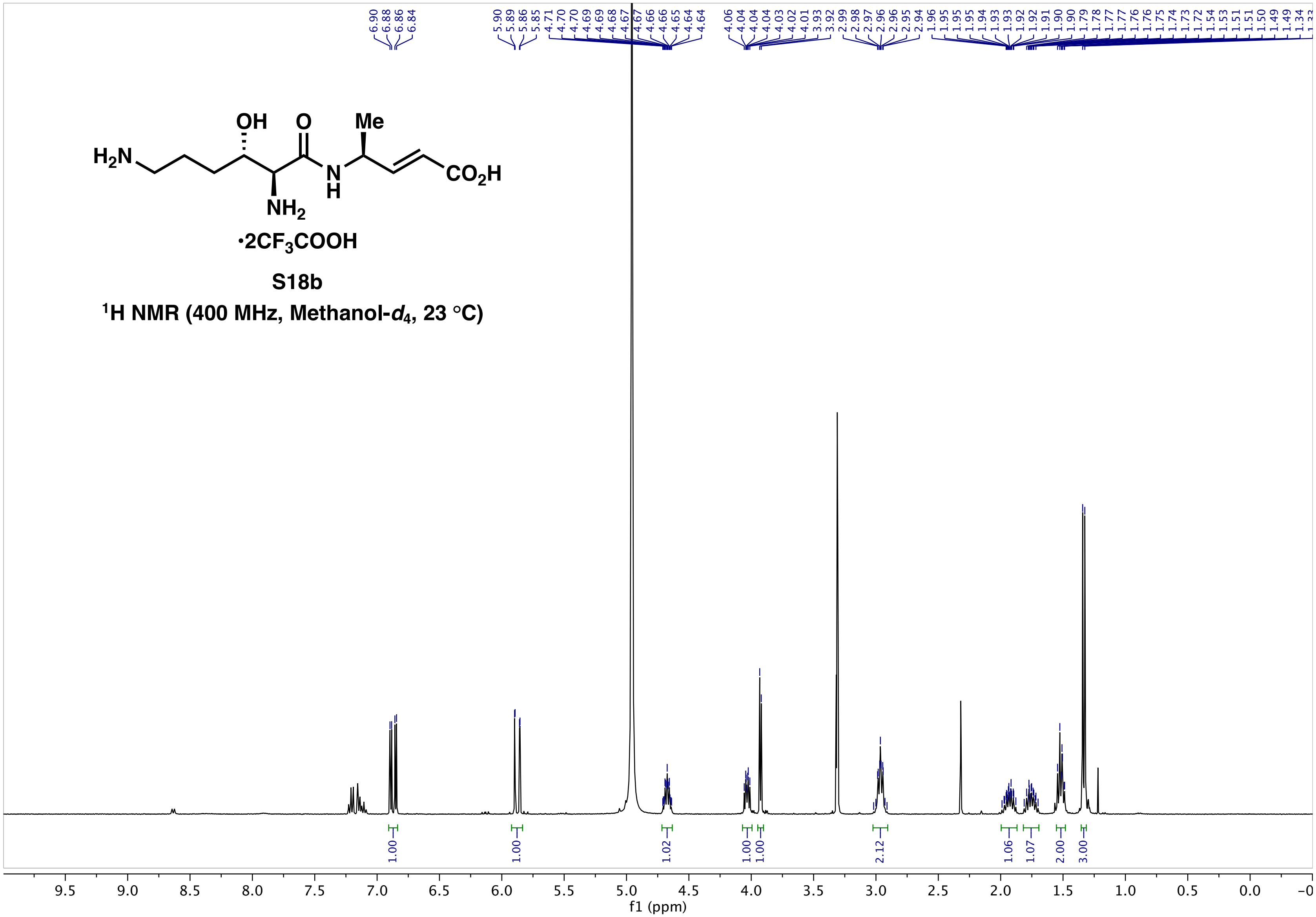
H2N
OH
O
Me
N
H
CO2H
NH2
•2CF3COOH
S18b
1H NMR (400 MHz, Methanol-d4, 23 °C)
f1 (ppm)

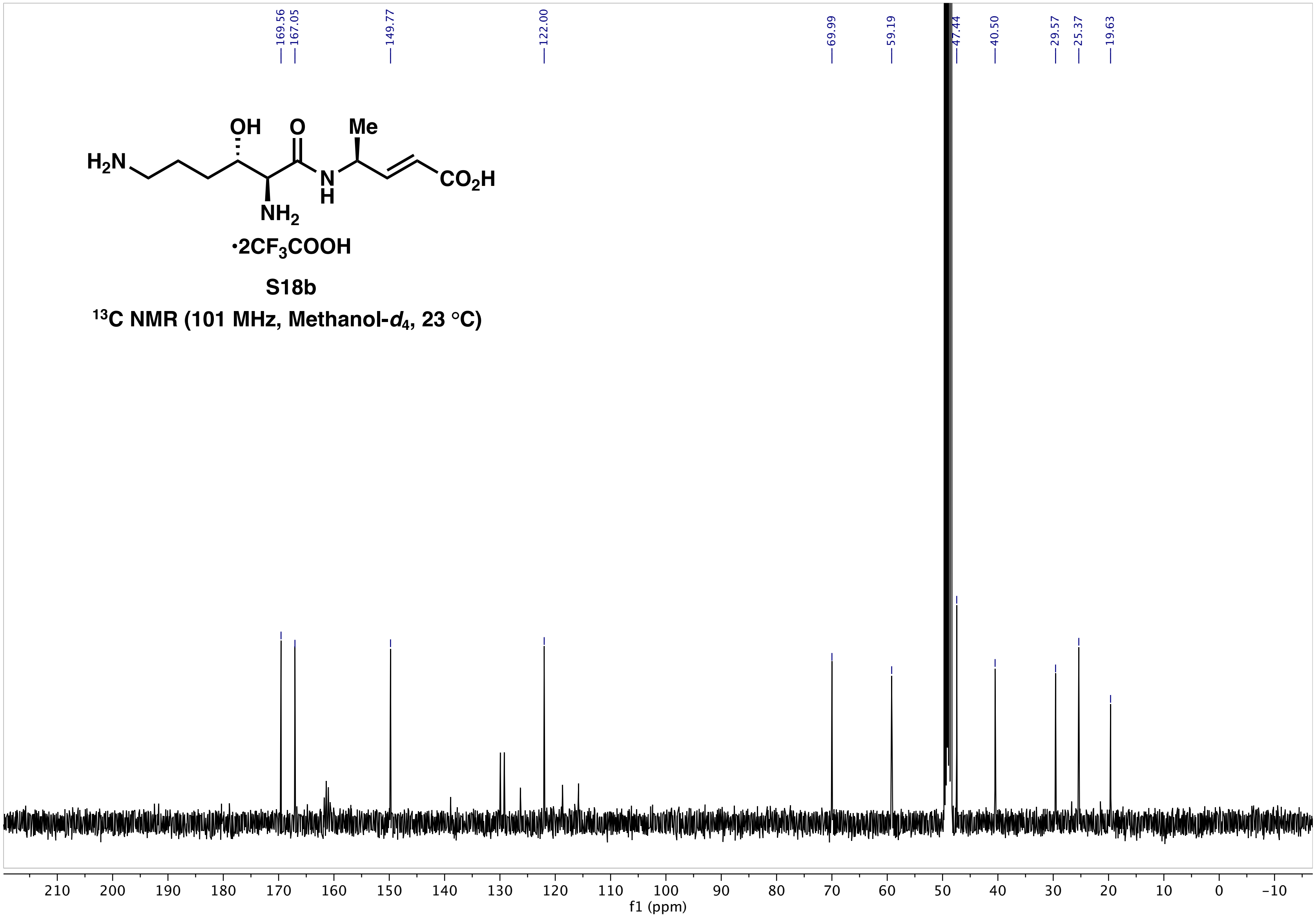

169.56
167.05
149.77
122.00
69.99
59.19
47.44
40.50
29.57
25.37
19.63
OH
O
Me
H2N
N
H
CO2H
NH2
•2CF3COOH
S18b
13C NMR (101 MHz, Methanol-d4, 23 °C)
210
200
190
180
170
160
150
140
130
120
110
100
90
80
70
60
50
40
30
20
10
0
-10
f1 (ppm)

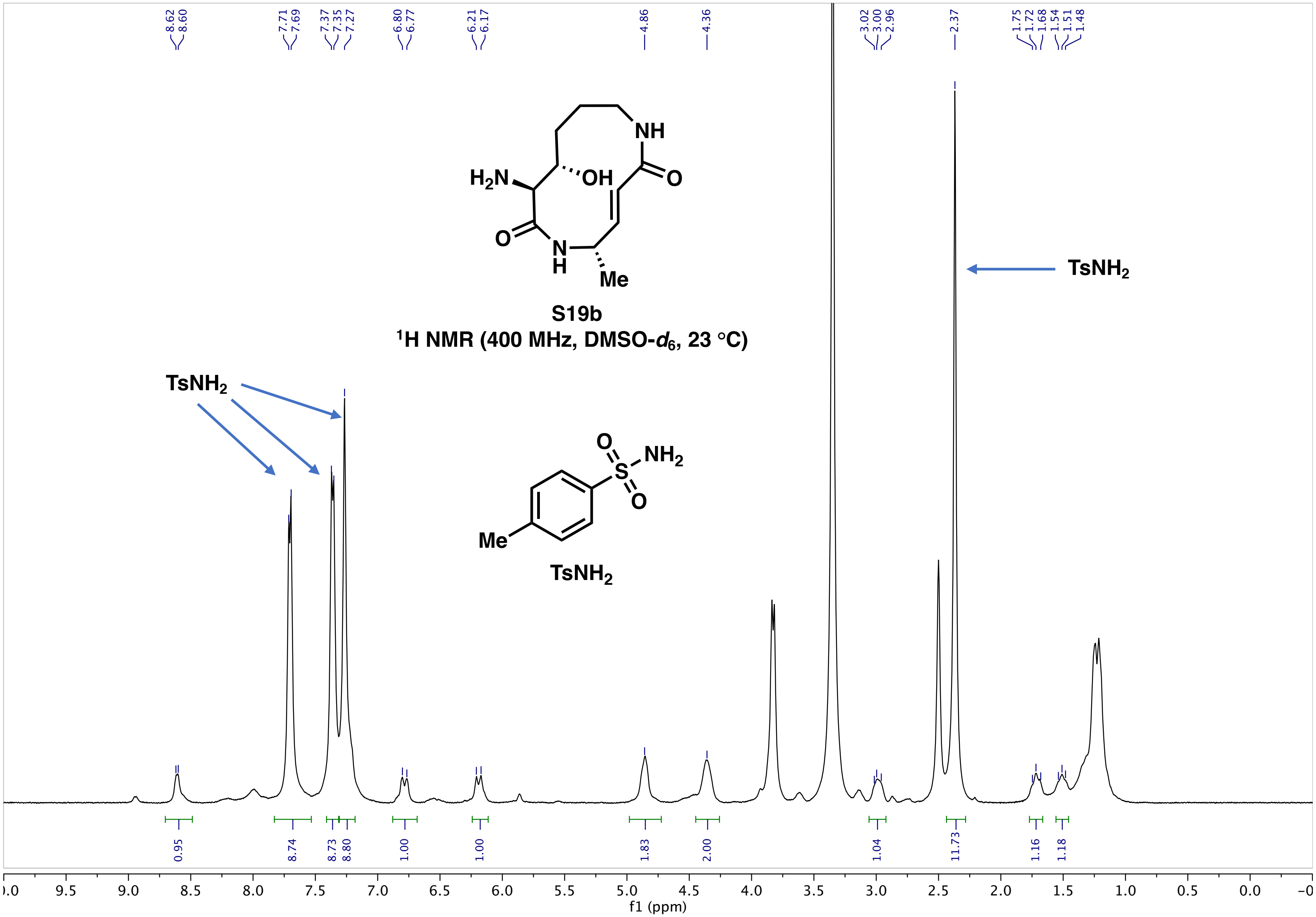
8.62
8.60
7.71
7.69
7.37
7.35
7.27
6.80
6.77
6.21
6.17
4.86
4.36
3.02
3.00
2.96
2.37
1.75
1.72
1.68
1.54
1.51
1.48
H2N
OH
NH
O
O
N
H
Me
S19b
1H NMR (400 MHz, DMSO-d6, 23 °C)
TsNH2
TsNH2
O
S
NH2
O
Me
TsNH2
0.95
8.74
8.73
8.80
1.00
1.00
1.83
2.00
1.04
11.73
1.16
1.18
9.5
9.0
8.5
8.0
7.5
7.0
6.5
6.0
5.5
5.0
4.5
4.0
3.5
3.0
2.5
2.0
1.5
1.0
0.5
0.0
f1 (ppm)

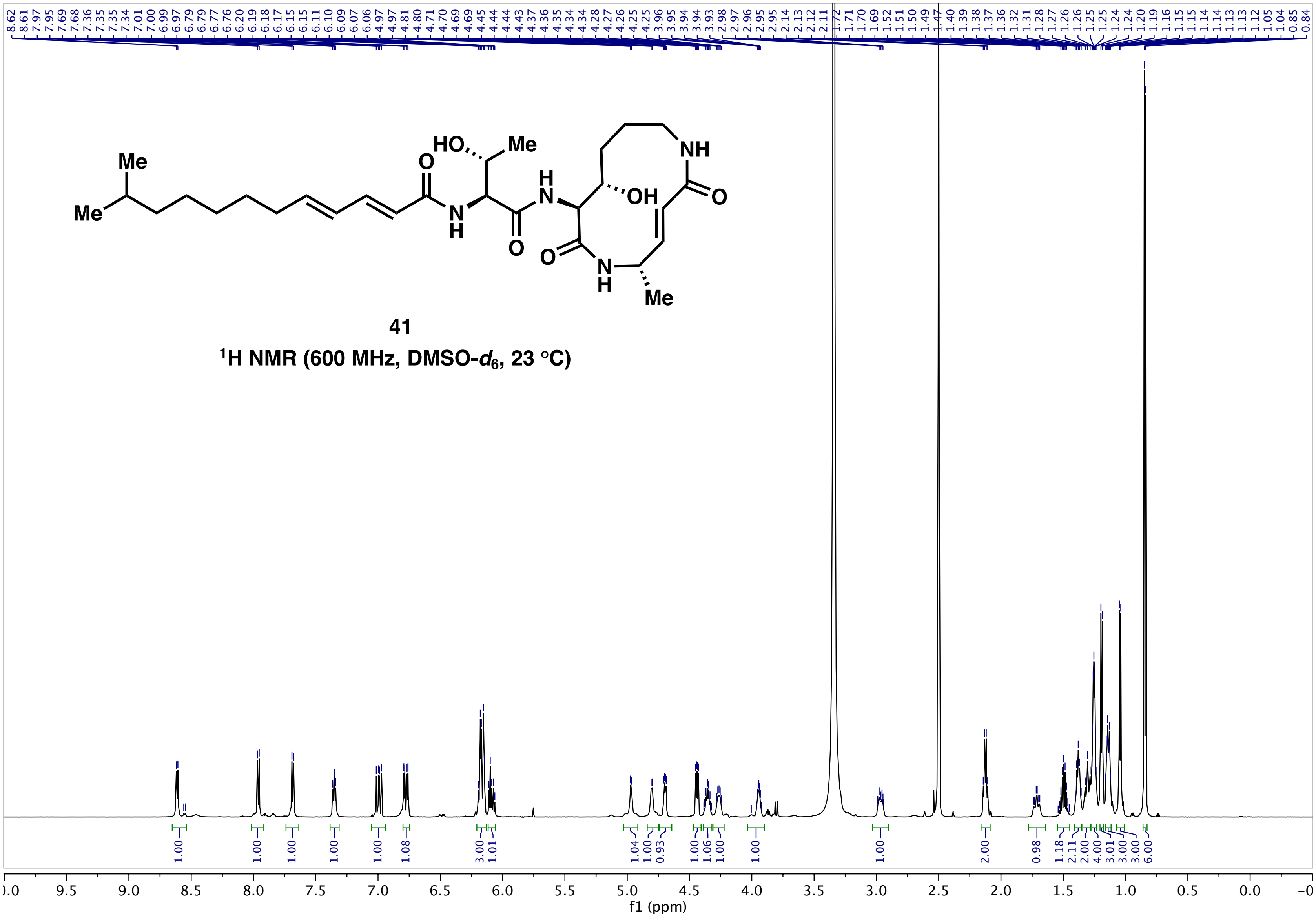

41

^{1}H NMR (600 MHz, DMSO-d_6, 23 °C)

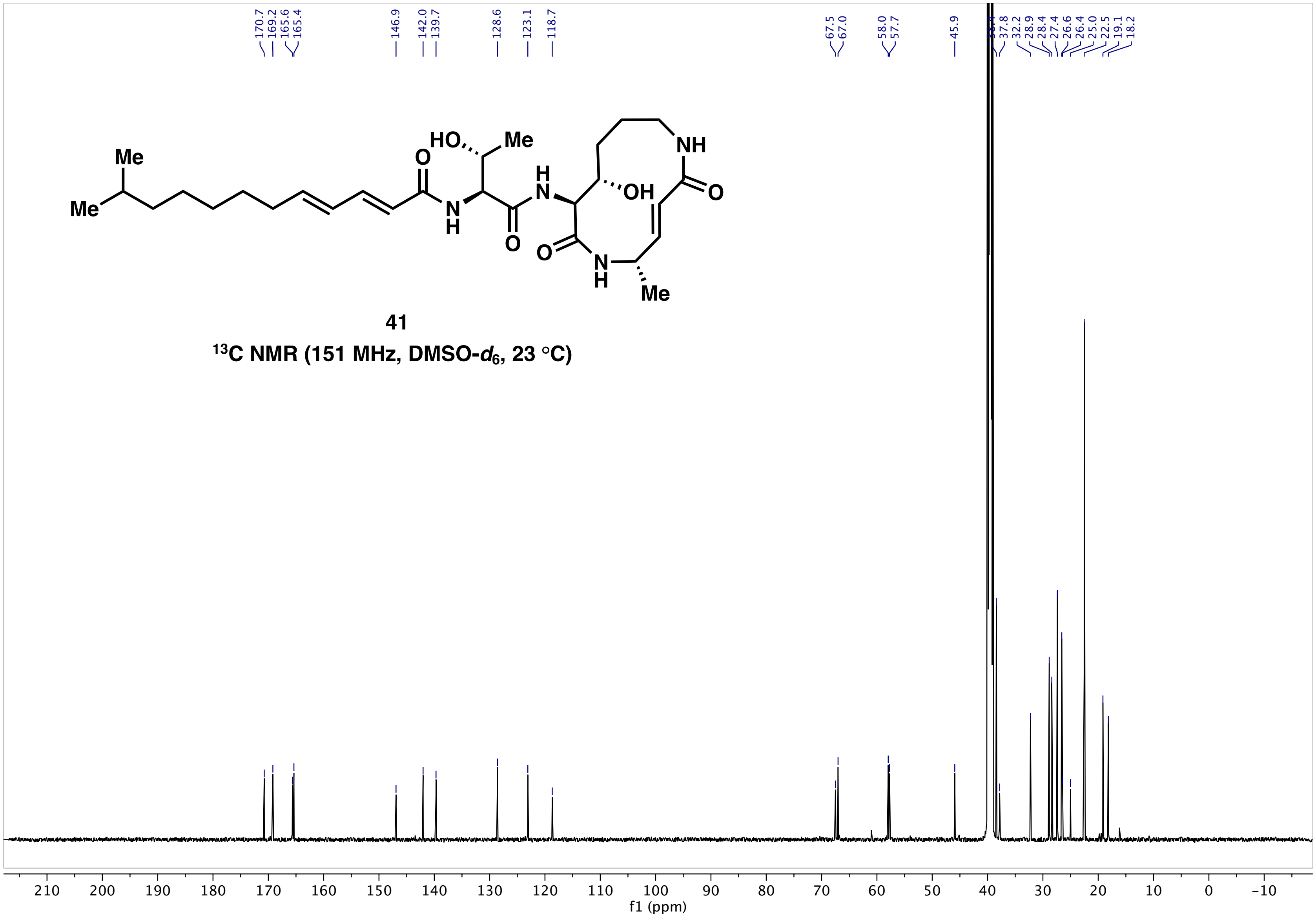
170.7
169.2
165.6
165.4
146.9
142.0
139.7
128.6
123.1
118.7
67.5
67.0
58.0
57.7
45.9
37.8
32.2
28.9
28.4
27.4
26.6
26.4
25.0
22.5
19.1
18.2
Me
Me
HO
Me
O
N
H
H
N
O
OH
NH
O
O
N
H
Me
41
13C NMR (151 MHz, DMSO-d6, 23 °C)
210 200 190 180 170 160 150 140 130 120 110 100 90 80 70 60 50 40 30 20 10 0 -10
f1 (ppm)

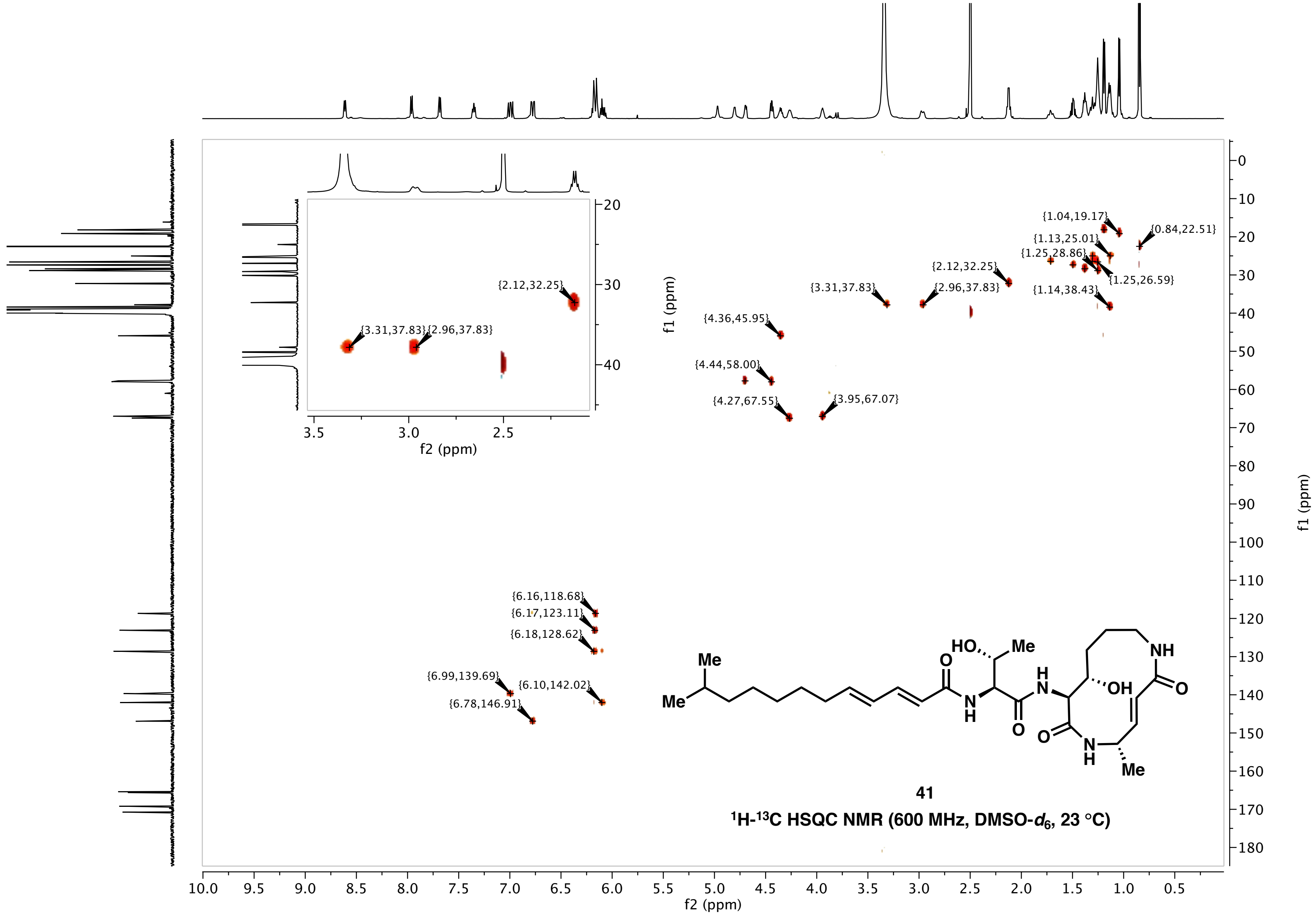

41

^{1}H-^{13}C HSQC NMR (600 MHz, DMSO-d_6, 23 °C)

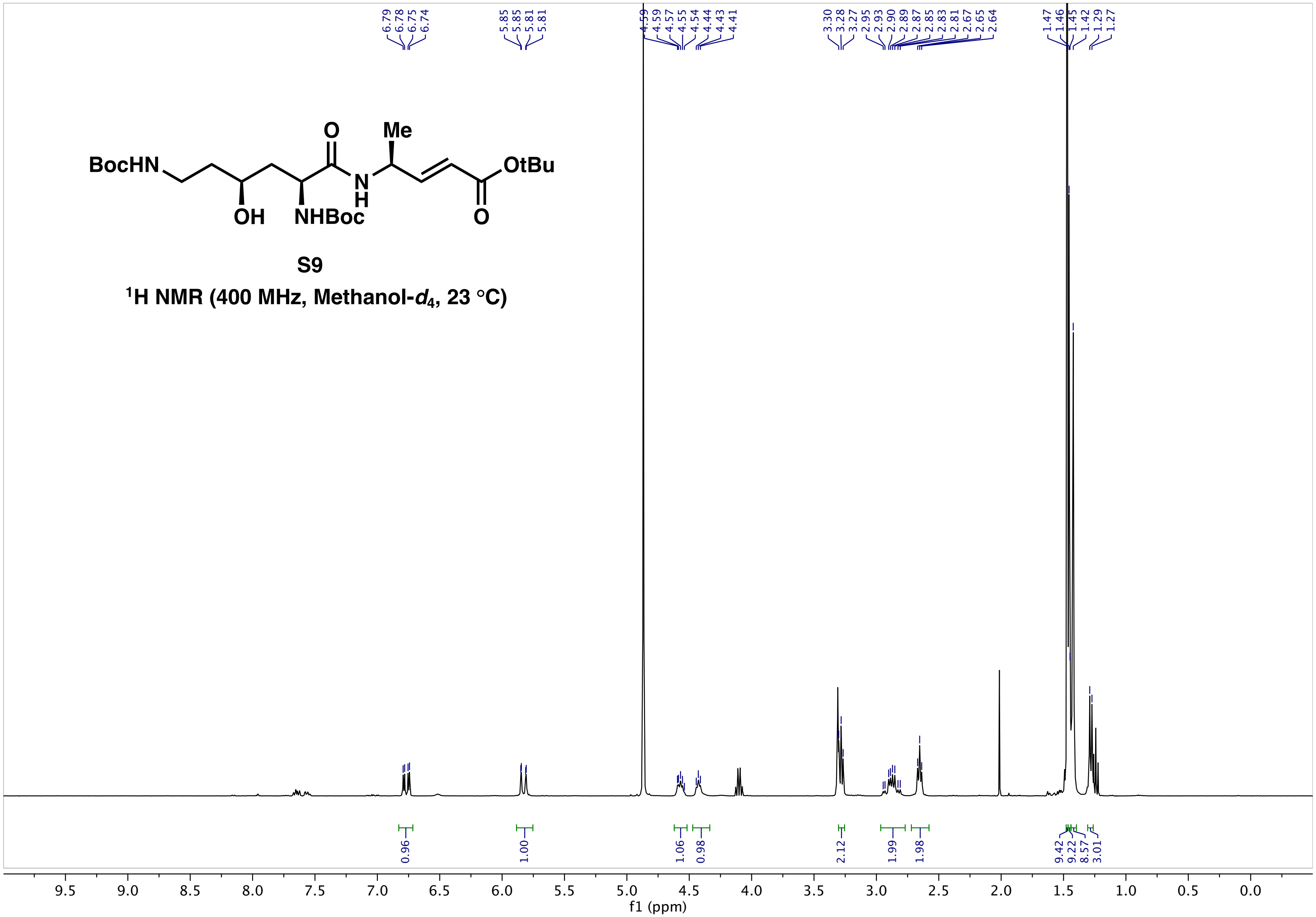

S9

^{1}H NMR (400 MHz, Methanol-d_4, 23 °C)

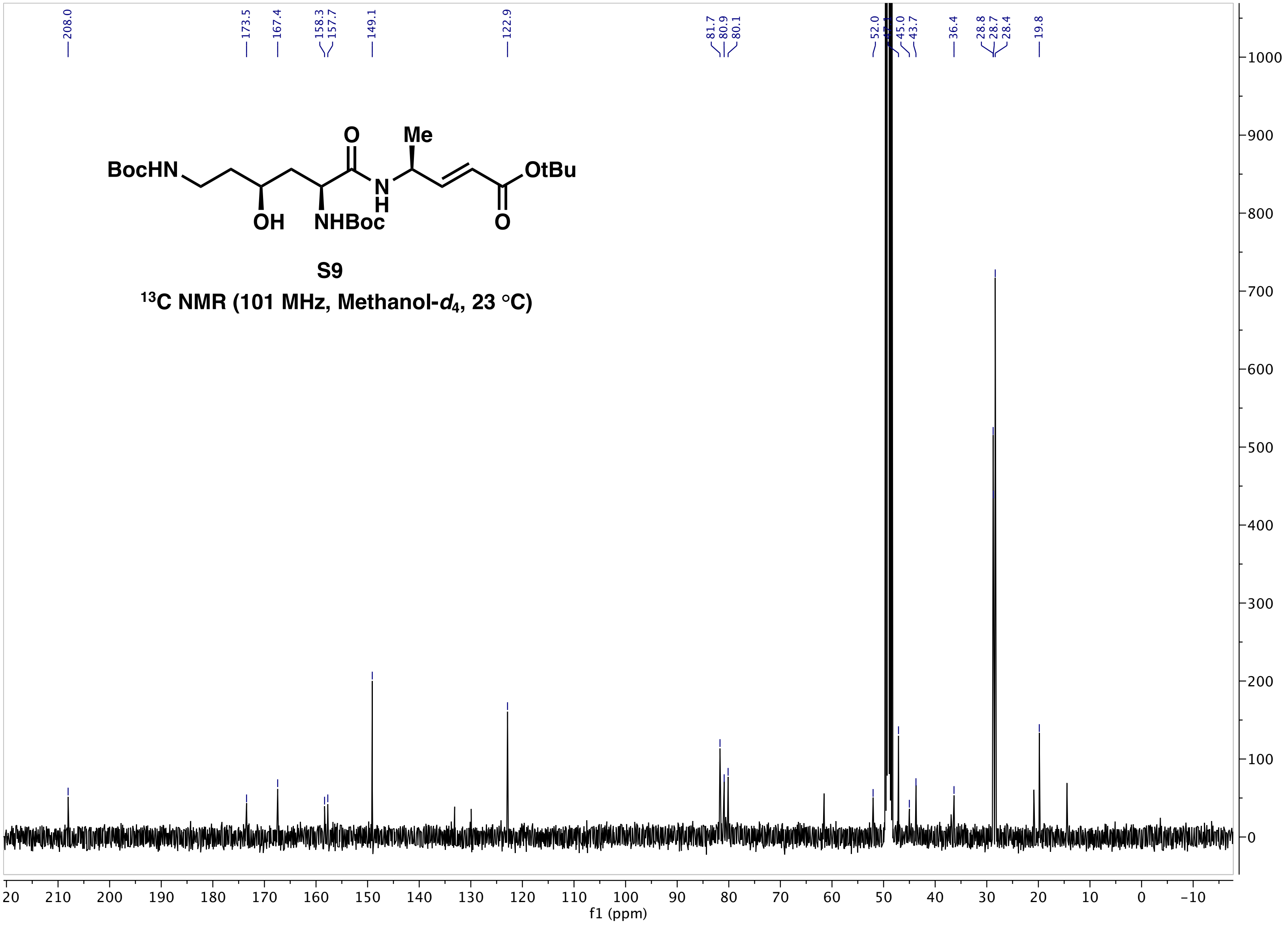

S9

^{13}C NMR (101 MHz, Methanol-d_4, 23 °C)

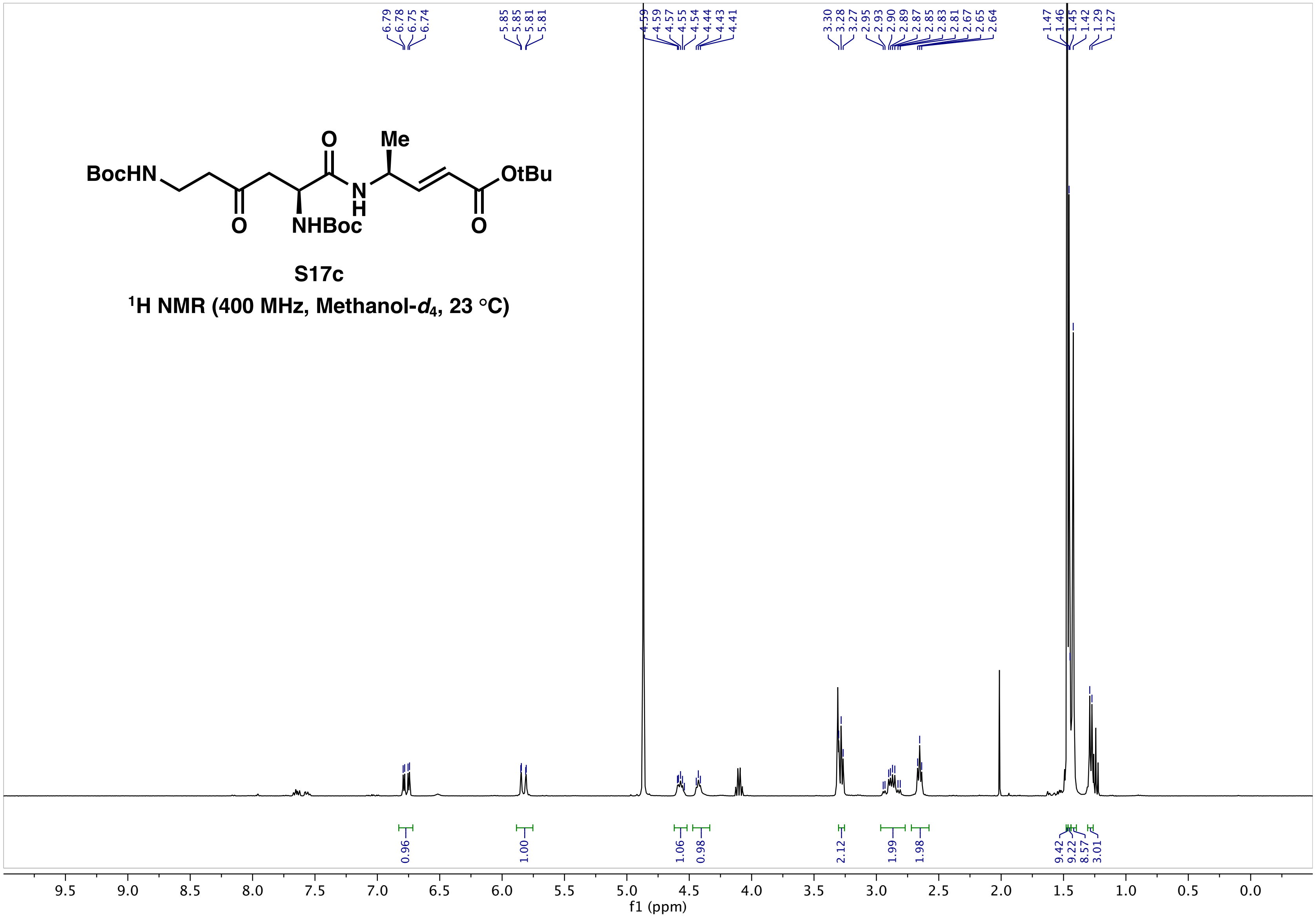

BocHN
O
NHBoc
O
Me
N
H
OtBu
O
S17c
^{1}H NMR (400 MHz, Methanol-d_4, 23 °C)
f1 (ppm)

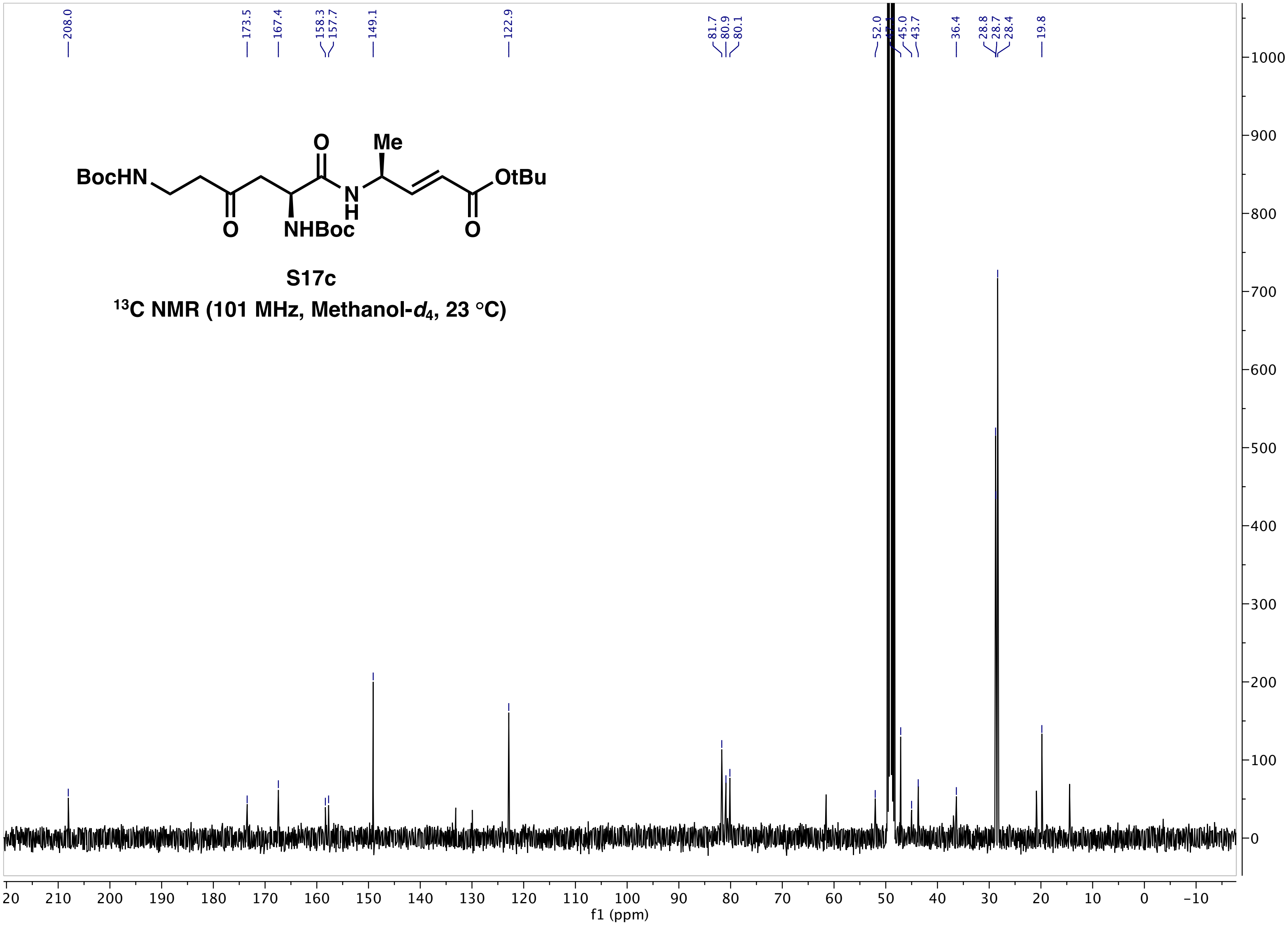

208.0
173.5
167.4
158.3
157.7
149.1
122.9
81.7
80.9
80.1
52.0
45.0
43.7
36.4
28.8
28.7
28.4
19.8
BocHN
O
Me
N
H
OtBu
O
NHBoc
O
S17c
^{13}C NMR (101 MHz, Methanol-d_4, 23 °C)
f1 (ppm)

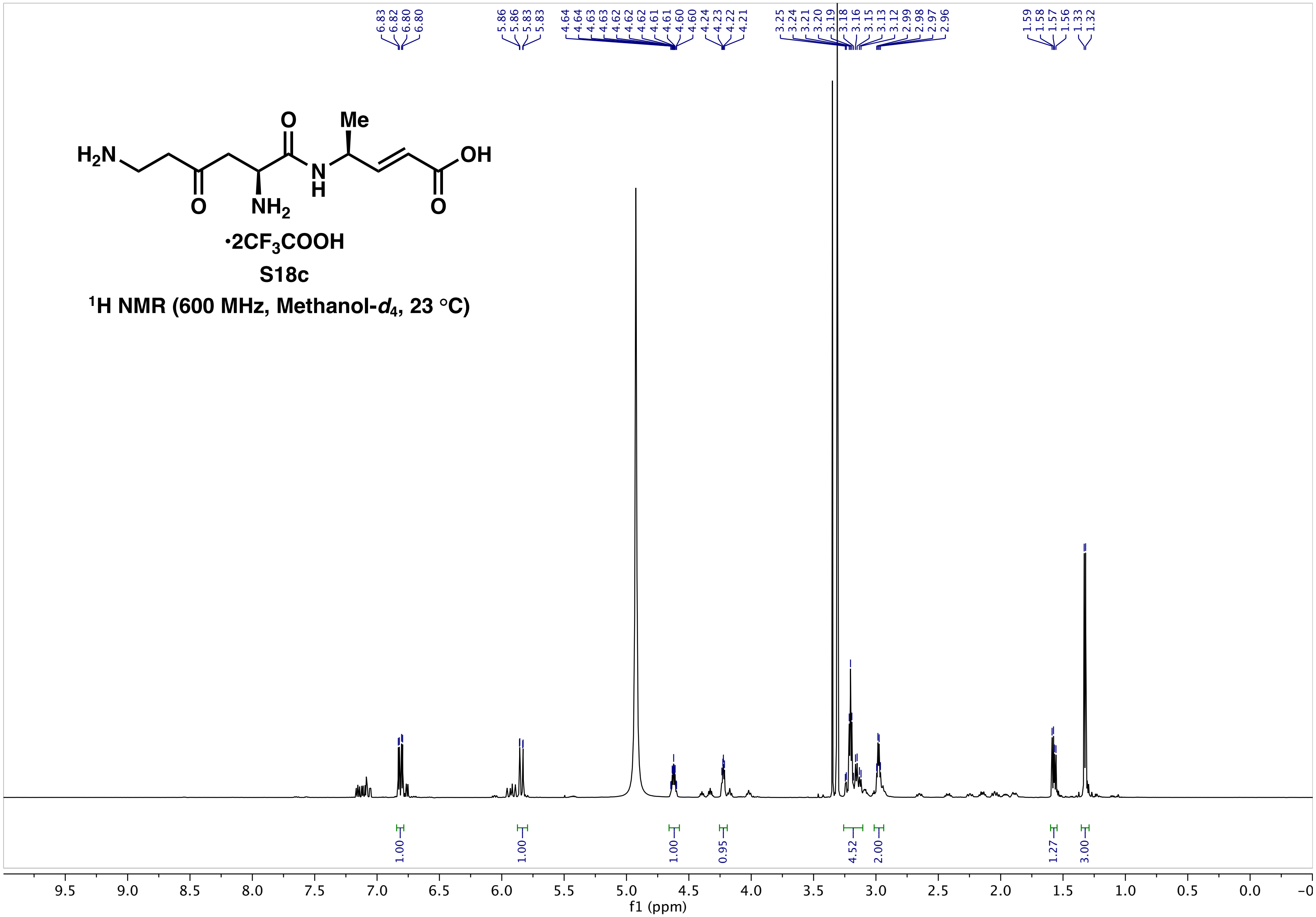

S18c

^{1}H NMR (600 MHz, Methanol-d_4, 23 °C)

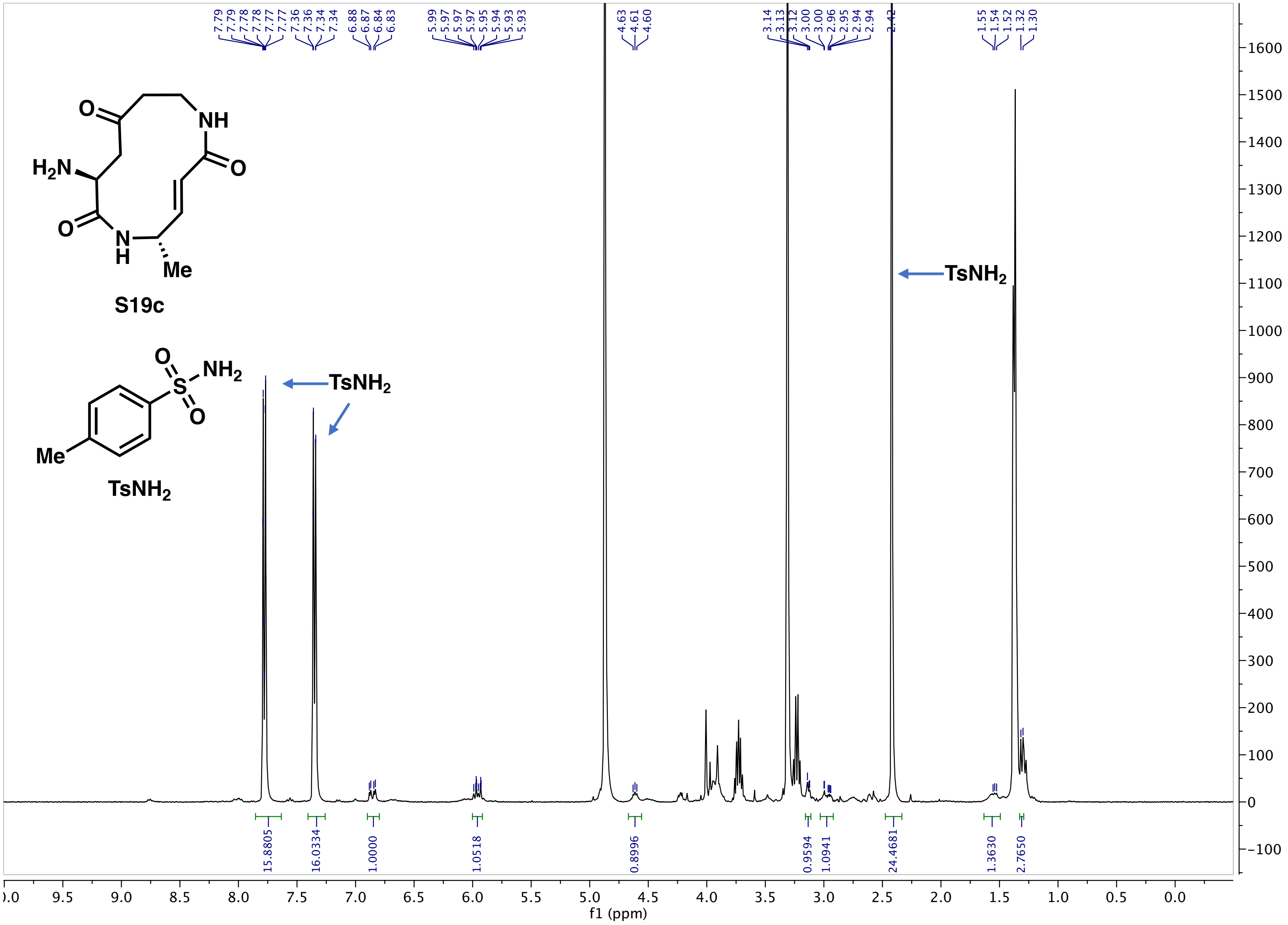
S19c
TsNH2
TsNH2
TsNH2
f1 (ppm)

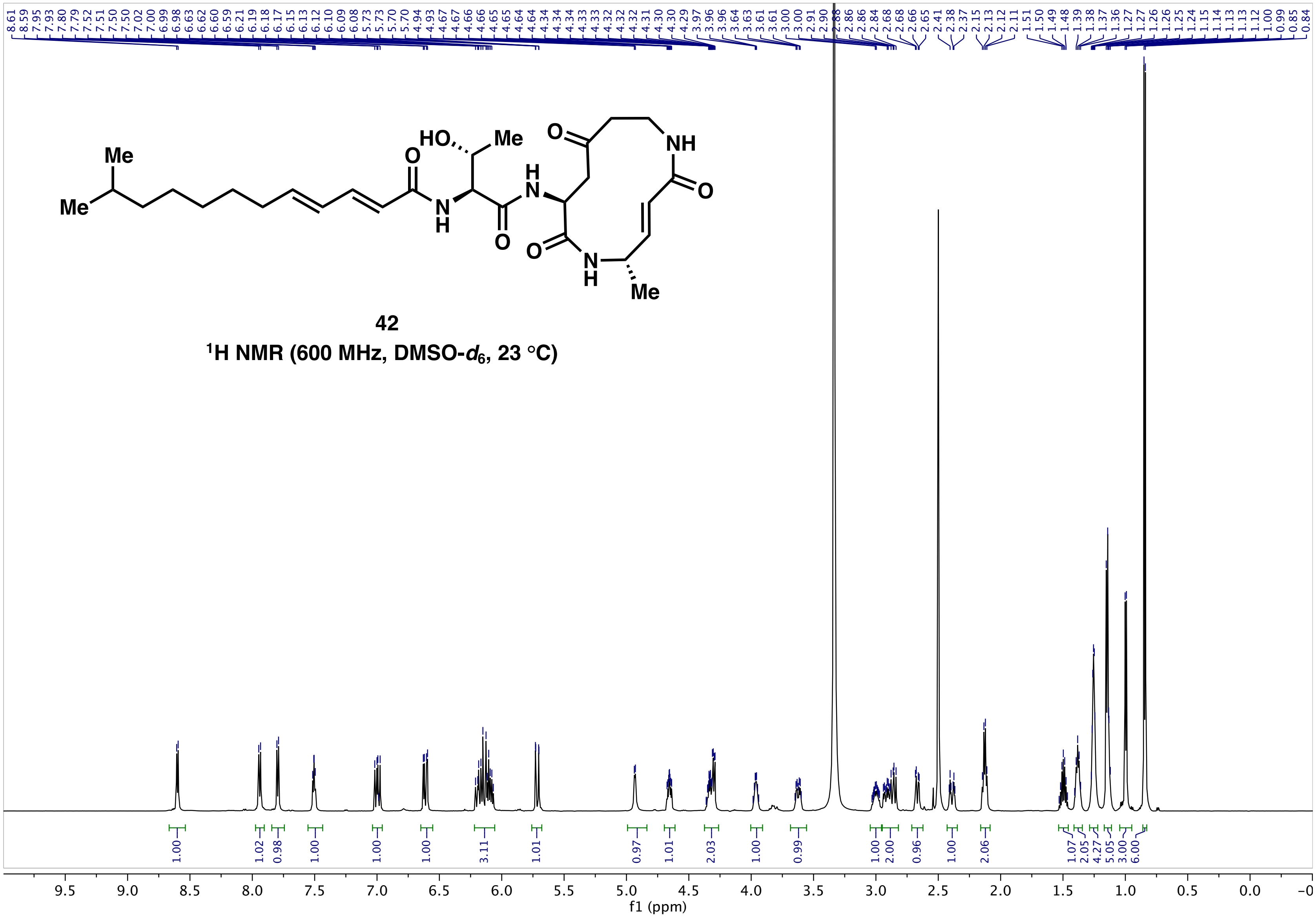

42

^{1}H NMR (600 MHz, DMSO-d_6, 23 °C)

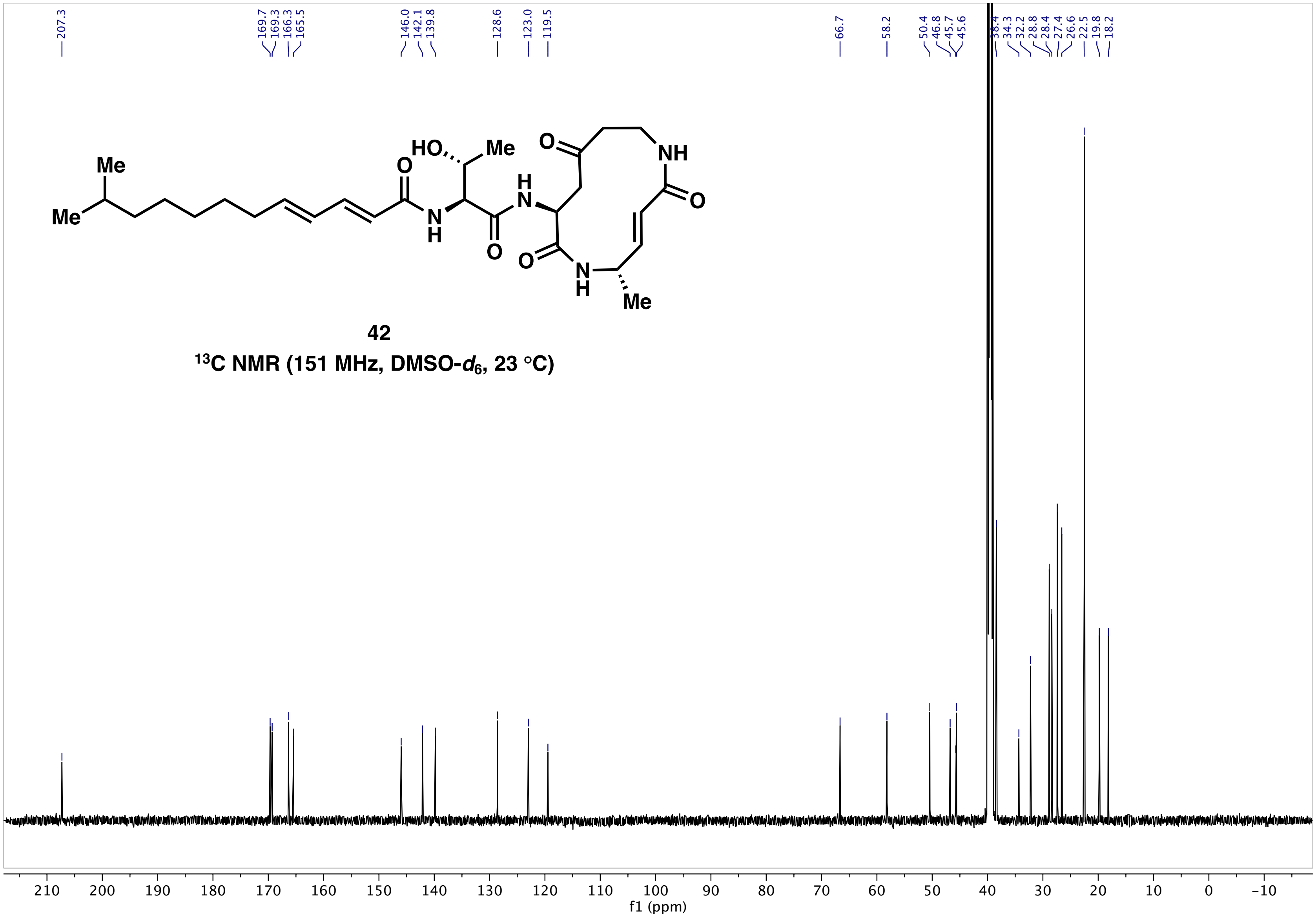
207.3
169.7
169.3
166.3
165.5
146.0
142.1
139.8
128.6
123.0
119.5
66.7
58.2
50.4
46.8
45.7
45.6
38.4
34.3
32.2
28.8
28.4
27.4
26.6
22.5
19.8
18.2
Me
Me
O
N
H
HO
Me
O
H
N
O
NH
O
O
N
H
Me
42
^{13}C NMR (151 MHz, DMSO-d_6, 23 °C)
210 200 190 180 170 160 150 140 130 120 110 100 90 80 70 60 50 40 30 20 10 0 -10
f1 (ppm)

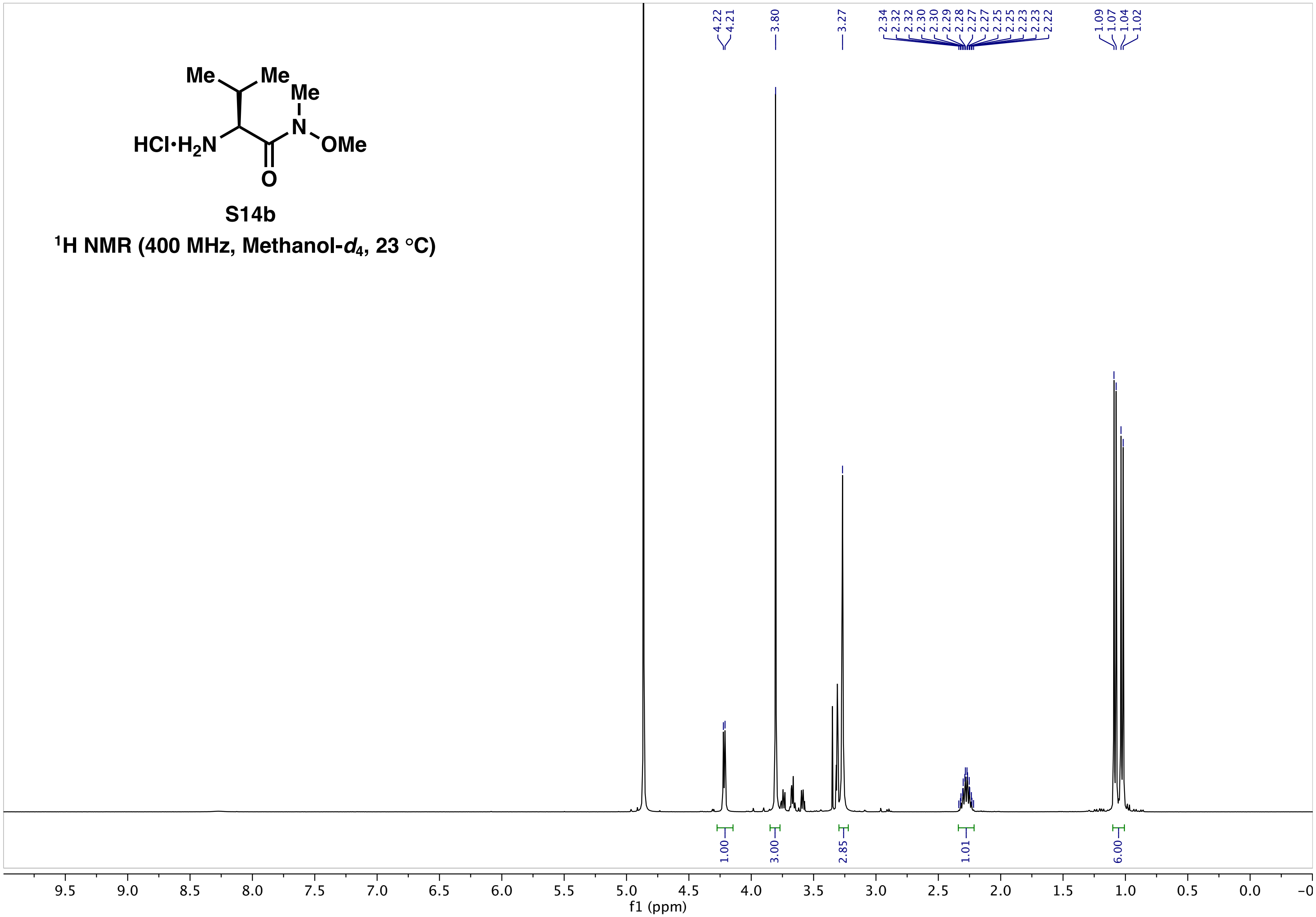

Me
Me
Me
HCl•H2N
N
OMe
O
S14b
1H NMR (400 MHz, Methanol-d4, 23 °C)
4.22
4.21
3.80
3.27
2.34
2.32
2.32
2.30
2.30
2.29
2.28
2.27
2.27
2.25
2.25
2.23
2.23
2.22
1.09
1.07
1.04
1.02
1.00
3.00
2.85
1.01
6.00
9.5
9.0
8.5
8.0
7.5
7.0
6.5
6.0
5.5
5.0
4.5
4.0
3.5
3.0
2.5
2.0
1.5
1.0
0.5
0.0
f1 (ppm)

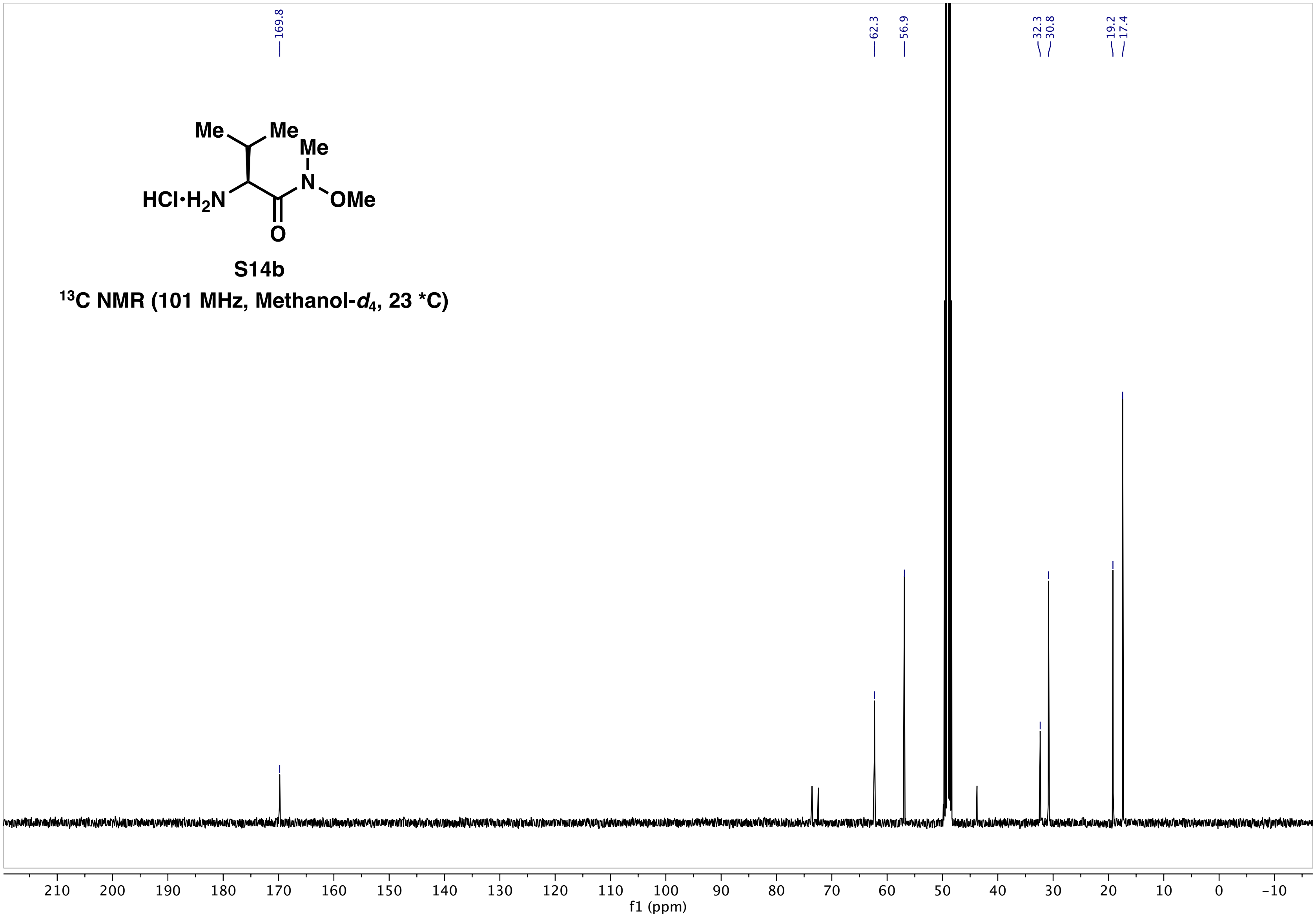

S14b

^{13}C NMR (101 MHz, Methanol-d_4, 23 *C)

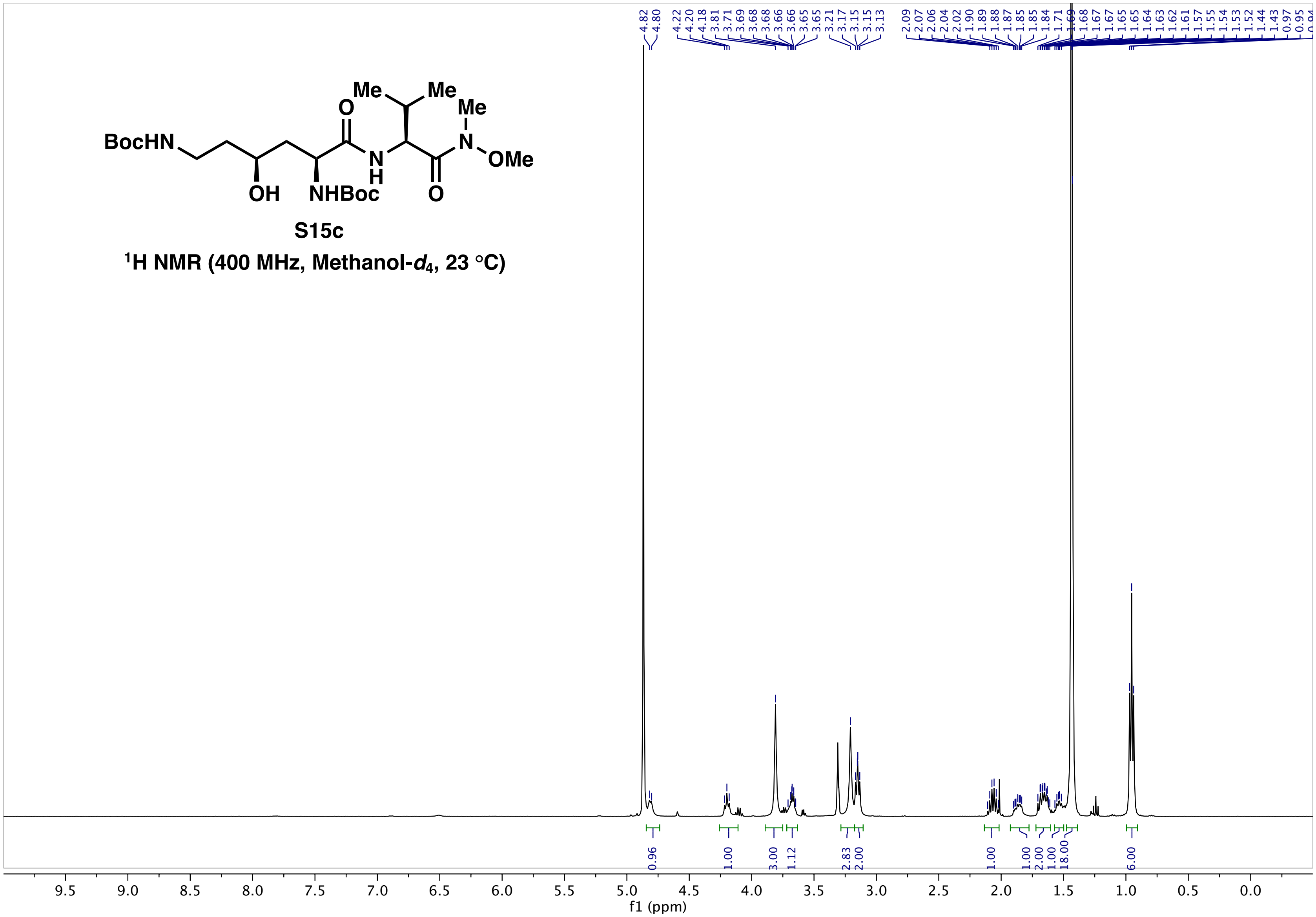
BocHN
OH
NHBoc
O
N
H
Me
Me
Me
N
OMe
O
S15c
^{1}H NMR (400 MHz, Methanol-d_4, 23 °C)
4.82 4.80 4.22 4.20 4.18 3.81 3.71 3.69 3.68 3.68 3.66 3.66 3.65 3.65 3.21 3.17 3.15 3.15 3.13 2.09 2.07 2.06 2.04 2.02 1.90 1.89 1.88 1.87 1.85 1.85 1.84 1.71 1.68 1.67 1.67 1.65 1.65 1.64 1.63 1.63 1.62 1.61 1.57 1.55 1.54 1.53 1.52 1.44 1.43 0.97 0.95
0.96 1.00 3.00 1.12 2.83 2.00 1.00 1.00 2.00 1.00 18.00 6.00
9.5 9.0 8.5 8.0 7.5 7.0 6.5 6.0 5.5 5.0 4.5 4.0 3.5 3.0 2.5 2.0 1.5 1.0 0.5 0.0
f1 (ppm)

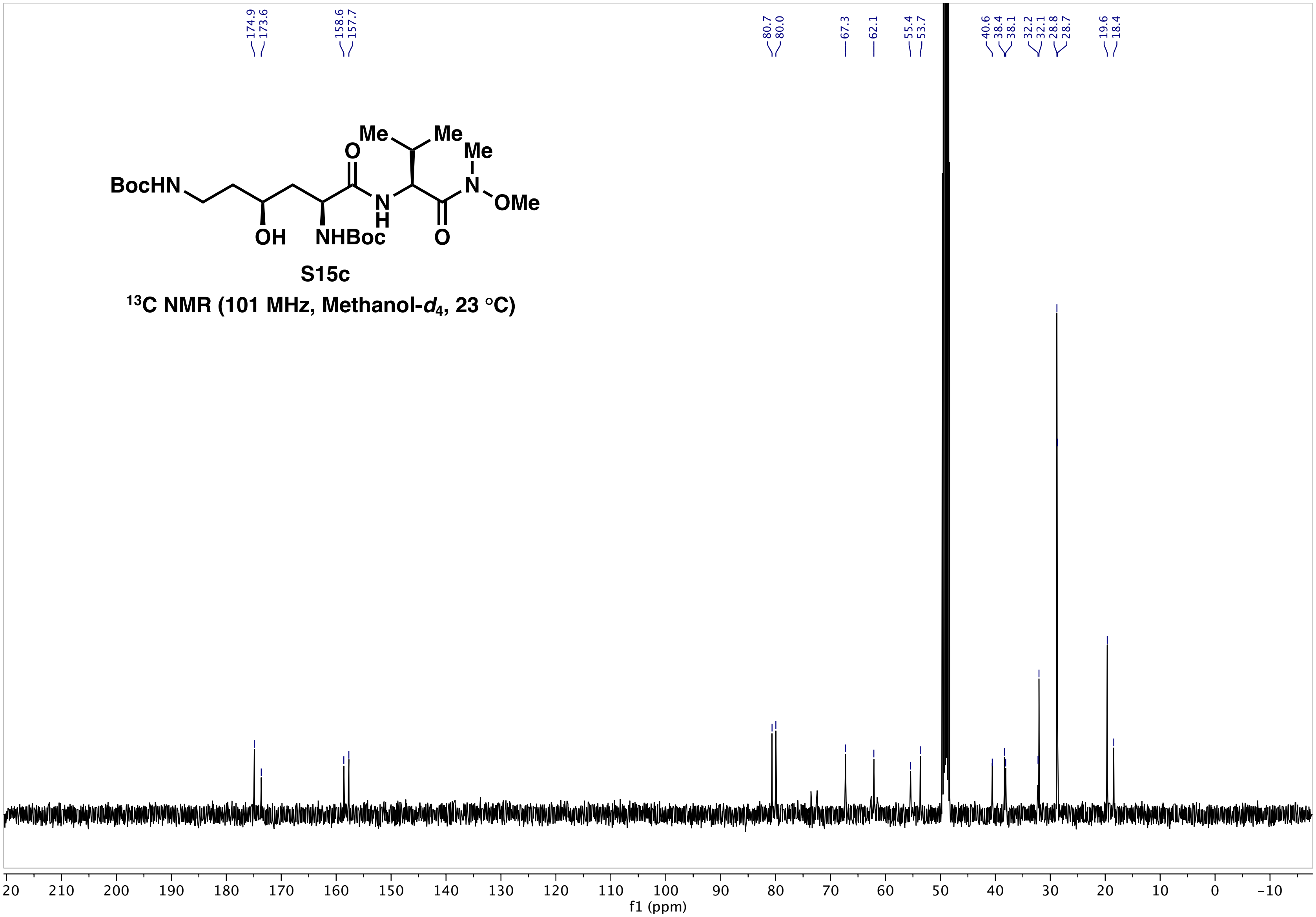

174.9
173.6
158.6
157.7
80.7
80.0
67.3
62.1
55.4
53.7
40.6
38.4
38.1
32.2
32.1
28.8
28.7
19.6
18.4
BocHN
OH
NHBoc
O
N
H
Me
Me
Me
N
OMe
O
S15c
^{13}C NMR (101 MHz, Methanol-d_4, 23 °C)
220 210 200 190 180 170 160 150 140 130 120 110 100 90 80 70 60 50 40 30 20 10 0 −10
f1 (ppm)

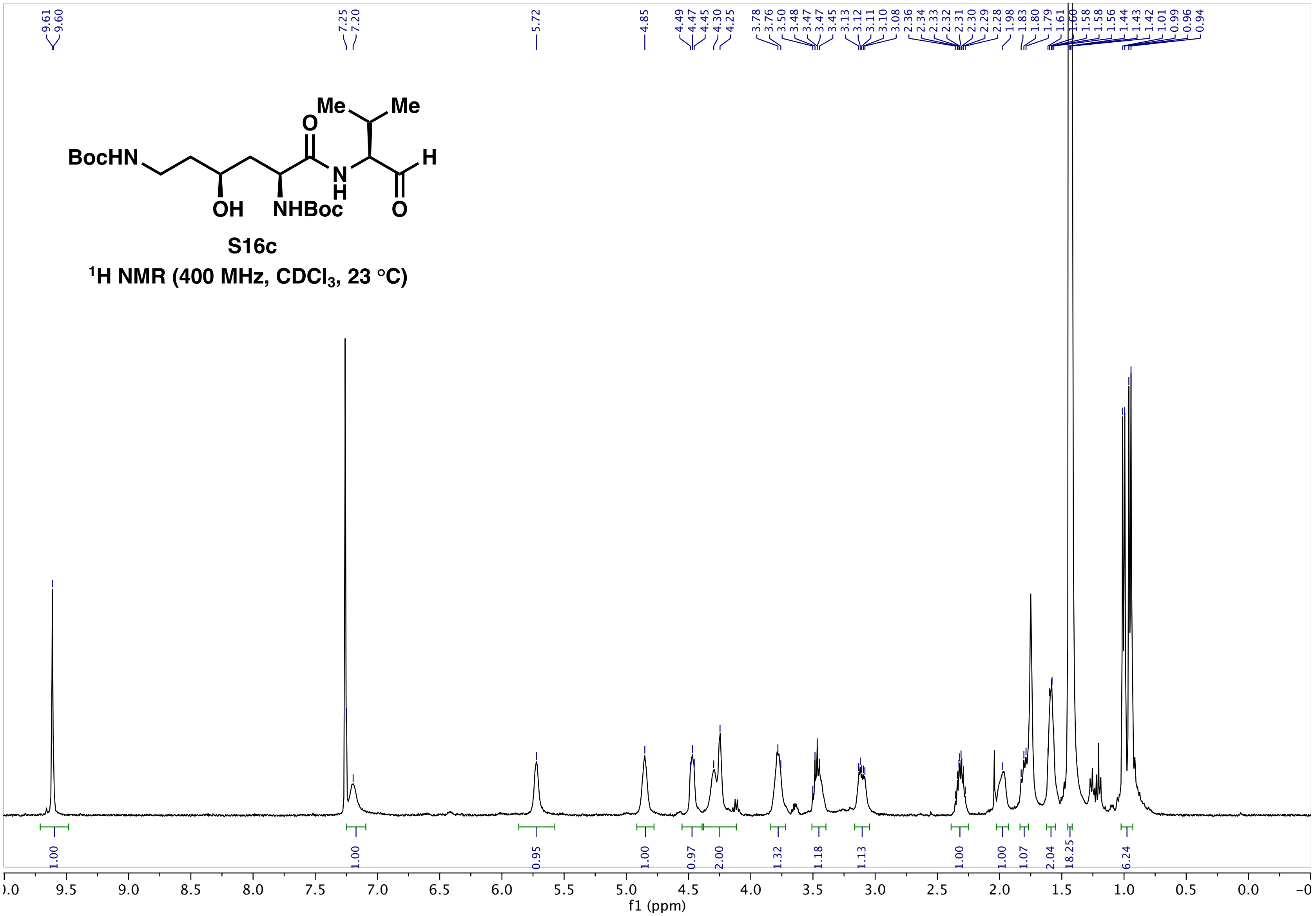
BocHN
OH
NHBoc
N
H
O
Me
Me
H
O
S16c
¹H NMR (400 MHz, CDCl₃, 23 °C)
f1 (ppm)

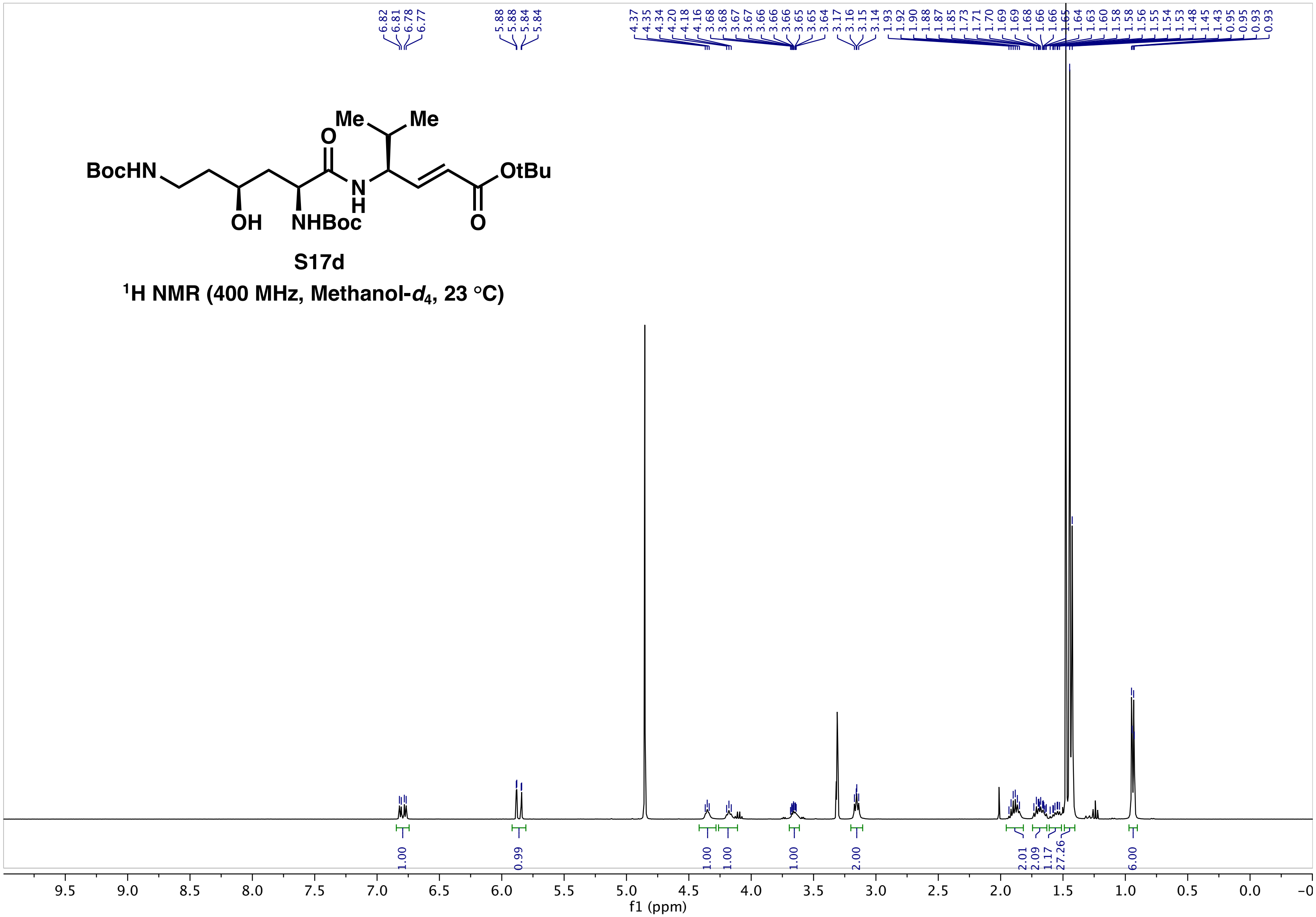

BocHN
OH
NHBoc
O
N
H
Me
Me
OtBu
O
S17d
¹H NMR (400 MHz, Methanol-d4, 23 °C)
f1 (ppm)

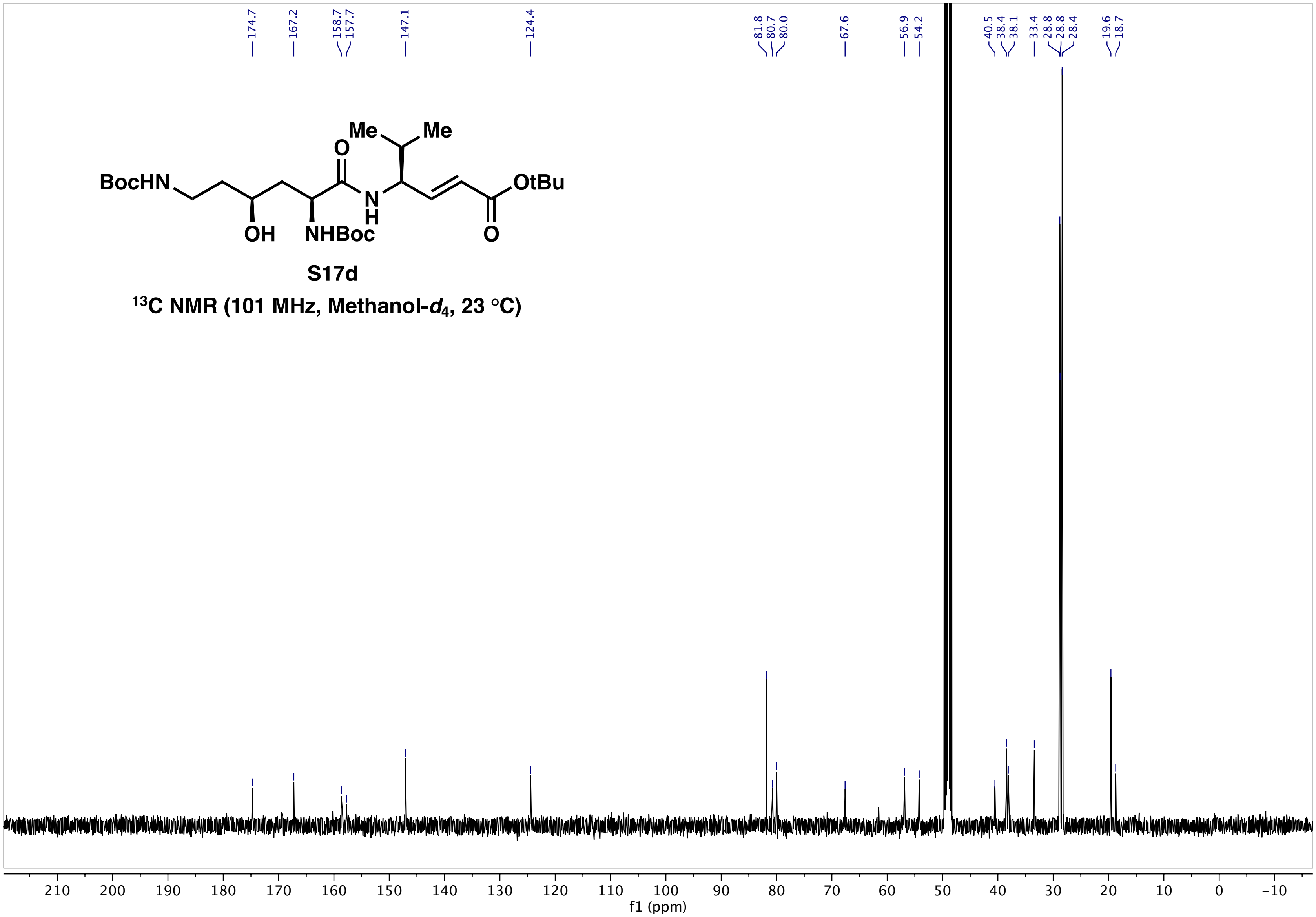

S17d

^{13}C NMR (101 MHz, Methanol-d_4, 23 °C)

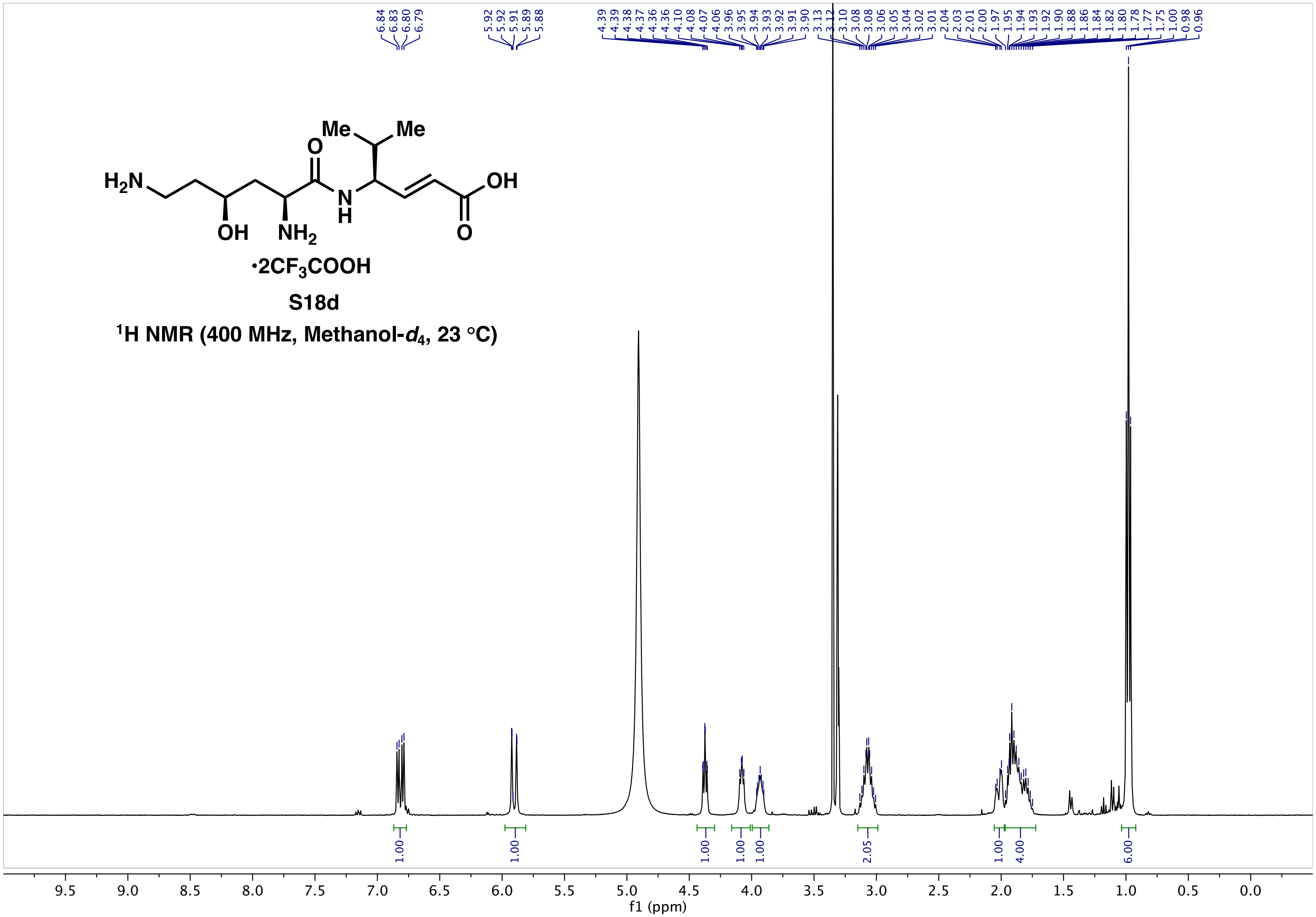

H2N
OH
NH2
O
Me
Me
N
H
OH
O
•2CF3COOH
S18d
1H NMR (400 MHz, Methanol-d4, 23 °C)
6.84
6.83
6.80
6.79
5.92
5.92
5.91
5.89
5.88
4.39
4.39
4.38
4.37
4.36
4.36
4.10
4.08
4.07
4.06
3.96
3.95
3.94
3.93
3.92
3.91
3.90
3.13
3.12
3.10
3.08
3.08
3.06
3.05
3.04
3.02
3.01
2.04
2.03
2.01
2.00
1.97
1.95
1.94
1.93
1.92
1.90
1.88
1.86
1.84
1.82
1.80
1.78
1.77
1.75
1.00
0.98
0.96
1.00
1.00
1.00
1.00
1.00
2.05
1.00
4.00
6.00
9.5
9.0
8.5
8.0
7.5
7.0
6.5
6.0
5.5
5.0
4.5
4.0
3.5
3.0
2.5
2.0
1.5
1.0
0.5
0.0
f1 (ppm)

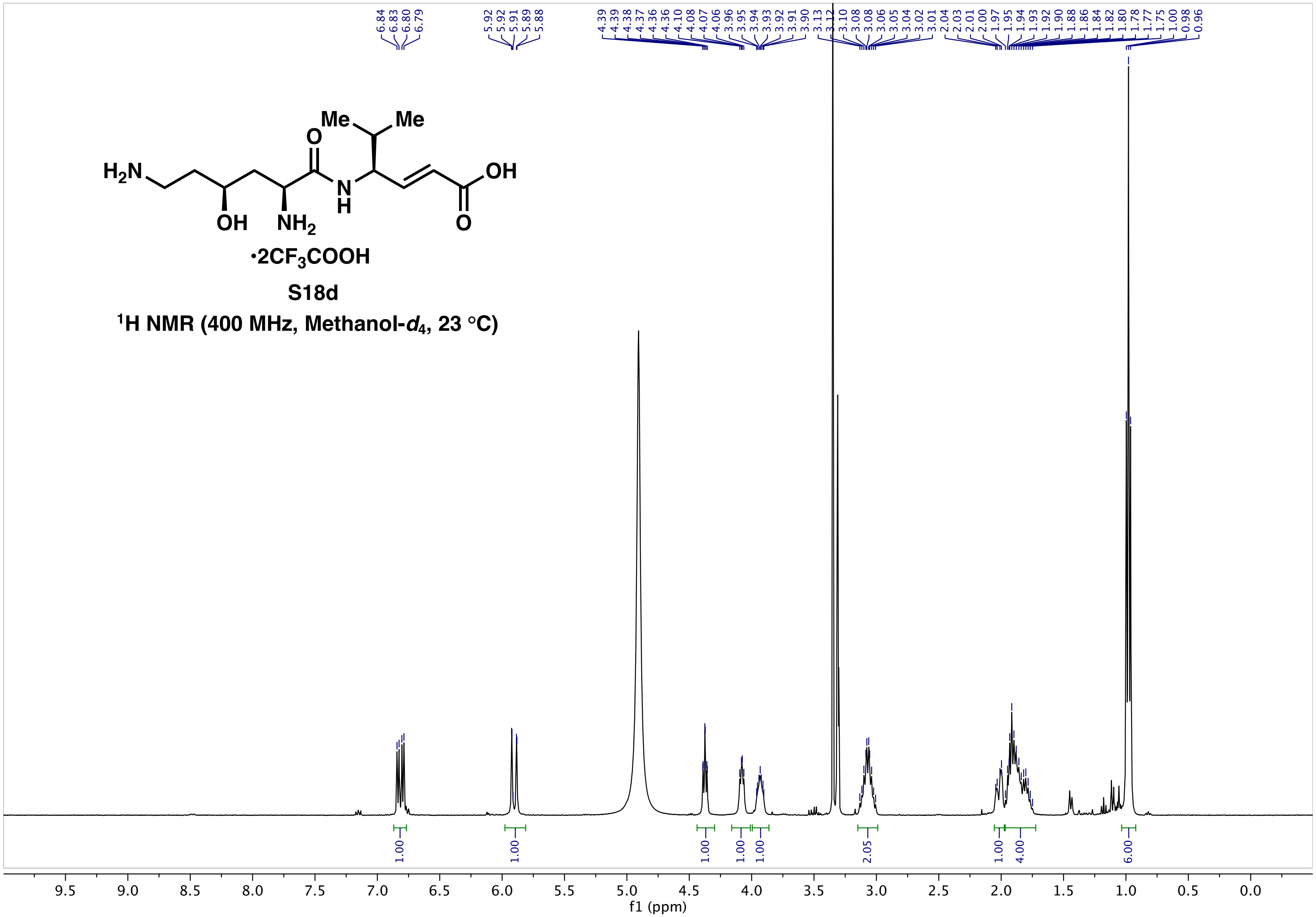

H2N
OH
NH2
O
Me
Me
N
H
OH
O
•2CF3COOH
S18d
1H NMR (400 MHz, Methanol-d4, 23 °C)
f1 (ppm)

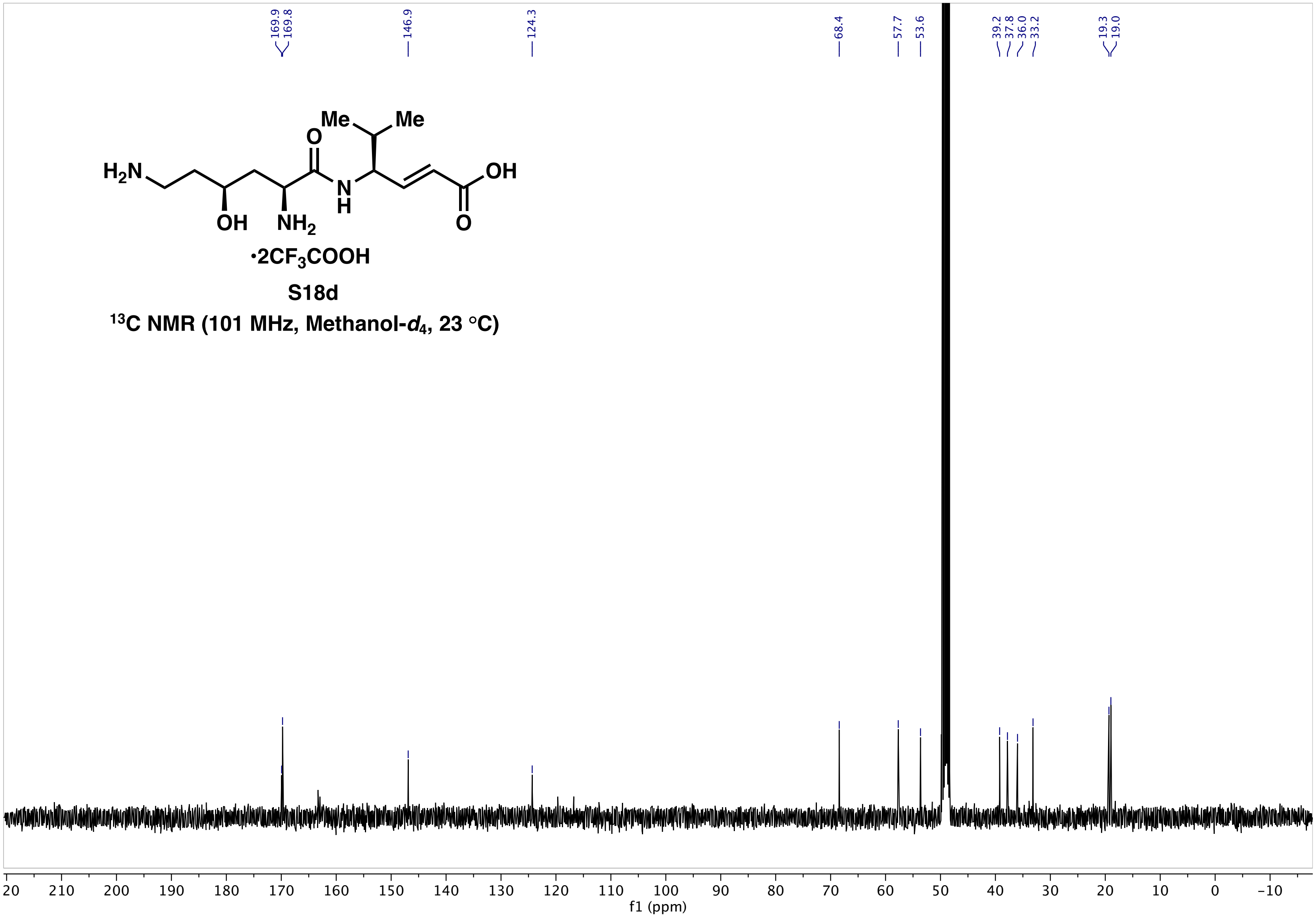
169.9
169.8
146.9
124.3
68.4
57.7
53.6
39.2
37.8
36.0
33.2
19.3
19.0
Me
Me
O
H_2N
OH
NH_2
N
H
OH
O
$\cdot 2CF_3COOH$
S18d
^{13}C NMR (101 MHz, Methanol-d_4, 23 °C)
f1 (ppm)

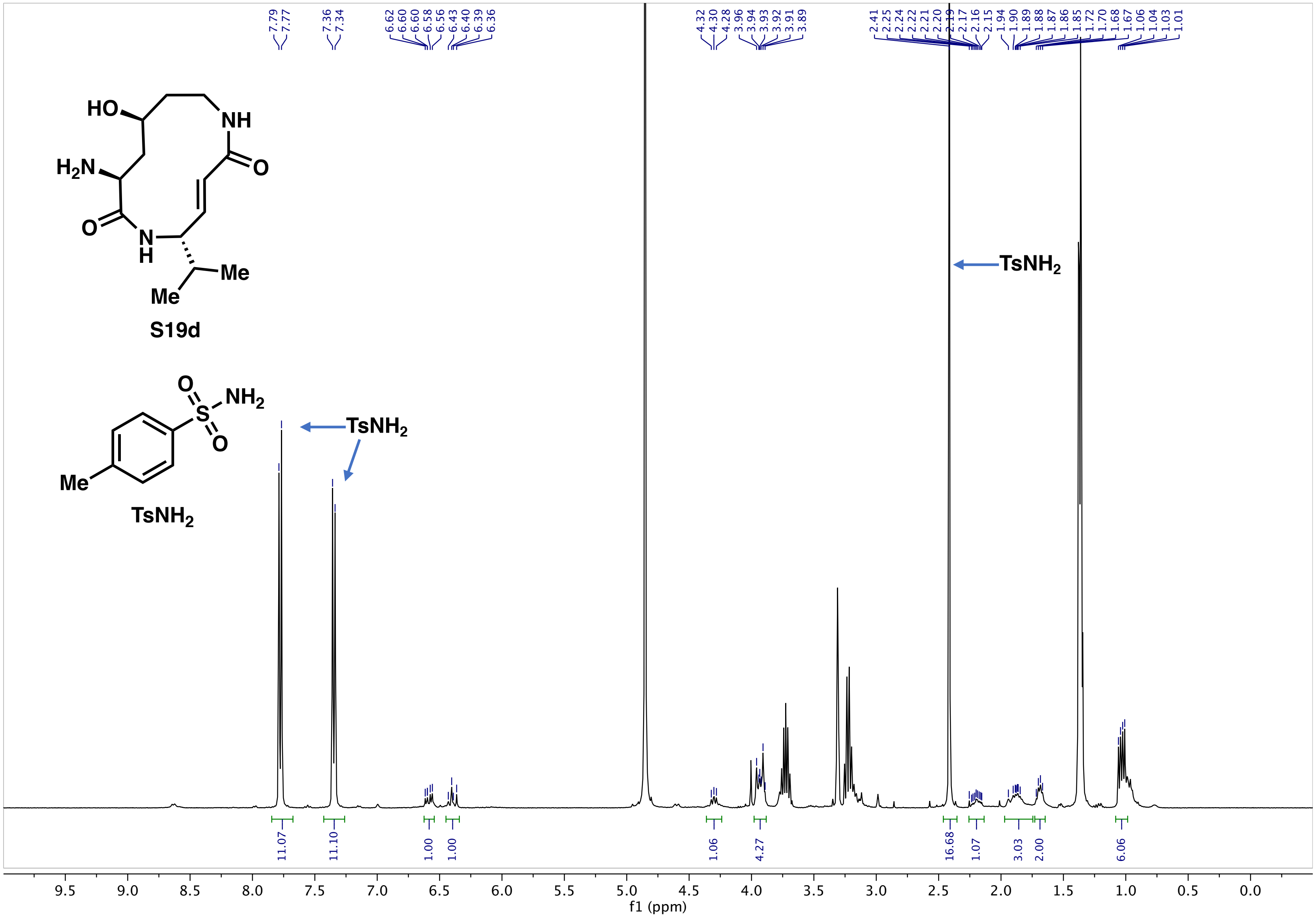

S19d
TsNH2
f1 (ppm)

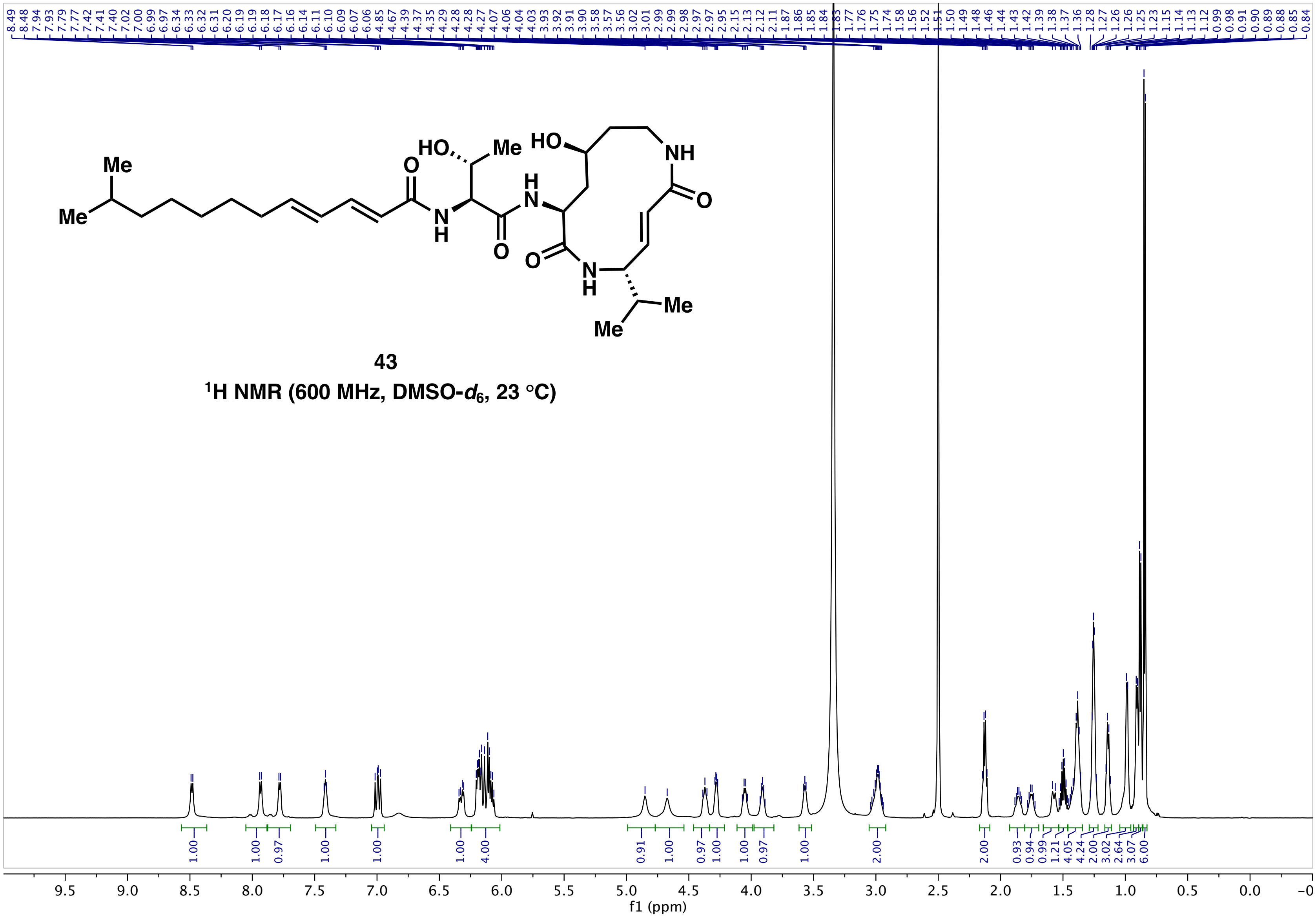
43
1H NMR (600 MHz, DMSO-d6, 23 °C)
f1 (ppm)

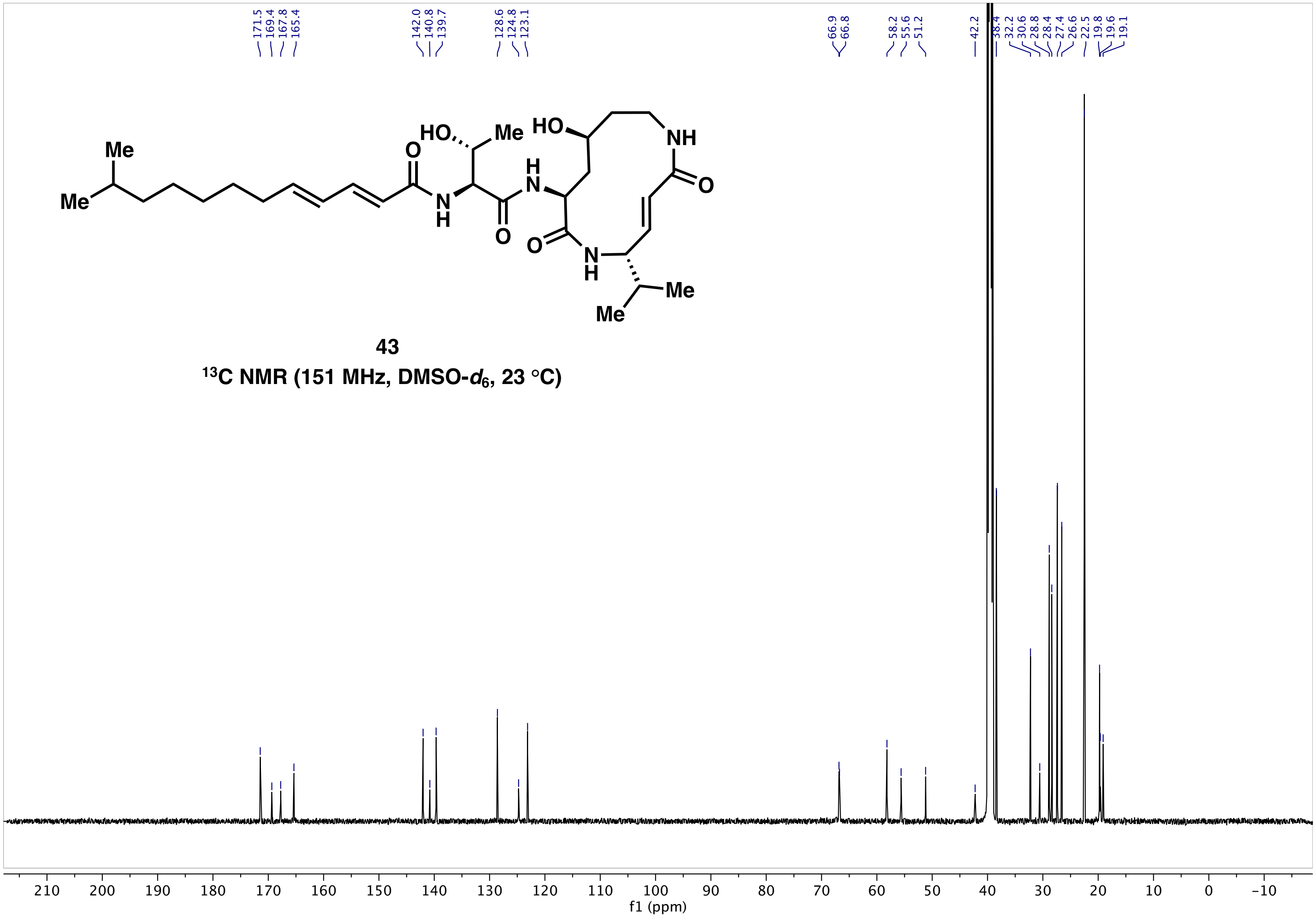
171.5
169.4
167.8
165.4
142.0
140.8
139.7
128.6
124.8
123.1
66.9
66.8
58.2
55.6
51.2
42.2
38.4
32.2
30.6
28.8
28.4
27.4
26.6
22.5
19.8
19.6
19.1
43
^{13}C NMR (151 MHz, DMSO-d_6, 23 °C)
f1 (ppm)

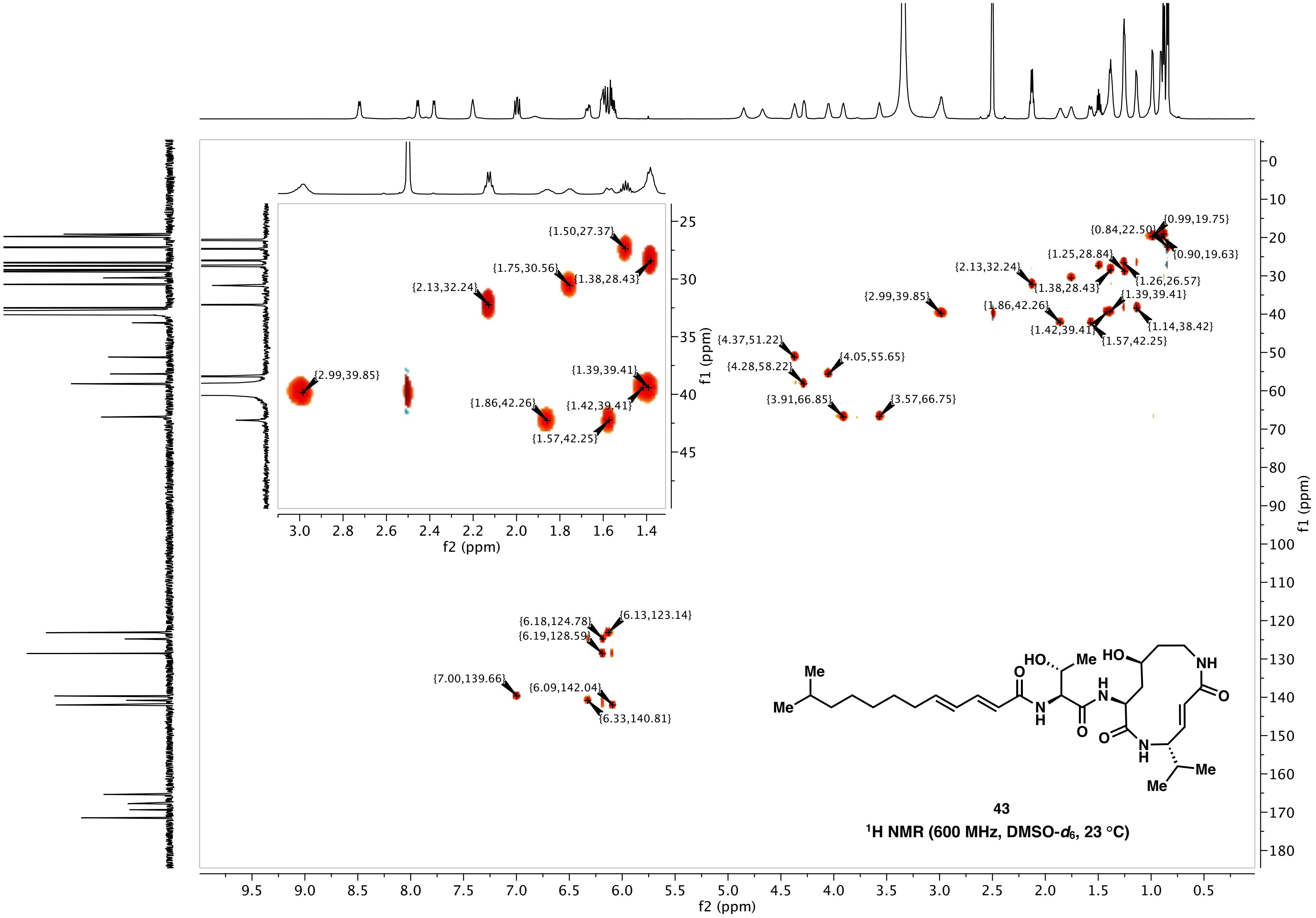

43

^{1}H NMR (600 MHz, DMSO-d_6, 23 °C)

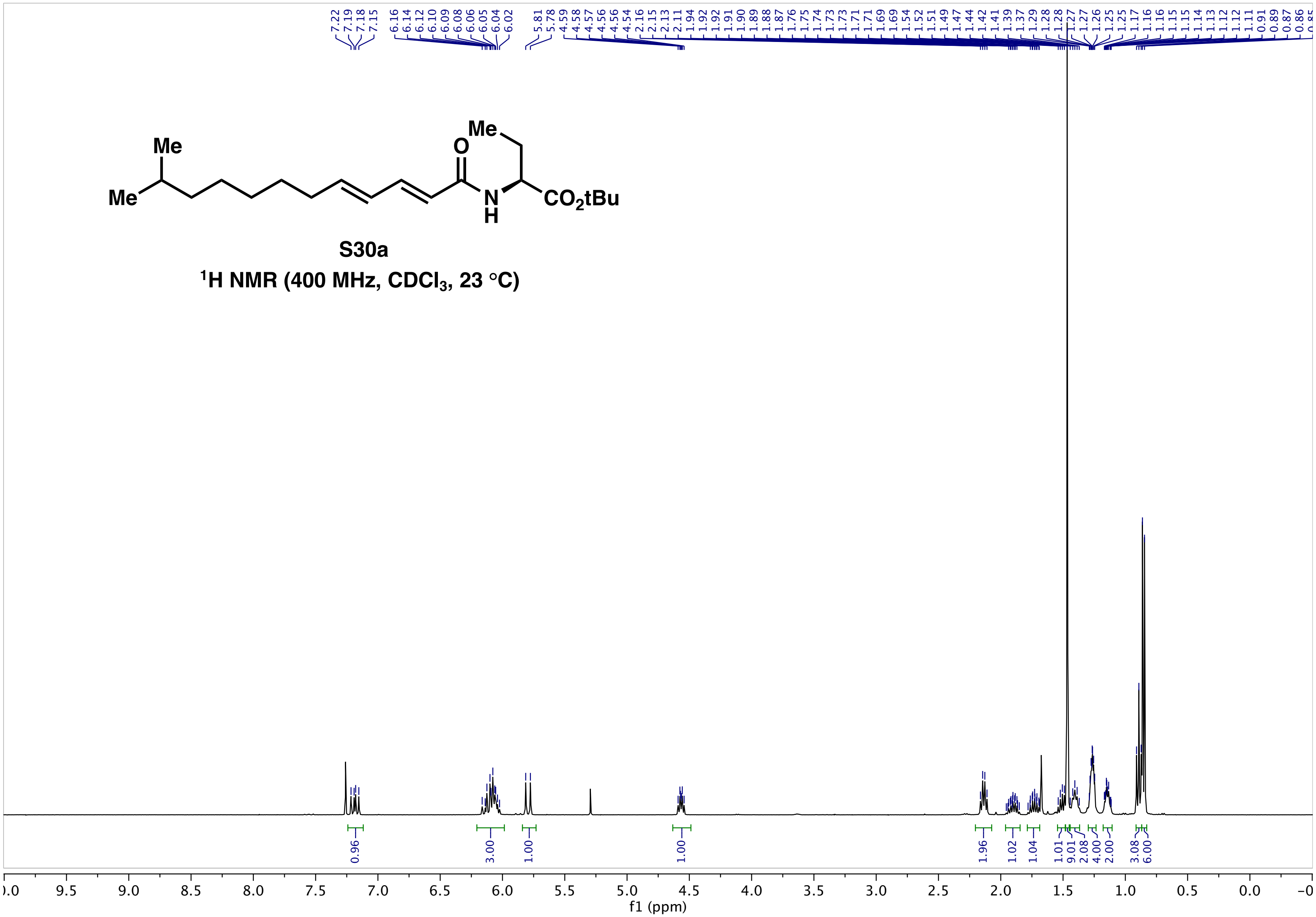
Me
Me
O
Me
N
H
CO2tBu
S30a
1H NMR (400 MHz, CDCl3, 23 °C)
f1 (ppm)

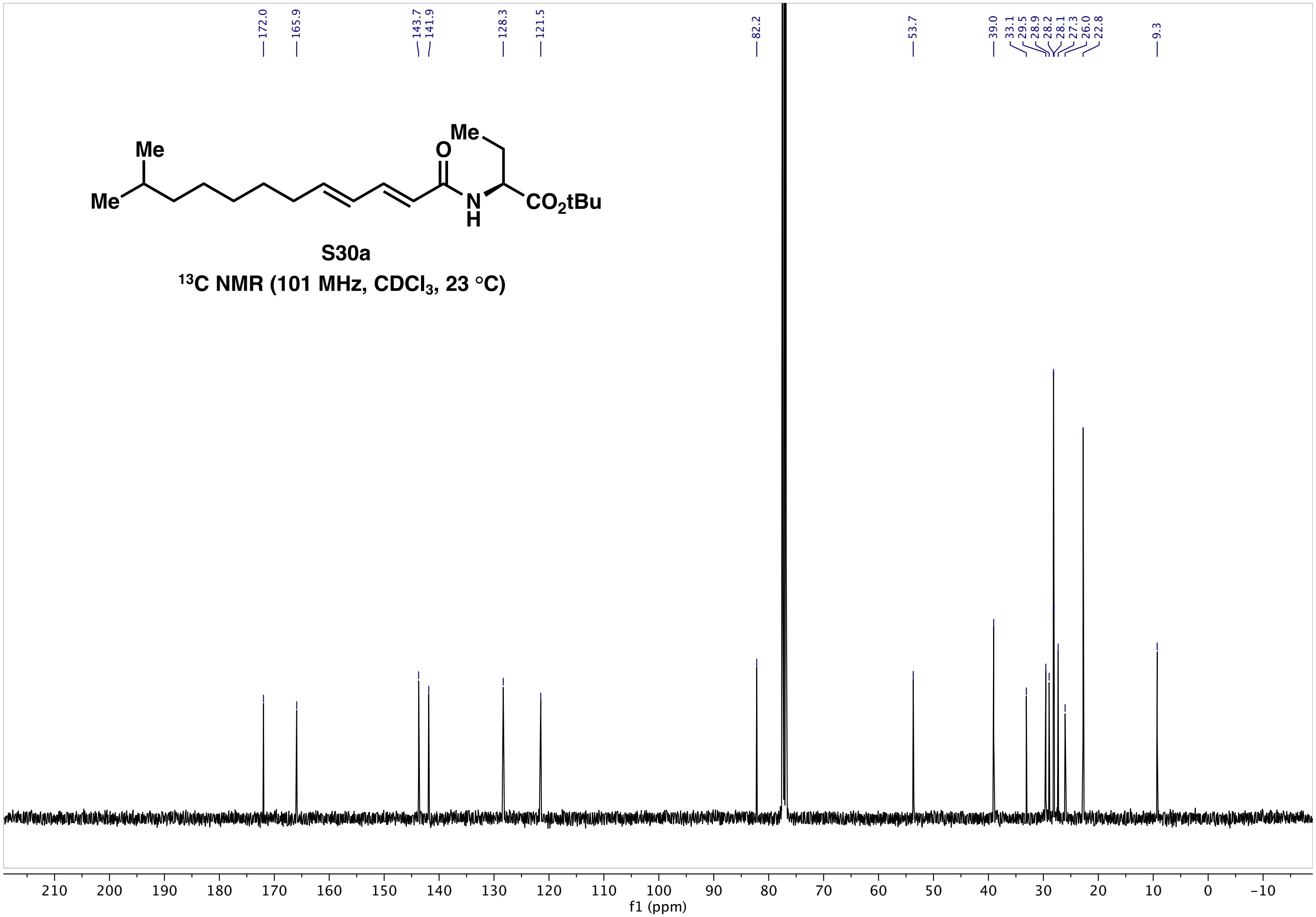

172.0
165.9
143.7
141.9
128.3
121.5
82.2
53.7
39.0
33.1
29.5
28.9
28.2
28.1
27.3
26.0
22.8
9.3
Me
Me
Me
O
N
H
CO2tBu
S30a
13C NMR (101 MHz, CDCl3, 23 °C)
210
200
190
180
170
160
150
140
130
120
110
100
90
80
70
60
50
40
30
20
10
0
–10
f1 (ppm)

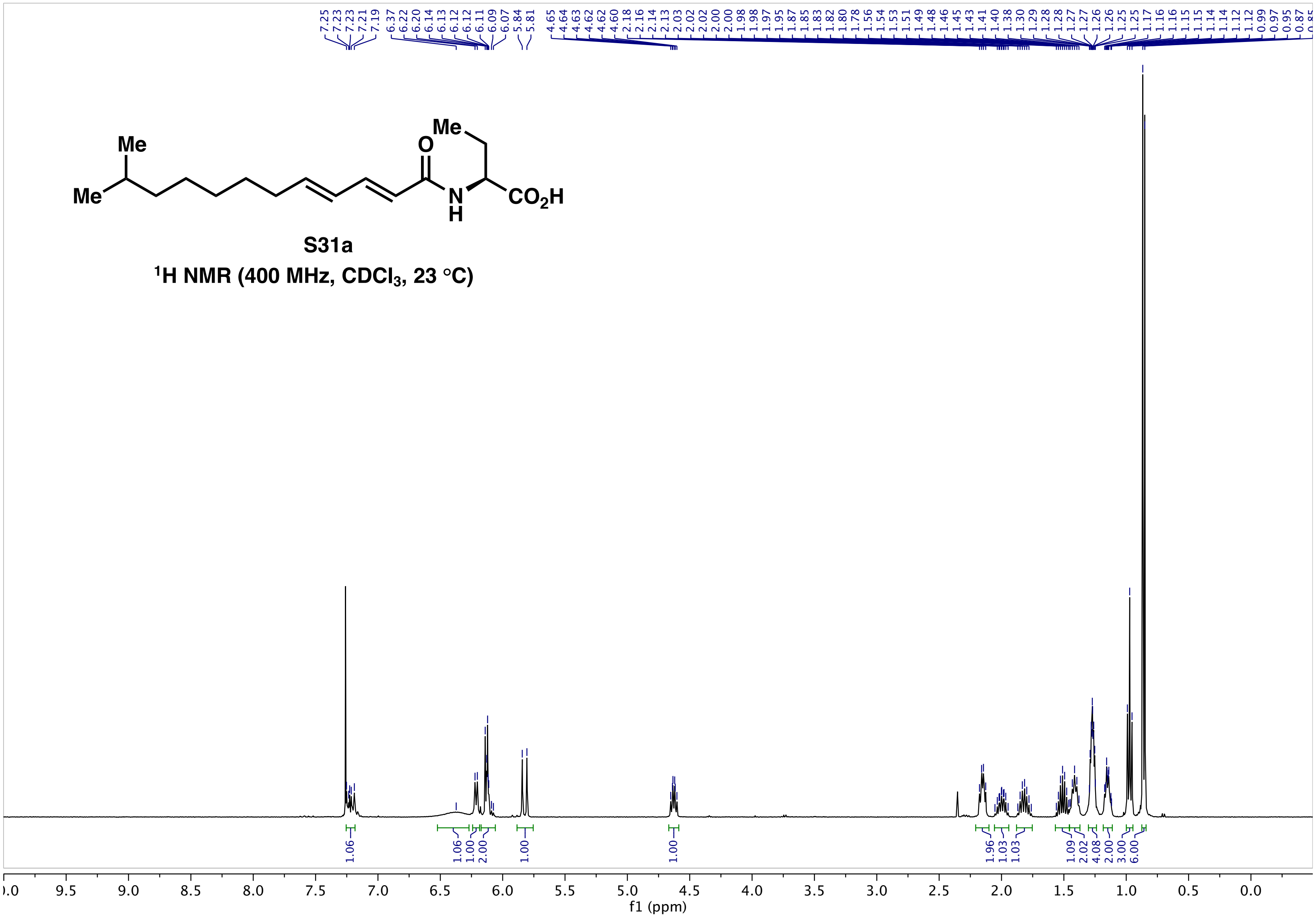

Me
Me
O
Me
N
H
CO2H
S31a
1H NMR (400 MHz, CDCl3, 23 °C)
f1 (ppm)

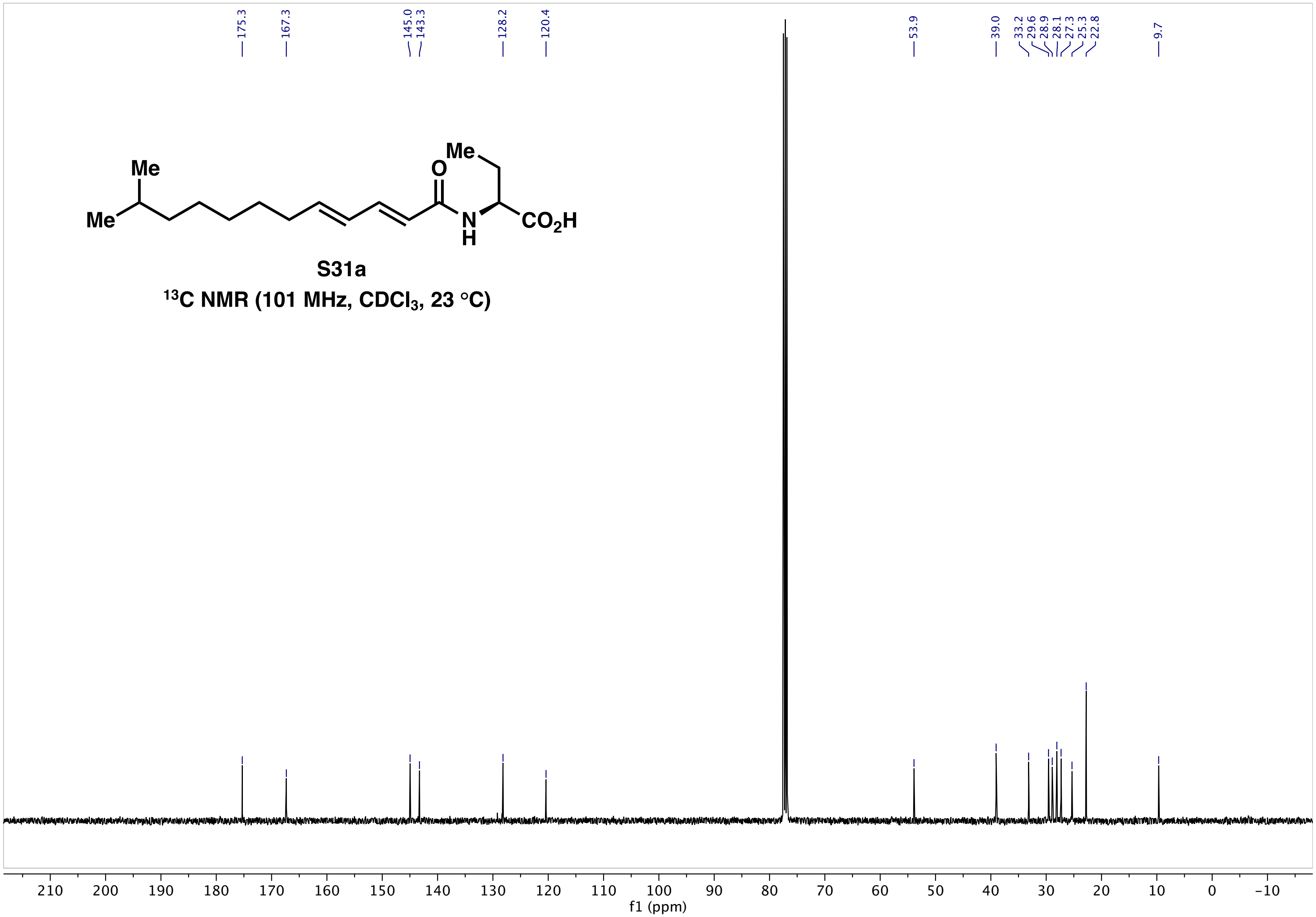
175.3
167.3
145.0
143.3
128.2
120.4
53.9
39.0
33.2
29.6
28.9
28.1
27.3
25.3
22.8
9.7
Me
Me
O
Me
N
H
CO_2H
S31a
^{13}C NMR (101 MHz, $CDCl_3$, 23 °C)
210
200
190
180
170
160
150
140
130
120
110
100
90
80
70
60
50
40
30
20
10
0
–10
f1 (ppm)

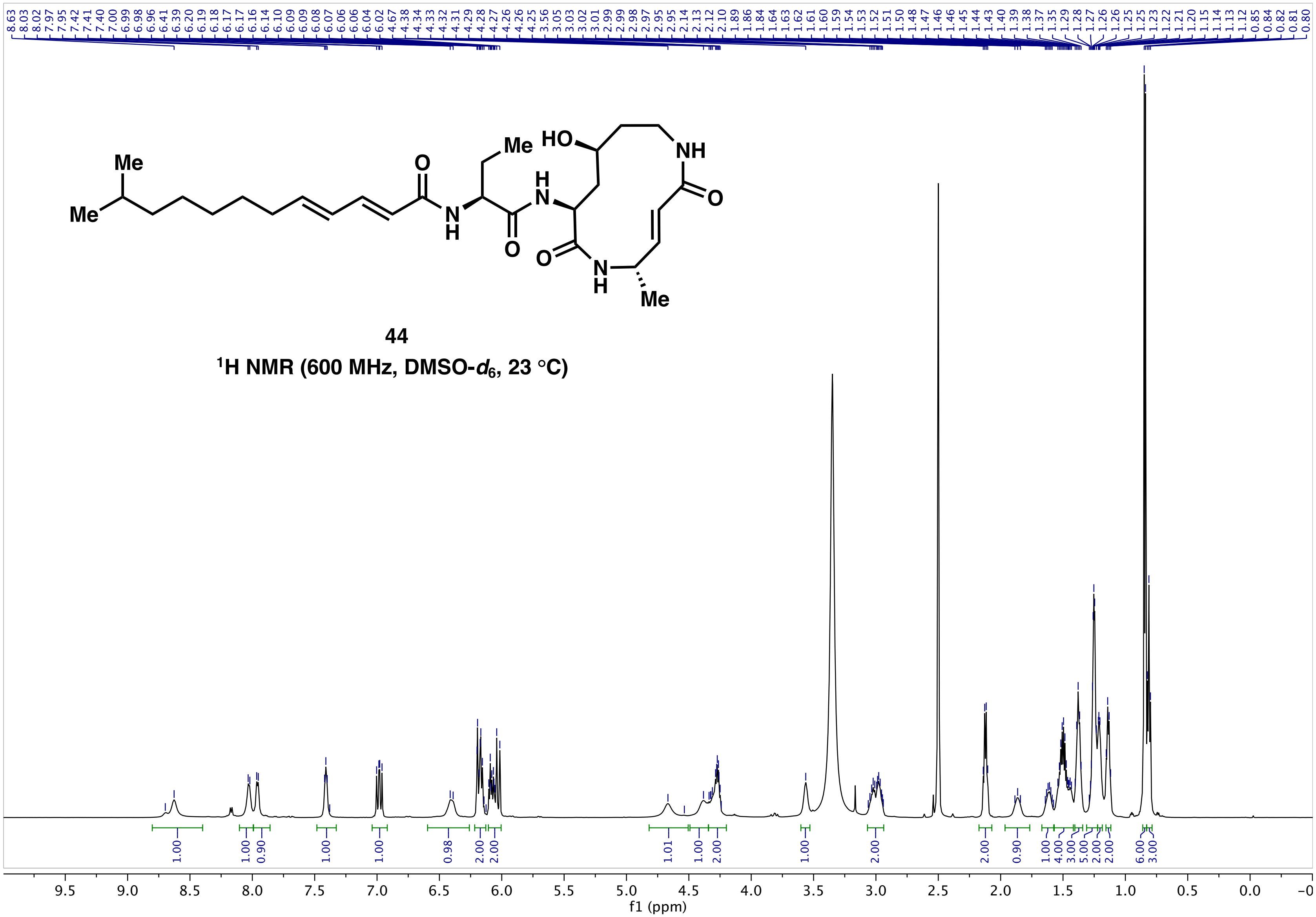

44

^1H NMR (600 MHz, DMSO-d_6, 23 °C)

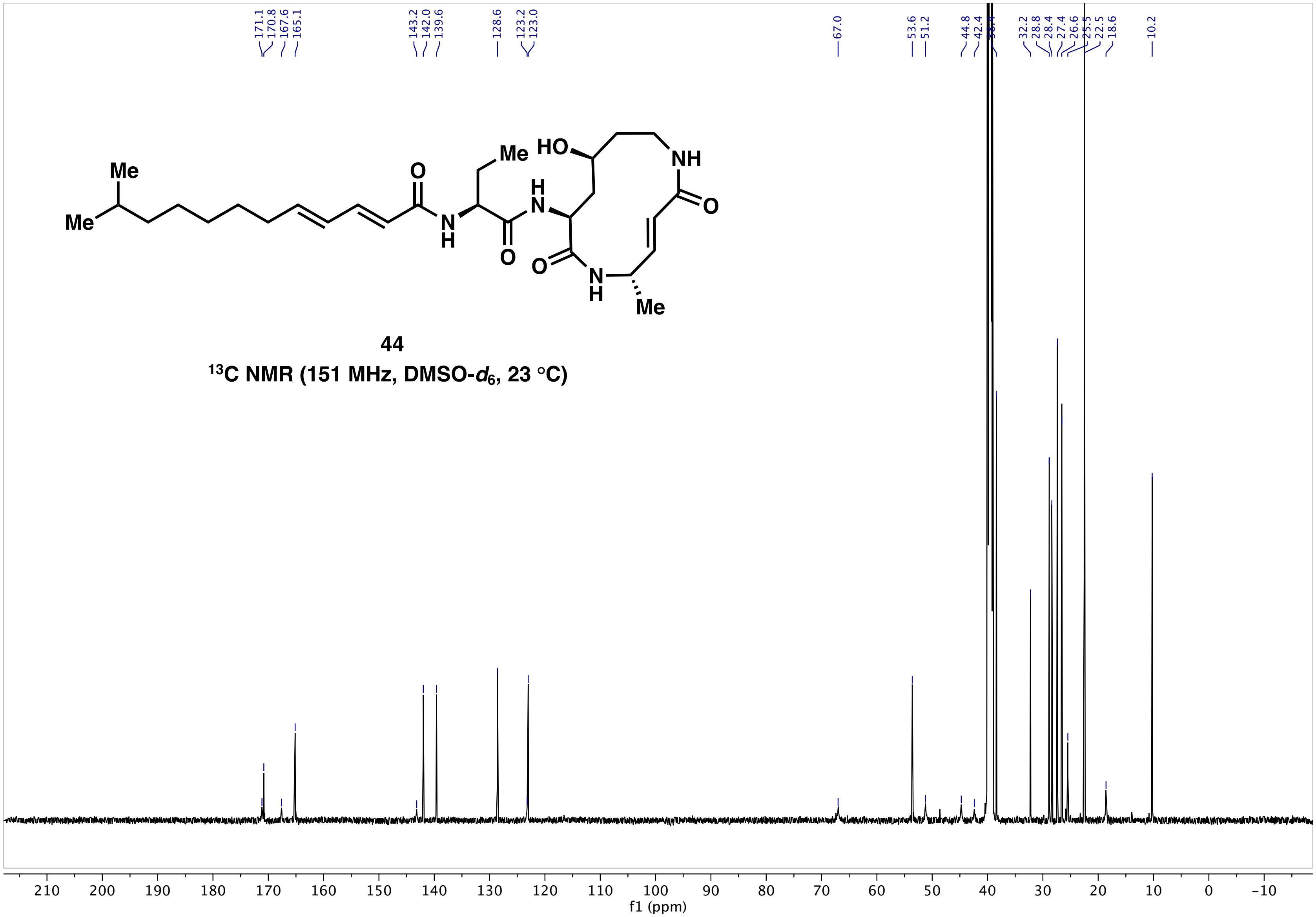

44

^{13}C NMR (151 MHz, DMSO-d_6, 23 °C)

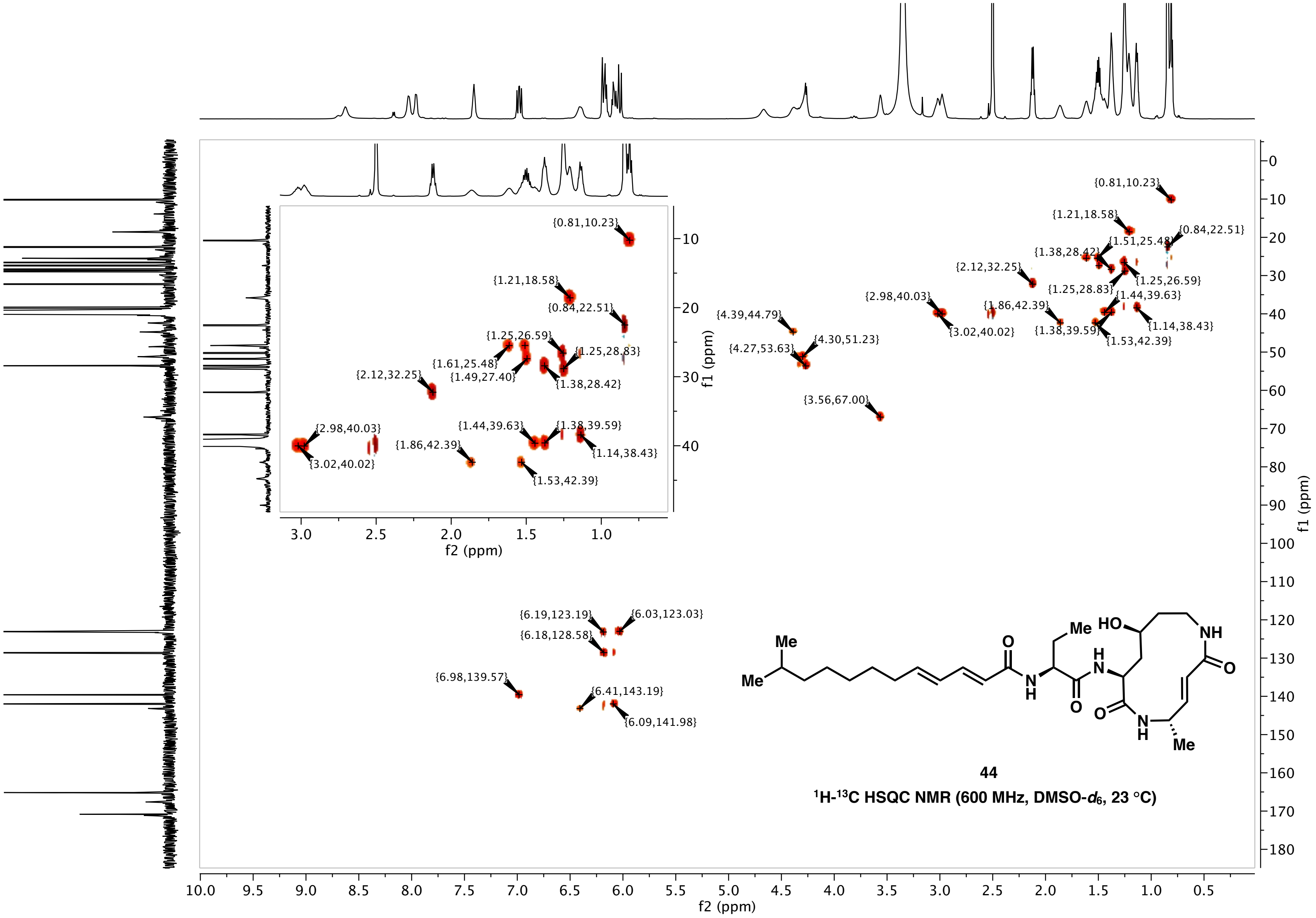

44

^{1}H-^{13}C HSQC NMR (600 MHz, DMSO-d_6, 23 °C)

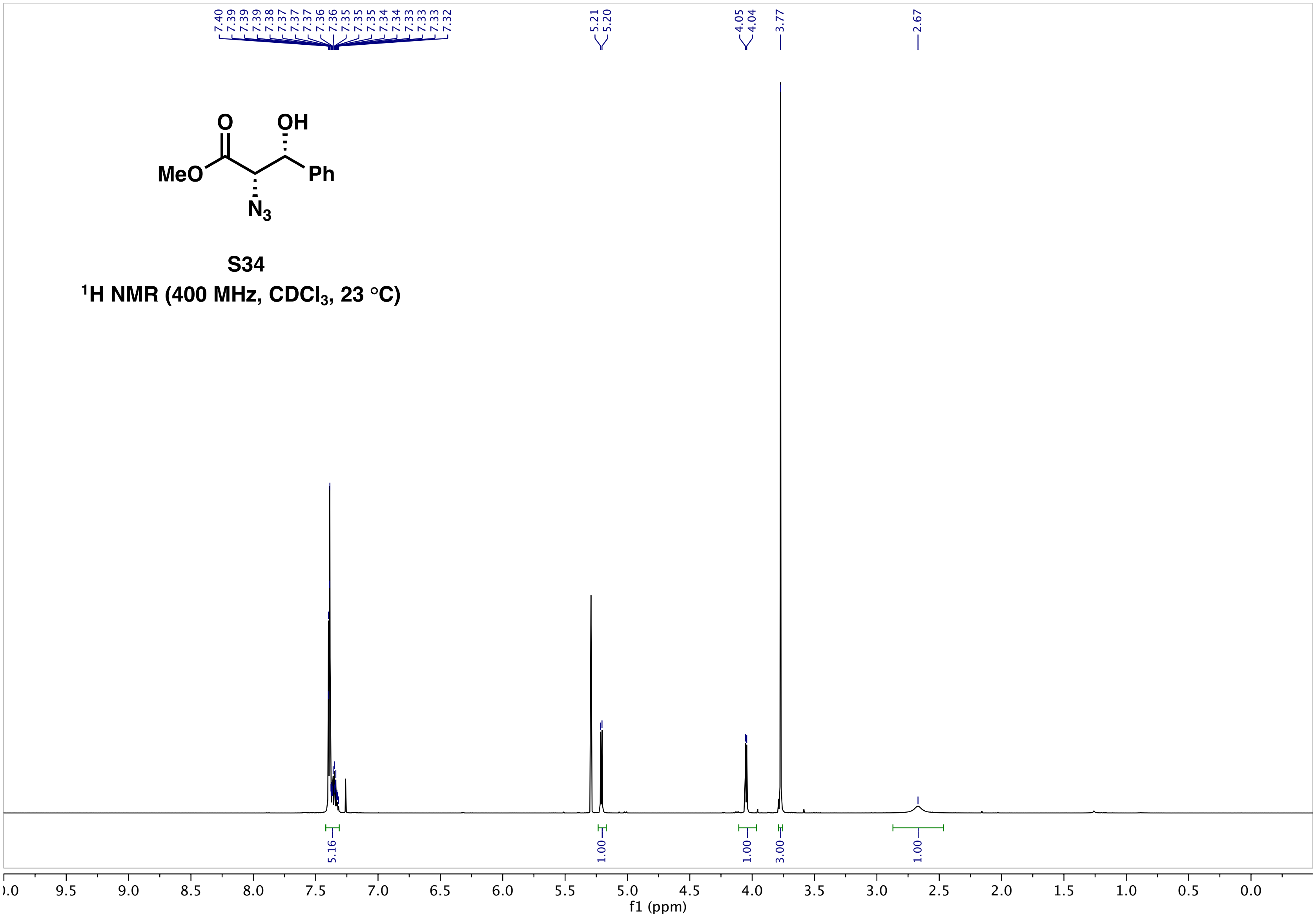

7.40 7.39 7.39 7.39 7.38 7.37 7.37 7.37 7.36 7.36 7.35 7.35 7.35 7.34 7.33 7.33 7.33 7.32
5.21 5.20
4.05 4.04
3.77
2.67
O
OH
MeO
Ph
N3
S34
1H NMR (400 MHz, CDCl3, 23 °C)
5.16
1.00
1.00
3.00
1.00
9.5 9.0 8.5 8.0 7.5 7.0 6.5 6.0 5.5 5.0 4.5 4.0 3.5 3.0 2.5 2.0 1.5 1.0 0.5 0.0
f1 (ppm)

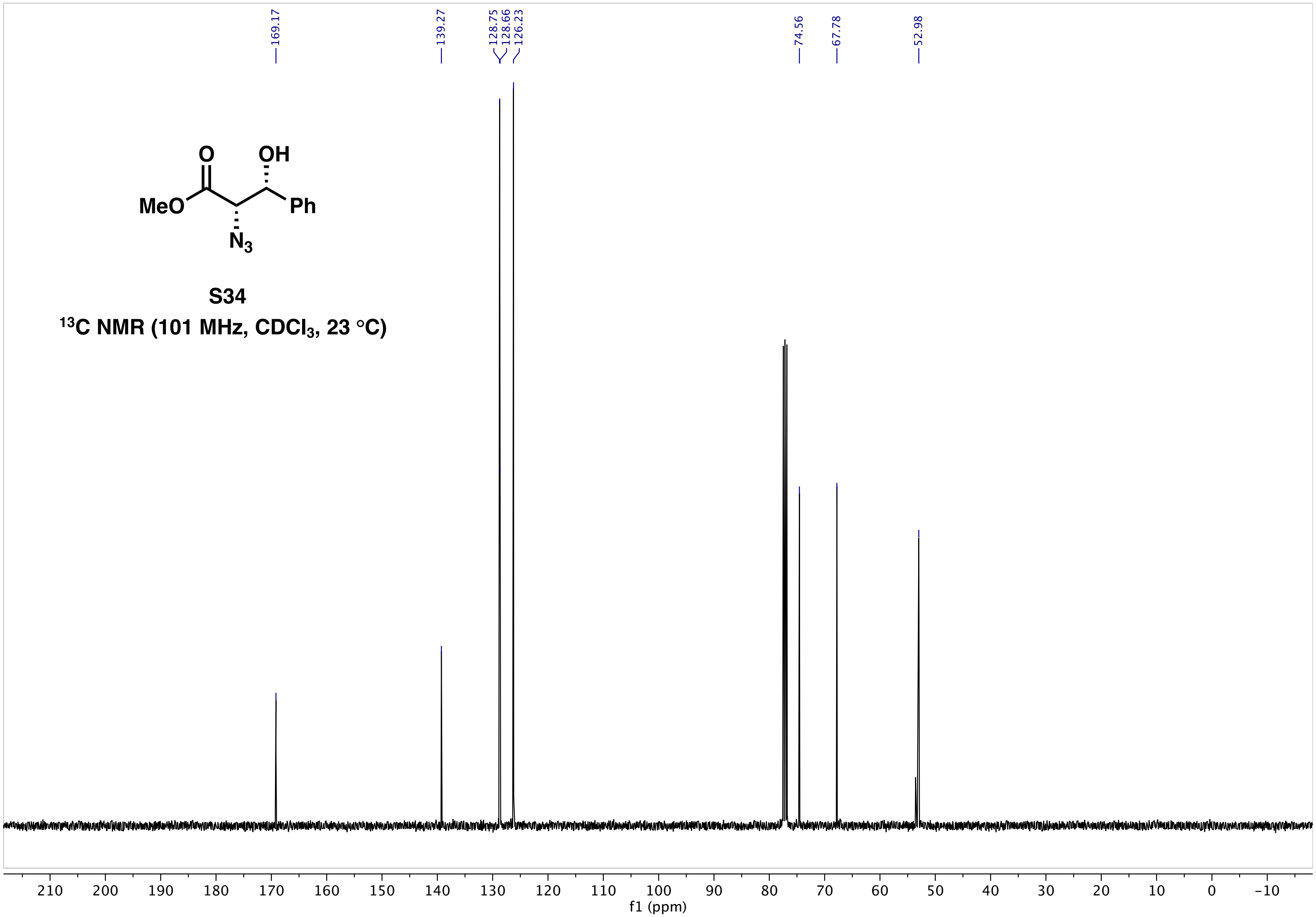

MeO
O
OH
Ph
N_3
S34
^{13}C NMR (101 MHz, $CDCl_3$, 23 °C)
169.17
139.27
128.75
128.66
126.23
74.56
67.78
52.98
210 200 190 180 170 160 150 140 130 120 110 100 90 80 70 60 50 40 30 20 10 0 -10
f1 (ppm)

S29b

^{1}H NMR (400 MHz, $CDCl_3$, 23 °C)

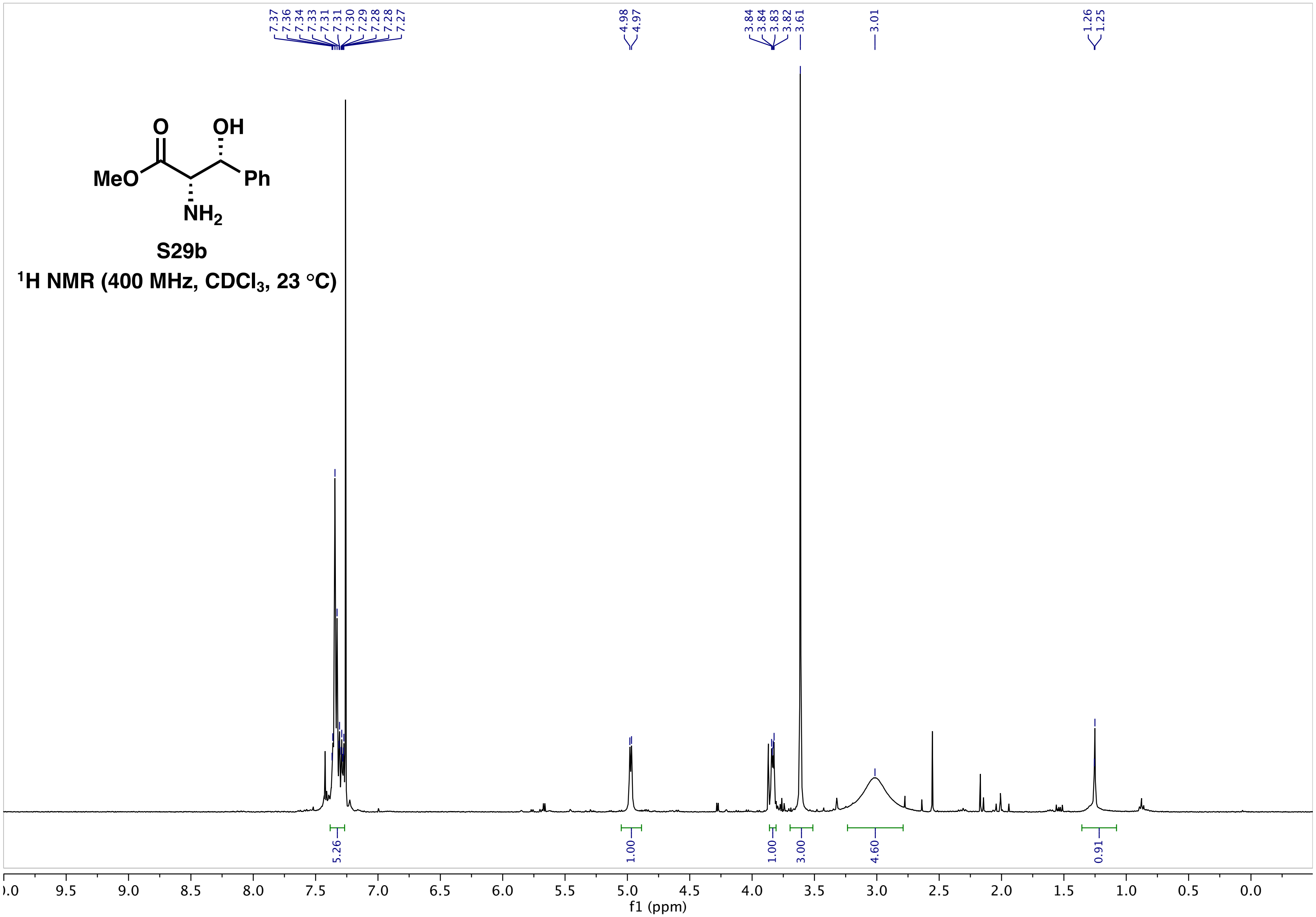

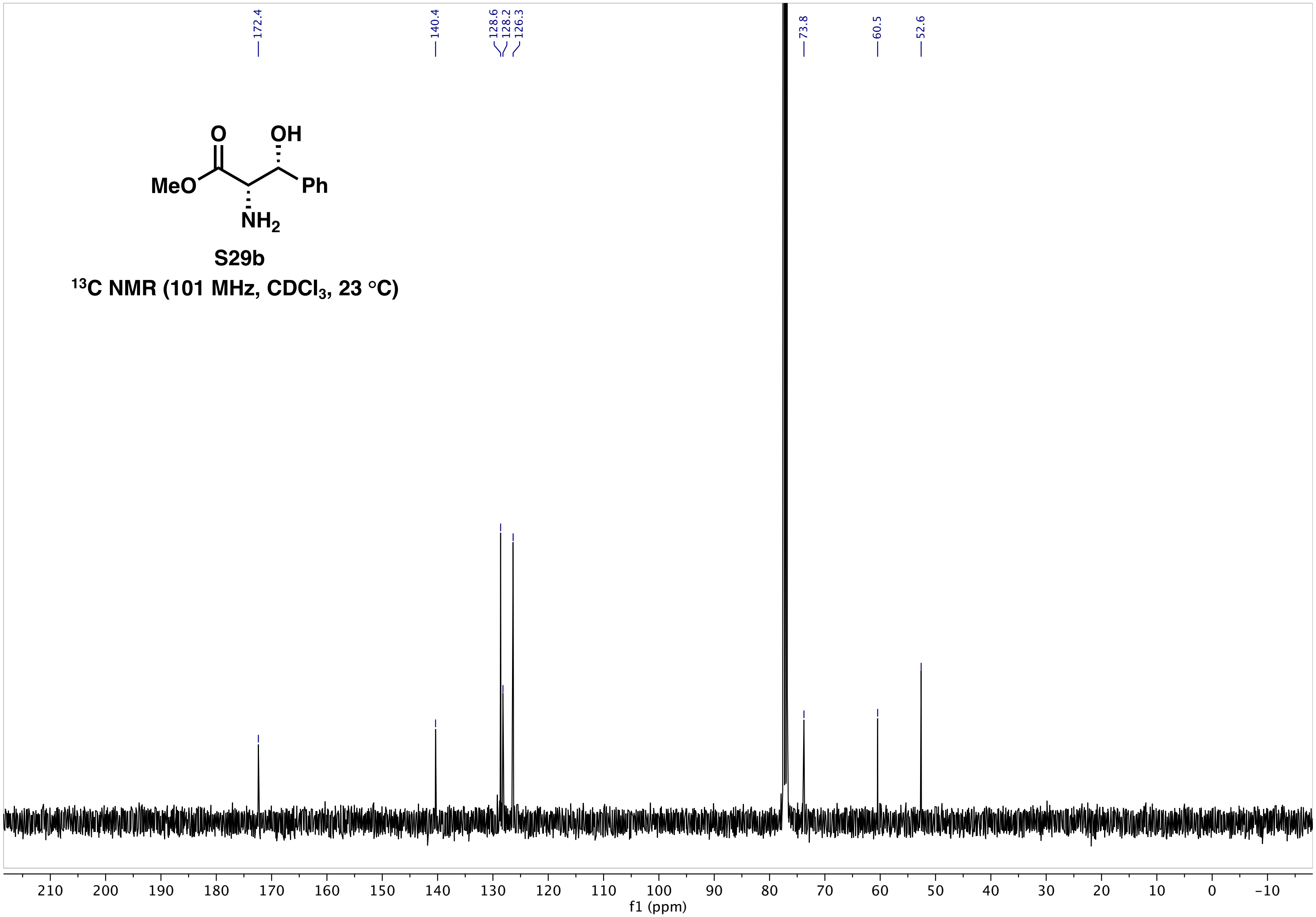
172.4
140.4
128.6
128.2
126.3
73.8
60.5
52.6
O
OH
MeO
Ph
NH2
S29b
13C NMR (101 MHz, CDCl3, 23 °C)
210 200 190 180 170 160 150 140 130 120 110 100 90 80 70 60 50 40 30 20 10 0 −10
f1 (ppm)

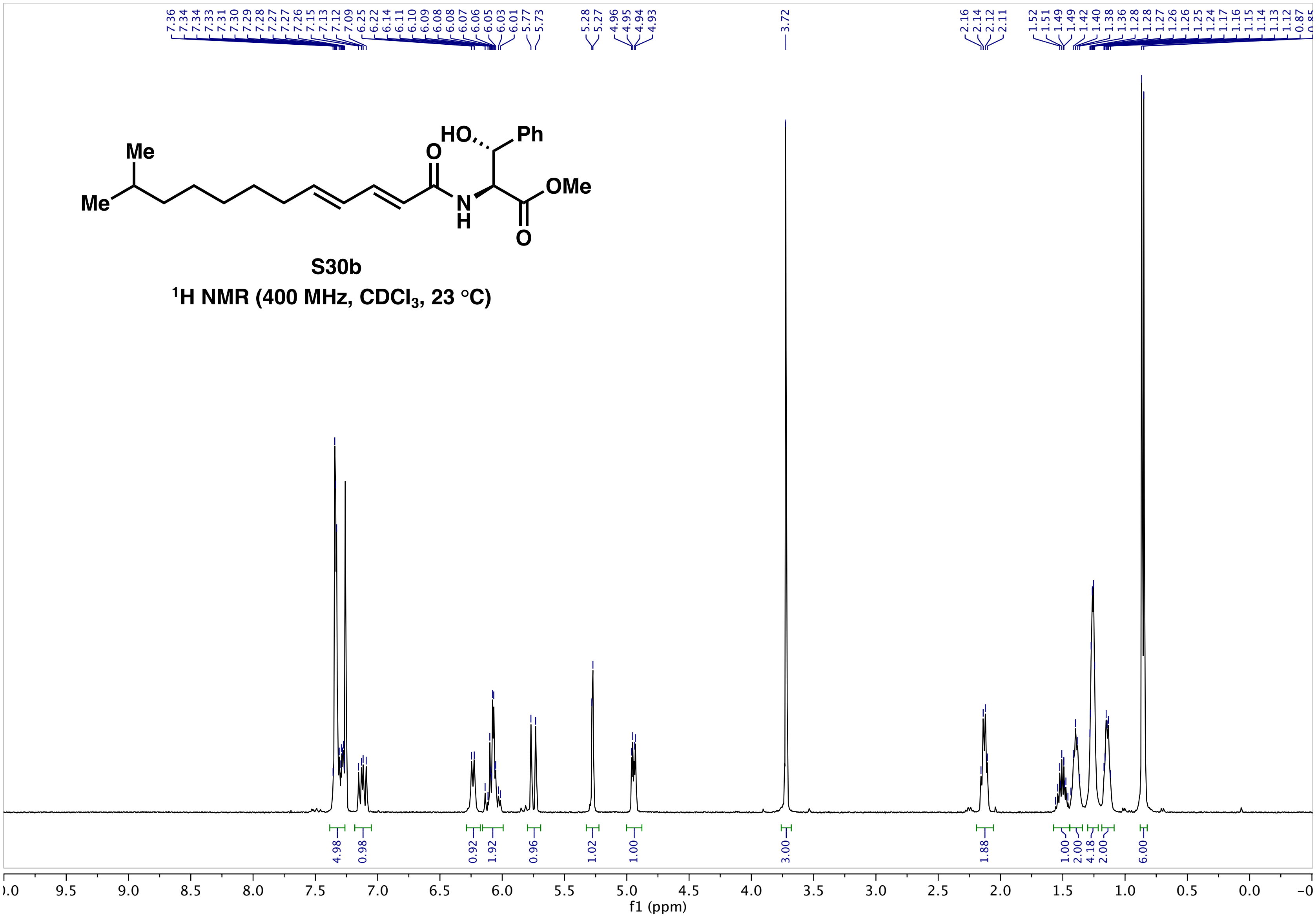
Me
Me
O
HO
Ph
N
H
OMe
O
S30b
1H NMR (400 MHz, CDCl3, 23 °C)
f1 (ppm)

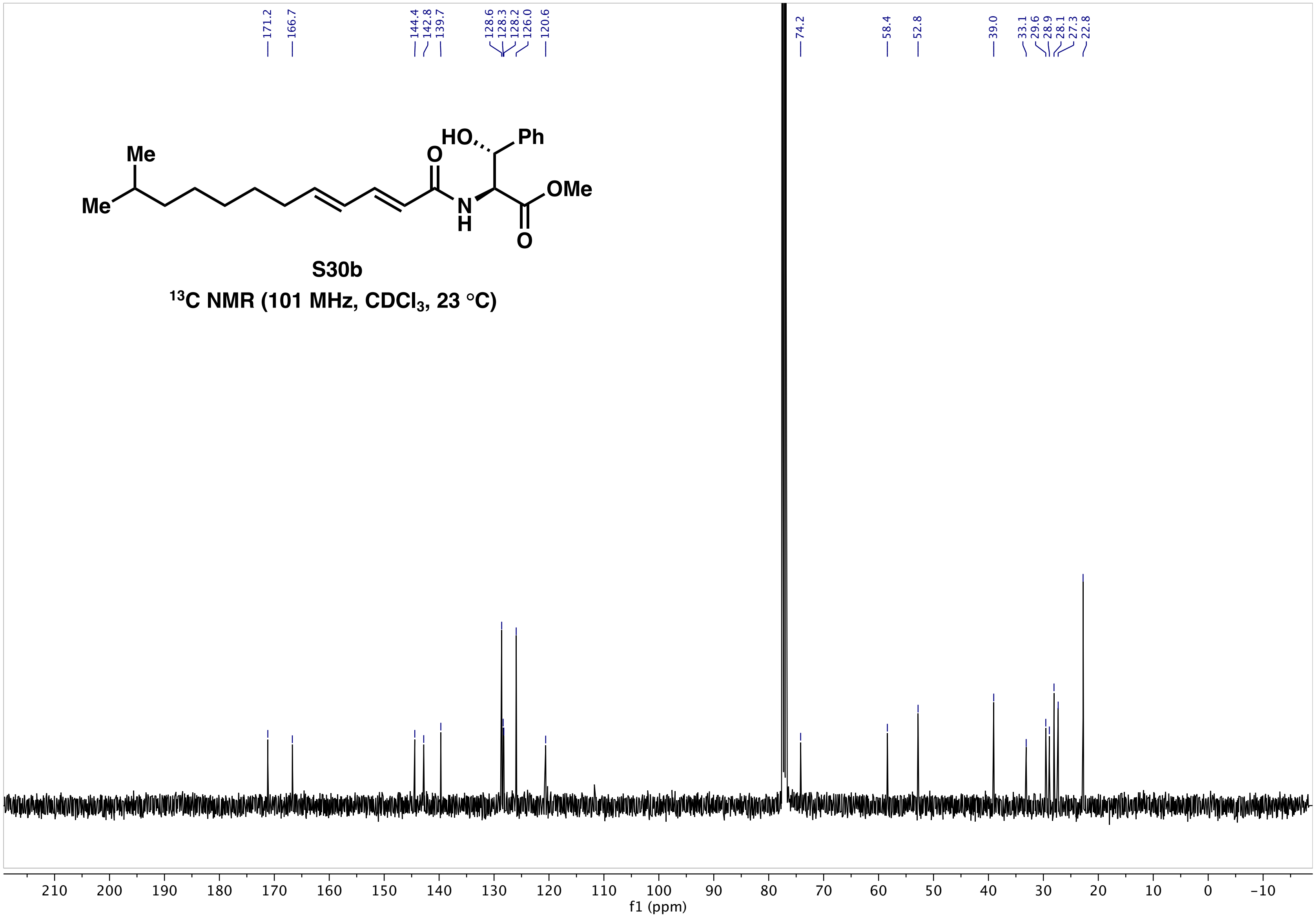
171.2
166.7
144.4
142.8
139.7
128.6
128.3
128.2
126.0
120.6
74.2
58.4
52.8
39.0
33.1
29.6
28.9
28.1
27.3
22.8
Me
Me
HO
Ph
O
OMe
N
H
O
S30b
^{13}C NMR (101 MHz, CDCl$_3$, 23 °C)
210
200
190
180
170
160
150
140
130
120
110
100
90
80
70
60
50
40
30
20
10
0
–10
f1 (ppm)

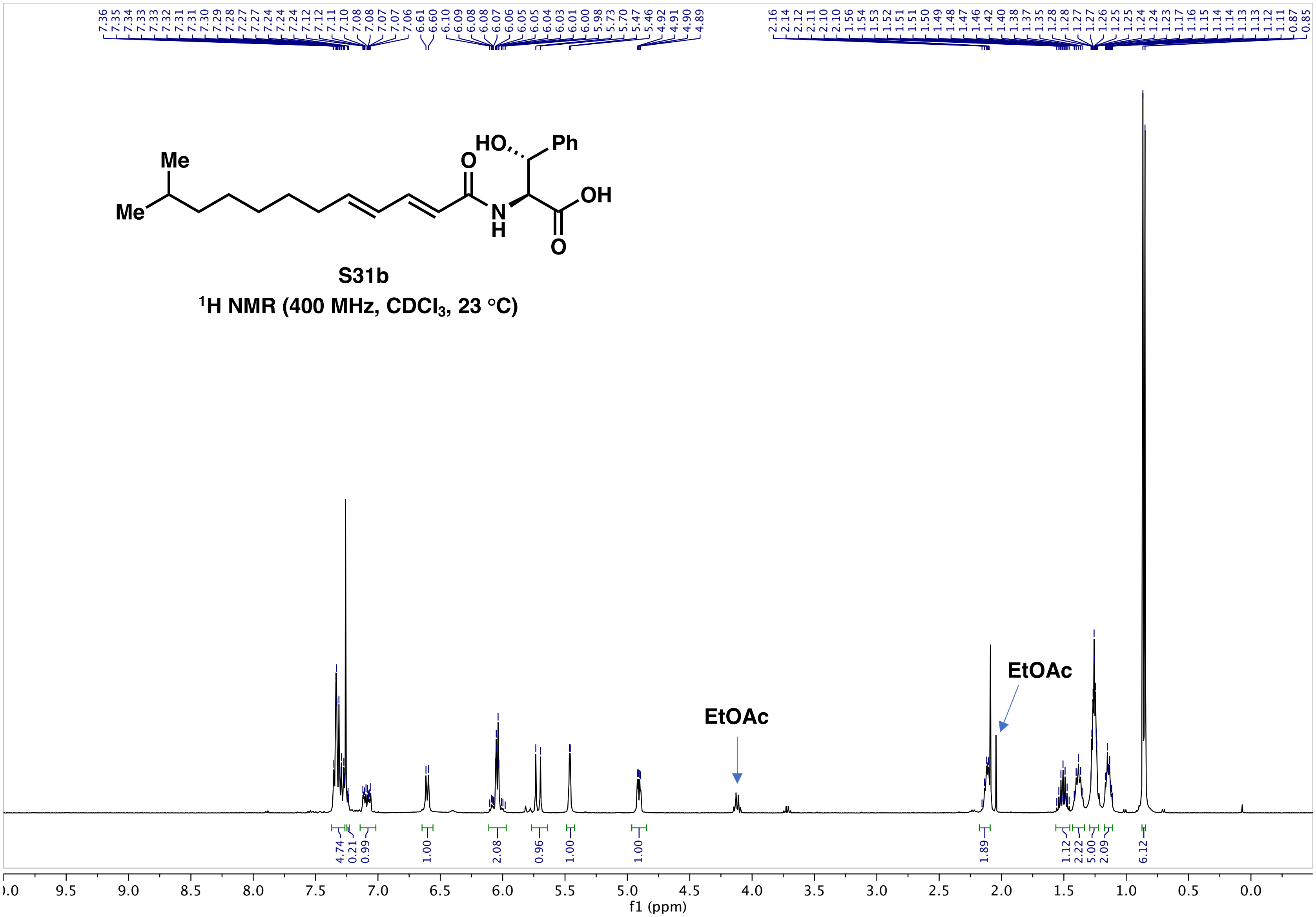
HO
Ph
Me
Me
O
N
H
OH
O
S31b
1H NMR (400 MHz, CDCl3, 23 °C)
EtOAc
EtOAc
f1 (ppm)

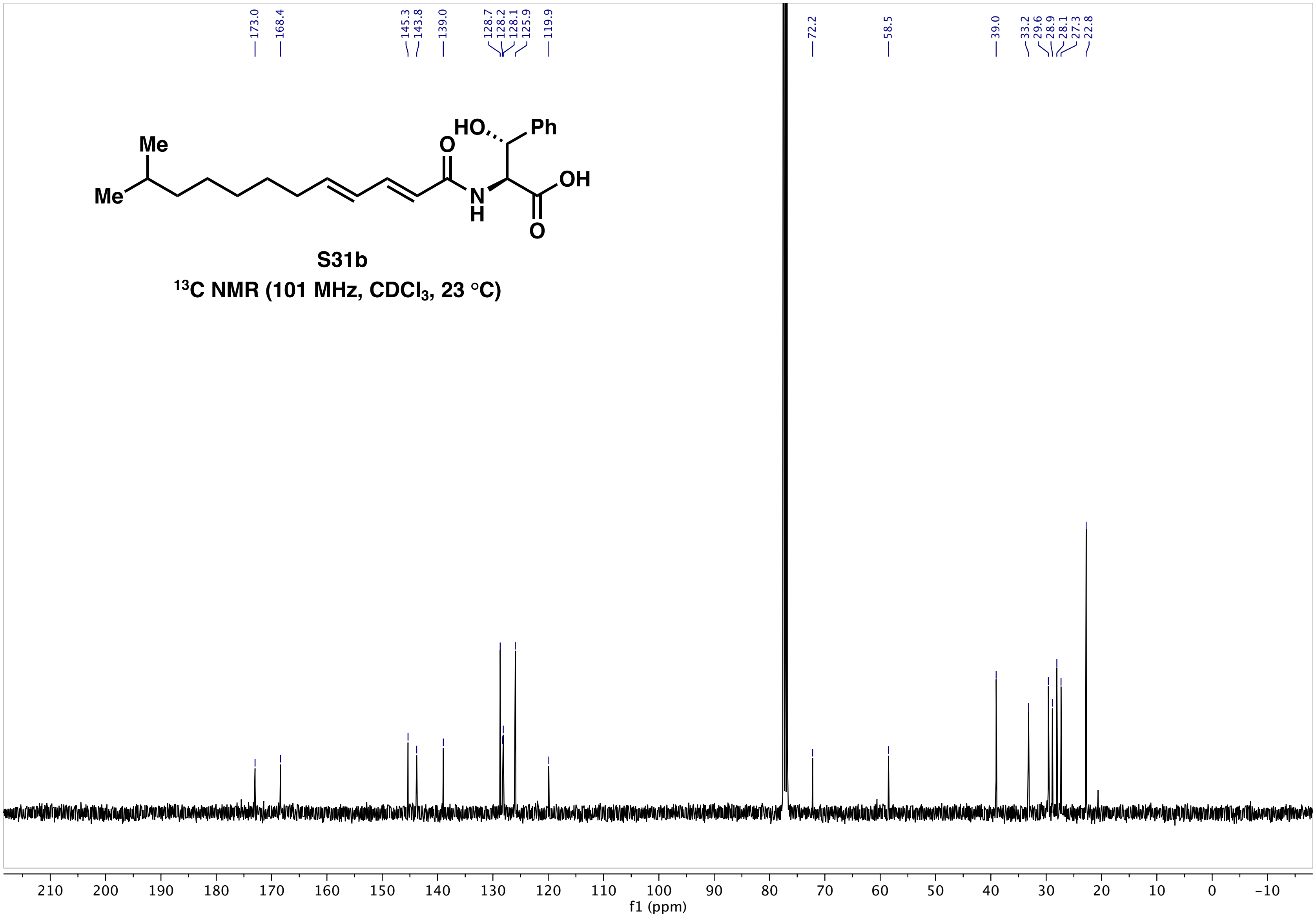

173.0
168.4
145.3
143.8
139.0
128.7
128.2
128.1
125.9
119.9
72.2
58.5
39.0
33.2
29.6
28.9
28.1
27.3
22.8
Me
Me
O
HO
Ph
N
H
OH
O
S31b
^{13}C NMR (101 MHz, $CDCl_3$, 23 °C)
210
200
190
180
170
160
150
140
130
120
110
100
90
80
70
60
50
40
30
20
10
0
–10
f1 (ppm)

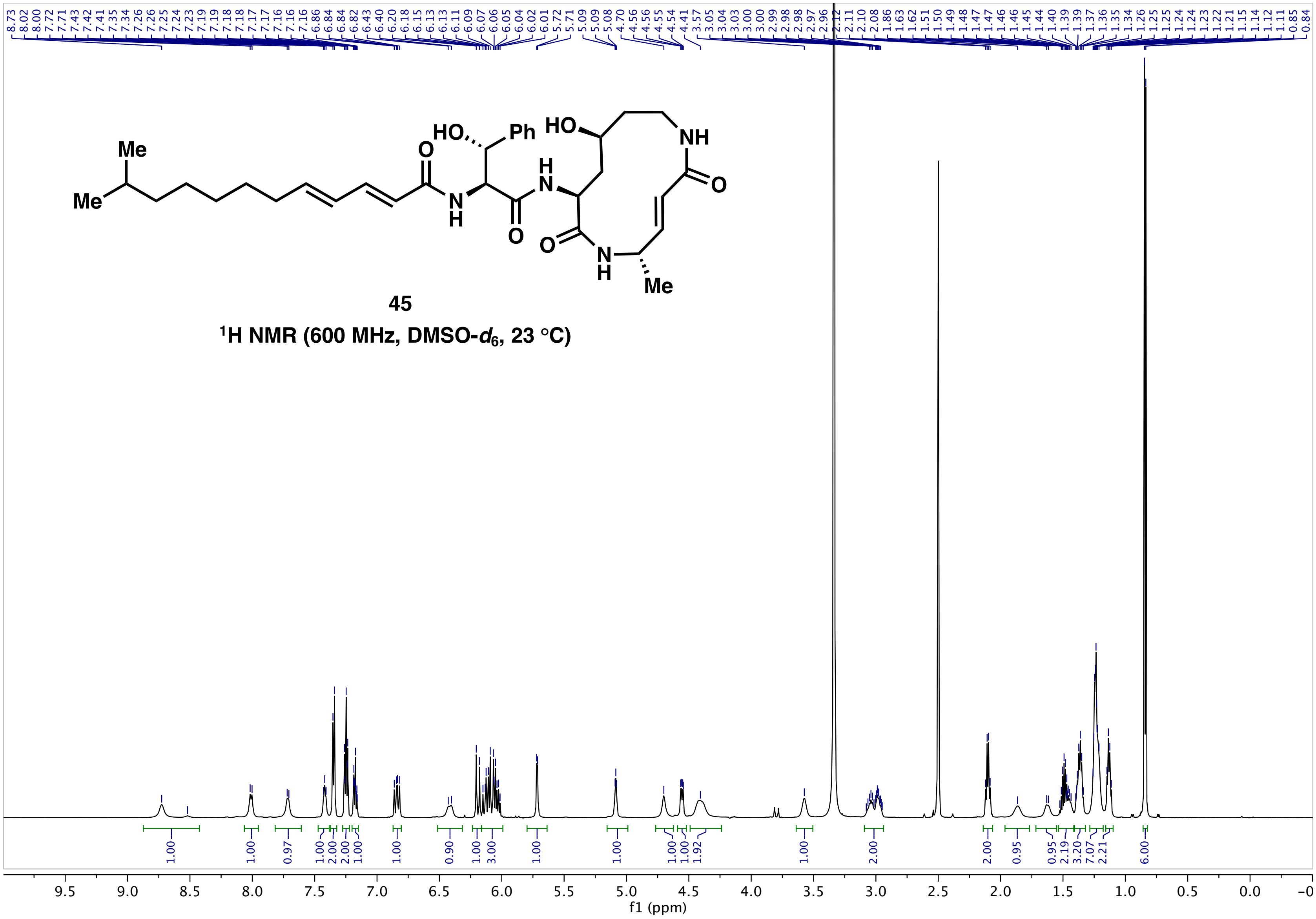

45

^{1}H NMR (600 MHz, DMSO-d_6, 23 °C)

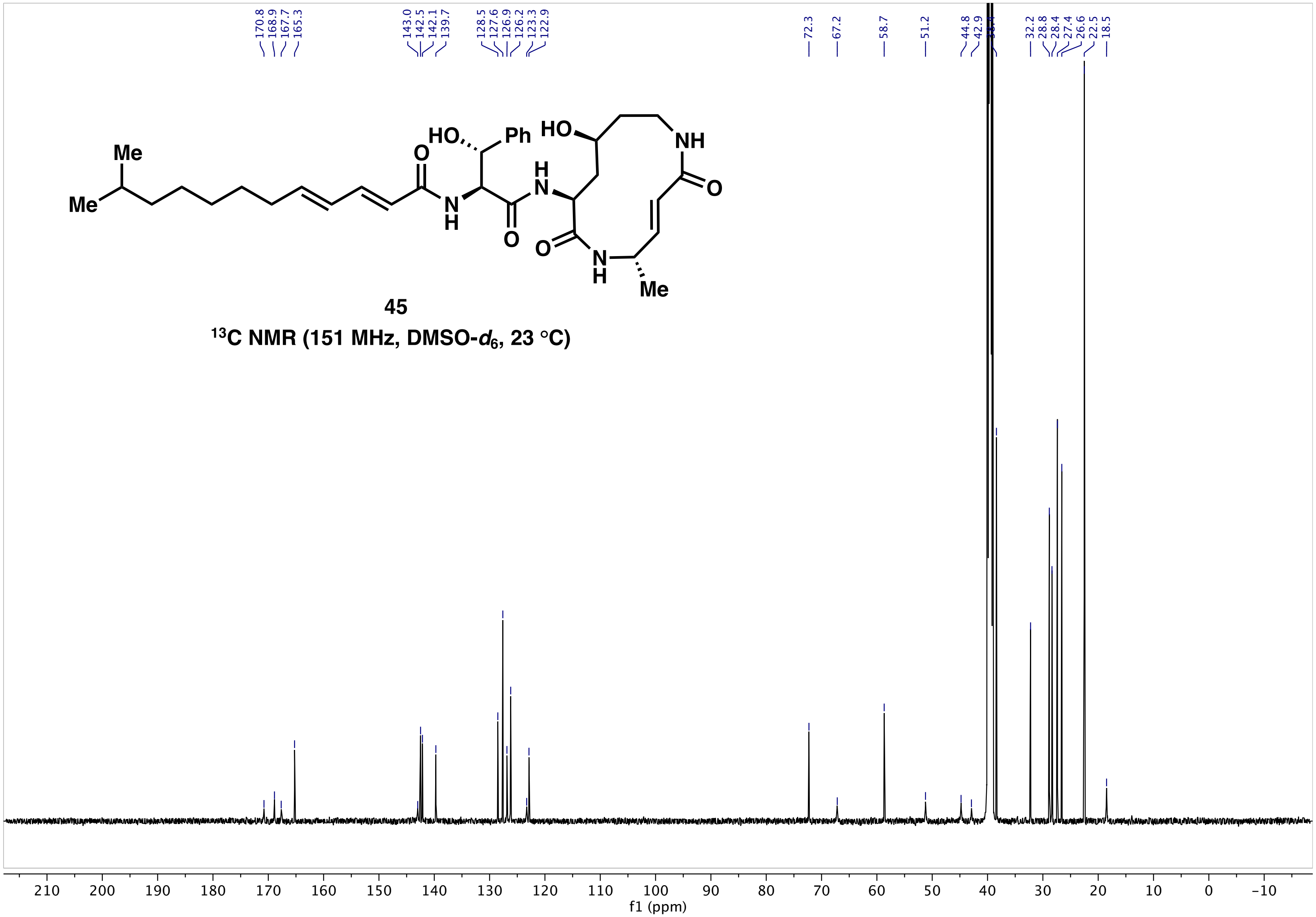

45

^{13}C NMR (151 MHz, DMSO-d_6, 23 °C)

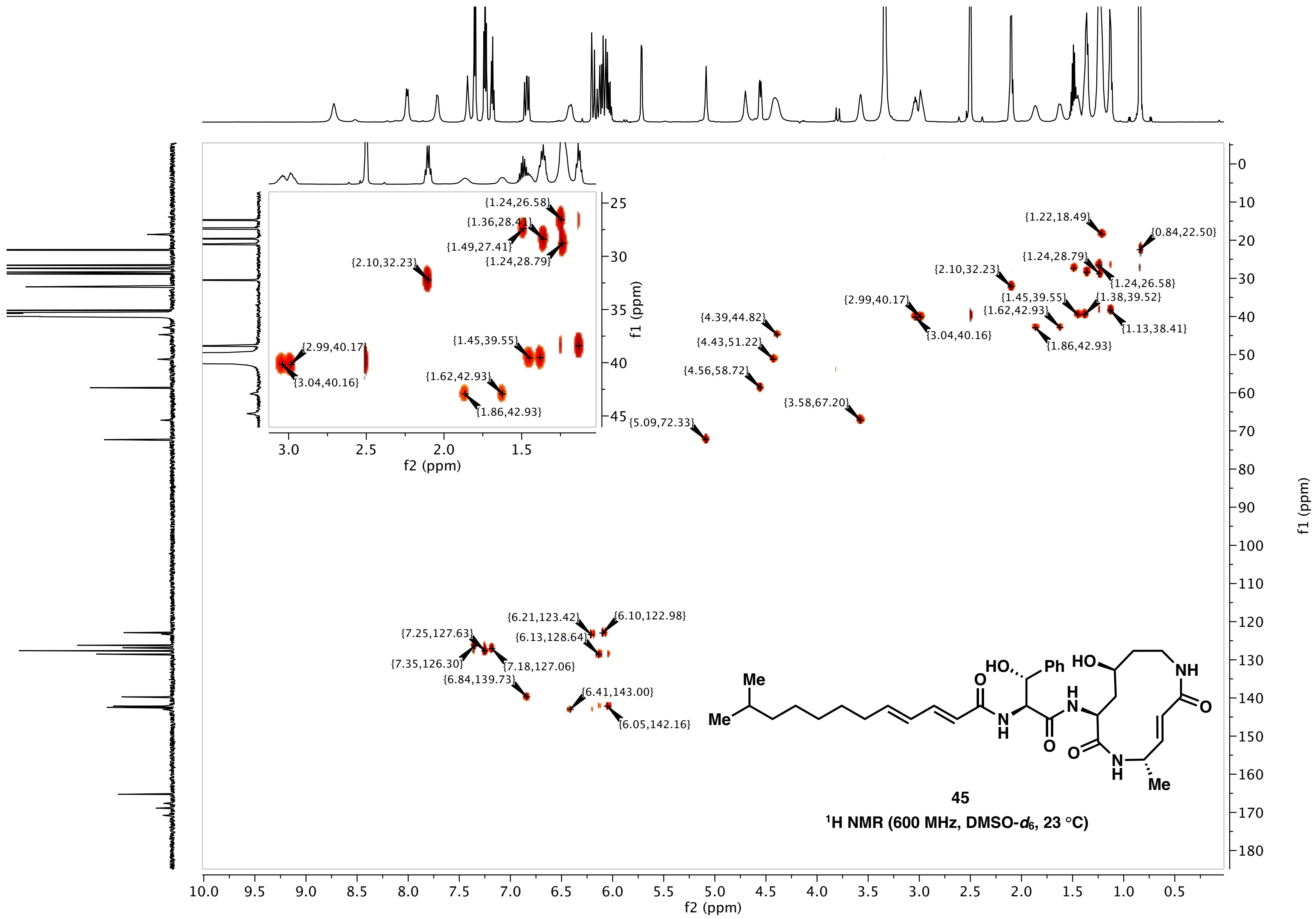

{1.22,18.49}
{0.84,22.50}
{1.24,28.79}
{2.10,32.23}
{1.24,26.58}
{2.99,40.17}
{1.45,39.55}
{1.38,39.52}
{1.62,42.93}
{4.39,44.82}
{3.04,40.16}
{1.13,38.41}
{4.43,51.22}
{1.86,42.93}
{4.56,58.72}
{3.58,67.20}
{5.09,72.33}
{1.24,26.58}
{1.36,28.41}
{1.49,27.41}
{1.24,28.79}
{2.10,32.23}
{2.99,40.17}
{1.45,39.55}
{3.04,40.16}
{1.62,42.93}
{1.86,42.93}
{6.21,123.42}
{6.10,122.98}
{7.25,127.63}
{6.13,128.64}
{7.35,126.30}
{7.18,127.06}
{6.84,139.73}
{6.41,143.00}
{6.05,142.16}
HO
Ph
HO
NH
Me
Me
O
N
H
H
N
O
O
O
N
H
Me
45
1H NMR (600 MHz, DMSO-d6, 23 °C)
f2 (ppm)
f1 (ppm)

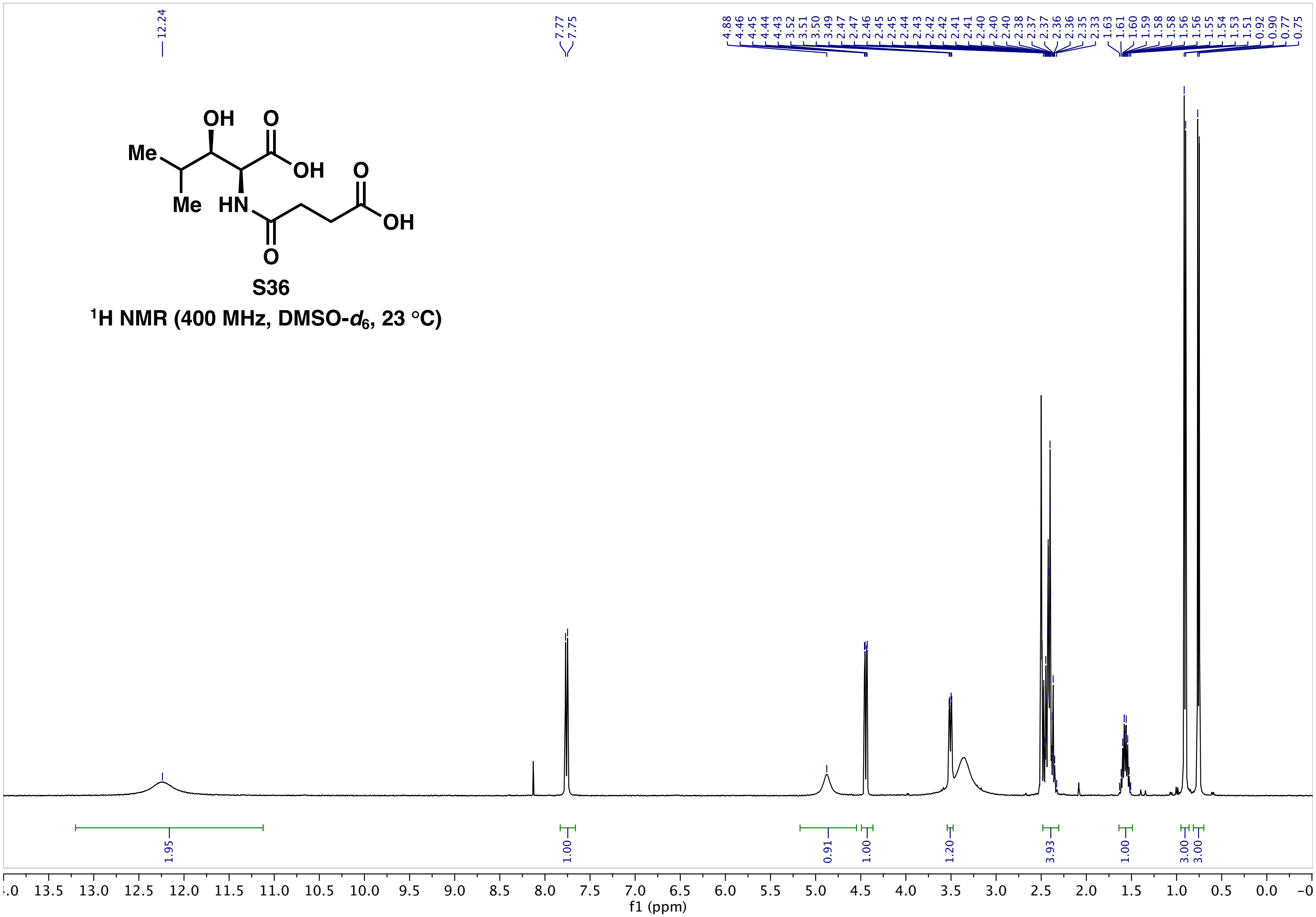
OH
O
Me
OH
O
Me
HN
OH
O
S36
1H NMR (400 MHz, DMSO-d6, 23 °C)

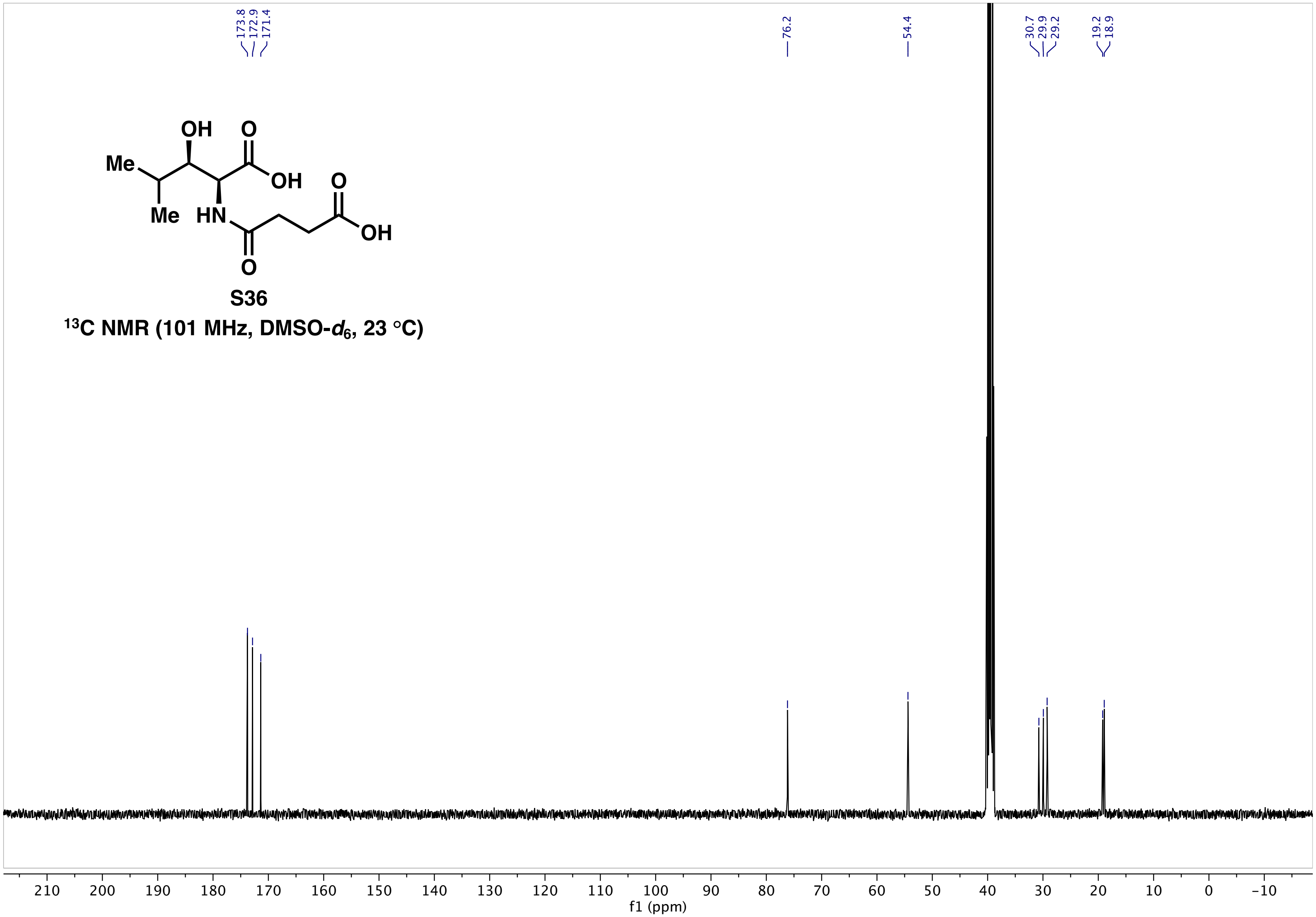
173.8
172.9
171.4
76.2
54.4
30.7
29.9
29.2
19.2
18.9
OH
O
Me
OH
O
Me
HN
OH
O
S36
^{13}C NMR (101 MHz, DMSO-d_6, 23 °C)
210
200
190
180
170
160
150
140
130
120
110
100
90
80
70
60
50
40
30
20
10
0
–10
f1 (ppm)

S37

^{1}H NMR (400 MHz, Methanol-d_4, 23 °C))

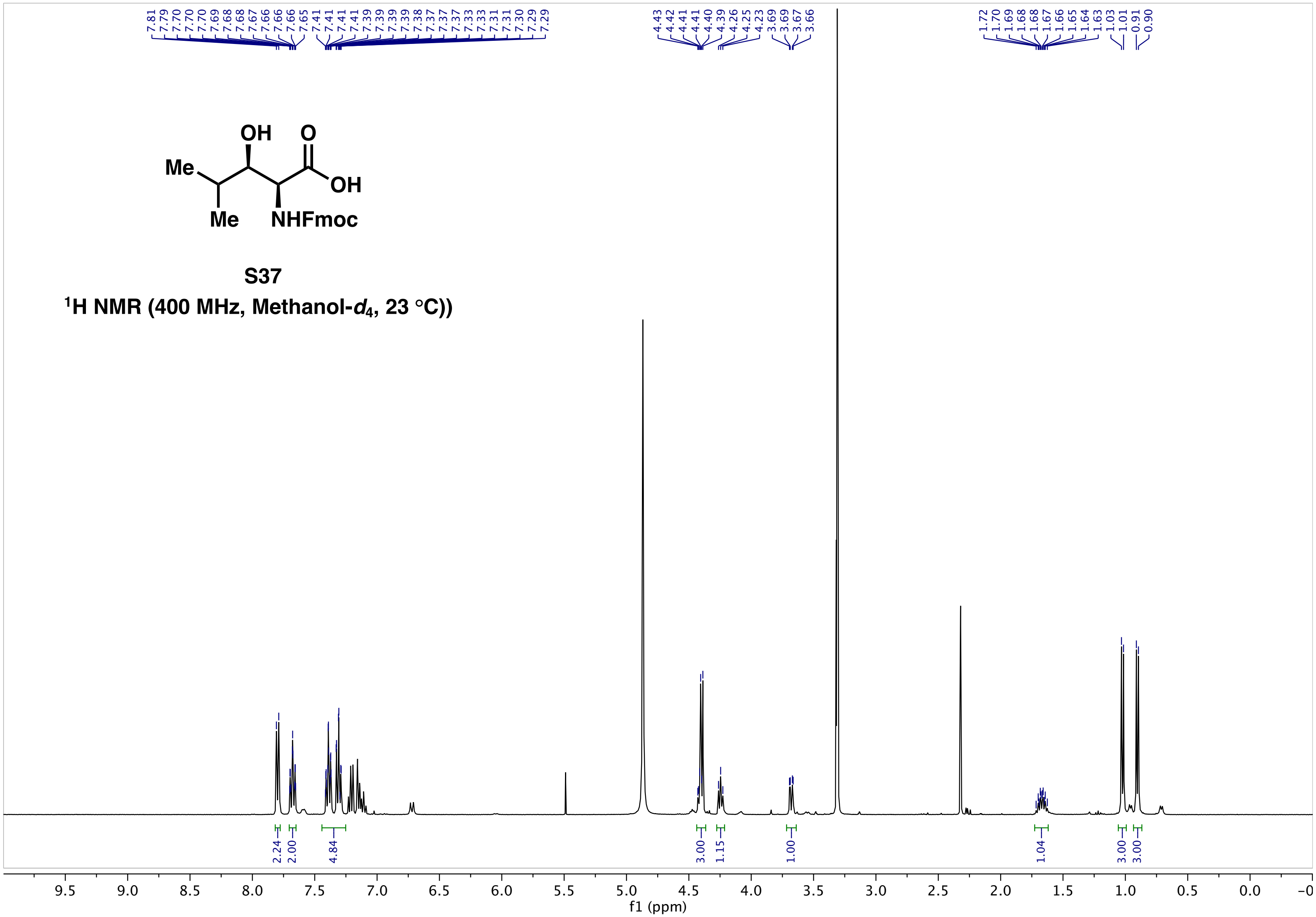

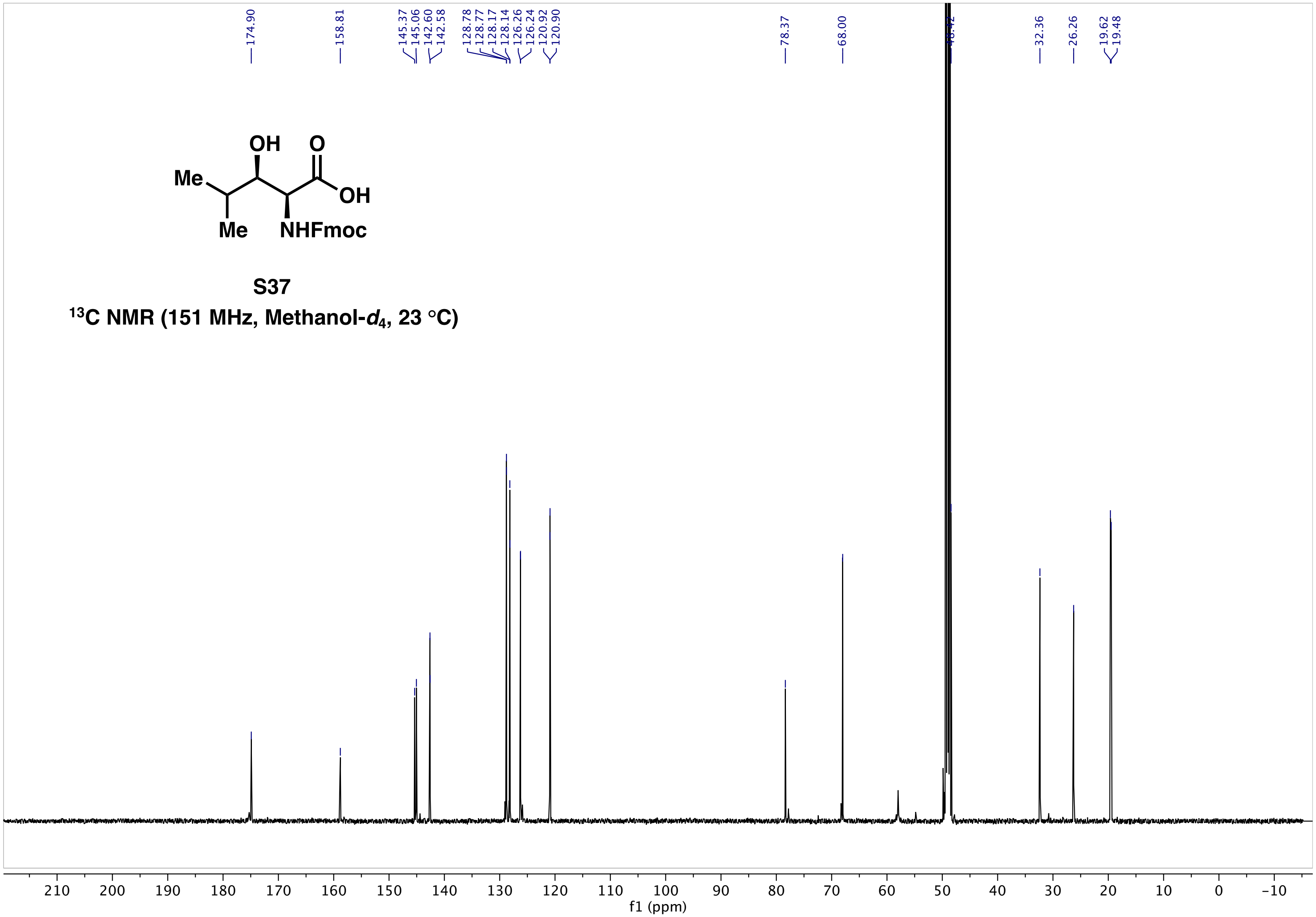
174.90
158.81
145.37
145.06
142.60
142.58
128.78
128.77
128.17
128.14
126.26
126.24
120.92
120.90
78.37
68.00
32.36
26.26
19.62
19.48
OH
O
Me
OH
Me
NHFmoc
S37
^{13}C NMR (151 MHz, Methanol-d_4, 23 °C)
210 200 190 180 170 160 150 140 130 120 110 100 90 80 70 60 50 40 30 20 10 0 −10
f1 (ppm)

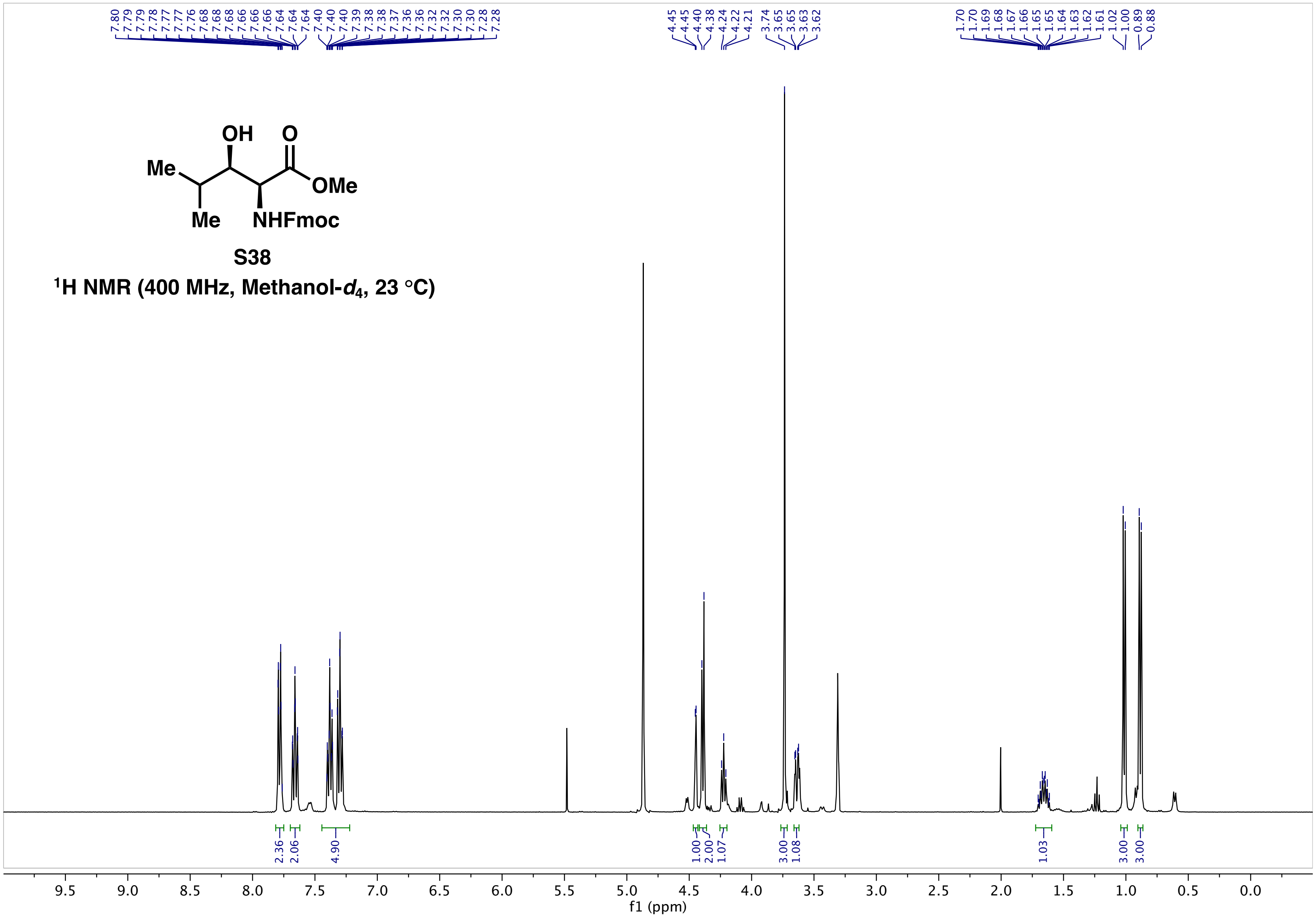
OH
O
Me
OMe
Me
NHFmoc
S38
1H NMR (400 MHz, Methanol-d4, 23 °C)
f1 (ppm)

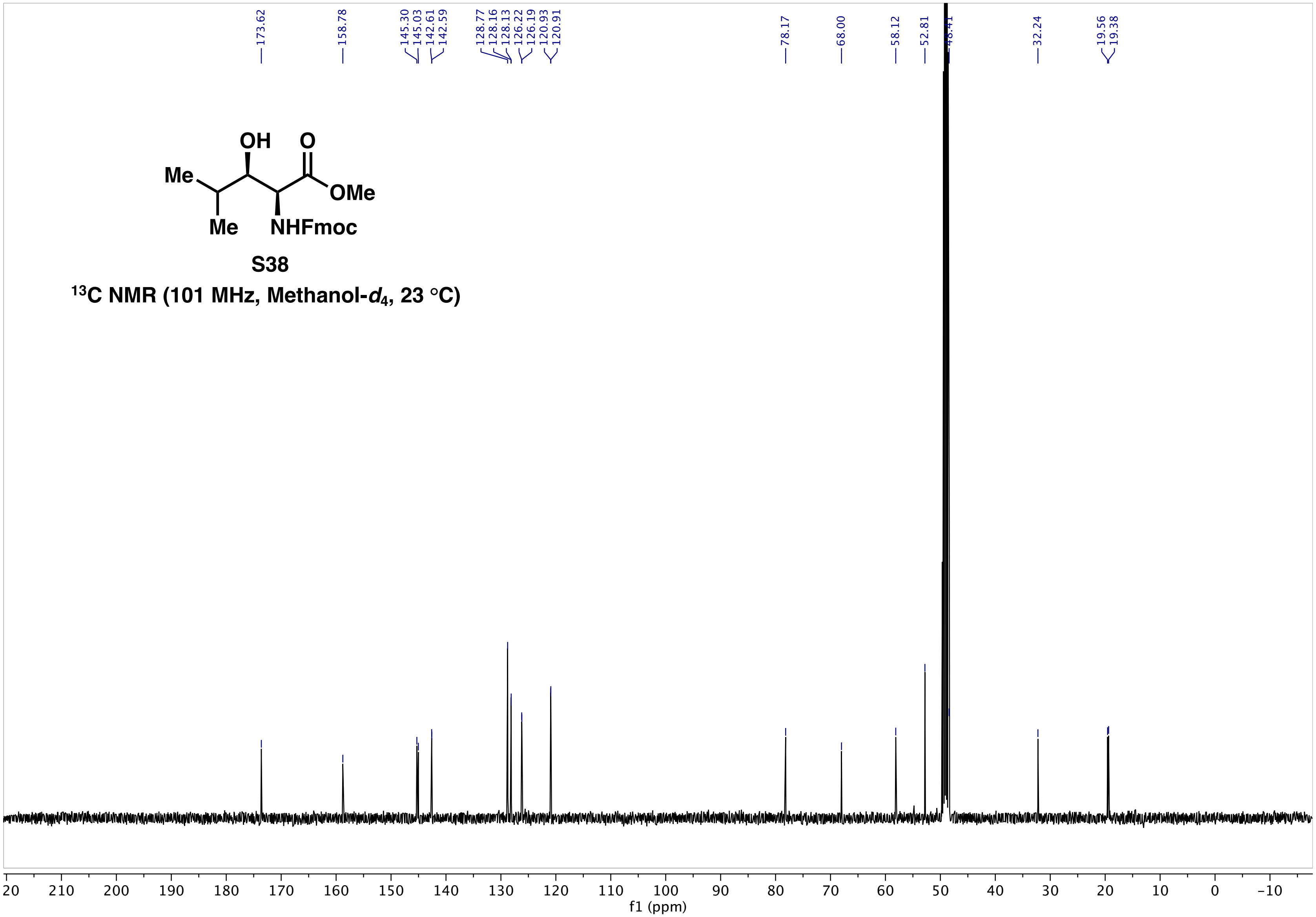
173.62
158.78
145.30
145.03
142.61
142.59
128.77
128.16
128.13
126.22
126.19
120.93
120.91
78.17
68.00
58.12
52.81
48.41
32.24
19.56
19.38
OH
O
Me
OMe
Me
NHFmoc
S38
13C NMR (101 MHz, Methanol-d4, 23 °C)
f1 (ppm)

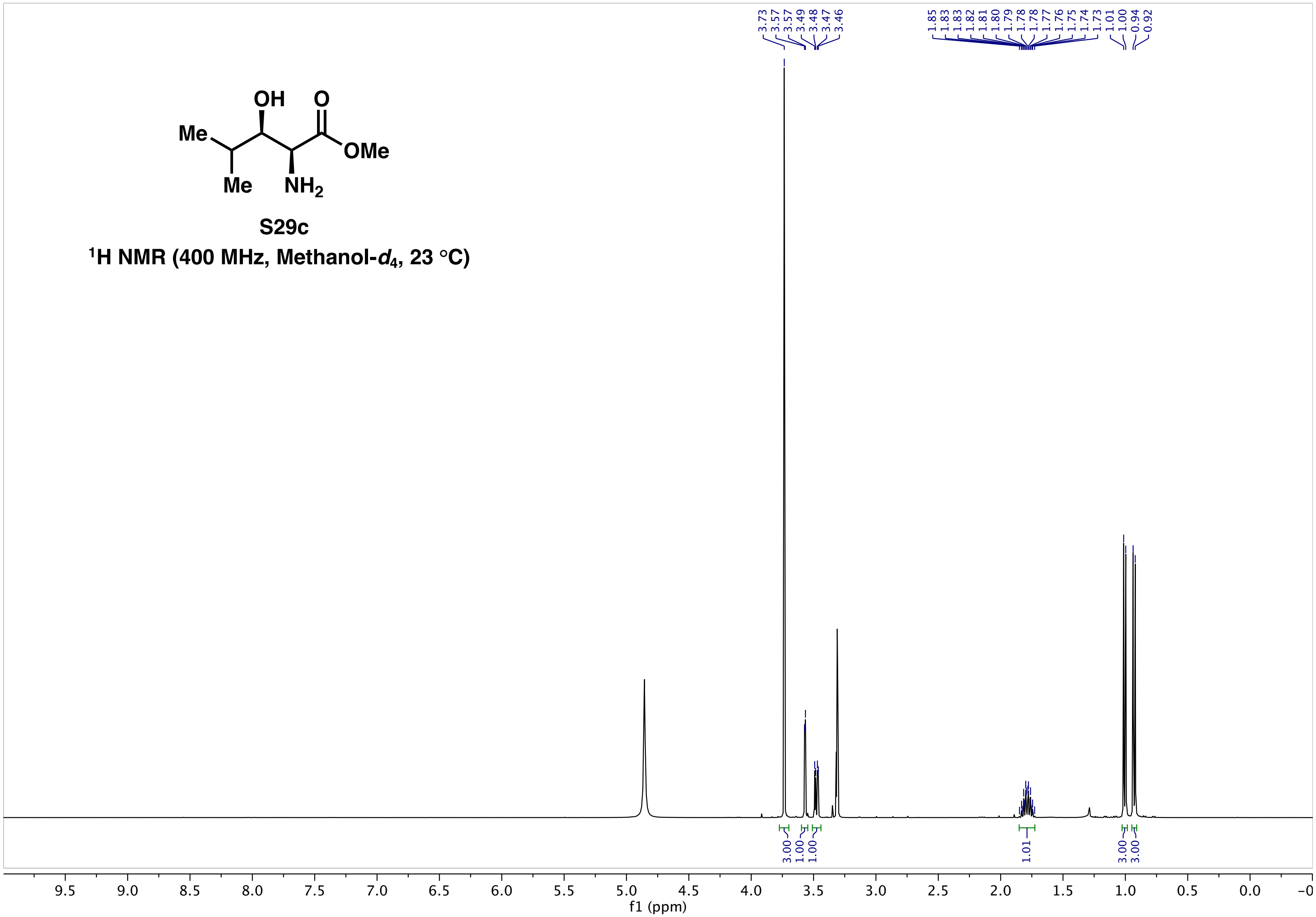
OH
O
Me
OMe
Me
NH2
S29c
1H NMR (400 MHz, Methanol-d4, 23 °C)
3.73 3.57 3.57 3.49 3.48 3.47 3.46
1.85 1.83 1.83 1.82 1.81 1.80 1.79 1.78 1.78 1.77 1.76 1.75 1.74 1.73 1.01 1.00 0.94 0.92
3.00 1.00 1.00
1.01
3.00 3.00
9.5 9.0 8.5 8.0 7.5 7.0 6.5 6.0 5.5 5.0 4.5 4.0 3.5 3.0 2.5 2.0 1.5 1.0 0.5 0.0
f1 (ppm)

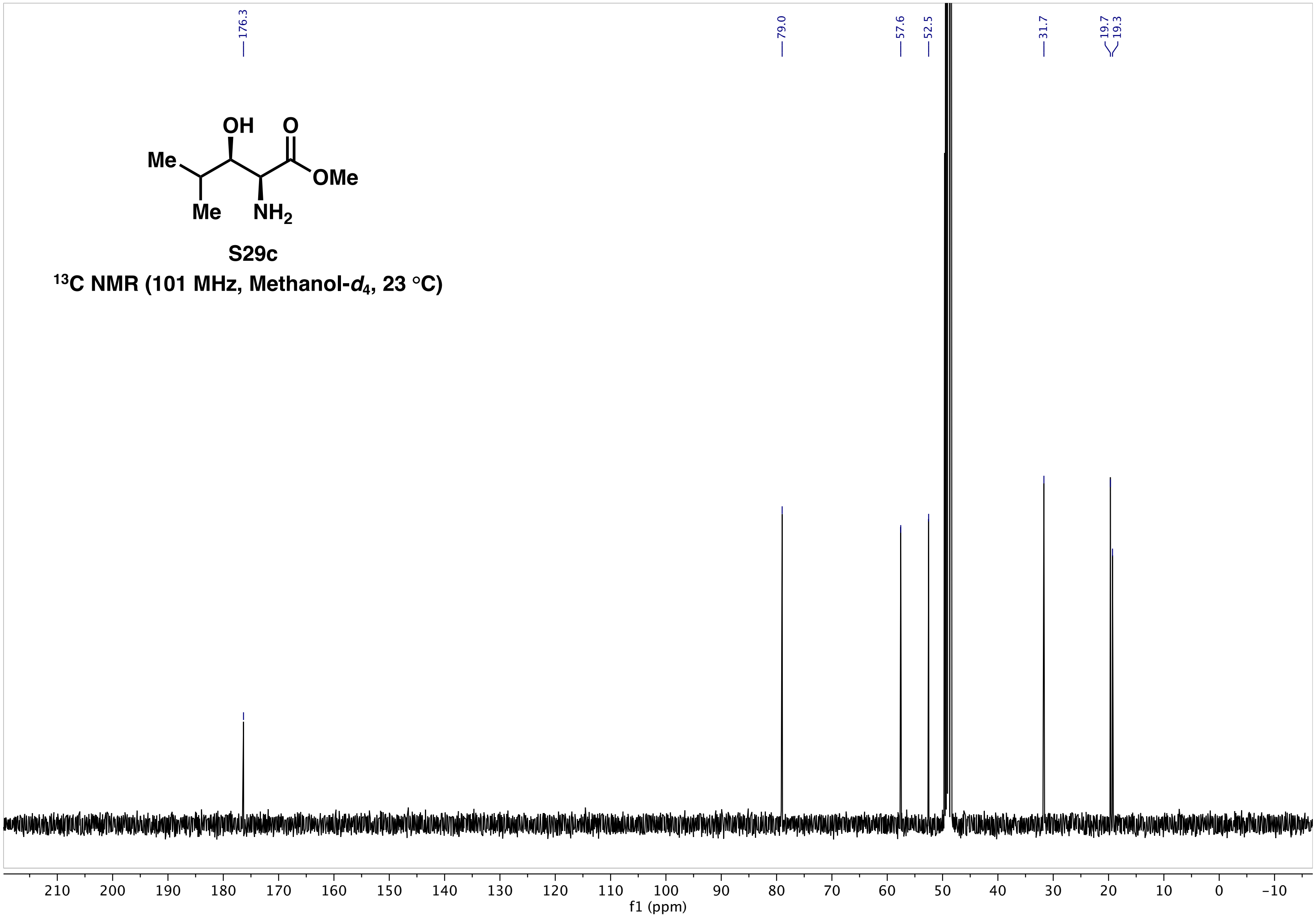
176.3
79.0
57.6
52.5
31.7
19.7
19.3
OH
O
Me
OMe
Me
NH2
S29c
13C NMR (101 MHz, Methanol-d4, 23 °C)
210 200 190 180 170 160 150 140 130 120 110 100 90 80 70 60 50 40 30 20 10 0 –10
f1 (ppm)

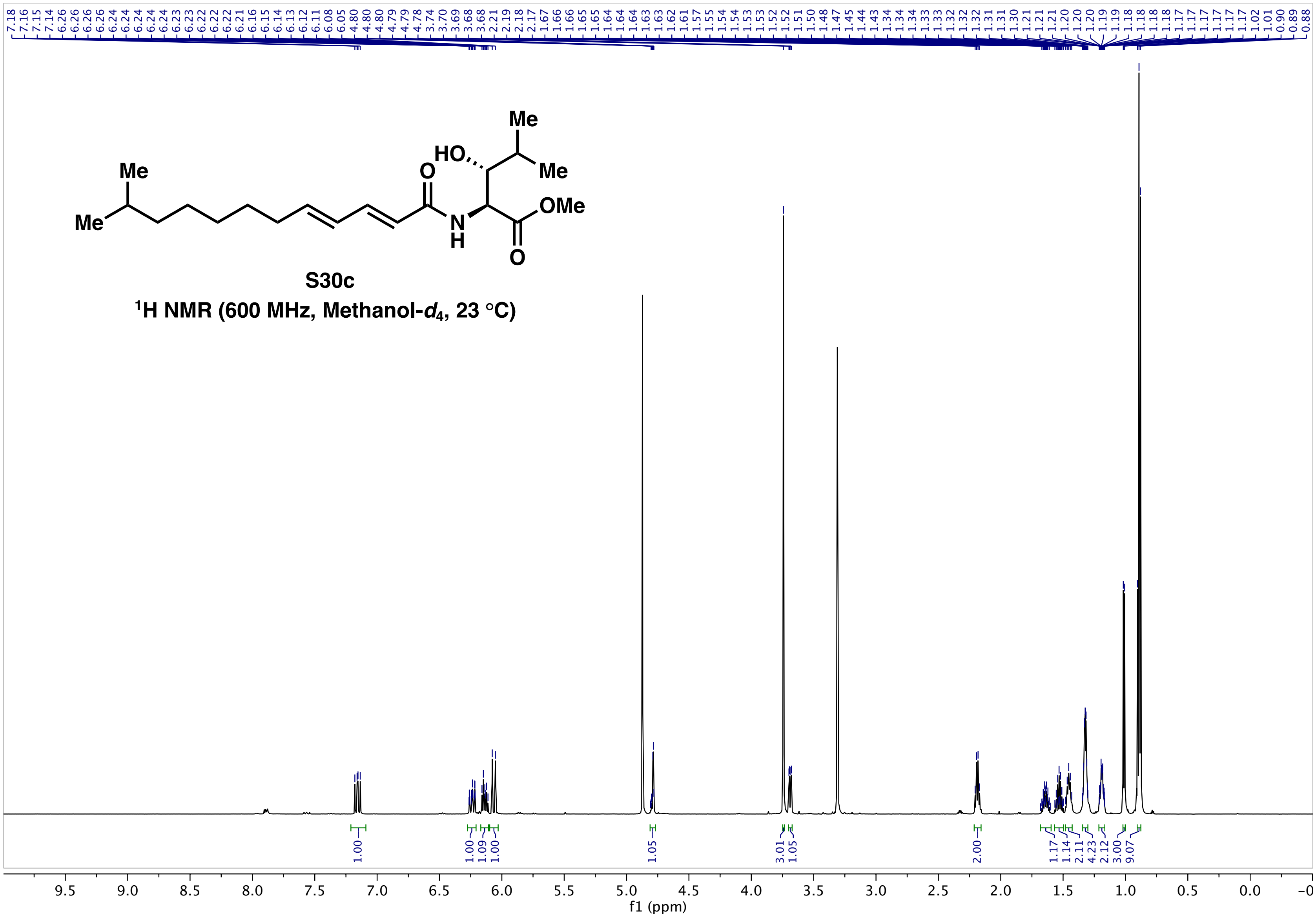
S30c
¹H NMR (600 MHz, Methanol-d_4, 23 °C)
f1 (ppm)

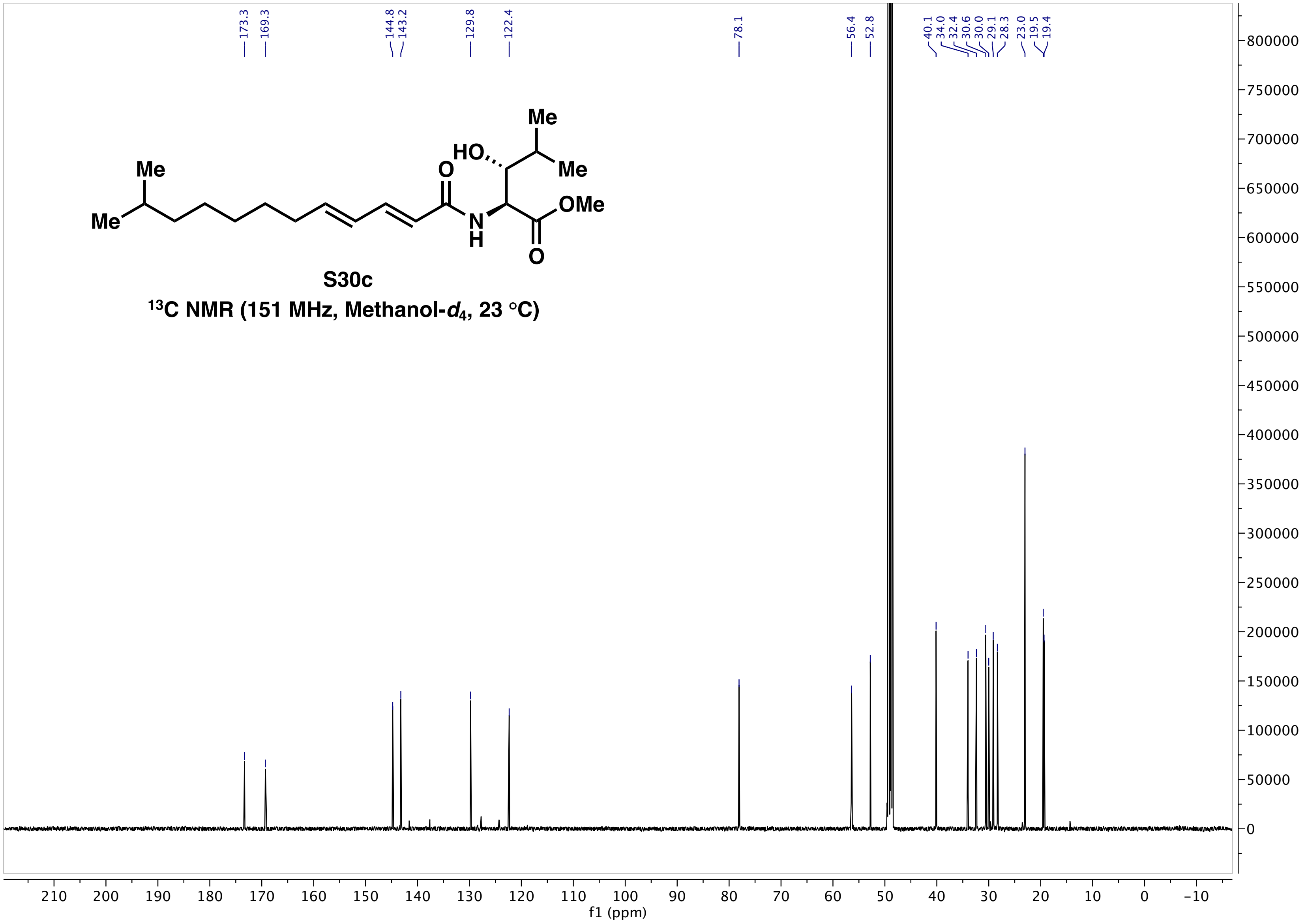
173.3
169.3
144.8
143.2
129.8
122.4
78.1
56.4
52.8
40.1
34.0
32.4
30.6
30.0
29.1
28.3
23.0
19.5
19.4
Me
HO
Me
Me
O
OMe
Me
N
H
O
S30c
^{13}C NMR (151 MHz, Methanol-d_4, 23 °C)
f1 (ppm)

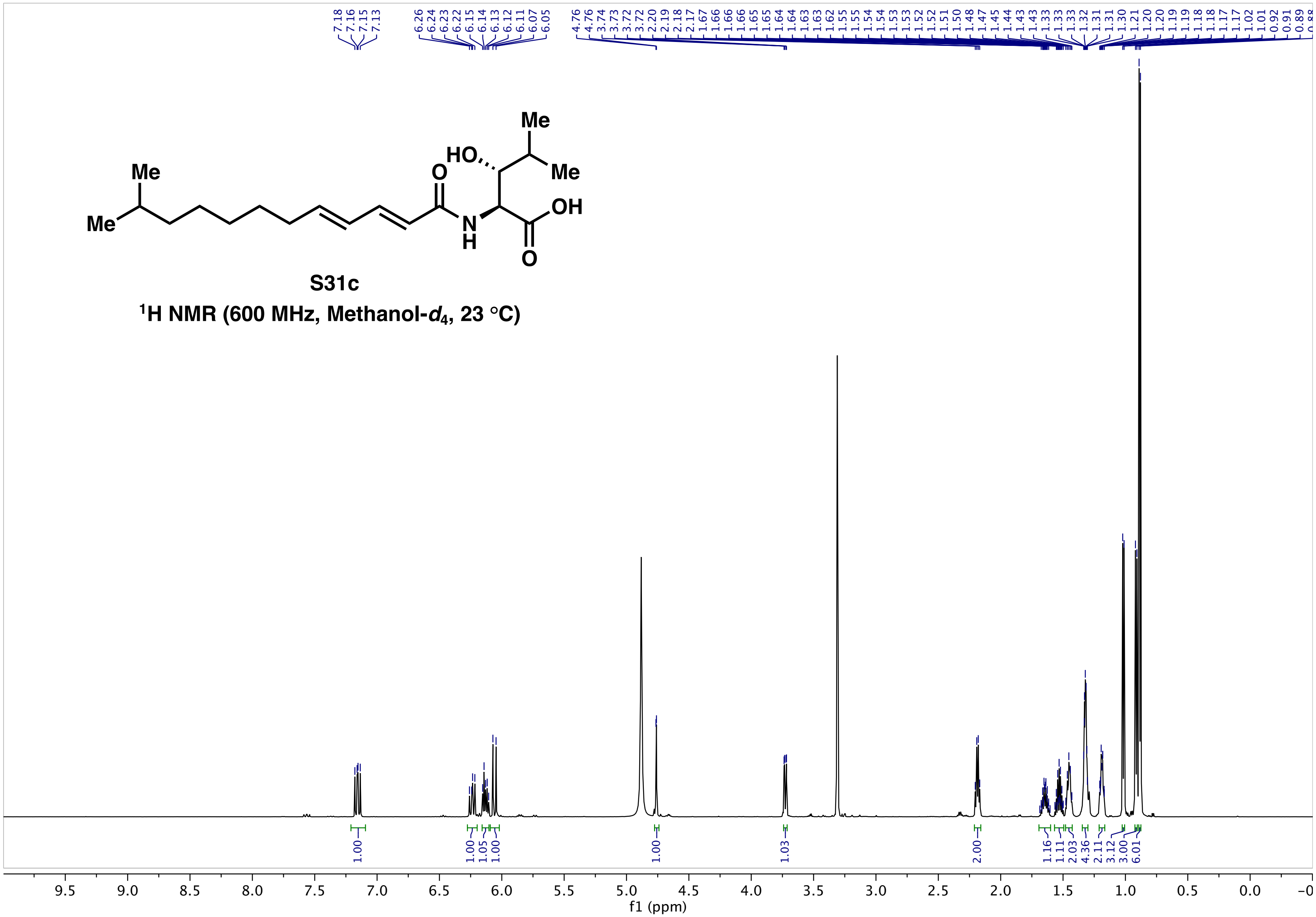

S31c

^{1}H NMR (600 MHz, Methanol-d_4, 23 °C)

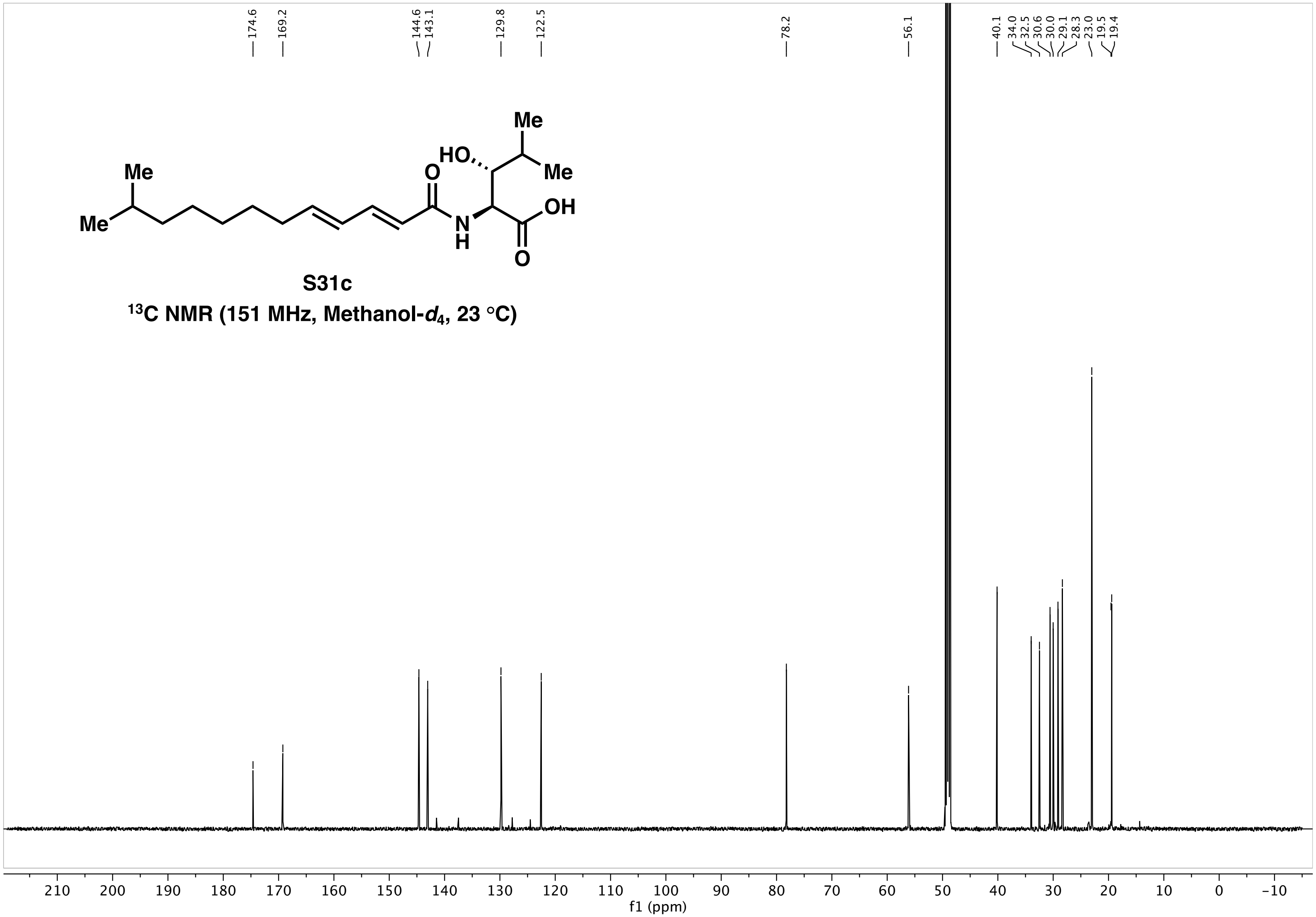
174.6
169.2
144.6
143.1
129.8
122.5
78.2
56.1
40.1
34.0
32.5
30.6
30.0
29.1
28.3
23.0
19.5
19.4
Me
HO
O
Me
Me
OH
Me
N
H
O
S31c
13C NMR (151 MHz, Methanol-d4, 23 °C)
210
200
190
180
170
160
150
140
130
120
110
100
90
80
70
60
50
40
30
20
10
0
–10
f1 (ppm)

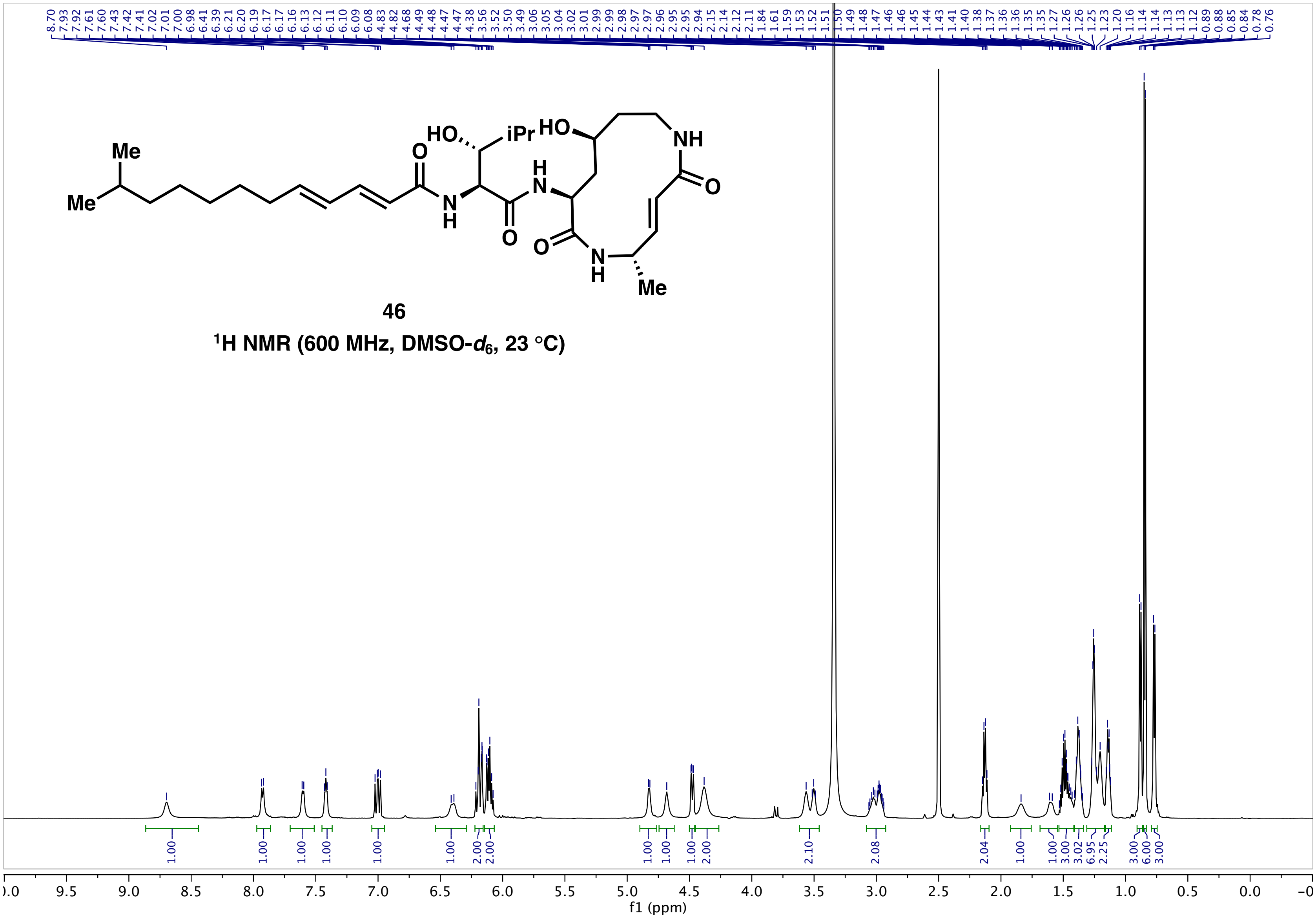
46
1H NMR (600 MHz, DMSO-d6, 23 °C)
f1 (ppm)

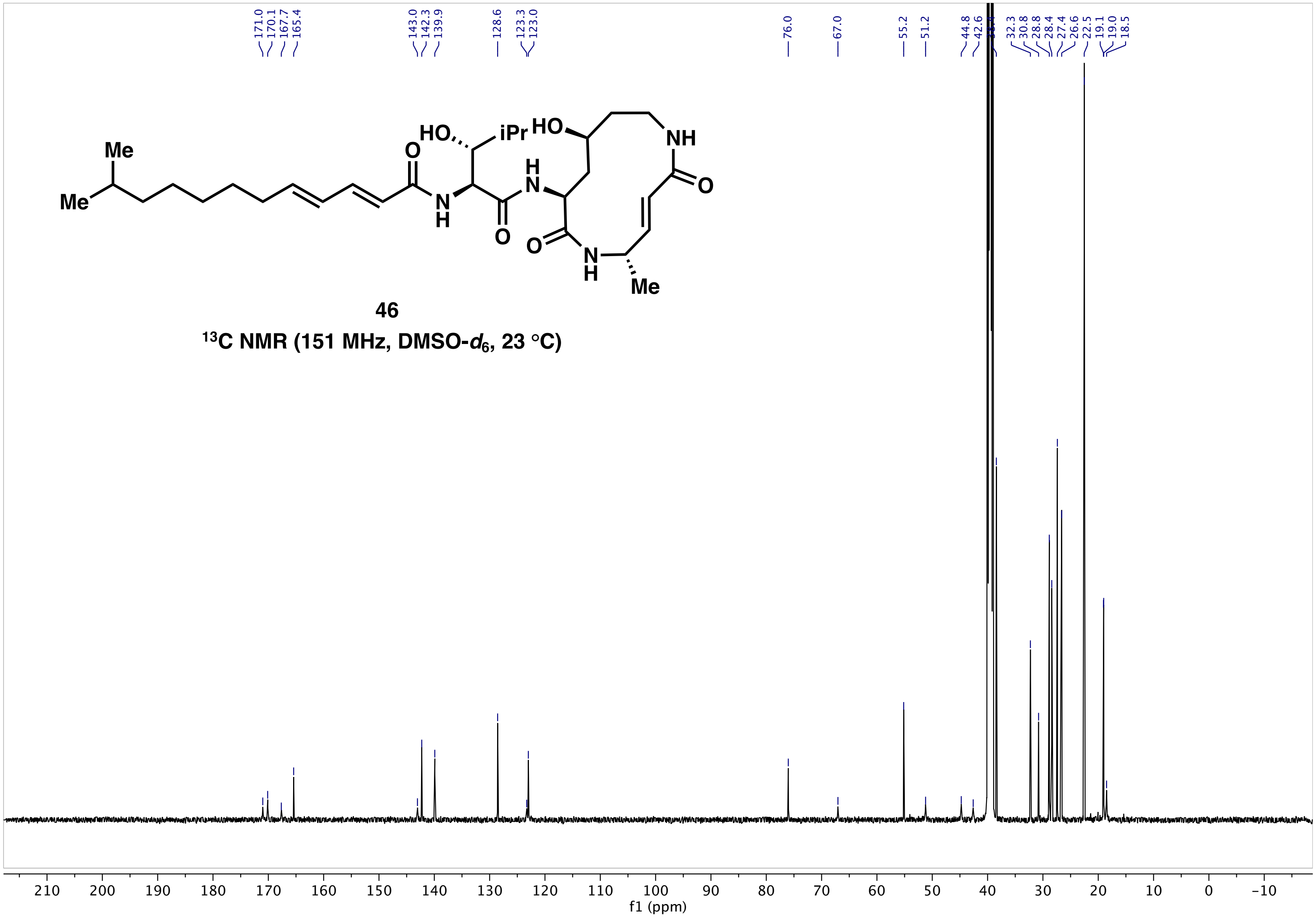

171.0
170.1
167.7
165.4
143.0
142.3
139.9
128.6
123.3
123.0
76.0
67.0
55.2
51.2
44.8
42.6
32.3
30.8
28.8
28.4
27.4
26.6
22.5
19.1
19.0
18.5
Me
Me
O
HO
iPr
HO
NH
O
N
H
N
H
O
O
N
H
Me
46
^{13}C NMR (151 MHz, DMSO-d_6, 23 °C)
210 200 190 180 170 160 150 140 130 120 110 100 90 80 70 60 50 40 30 20 10 0 -10
f1 (ppm)

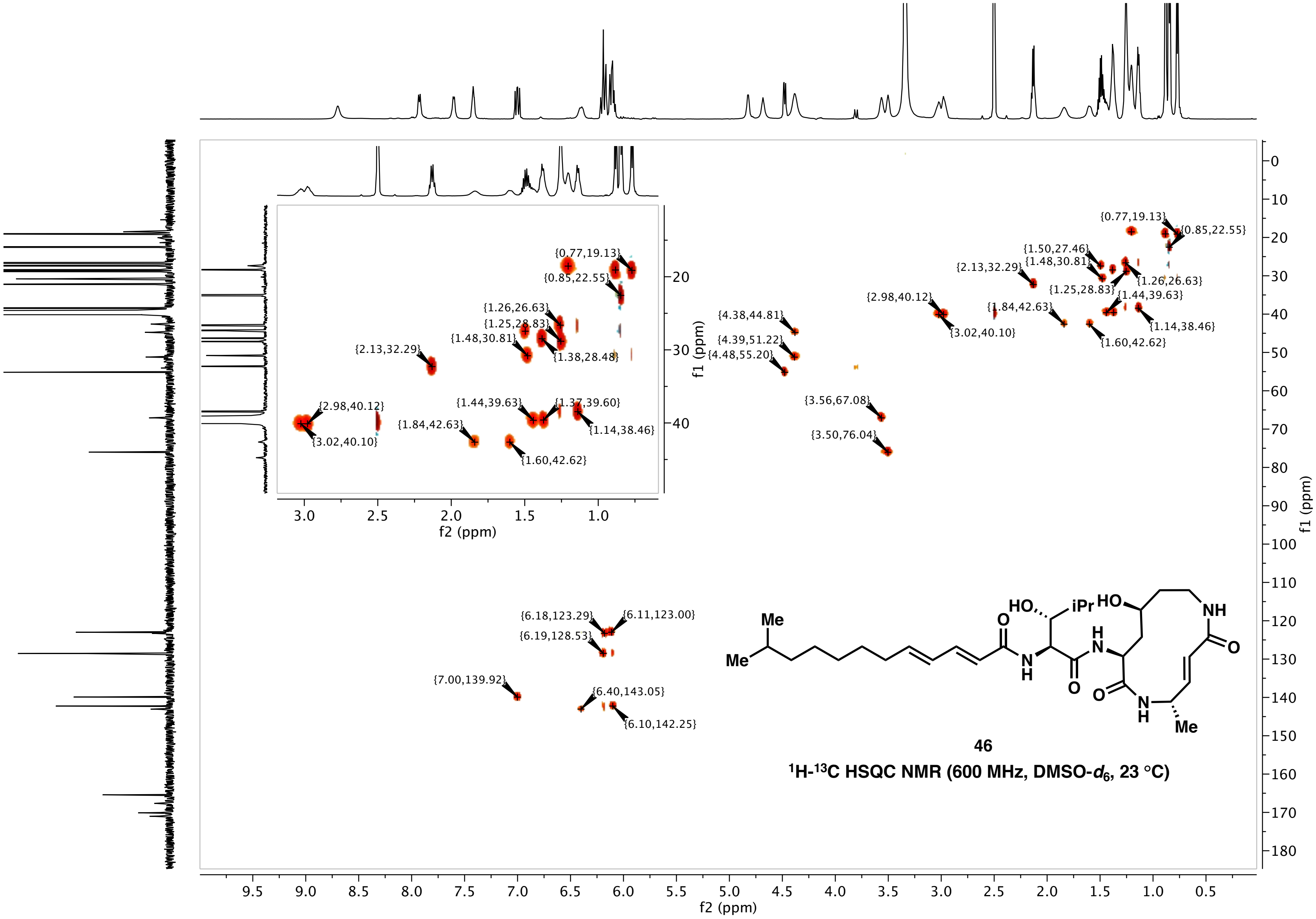

46

^{1}H-^{13}C HSQC NMR (600 MHz, DMSO-d_6, 23 °C)

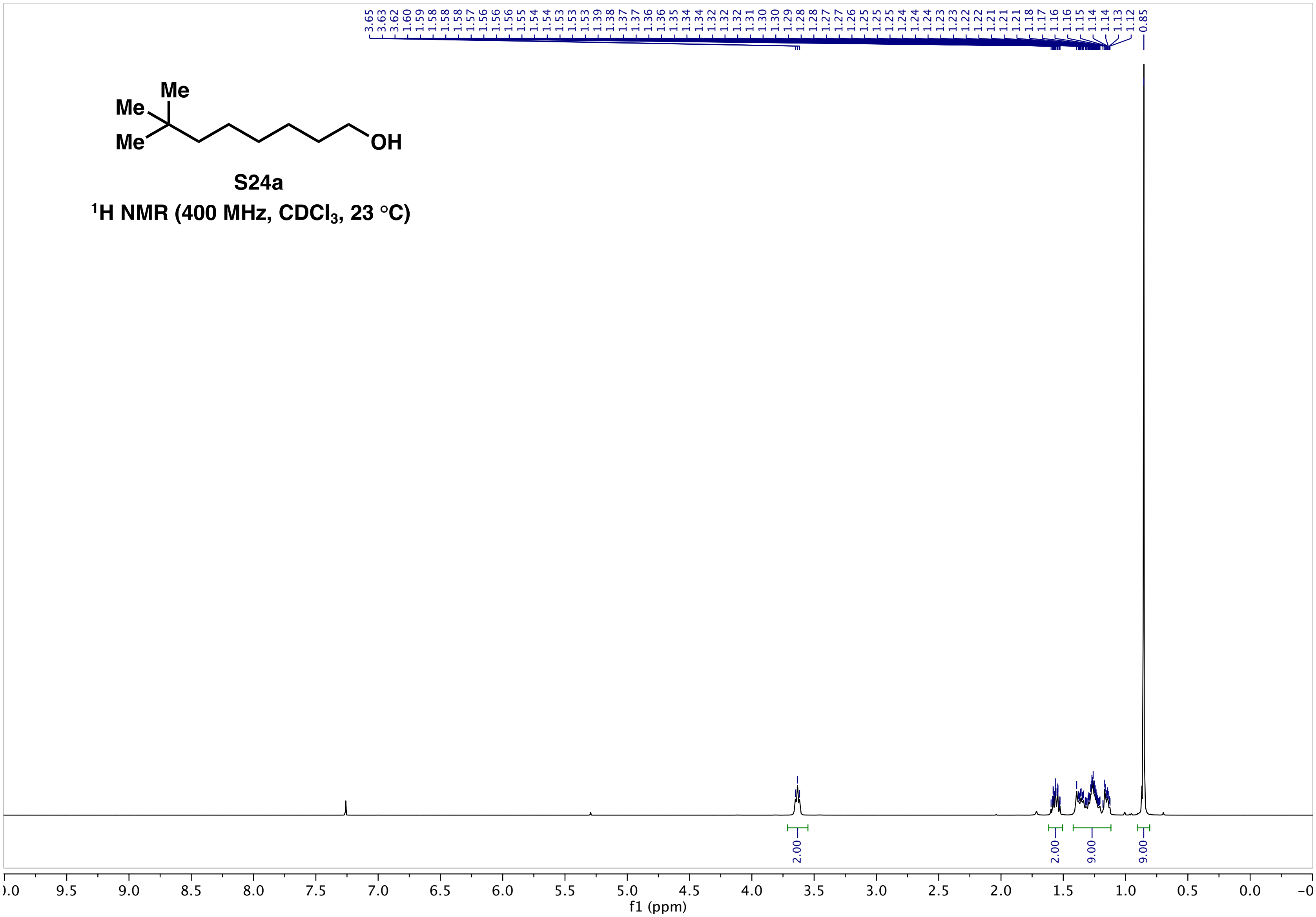
Me
Me
Me
OH
S24a
¹H NMR (400 MHz, CDCl3, 23 °C)
f1 (ppm)

S24a

^{13}C NMR (101 MHz, $CDCl_3$, 23 °C)

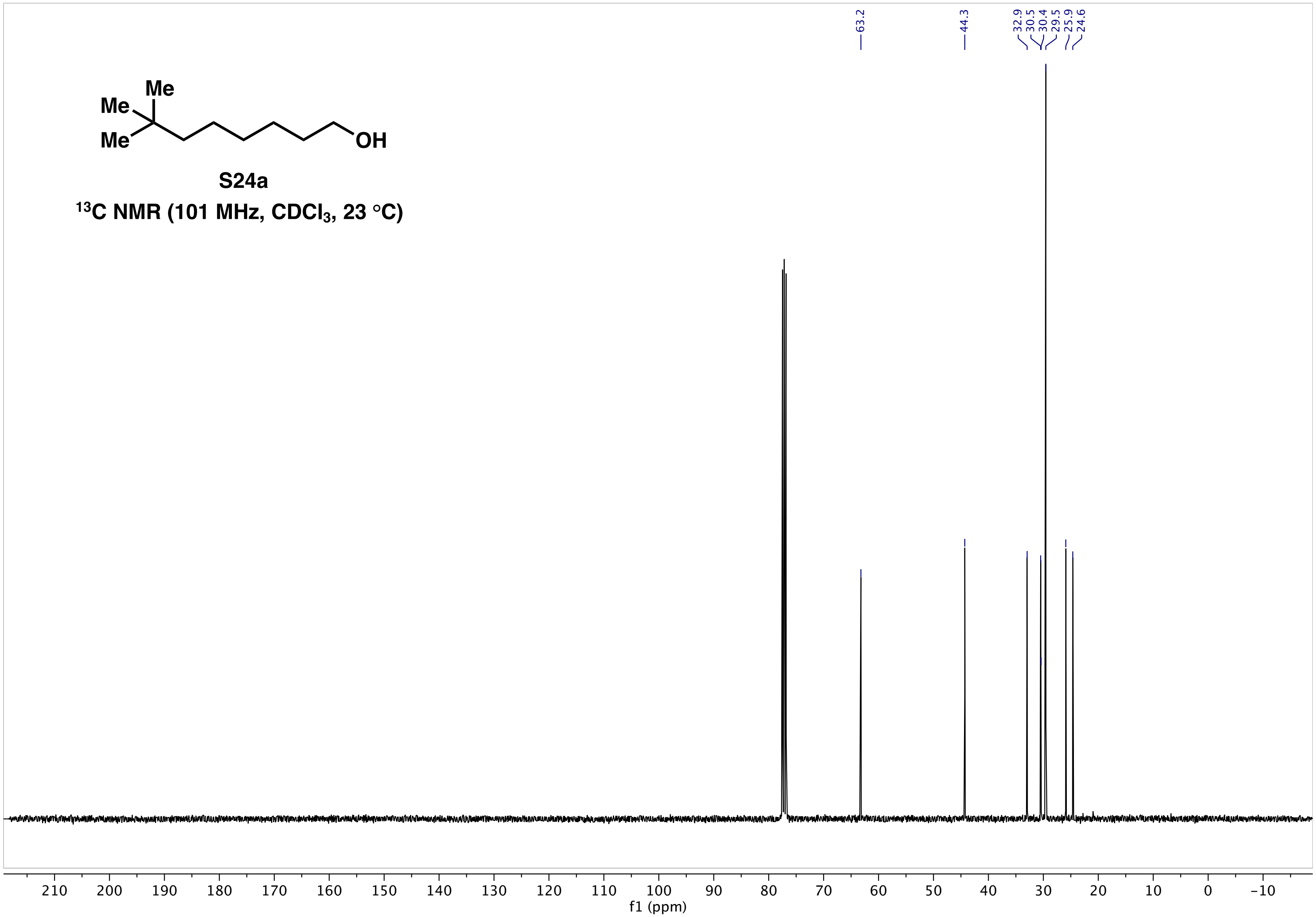

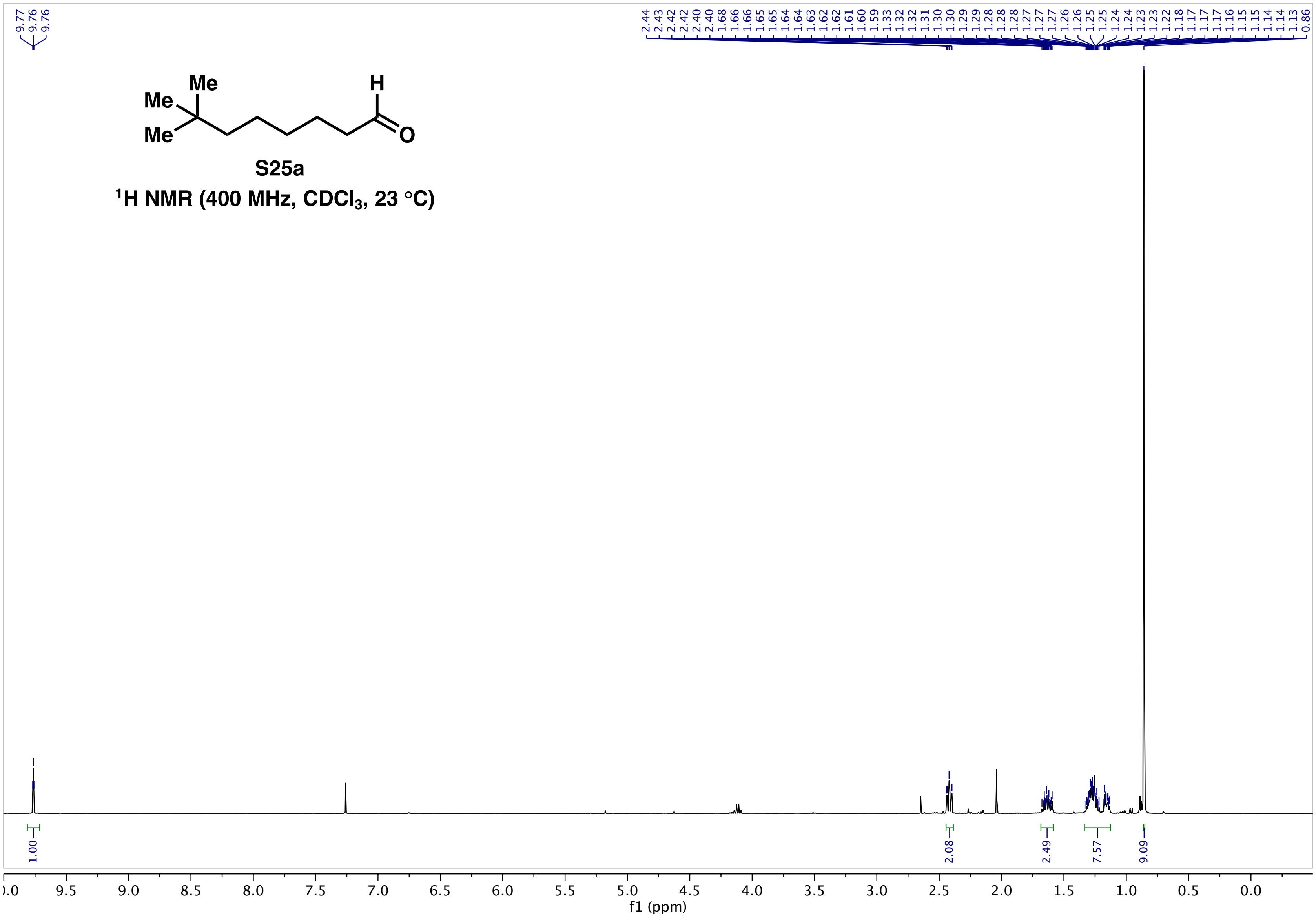

Me
Me
Me
H
O
S25a
^{1}H NMR (400 MHz, CDCl$_3$, 23 °C)
9.77 9.76 9.76
2.44 2.43 2.42 2.42 2.40 1.68 1.66 1.66 1.65 1.64 1.64 1.63 1.62 1.62 1.61 1.60 1.59 1.33 1.32 1.32 1.31 1.30 1.30 1.29 1.29 1.28 1.28 1.28 1.27 1.27 1.27 1.26 1.25 1.25 1.24 1.24 1.23 1.23 1.22 1.18 1.17 1.17 1.17 1.16 1.15 1.15 1.14 1.14 1.13 0.86
1.00
2.08
2.49
7.57
9.09
f1 (ppm)

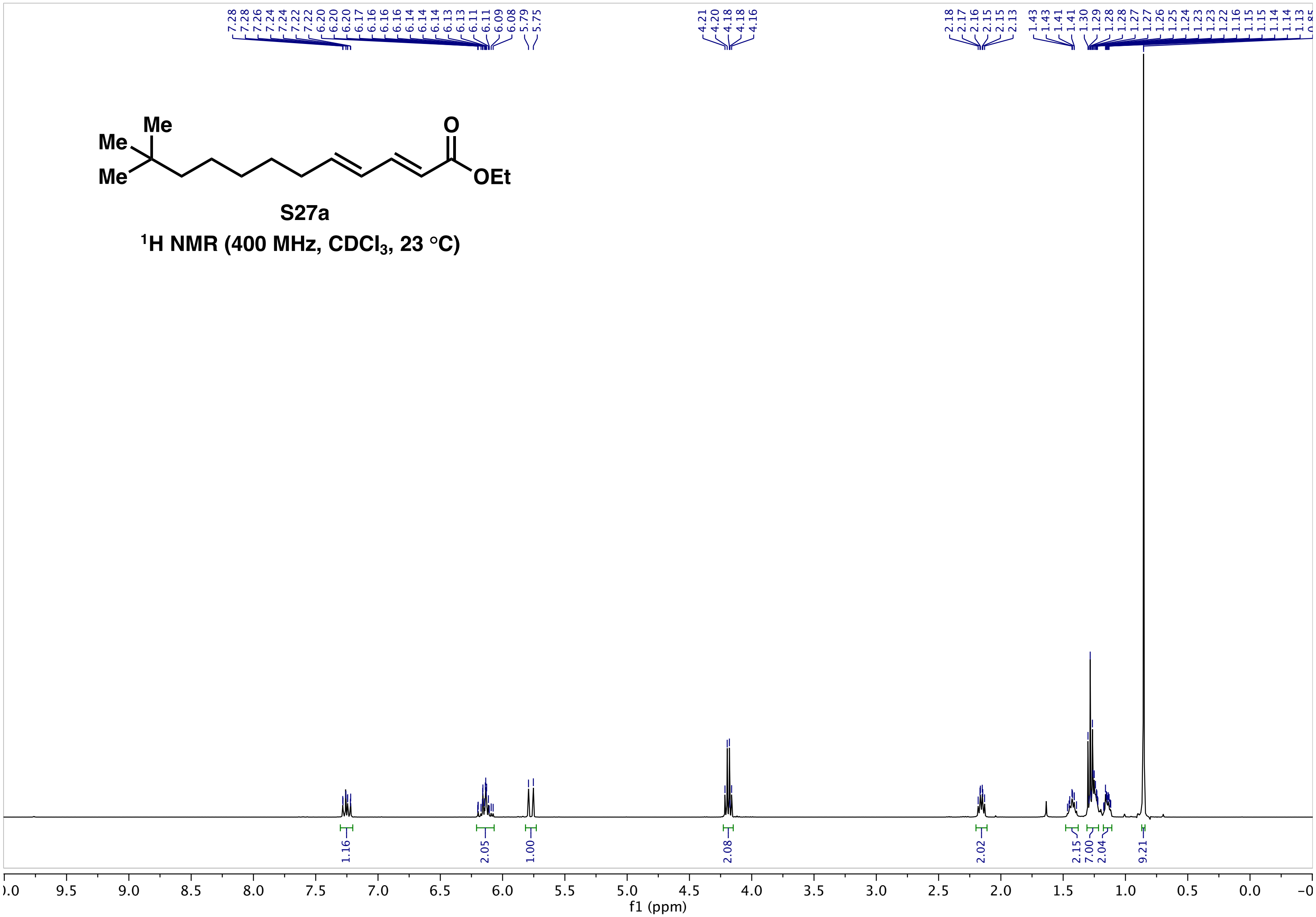

S27a

^{1}H NMR (400 MHz, $CDCl_3$, 23 °C)

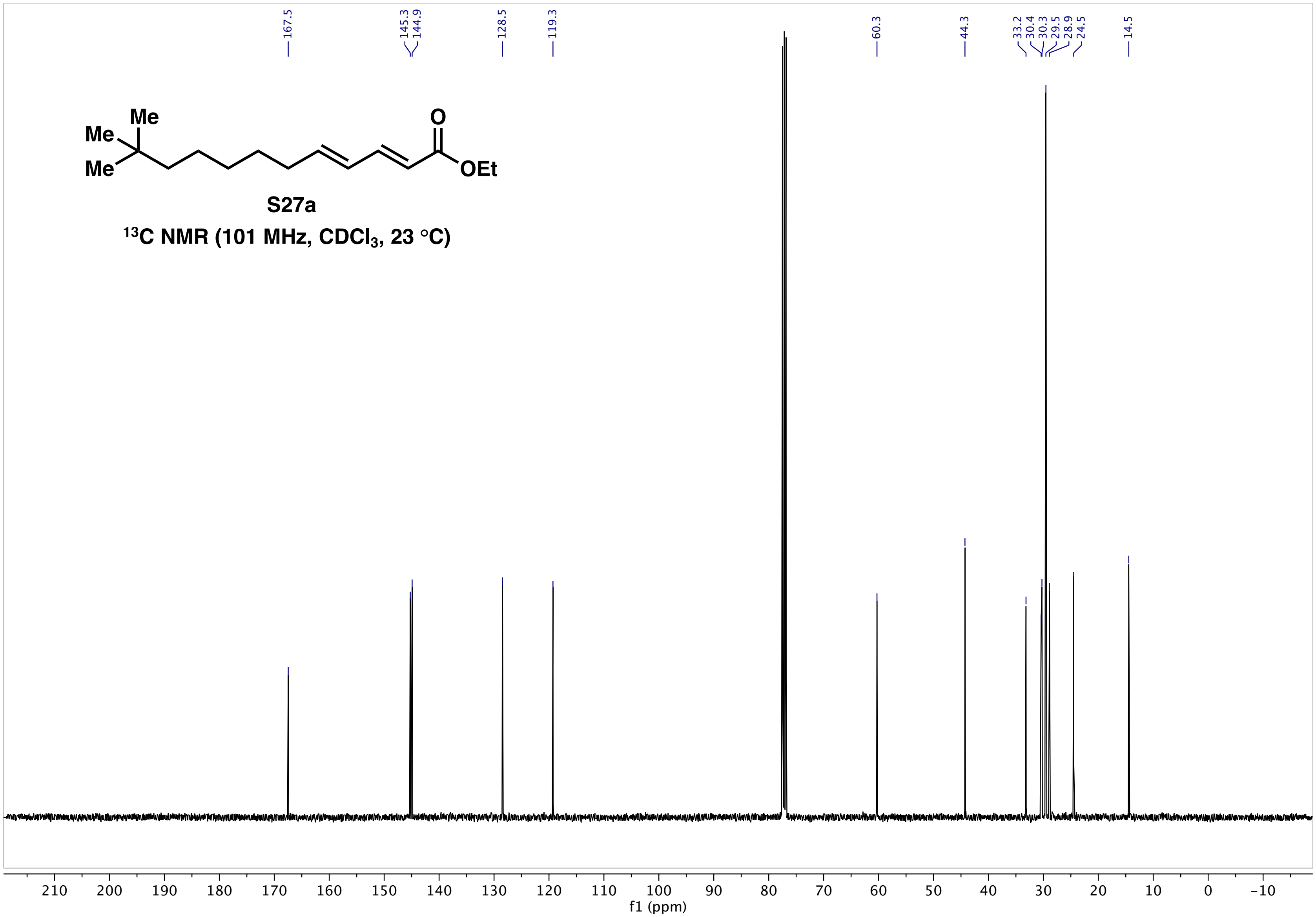

Me
Me
Me
O
OEt
S27a
13C NMR (101 MHz, CDCl3, 23 °C)
167.5
145.3
144.9
128.5
119.3
60.3
44.3
33.2
30.4
30.3
29.5
28.9
24.5
14.5
210 200 190 180 170 160 150 140 130 120 110 100 90 80 70 60 50 40 30 20 10 0 −10
f1 (ppm)

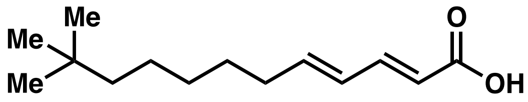

S28b

^{1}H NMR (400 MHz, $CDCl_3$, 23 °C)

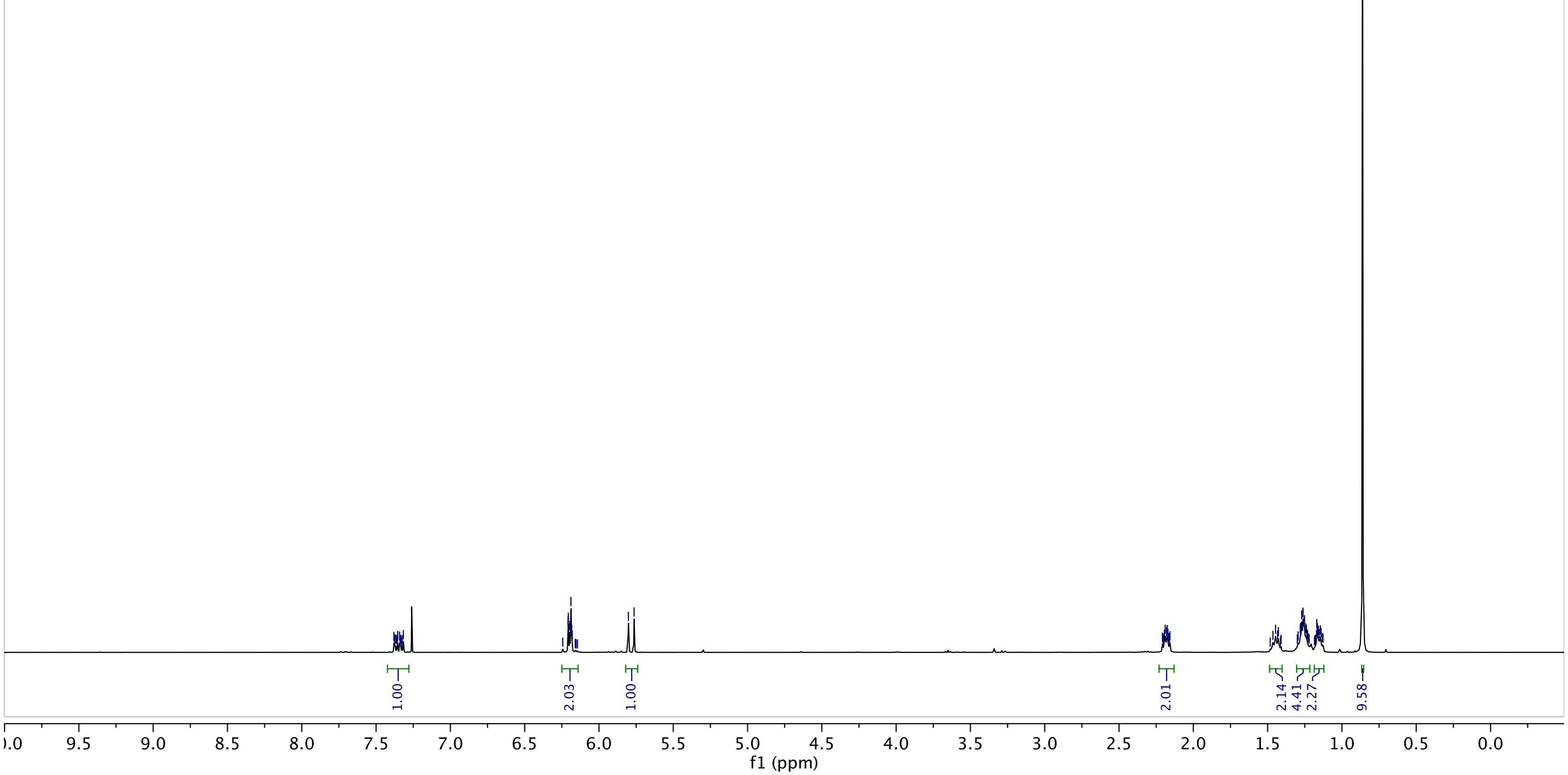

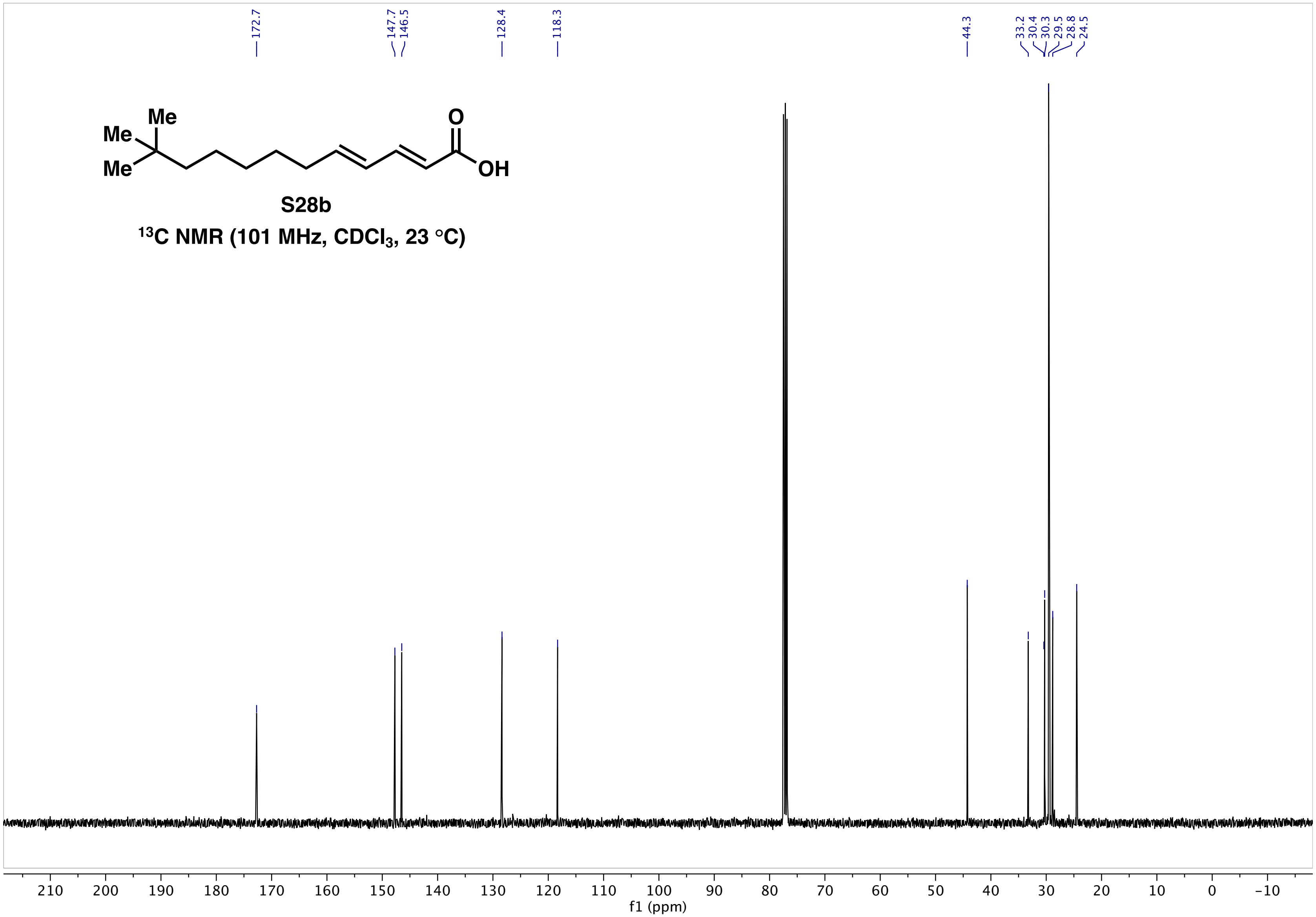
Me
Me
Me
O
OH
S28b
^{13}C NMR (101 MHz, $CDCl_3$, 23 °C)
172.7
147.7
146.5
128.4
118.3
44.3
33.2
30.4
30.3
29.5
28.8
24.5
f1 (ppm)

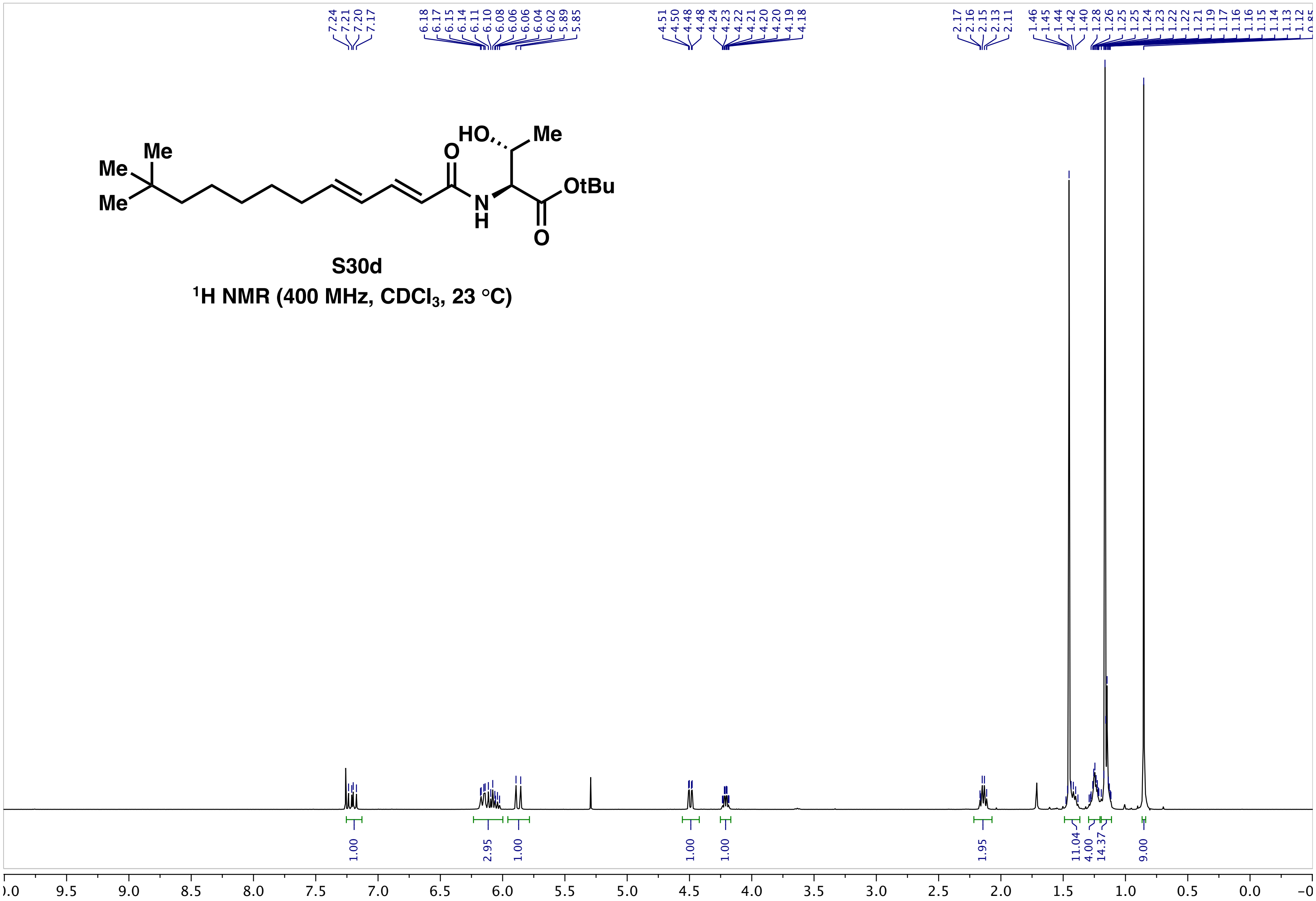

S30d

^{1}H NMR (400 MHz, $CDCl_3$, 23 °C)

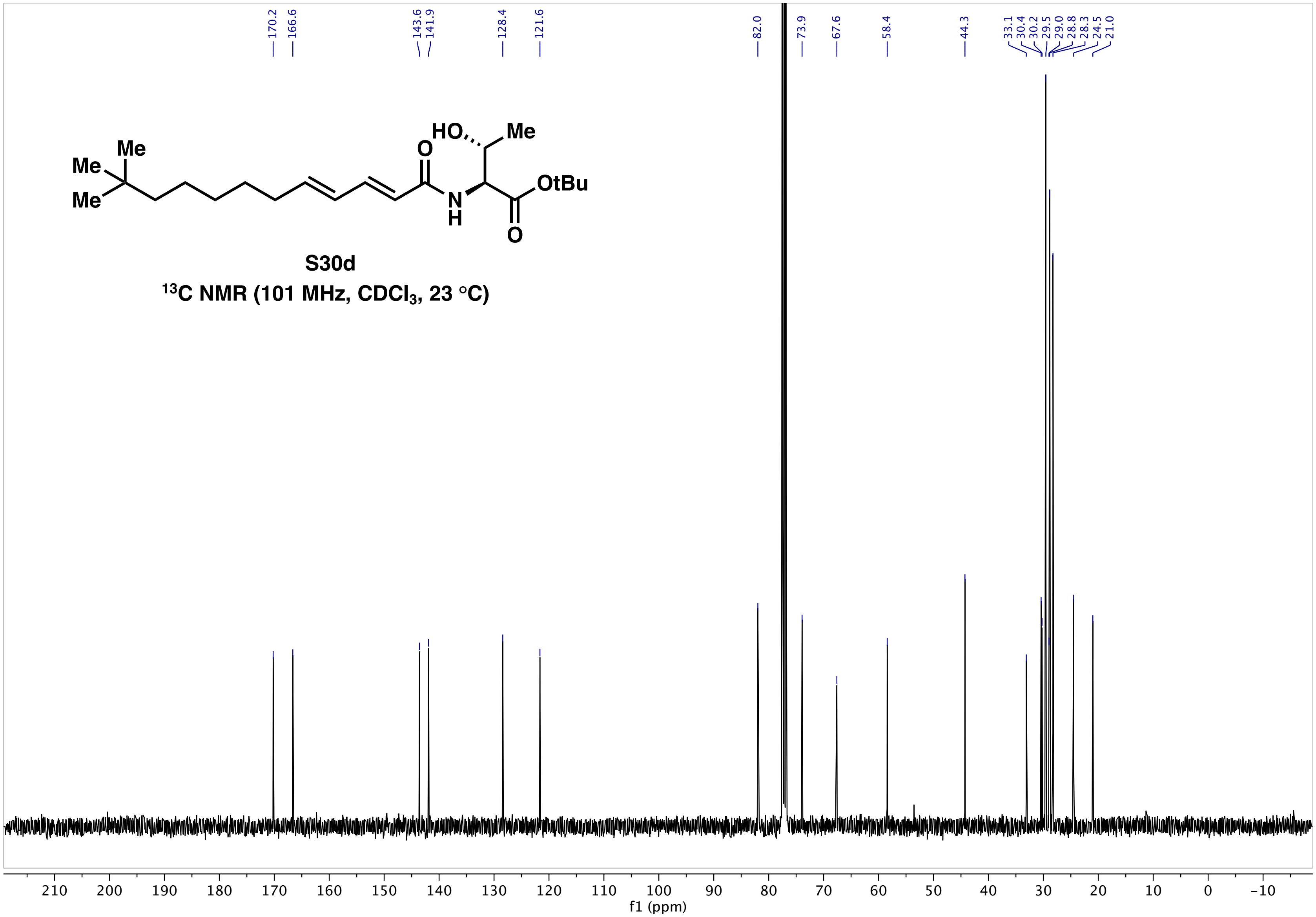
170.2
166.6
143.6
141.9
128.4
121.6
82.0
73.9
67.6
58.4
44.3
33.1
30.4
30.2
29.5
29.0
28.8
28.3
24.5
21.0
Me
Me
Me
O
HO
Me
N
H
OtBu
O
S30d
^{13}C NMR (101 MHz, $CDCl_3$, 23 °C)
210
200
190
180
170
160
150
140
130
120
110
100
90
80
70
60
50
40
30
20
10
0
–10
f1 (ppm)

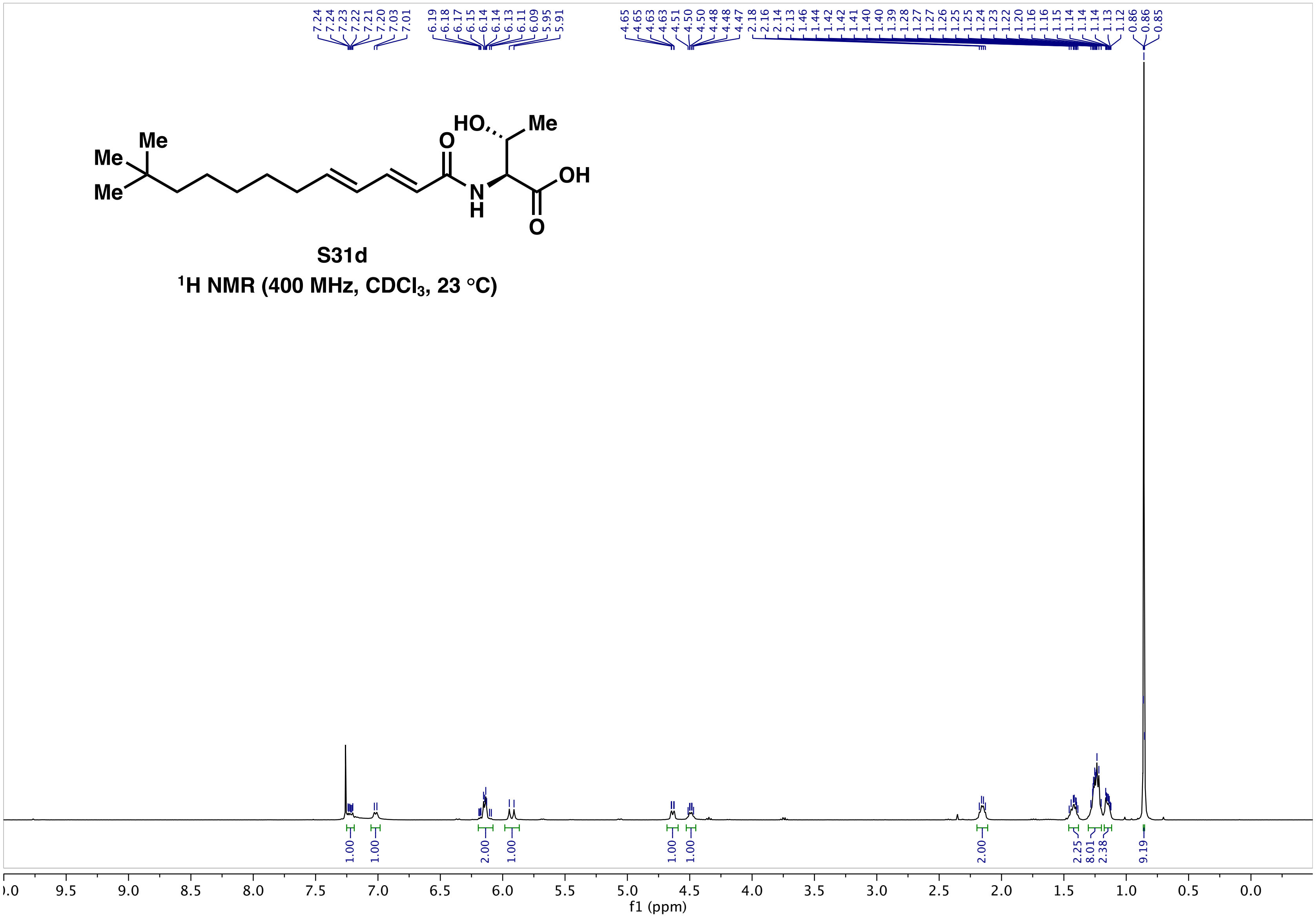

S31d

^{1}H NMR (400 MHz, $CDCl_3$, 23 °C)

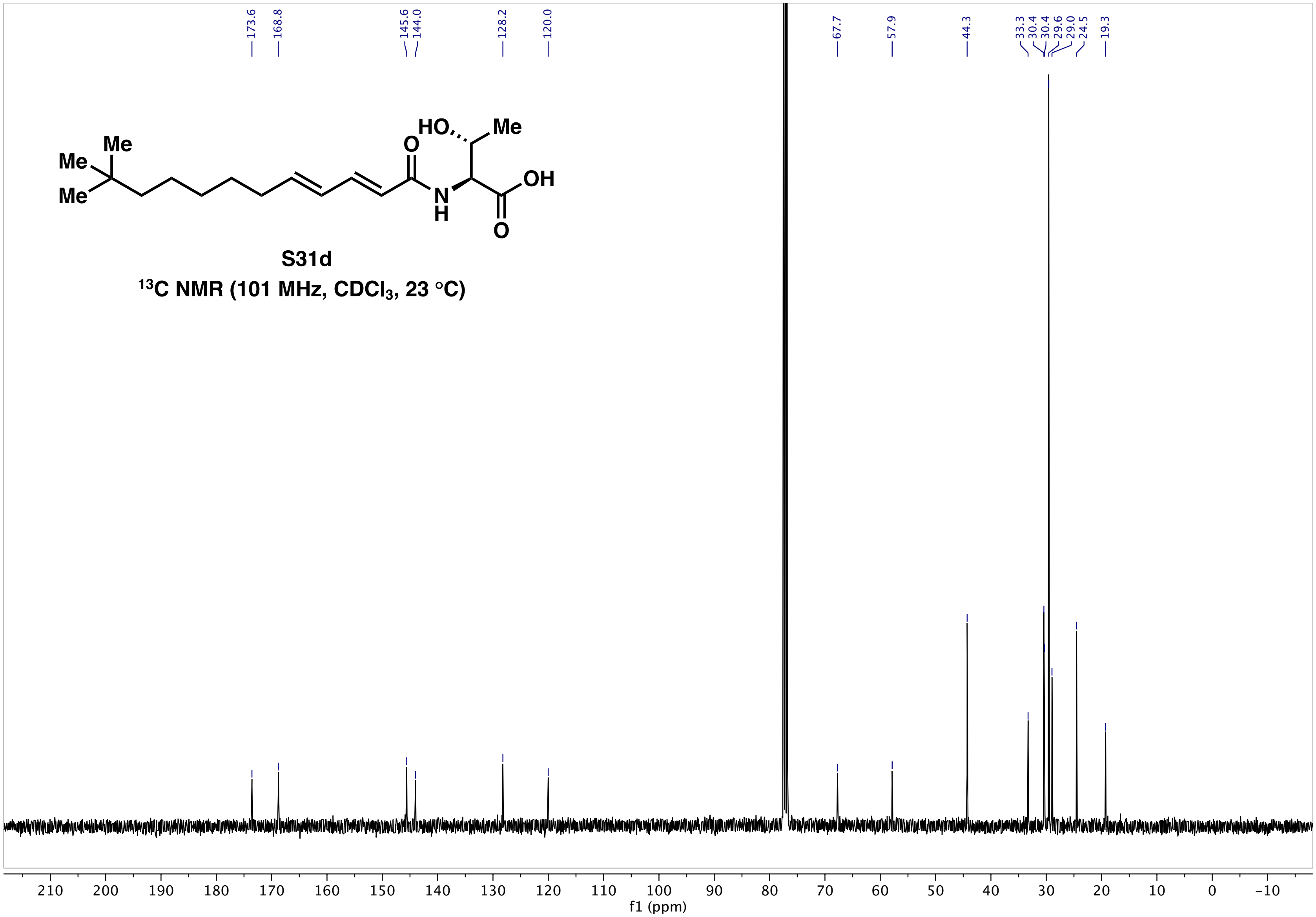
173.6
168.8
145.6
144.0
128.2
120.0
67.7
57.9
44.3
33.3
30.4
30.4
29.6
29.0
24.5
19.3
Me
Me
Me
O
HO
Me
N
H
OH
O
S31d
^{13}C NMR (101 MHz, $CDCl_3$, 23 °C)
210
200
190
180
170
160
150
140
130
120
110
100
90
80
70
60
50
40
30
20
10
0
–10
f1 (ppm)

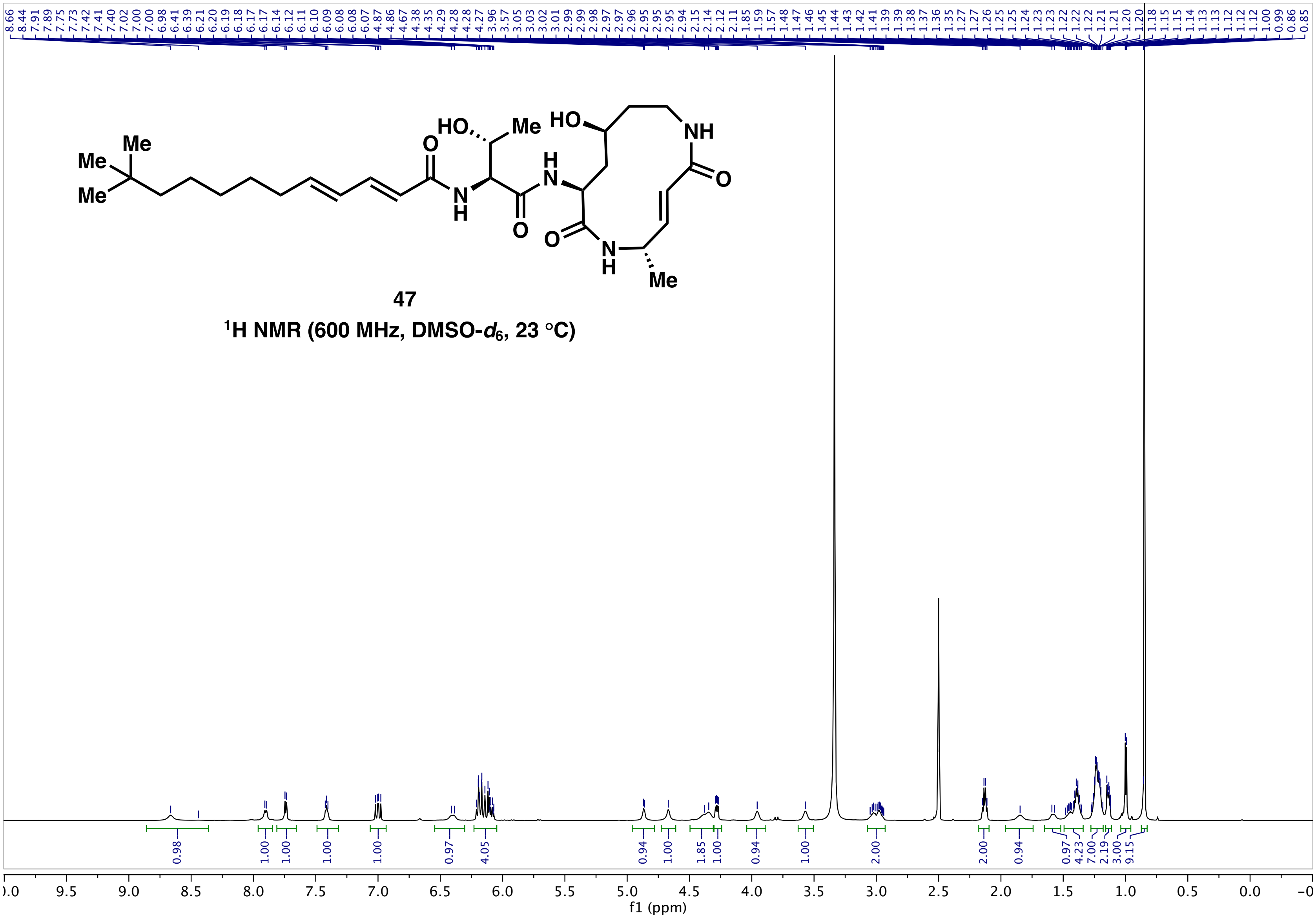

47

^{1}H NMR (600 MHz, DMSO-d_6, 23 °C)

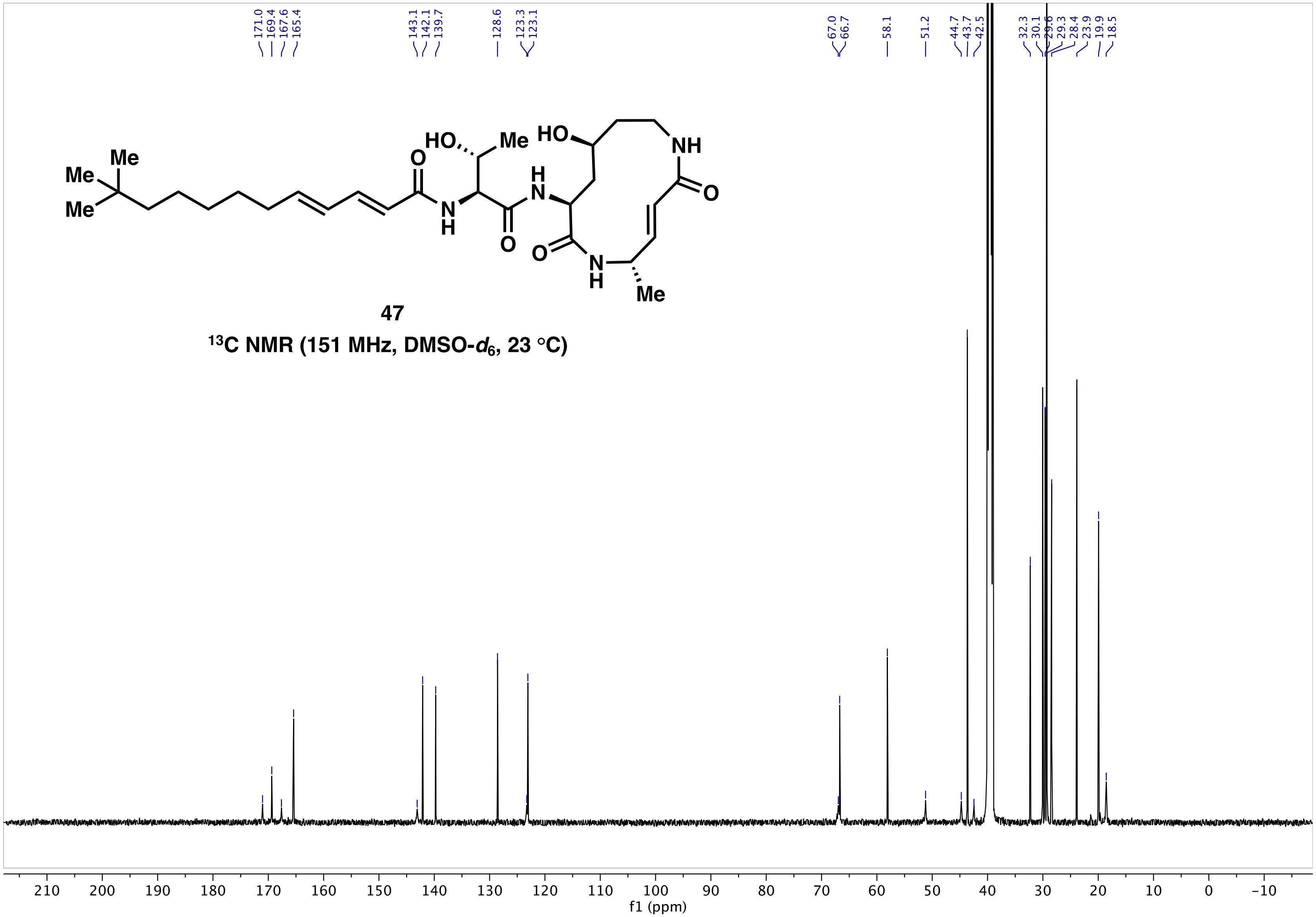
171.0
169.4
167.6
165.4
143.1
142.1
139.7
128.6
123.3
123.1
67.0
66.7
58.1
51.2
44.7
43.7
42.5
32.3
30.1
29.6
29.3
28.4
23.9
19.9
18.5
Me
Me
Me
O
N
H
HO
Me
O
H
N
HO
NH
O
O
N
H
Me
47
^{13}C NMR (151 MHz, DMSO-d_6, 23 °C)
210 200 190 180 170 160 150 140 130 120 110 100 90 80 70 60 50 40 30 20 10 0 -10
f1 (ppm)

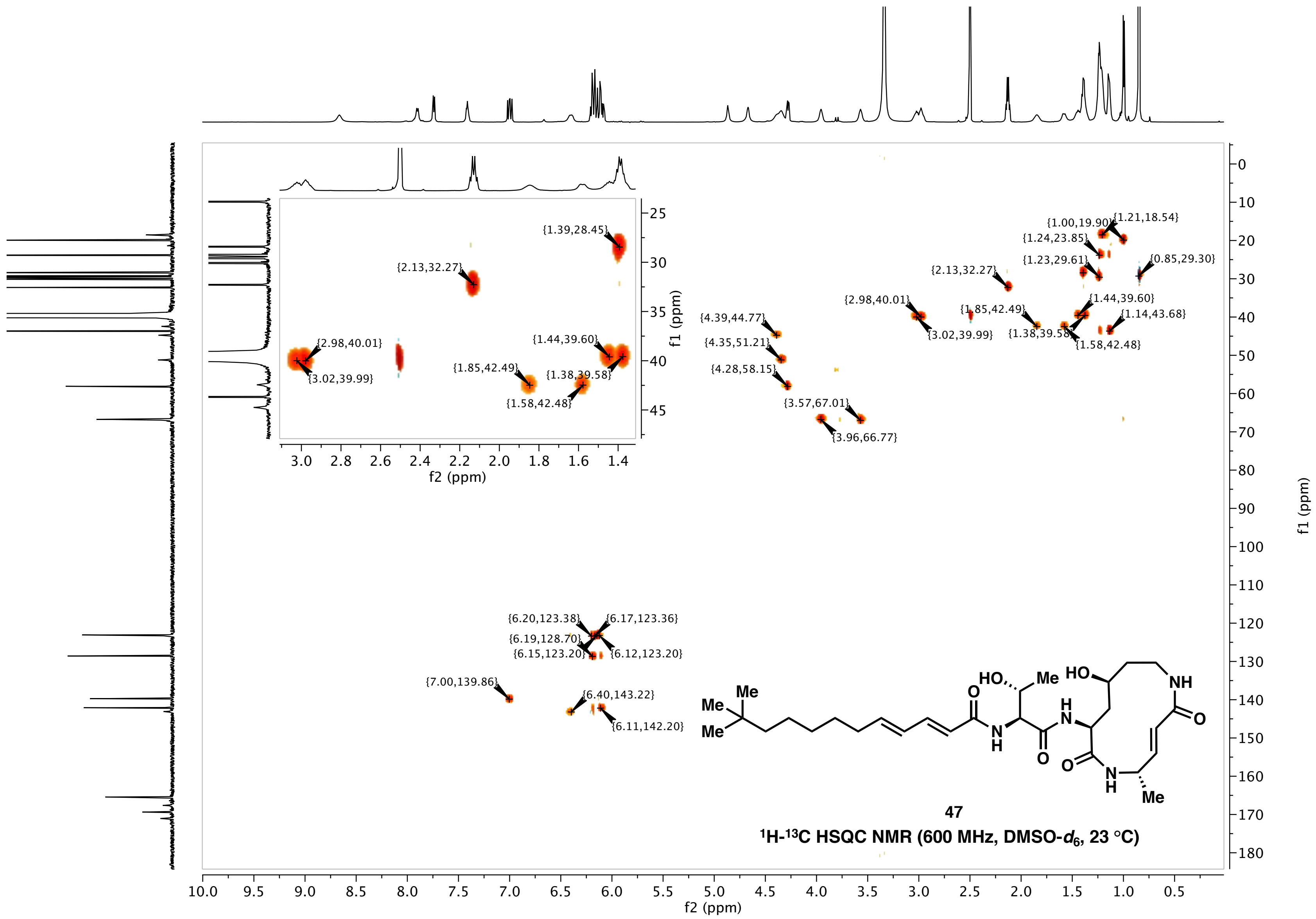

47
¹H-¹³C HSQC NMR (600 MHz, DMSO-d6, 23 °C)
f2 (ppm)
f1 (ppm)
{7.00,139.86}
{6.40,143.22}
{6.11,142.20}
{6.20,123.38}
{6.17,123.36}
{6.19,128.70}
{6.15,123.20}
{6.12,123.20}
{4.39,44.77}
{4.35,51.21}
{4.28,58.15}
{3.57,67.01}
{3.96,66.77}
{2.98,40.01}
{3.02,39.99}
{2.13,32.27}
{1.85,42.49}
{1.38,39.58}
{1.58,42.48}
{1.44,39.60}
{1.14,43.68}
{1.39,28.45}
{1.00,19.90}
{1.21,18.54}
{1.24,23.85}
{1.23,29.61}
{0.85,29.30}

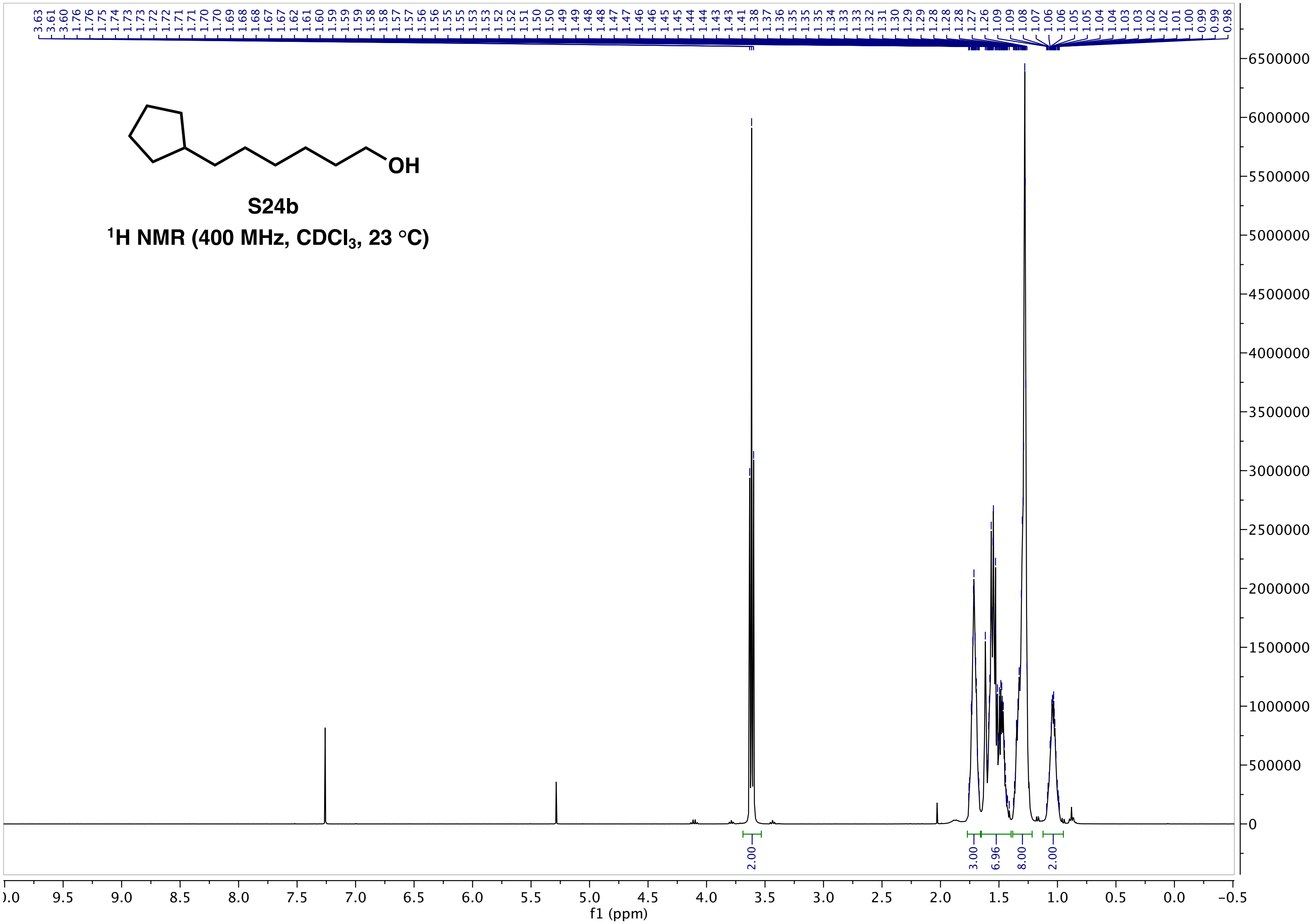

OH
S24b
1H NMR (400 MHz, CDCl3, 23 °C)
3.63 3.61 3.60 1.76 1.76 1.75 1.74 1.73 1.73 1.72 1.72 1.71 1.71 1.70 1.69 1.68 1.68 1.67 1.67 1.62 1.61 1.60 1.59 1.59 1.59 1.58 1.58 1.57 1.57 1.56 1.56 1.55 1.55 1.53 1.53 1.53 1.52 1.52 1.51 1.50 1.50 1.49 1.49 1.48 1.48 1.47 1.47 1.46 1.45 1.45 1.44 1.44 1.43 1.43 1.41 1.38 1.37 1.36 1.35 1.35 1.35 1.34 1.33 1.33 1.32 1.31 1.30 1.29 1.29 1.28 1.28 1.28 1.27 1.26 1.09 1.09 1.08 1.07 1.06 1.06 1.05 1.05 1.04 1.04 1.03 1.03 1.02 1.02 1.01 1.00 0.99 0.99 0.98
2.00
3.00
6.96
8.00
2.00
f1 (ppm)

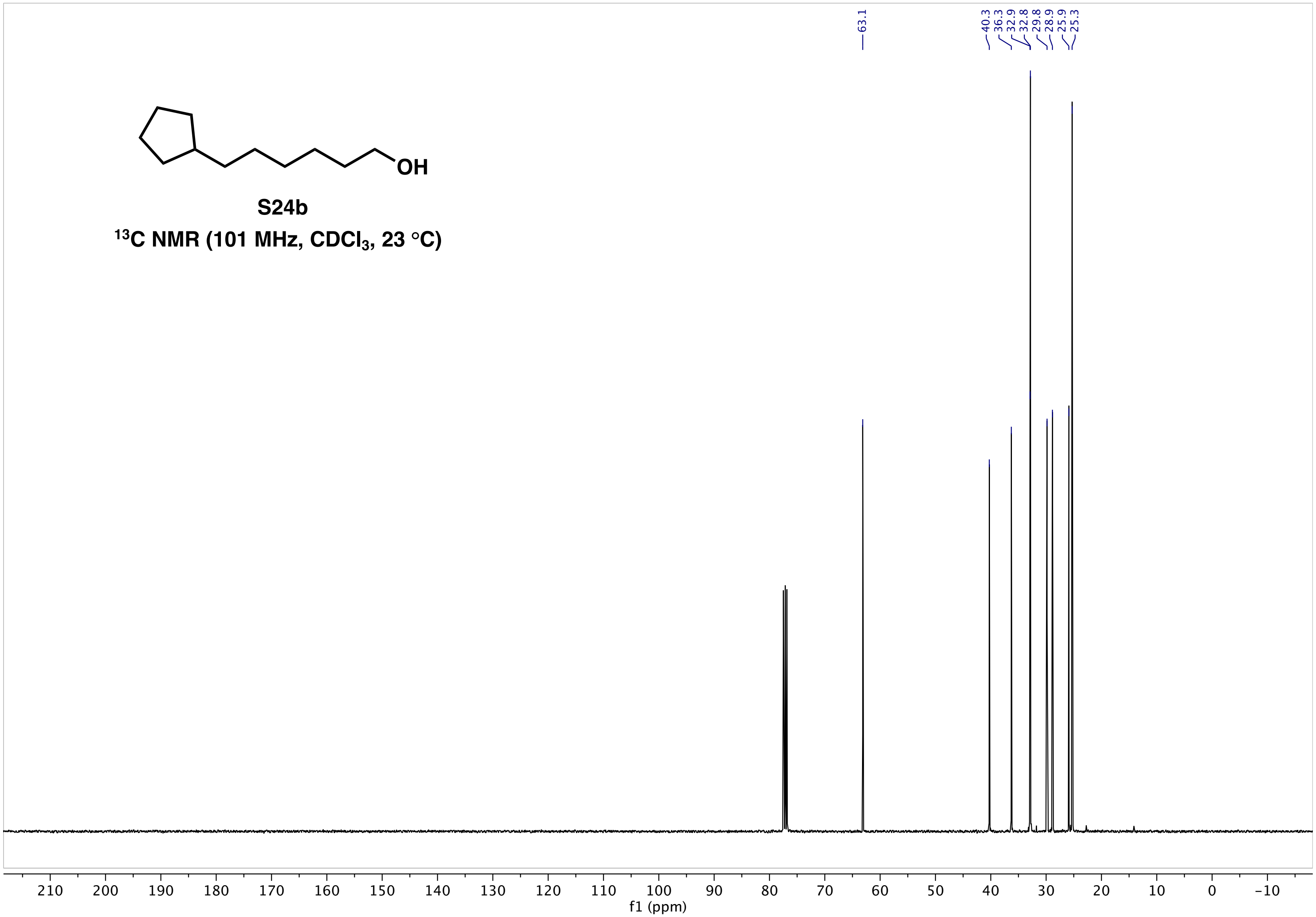

OH
S24b
^{13}C NMR (101 MHz, $CDCl_3$, 23 °C)
63.1
40.3
36.3
32.9
32.8
29.8
28.9
25.9
25.3
210 200 190 180 170 160 150 140 130 120 110 100 90 80 70 60 50 40 30 20 10 0 −10
f1 (ppm)

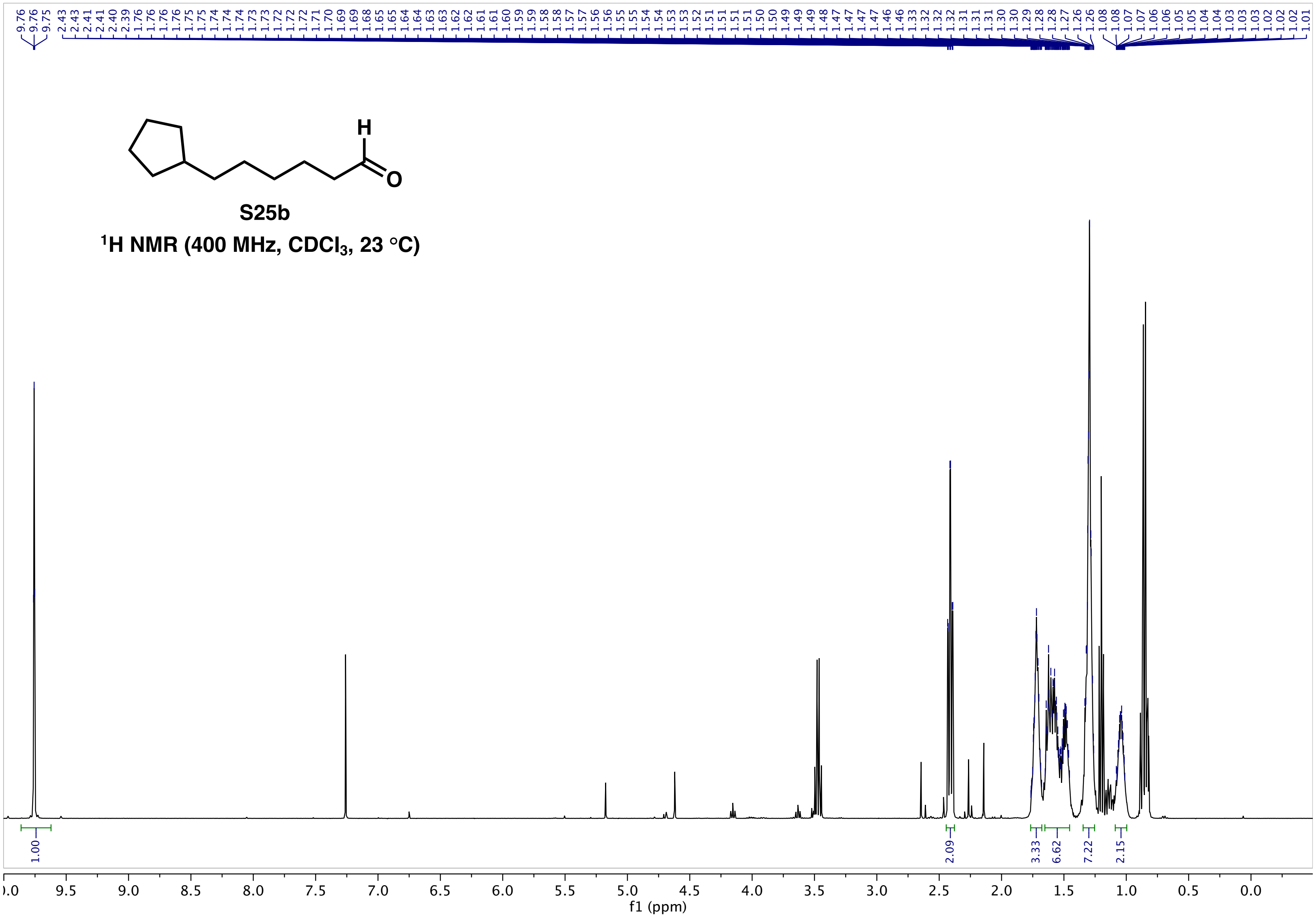

H
O
S25b
1H NMR (400 MHz, CDCl3, 23 °C)
1.00
2.09
3.33
6.62
7.22
2.15
f1 (ppm)

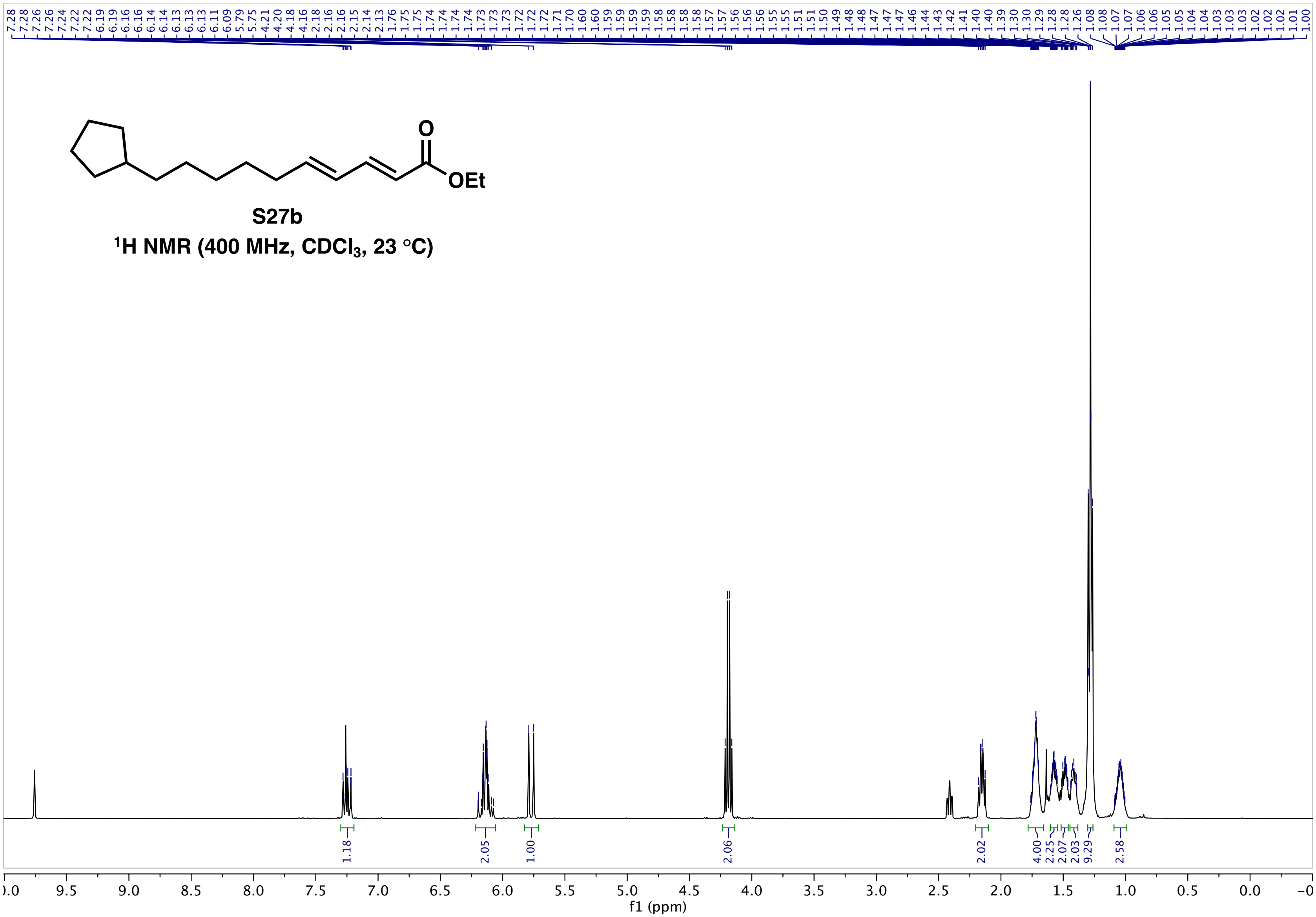
O
OEt
S27b
^{1}H NMR (400 MHz, CDCl$_3$, 23 °C)
f1 (ppm)

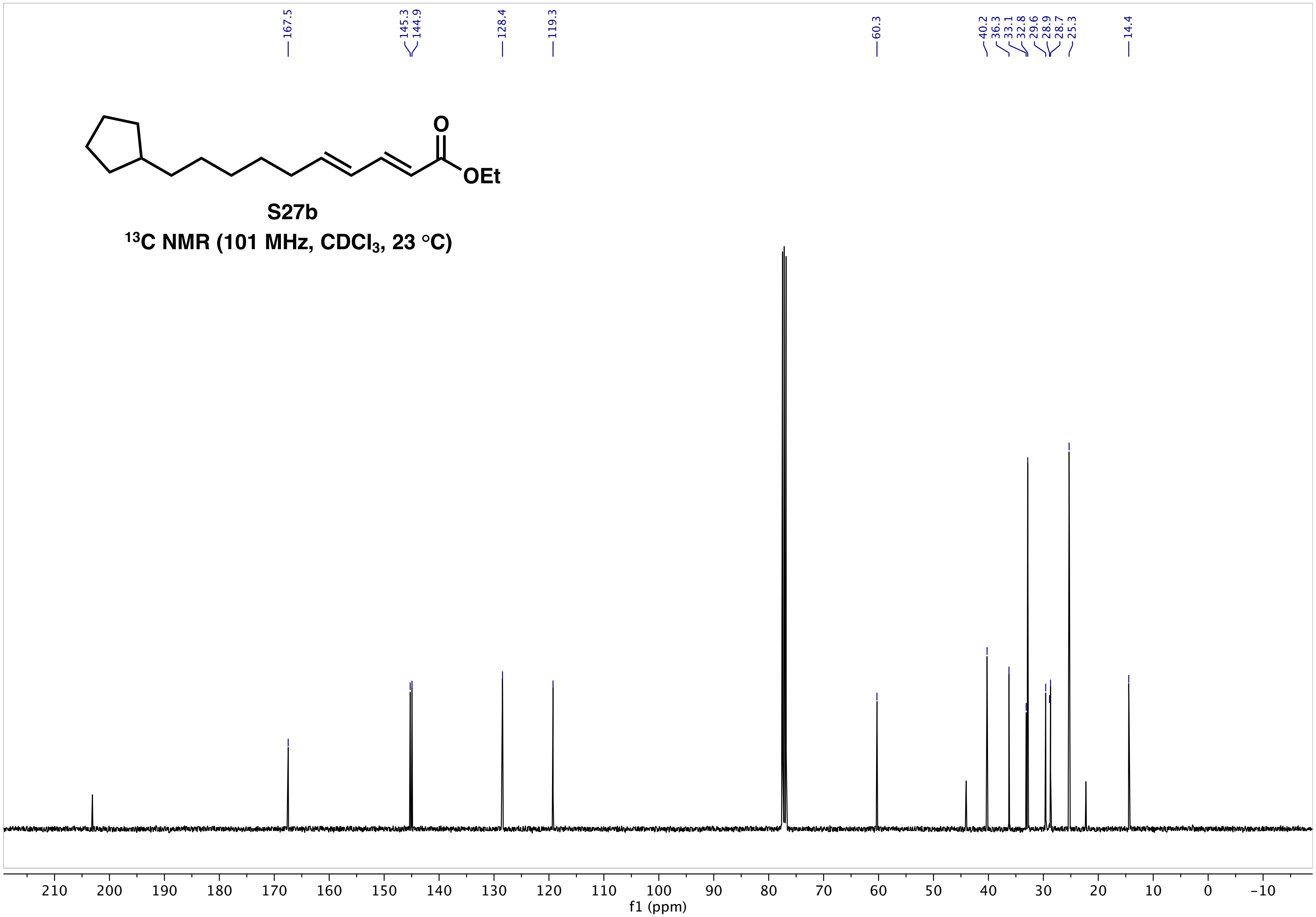

S27b

^{13}C NMR (101 MHz, $CDCl_3$, 23 °C)

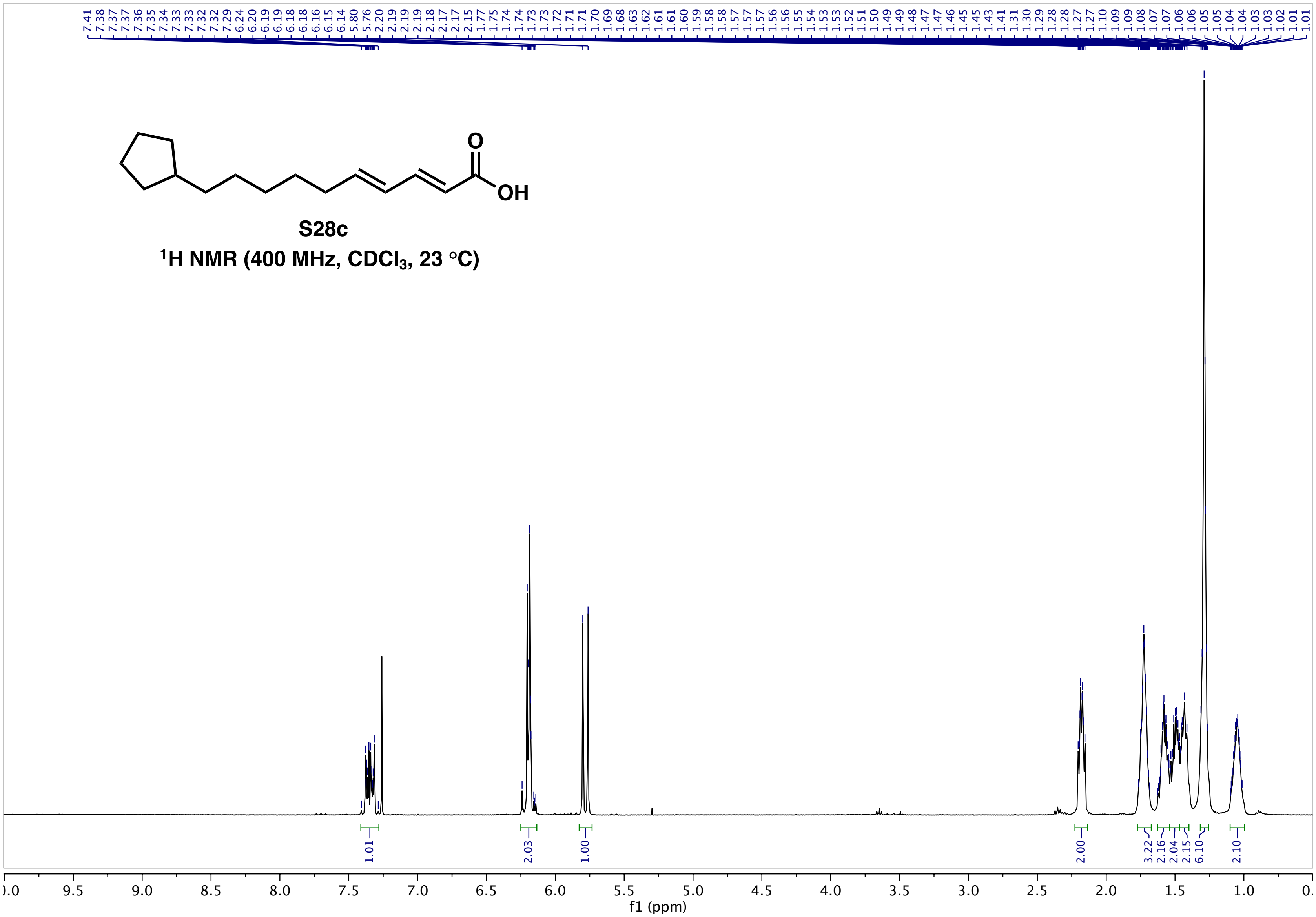
S28c
^{1}H NMR (400 MHz, $CDCl_3$, 23 °C)
f1 (ppm)

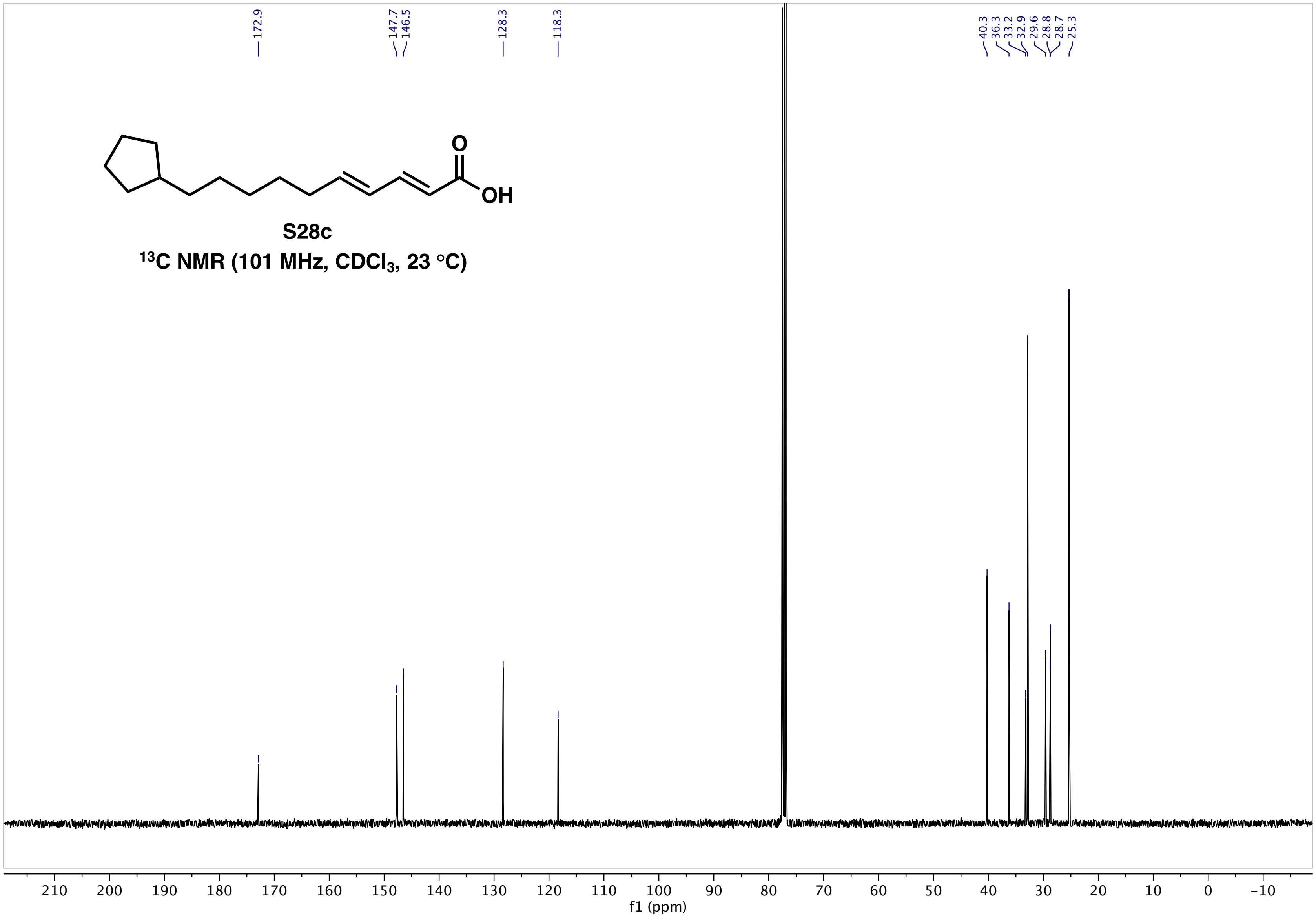

172.9
147.7
146.5
128.3
118.3
40.3
36.3
33.2
32.9
29.6
28.8
28.7
25.3
O
OH
S28c
^{13}C NMR (101 MHz, $CDCl_3$, 23 °C)
210 200 190 180 170 160 150 140 130 120 110 100 90 80 70 60 50 40 30 20 10 0 –10
f1 (ppm)

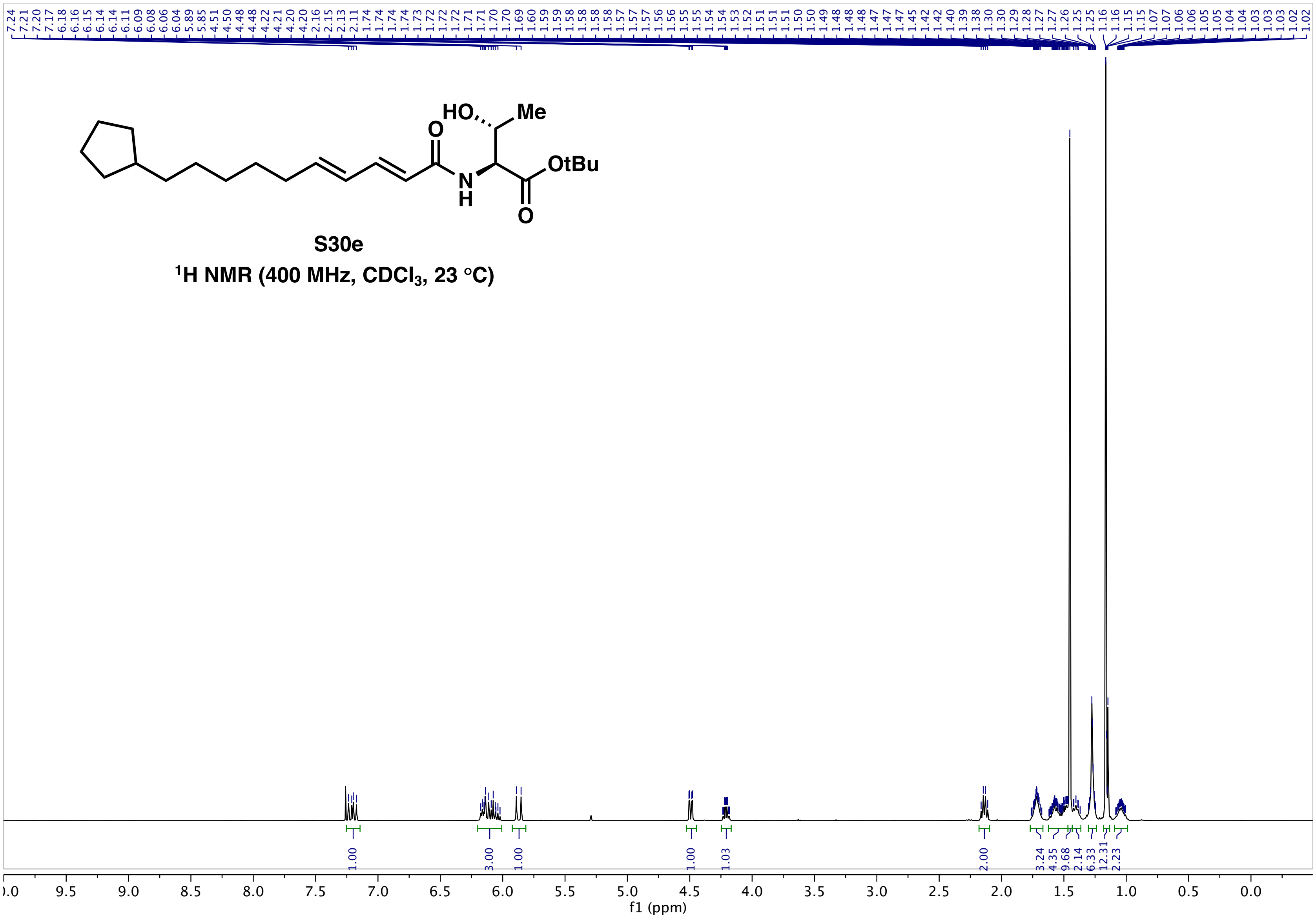
S30e
¹H NMR (400 MHz, CDCl₃, 23 °C)
HO
Me
O
OtBu
N
H
O
f1 (ppm)

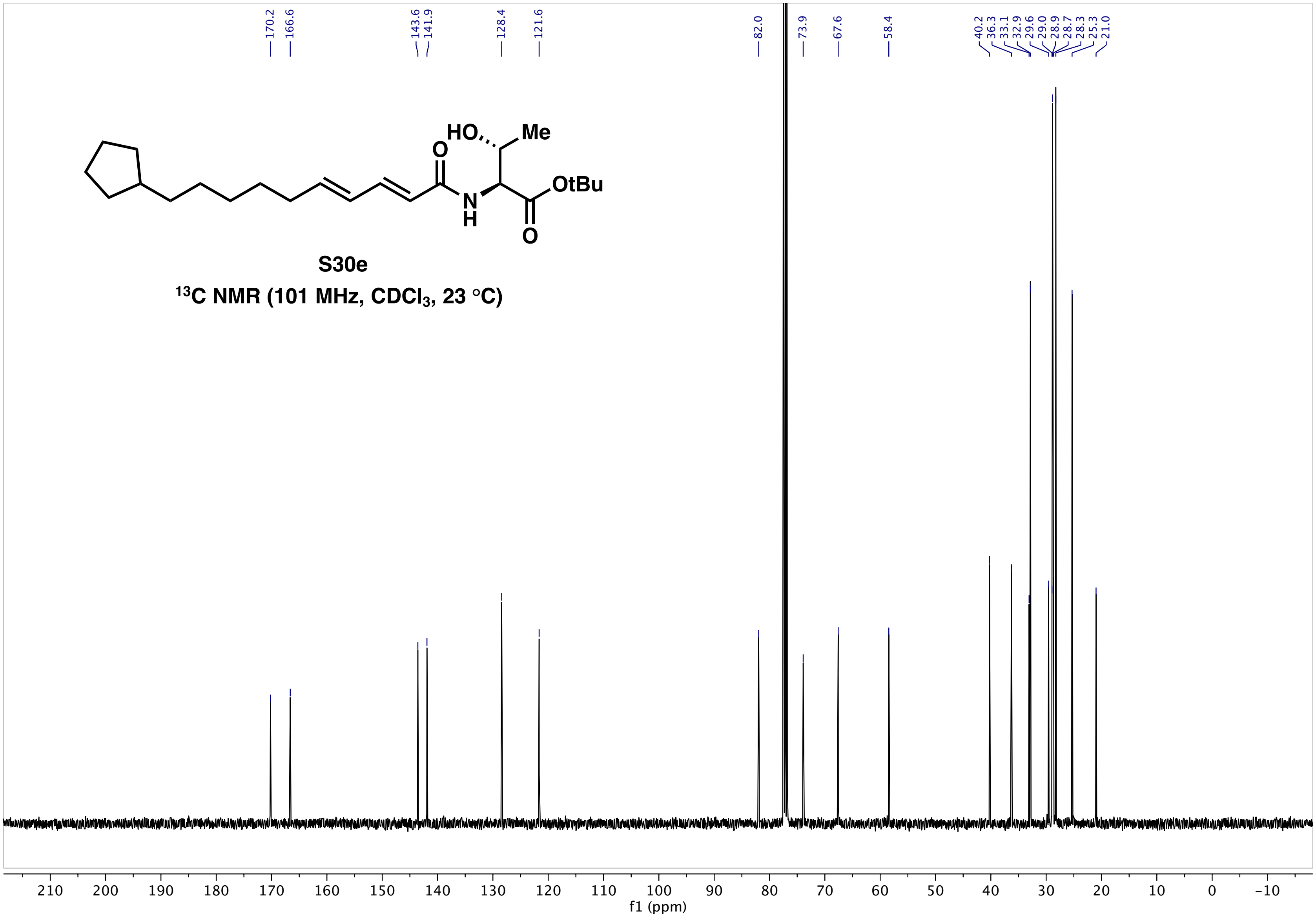
HO
Me
O
OtBu
N
H
O
S30e
^{13}C NMR (101 MHz, CDCl$_3$, 23 °C)
170.2
166.6
143.6
141.9
128.4
121.6
82.0
73.9
67.6
58.4
40.2
36.3
33.1
32.9
29.6
29.0
28.9
28.7
28.3
25.3
21.0
210 200 190 180 170 160 150 140 130 120 110 100 90 80 70 60 50 40 30 20 10 0 -10
f1 (ppm)

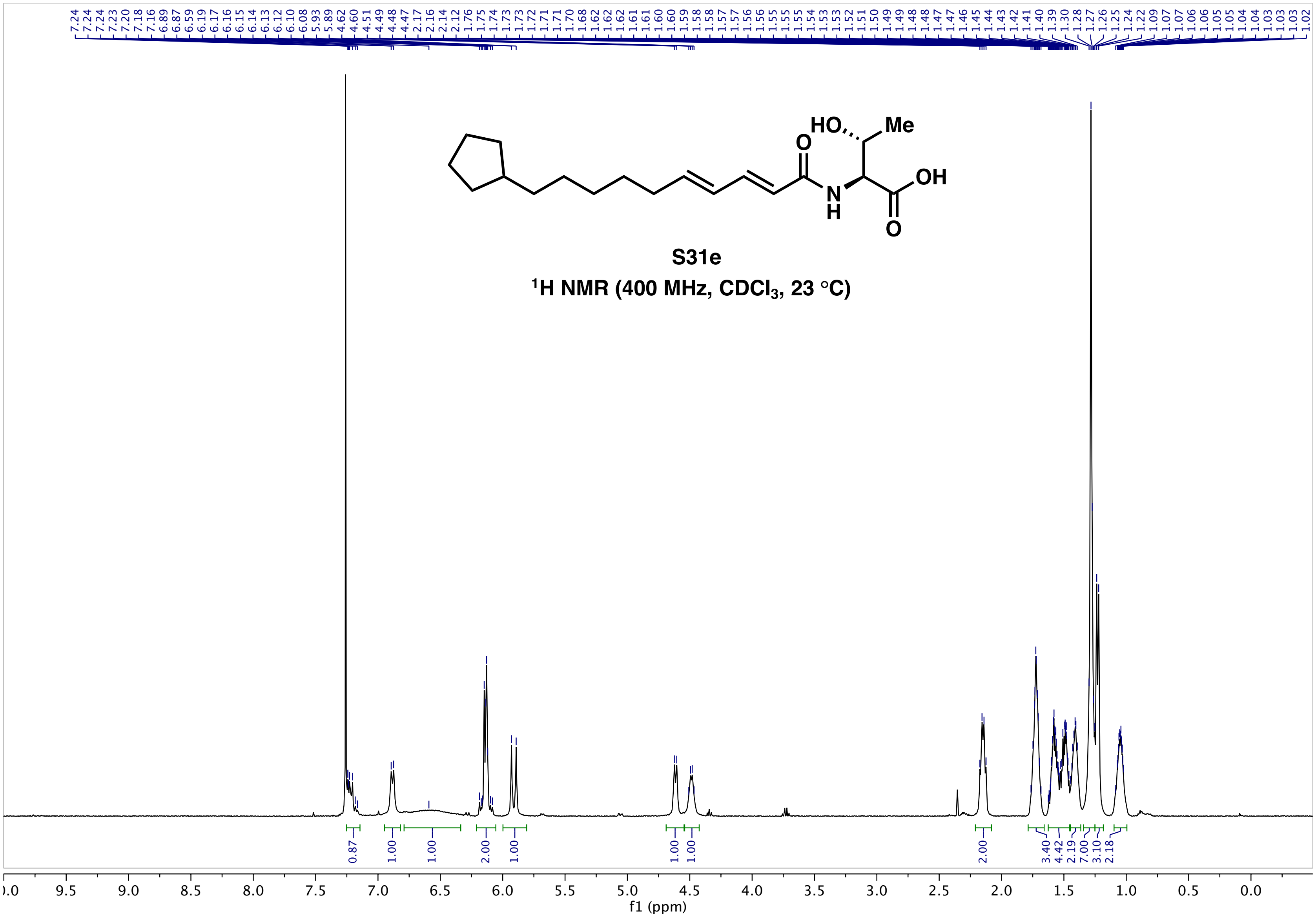

S31e

^{1}H NMR (400 MHz, $CDCl_3$, 23 °C)

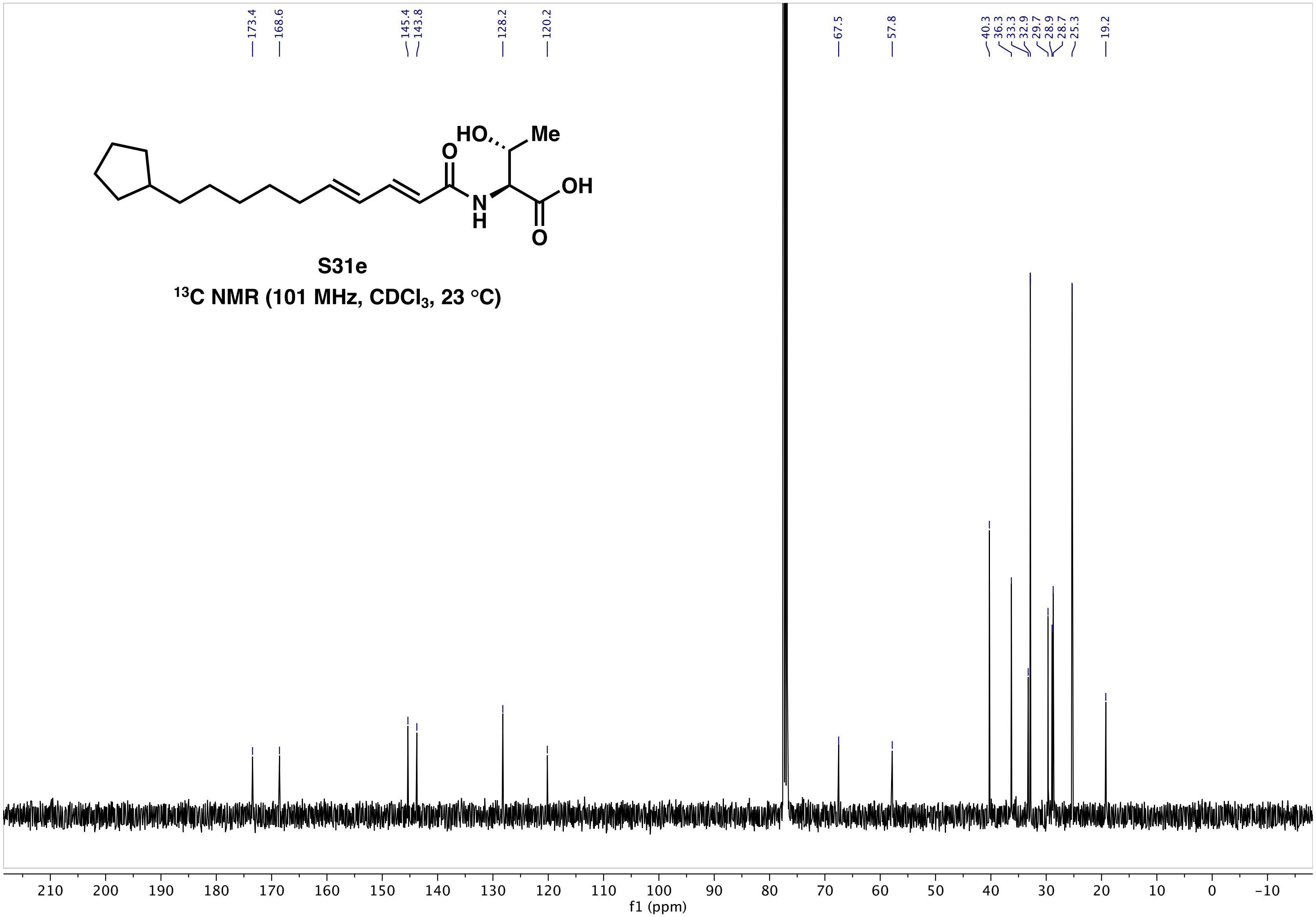

HO
Me
O
OH
N
H
O
S31e
^{13}C NMR (101 MHz, CDCl$_3$, 23 °C)
173.4
168.6
145.4
143.8
128.2
120.2
67.5
57.8
40.3
36.3
33.3
32.9
29.7
28.9
28.7
25.3
19.2
210
200
190
180
170
160
150
140
130
120
110
100
90
80
70
60
50
40
30
20
10
0
–10
f1 (ppm)

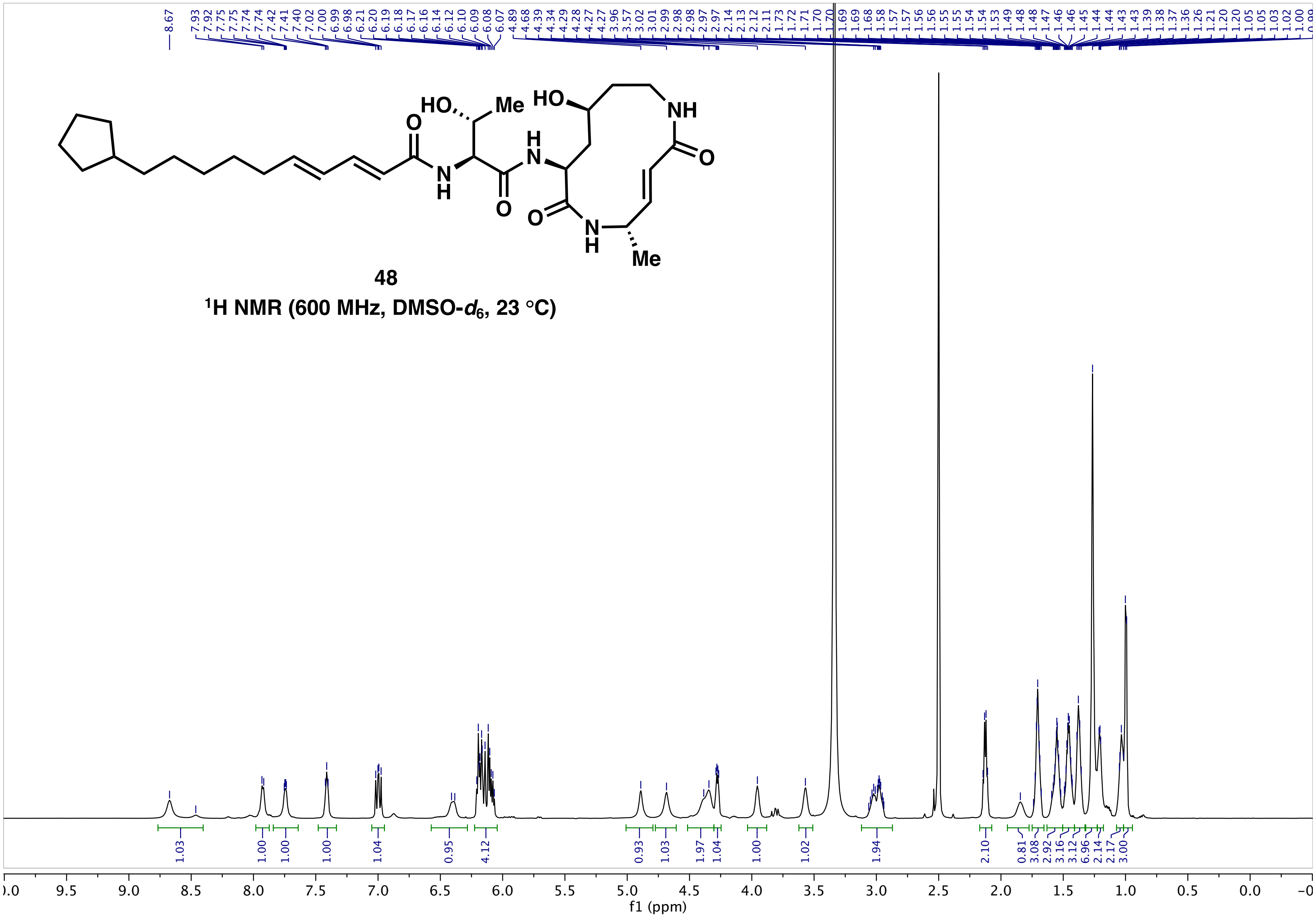

48

^{1}H NMR (600 MHz, DMSO-d_6, 23 °C)

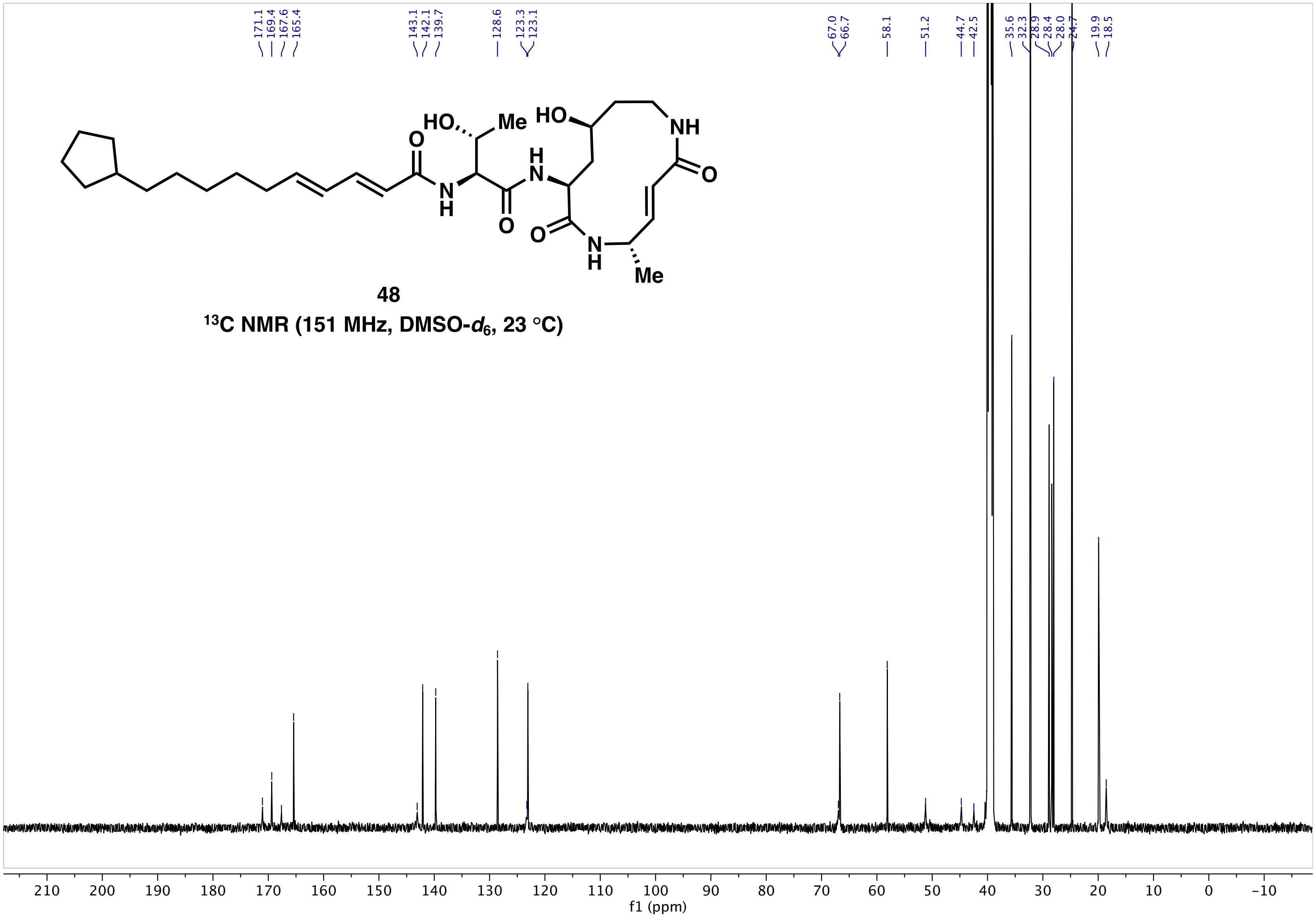

48

^{13}C NMR (151 MHz, DMSO-d_6, 23 °C)

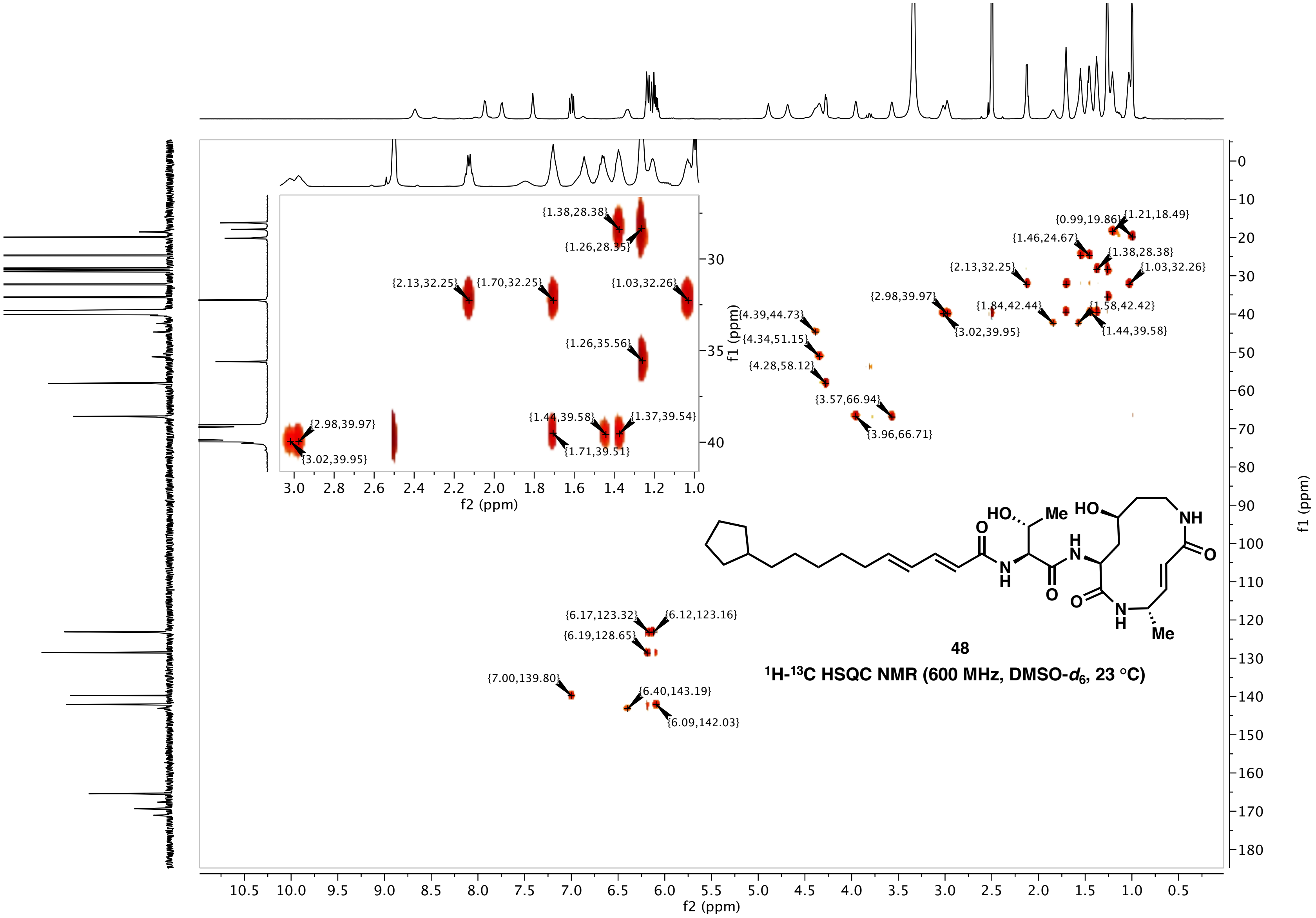

^{1}H-^{13}C HSQC NMR (600 MHz, DMSO-d_6, 23 °C)

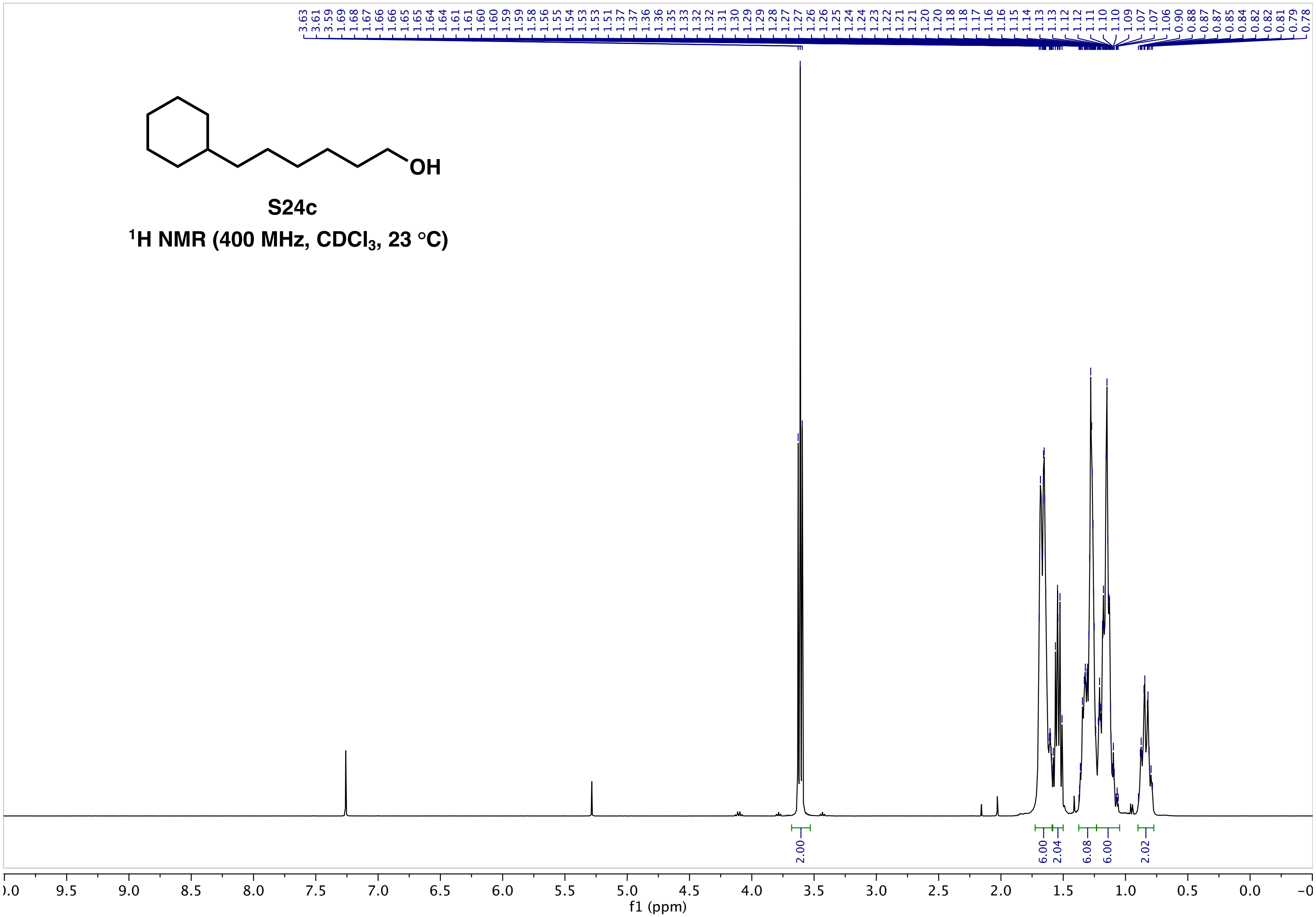

S24c

^1H NMR (400 MHz, $CDCl_3$, 23 °C)

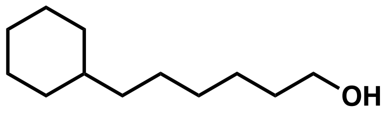

S24c

^{13}C NMR (101 MHz, $CDCl_3$, 23 °C)

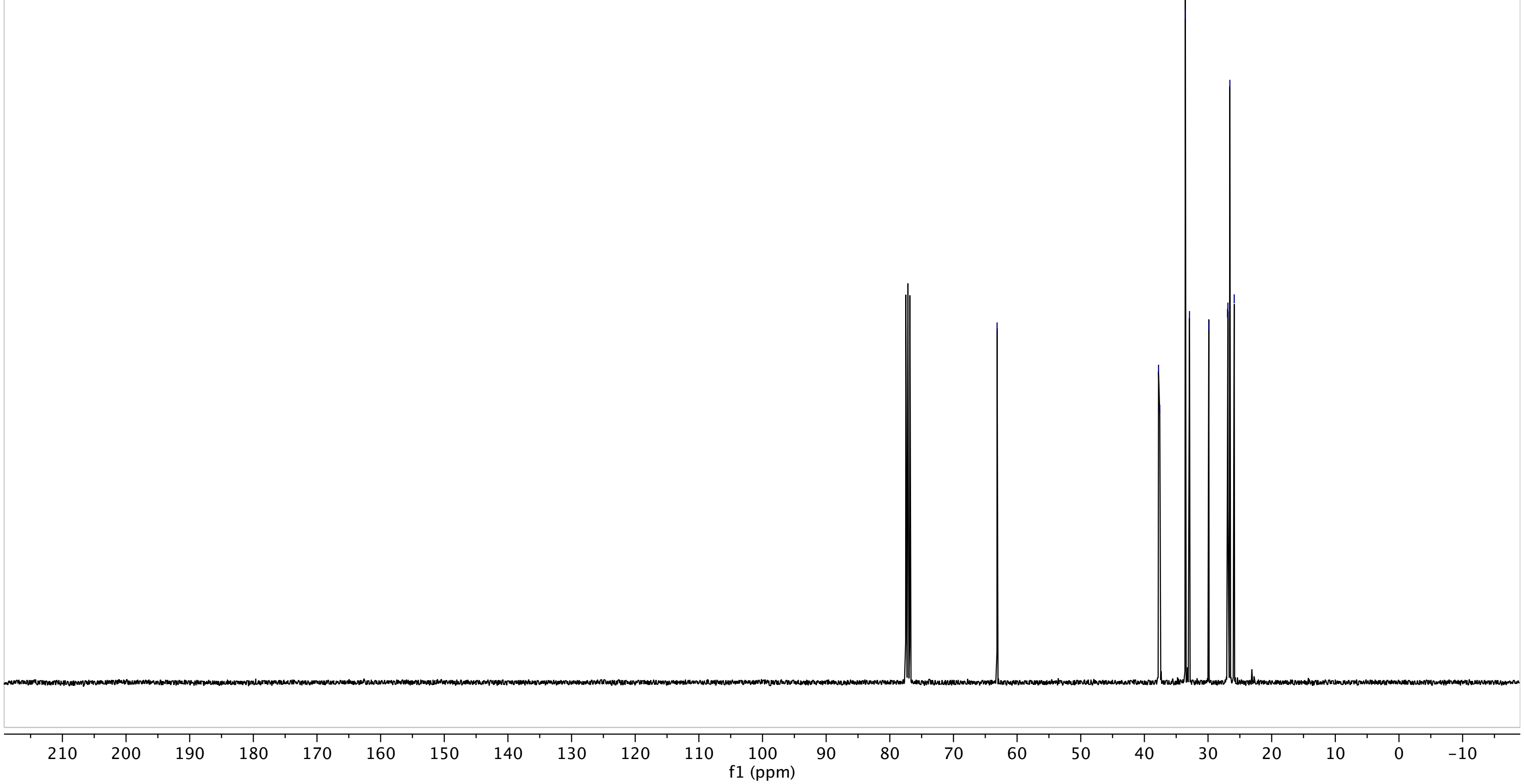

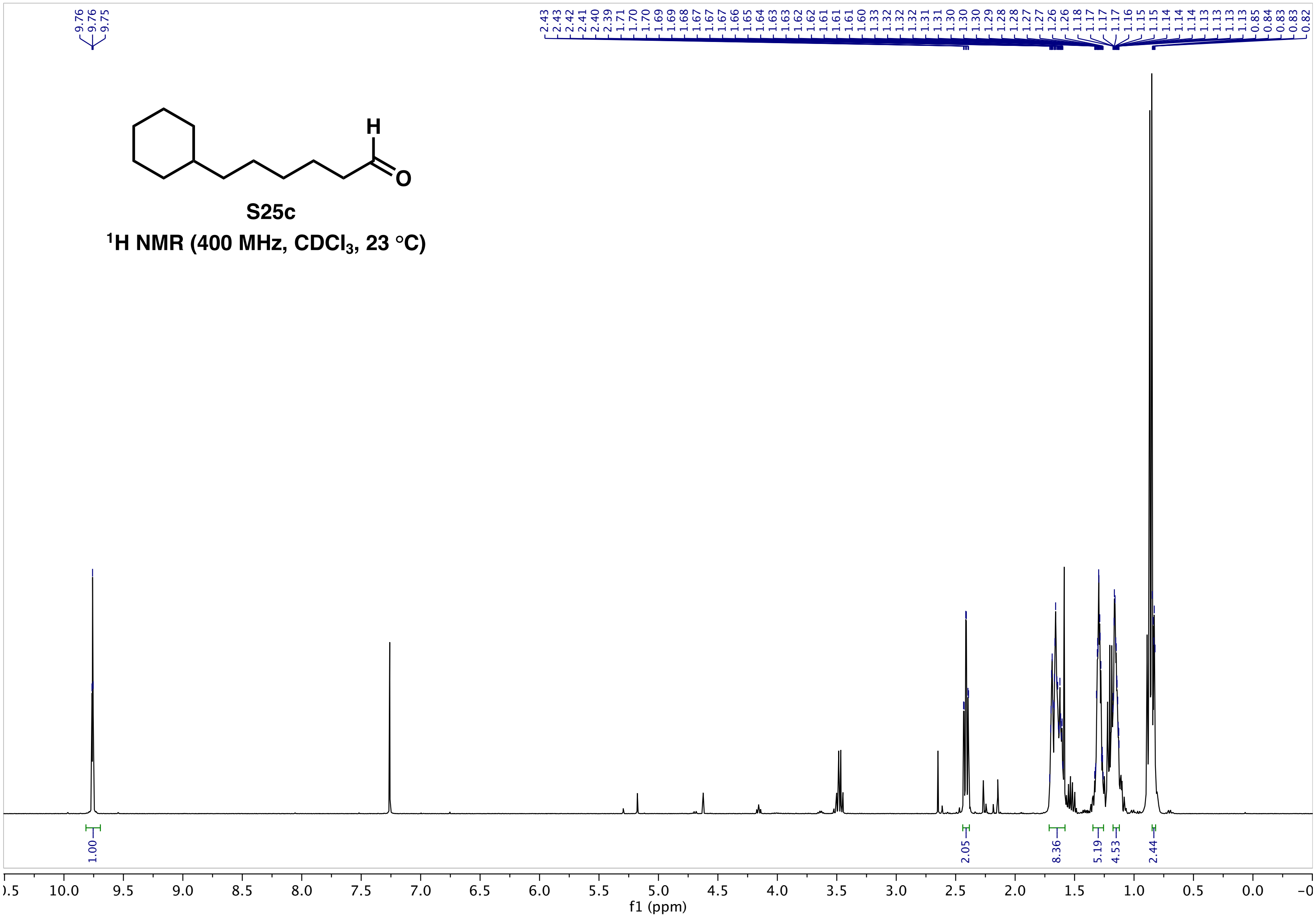

S25c

^{1}H NMR (400 MHz, $CDCl_3$, 23 °C)

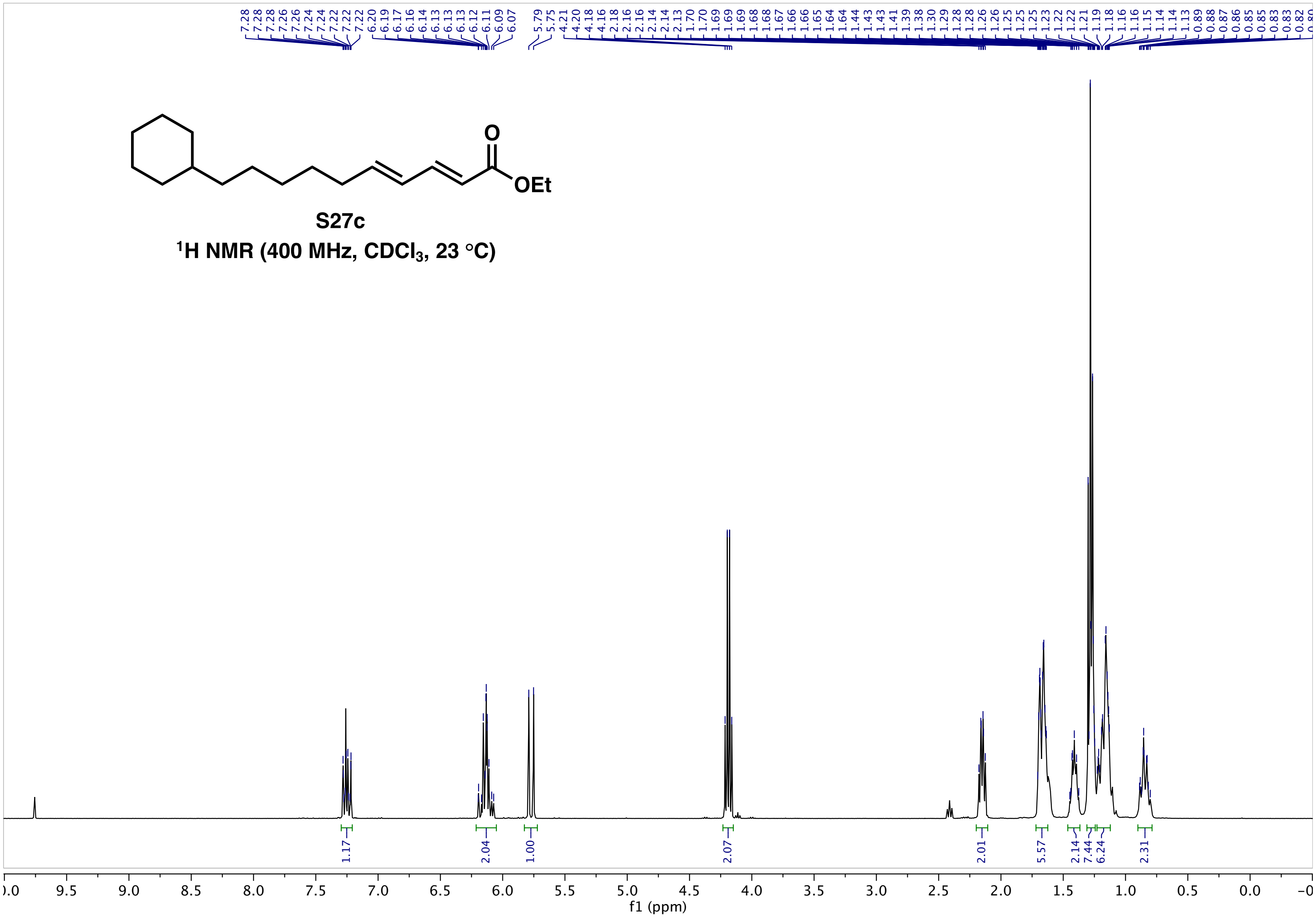
O
OEt
S27c
1H NMR (400 MHz, CDCl3, 23 °C)
f1 (ppm)

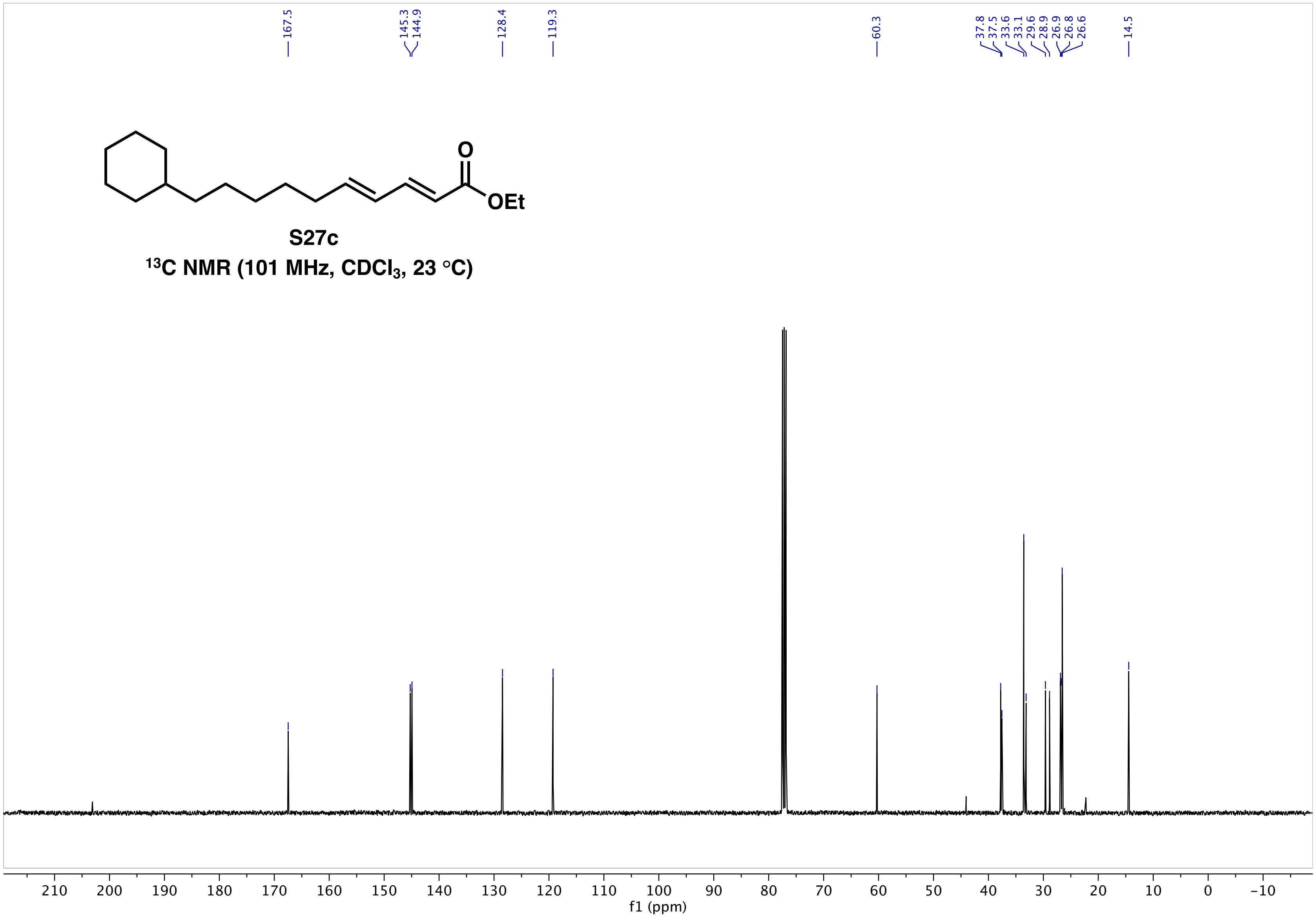
167.5
145.3
144.9
128.4
119.3
60.3
37.8
37.5
33.6
33.1
29.6
28.9
26.9
26.8
26.6
14.5
O
OEt
S27c
^{13}C NMR (101 MHz, $CDCl_3$, 23 °C)
210
200
190
180
170
160
150
140
130
120
110
100
90
80
70
60
50
40
30
20
10
0
−10
f1 (ppm)

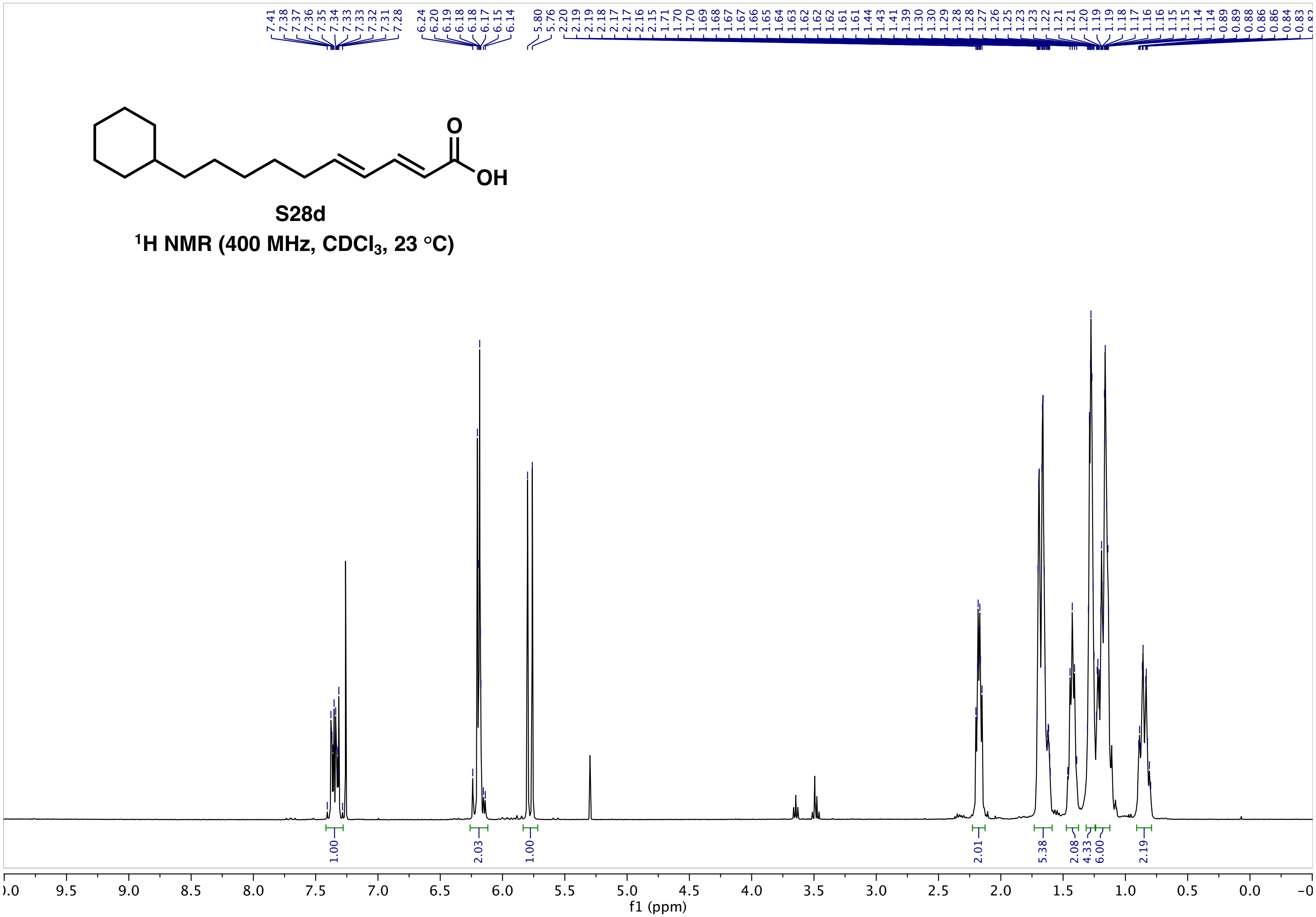

O
OH
S28d
1H NMR (400 MHz, CDCl3, 23 °C)
f1 (ppm)

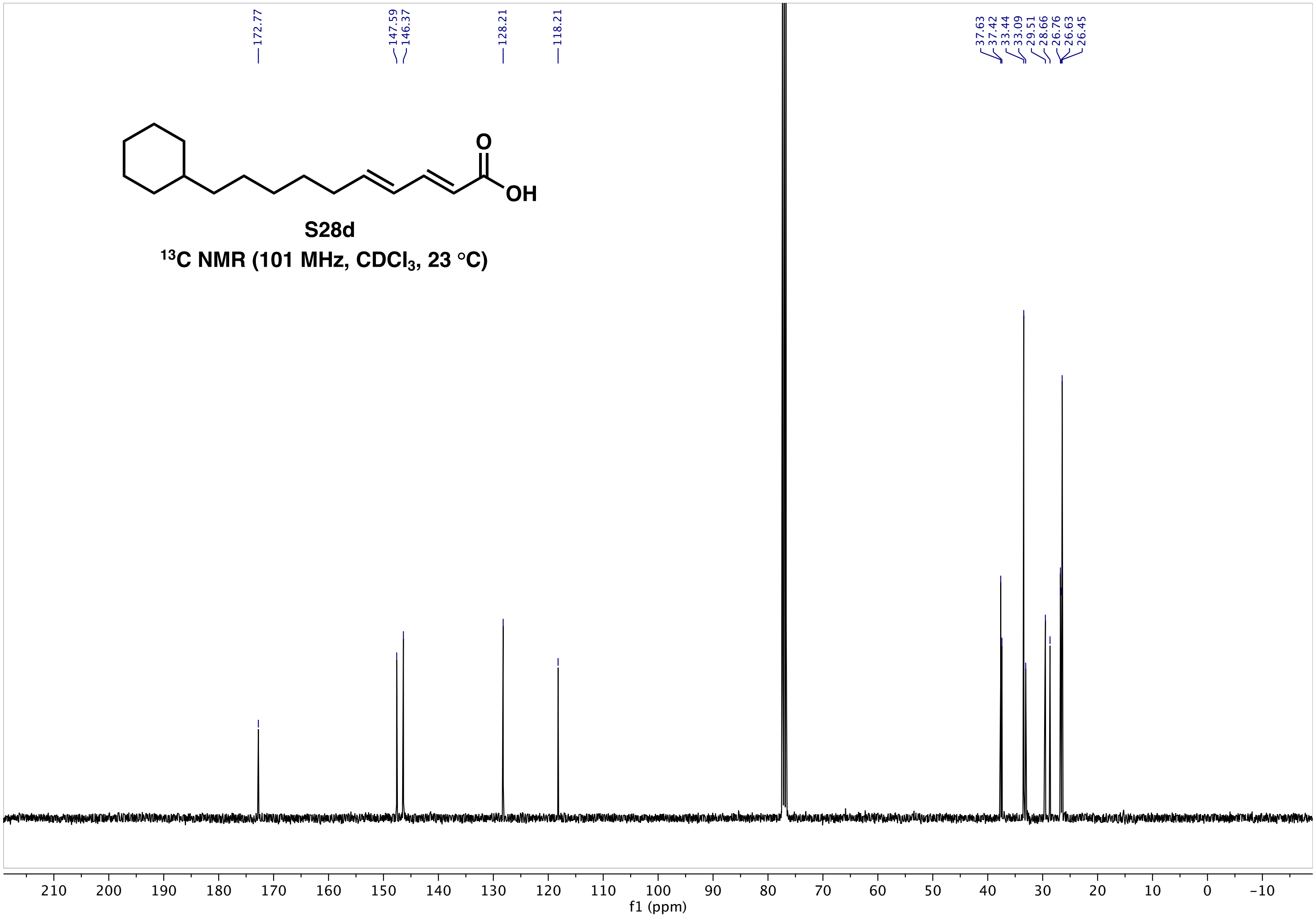
172.77
147.59
146.37
128.21
118.21
37.63
37.42
33.44
33.09
29.51
28.66
26.76
26.63
26.45
O
OH
S28d
^{13}C NMR (101 MHz, CDCl$_3$, 23 °C)
210 200 190 180 170 160 150 140 130 120 110 100 90 80 70 60 50 40 30 20 10 0 −10
f1 (ppm)

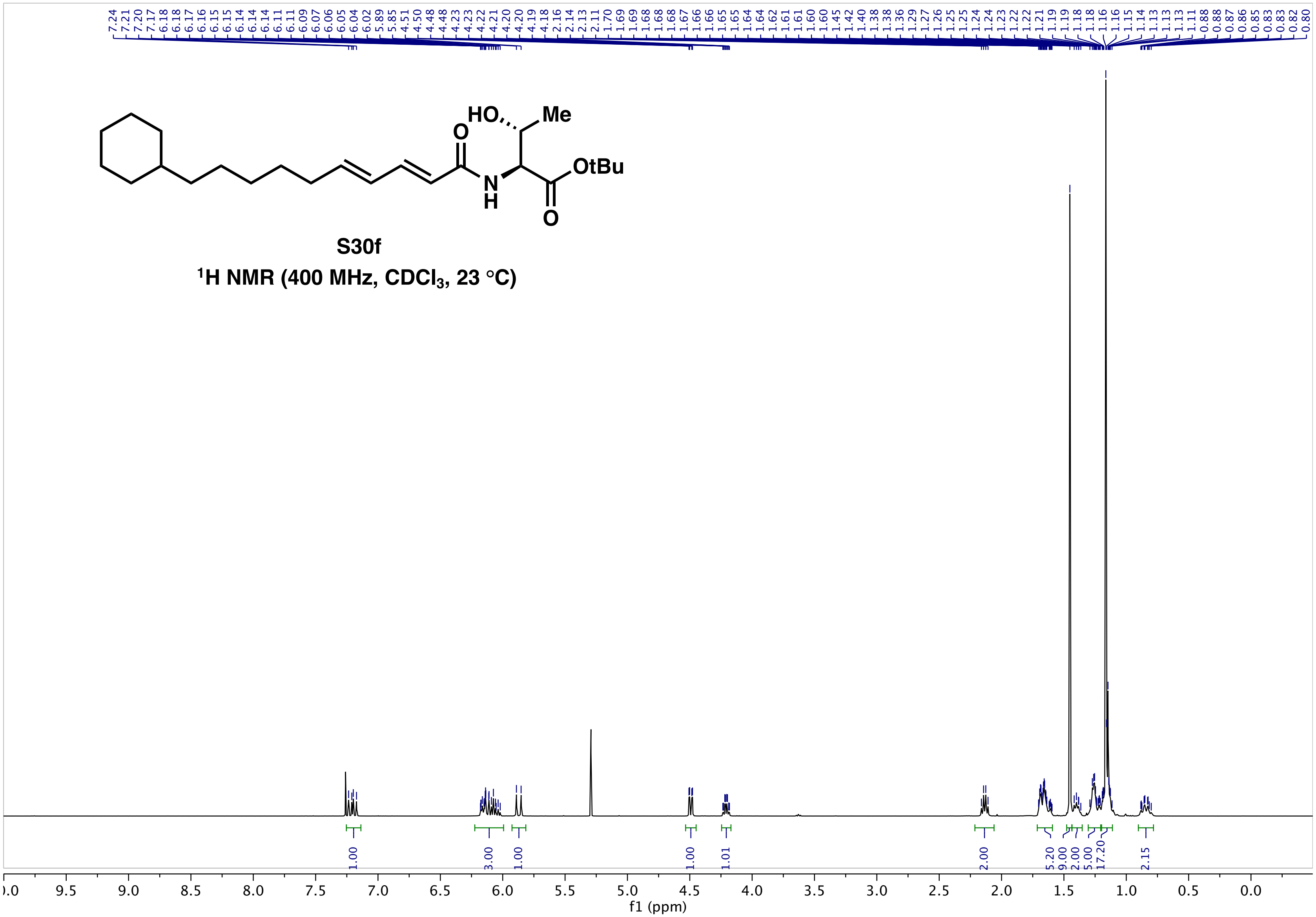

S30f

^{1}H NMR (400 MHz, $CDCl_3$, 23 °C)

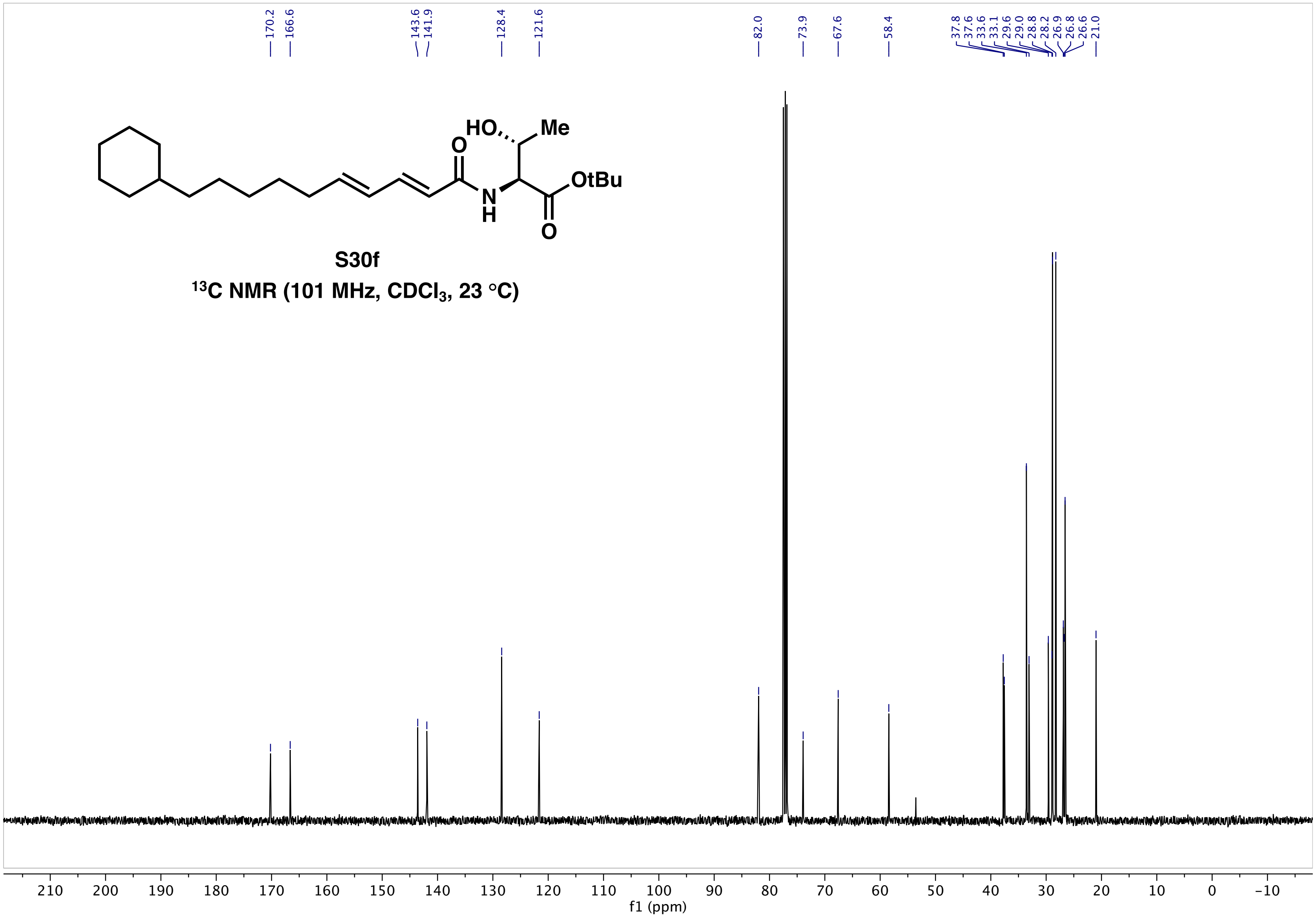

HO
Me
O
OtBu
N
H
O
S30f
13C NMR (101 MHz, CDCl3, 23 °C)
170.2
166.6
143.6
141.9
128.4
121.6
82.0
73.9
67.6
58.4
37.8
37.6
33.6
33.1
29.6
29.0
28.8
28.2
26.9
26.8
26.6
21.0
210
200
190
180
170
160
150
140
130
120
110
100
90
80
70
60
50
40
30
20
10
0
-10
f1 (ppm)

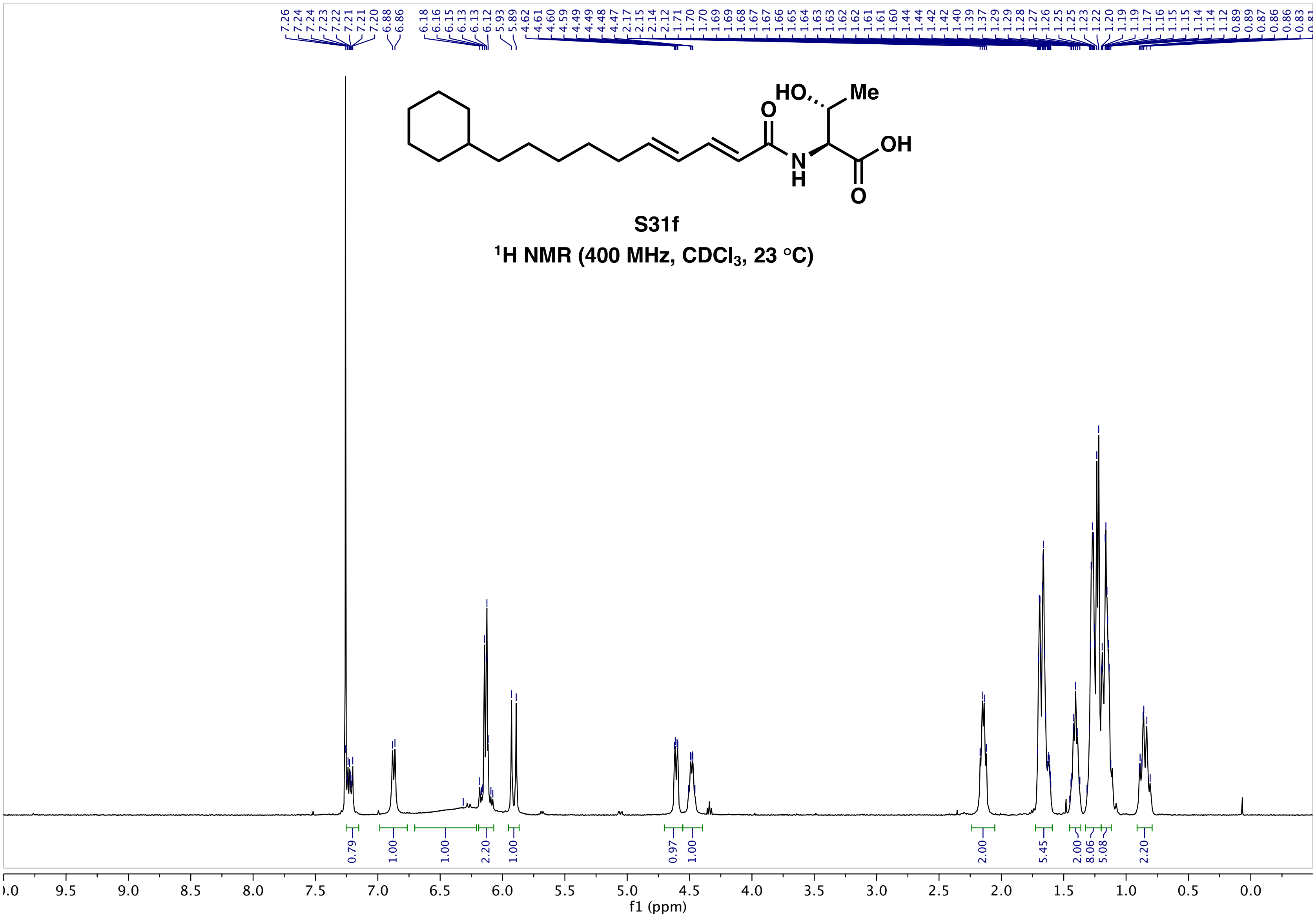
HO
Me
O
OH
N
H
O
S31f
¹H NMR (400 MHz, CDCl₃, 23 °C)
f1 (ppm)

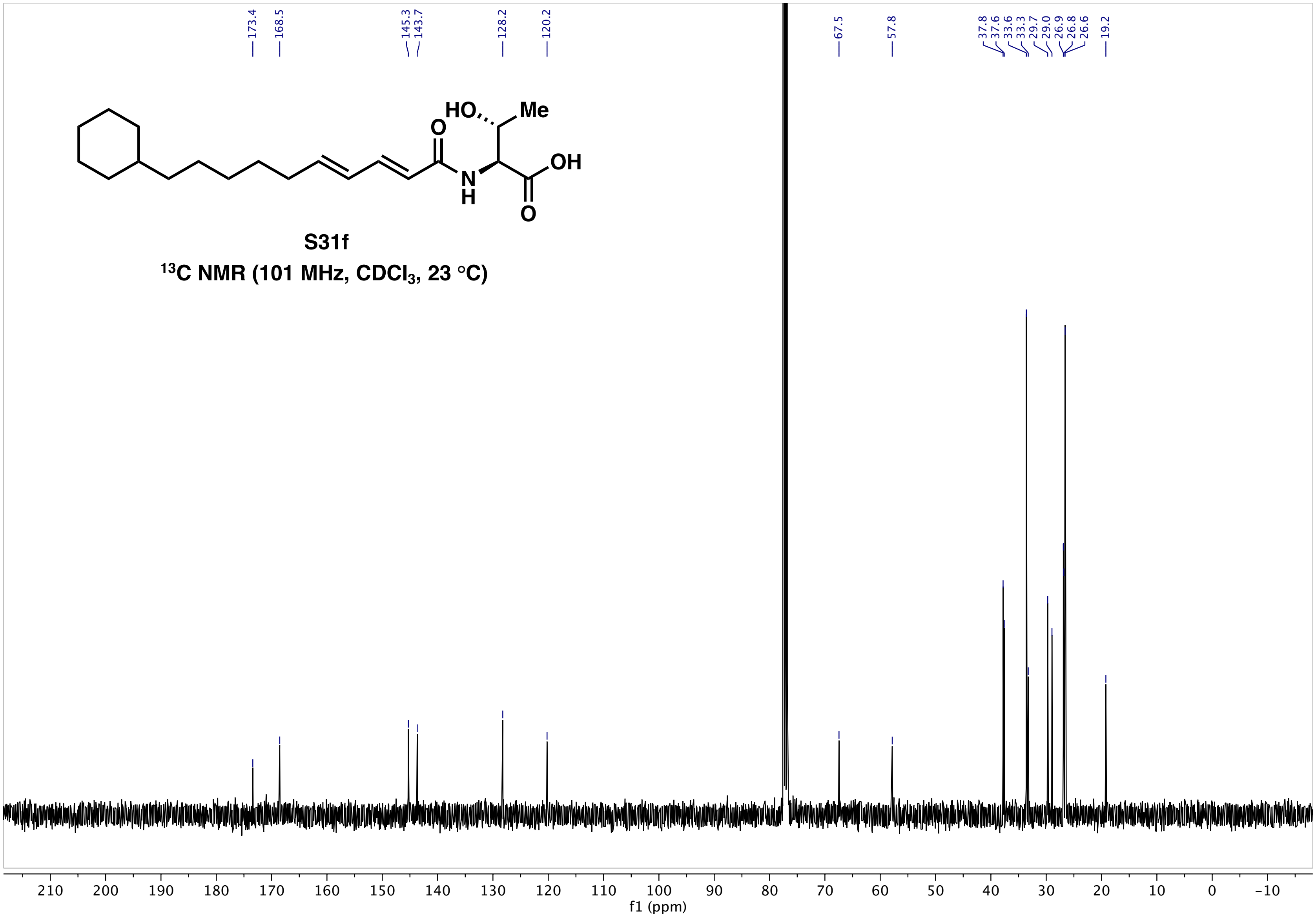
173.4
168.5
145.3
143.7
128.2
120.2
67.5
57.8
37.8
37.6
33.6
33.3
29.7
29.0
26.9
26.8
26.6
19.2
HO
Me
O
OH
N
H
O
S31f
^{13}C NMR (101 MHz, $CDCl_3$, 23 °C)
210 200 190 180 170 160 150 140 130 120 110 100 90 80 70 60 50 40 30 20 10 0 −10
f1 (ppm)

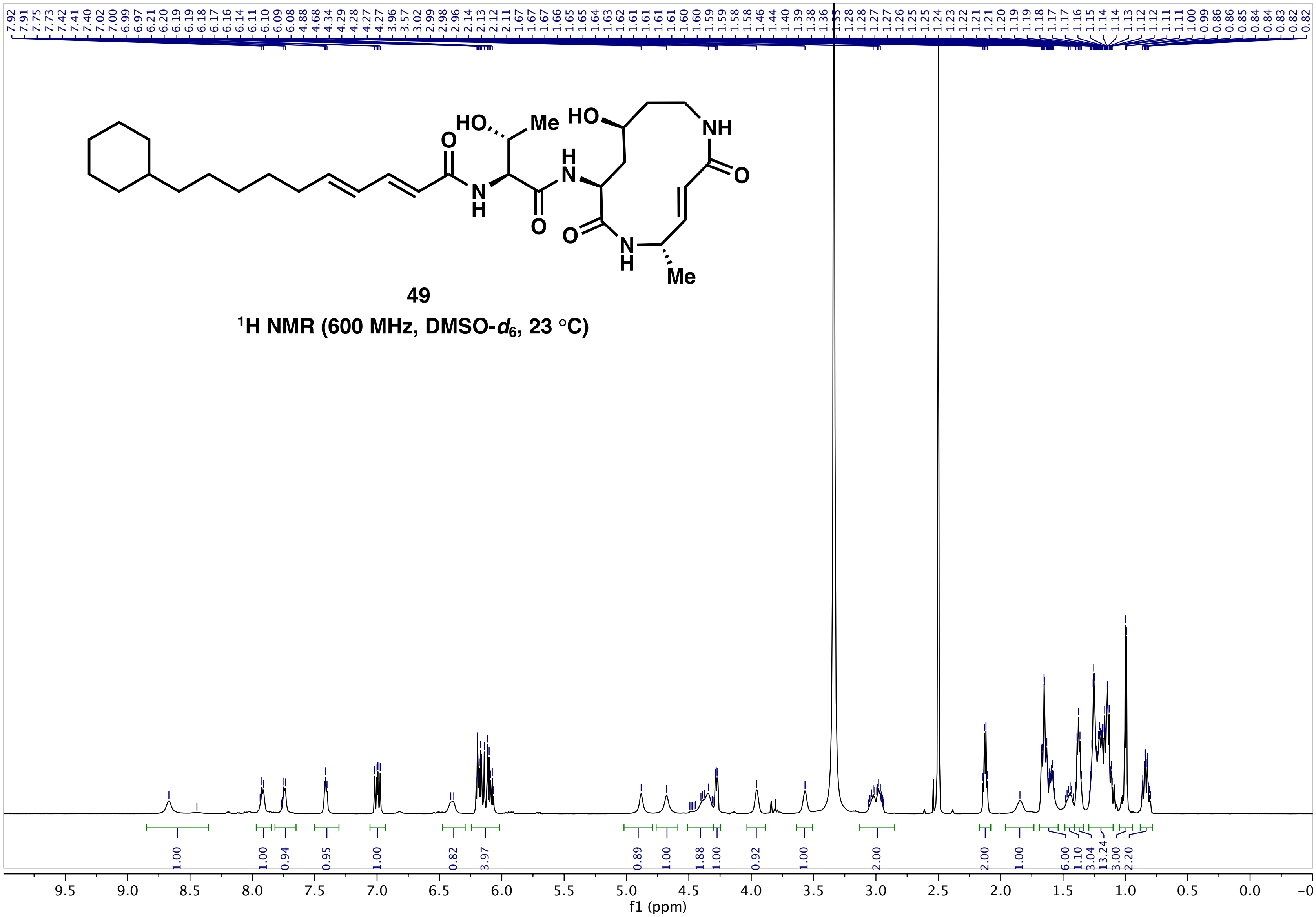

49

^{1}H NMR (600 MHz, DMSO-d_6, 23 °C)

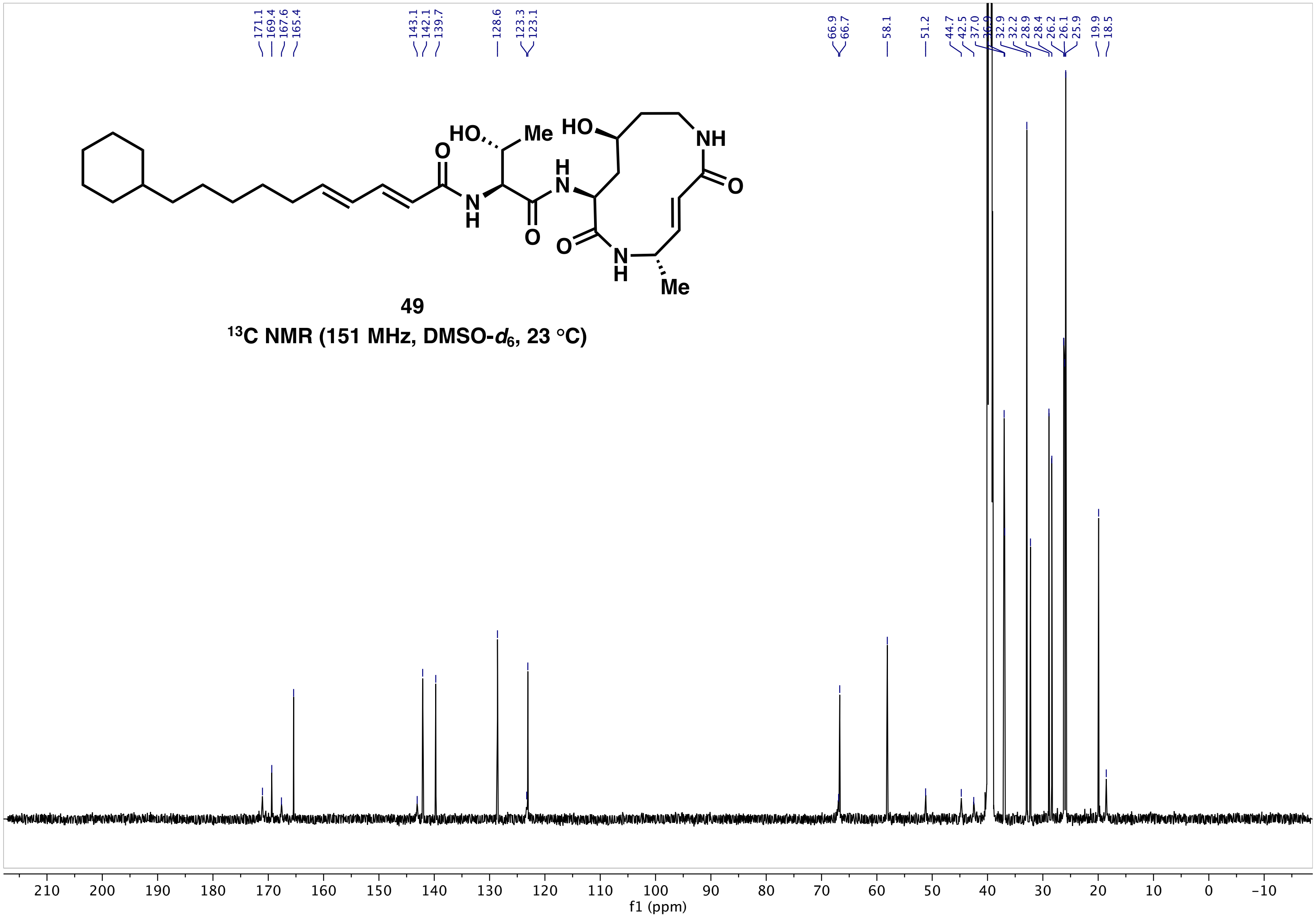

171.1
169.4
167.6
165.4
143.1
142.1
139.7
128.6
123.3
123.1
66.9
66.7
58.1
51.2
44.7
42.5
37.0
32.9
32.2
28.9
28.4
26.2
26.1
25.9
19.9
18.5
HO
Me
HO
NH
O
H
N
N
H
O
O
N
H
O
Me
49
^{13}C NMR (151 MHz, DMSO-d_6, 23 °C)
210 200 190 180 170 160 150 140 130 120 110 100 90 80 70 60 50 40 30 20 10 0 -10
f1 (ppm)

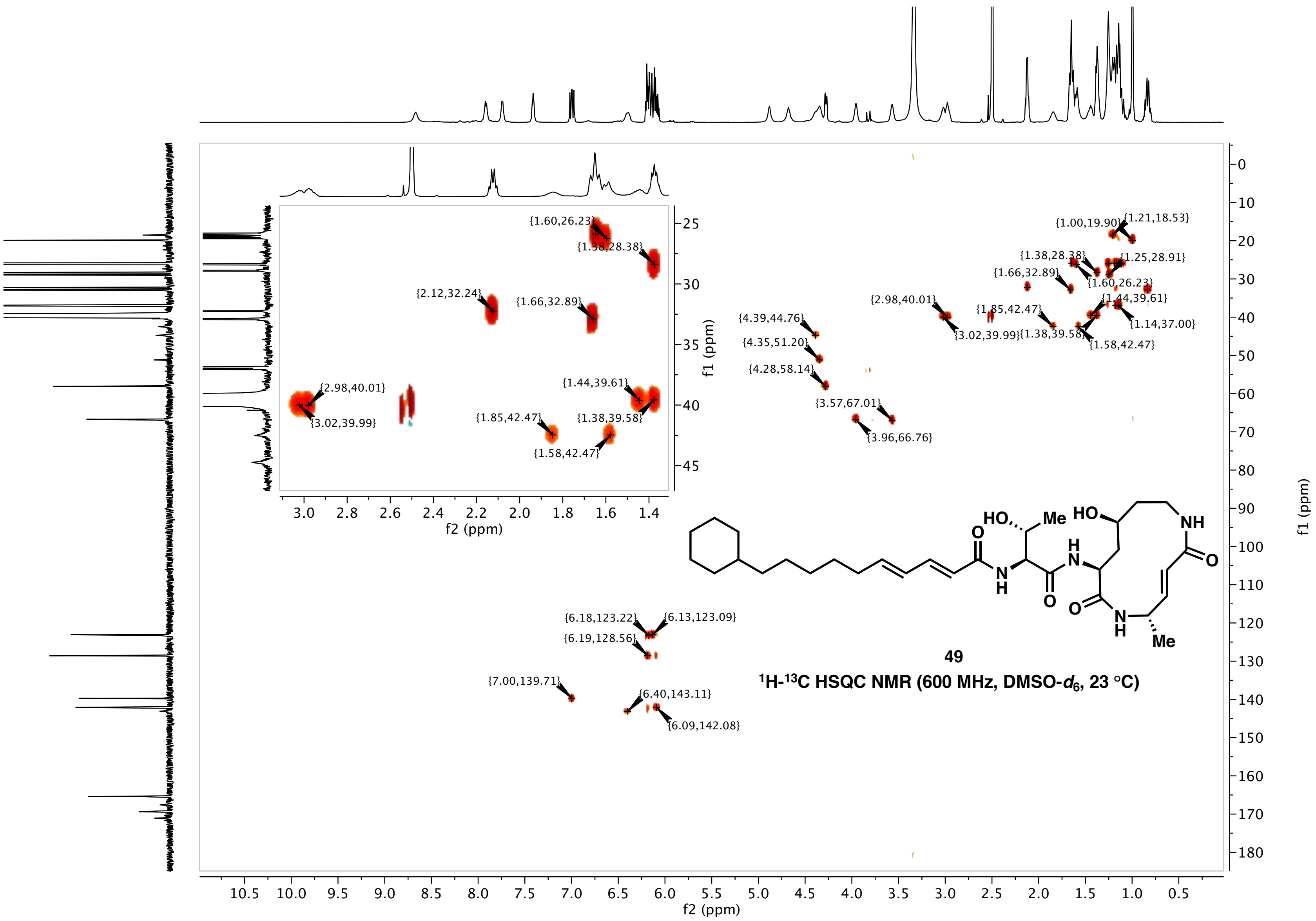

49

^1H-^{13}C HSQC NMR (600 MHz, DMSO-d_6, 23 °C)

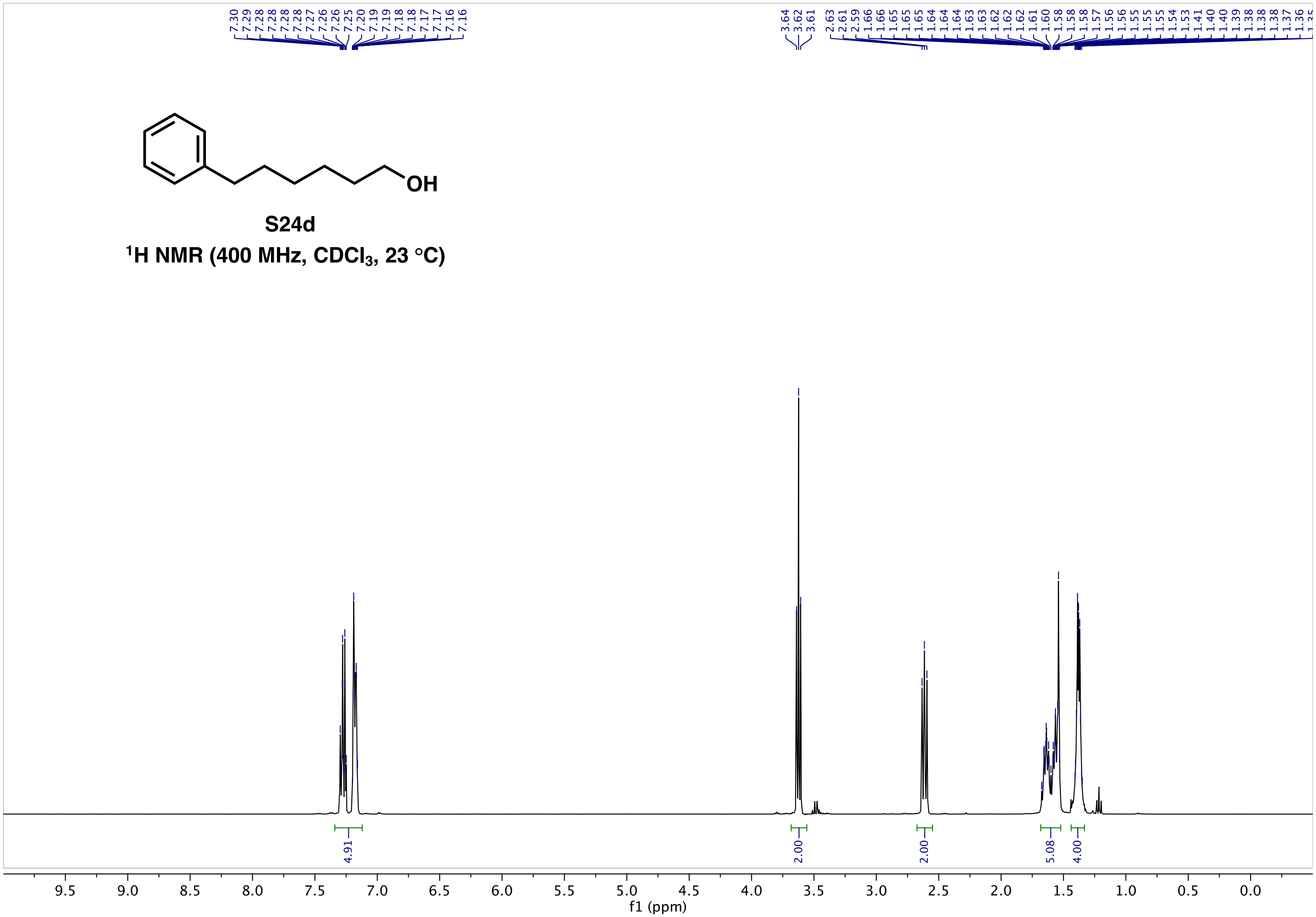

S24d

^{1}H NMR (400 MHz, $CDCl_3$, 23 °C)

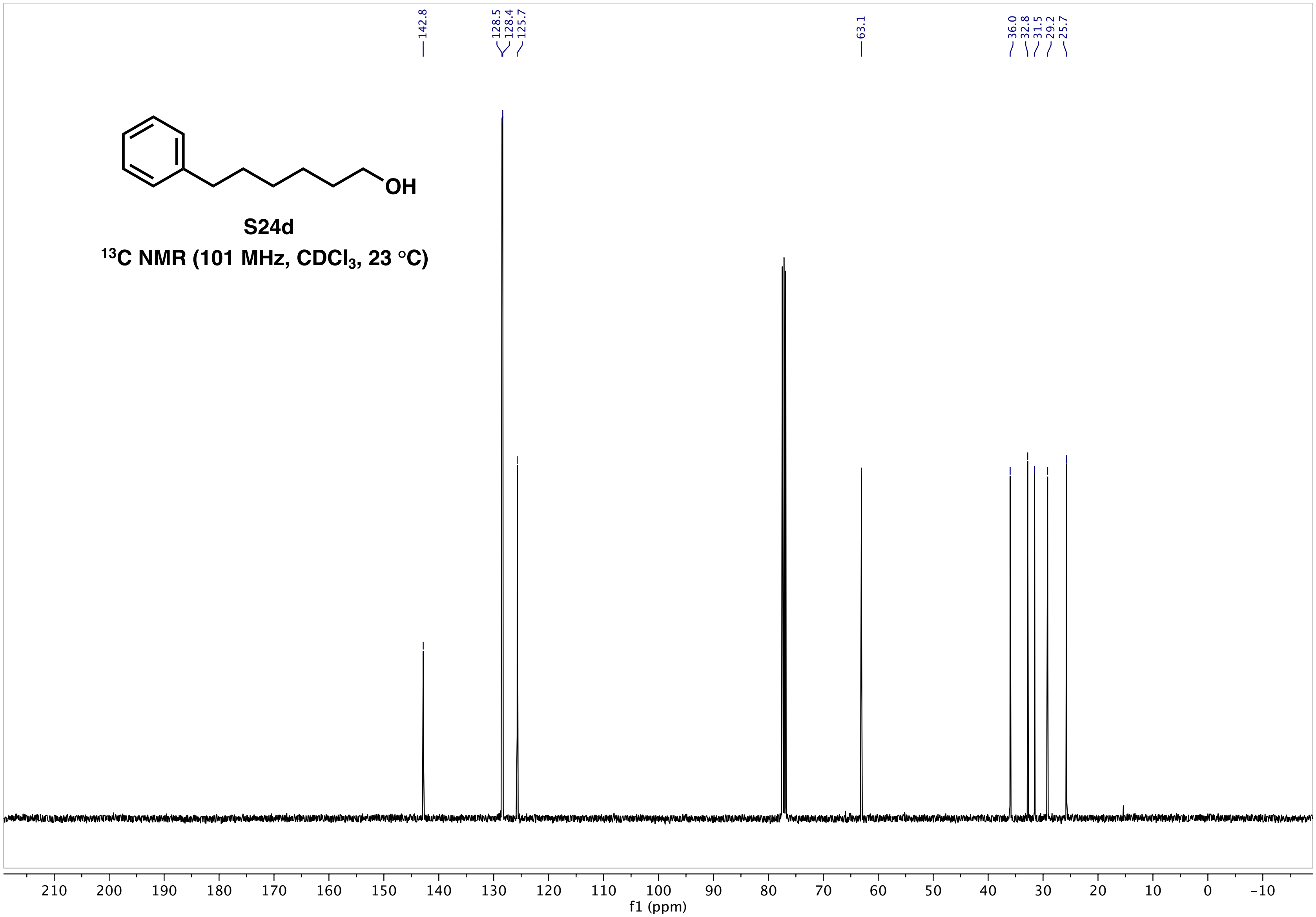

142.8
128.5
128.4
125.7
63.1
36.0
32.8
31.5
29.2
25.7
OH
S24d
13C NMR (101 MHz, CDCl3, 23 °C)
210 200 190 180 170 160 150 140 130 120 110 100 90 80 70 60 50 40 30 20 10 0 -10
f1 (ppm)

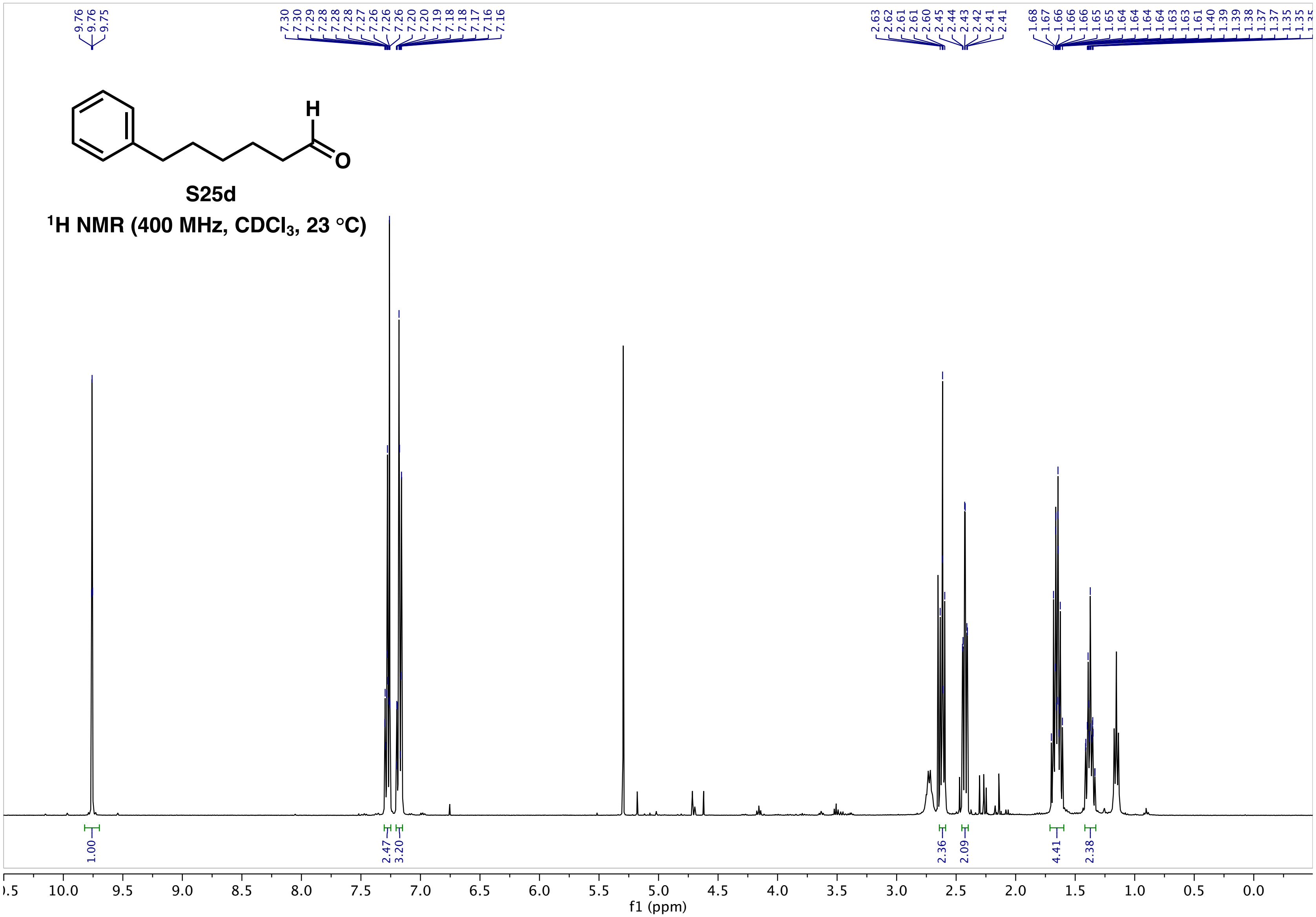
S25d
1H NMR (400 MHz, CDCl3, 23 °C)
f1 (ppm)

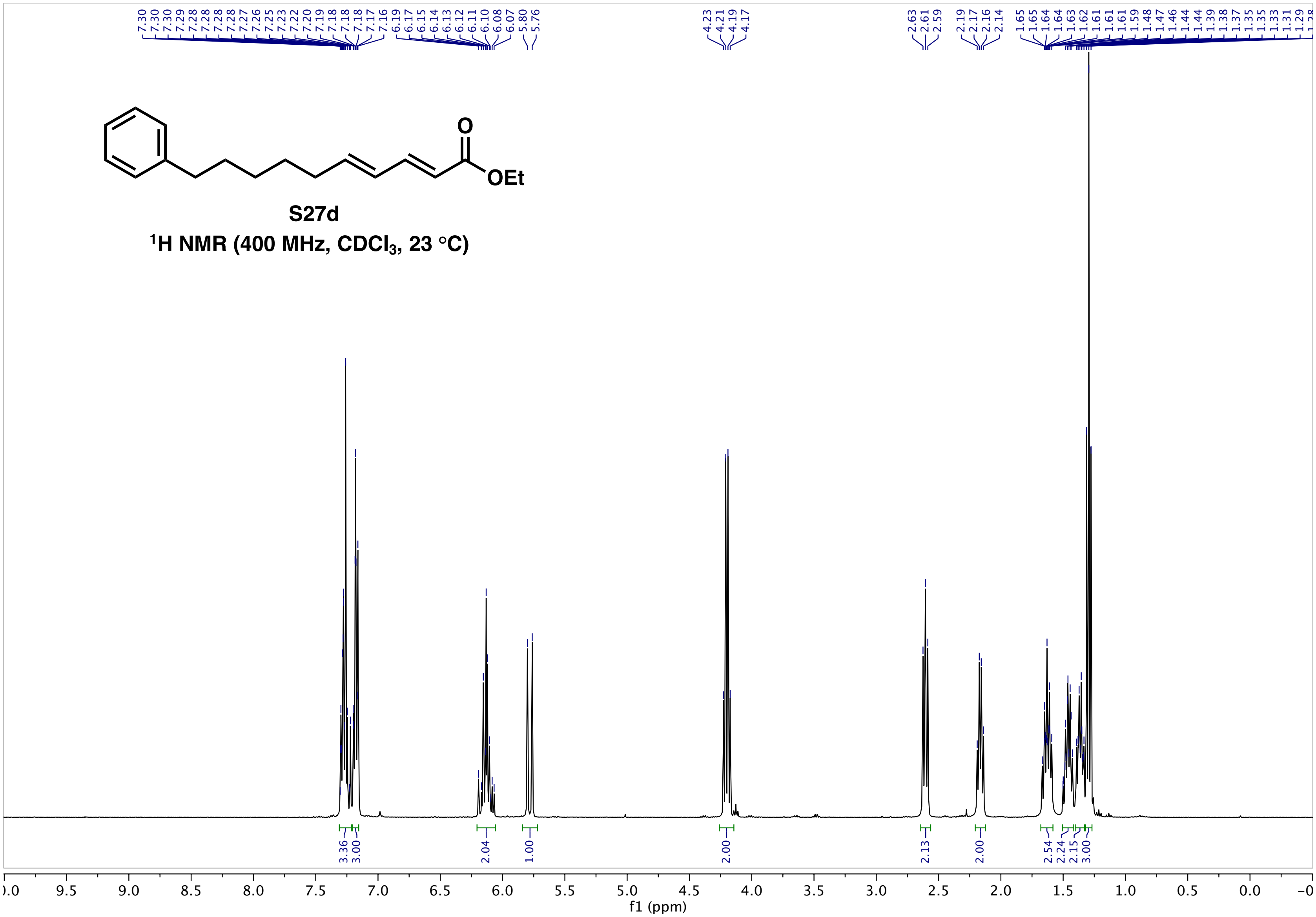
O
OEt
S27d
1H NMR (400 MHz, CDCl3, 23 °C)
f1 (ppm)

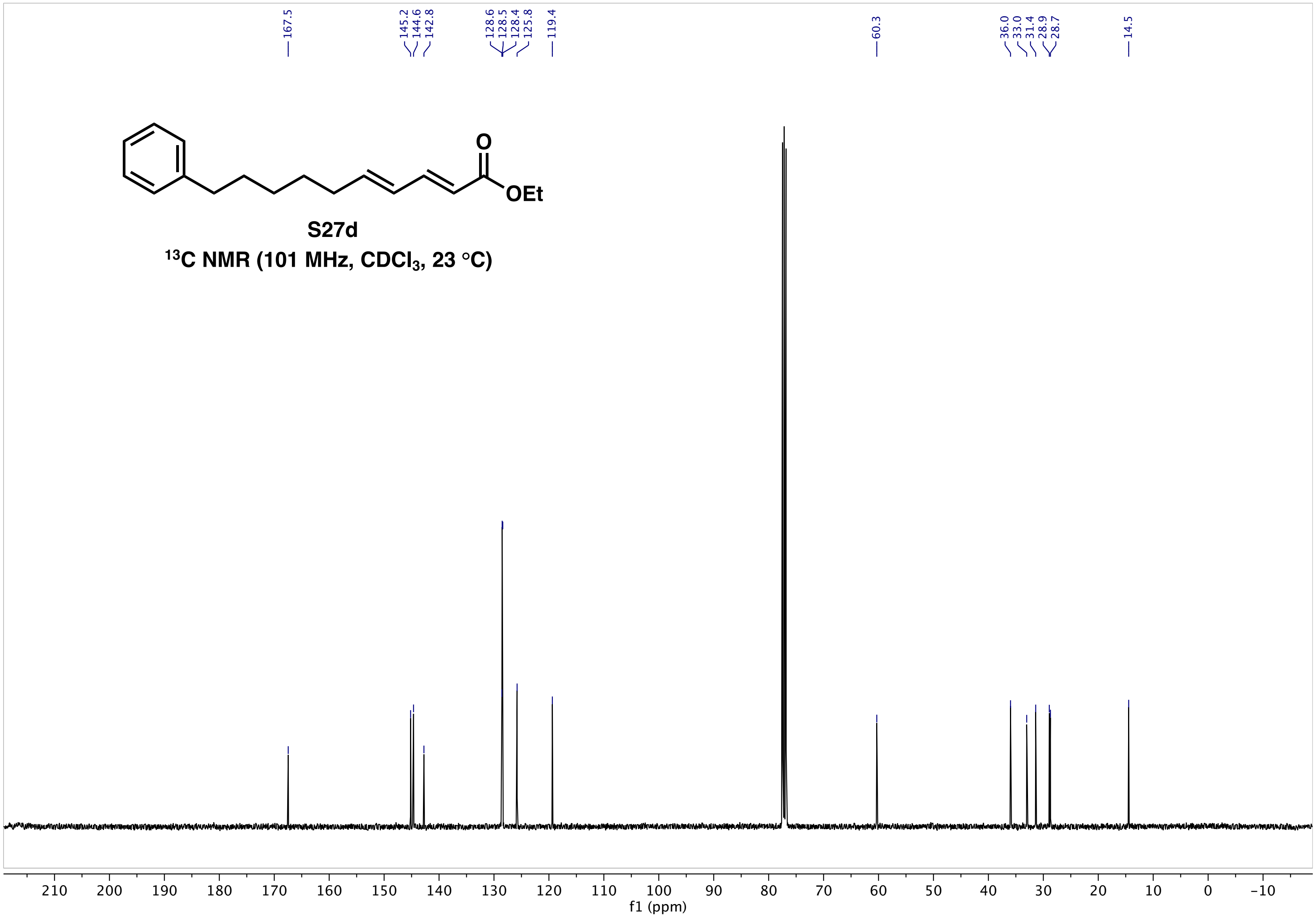
167.5
145.2
144.6
142.8
128.6
128.5
128.4
125.8
119.4
60.3
36.0
33.0
31.4
28.9
28.7
14.5
O
OEt
S27d
13C NMR (101 MHz, CDCl3, 23 °C)
210
200
190
180
170
160
150
140
130
120
110
100
90
80
70
60
50
40
30
20
10
0
-10
f1 (ppm)

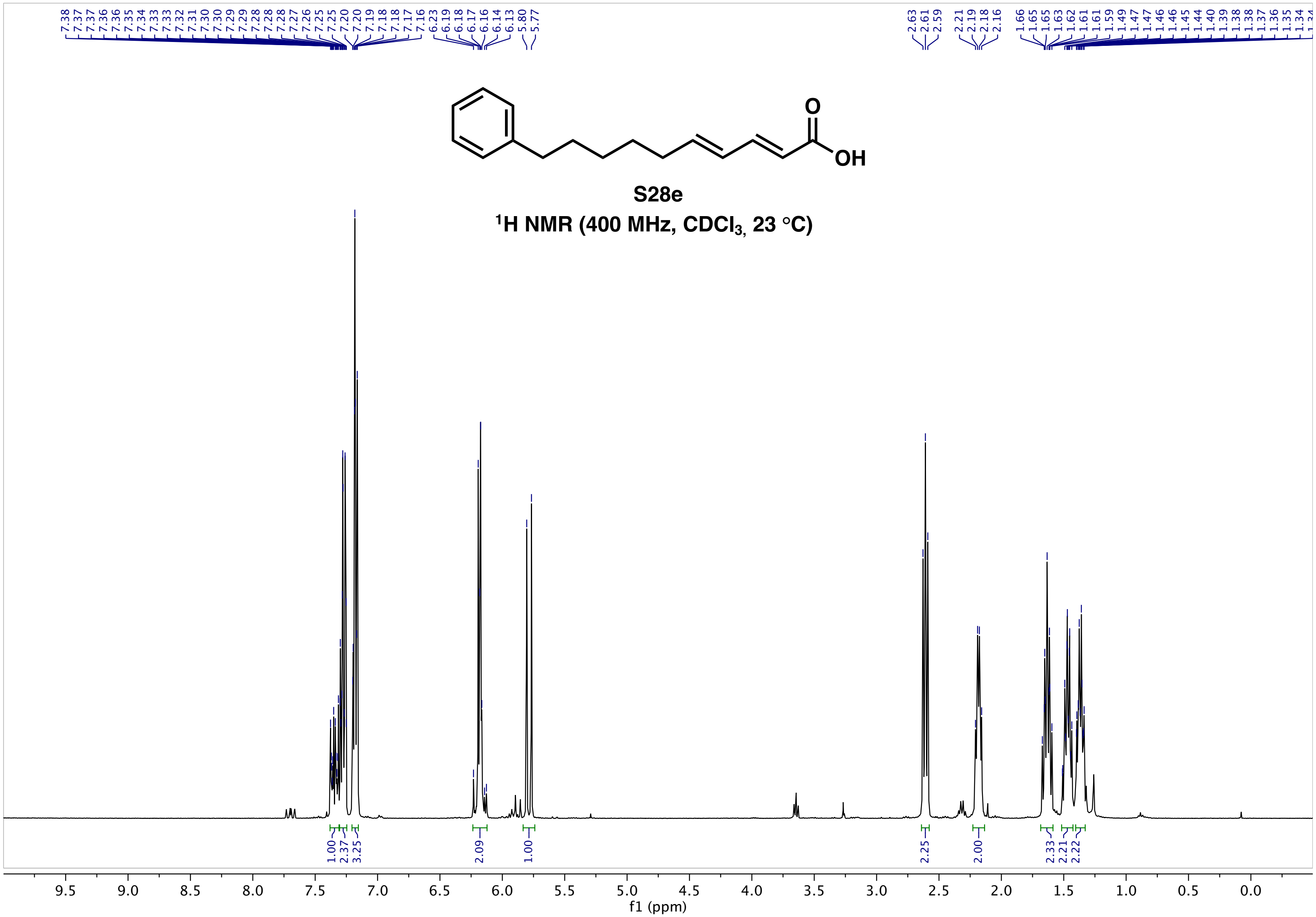

S28e
1H NMR (400 MHz, CDCl3, 23 °C)
f1 (ppm)

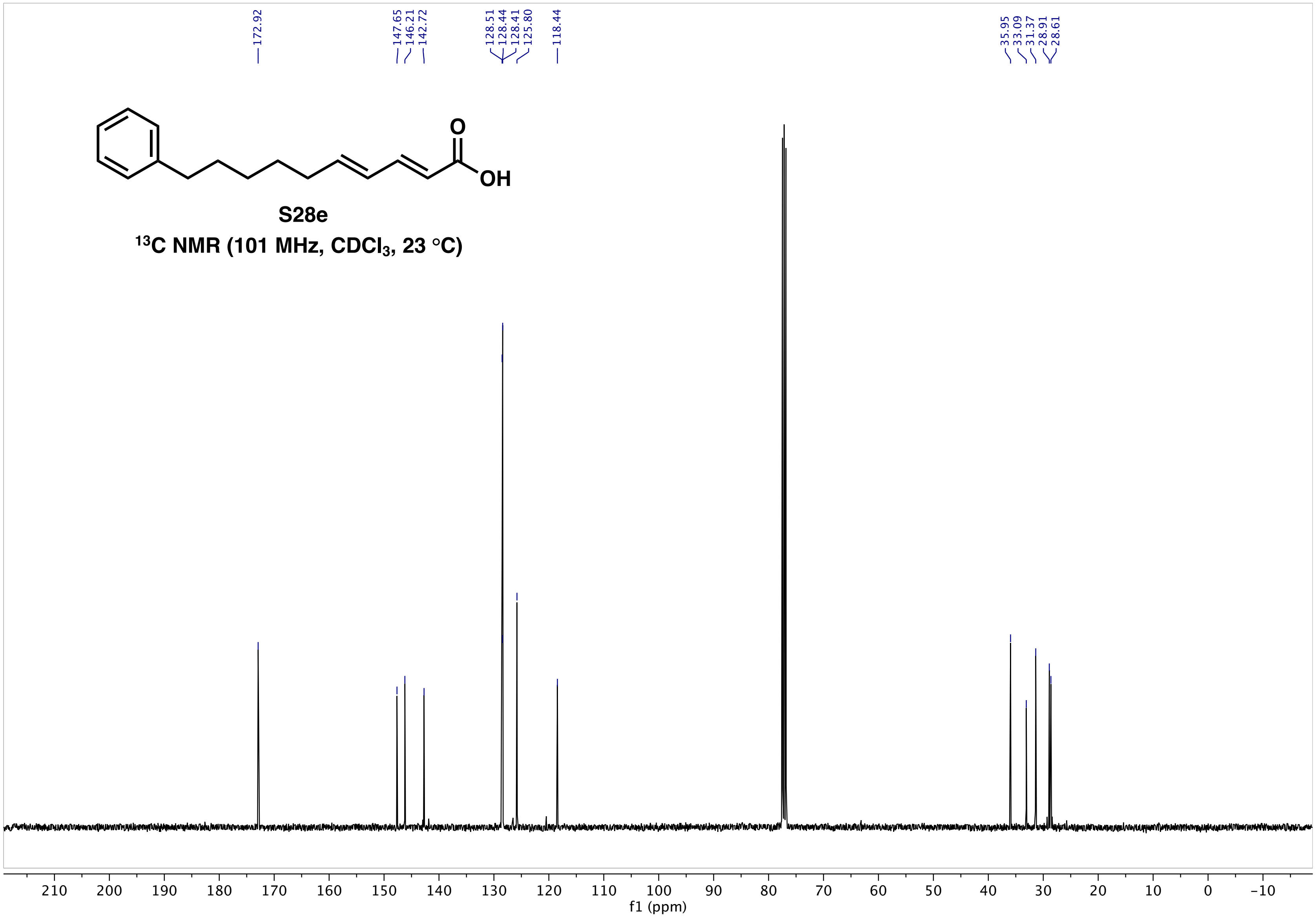

172.92
147.65
146.21
142.72
128.51
128.44
128.41
125.80
118.44
35.95
33.09
31.37
28.91
28.61
O
OH
S28e
13C NMR (101 MHz, CDCl3, 23 °C)
210 200 190 180 170 160 150 140 130 120 110 100 90 80 70 60 50 40 30 20 10 0 -10
f1 (ppm)

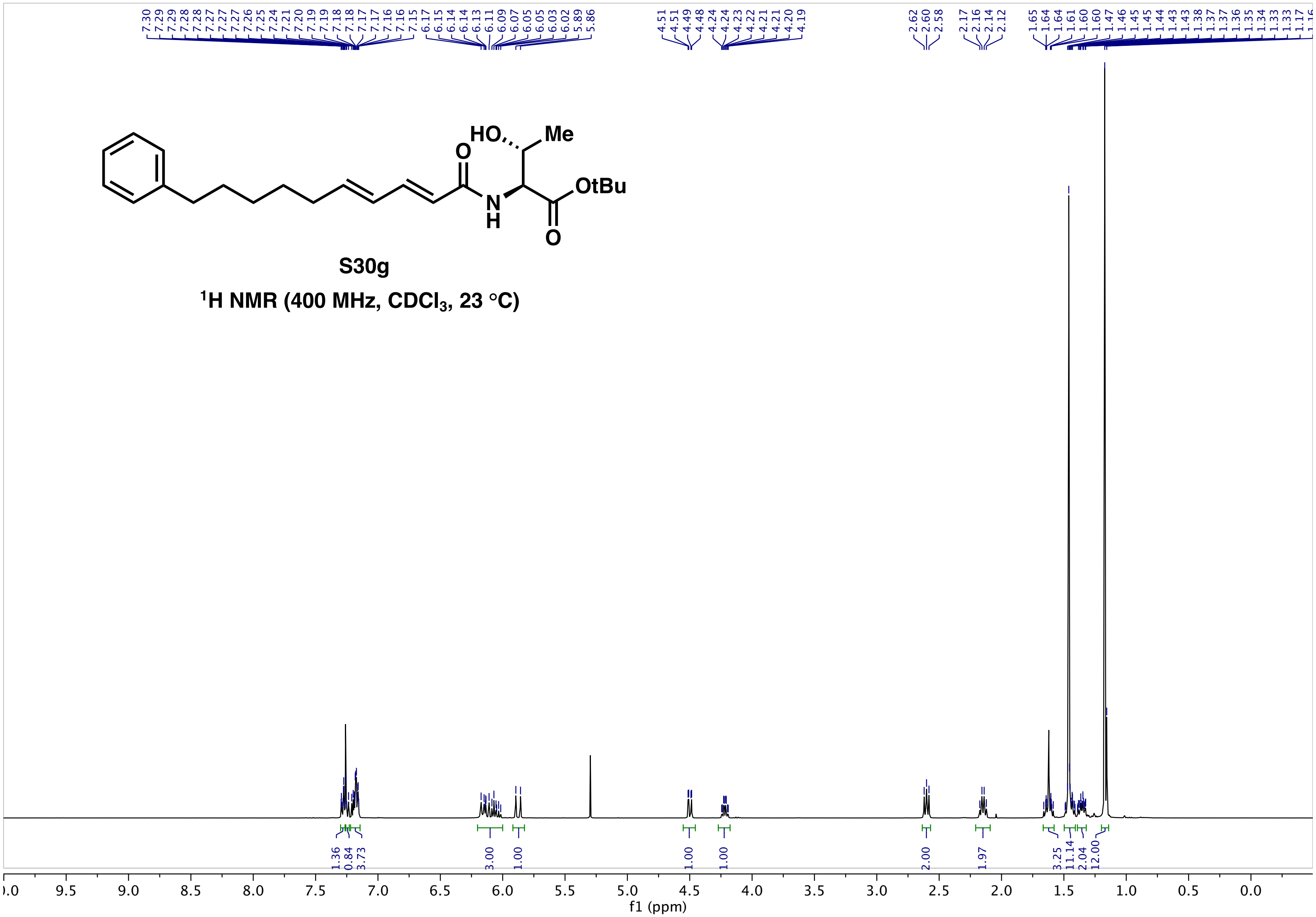
7.30 7.29 7.29 7.28 7.28 7.27 7.27 7.27 7.26 7.25 7.24 7.21 7.20 7.19 7.19 7.18 7.18 7.17 7.17 7.16 7.16 7.15
6.17 6.15 6.14 6.13 6.11 6.09 6.07 6.05 6.03 6.02 5.89 5.86
4.51 4.51 4.49 4.48 4.24 4.24 4.23 4.22 4.21 4.21 4.20 4.19
2.62 2.60 2.58
2.17 2.16 2.14 2.12
1.65 1.64 1.64 1.61 1.60 1.60 1.47 1.46 1.45 1.45 1.44 1.43 1.38 1.37 1.37 1.36 1.35 1.34 1.33 1.33 1.17
HO
Me
O
OtBu
N
H
O
S30g
^{1}H NMR (400 MHz, CDCl$_3$, 23 °C)
1.36 0.84 3.73 3.00 1.00 1.00 1.00 2.00 1.97 3.25 11.14 2.04 12.00
10.0 9.5 9.0 8.5 8.0 7.5 7.0 6.5 6.0 5.5 5.0 4.5 4.0 3.5 3.0 2.5 2.0 1.5 1.0 0.5 0.0
f1 (ppm)

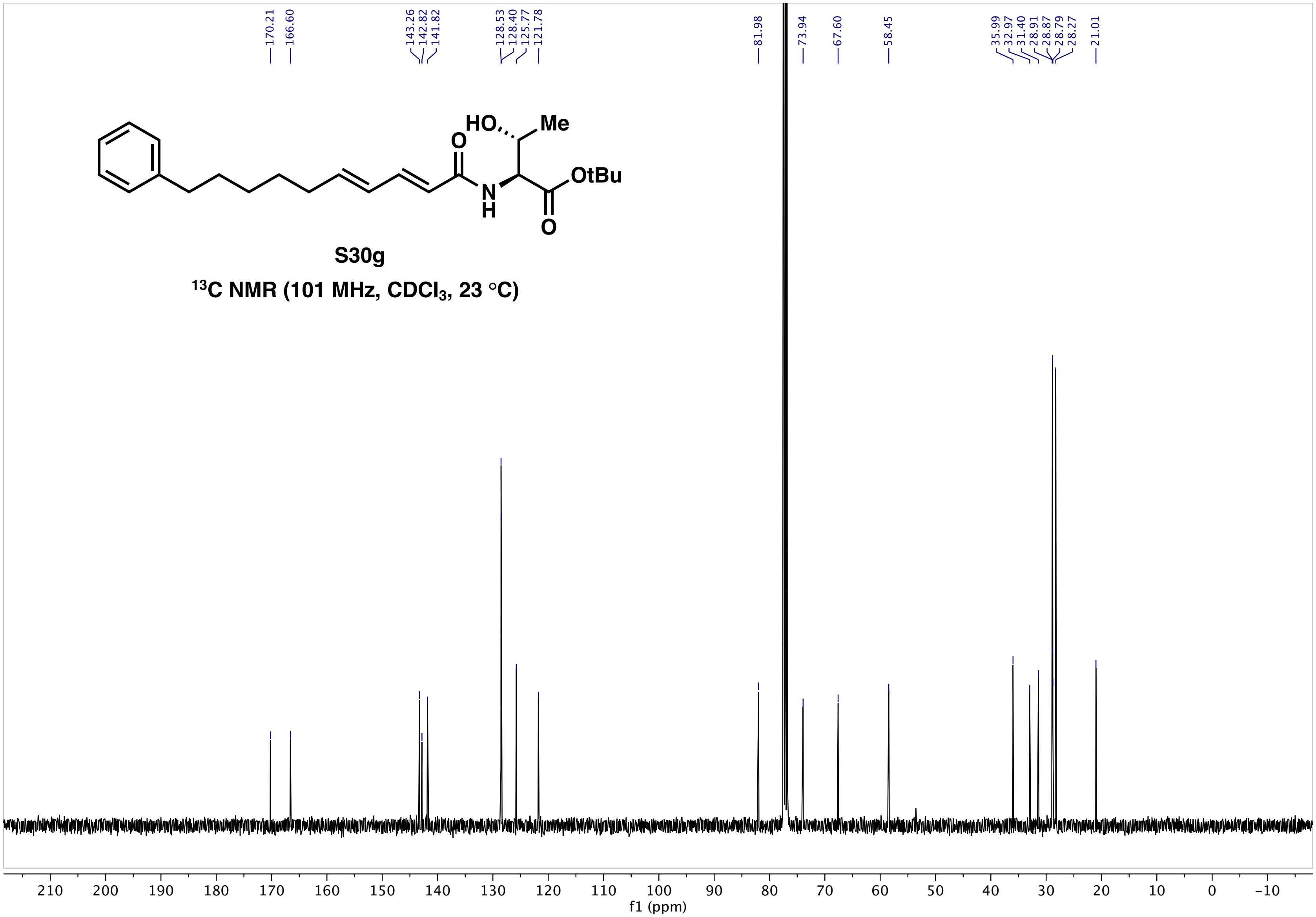
170.21
166.60
143.26
142.82
141.82
128.53
128.40
125.77
121.78
81.98
73.94
67.60
58.45
35.99
32.97
31.40
28.91
28.87
28.79
28.27
21.01
HO
Me
O
OtBu
N
H
O
S30g
^{13}C NMR (101 MHz, CDCl$_3$, 23 °C)
210
200
190
180
170
160
150
140
130
120
110
100
90
80
70
60
50
40
30
20
10
0
-10
f1 (ppm)

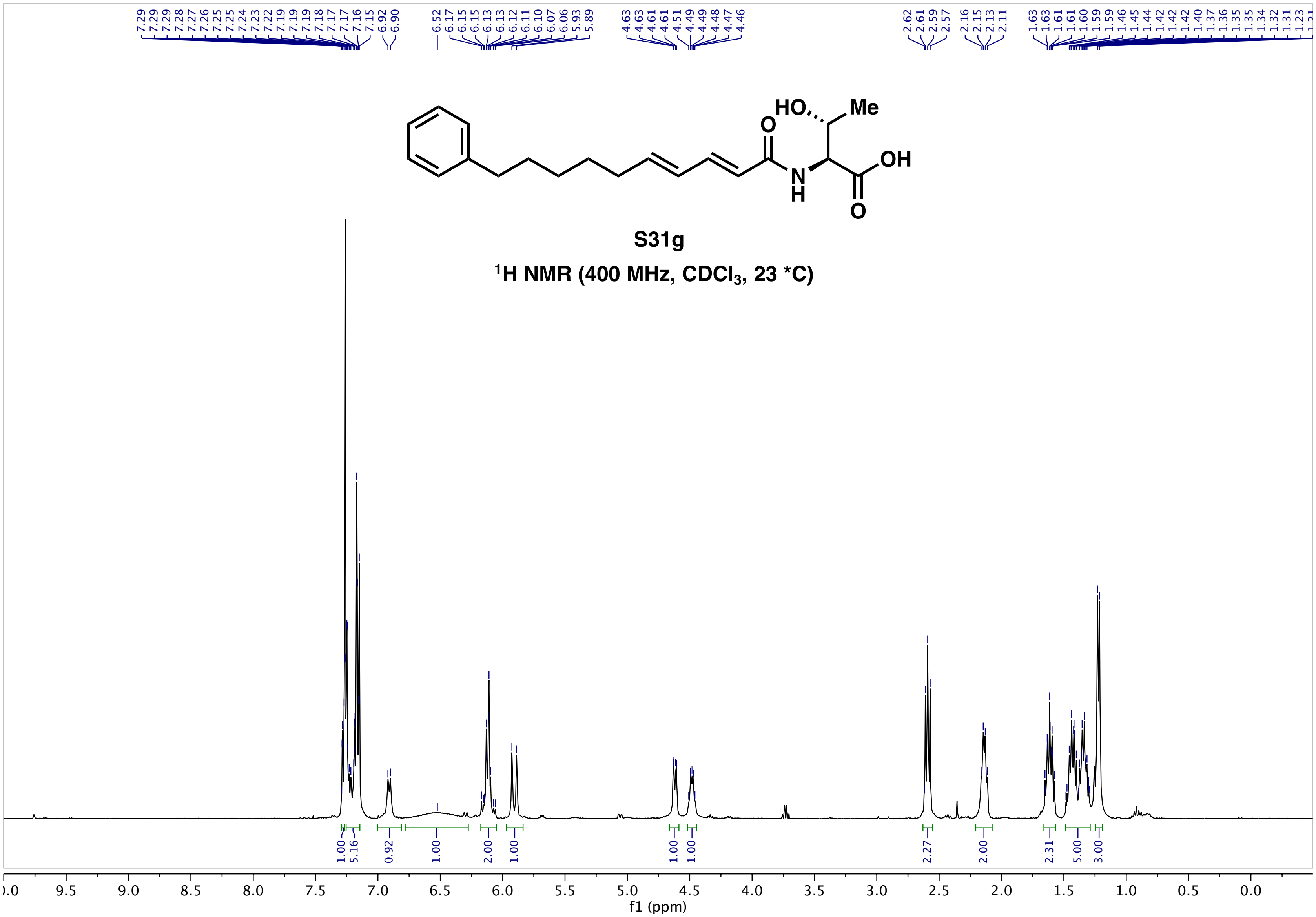

S31g
^{1}H NMR (400 MHz, CDCl$_3$, 23 *C)
f1 (ppm)

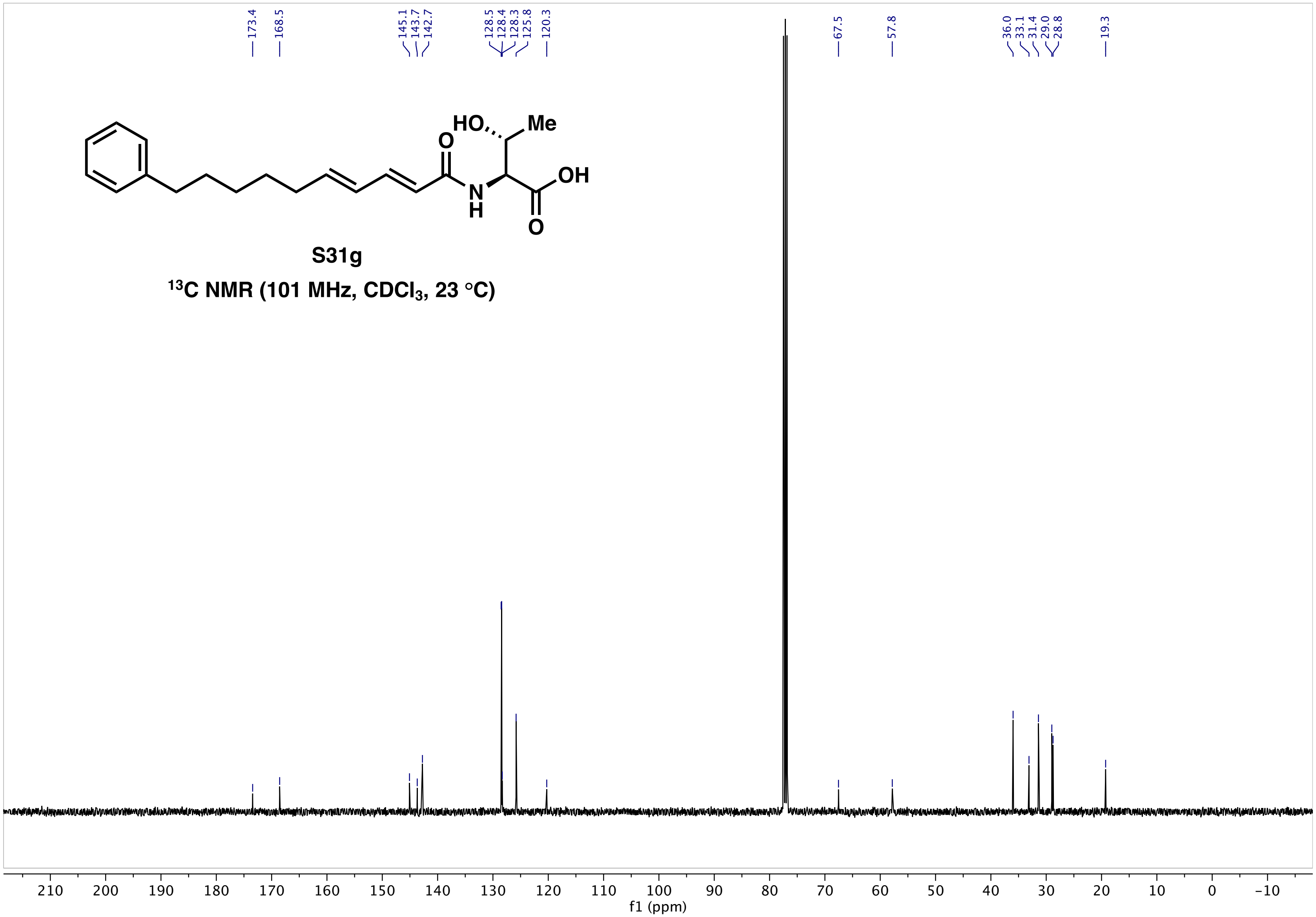

HO
Me
O
OH
N
H
O
S31g
^{13}C NMR (101 MHz, $CDCl_3$, 23 °C)
173.4
168.5
145.1
143.7
142.7
128.5
128.4
128.3
125.8
120.3
67.5
57.8
36.0
33.1
31.4
29.0
28.8
19.3
210 200 190 180 170 160 150 140 130 120 110 100 90 80 70 60 50 40 30 20 10 0 -10
f1 (ppm)

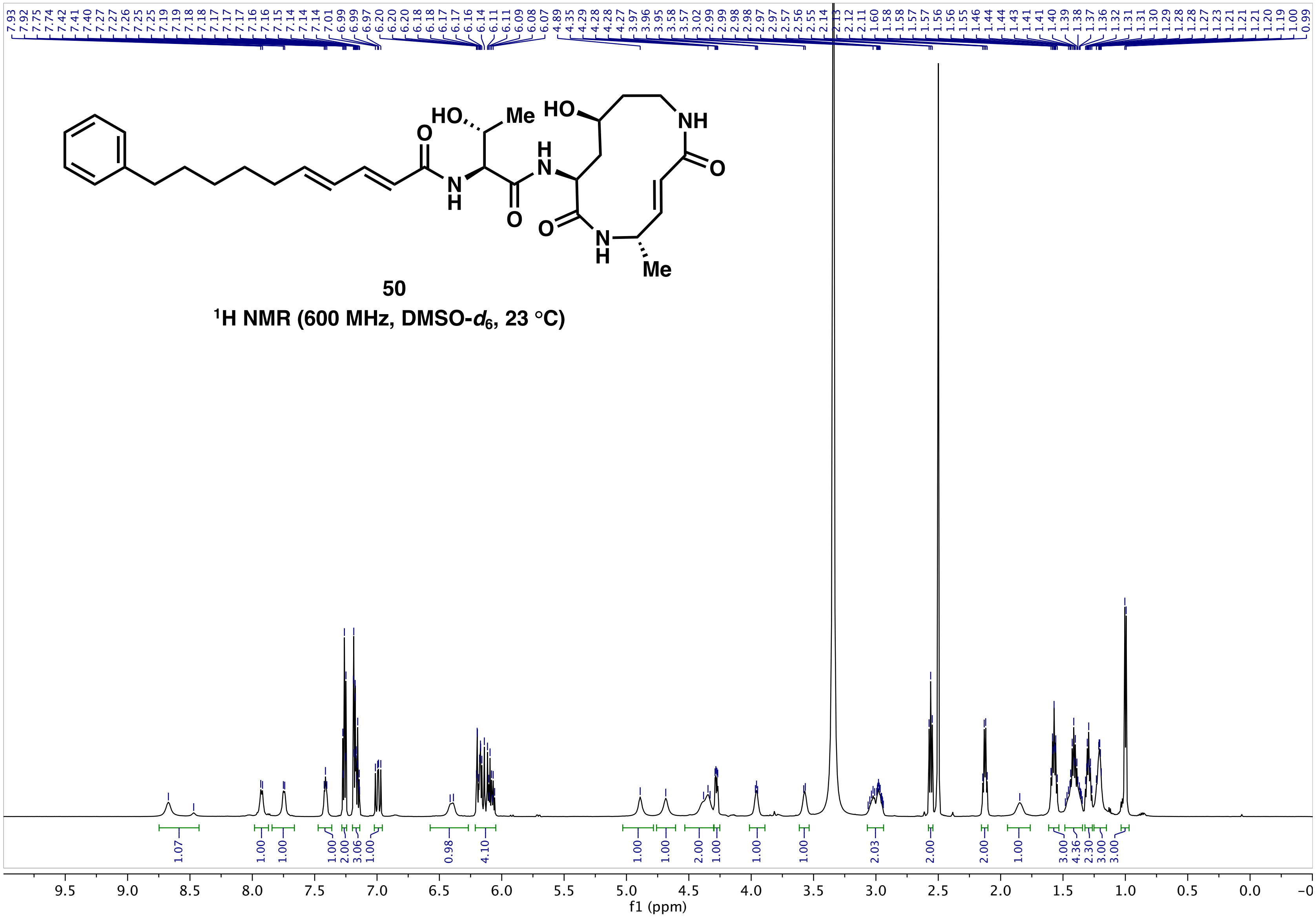
50
1H NMR (600 MHz, DMSO-d6, 23 °C)
f1 (ppm)

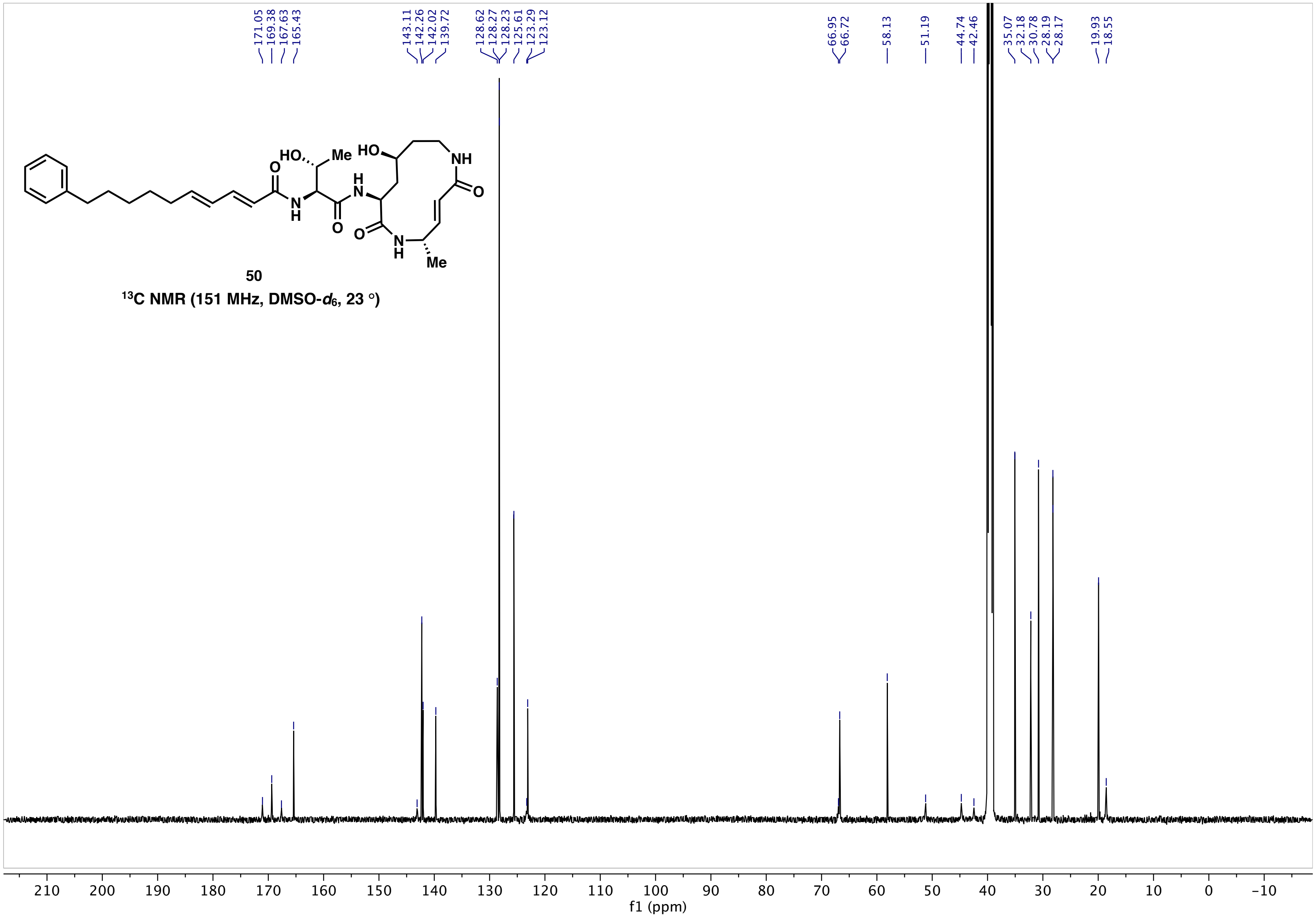
171.05
169.38
167.63
165.43
143.11
142.26
142.02
139.72
128.62
128.27
128.23
125.61
123.29
123.12
66.95
66.72
58.13
51.19
44.74
42.46
35.07
32.18
30.78
28.19
28.17
19.93
18.55
HO
Me
HO
NH
O
O
N
H
H
N
O
O
N
H
Me
50
^{13}C NMR (151 MHz, DMSO-d_6, 23 °)
210
200
190
180
170
160
150
140
130
120
110
100
90
80
70
60
50
40
30
20
10
0
–10
f1 (ppm)

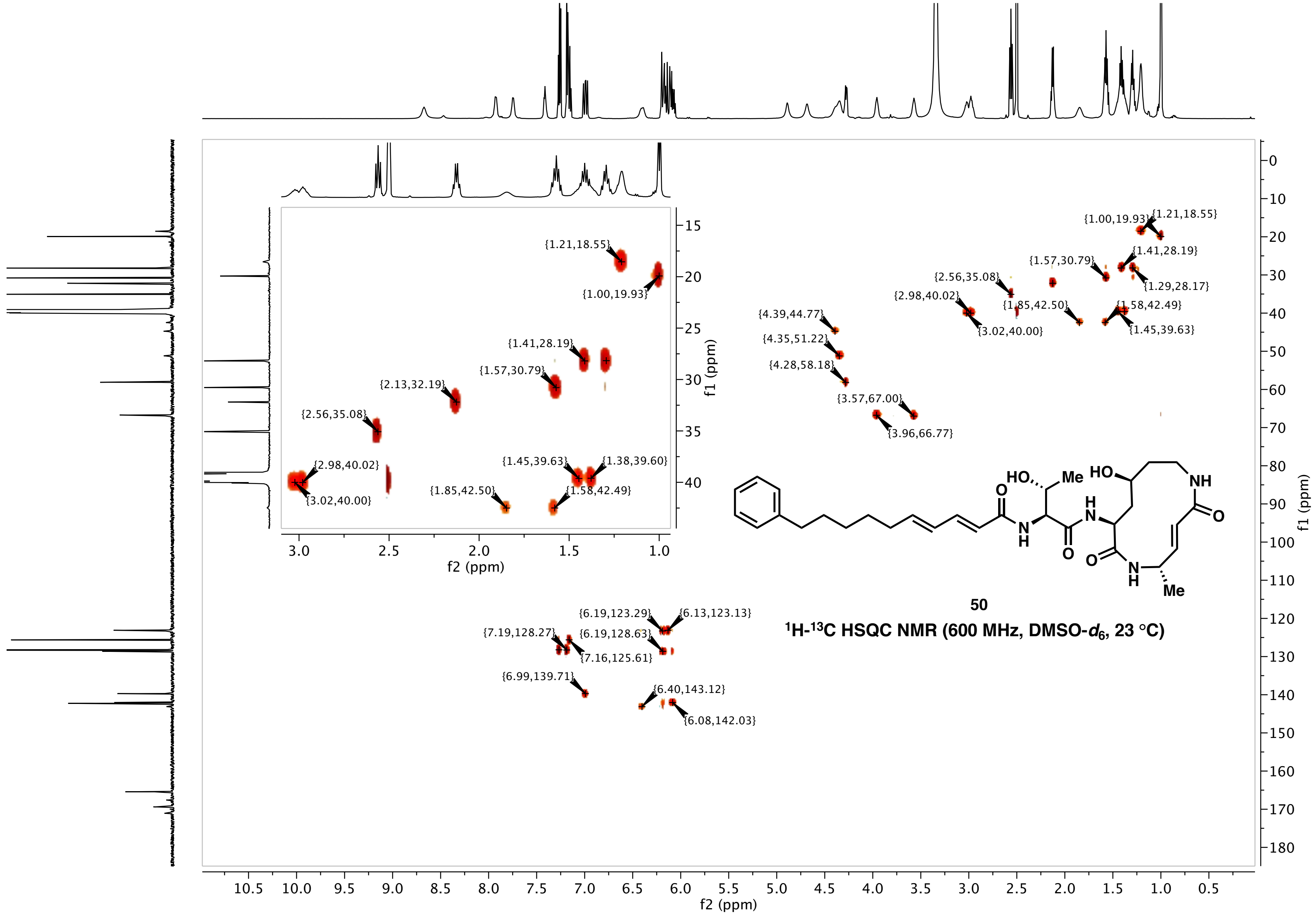

50

^{1}H-^{13}C HSQC NMR (600 MHz, DMSO-d_6, 23 °C)

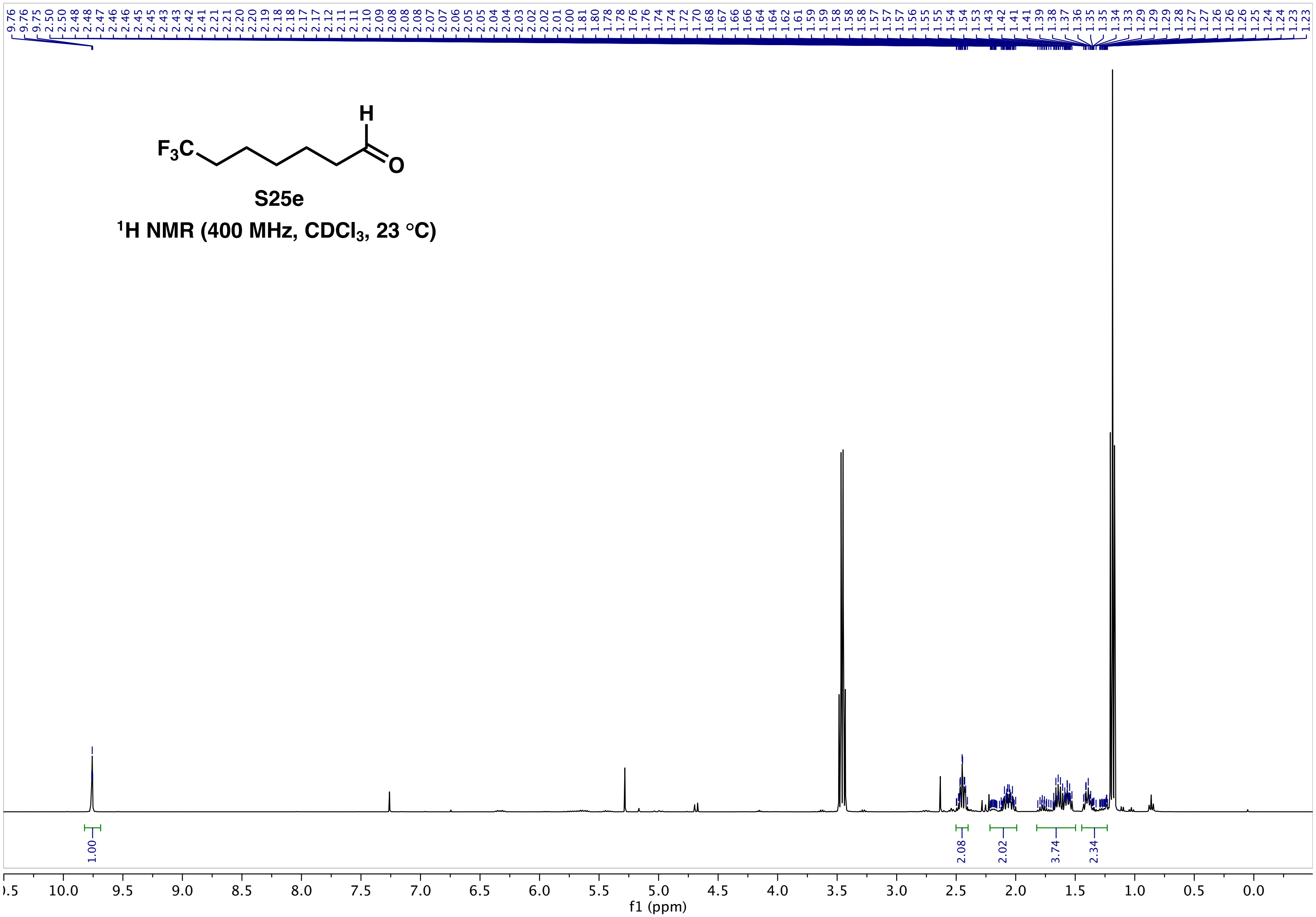

9.76 9.76 9.75 2.50 2.50 2.48 2.48 2.47 2.46 2.45 2.45 2.43 2.43 2.42 2.41 2.21 2.21 2.20 2.20 2.19 2.18 2.18 2.17 2.17 2.12 2.11 2.11 2.10 2.09 2.08 2.08 2.08 2.07 2.07 2.06 2.05 2.05 2.04 2.04 2.03 2.02 2.02 2.01 2.00 1.81 1.80 1.78 1.78 1.76 1.74 1.74 1.72 1.70 1.68 1.67 1.66 1.66 1.64 1.64 1.62 1.61 1.59 1.59 1.58 1.58 1.58 1.57 1.57 1.57 1.56 1.55 1.55 1.54 1.54 1.53 1.43 1.42 1.41 1.41 1.39 1.38 1.37 1.36 1.35 1.35 1.35 1.34 1.33 1.29 1.29 1.28 1.27 1.26 1.26 1.25 1.24 1.24 1.23 1.23
F_3C
H
O
S25e
^{1}H NMR (400 MHz, $CDCl_3$, 23 °C)
1.00
2.08
2.02
3.74
2.34
10.0 9.5 9.0 8.5 8.0 7.5 7.0 6.5 6.0 5.5 5.0 4.5 4.0 3.5 3.0 2.5 2.0 1.5 1.0 0.5 0.0
f1 (ppm)

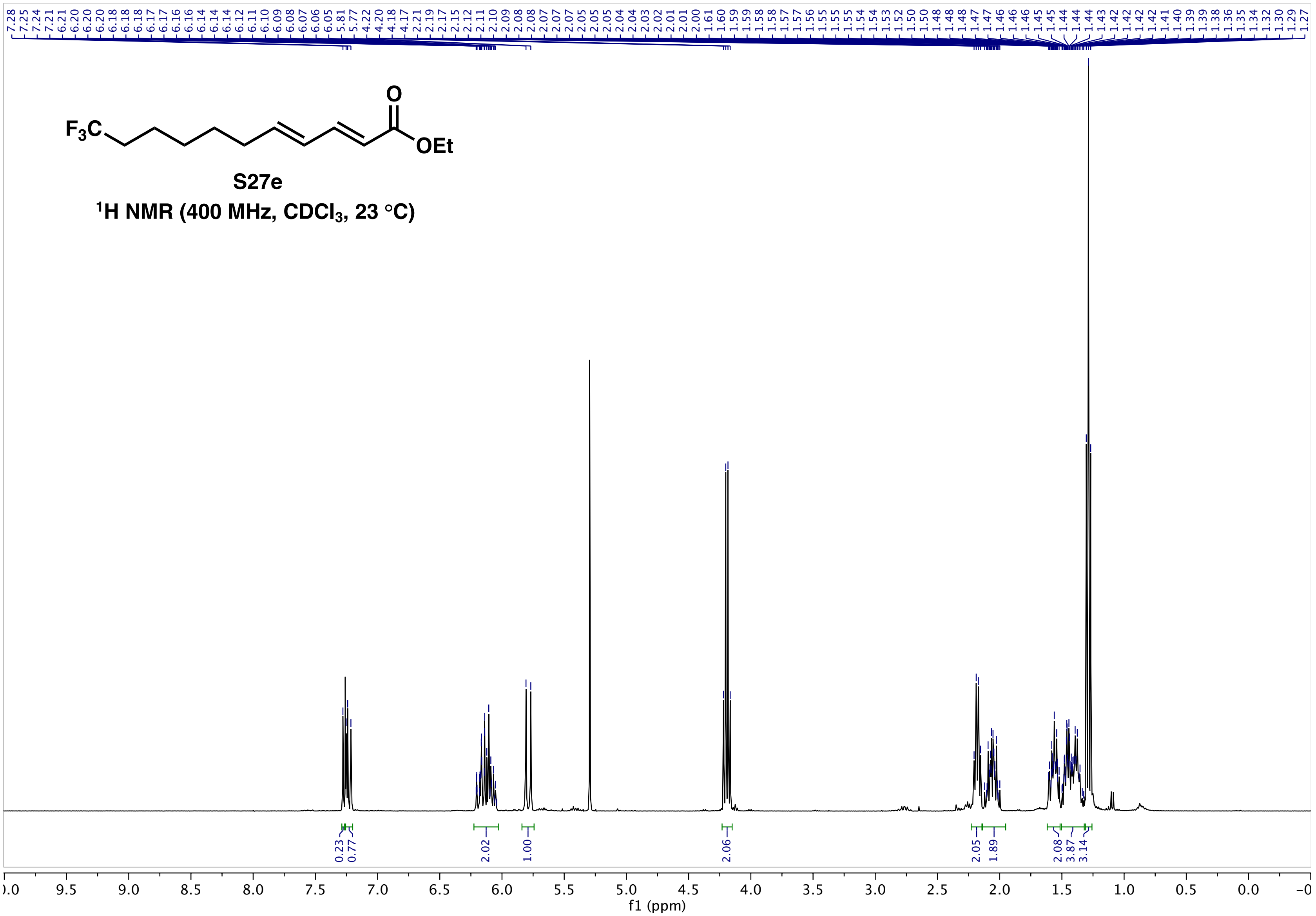
S27e
¹H NMR (400 MHz, CDCl₃, 23 °C)
f1 (ppm)

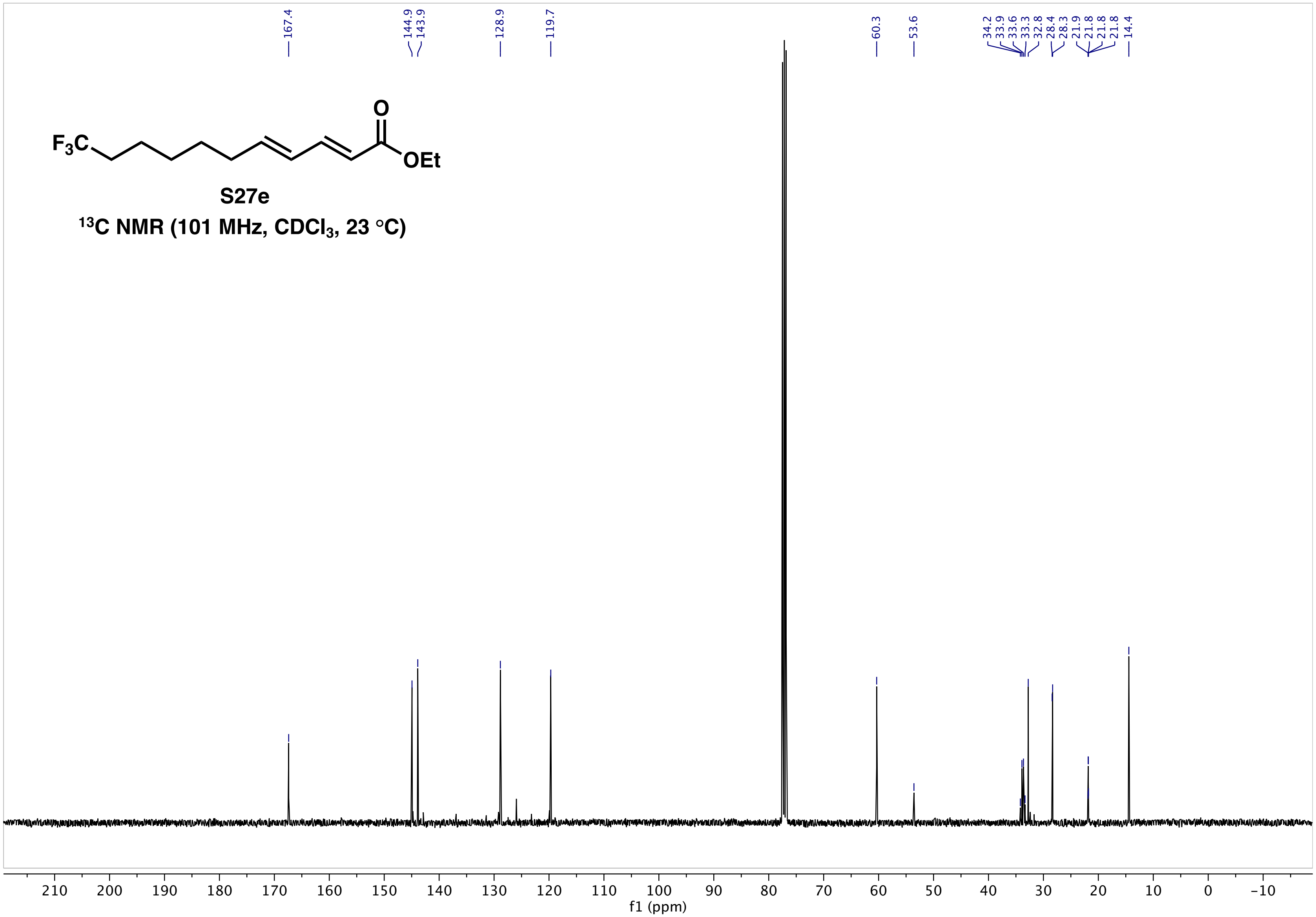

F3C
O
OEt
S27e
13C NMR (101 MHz, CDCl3, 23 °C)
167.4
144.9
143.9
128.9
119.7
60.3
53.6
34.2
33.9
33.6
33.3
32.8
28.4
28.3
21.9
21.8
21.8
21.8
14.4
210 200 190 180 170 160 150 140 130 120 110 100 90 80 70 60 50 40 30 20 10 0 -10
f1 (ppm)

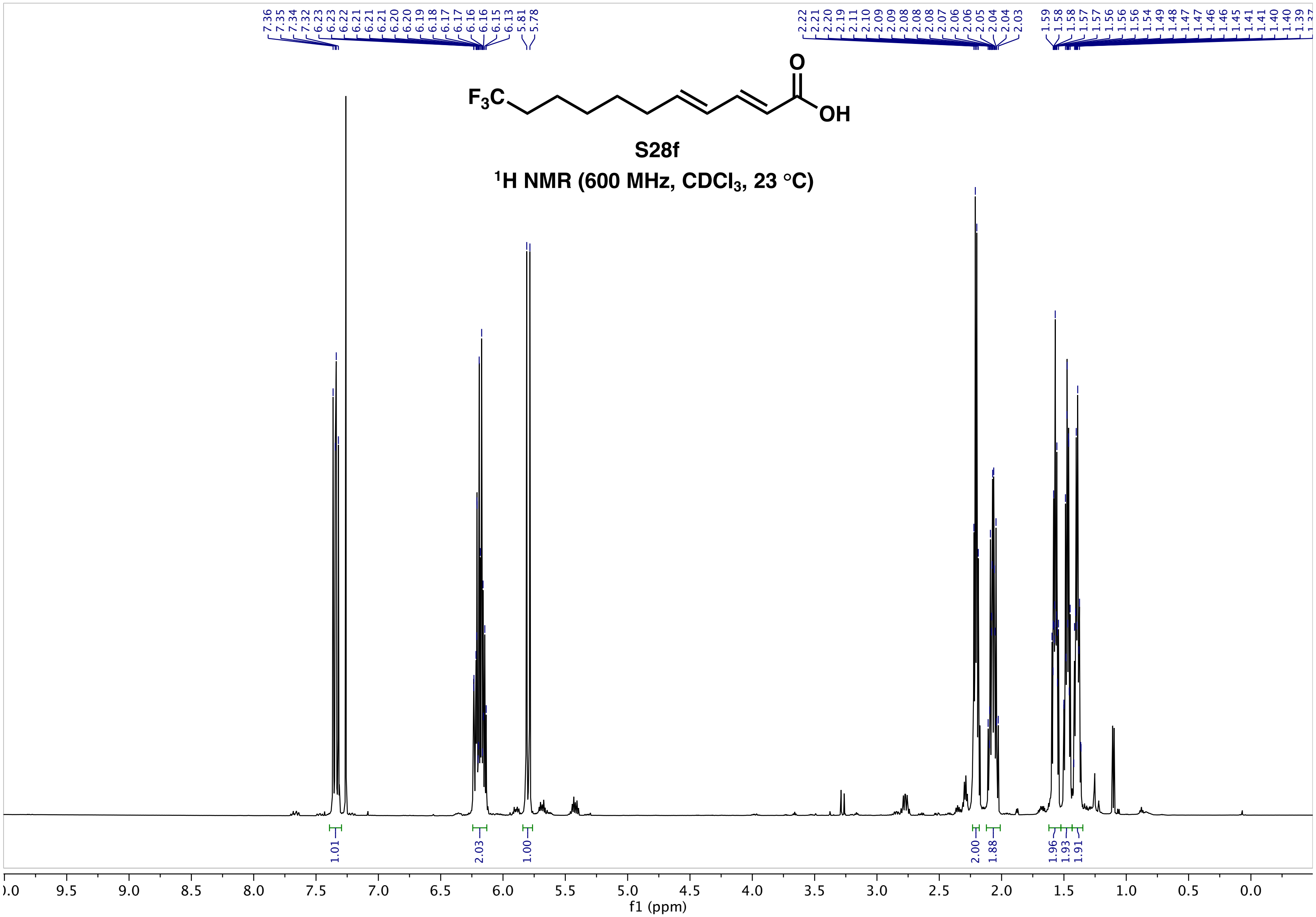
S28f
1H NMR (600 MHz, CDCl3, 23 °C)
f1 (ppm)

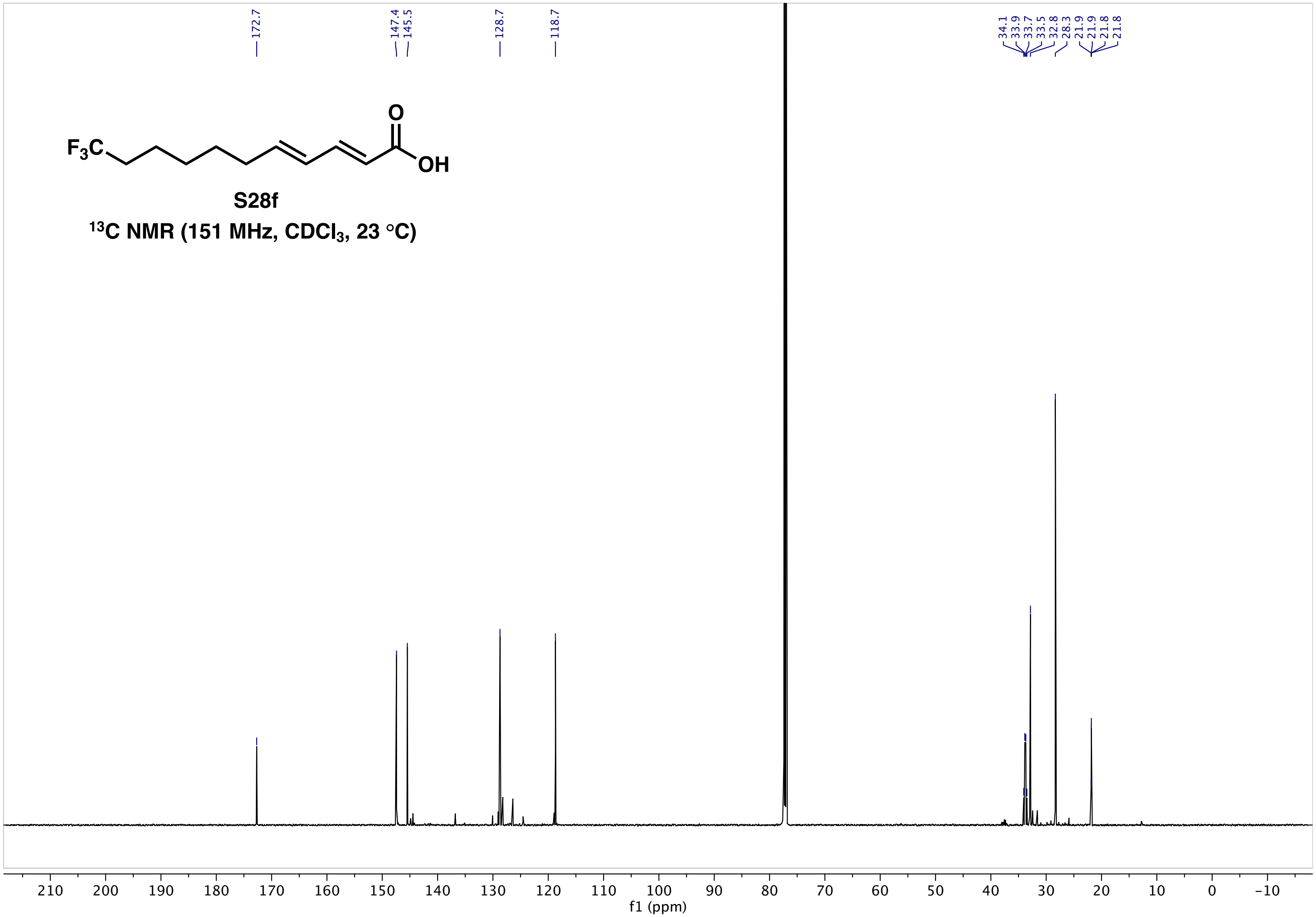

F3C
O
OH
S28f
13C NMR (151 MHz, CDCl3, 23 °C)
172.7
147.4
145.5
128.7
118.7
34.1
33.9
33.7
33.5
32.8
28.3
21.9
21.9
21.8
21.8
210 200 190 180 170 160 150 140 130 120 110 100 90 80 70 60 50 40 30 20 10 0 −10
f1 (ppm)

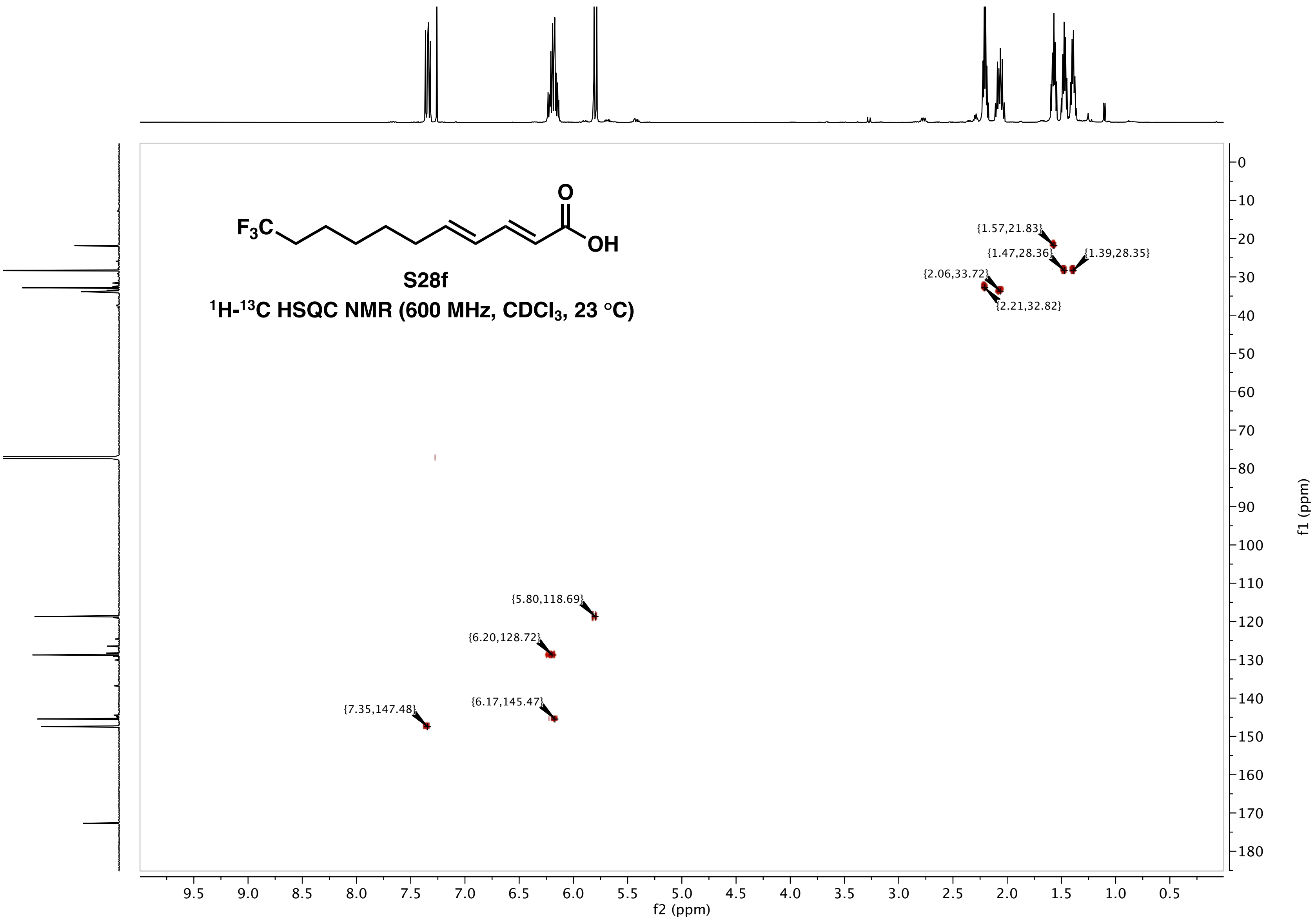

F3C
O
OH
S28f
1H-13C HSQC NMR (600 MHz, CDCl3, 23 °C)
{1.57,21.83}
{1.47,28.36}
{1.39,28.35}
{2.06,33.72}
{2.21,32.82}
{5.80,118.69}
{6.20,128.72}
{6.17,145.47}
{7.35,147.48}
f2 (ppm)
f1 (ppm)

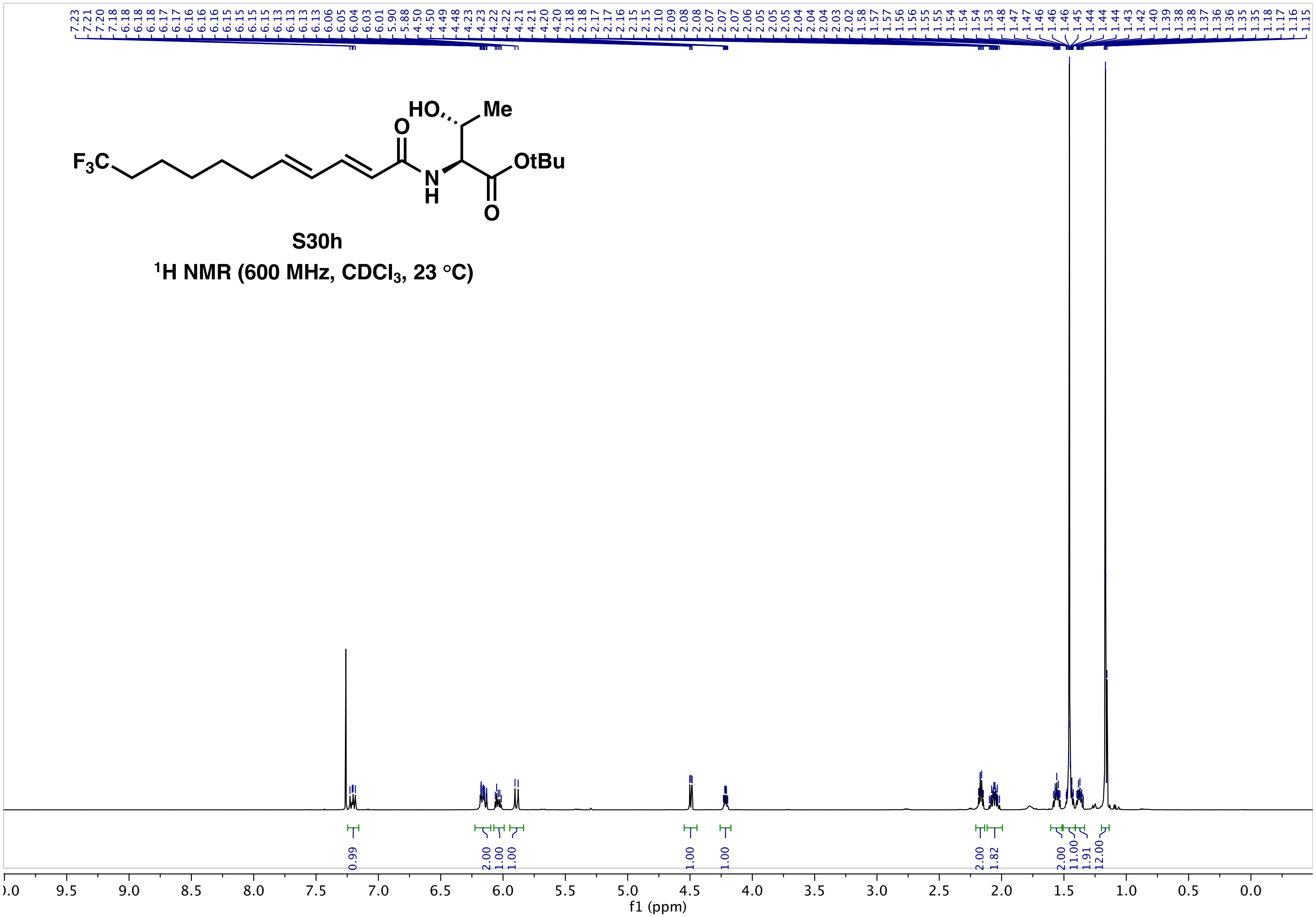

HO
Me
O
F_3C
N
H
OtBu
O
S30h
^{1}H NMR (600 MHz, $CDCl_3$, 23 °C)
0.99
2.00
1.00
1.00
1.00
1.00
2.00
1.82
2.00
11.00
1.91
12.00
f1 (ppm)

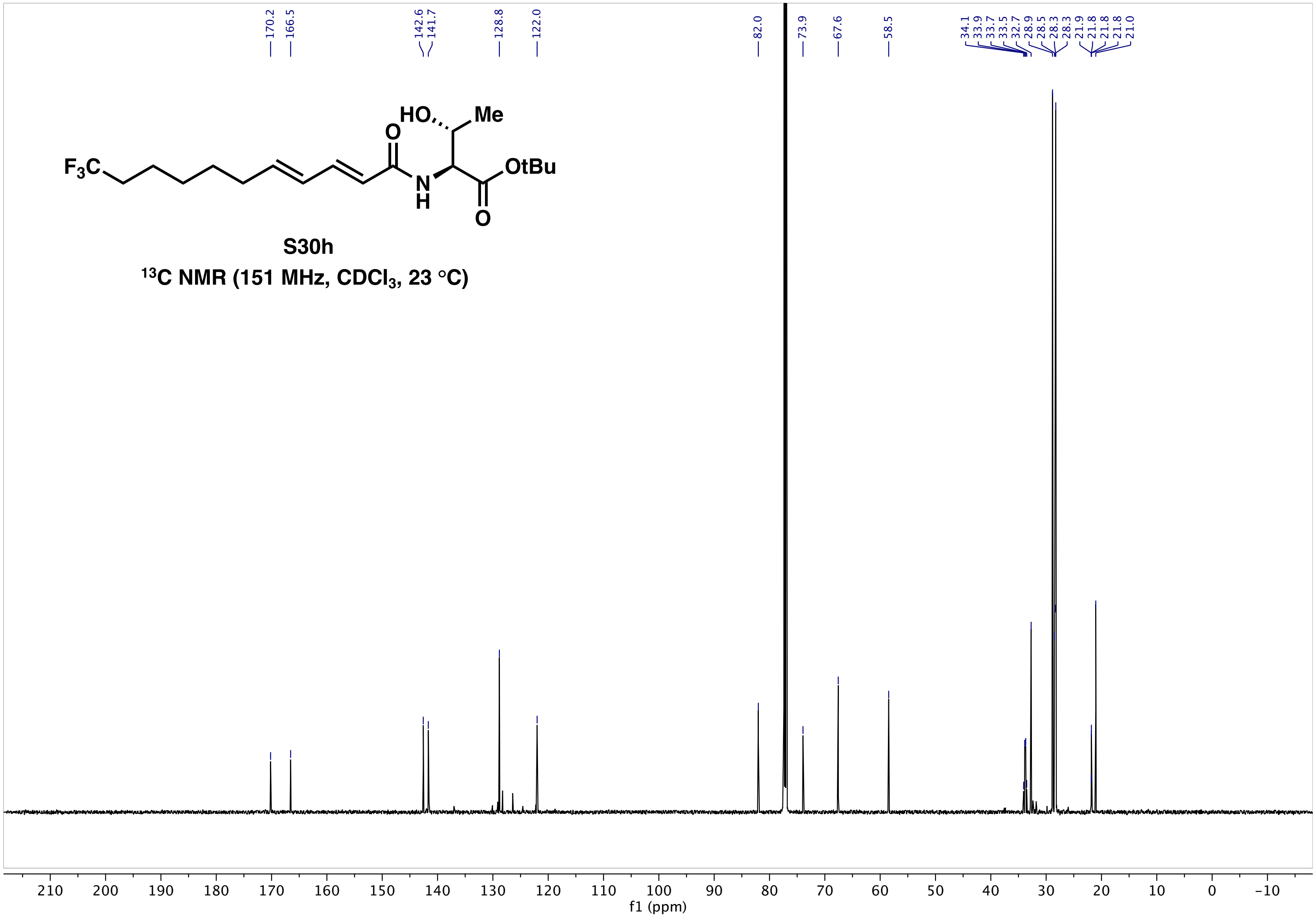
170.2
166.5
142.6
141.7
128.8
122.0
82.0
73.9
67.6
58.5
34.1
33.9
33.7
33.5
32.7
28.9
28.5
28.3
28.3
21.9
21.8
21.8
21.8
21.0
HO
Me
O
F3C
N
H
OtBu
O
S30h
13C NMR (151 MHz, CDCl3, 23 °C)
210 200 190 180 170 160 150 140 130 120 110 100 90 80 70 60 50 40 30 20 10 0 -10
f1 (ppm)

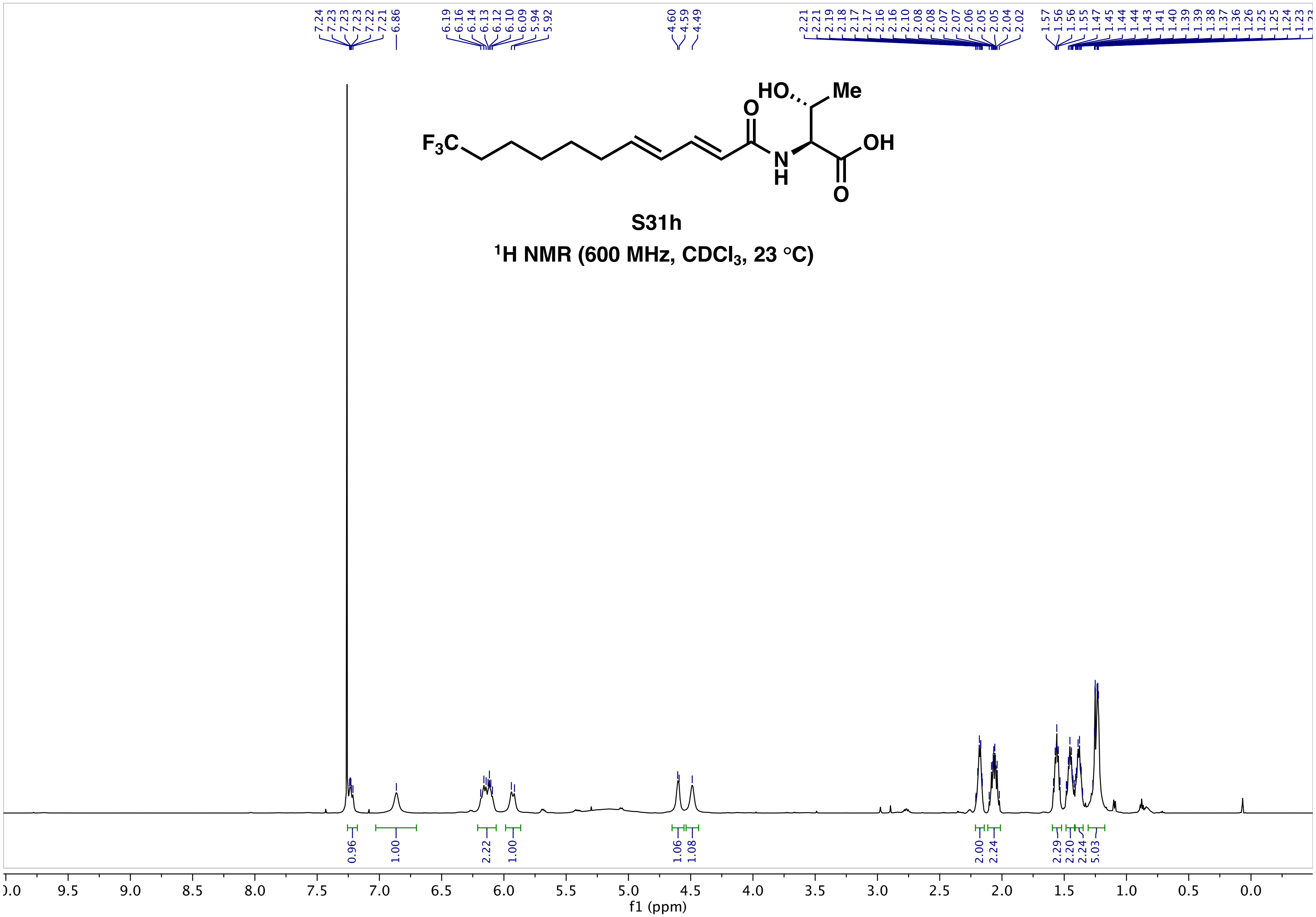
HO
Me
O
F_3C
N
H
OH
O
S31h
¹H NMR (600 MHz, CDCl₃, 23 °C)
f1 (ppm)

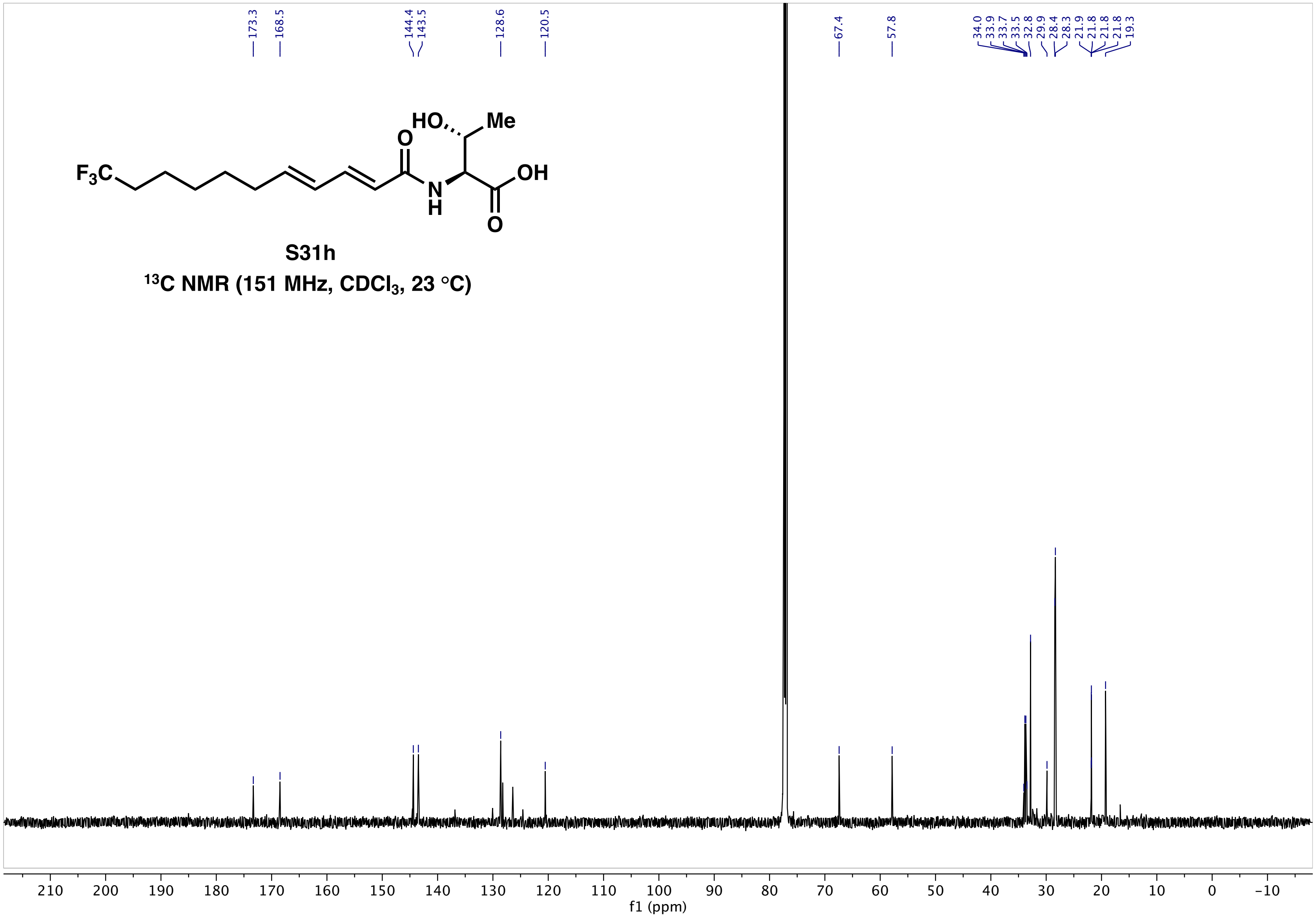

S31h

^{13}C NMR (151 MHz, $CDCl_3$, 23 °C)

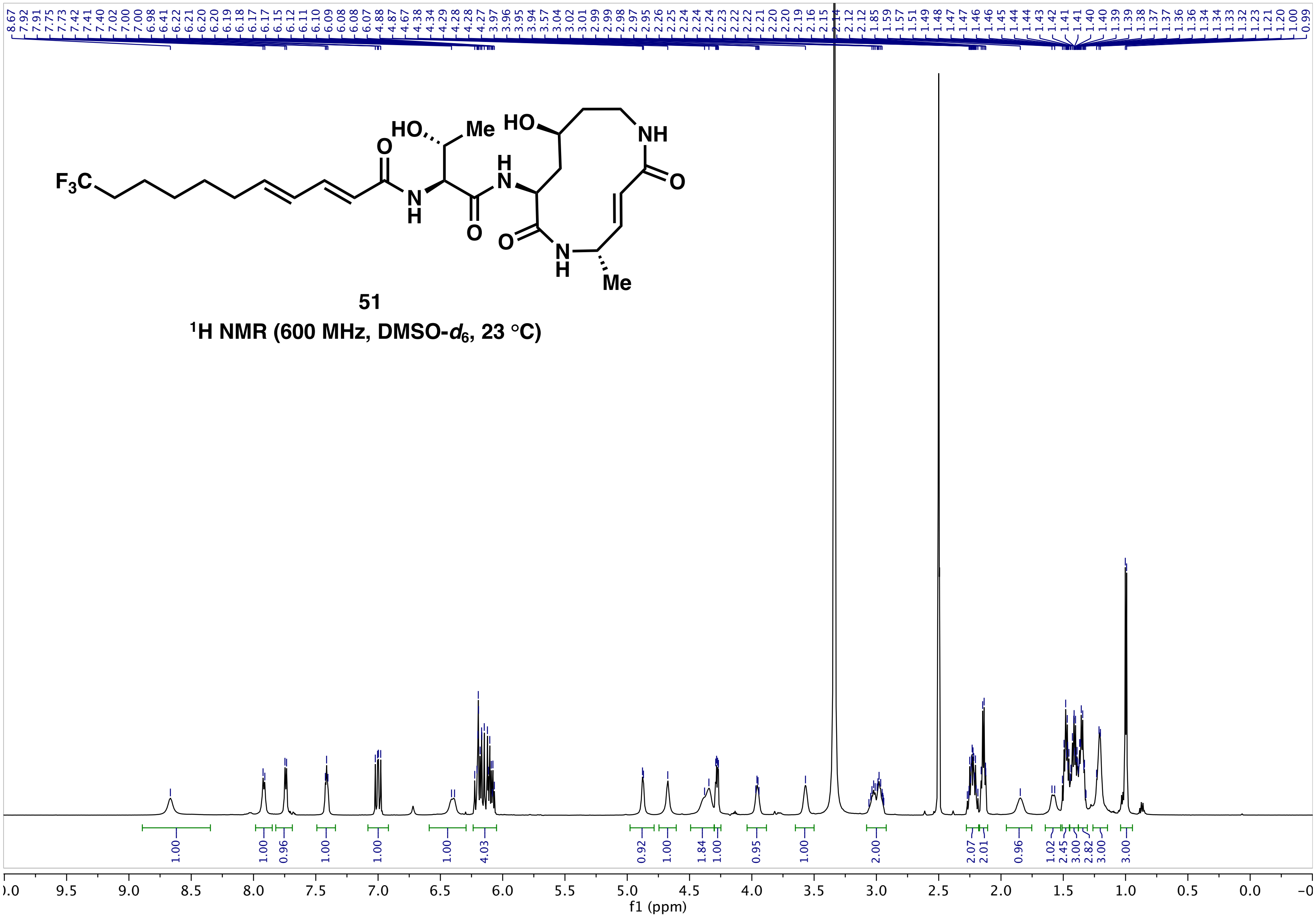
F3C
HO
Me
HO
NH
O
N
H
O
O
O
N
H
Me
51
1H NMR (600 MHz, DMSO-d6, 23 °C)
f1 (ppm)

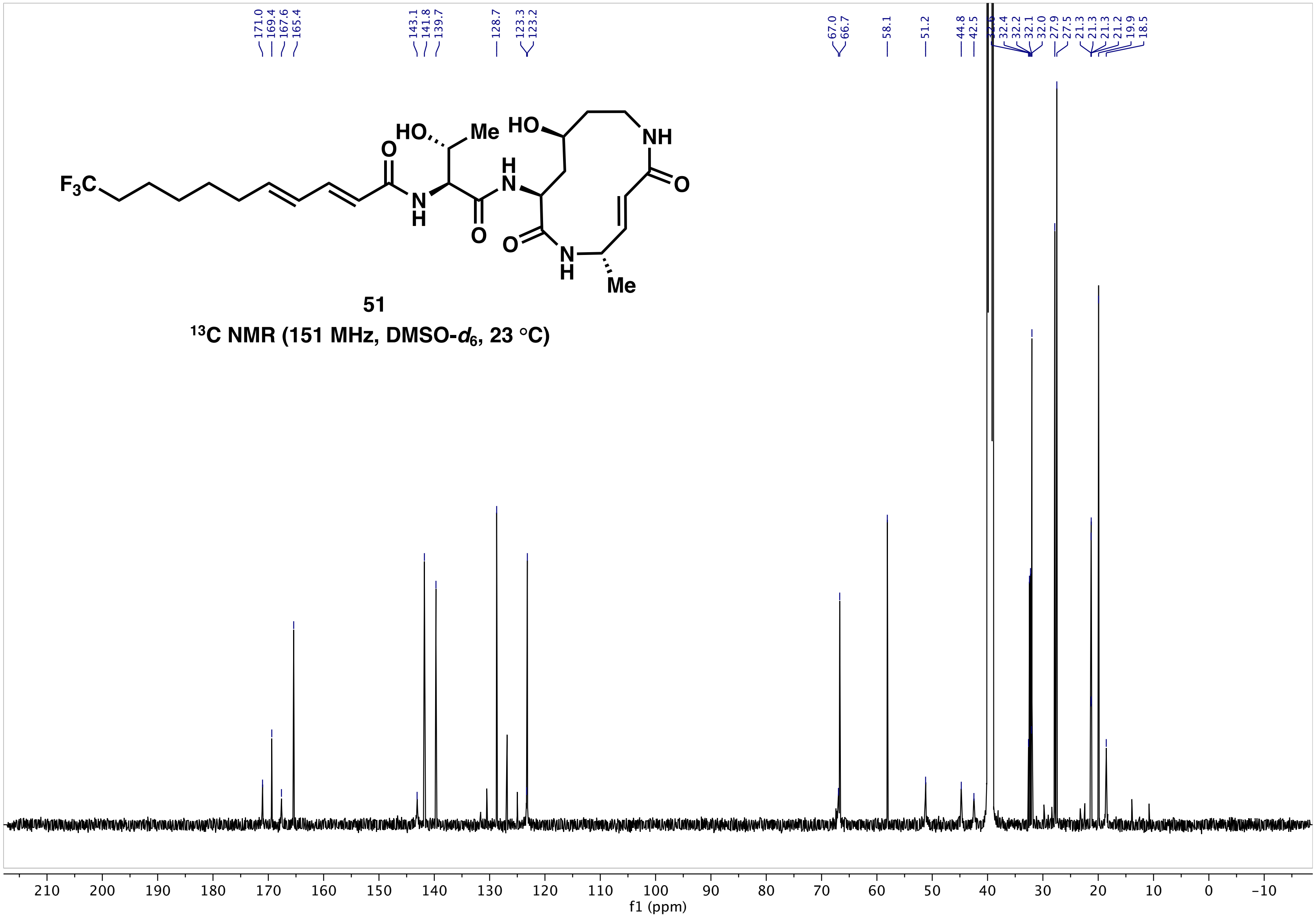

HO
Me
HO
NH
O
H
F3C
N
O
N
H
O
O
N
H
Me
51
13C NMR (151 MHz, DMSO-d6, 23 °C)
171.0
169.4
167.6
165.4
143.1
141.8
139.7
128.7
123.3
123.2
67.0
66.7
58.1
51.2
44.8
42.5
32.4
32.2
32.1
32.0
27.9
27.5
21.3
21.3
21.3
21.2
19.9
18.5
f1 (ppm)

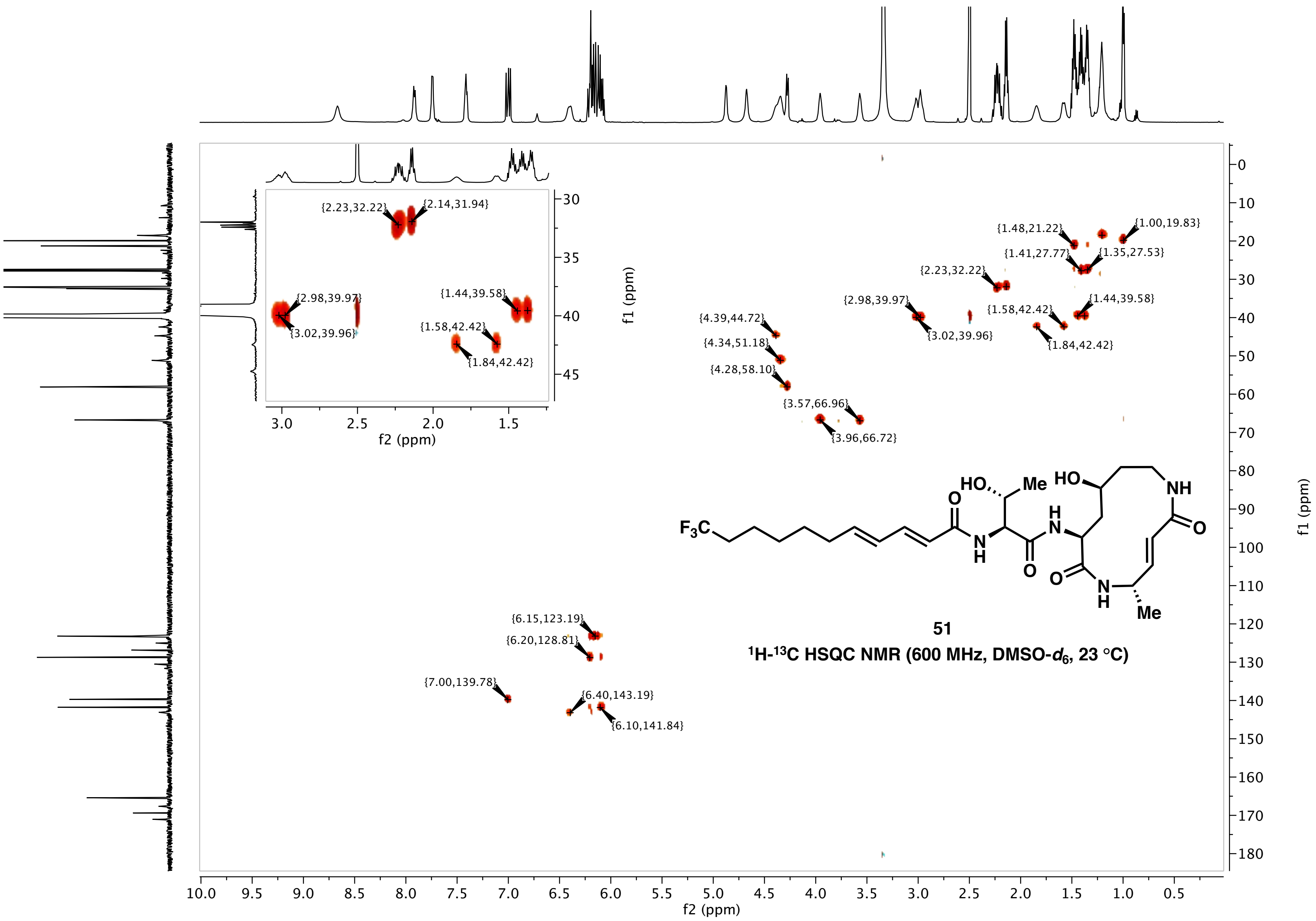
51
¹H-¹³C HSQC NMR (600 MHz, DMSO-d6, 23 °C)
f2 (ppm)
f1 (ppm)

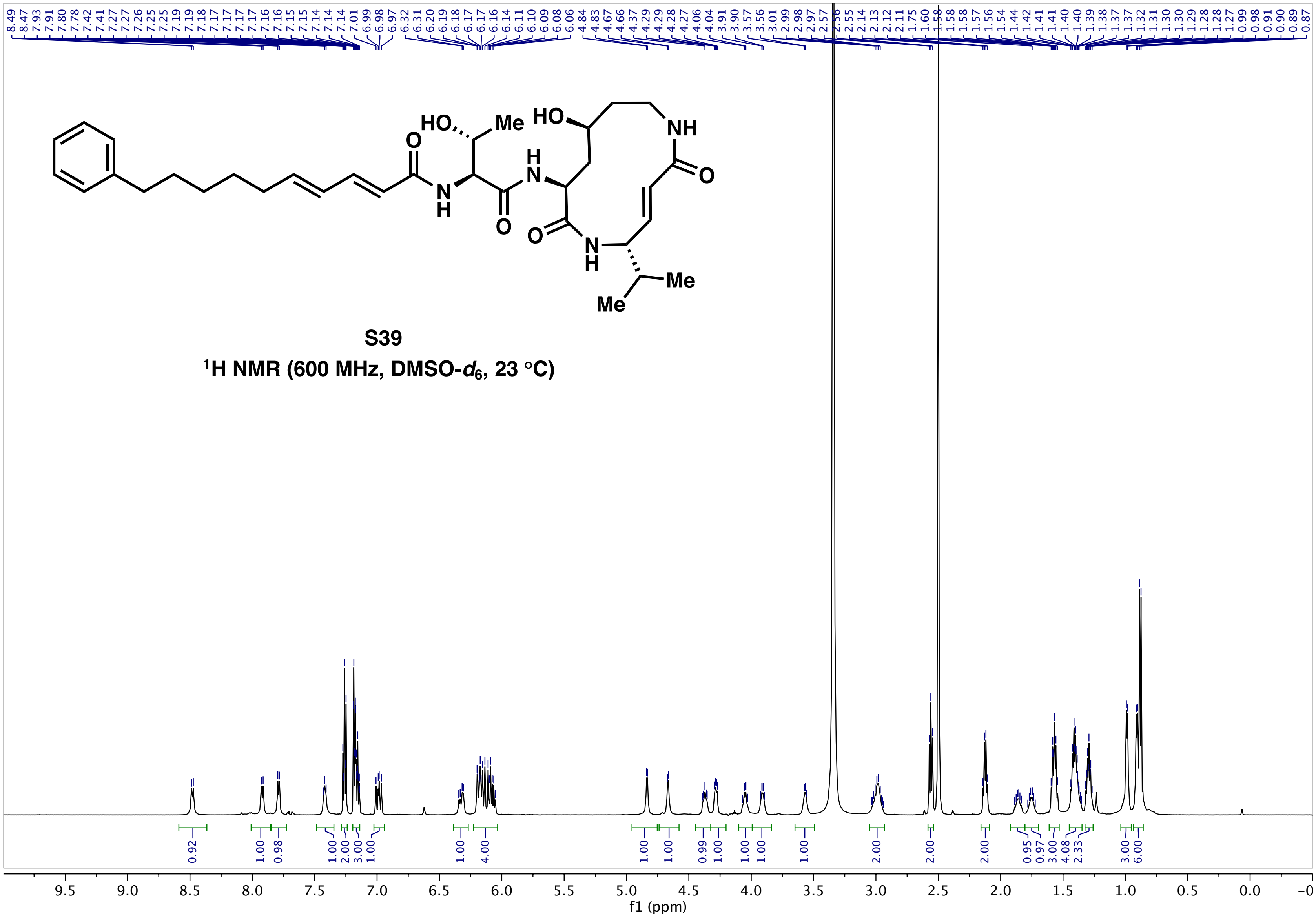
S39
^1H NMR (600 MHz, DMSO-d_6, 23 °C)
f1 (ppm)

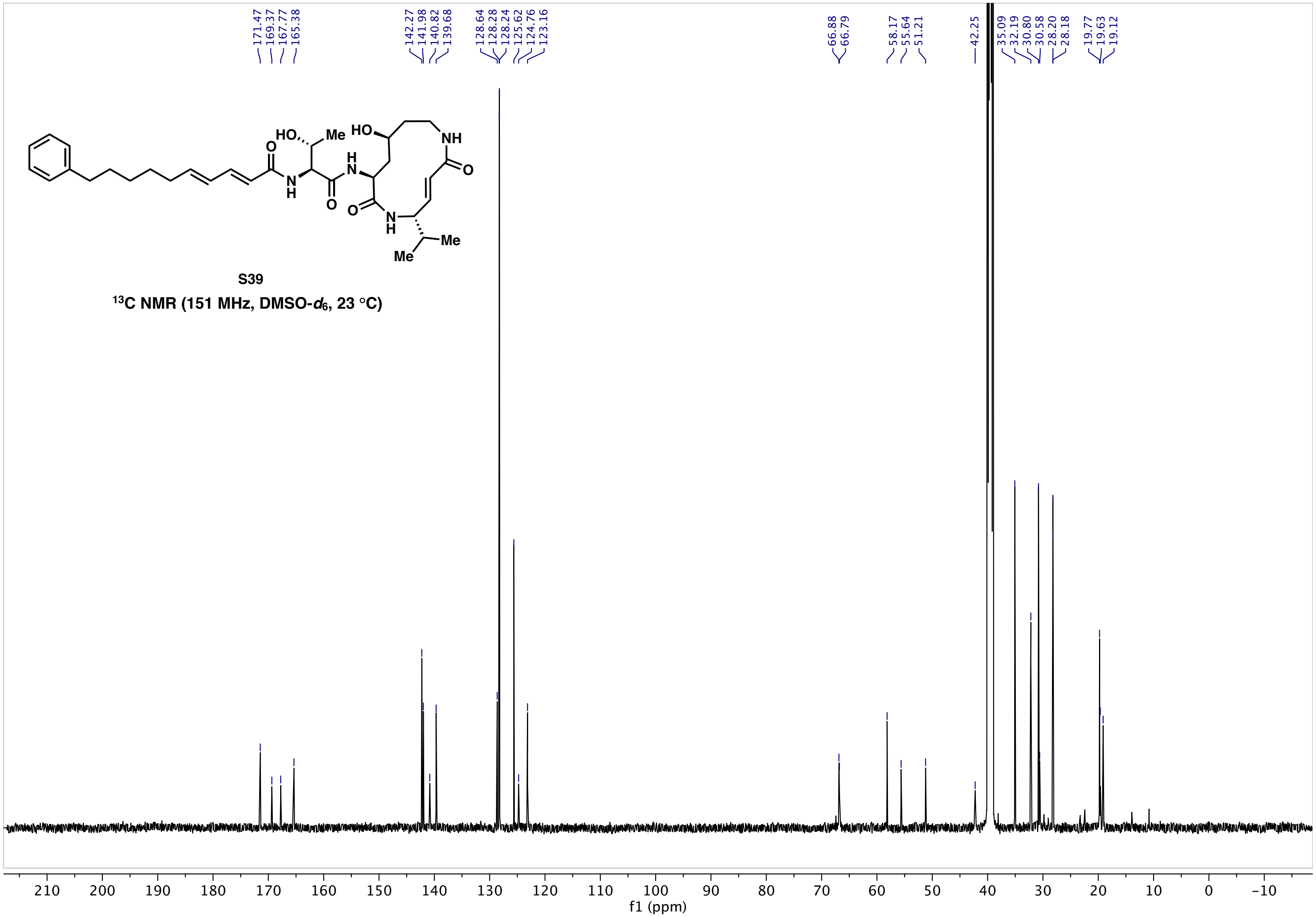
171.47
169.37
167.77
165.38
142.27
141.98
140.82
139.68
128.64
128.28
128.24
125.62
124.76
123.16
66.88
66.79
58.17
55.64
51.21
42.25
35.09
32.19
30.80
30.58
28.20
28.18
19.77
19.63
19.12
HO
Me
HO
NH
O
H
N
O
N
H
O
O
N
H
Me
Me
S39
^{13}C NMR (151 MHz, DMSO-d_6, 23 °C)
210
200
190
180
170
160
150
140
130
120
110
100
90
80
70
60
50
40
30
20
10
0
-10
f1 (ppm)

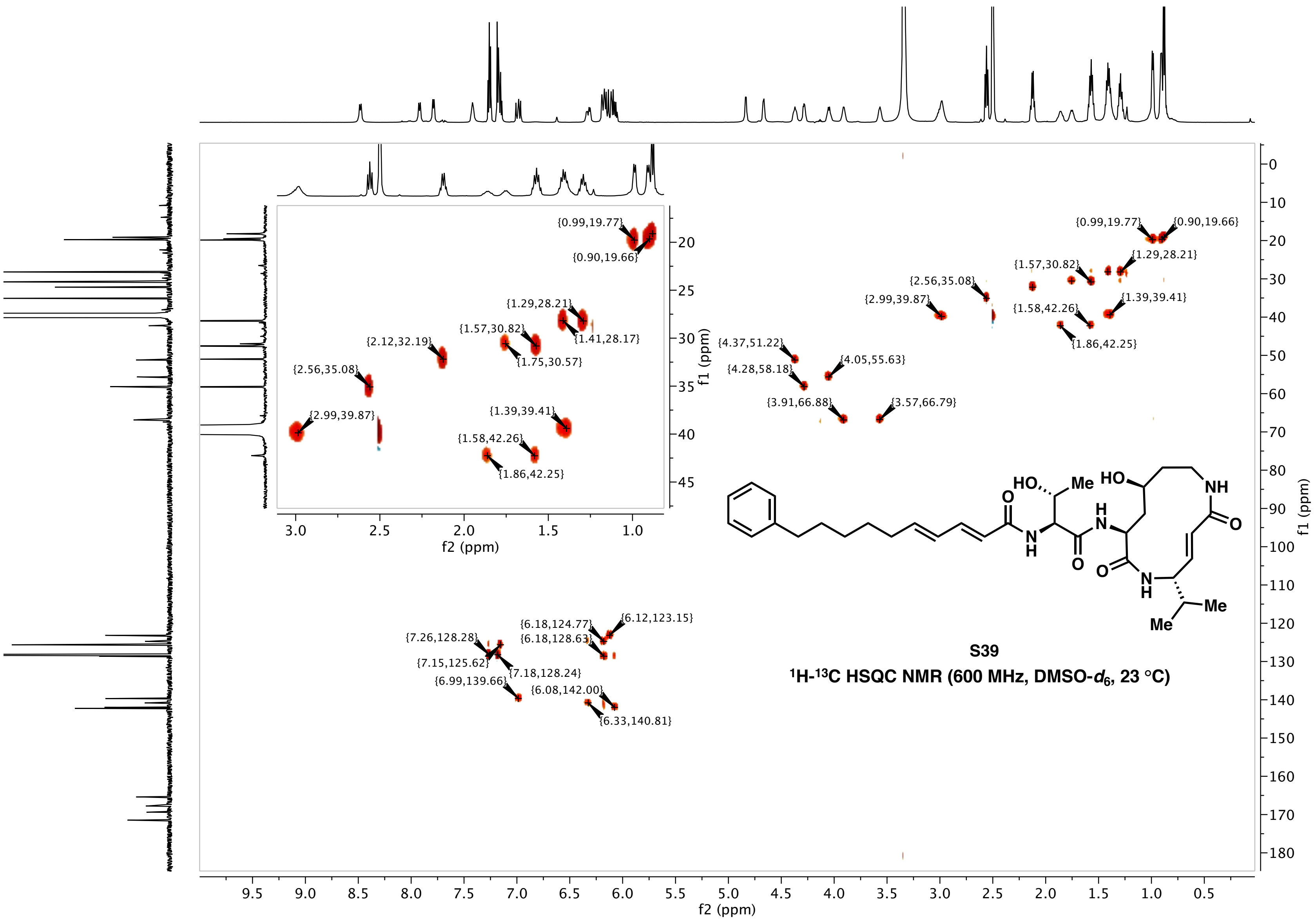

S39

^{1}H-^{13}C HSQC NMR (600 MHz, DMSO-d_6, 23 °C)
