# Supplementary material for: Comprehensive Structure–Activity Relationship Studies of Cepafungin Enabled by Biocatalytic C–H Oxidations
Source: ACS Cent Sci. 2023 Jan 27;9(2):239–51. doi: 10.1021/acscentsci.2c01219 (PMC9951290; doi:10.1021/acscentsci.2c01219)
Supplement: Supplementary file 4 — oc2c01219_si_004.pdf [file oc2c01219_si_004.pdf]

oc-2022-01219g.R1

Name: Peer Review Information for "Comprehensive Structure-Activity Relationship Studies of Cepafungin Enabled by Biocatalytic C–H Oxidations"

First Round of Reviewer Comments

Reviewer: 1

Comments to the Author

This paper describes the chemoenzymatic 9-step synthesis of cepafungins as highly potent and selective eukaryotic proteasome inhibitors with the generation of an additional 13 analogs. They also perform chemoproteomic and quantitative proteomic experiments showing the selectivity of their natural product for proteasome subunits as well as overlapping proteomic changes with bortezomib. Overall, this is a very interesting paper which could be suitable for publication, but this reviewer could not assess some of the data. On page 9, it seems like there's reference to a Figure 1 that should show some gels that speak to potential selectivity data or potentially the chemoproteomics data? It also looks like the authors do not actually show any of the chemoproteomic data or even have a table for the chemoproteomic data. The only tables I could find were for the quantitative proteomics data. The paper should be made to be more presentable.

Reviewer: 2

Comments to the Author

The authors report the development of a chemical synthesis of cepafungin and analogues as well as their in-depth biochemical and biological evaluation. Cepafungin is a member of the syrbactin natural product family that has aroused significant interest due to their potent proteasome inhibition properties. Proteasome inhibition is an established anticancer strategy and to date, three different proteasome inhibitors have received FDA approval. However, due to persisting challenges in their toxicity and efficacy profile, alternative potent and selective proteasome inhibitors are still urgently sought. The present study thus addresses a timely and highly relevant research topic from chemical biology and medicinal chemistry.

Key step in their synthesis is the implementation of an elegant biocatalytic CH oxidation step in an overall ‘classical’ chemical synthesis route that enables an impressive streamlining and, in comparison to previously published syntheses of syrbactins, shortening of their synthesis route. This biocatalytic step is based on a simple nevertheless original ‘trick’: they express the corresponding biosynthetic enzyme from the biosynthesis assembly line and use it, after optimization of reaction conditions, to generate a key hydroxyl lysine intermediate that by ‘classical’ chemical synthesis is achieved only after multiple steps. Indeed, this and further related biocatalytic reactions reported in this study allowed the synthesis

of a library of cepafungin analogues for targeted structure-activity relationship studies. This is an advance to previous publications that due to more complex syntheses have all been limited to a small number of synthetic analogues. The present study is thereby based, but nevertheless represents a significant extension of a previous study from the authors (published in Cell ChemBiol and cited as ref. [35]). The subsequent biochemical and biological data evaluation is state-of-the-art and consisted of biochemical, biological and proteomics-based assays. Overall, I really like the presented synthetic approach and its usage to design and evaluate novel proteasome inhibitors. These are overall very important findings that warrant publication in a prestigious journal such as ACS Central Science.

As the study is also technically very solid and well-written, I have only some minor comments:

1) The authors mention salinosporamide on several occasions (e.g. in the main text or Fig. 1). I might be wrong here but to my knowledge, salinosporamide did not pass the clinical trials related to treatment of multiple myeloma. Instead, it is currently tested in clinical studies vs. glioblastoma.

2) The numbering of Scheme 1 (pg. 4, scheme legend) seems wrong. It has been labeled as Scheme 2.

3) Pg. 5 line 11: Hydroxylation was followed by in situ Boc protection to provide 20 in quantitative yield over two steps. The corresponding Scheme 1B however reports a yield of 62% over two steps?

4) I find Scheme 2B not easy to understand. The authors report a 40% yield for conversion of 32 into 34 (via 4 steps). However, 34 is an intermediate that has not been isolated (according to the brackets?). In addition, they report an NMR yield of 63% for this step – related to which starting material? This (not isolated?) product is then converted to 1 in 33% yield? Please clarify.

5) Pg. 8, line 40. The authors report that 1 binds to beta5, beta2i, beta1, beta2 and alpha5? Alpha5 is no catalytic subunit of the proteasome and thus does not seem right. Which subunit is meant here?

6) The authors discuss their observed structure-activity-relationships in relation to a published X-ray cocrystal structure of Cepafungin with the yeast proteasome. However, they report inhibitory values for the human proteasome (which is naturally the more relevant enzyme variant). Both enzymes are highly homologues but not identical. This should perhaps be mentioned and considered in the discussion of their inhibition results.

7) I am surprised that derivative 51 (with the CF3 residue) has been completely inactive as even analogues without any lipid chain usually retain some proteasome inhibitory properties. Did the authors observe any issues with this compound, e.g. low solubility that may explain this complete loss of activity?

8) Fig. 4. Could the authors please add the information on the number of biological(?) replicates used in each experiment (n=?). The depicted errors represent standard deviation?

9) The proteomics studies (e.g. in Fig. 5) but also the pulldown with S10 give important information on the molecular mode-of-action of the synthesized compounds. Please note that there has been a previous gel-based target selectivity profiling with a syrbactin probe (reported in Chembiochem 2009, 10, 2638). The present pulldown based on quantitative MS is obviously much more advanced but this reference might be worth mentioning. I also missed the link (and data in the SuppInfos?) to the competitive ABPP approach between Cepafungin (1) and S10 reported in the main text.

10) Just as an ‘alternative’ thought for the reported structure-activity-relationship studies: The authors have investigated the impact of the hydroxyl moiety on the lysine residue for inhibition (by also synthesizing the corresponding epimer or oxidizing the hydroxyl moiety, etc.). Differences in the inhibition profile are then related to differing overall ring strain (that favors nucleophilic attack to the enamide) or additional interactions with the proteasome. In my view, a third factor might also play a role and that is the impact of the hydroxyl group on the overall folding and thus structural preorganization for binding of the medium ring system (which is a difficult issue in medium-sized macrocycles). Although not taking part in direct interactions, the arrangement of the hydroxyl moiety might select or stabilize certain ring conformations that better fit to the substrate binding site, thus resulting in more efficient inhibition.

Author's Response to Peer Review Comments:

Prof. Editor
Editor, ACS Central Science

Dear Prof. Editor,

We are resubmitting a revised version of our manuscript entitled "Comprehensive Structure-Activity Relationship Studies of Cepafungin Enabled by Biocatalytic C–H Oxidations" for publication as a Research Article in ACS Central Science. An accompanying Supporting Information, which contains experimental procedures and compound characterization, and two supplementary tables (Tables S3 and S5), which contain our full proteomics data sets, can also be found in the submission. An initial version of the manuscript was submitted to ACS Central Science on October 13, 2022 and was evaluated with the assistance of two reviewers. The reviewers' comments are generally positive, though Reviewer 1 requested for additional chemoproteomic data and Reviewer 2 raised several minor comments that need addressing. To address these concerns, we have carefully revised the manuscript accordingly. The biggest change to the manuscript is the addition of competitive pulldown proteomics data that are provided in a spreadsheet format in Table S3 and represented as a volcano plot in Figure S1 in the Supporting Information. We have also addressed the editorial comments by shortening the abstract and adding Supporting Information statement, TOC graphic and synopsis. In addition, we are supplying a version of the manuscript with tracked changes, which are highlighted in yellow. Below please find our point-by-point response to the reviewers' comments.

Reviewer: 1

Recommendation: Reconsider after major revisions noted.

Comments:
This paper describes the chemoenzymatic 9-step synthesis of cepafungins as highly potent and selective eukaryotic proteasome inhibitors with the generation of an additional 13 analogs. They also perform chemoproteomic and quantitative proteomic experiments showing the selectivity of their natural product for proteasome subunits as well as overlapping proteomic changes with bortezomib. Overall, this is a very interesting paper which could be suitable for publication, but this reviewer could not assess some of the data. On page 9, it seems like there's reference to a Figure 1 that should show some gels that speak to potential selectivity data or potentially the chemoproteomics data? It also looks like the authors do not actually show any of the chemoproteomic data or even have a table for the chemoproteomic data. The only tables I could find were for the quantitative proteomics data. The paper should be made to be more presentable.

Response:

We thank the reviewer for the assessment and apologize for this omission. The competitive pulldown proteomics data is now shown as a volcano plot in Figure S1 and the corresponding full dataset is listed in Table S3.

Additional Questions:

Quality of experimental data, technical rigor: Moderate

Significance to chemistry researchers in this and related fields: High

Broad interest to other researchers: High

Novelty: High

Is this research study suitable for media coverage or a First Reactions (a News & Views piece in the journal)?: No

Reviewer: 2

Recommendation: Publish in ACS Central Science after minor revisions noted.

Comments:
The authors report the development of a chemical synthesis of cepafungin and analogues as well as their in-depth biochemical and biological evaluation. Cepafungin is a member of the syrbactin natural product family that has aroused significant interest due to their potent proteasome inhibition properties. Proteasome inhibition is an established anticancer strategy and to date, three different proteasome inhibitors have received FDA approval. However, due to persisting challenges in their toxicity and efficacy profile, alternative potent and selective proteasome inhibitors are still urgently sought. The present study thus addresses a timely and highly relevant research topic from chemical biology and medicinal chemistry.

Key step in their synthesis is the implementation of an elegant biocatalytic CH oxidation step in an overall ‘classical’ chemical synthesis route that enables an impressive streamlining and, in comparison to previously published syntheses of syrbactins, shortening of their synthesis route. This biocatalytic step is based on a simple nevertheless original ‘trick’: they express the corresponding biosynthetic enzyme from the biosynthesis assembly line and use it, after optimization of reaction conditions, to generate a key hydroxyl lysine intermediate that by ‘classical’ chemical synthesis is achieved only after multiple steps. Indeed, this and further related biocatalytic reactions reported in this study allowed the synthesis of a library of cepafungin analogues for targeted structure-activity relationship studies. This is an advance to previous publications that due to more complex syntheses have all been limited to a small number of synthetic analogues. The present study is thereby based, but nevertheless represents a significant extension of a previous study from the authors (published in Cell ChemBiol and cited as ref. [35]). The subsequent biochemical and biological data evaluation is state-of-the-art and consisted of biochemical, biological and proteomics-based assays. Overall, I really like the presented synthetic approach and its usage to design and

evaluate novel proteasome inhibitors. These are overall very important findings that warrant publication in a prestigious journal such as ACS Central Science.

Response:

We thank the reviewer for the kind assessment of the manuscript.

As the study is also technically very solid and well-written, I have only some minor comments:

1) The authors mention salinosporamide on several occasions (e.g. in the main text or Fig. 1). I might be wrong here but to my knowledge, salinosporamide did not pass the clinical trials related to treatment of multiple myeloma. Instead, it is currently tested in clinical studies vs. glioblastoma.

Response:

We thank the reviewer for correcting this inaccurate statement. The error has been corrected in the revised manuscript.

2) The numbering of Scheme 1 (pg. 4, scheme legend) seems wrong. It has been labeled as Scheme 2.

Response:

We have changed the scheme numbering accordingly.

3) Pg. 5 line 11: Hydroxylation was followed by in situ Boc protection to provide 20 in quantitative yield over two steps. The corresponding Scheme 1B however reports a yield of 62% over two steps?

Response:

The 62% yield is cited from an earlier attempt using unoptimized conditions. In this case, the one-pot Boc protection was stopped at ~70% conversion (LCMS) and some product was likely lost during aqueous workup. In the optimized conditions, the two-step sequence gave essentially quantitative yield on 16 mmol scale. This discrepancy has been rectified in the revised manuscript. In addition, we have provided below two notebook scans to verify our results (notebook page 41 from 9/14/17 reflects the unoptimized conditions and notebook scan page 68, 72–73 from 7/11/21 reflects the optimized conditions.

Project

Continued from Page

9/14/17 Procedure AA1-6

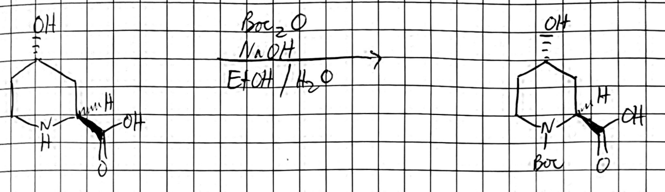

145.2 g/mol — 245.28 g/mol

15 mmol ; 1 eq

1 pot from FoPip4H rxn

Boc_2O : 218.25 g/mol ; 2.5 eq ; 37.5 mmol ; 8.184 g

NaOH : 2M NaOH added until pH ~10 (final aq V = ~400 mL)

EtOH : run in 2:1 H_2O : EtOH → 200 mL EtOH

Supernatant of FoPip4H rxn basified to pH 10 w/ 2M NaOH, then added Boc_2O in EtOH. Rxn set @ 7:30 PM. (note: rxn mixture darkened from yellow → red → ~~orange~~ red/black upon basification).

LC-MS ON (t = 14 hrs) → ~2.7 : 1 Pr : S.M.

Next time conc. aq. layer before Boc protection?

Acidified to pH ~2 w/ $KHSO_4$, evaporated ~~EtOH and to dryness~~ to ~50 mL aq remaining. pH → 1 w/ $KHSO_4$ not possible w/o large vol., switched to 1M HCl to pH ~1 → vol @ ~100+ mL aq. Extracted w/ 300 mL EtOAc, checked TLC. Did another 2x 125 mL EtOAc extraction. Washed w/ 6:6 mL brine : 1M HCl mixture. Dried over $MgSO_4$, conc'd. 100 mL RB t = 83.8989 g crude yield: 4.345 g

Column: 1.5" x 6" SiO_2 ; 300 mL @ 95:5:0.2 DCM : MeOH : AcOH

finish w/ 90:10:0.2 DCM : MeOH : AcOH

collected fr. 19-26 w/ toluene azeotrope — yield: ~~[illegible]~~ 2.282 g — 1-41-33 (62%)

100 mL RB t = 84.7610 g — NMR → some impurities...

• purify after TBS protection — 1-45 →

• starting material recovered by ion exchange... 100 mL t = 83.45 g

recovered ~0.55 g SM, Boc protection 1-48 →

↳ 25.3%

Continued on Page

Read and Understood By

Signed Date Signed Date

Continued from Page

7/11/21 FoPip 4H re-do for chain

Cell stock of FoPip4H into 4ml LB + kan, set 250 RPM / 37°C @ 8:30 AM.

1x 500ml TB prepared (w/ 4ml 50% glycerol ~~[illegible]~~). (23.8g TB).

7/12/21: 2.5ml starter to 500ml TB @ 10:45AM. Set 250 RPM @ 37°C. At 2.5 hrs → OD ≈ 0.7. Media onto ice for 15min.

Added 500μl of 25mM IPTG, set 250 RPM / 20°C @ 2PM

7/13/21: Spun down 15 min @ 4200 RPM, resuspended to OD ≈ 30 w/ pH 7 KPi, (A) 100ml → OD ≈ 0.28, (B) 100ml → OD = 0.26

Lysed in 1x 200ml batch, 50% A w/ wide tip, 3 min (1s on, 4s off). Stirred then + 1 min sonics. Final OD = 15. (Dilute 1:1 w/ KPi for rxn).

Set 2x 200ml rxns.

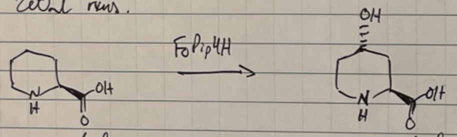

129.2 g/mol → 145.2 g/mol

40mM; 8 mmol / rxn

1.034 g / rxn (total: 2.067g)

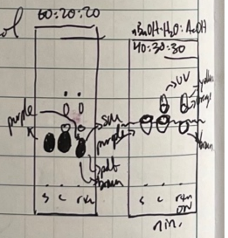

αKG: 226.13 g/mol; 2 eq; 16 mmol; 3.618g

asc: 176.12 g/mol; 0.5 eq; 4 mmol; 704mg

$FeSO_4$: 278.01 g/mol; 0.05 eq; 0.4 mmol; 111mg

22°C / ~~250~~ ~~200~~ 200 RPM O/N. Set @ 1:45 PM. ~8 AM LCMS chk. (1:1 1M HCl quench / spun down) → incomplete, significant S.M. 7/14/21 AM LCMS → still incomplete. Rxn to pH ~2 w/ 6M HCl, spun down, 15 min @ 4200 RPM. ~~+~~ note: TLC → rxn looks ~~complete~~ mostly complete. Air-dried over day...

Continued on Page 72

Read and Understood By

Signed Date Signed Date

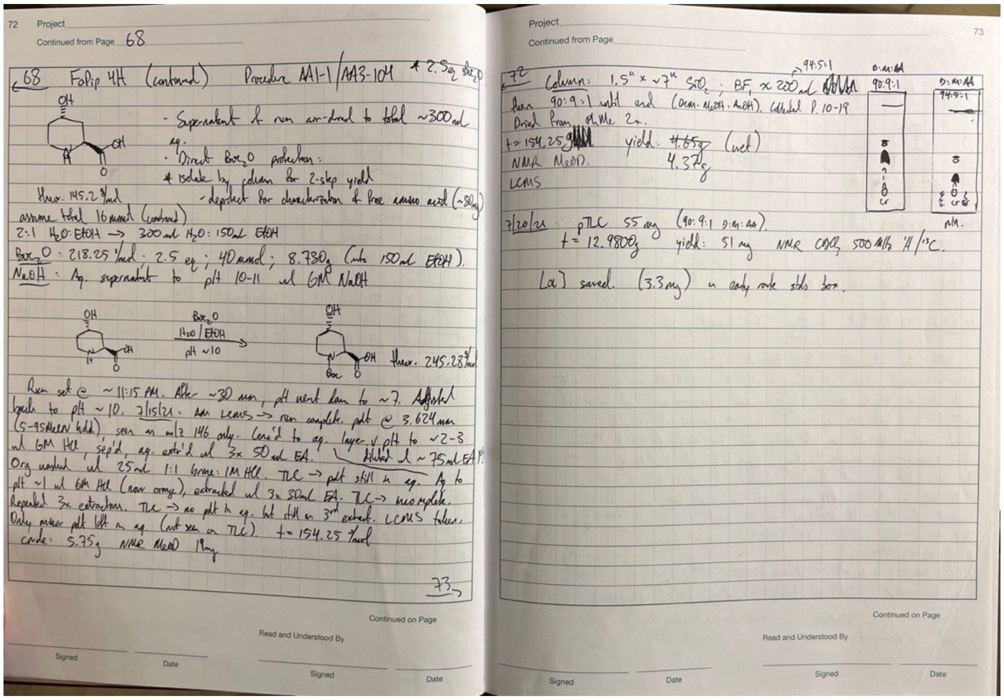

4) I find Scheme 2B not easy to understand. The authors report a 40% yield for conversion of 32 into 34 (via 4 steps). However, 34 is an intermediate that has not been isolated (according to the brackets?). In addition, they report an NMR yield of 63% for this step – related to which starting material? This (not isolated?) product is then converted to 1 in 33% yield? Please clarify.

Response:

From 32, LAH reduction produces an alpha-chiral aldehyde that epimerizes easily on silica with or without basic neutralization. The aldehyde is obtained in crude form by aqueous workup and carried forward through Wittig olefination for a 2-step isolated yield of 67%. Global deprotection in TFA and purification by precipitation in ether provides the macrocyclization precursor in 94% yield. Cyclization of this compound with DMTMMT produces the desired macrolactam in 63% assay yield by NMR, using p-toluenesulfonamide as internal standard. The product is only semi-purified by precipitation and directly used in the final fragment coupling. The crude macrocycle is stable as a solid for several months when stored at -20 °C. Fragment coupling provides the natural product in 33% isolated yield, with respect to the mmol of 34 starting material (determined by NMR) being used.

5) Pg. 8, line 40. The authors report that 1 binds to beta5, beta2i, beta1, beta2 and alpha5? Alpha5 is no catalytic subunit of the proteasome and thus does not seem right. Which subunit is meant here?

Response:

The enrichment of the non-catalytic subunit PSMA5 was indeed unexpected. We do not believe that PSMA5 was directly bound by the cepafungin alkyne probe but was rather indirectly enriched in a complex with one of the PSMB subunits.

6) The authors discuss their observed structure-activity-relationships in relation to a published X-ray cocrystal structure of Cepafungin with the yeast proteasome. However, they report inhibitory values for the human proteasome (which is naturally the more relevant enzyme variant). Both enzymes are highly homologues but not identical. This should perhaps be mentioned and considered in the discussion of their inhibition results.

Response:

We thank the reviewer for pointing this out. The statement has been revised to state that in the absence of the cocrystal structure of cepafungin with human proteasome, the structure of cepafungin:yeast proteasome complex was used as reference due to the high homology and general structural conservation between the yeast and human proteasomes.

7) I am surprised that derivative 51 (with the CF3 residue) has been completely inactive as even analogues without any lipid chain usually retain some proteasome inhibitory properties. Did the authors observe any issues with this compound, e.g. low solubility that may explain this complete loss of activity?

Response:

No issues were noted during the synthesis or final purification of analog 51. NMR and LCMS purity is consistent with all other final compounds after HPLC. It should be noted that our proteasome inhibition and cytotoxicity assays will not account for various pharmacokinetics parameters such as cell permeability, which might contribute to the loss of activity here. However, the verification of this hypothesis will fall beyond the scope of the present work.

8) Fig. 4. Could the authors please add the information on the number of biological(?) replicates used in each experiment (n=?). The depicted errors represent standard deviation?

Response:

All experiments were performed in biological triplicate (n=3) and the depicted error bars represent standard deviation. We now specify the number of biological replicates and explain the nature of the error bars in Fig. 4, Fig. S2 and Fig. S3.

9) The proteomics studies (e.g. in Fig. 5) but also the pulldown with S10 give important information on the molecular mode-of-action of the synthesized compounds. Please note that there has been a previous gel-based target selectivity profiling with a syrbactin probe (reported in Chembiochem 2009, 10, 2638). The present pulldown based on quantitative MS is obviously much more advanced but this reference might be worth mentioning. I also missed the link (and data in the SuppInfos?) to the competitive ABPP approach between Cepafungin (1) and S10 reported in the main text.

Response:

We thank Reviewer 2 for drawing our attention to the omitted important citation. The mentioned Chembiochem publication is now cited in our manuscript as ref. 40. The competitive pulldown proteomics data is now shown as a volcano plot in Figure S1 and the corresponding full dataset is listed in Table S3.

10) Just as an 'alternative' thought for the reported structure-activity-relationship studies: The authors have investigated the impact of the hydroxyl moiety on the lysine residue for inhibition (by also synthesizing the corresponding epimer or oxidizing the hydroxyl moiety, etc.). Differences in the inhibition profile are then related to differing overall ring strain (that favors nucleophilic attack to the enamide) or additional interactions with the proteasome. In my view, a third factor might also play a role and that is the impact of the hydroxyl group on the overall folding and thus structural preorganization for binding of the medium ring system (which is a difficult issue in medium-sized macrocycles). Although not taking part in direct interactions, the arrangement of the hydroxyl moiety might select or stabilize certain ring conformations that better fit to the substrate binding site, thus resulting in more efficient inhibition.

Response:

We thank the reviewer for the suggestion. While prior crystallographic evidence (refs. 5a and 12) suggests that there is no significant difference in macrocyclic conformation between cepafungin and its desoxy variant and that such difference could only be observed upon the introduction of additional unsaturation in the macrocycle (ie. syringolin A), it is still possible that alternative oxidation patterns could induce a shift in conformation, which in turn will lead to different fit in the binding site. Though our results eventually show that analogs 40–42 are inferior to cepafungin itself, we agree with the reviewer that this would be a viable initial hypothesis to motivate the investigation of these analogs. This discussion has now been added to the manuscript, and the relevant section now reads:

"In addition to the aforementioned interactions, it is also possible that the introduction of non-natural oxidation patterns might induce alternative ring conformations that might lead to superior binding in the active site. In prior studies, the conformations of proteasome-bound syringolin B and glidobactin A were noted to be almost identical but showed marked differences to that of proteasome-bound syringolin A."

Additional Questions:

Quality of experimental data, technical rigor: Top 5%

Significance to chemistry researchers in this and related fields: Top 5%

Broad interest to other researchers: Top 5%

Novelty: Top 5%

Is this research study suitable for media coverage or a First Reactions (a News & Views piece in the journal)?: Yes

We hope that the revisions have been made to your satisfaction and we thank you for taking the time to evaluate this work.

Sincerely,

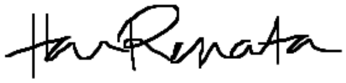

Hans Renata, Ph.D.

Associate Professor, Department of Chemistry

Rice University
